# Formation Mechanism of Key Flavor Compounds During the Fermentation of Strawberry Juice with Water Kefir Grains

**DOI:** 10.3390/foods15081312

**Published:** 2026-04-10

**Authors:** Linlin Yin, Shunchang Pu, Qianqian Tong, Zhina Chen, Tao Ye, Shoubao Yan

**Affiliations:** 1 School of Biological Engineering, Huainan Normal University, Huainan 230038, China; yinlinlin@hnnu.edu.cn (L.Y.); tongqianqian@hnnu.edu.cn (Q.T.); chenzhina@hnnu.edu.cn (Z.C.); yetao@hnnu.edu.cn (T.Y.); 2 Department of Biology and Food Engineering, Bozhou University, Bozhou 236800, China; pushunchang@bzuu.edu.cn

**Keywords:** water kefir, strawberry juice, fermentation, flavor compounds, dominant flora

## Abstract

Water kefir grains are complex probiotic granules that can efficiently ferment fruit and vegetable juices and significantly improve product flavor. However, the mechanisms of flavor formation remain unclear, which limits the process optimization of this technology. This study investigated the mechanisms involved in flavor formation during the fermentation of strawberry juice with water kefir grains. The results showed that as fermentation progressed, the total acidity increased, whereas the pH value and soluble solids content decreased. Additionally, the contents of citric acid and malic acid gradually decreased with fermentation, while the contents of lactic, acetic, and succinic acid increased, and three soluble sugars showed reduced levels. A total of 218 volatile compounds were identified. Eight dominant bacterial genera and one dominant yeast species were detected. Significant correlations between some key microorganisms and flavor compounds were observed. Specifically, *Lactiplantibacillus* was positively correlated with hexyl acetate. Meanwhile, *Gluconobacter* and *Acetobacter* were positively correlated with methyl (Z,Z)-9,12-octadecadienoate, isoamyl acetate, etc. In contrast, LAB such as *Lacticaseibacillus* and *Schleiferilactobacillus* showed the opposite correlations with these key flavor compounds. *Saccharomyces* showed a positive correlation with ethyl palmitate, ethyl propionate, phenylsuccinic acid, and 1-pentanol. The main flavor compound metabolic pathways were predicted and they were significantly related with yeasts, acetic acid bacteria, and lactic acid bacteria. Overall, this study offers a theoretical basis for the directional regulation and optimization of the flavor quality of strawberry juice fermented with water kefir.

## 1. Introduction

Water kefir grains are translucent gel-like granules formed through the symbiotic association of various microorganisms, including lactic acid bacteria (LAB), acetic acid bacteria (AAB), and yeasts [1]. Their fermented products have diverse functions, which include regulating intestinal flora, antibacterial activity, and immune regulation [2,3,4]. With the continuous growth of global consumer demand for plant-based products (approximately 10% annually), water kefir, a fermented sugar–water matrix with water kefir grains, has significant market potential as an ideal dairy alternative [5]. Currently, research on water kefir-fermented fruit and vegetable juices has made certain progress. Studies have shown that water kefir grains can successfully ferment a variety of fruit and vegetable juice matrices, including plum, pear, mango, and coconut juice [6,7,8]. This fermentation significantly alters the physicochemical properties, microbial composition, and biological activity of the juices [9]. Current research on the application of water kefir grains in the fermentation of various fruit and vegetable juices mainly focuses on process optimization, quality changes during storage, and macro-characterization of final products.

Flavor is the core factor that determines the acceptability of fermented foods. The flavor formed through water kefir fermentation is the result of the synergistic metabolism of microorganisms. Studies have shown that the early stage of water kefir fermentation is typically dominated by yeast-driven alcoholic fermentation, followed by a stage of organic acid production involving *Lentilactobacillus hilgardii* and *Acetobacter tropicalis* [10]. One study identified 41 volatile compounds in water kefir fermentation broth and found that specific microbial groups are closely associated with key volatile compounds [11]. Furthermore, the synergistic interaction among microorganisms directly affects the formation of key flavor esters such as ethyl octanoate, ethyl decanoate, and isoamyl acetate [12].

Strawberries are rich in vitamin C, phenols, flavonoids, and natural sugars; however, this fresh fruit has a short shelf life and is susceptible to rotting. Deep processing is therefore an effective strategy to increase their added value and reduce resource waste [13]. Previous study demonstrated that water kefir grains used to ferment strawberry juice significantly enhance the biological activity of the fermented juice [14]. Building on this, our preliminary research team further found that fermenting strawberry juice with water kefir grains not only produced products with outstanding sensory characteristics and high overall acceptability but also exhibited good storage stability under refrigerated conditions [5]. However, despite the promising application of this potential fermentation system, the specific mechanism and key metabolic pathways involved in the formation of unique flavors are still unclear. This, to a certain extent, restricts the targeted design and quality regulation of related products.

The dynamic interplay between the food raw material matrix and the microbial community governs the succession and transformation of flavor compounds throughout the fermentation process. In the strawberry juice system, abundant soluble sugars such as fructose, glucose, and sucrose provide the main carbon source for microbial growth. These sugars can be metabolized into organic acids such as lactic acid and acetic acid, as well as volatile flavor compounds including esters and aldehydes. However, in this complex fermentation process, several key aspects remain unclear. These include the dynamic patterns of sugar consumption, and organic acid profiles at different fermentation stages, the evolution pathways of volatile flavor compounds, and the relationship between these changes and microbial community structure. In addition, the key microorganisms driving the formation of specific flavor compounds remain unidentified. For the water kefir grains fermented strawberry juice system, these questions still lack definitive answers.

This study systematically investigated the mechanisms involved in flavor formation during the fermentation of strawberry juice with water kefir grains. Initially, the dynamic changes in microbial community structure and major flavor compounds during the fermentation of strawberry juice with water kefir grains were systematically explored. Metagenomic technology was then used to analyze the dynamic succession of bacterial and fungal communities at the genus/species level. High-performance liquid chromatography (HPLC) was used to analyze the metabolic changes of main organic acids and soluble sugars, and gas chromatography–mass spectrometry (GC-MS) was combined to identify key volatile flavor compounds. To further identify the key microbial groups that are closely related to the formation of specific flavors, Spearman correlation analysis was used. The results of this study can provide a theoretical reference for the directional regulation of the flavor of kefir fermented fruit and vegetable juice beverages.

## 2. Materials and Methods

### 2.1. Preparation of Water Kefir Grains and Strawberry Juice

Water kefir grains were purchased from a local supplier (Hefei, Anhui Province, China) and activated in a sterilized 10% brown sugar (Ganzheyuan, Nanjing, China) solution at 25 °C for 12 h. Following filtration, the grains were rinsed with sterile distilled water, and this procedure was repeated three times. Strawberries were purchased from a local fruit market (Huainan, Anhui Province, China; variety: Hongyan, SSC = 12°Brix, titratable acidity = 0.8 g/100 g).

Fresh strawberries were rinsed with distilled water and then blended at a mass ratio of 2:1 (strawberry:water). To this pulp, 0.05% ascorbic acid, and 0.2% mixture of pectinase (30,000 U/g) and cellulase (10,000 U/g) in a 3:1 mass ratio (pectinase:cellulase) (Wanbang, Zhengzhou, China) were added. After thorough mixing, the pulp was sealed and incubated in a water bath at 42 °C for 35 min, then rapidly cooled and filtered with a 200-mesh filter cloth. The final resultant pulp was sterilized using heat treatment at 85 °C for 20 min and cooled to obtain sterile strawberry juice.

### 2.2. Preparation of Samples at Different Fermentation Stages

**Figure 1:**
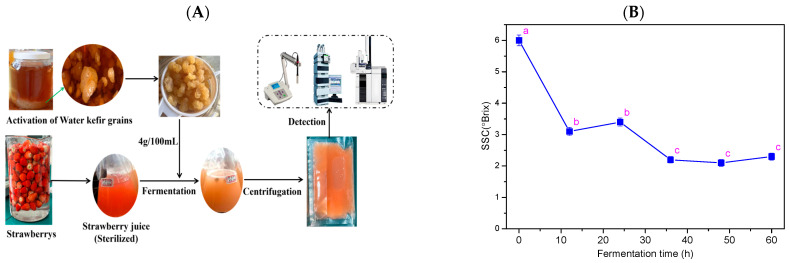

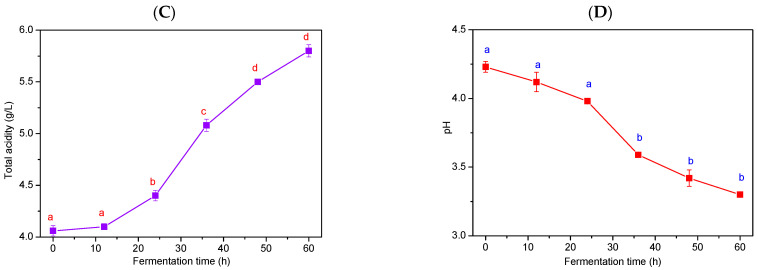
Changes in soluble solid contents (SSC) (**B**), titratable acidity (**C**), and pH (**D**) of strawberry juice fermented (**A**) with water kefir grains. (Note: Values within the same parameter followed by different lowercase letters are significantly different (*p* < 0.05)).

Activated water kefir grains were aseptically inoculated into sterilized strawberry juice samples (strawberry juice:sterile sugar water (10%) = 6:4, *v*/*v*; inoculum size = 4 g/100 mL). The samples were collected immediately and recorded as 0 h samples. The inoculated strawberry juice was incubated at 25 °C, and samples were collected at the following fermentation time points: 12, 24, 36, 48, and 60 h. After sampling, all samples were filtered under sterile conditions to remove water kefir grains, and the fermented strawberry juice was collected and stored at −80 °C for subsequent analysis (Figure 1A).

### 2.3. Determination of SSC, Total Acidity, and pH

The pH value was measured using a pH meter (PHS-3C, Shanghai Yueping Scientific Instrument Manufacturing Co., Ltd., Shanghai, China). The soluble solids content (SSC, refers to the total concentration of dissolved compounds in water, such as sugars and acids) was measured using an Abbe refractometer (WAY-80, Shandong Haizhou Mining Safety Equipment Co., Ltd., Tai’an, China) and expressed as °Brix. Total titratable acidity was determined by potentiometric titration according to GB/T 12456-2021 [15].

### 2.4. Determination of Organic Acids in Fermented Strawberry Juice

The main organic acids were determined through HPLC (1260, Agilent, Santa Clara, CA, USA). The samples were centrifuged at 10,766× *g* for 10 min (TGL-16M, Hunan Xiangyi Laboratory Instrument Development Co., Ltd., Changsha, China), filtered through a 0.22 μm microporous membrane, and then analyzed by HPLC. The chromatographic conditions were as follows: Agilent C18 AQ chromatographic column (4.6 mm × 250 mm, 5 μm), injection volume of 10 μL, detection wavelength of 210 nm, and a flow rate of 0.6 mL/min.

The mobile phase consisted of (A) 0.1% phosphoric acid aqueous solution and (B) methanol. The gradient elution program was as follows: (i) For A—97.5%, 97.5%, 0%, 0%, 97.5%, and 97.5%; (ii) For B—2.5%, 2.5%, 100%, 100%, 2.5%, and 2.5%. The retention times were set to 0, 13, 13.1, 18, 18.1, and 25 min, respectively [16]. The quantification was performed with reference to GB 5009.157-2016 [17].

### 2.5. Determination of Soluble Sugars in Fermented Strawberry Juice

Using the HPLC method, the soluble sugars were detected in the samples of fermented strawberry juice (1200, Agilent, USA). The samples were ultrasonically treated for 30 min (800 W, 40 kHz), filtered through a 0.45 μm microporous membrane, and then analyzed. The chromatographic conditions were as follows: an amino column (Agilent, 250 × 4.6 mm, 5 μm), differential refractive detector, column temperature of 35 °C, injection volume of 10 μL, mobile phase of acetonitrile:water = 70:30 (*v*/*v*), and a flow rate of 1.0 mL/min. The results were calculated with reference to GB 5009.8-2023 [18].

### 2.6. Determination of Volatile Flavor Compounds

Volatile flavor compounds in fermented strawberry juice were extracted using headspace solid-phase microextraction (HS-SPME) and analyzed through GC-MS (7890B-7000D, Agilent, USA). Samples were placed in a 20 mL headspace vial, to which an appropriate amount of saturated sodium chloride and the internal standard 2-octanol (3 mg/L) was added. The vials were heated at 80 °C for 30 min, after which the SPME fiber needle was inserted into the headspace vial and heated for 30 min. Subsequently, the fiber was desorbed in the GC injection port at 250 °C for 5 min.

The GC-MS conditions were as follows: chromatographic column, HP-5MS (30 m × 0.25 mm × 0.25 μm); column temperature: initial temperature 50 °C for 2 min, increased to 180 °C at 5 °C/min for 5 min, then increased to 250 °C at 10 °C/min for 5 min; injection port temperature: 250 °C; transfer line temperature: 280 °C; carrier gas flow rate: 1.0 mL/min; injection mode: split less; The MS conditions were as follows: electron ionization (EI) source, ion source temperature 230 °C, quadrupole temperature 150 °C, full scan mode in the range of *m*/*z* 40-600 [19].

According to the characteristic ions of compounds in the total ion current diagram, the NIST20 library was used for retrieval to determine the chemical composition of volatile compounds, and the relative content was calculated using the internal standard method.

### 2.7. Analysis of Microbial Community Structure

Metagenomic technology was used to analyze the microbial community structure. First, the total microbial DNA in fermented strawberry juice was extracted by the CTAB method for environmental microbial DNA extraction. The integrity, purity, and concentration of the extracted microbial genomic DNA were determined by 1% agarose gel electrophoresis, NanoDrop spectrophotometry, and Qubit 2.0 fluorometry, respectively. Following library construction and quality assessment, different libraries were pooled into the flow cell according to the effective concentration and target offline data volume requirements. Cluster generation was performed using cBOT, and sequencing was performed on an Illumina PE150 (Illumina, Inc., San Diego, CA, USA) (2 × 150) high-throughput platform.

The raw sequencing data were processed following the procedures described by Bolger et al. [20] and Langmead et al. [21], and quality control and host sequence removal were performed. This included the removal of adapter sequences (parameter ILLUMINACLIP: adapters_path: 2: 30: 10); scanning sequences using a 4 bp sliding window, in which the sequences were truncated when the average quality score fell below 20 (parameter SLIDINGWINDOW:4:20); and the removal of sequences with a final length of less than 50 bp (parameter MINLEN:50), to obtain effective sequences for subsequent analysis. Kraken2 (2.0.7-beta) was used to annotate and classify all effective sequences from all samples, and Bracken was applied to perform Bayesian re-estimation of abundance based on Kraken2 classification results, to estimate the genus-level or species-level abundances of metagenomic samples. Subsequently, the quality-controlled and host-depleted sequences were aligned against the UniProt UniRef90 protein database using HUMAnN3 software with the DIAMOND aligner. Reads that failed to align were filtered out with the default HUMAnN3 parameters: translated_query_coverage_threshold = 90.0, prescreen_threshold = 0.01, evalue_threshold = 1.0, translated_subject_coverage_threshold = 50.0. The relative abundance of each UniRef90 protein was then quantified. Based on the correspondence between UniRef90 IDs and KEGG functional IDs, the abundances of genes (UniRef90) belonging to the same functional category were summed to obtain the relative abundance of corresponding functions in the functional database. In present study, the data output quality, including the total reads and read counts before and after filtering for each sample of the community compositions analysis, is shown in Supplementary Table S1. Additionally, the list of analyzed taxonomies, along with their OTUs, and the GenBank accession number of the community compositions analysis of the fermented strawberry juice samples are provided in Supplementary Table S2.

### 2.8. Data Analysis

For statistical analysis and plotting of the main physicochemical indices of strawberry juice at different fermentation stages, the SPSS17.0 software (IBM, Armonk, NY, USA) and Origin8.0 software (OriginLab, Northampton, MA, USA) were used. Duncan’s multiple range test was applied for significant difference analysis, with *p* < 0.05 considered statistically significant (indicated by different letters). SIMCA 14.0 software (Umetrics, Umeaa, Sweden) was used for partial least squares discriminant analysis (PLS-DA) of volatile substances and screening of important contributing substances (Variable Importance in Projection, VIP > 1). The Shanghai Micromethod Bioinformatics Data Platform was used for microbial community structure and differential analysis, as well as heat map analysis of microorganisms and flavor compounds. Each sample was analyzed in three technical replicates for all determinations.

## 3. Results and Discussion

### 3.1. Changes of SSC During Fermentation

To evaluate the fermentation process, the dynamic changes in the SSC throughout the fermentation of fruit and vegetable juices are an important factor. The fermentation process could be divided into three distinct stages: the early stage (0–24 h), the middle stage (24–36 h), and the late stage (48–60 h). The SSC of strawberry juice samples exhibited an overall decreasing trend throughout the fermentation. Notably, the SSC decreased significantly during the intervals of 0–12 h and 24–36 h, after which the values remained stabilized (Figure 1B). The observed decrease in SSC can be attributed to the metabolic activity of the microbial community throughout the fermentation process. Microorganisms actively utilize soluble components—particularly sugars—as carbon and energy sources, leading to their gradual depletion. Concurrently, the microbial metabolism of these sugars promotes the accumulation of byproducts, notably organic acids, thereby altering the physicochemical profile of the system.

### 3.2. Changes in Total Acidity and pH During Fermentation

The pH and total acidity of fruit juice are the key indicators that reflect the changes in acidity and can also be used to infer the metabolic condition of microorganisms during the fermentation process. The total acidity of strawberry juice showed an overall increasing trend with an increase in fermentation duration, with a significant rise observed after 24 h. In contrast, the pH value of fermented strawberry juice exhibited a decreasing trend and showed a significant decline only during the 24–36 h fermentation period (Figure 1C,D). A similar trend was observed in a previous study by Randazzo et al. [22], who found that the total acidity of apples, grapes, pomegranates, and cacti juices fermented with water kefir grains increased. Esatbeyoglu et al. [23] and Bueno et al. [24], etc., reported a decrease in the pH value of aronia, red tequila, and other fruit juices fermented with water kefir grains, which is consistent with the trends observed in the present study. The production of organic acids during food fermentation leads to a reduction in pH value and thereby increases the total acidity [25]. Therefore, the increase in total acidity and decrease in pH value of fermented strawberry juice are related to the fermentation of sugars, alcohols, etc., in strawberry juice by LAB and AAB present in water kefir grains, resulting in acid production. The microbial fermentation of sugars in strawberry juice results in the accumulation of organic acids. Some microorganisms utilize endogenous organic acids, such as malic acid and citric acid in strawberry juice, as carbon sources, resulting in their consumption. Overall, the combined effect of acid production and its utilization determines the dynamic changes in total acidity and pH value of strawberry juice during fermentation.

### 3.3. Changes in Soluble Sugar Content During Fermentation

**Figure 2:**
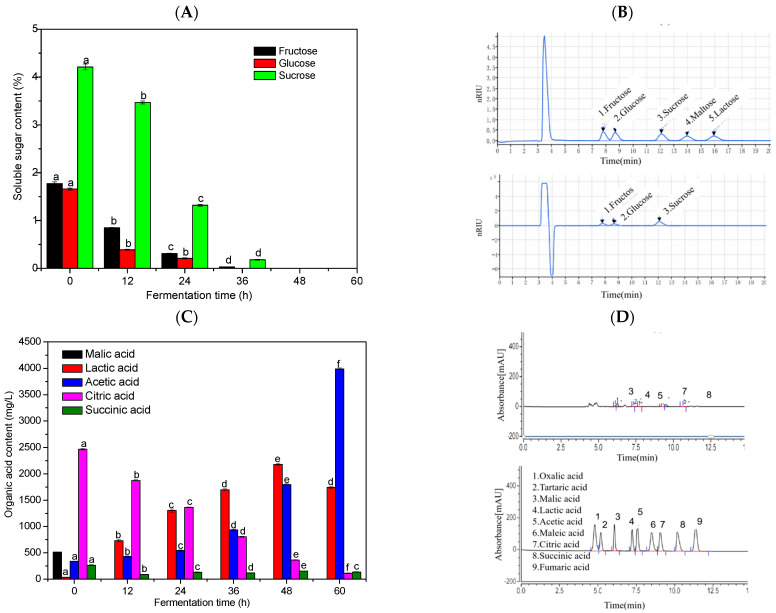
Changes in soluble sugar content (**A**), organic acid content (**C**), and their HPLC chromatograms ((**B**) soluble sugar, (**D**) organic acid) of strawberry juice fermented with water kefir grains during fermentation. (Note: Values within the same parameter followed by different lowercase letters are significantly different (*p* < 0.05)).

Based on the changes observed in SSC, pH, and total acidity in of fermented strawberry juice samples (Figure 1B–D), which are closely related to the metabolism of soluble sugars, the dynamic detection of soluble sugars at different times were investigated (Figure 2A). The contents of three soluble sugars such as sucrose, glucose, and fructose were detected in fermented strawberry juice samples and decreased rapidly as fermentation progressed. By 36 h of fermentation, fructose and glucose were almost undetectable, while sucrose content was only 0.18%. A similar trend was also observed in a previous study by Dikmetas et al. [26], who observed that the total sugar content was inversely proportional to fermentation time during the fermentation of apple, orange, pitaya, and kiwifruit juice with water kefir grains. During the fermentation process, the microorganisms in water kefir grains metabolize sugars to produce metabolic products such as organic acids, thereby further reducing the SSC and increasing the acidity of the samples.

### 3.4. Changes in Organic Acid Content During Fermentation

The accumulation of organic acids caused by the metabolism of sugars by microorganisms in fermented strawberry juice may be the main reason for the increase in total acidity and decrease in pH. Therefore, in this study, the main organic acids in strawberry juice fermented with water kefir grains were analyzed. A total of five organic acids were detected: malic, lactic, acetic, citric, and succinic acid (Figure 2C). The contents of citric and malic acid gradually decreased during fermentation. Among them, malic acid was detected to be 515.14 mg/L in the 0 h sample and was no longer detectable after 12 h of fermentation. The citric acid contents were detected to be 2465.71 mg/L at 0 h and 112.98 mg/L at 60 h of fermentation. In contrast, lactic and acetic acid exhibited an increasing trend. The lactic acid content increased significantly from 0 h to 48 h and decreased at 60 h. This may be attributed to the metabolism of lactic acid by certain microorganisms, including LAB (*Lab. Shb. Leb. Lqb.*, etc.), AAB (*Acetobacter* and *Gluconobacter*). The acetic acid content increased significantly throughout the fermentation period, especially during the middle and late stages of 36 h. This increase may be attributed to the substantial proliferation of AAB within the water kefir grains during the late fermentation stage. These microorganisms are capable of oxidizing the ethanol produced by yeast fermentation in the strawberry juice, leading to the accumulation of acetic acid. In contrast, succinic acid exhibited a fluctuating pattern of accumulation and depletion throughout fermentation, which likely reflects the dynamic metabolic interplay among the complex microbial community.

Similar results have been reported by Du et al. [27] and Bueno et al. [24], who detected that malic, lactic, acetic, citric, and other organic acids were detected in fermented longan and apple juices. The observed trends were consistent with those in the present study, with significant decreases in citric and malic acid and increases in lactic and acetic acid contents. The decrease in citric acid content after fermentation may be attributed to its conversion by LAB into metabolites such as diacetyl, lactic, and acetic acid [28]. In addition, lactic acid fermentation can reduce the malic acid content in fruit juice due to its involvement in the malolactic pathway and tricarboxylic acid (TCA) cycle [29]. The accumulation of large amounts of organic acid metabolites during fermentation not only affects the acidity of fermented strawberry juice but also has an important impact on the stability and metabolic activity of the overall fermentation system [30].

### 3.5. Analysis of Volatile Flavor Compounds

**Figure 3:**
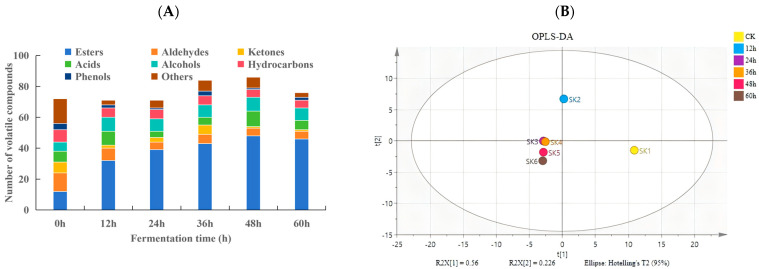

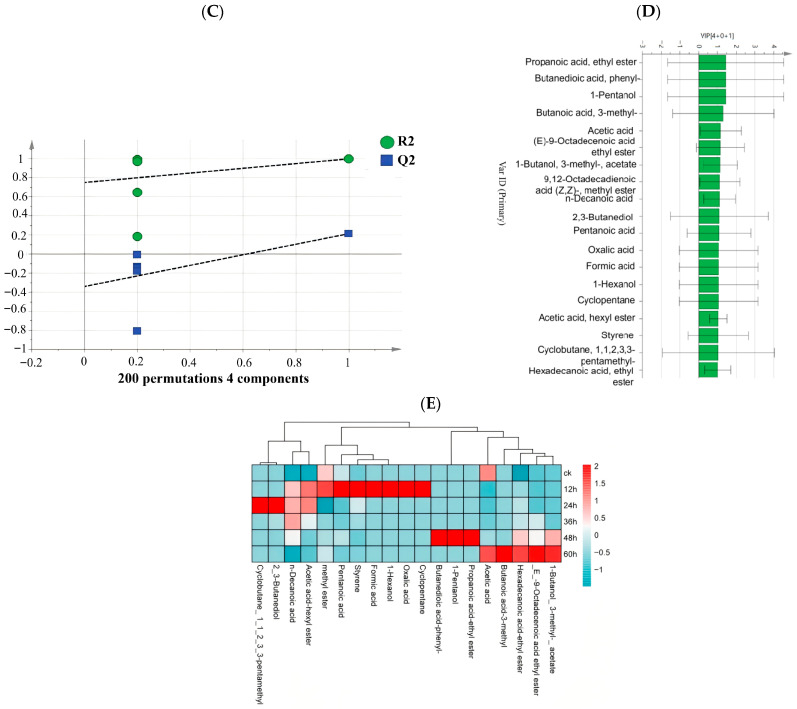
Changes in volatile compounds of strawberry juice fermented with water kefir grains during fermentation ((**A**) Types of volatile compounds; (**B**) OPLS-DA analysis of volatile compounds; (**C**) Cross-validation of 200 permutation tests; (**D**) Differential volatile compounds with VIP Value > 1; (**E**) Correlation heatmap of volatile compounds among each fermentation group).

A total of 218 volatile compounds were identified in the strawberry juice samples fermented with water kefir grains at different fermentation stages. These included 77 esters, 25 alcohols, 14 ketones, 18 aldehydes, 19 acids, 22 hydrocarbons, 6 phenols, and 37 other compounds (Figure 3A). The number of volatile compounds across all fermented samples exceeded 70. The highest number of volatile compounds was observed at 48 h, reaching 86 compounds, while the samples from 0 to 24 h contained fewer compounds but more than 70. The relative contents (>1%) of volatile aroma compounds of fermented strawberry juice (%) are shown in Supplementary Table S3.

Esters were the most abundant volatile compounds at each fermentation stage in strawberry juice samples, except at 0 h, accounting for 77 species (35.33%). The number of ester compounds increased significantly with the increase in fermentation time and reached a maximum of 48 species at 48 h. In contrast, the aldehydes were the most abundant in unfermented strawberry juice samples, with 12 species, and decreased remarkably with the increase in fermentation time. The number of other volatile compounds showed no significant change except for acids, which were significantly higher at 48 h than at other time periods.

Aldehydes and ketones were the most abundant in unfermented strawberry juice samples, contributing to a strong fresh fruit aroma. For example, 3,4-dimethylbenzaldehyde and benzaldehyde impart nutty and bitter almond flavors; while nonanal, trans-2-decenal, 2-octanone, and acetophenone contribute orange, citrus, apple, and hawthorn odors, respectively [31]. Esters were the main volatile components in fermented strawberry juice and the main contributor to the fruity flavor of strawberries and the characteristic flavor of fermented products [32]. The abundance of ester compounds was highest when the fermentation was extended to 48 h, resulting in a strong ester flavor. The compounds such as ethyl palmitate, ethyl laurate, ethyl decanoate, methyl (Z,Z)-9,12-octadecadienoate, ethyl linolenate, and other substances, impart oily, fruity, and wine-like aromas [33].

Using the relative contents of volatile flavor compounds as the response variables, supervised Orthogonal Partial Least Squares Discriminant Analysis (OPLS-DA) was applied to analyze the differences between strawberry juices fermented with water kefir at different time periods. The model fitting results were R^2^X = 0.955, R^2^Y = 0.800 and Q^2^ = −0.178, indicating a good fit for this set of data.

As shown in the score plot of OPLS-DA (Figure 3B), the SK1 group (CK group) was separated from the other groups along the X-axis. This indicated that there were significant differences in volatile substances between unfermented and fermented strawberry juice samples with water kefir grains at different times. The SK2 group (12 h) was separated from the other groups along the Y-axis, whereas the volatile flavor profiles of strawberry juice fermented for 24–60 h were very closely clustered. This indicated that the volatile substances in strawberry juice inclined to become similar after prolonged fermentation, and esters showed an obvious advantage. At the same time, the cross-validation result of 200 permutation tests (Figure 3C) showed that the intersection point of the regression curve of Q^2^ with the vertical axis was less than 0, indicating that the model was not over-fitted and was reliable and effective.

The VIP score was used to evaluate the contribution of each variable to the classification process in the OPLS-DA model. The statistical analysis VIP values of the discriminant metabolites for each sample of the strawberry juice with water kefir grains are shown in Supplementary Table S4.

Metabolites with VIP > 1 were considered important contributors to classification. Based on VIP values greater than 1, 19 volatile compounds that contributed significantly to flavor differences were screened (Figure 3D). Among them, 10 compounds including ethyl propionate, phenylsuccinic acid, 1-pentanol, isovaleric acid, acetic acid, ethyl elaidate, isoamyl acetate, methyl (Z,Z)-9,12-octadecadienoate, decanoic acid, and (1R,2S,5R)-endo-5-methyl-2-(1-methylethenyl)cyclohexanol, had VIP above 1.1. Whereas 9 compounds, including valeric acid, oxalic acid, formic acid, 1-hexanol, cyclopentane, hexyl acetate, styrene and ethyl palmitate, had VIP values between 1.0 and 1.1.

Esters such as ethyl propionate, ethyl elaidate, hexyl acetate, and methyl (Z,Z)-9,12-octadecadienoate reflected the differences in ester accumulation in strawberry juice samples at different fermentation times. Differences in alcohols indicated that fermentation weakened the fresh fruit flavor of strawberry juice. Whereas the differences in organic acids such as phenylsuccinic acid, decanoic acid, isovaleric acid, and acetic acid reflected the complexity of organic acid metabolism at different fermentation stages. On one hand, microorganisms ferment sugars to produce acids; on the other hand, these acids are used as important precursors for the formation of compounds such as alcohols, aldehydes, ketones, and esters [34]. Acids impart a pleasant flavor to fermented fruit juice at low concentrations but cause an unpleasant odor at higher concentrations [35].

Previous studies [36] have shown that the main aromatic compounds produced during the fermentation of sugar water with water kefir grains include 2-methyl-1-propanol, isoamyl alcohol, ethyl acetate, isoamyl acetate, and octyl acetate. In this study, only isoamyl acetate and 1-pentanol were detected. Isoamyl acetate imparts a strong, sweet, and banana-like aroma to the fermentation broth and is a key contributor to the volatile aroma characteristics [37]. This indicates that the synthesis and metabolism of esters during the fermentation of water kefir grain flora are significantly influenced by the strawberry juice matrix while retaining the characteristic aroma features.

The heat map (Figure 3E) showed significant differences in some volatile substances at different fermentation stages. Except for methyl (Z,Z)-9,12-octadecadienoate, other esters accumulated significantly post-fermentation with water kefir grains, and some volatile acids reached the highest value in the early fermentation stage (12 h). Among them, ethyl acetate showed the highest enrichment at 12 h and 24 h (relative content above 1.4%), ethyl palmitate continued to accumulate throughout fermentation, and ethyl elaidate and isoamyl acetate increased significantly only in the late stage of fermentation (after 48 h).

The changes of esters during fermentation may be attributed to the differences in the activity of ester-converting microorganisms in water kefir grains. Valeric acid, decanoic acid, oxalic acid, and formic acid were significantly higher at 12 h than at other fermentation stages. This may be due to the vigorous metabolism of acid-producing microorganisms in the early stage of fermentation. As fermentation progresses, these organic acids are then consumed by some microorganisms that use them as carbon sources to synthesize other metabolites, such as esters [38]. The accumulation of acetic acid increased significantly at 60 h; this may be due to the utilization of alcohols accumulated in the early stage by AAB in water kefir grains, resulting in the production of acetic acid in the late stage of fermentation.

### 3.6. Changes in Microbial Community Structure

#### 3.6.1. Analysis of Bacterial and Fungal Community Structure

**Figure 4:**
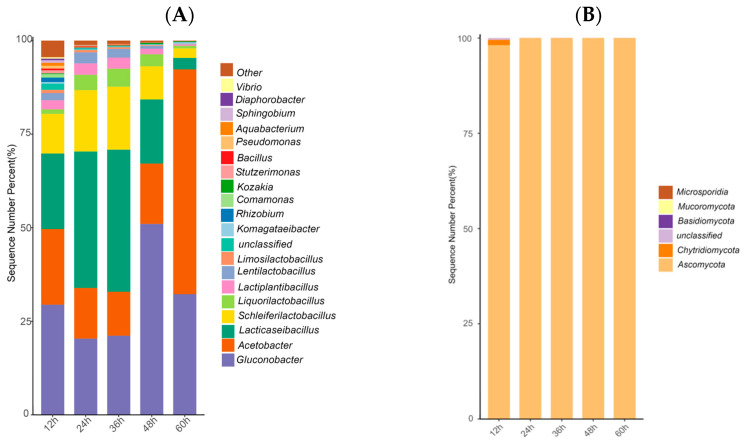

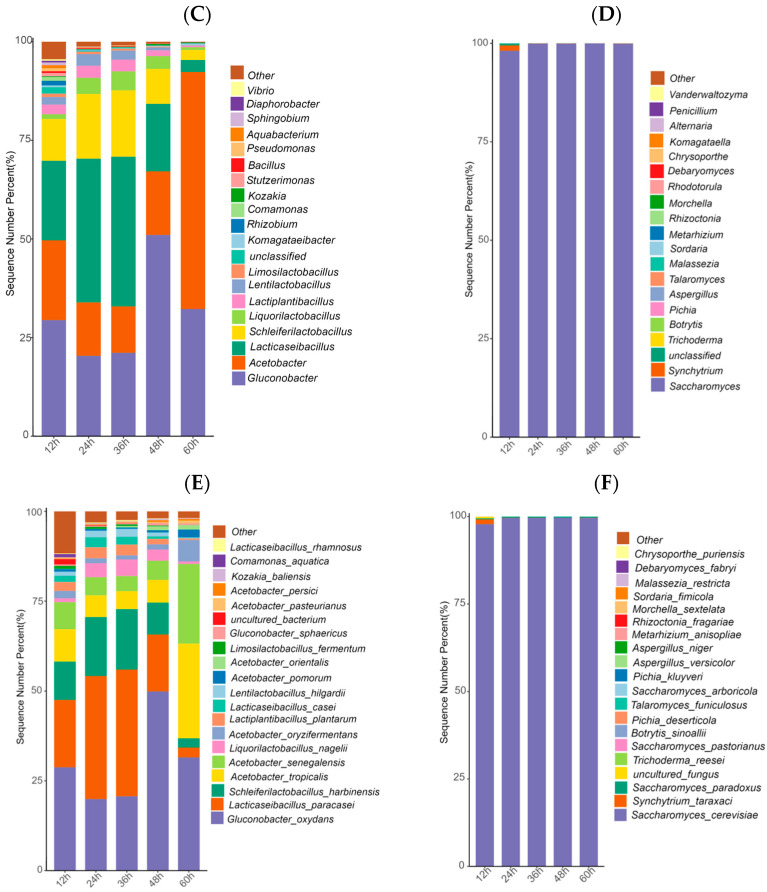
Changes in microbial flora of strawberry juice fermented with water kefir grains during fermentation ((**A**) Bacterial phyla; (**B**) Fungal phyla; (**C**) Bacterial genera; (**D**) Fungal genera; (**E**) Bacterial species; (**F**) Fungal species).

The main bacterial phyla observed in strawberry juice fermented with water kefir grains were *Pseudomonadota* (33.53–93.13%) and *Bacillota* (6.85–65.92%) (They were referred to as *Proteobacteria/Firmicutes* in early literature.), which accounted for more than 98% of the total bacterial community. Among them, *Pseudomonadota* was significantly higher than *Bacillota* at 12, 48, and 60 h of fermentation, and was the dominant bacterial phylum, accounting for 59.93%, 68.06%, and 93.13%, respectively. *Bacillota* was the dominant phylum at 24 and 36 h of fermentation, accounting for 64.25% and 65.92%, respectively (Figure 4A). These results differ from those reported by Wang et al. [28] and Fiorda et al. [4] that *Proteobacteria* and *Firmicutes* were the dominant phyla in water kefir grains from different sources at the phylum level. This difference may be related to the influence of the strawberry juice matrix on the bacterial community.

At each fermentation stage, the bacterial genera with relative abundance greater than 1% (Figure 4C) included *Gluconobacter* (20.36–51%), *Acetobacter* (11.76–60.09%), *Lacticaseibacillus* (*Lab*.) (3.03–37.93%), *Schleiferilactobacillus* (*Shb*.) (2.54–16.83%), *Liquorilactobacillus* (*Lqb*.) (0.7–4.8%), *Lactiplantibacillus* (*Lpb*.) (0.35–3.05%), *Lentilactobacillus* (*Llb*.) (0.12–2.96%), *Rhizobium* (0–1.18%), and unclassified (0–1.67%).

Mainly, at the species level, *Gluconobacter* was represented by *Gluconobacter oxydans*; *Acetobacter* by *Acetobacter tropicalis* and *Acetobacter senegalensis*; *Lab.* by *Lab. paracasei*; *Shb.* by *Shb. harbinensis*; *Lqb.* by *Lqb. nagelii*; *Lpb.* by *Lpb. plantarum*; and *Llb.* by *Llb. hilgardii* (Figure 4E).

Previous studies have shown that the bacterial genera in water kefir grains and their sugar water fermentation broth were mainly LAB, such as *Lqb.*, *Shb.*, and *Llb.* [4,11,39,40]. In this study, in addition to the above dominant LAB genera, *Gluconobacter* and *Acetobacter* showed relatively high abundance during the entire fermentation period. As the fermentation progressed, they also showed competitive alternating changes with the dominant LAB genera exhibiting a strong temporal replacement pattern. Their dominance became more pronounced in the late fermentation stage (48–60 h). Specifically, *Gluconobacter* and *Acetobacter* were dominant bacterial genera during 12 to 60 h fermentation, with relative abundances exceeding 10%. The relative abundance of *Acetobacter* was significantly affected by fermentation time, showing no significant change during 12 to 48 h, but increasing sharply to 60.09% at 60 h. This indicated that the late stage of fermentation was mainly dominated by acetic acid fermentation. *Lab.* and *Shb.* showed high relative abundance (above 10%) within 48 h of fermentation, but decreased significantly at 60 h, since AAB became the dominant genus in the samples at this stage. The change patterns of *Lpb.* and *Llb.* were similar to those of *Lab.* and *Shb.*, with relative abundances below 5%. This further indicated that LAB were dominant in strawberry juice fermented with water kefir grains during the first 48 h of fermentation.

*Rhizobium* (1.18%) and unclassified bacterial genera (1.67%) were also detected in the samples at the early stage of fermentation (12 h), indicating high bacterial diversity at this stage. This may be due to the difference in the fermentation matrix, and is also related to the utilization of the carbon source by LAB and AAB. LAB uses soluble sugars such as sucrose and glucose in the system as carbon sources to produce lactic acid in the early stage of fermentation, whereas AAB proliferate using secondary carbohydrate metabolites such as organic acids and ethanol, accumulated in the middle and late stages of fermentation as carbon sources [41]. This process is also influenced by the changes in dissolved oxygen in the fermentation broth during the middle and late stages of fermentation.

The main fungal phylum observed was *Ascomycota*, with relative abundance exceeding 97%, and the main fungal genus was *Saccharomyces*, with *Saccharomyces cerevisiae* as the dominant species. Its relative abundance was above 99.9% in fermented strawberry juice from 24 to 60 h (Figure 4B,D). *Synchytrium*, which accounted for 1.38%, was also detected in the 12 h sample. This indicated that yeast was absolutely dominant during the fungal fermentation of strawberry juice fermented with water kefir grains. This result is also consistent with previous studies reporting *S. cerevisiae* as the main fungus in the microbial floral analysis of water kefir grains [4].

#### 3.6.2. Analysis of Bacterial and Fungal Community Differences

**Figure 5:**
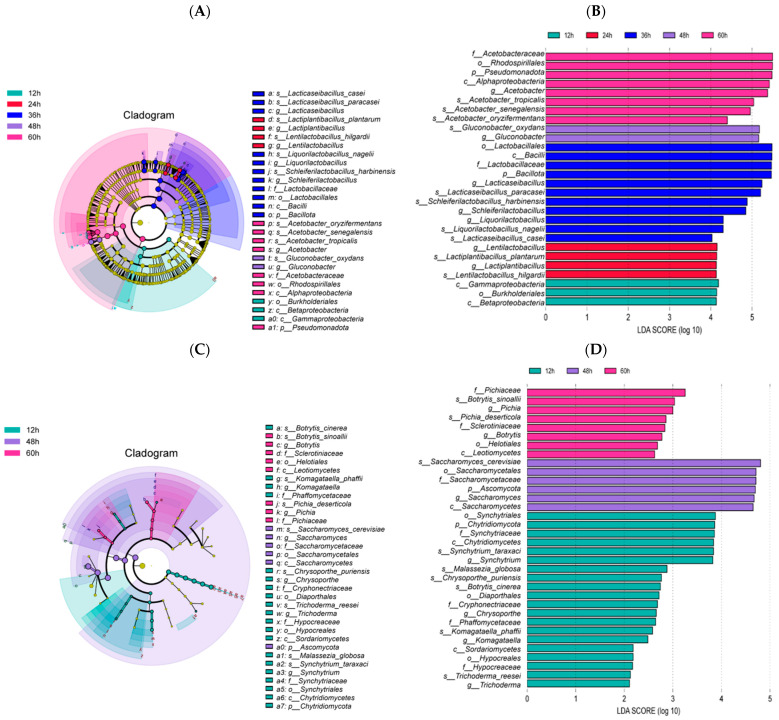
Changes in microbial flora differences of strawberry juice fermented with water kefir grains during fermentation ((**A**,**B**) LEfSe analysis of bacterial species; (**C**,**D**) LEfSe analysis of fungal species).

To further analyze the differences in the microbial communities at each fermentation time period in strawberry juice samples, Discriminant Analysis Effect size (LEfSe) was used (Figure 5A–D). The LEfSe cladogram revealed the taxonomic units with significant differences in the microbial community at different time points, whereas the LEfSe bar chart showed the differential microbial taxa at different fermentation times (linear discriminant analysis, LDA > 4 for bacteria, LDA > 2 for fungi).

For bacteria, a total of 28 representative differential bacterial taxa in strawberry juice samples at each fermentation stage were identified. These included 3 taxa at 12 h (*Gammaproteobacteria*, *Burkholderiales*, *Betaproteobacteria*); 4 at 24 h (*Llb.*, *Lpb.*, *Lpb.plantarum*); 11 at 36 h (*Lqb. nagelii*, *Lab.*, *Lmb.*, etc.); 2 at 48 h (*Gluconobacter*, *G. oxydans*); 8 at 60 h (*Rhodospirillales*, *Acetobacteraceae*, *A. senegalensis*, etc.).

For fungi, at the discrimination level of LDA > 2, no differential fungal species were detected in fermented strawberry juice samples at 24 and 36 h. A total of 34 representative differential fungal groups were identified in the strawberry juice at 12, 48, and 60 h. These included 20 taxa at 12 h (*Synchytriales*, *Chytridiomycota*, *Synchytriaceae*, etc.); 6 at 48 h (*S. cerevisiae*, *Saccharomycetales*, *Saccharomycetaceae*, etc.); 8 at 60 h (*Pichiaceae*, *Botrytis sinoallii*, *Pichia*, etc.).

The dominant LAB and other microbial flora in water kefir grains were not significantly enriched at 12 h, corresponding to the early fermentation stage. LAB was significantly dominant during 24 to 36 h of fermentation, which indicated that lactic acid fermentation entered the peak stage. *Gluconobacter* and other AAB were dominant at 48 h, suggesting the initiation of acetic acid fermentation. A large number of AAB were enriched at 60 h, with the fermentation process mainly being dominated by acetic acid fermentation and related metabolic processes.

Overall, the bacterial community demonstrated a significant change during the fermentation of strawberry juice with water kefir, whereas the fungal community was consistently dominated by *S. cerevisiae*. The microbial community structure shifted significantly with time, where it transitioned from an early fermentation stage dominated by LAB to a later stage dominated by AAB, *Bacillus*, and strains with strong environmental adaptability.

### 3.7. Correlation Analysis Between Microbial Community and Flavor Compounds

**Figure 6:**
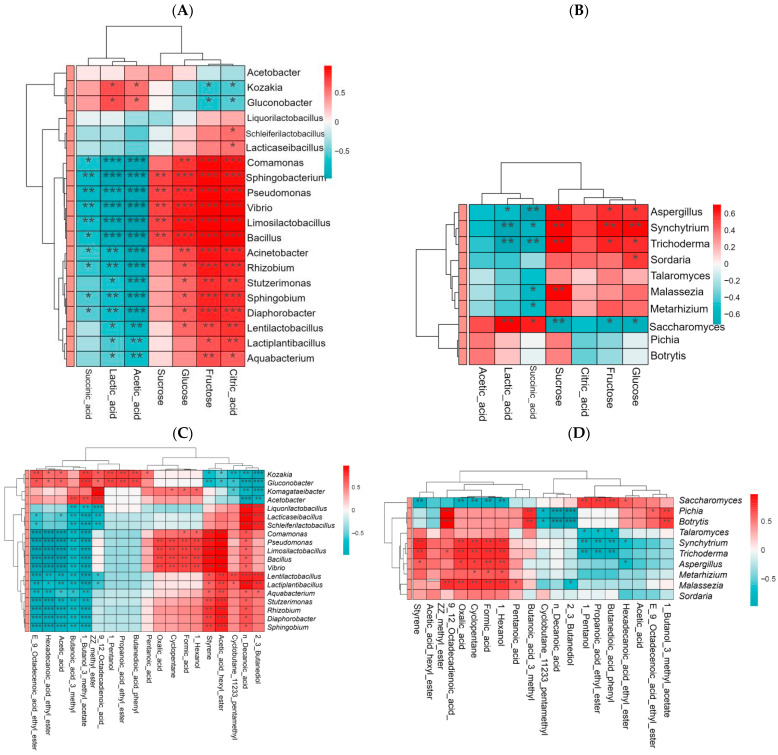
Correlation heatmap analysis of major microbial genera and flavor compounds in strawberry juice fermented with water kefir grains ((**A**) Bacterial genera vs. Organic acids and soluble sugars; (**B**) Fungal genera vs. Organic acids and soluble sugars; (**C**) Bacterial genera vs. Volatile compounds; (**D**) Fungal genera vs. Volatile compounds). (*: *p* < 0.05, **: *p* < 0.01, ***: *p* < 0.001).

Significant correlations were observed between multiple bacterial genera and specific organic acids and soluble sugars (Figure 6A). LAB genera such as *Lab.*, *Lmb.*, *Llb.*, and *Lpb.* were strongly positively correlated with lactic acid, indicating their involvement in the lactic acid fermentation in strawberry juice samples. Genera such as *Comamonas* and *Pseudomonas* were significantly correlated with TCA cycle intermediates, such as succinic acid and citric acid, and may be involved in carbon metabolism regulation. *Acetobacter* and *Gluconobacter* showed a strong positive correlation with acetic acid but a negative correlation with other organic acids. This indicated their important role in acetic acid fermentation, which is related to the strong metabolic capacity of acetic acid bacteria in environments rich in organic acids [42]. Most of the main bacterial genera showed a strong negative correlation with glucose, fructose, and sucrose, indicating that soluble sugars were gradually consumed during bacterial metabolism.

Similarly, significant correlations were also observed between the core dominant fungal genus (*Saccharomyces*) in fermented strawberry juice and main organic acids and soluble sugars (Figure 6B). *Saccharomyces* showed a significant negative correlation with fructose, glucose, and sucrose, and a positive correlation with succinic, lactic, and acetic acid. This indicated that yeast actively metabolized sugars to produce acids during fermentation. *Synchytrium* was significantly positively correlated with three soluble sugars. This may be due to the secretion of pectinase, glucoamylase, and other enzymes by *Synchytrium* during fermentation, further leading to the degradation of macromolecular substances such as pectin and cellulose to produce small molecular sugars in strawberry juice.

Figure 6C shows that the high-abundance AAB genera (*Gluconobacter*, *Acetobacter*) and LAB genera (*Lab.*, *Shb.*, *Lqb.*, *Lpb.*, *Llb.*) exhibited opposite correlation characteristics with key volatile compounds. For example, *Gluconobacter* and *Acetobacter* were significantly negatively correlated with 2,3-butanediol and decanoic acid, then significantly positively correlated with methyl (Z,Z)-9,12-octadecadienoate, isoamyl acetate, ethyl palmitate, etc., as some acetic acid producing bacterium (such as *Gluconobacter* and *Acetobacter*) can secrete ester synthase, which catalyzes the formation of ester bonds under specific conditions [11], whereas LAB such as *Lab.* and *Shb.* showed the opposite trend. Which might be attributed to their enzyme system expression.

Ethyl palmitate, methyl linoleate, and related compounds can contribute complex flavors, such as mellow and oily notes to fruit juice in the middle and late stages of fermentation, and their oxidation derivatives can impart secondary aromas including grassy and nutty notes [43]. Isoamyl acetate is a characteristic aroma compound with a strong fruity flavor. These results indicate that adjusting the abundance of *Gluconobacter* and *Acetobacter* during the fermentation may promote the accumulation of such characteristic esters and enhance the rich flavor of fermented strawberry juice. It should be noted that among the high-abundance LAB, *Lpb.* was significantly positively correlated with hexyl acetate. Hexyl acetate imparts a fresh and sweet pear, apple, green banana-like aroma with a hint of grassy and floral notes, which can enhance the fresh and juicy sensory characteristics of fermented strawberry juice, further complementing and balancing the strong sweet aroma of isoamyl acetate [44]. Together, these results suggest that high-abundance LAB may have a certain inhibitory effect on the accumulation of important characteristic flavor compounds during strawberry juice fermentation. Previous studies have reported that *Shb.,* which shows high relative abundance in water kefir, is correlated with specific important metabolites [45], which was also observed in this experiment (Figure 6C).

*Saccharomyces* was significantly positively correlated with ethyl palmitate, ethyl propionate, phenylsuccinic acid, and 1-pentanol, but significantly negatively correlated with 1-hexanol, cyclopentane, oxalic acid, and formic acid (Figure 6D). which is due to its high expression of long-chain acyl-CoA synthetase and fatty acid synthase (FAS) [43,46] during fermentation: FAS synthesizes endogenous long-chain fatty acids (e.g., palmitic acid, propionic acid) in yeast cells, and acyl-CoA synthetase activates these fatty acids to form acyl-CoA, which then condenses with ethanol (the main alcohol produced by yeast fermentation) to generate long-chain fatty acid ethyl esters; meanwhile, yeast can secrete pectinase and glucosidase to decompose macromolecular substances in strawberry juice into small molecular sugars, providing carbon sources for its own metabolism and the secondary metabolism of AAB, forming a microbial synergy for ester synthesis.

Ethyl palmitate has mild, waxy, fruity, and creamy aromas, which can enhance the fullness and mellow sense of the product, and it is generally accumulated in the late stage of fermentation. Long-chain fatty acid ethyl esters are often regarded as important auxiliary aroma components that contribute to the flavor complexity [42]. Ethyl propionate exhibits distinct fruity, sweet, and ether-like aromas, is a key aroma-active ester in many fermented fruit juices and can enhance the fresh fruit flavor of the product [46]. As an acidic compound, phenylsuccinic acid can slightly influence the pH value and acidic flavor, but its more important role may be as a flavor precursor or modifier. 1-Pentanol has a herbal, nutty, and slightly fermented odor, but may become irritating at high concentrations [33]. *Pichia* and *Botrytis* showed extremely significant positive correlations with methyl (Z,Z)-9,12-octadecadienoate, and suggested that low-abundance microorganisms also play an important role in the metabolism of key flavor compounds during the fermentation process.

### 3.8. Prediction of Main Flavor Compound Metabolic Pathways

Flavor is one of the important qualities of kefir products. Kefir is mainly formed by the co-fermentation of complex microbial communities, including lactic acid bacteria, yeast, and a few other bacteria. The biodiversity of microbial communities provides kefir with rich flavors. The correlation analysis of present study indicated that, as fermentation progresses, acid-producing bacteria such as LAB consume reducing sugars to produce acetic acid, and lactic acid, etc.; *Comamonas* and *Pseudomonas* were significantly correlated with TCA cycle intermediates, such as succinic acid and citric acid; *Acetobacter* and *Gluconobacter* showed strong positive correlations with acetic acid. *Saccharomyces* showed a positive correlation with succinic, lactic, and acetic acid. It is reported that microbial enzymes such as yeast use acetic acid, lactic acid, and alcohol as substrates to synthesize esters such as ethyl caproate, ethyl acetate, and ethyl lactate [11]. In our present investigation, *Saccharomyces* was significantly positively correlated with ethyl palmitate, ethyl propionate, phenylsuccinic acid, and 1-pentanol. Additionally, the high-abundance LAB, *Lpb.* was significantly positively correlated with hexyl acetate. These indicate that LAB, yeast, and *Gluconobacter*, etc., in the strawberry juice jointly regulate the fermentation of the process.

**Figure 7:**
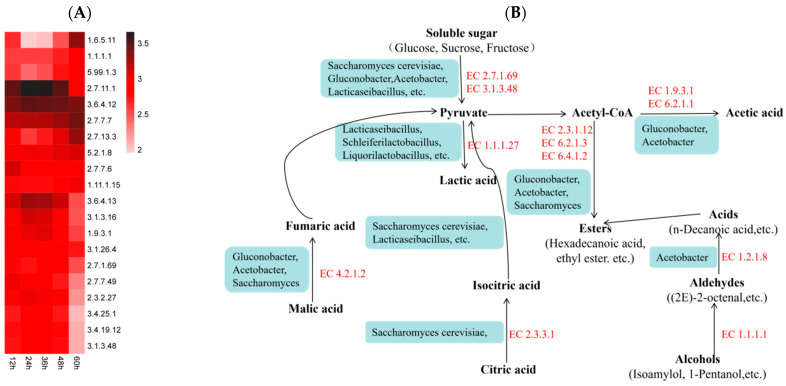
Prediction of metabolic pathways of major flavor compounds in strawberry juice fermented with water kefir grains ((**A**) Heatmap of relevant enzymes during fermentation (Top 20); (**B**) Prediction of metabolic pathways of major compounds).

To elucidate the underlying mechanisms of flavor formation, a correlation analysis was conducted among the dominant microbial genera, soluble sugars, organic acids, and key volatile substances throughout the fermentation process. This analysis, combined with the expression profiles of high-abundance enzymes, enabled the prediction of pivotal metabolic pathways involved in the generation of key flavor compounds (Figure 7, partially listed; Figure 7B).

Combined with the changes in the soluble sugars (Figure 2A), sucrose, glucose, and fructose all decreased rapidly in the early stage of fermentation (0–24 h). At this stage, sugars may be catalyzed by EC 2.7.1.69 and EC 3.1.3.48, produced by yeasts, AAB, and LAB, to enter glycolysis or the pentose phosphate pathway through phosphorylation and are converted into pyruvate. Pyruvate is converted into lactic acid through one pathway, catalyzed by EC 1.1.1.27 of LAB such as *Lab.*, and into acetyl-CoA through decarboxylation reaction, then into acetic acid by *Gluconobacter* and *Acetobacter,* catalyzed by EC 1.9.3.1 and EC 6.2.1.1.

EC 2.3.3.1, highly expressed by *Saccharomyces*, can catalyze the conversion of citric acid into isocitric acid, which is then converted into pyruvate through the reverse (TCA) cycle or anaplerotic pathway. Malic acid is converted into fumaric acid through the (TCA) cycle, catalyzed by EC 4.2.1.2 produced by *Gluconobacter,* and then into pyruvate.

Acetyl-CoA formed by pyruvate decarboxylation is converted into esters through catalysis by EC 2.3.1.12, EC 6.2.1.3, etc. Alcohols such as isoamyl alcohol and 1-pentanol are converted at the same time are converted into aldehydes through EC 1.1.1.1 (alcohol dehydrogenase), to acids (such as n-decanoic acid) by EC 1.2.1.8, and further into esters.

This predicted pathway provides a visual metabolic network for understanding the conversion mechanism of soluble sugars and main organic acids in strawberry juice fermented with water kefir grains, and further regulating fermentation flavor characteristics, such as acidity and volatile odor.

## 4. Conclusions

Through dynamic analysis of main physicochemical properties, flavor compounds, and microbial flora in strawberry juice fermented with water kefir during 0–60 h fermentation, it was found that fermentation time had a significant impact on important indexes of strawberry juice. Among them, total acidity gradually increased, while pH value and SSC decreased significantly in the early stage of fermentation; the contents of malic acid and citric acid detected during fermentation gradually decreased, acetic acid content increased in a gradient, lactic acid increased significantly in the first 48 h, while succinic acid alternately increased and decreased, and three soluble sugars (fructose, glucose, and sucrose) all gradually decreased.

A total of 218 volatile substances were identified, including esters, alcohols, ketones, aldehydes, acids, and other compounds in strawberry juice at each stage of fermentation. The microbial community in the fermented strawberry juice was dominated by AAB (*Gluconobacter* and *Acetobacter*), LAB (*Lab*., *Shb.* and *Lpb.*) and yeast (*Saccharomyces cerevisiae*), and their temporal succession was identified as the key factor regulating flavor formation. Specifically, *Saccharomyces cerevisiae* and LAB in the early and middle fermentation stages provided small-molecular precursors for subsequent fermentation processes through glycolysis, lactic acid fermentation and extracellular enzyme secretion. In the late stage, AAB promoted ester synthesis by expressing acyl-CoA synthetase and alcohol acyltransferase, and moderately oxidized ethanol to optimize the substrate microenvironment for ester biosynthesis. The main flavor metabolic pathways were centered on glycolysis and the TCA cycle, with key enzymes including alcohol dehydrogenase, acyl-CoA synthetase and alcohol acyltransferase derived from yeast, AAB and LAB serving as the catalytic core. These enzymes enabled the transformation of soluble sugars and endogenous organic acids into microbial-derived organic acids and characteristic esters. This study clarified the key microbial consortia and their enzyme-catalyzed mechanisms governing flavor formation in strawberry juice fermented with water kefir grains and provided a theoretical basis for the directional regulation of flavor quality in fermented fruit and vegetable juices. Additionally, in this study, strawberries were selected as the fermentation raw material, aiming to expand the market potential of kefir drinks. This approach not only meets consumers’ diverse demands for beverage flavors but also promotes the deep processing of strawberry products, thereby supporting agricultural development.

## Data Availability

The original contributions presented in this study are included in the article/Supplementary Materials. Further inquiries can be directed to the corresponding author.

## Supplementary Materials

The following supporting information can be downloaded at: https://www.mdpi.com/article/10.3390/foods15081312/s1, Table S1: Data output quality including the total reads and read counts before and after filtering for each sample of the community compositions analysis; Table S2: List of analyzed Taxonomy, along with their OTU, and GenBank accession number of the community compositions analysis of fermented strawberry juice samples; Table S3: The volatile aroma compounds detected and measured in the samples collected from strawberry juice fermented with water kefir grains; Table S4: The statistical analysis VIP values of the discriminant metabolites for each sample of the strawberry juice with water kefir grains.

## References

[1] Pariya D., Valérie O., José L.M. (2021). Process optimization for development of a novel water kefir drink with high antioxidant activity and potential probiotic properties from russian olive fruit (*Elaeagnus angustifolia*). Food Bioprocess Technol..

[2] Calatayud M., Aragao R., Ghyselinck J., Verstrepen L., De Medts J., Van den Abbeele P., Boulangé C.L., Priour S., Marzorati M., Damak S. (2021). Water kefir and derived pasteurized beverages modulate gut microbiota, intestinal permeability and cytokine production in vitro. Nutrients.

[3] Egea M.B., Santos D.C.D., Oliveira Filho J.G., Ores J.D.C., Takeuchi K.P., Lemes A.C. (2020). A review of non-dairy kefir products: Their characteristics and potential human health benefits. Crit. Rev. Food Sci..

[4] Fiorda F.A., Pereira G.V.D.M., Thomaz-Soccol V., Rakshit S.K., Pagnoncelli M.G.B., Vandenberghe L.P.D.S. (2017). Microbiological, biochemical, and functional aspects of sugary kefir fermentation—A review. Food Microbiol..

[5] Yin L.L., Chen Z.N., Sun R.X., Liu X.Y., Wang Y.X. (2025). Analysis of the quality stability of strawberry juice fermented with water kefir grains during refrigeration. Sci. Technol. Food Ind..

[6] Alves V., Scapini T., Frumi C.A., Bonatto C., Spitza Stefanski F., Pompeu de Jesus E., Techi Diniz L.G., Canhadas Bertan L., Resende Maldonado R., Treichel H. (2021). Development of fermented beverage with water kefir in water soluble coconut extract (*Cocos nucifera* L.) with inulin addition. LWT—Food Sci. Technol..

[7] Gökırmaklı Ç., Gün İ., Kartal M.O., Güzel Seydim Z.B. (2025). Antioxidant capacity, volatile compounds, microbial, chemical and sensory properties of plum (*Prunus domestica*) juice water kefir. Discover Food.

[8] Spizzirri U.G., Loizzo M., Aiello F., Prencipe S.A., Restuccia D. (2023). Non-dairy kefir beverages: Formulation, composition, and main features. J. Food Compos. Anal..

[9] Jorge L.P., María L.E.G., Isabel M.V. (2022). A new functional kefir fermented beverage obtained from fruit and vegetable juice: Development and characterization. LWT—Food Sci. Technol..

[10] Martínez-Torres A., Gutiérrez-Ambrocio S., Heredia-Del-Orbe P., Villa-Tanaca L., Hernandez-Rodriguez C. (2017). Inferring the role of microorganisms in water kefir fermentations. Int. J. Food Sci. Technol..

[11] Wang Y., Wang X.D., Xu Y.H., Li Y.L., Liu Y.G., Lin X.N. (2025). Volatile flavor characteristics and microbial fermentation differences of water kefir grains from different origins. Eur. Food Res. Technol..

[12] Tan Y., Zhong H., Zhao D., Du H., Xu Y. (2019). Succession rate of microbial community causes flavor difference in strong-aroma Baijiu making process. Int. J. Food Microbiol..

[13] Chen X.W., Cheng Y.J., Jiang L.X., Cui Y.L., Mao J.W., Sha R.Y. (2020). Studies on the changes of metabolites and antioxidant activity during the fermentation process of strawberry jiaosu. J. Chin. Inst. Food Sci. Technol..

[14] Da Cintia S.A., Macedo L.L., Luciano J.Q.T. (2023). Use of mid-infrared spectroscopy to predict the content of bioactive compounds of a new non-dairy beverage fermented with water kefir. LWT—Food Sci. Technol..

[15] (2021). National Food Safety Standard—Determination of Total Acid in Foods.

[16] Vatsala S., Shanti P., Emmandi R., Ajay K.A., Veena B., Vivekanand K., Deepak S. (2017). Detailed characterization of bio-oil from pyrolysis of non-edible seed-cakes by Fourier Transform Infrared Spectroscopy (FTIR) and gas chromatography mass spectrometry (GC-MS) techniques. J. Chromatogr. B.

[17] (2016). National Food Safety Standard—Determination of Organic Acid in Foods.

[18] (2023). National Food Safety Standard—Determination of Fructose, Glucose, Sucrose, Maltose and Lactose in Foods.

[19] Yaqub G., Hamid A., Khan N., Ashfaq S., Banzir A., Javed T. (2020). Biomonitoring of workers exposed to volatile organic compounds associated with different occupations by headspace GC-FID. J. Chem..

[20] Bolger A.M., Marc L., Bjoern U. (2014). Trimmomatic: A flexible trimmer for Illumina sequence data. Bioinformatics.

[21] Langmead B., Salzberg S.L. (2012). Fast gapped-read alignment with bowtie 2. Nat. Methods.

[22] Randazzo W., Corona O., Guarcello R., Francesca N., Germanà M.A., Erten H., Moschetti G., Settanni L. (2016). Development of new non-dairy beverages from mediterranean fruit juices fermented with water kefir microorganisms. Food Microbiol..

[23] Esatbeyoglu T., Fischer A., Legler A.D.S., Oner M.E., Wolken H.F., Köpsel M., Ozogul Y., Özyurt G., De Biase D., Ozogul F. (2023). Physical, chemical, and sensory properties of water kefir produced from Aronia melanocarpa juice and pomace. Food Chem. X.

[24] Bueno R.S., Ressutte J.B., Hata N.N.Y., Henrique-Bana F.C., Guergoletto K.B., De Oliveira A.G., Spinosa W.A. (2021). Quality and shelf life assessment of a new beverage produced from water kefir grains and red pitaya. LWT—Food Sci. Technol..

[25] Puerari C., Magalhaes-Guedes K.T., Schwan R.F. (2015). Physicochemical and microbiological characterization of chicha, a rice-based fermented beverage produced by Umutina Brazilian Amerindians. Food Microbiol..

[26] Dikmetas D.N., Acar E.G., Ceylan F.D., Lkadm F., Zer H., Karbancioglu-Guler F. (2025). Functional fermented fruit juice production and characterization by using water kefir grains. J. Food Sci. Technol..

[27] Du G., Qing Y., Wang H., Wang N., Yue T., Yuan Y. (2023). Effects of Tibetan kefir grain fermentation on the physicochemical properties, phenolics, enzyme activity, and antioxidant activity of *Lycium barbarum* (*Goji berry*) juice. Food Biosci..

[28] Wang Z., Feng Y., Yang N., Jiang T., Xu H., Lei H. (2022). Fermentation of kiwifruit juice from two cultivars by probiotic bacteria: Bioactive phenolics, antioxidant activities and flavor volatiles. Food Chem..

[29] Li S., Tao Y., Li D., Wen G., Zhou J., Manickam S., Han Y., Chai W.S. (2021). Fermentation of blueberry juices using autochthonous lactic acid bacteria isolated from fruit environment: Fermentation characteristics and evolution of phenolic profiles. Chemosphere.

[30] Laureys D., Frédéric L., Hauffman T., Raes M., Aerts M., Vandamme P. (2021). The type and concentration of inoculum and substrate as well as the presence of oxygen impact the water kefir fermentation process. Front. Microbiol..

[31] Shu W.X., Wu Z.F., Liu L.L., Weng P.F. (2018). Volatile flavor compounds of fermented grapefruit juice with probiotics. Food Sci..

[32] Aihaiti A., Zhao L., Maimaitiyiming R., Wang L., Liu R., Mu Y. (2025). Changes in volatile flavors during the fermentation of tomato (*Solanum lycopersicum* L.) juice and its storage stabilization. Food Chem..

[33] Lan T., Lv X., Zhao Q., Lei Y., Gao C., Yuan Q. (2023). Optimization of strains for fermentation of kiwifruit juice and effects of mono-and mixed culture fermentation on its sensory and aroma profiles. Food Chem. X.

[34] Jiao Y.W., Dou Z.T., Tian S., Liu M., Fang H.T. (2025). HS-SPME-GC-MS-based profiling of volatile metabolite variations during potato thick juice fermentation. Sci. Technol. Food Ind..

[35] Zhang X.Q., Su H., Li D., An Y.L., Jiang S.X., Zhang F. (2024). Comparative analysis of the quality of rose hip fruit wine fermented with different commercial *Saccharomyces cerevisiae*. China Brew..

[36] Laureys D., De Vuyst L. (2017). The water kefir grain inoculum determines the characteristics of the resulting water kefir fermentation process. J. Appl. Microbiol..

[37] Chen T., Wang H., Su W., Mu Y., Tian Y. (2023). Analysis of the formation mechanism of volatile and non-volatile flavor substances in corn wine fermentation based on high-throughput sequencing and metabolomics. Food Res. Int..

[38] Ma Y., Geng W.T., Wu J.J., Wang Y.P. (2019). Lactic acid bacteria metabolism and the formation of food flavor substances. China Condiment.

[39] Lynch K.M., Wilkinson S., Daenen L., Arendt E.K. (2021). An update on water kefir: Microbiology, composition and production. Int. J. Food Microbiol..

[40] Patel S.H., Tan J.P., Brner R., Zhang S., Priour S., Lima A. (2022). A temporal view of the water kefir microbiota and flavor attributes. Innov. Food Sci. Emerg..

[41] Laureys D., Luc D.V. (2014). Microbial species diversity, community dynamics, and metabolite kinetics of water kefir fermentation. Appl. Environ. Microbiol..

[42] Philippe C., Krupovic M., Jaomanjaka F., Claisse O., Petrel M., Le Marrec C. (2018). Bacteriophage GC1, a novel Tectivirus infecting *Gluconobacter cerinus*, an acetic acid bacterium associated with wine-making. Viruses.

[43] Molina A.M., Guadalupe V., Varela C., Swiegers J.H., Pretorius I.S., Agosin E. (2009). Differential synthesis of fermentative aroma compounds of two related commercial wine yeast strains. Food Chem..

[44] Lilly M., Bauer F.F., Styger G., Lambrechts M.G., Pretorius I.S. (2006). The effect of increased branched-chain amino acid transaminase activity in yeast on the production of higher alcohols and on the flavour profiles of wine and distillates. FEMS Yeast Res..

[45] Ma D., Wang B., Suo H., Wang J.H., Bai W.B. (2025). Analysis of microbial community structure and metabolite dynamics during the fermentation process of water kefir. Food Ferment. Ind..

[46] Pino J.A., Queris O. (2010). Analysis of volatile compounds of pineapple wine using solid-phase microextraction techniques. Food Chem..

