# Supplementary material for: Formation Mechanism of Key Flavor Compounds During the Fermentation of Strawberry Juice with Water Kefir Grains
Source: Foods. 2026 Apr 10;15(8):1312. doi: 10.3390/foods15081312 (PMC13115247; doi:10.3390/foods15081312)
Supplement: Supplementary file 1 [file foods-15-01312-s001.zip › foods-4204812-supplementary.pdf]

Supplementary materials

Supplementary Table S1 Data output quality including the total reads and read counts before and after filtering for each sample of the community compositions analysis

Sample ID	InsertSize (bp)	SeqStrategy	RawReads(#)	Raw Base (GB)	%GC	Raw Q20(%)	Raw Q30 (%)	Clean Reads (#)	Cleaned (%)	Clean Q20 (%)	Clean Q30 (%)
KS12h1	350	(150:150)	23798671	7.14	44	99.55	97.72	23098541	97.06	99.82	98.47
KS12h2	350	(150:150)	23967380	7.19	48	99.42	97.43	23168159	96.67	99.81	98.4
KS12h3	350	(150:150)	23049884	6.91	43	99.59	97.75	22285255	96.68	99.83	98.44
KS24h1	350	(150:150)	23148039	6.94	41	99.63	98.04	22468026	97.06	99.85	98.68
KS24h2	350	(150:150)	22425494	6.73	40	99.61	97.93	21800049	97.21	99.84	98.56
KS24h3	350	(150:150)	24104109	7.23	40	99.63	98.05	23424865	97.18	99.85	98.68
KS36h1	350	(150:150)	21781421	6.53	42	99.62	97.93	21164328	97.17	99.84	98.56
KS36h2	350	(150:150)	20737382	6.22	40	99.43	97.3	20260265	97.7	99.76	98.1
KS36h3	350	(150:150)	22744385	6.82	41	99.66	98.14	22138130	97.33	99.86	98.73
KS48h1	350	(150:150)	23670876	7.1	45	99.44	97.32	23124600	97.69	99.77	98.14
KS48h2	350	(150:150)	20471362	6.14	45	99.46	97.34	19985730	97.63	99.77	98.13
KS48h3	350	(150:150)	25577178	7.67	47	99.64	97.93	24817959	97.03	99.85	98.58
KS60h1	350	(150:150)	25005509	7.5	54	99.43	97.2	24375961	97.48	99.77	98.1
KS60h2	350	(150:150)	24429405	7.33	53	99.46	97.36	23859283	97.67	99.79	98.23
KS60h3	350	(150:150)	29240747	8.77	54	99.48	97.35	28537112	97.59	99.78	98.17

Supplementary Table S2 List of analyzed Taxonomy, along with their OTU, and GenBank accession number of the community compositions analysis of fermentated strawberry juice samples

#OTU ID	KS 24h 2	KS 24h 3	KS 24h 1	KS 12h 3	KS 12h 1	KS 12h 2	KS 36h 1	KS 36h 2	KS 36h 3	KS 48h 1	KS 48h 2	KS 48h 3	KS 60h 2	KS 60h 1	KS 60h 3	Taxonomy
NCBI100468	54	71	105	208	156	126	80	69	76	90	54	155	97	96	44	k__Bacteria;p__Bacillota;c__Bacilli;o__Lactobacillales;f__Lactobacillaceae;g__Schleiferilactobacillus;s__Schleiferilactobacillus_perolens
NCBI1006576	0	0	0	0	0	0	0	0	0	0	0	1	0	0	0	k__Bacteria;p__Thermotogota;c__Thermotogae;o__Petrotogales;f__Petrotogaceae;g__Defluviitoga;s__Defluviitoga_tunisiensis
NCBI1010	0	0	0	0	0	1	0	0	0	0	0	0	0	0	0	k__Bacteria;p__Bacteroidota;c__Sphingobacteriia;o__Sphingobacteriales;f__Sphingobacteriaceae;g__Sphingobacterium;s__Sphingobacterium_mizutaii
NCBI101028	0	0	0	0	0	1	0	0	0	1	0	0	0	0	0	k__Fungi;p__Ascomycota;c__Sordariomycetes;o__Hypocreales;f__Nectriaceae;g__Fusarium;s__Fusarium_pseudograminearum
NCBI101192	1	0	0	0	0	0	0	0	0	0	0	0	0	0	0	k__Archaea;p__Euryarchaeota;c__Methanomicrobia;o__Methanosarcinales;f__Methanosarcinaceae;g__Methanomethylovorans;s__Methanomethylovorans_hollandica
NCBI101201	0	0	0	0	0	0	0	0	0	0	1	0	0	0	0	k__Fungi;p__Ascomycota;c__Sordariomycetes;o__Hypocreales;f__Hypocreaceae;g__Trichoderma;s__Trichoderma_asperellum
NCBI101564	20	27	19	0	48	552	24	3	19	0	0	7	0	6	0	k__Bacteria;p__Pseudomonadota;c__Gammaproteobacteria;o__Pseudomonadales;f__Pseudomonadaceae;g__Pseudomonas;s__Pseudomonas_alcaliphila
NCBI1016987	45	40	20	2	67	1380	46	0	19	8	0	11	6	0	7	k__Bacteria;p__Pseudomonadota;c__Alphaproteobacteria;o__Sphingomonadales;f__Sphingomonadaceae;g__Novosphingobium;s__Novosphingobium_sp._THN1
NCBI1	0	1	1	0	0	0	0	3	0	0	0	0	0	0	0	k__Bacteria;p__Bacteroidota;c__Flavobacteriia;o__Flavo

017																bacteriales;f__Flavobacteriaceae;g__Capnocytophaga;s__Capnocytophaga_gingivalis
NCBI1019	0	0	0	2	0	0	0	2	0	0	0	0	0	0	0	k__Bacteria;p__Bacteroidota;c__Flavobacteriia;o__Flavobacteriales;f__Flavobacteriaceae;g__Capnocytophaga;s__Capnocytophaga_sputigena
NCBI10258	0	0	0	0	0	0	0	1	0	0	0	0	0	0	0	k__Bamfordvirae;p__Nucleocytoviricota;c__Pokkesviricetes;o__Chitovirales;f__Poxviridae;g__Parapoxvirus;s__Orf_virus
NCBI102684	2	0	0	0	6	19	0	0	0	0	0	0	0	0	0	k__Bacteria;p__Bacillota;c__Bacilli;o__Lactobacillales;f__Streptococcaceae;g__Streptococcus;s__Streptococcus_infantarius
NCBI1028752	0	0	0	0	0	0	0	0	0	0	0	2	0	0	0	k__Bacteria;p__Pseudomonadota;c__Gammaproteobacteria;o__Alteromonadales;f__Shewanellaceae;g__Shewanella;s__Shewanella_aestuarii
NCBI1028989	0	0	0	0	0	1	1	0	0	0	0	0	0	0	0	k__Bacteria;p__Pseudomonadota;c__Gammaproteobacteria;o__Pseudomonadales;f__Pseudomonadaceae;g__Pseudomonas;s__Pseudomonas_sp._StFLB209
NCBI1030157	1	3	0	0	5	79	3	0	0	0	0	0	0	0	0	k__Bacteria;p__Pseudomonadota;c__Alphaproteobacteria;o__Sphingomonadales;f__Sphingomonadaceae;g__Sphingomonas;s__Sphingomonas_sp._KC8
NCBI1032851	0	0	0	0	0	0	0	0	0	0	0	1	0	0	0	k__Bacteria;p__Actinomycetota;c__Actinomycetes;o__Mycobacteriales;f__Corynebacteriaceae;g__Corynebacterium;s__Corynebacterium_nuruki
NCBI1033846	0	0	0	0	0	0	0	0	0	0	0	3	0	0	0	k__Bacteria;p__Pseudomonadota;c__Gammaproteobacteria;o__Pseudomonadales;f__Marinobacteraceae;g__Marinobacter;s__Marinobacter_adhaerens
NCBI1034889	0	1	0	0	8	9	0	0	1	0	2	2	0	0	1	k__Bacteria;p__Pseudomonadota;c__Betaproteobacteria;o__Burkholderiales;f__Comamonadaceae;g__Variovorax;s__Variovorax_sp._HW608

NCBI103731	0	0	0	0	0	5	0	0	0	0	0	0	0	0	0	k__Bacteria;p__Actinomycetota;c__Actinomycetes;o__Pseudonocardiales;f__Pseudonocardiaceae;g__Saccharothrix;s__Saccharothrix_espanaensis
NCBI103733	0	0	0	0	0	5	0	0	0	0	0	0	0	0	0	k__Bacteria;p__Actinomycetota;c__Actinomycetes;o__Pseudonocardiales;f__Pseudonocardiaceae;g__Saccharothrix;s__Saccharothrix_syringae
NCBI103816	0	0	0	0	0	10	0	0	0	0	0	0	0	0	0	k__Bacteria;p__Actinomycetota;c__Actinomycetes;o__Mycobacteriales;f__Nocardiaceae;g__Rhodococcus;s__Rhodococcus_pyridinivorans
NCBI103855	11	5	0	0	11	45	5	10	17	5	10	20	379	448	435	k__Bacteria;p__Pseudomonadota;c__Betaproteobacteria;o__Burkholderiales;f__Alcaligenaceae;g__Bordetella;s__Bordetella_hinzii
NCBI10407	0	2	0	0	0	0	0	0	0	0	0	0	0	0	0	k__Pararnavirae;p__Artverviricota;c__Revtraviricetes;o__Blubervirales;f__Hepadnaviridae;g__Orthohepadnavirus;s__Hepatitis_B_virus
NCBI1040979	0	0	0	0	0	1	0	0	0	0	0	0	0	0	0	k__Bacteria;p__Pseudomonadota;c__Betaproteobacteria;o__Burkholderiales;f__Burkholderiaceae;g__Cupriavidus;s__Cupriavidus_neocaledonicus
NCBI104100	0	0	9	30	22	45	19	8	0	8	0	17	0	9	36	k__Bacteria;p__Pseudomonadota;c__Alphaproteobacteria;o__Rhodospirillales;f__Acetobacteraceae;g__Acetobacter;s__Acetobacter_lovaniensis
NCBI104102	168218	172706	179284	573016	448237	347981	222735	141686	202134	491082	464877	735501	4679682	4756184	5851575	k__Bacteria;p__Pseudomonadota;c__Alphaproteobacteria;o__Rhodospirillales;f__Acetobacteraceae;g__Acetobacter;s__Acetobacter_tropicalis
NCBI1041607	0	0	0	1	0	0	0	0	0	0	0	0	0	1	0	k__Fungi;p__Ascomycota;c__Saccharomycetes;o__Saccharomycetales;f__Phaffomycetaceae;g__Wickerhamomyces;s__Wickerhamomyces_ciferrii
NCBI1042	0	0	0	0	1	2	0	0	0	0	0	0	0	0	0	k__Bacteria;p__Pseudomonadota;c__Alphaproteobacteria;o__Sphingomonadales;f__Erythrobacteraceae;g__Erythrobacter;s__Erythrobacter_sp.

NCBI1042133	0	0	0	0	0	0	0	0	0	0	0	8	0	0	0	k__Fungi;p__Ascomycota;c__Sordariomycetes;o__Hypocreales;f__Nectriaceae;g__Fusarium;s__Fusarium_musae
NCBI104336	6	0	8	0	2	8	1	3	0	0	0	1	0	0	0	k__Bacteria;p__Actinomycetota;c__Actinomycetes;o__Micrococcales;f__Microbacteriaceae;g__Microbacterium;s__Microbacterium_foliorum
NCBI104355	0	0	0	0	0	0	0	0	1	0	0	0	0	0	0	k__Fungi;p__Basidiomycota;c__Agaricomycetes;o__Gloeophyllales;f__Gloeophyllaceae;g__Gloeophyllum;s__Gloeophyllum_trabeum
NCBI1045317	0	0	1	0	1	0	0	0	0	0	0	0	0	0	0	k__Bacteria;p__Pseudomonadota;c__Alphaproteobacteria;o__Sphingomonadales;f__Sphingomonadaceae;g__Sphingomonas;s__Sphingomonas_lutea
NCBI1045808	0	0	0	0	0	20	0	0	0	0	0	0	0	0	0	k__Bacteria;p__Actinomycetota;c__Actinomycetes;o__Mycobacteriales;f__Nocardiaceae;g__Rhodococcus;s__Rhodococcus_sp._YL-1
NCBI1047172	0	0	0	0	0	1	0	0	0	0	0	0	0	0	0	k__Bacteria;p__Actinomycetota;c__Actinomycetes;o__Mycobacteriales;f__Nocardiaceae;g__Nocardia;s__Nocardia_sp._CS682
NCBI1049	0	0	0	0	0	2	0	0	0	0	0	0	0	0	0	k__Bacteria;p__Pseudomonadota;c__Gammaproteobacteria;o__Chromatiales;f__Chromatiaceae;g__Allochromatium;s__Allochromatium_vinosum
NCBI104955	1	6	0	4	2	6	1	1	0	2	2	3	1	0	1	k__Bacteria;p__Bacillota;c__Bacilli;o__Lactobacillales;f__Lactobacillaceae;g__Limosilactobacillus;s__Limosilactobacillus_frumenti
NCBI10497	0	0	6	0	0	0	0	0	0	0	0	0	0	0	0	k__Bamfordvirae;p__Nucleocytoviricota;c__Pokkesviricetes;o__Asfuvirales;f__Asfarviridae;g__Asfivirus;s__African_swine_fever_virus
NCBI1050370	0	0	0	0	0	2	0	0	0	0	0	0	0	0	0	k__Bacteria;p__Pseudomonadota;c__Betaproteobacteria;o__Burkholderiales;f__;g__Thiomonas;s__Thiomonas_sp._X19

NCBI105219	155	160	308	185	468	2449	195	92	92	60	47	3696	52	39	84	k__Bacteria;p__Pseudomonadota;c__Betaproteobacteria;o__Burkholderiales;f__Burkholderiaceae;g__Ralstonia;s__Ralstonia_mannitolilytica
NCBI1054455	0	0	0	19	0	0	0	0	0	0	0	0	0	0	0	k__Fungi;p__Ascomycota;c__Saccharomycetes;o__Saccharomycetales;f__Saccharomycetaceae;g__;s__uncultured_Zygosaccharomyces
NCBI1055101	0	0	0	0	0	1	0	0	0	0	0	0	0	0	0	k__Bacteria;p__Pseudomonadota;c__Gammaproteobacteria;o__Oceanospirillales;f__Halomonadaceae;g__Cobetia;s__Cobetia_pacifica
NCBI105560	0	0	0	0	0	92	0	0	0	0	0	0	0	0	3	k__Bacteria;p__Pseudomonadota;c__Betaproteobacteria;o__Burkholderiales;f__Sphaerotilaceae;g__Methylibium;s__Methylibium_petroleiphilum
NCBI105841	0	0	1	0	0	9	0	0	0	0	0	1	0	0	0	k__Bacteria;p__Bacillota;c__Clostridia;o__Eubacteriales;f__Lachnospiraceae;g__Anaerostipes;s__Anaerostipes_caccae
NCBI1063	0	0	0	0	0	4	0	0	0	0	0	0	0	0	0	k__Bacteria;p__Pseudomonadota;c__Alphaproteobacteria;o__Rhodobacterales;f__Paracoccaceae;g__Cereibacter;s__Cereibacter_sphaeroides
NCBI106590	3	7	0	0	9	246	1	0	6	0	0	1	2	0	2	k__Bacteria;p__Pseudomonadota;c__Betaproteobacteria;o__Burkholderiales;f__Burkholderiaceae;g__Cupriavidus;s__Cupriavidus_necator
NCBI106592	15	8	1	6	39	284	16	0	2	3	0	20	0	0	2	k__Bacteria;p__Pseudomonadota;c__Alphaproteobacteria;o__Hyphomicrobiales;f__Rhizobiaceae;g__Ensifer;s__Ensifer_adhaerens
NCBI106648	2	5	11	3	16	29	9	0	0	0	0	3	3	0	0	k__Bacteria;p__Pseudomonadota;c__Gammaproteobacteria;o__Moraxellales;f__Moraxellaceae;g__Acinetobacter;s__Acinetobacter_bereziniae
NCBI106649	20	15	4	51	82	27	26	9	8	10	9	90	1	1	7	k__Bacteria;p__Pseudomonadota;c__Gammaproteobacteria;o__Moraxellales;f__Moraxellaceae;g__Acinetobacter;s__Acinetobacter_guillouiae

NCBI106654	4	0	1	2	5	11	0	0	0	0	2	7	8	3	8	k__Bacteria;p__Pseudomonadota;c__Gammaproteobacteria;o__Moraxellales;f__Moraxellaceae;g__Acinetobacter;s__Acinetobacter_nosocomialis
NCBI1069	0	0	0	0	0	0	0	2	0	0	0	0	0	0	0	k__Bacteria;p__Pseudomonadota;c__Alphaproteobacteria;o__Hyphomicrobiales;f__Hyphomicrobiaceae;g__Rhodomicrobium;s__Rhodomicrobium_vannielii
NCBI1069201	0	0	0	1	1	3	0	0	0	0	0	0	0	0	0	k__Fungi;p__Ascomycota;c__Eurotiomycetes;o__Eurotiales;f__Aspergillaceae;g__Aspergillus;s__Aspergillus_luchuensis
NCBI1071072	0	0	0	0	0	120	0	0	0	0	0	6	0	0	0	k__Bacteria;p__Bacillota;c__Bacilli;o__Bacillales;f__Bacillaceae;g__Bacillus;s__Bacillus_sp._NSP2.1
NCBI1071078	0	0	0	0	0	0	0	0	0	0	0	0	1	0	0	k__Bacteria;p__Bacillota;c__Bacilli;o__Bacillales;f__Bacillaceae;g__Bacillus;s__Bacillus_sp._NSP9.1
NCBI1075	0	0	0	0	0	1	0	0	0	0	0	0	0	0	0	k__Bacteria;p__Pseudomonadota;c__Alphaproteobacteria;o__Rhodobacterales;f__Paracoccaceae;g__Fuscovulum;s__Fuscovulum_blasticum
NCBI1076	1	4	2	11	14	140	4	1	1	0	0	2	1	2	2	k__Bacteria;p__Pseudomonadota;c__Alphaproteobacteria;o__Hyphomicrobiales;f__Nitrobacteraceae;g__Rhodopseudomonas;s__Rhodopseudomonas_palustris
NCBI1076124	0	0	0	0	0	16	0	0	0	0	0	0	0	0	0	k__Bacteria;p__Actinomycetota;c__Actinomycetes;o__Micromonosporales;f__Micromonosporaceae;g__Phytohabitans;s__Phytohabitans_flavus
NCBI1076596	2699	2523	2761	6179	6113	3920	5041	3098	4499	36345	34234	53486	78382	83813	97546	k__Bacteria;p__Pseudomonadota;c__Alphaproteobacteria;o__Rhodospirillales;f__Acetobacteraceae;g__Acetobacter;s__Acetobacter_persici
NCBI1077935	0	1	0	0	0	10	0	0	0	0	0	0	0	0	0	k__Bacteria;p__Pseudomonadota;c__Alphaproteobacteria;o__Rhodobacterales;f__Paracoccaceae;g__Paracoccus;s__Paracoccus_zhejiangensis
NCBI1078471	0	0	0	2	6	51	0	0	0	0	0	1	0	0	0	k__Bacteria;p__Actinomycetota;c__Actinomycetes;o__Micrococcales;f__Ornithinimicrobiaceae;g__Serinicoccu

																s;s__Serinicoccus_profundi
NCBI1080349	0	0	0	0	0	0	0	6	0	0	0	0	0	0	0	k__Fungi;p__Ascomycota;c__Saccharomycetes;o__Saccharomycetales;f__Saccharomycetaceae;g__Saccharomyces;s__Saccharomyces_eubayanus
NCBI108150	0	0	0	0	0	0	0	0	0	0	0	1	0	0	0	k__Bacteria;p__Bacillota;c__Clostridia;o__Thermoanaerobacterales;f__Thermoanaerobacteraceae;g__Thermoanaerobacter;s__Thermoanaerobacter_italicus
NCBI1081866	0	0	0	0	0	1	0	0	0	0	0	0	0	0	0	k__Bacteria;p__Pseudomonadota;c__Gammaproteobacteria;o__Oceanospirillales;f__Halomonadaceae;g__Halomonas;s__Halomonas_socia
NCBI1082851	0	0	0	0	0	4	0	0	0	0	0	0	0	0	0	k__Bacteria;p__Pseudomonadota;c__Betaproteobacteria;o__Burkholderiales;f__Comamonadaceae;g__Comamonas;s__Comamonas_serinivorans
NCBI1084	0	0	0	0	0	2	0	0	0	0	0	0	0	0	0	k__Bacteria;p__Pseudomonadota;c__Alphaproteobacteria;o__Rhodospirillales;f__Rhodospirillaceae;g__Pararhodospirillum;s__Pararhodospirillum_photometricum
NCBI108486	0	0	0	0	4	0	0	0	0	0	0	0	0	0	0	k__Bacteria;p__Actinomycetota;c__Actinomycetes;o__Mycobacteriales;f__Corynebacteriaceae;g__Corynebacterium;s__Corynebacterium_falsenii
NCBI108980	3	0	0	0	20	14	8	0	3	0	0	0	0	0	0	k__Bacteria;p__Pseudomonadota;c__Gammaproteobacteria;o__Moraxellales;f__Moraxellaceae;g__Acinetobacter;s__Acinetobacter_ursingii
NCBI108981	0	0	1	3	19	6	5	1	1	3	0	4	5	0	0	k__Bacteria;p__Pseudomonadota;c__Gammaproteobacteria;o__Moraxellales;f__Moraxellaceae;g__Acinetobacter;s__Acinetobacter_schindleri
NCBI1093900	0	0	0	0	1	0	0	0	0	0	0	0	0	0	0	k__Fungi;p__Ascomycota;c__Sordariomycetes;o__;f__Thyridiaceae;g__Thyridium;s__Thyridium_curvatum
NCBI1094342	0	0	0	0	0	3	0	0	0	0	0	1	0	0	0	k__Bacteria;p__Pseudomonadota;c__Gammaproteobacteria;o__Oceanospirillales;f__Alcanivoracaceae;g__Alloalc

																anivorax;s__Alloalcanivorax_xenomutans
NCBI1100043	0	0	0	4	2	2	0	0	0	0	0	0	0	0	0	k__Viruses;p__;c__;o__;f__;g__;s__Apis_mellifera_filamentous_virus
NCBI11008	10	9	9	2	2	1	4	3	6	3	2	1	0	1	1	k__Orthornavirae;p__Duplornaviricota;c__Chrymotiviricetes;o__Ghabrivirales;f__Totiviridae;g__Totivirus;s__Saccharomyces_cerevisiae_virus_L-A
NCBI110319	0	0	0	0	0	70	0	0	0	0	0	0	0	0	0	k__Bacteria;p__Actinomycetota;c__Actinomycetes;o__Propionibacteriales;f__Nocardioidaceae;g__Nocardioides;s__Nocardioides_sp._CF8
NCBI110321	0	0	0	0	0	49	0	0	0	0	0	0	0	0	0	k__Bacteria;p__Pseudomonadota;c__Alphaproteobacteria;o__Hyphomicrobiales;f__Rhizobiaceae;g__Sinorhizobium;s__Sinorhizobium_medicae
NCBI1108595	0	0	0	0	0	1	0	0	0	0	0	0	0	0	0	k__Bacteria;p__Pseudomonadota;c__Betaproteobacteria;o__Neisseriales;f__Chromobacteriaceae;g__Chromobacterium;s__Chromobacterium_vaccinii
NCBI1108849	3	1	6	18	9	9	10	0	1	0	0	39	0	1	0	k__Fungi;p__Ascomycota;c__Eurotiomycetes;o__Eurotiales;f__Aspergillaceae;g__Penicillium;s__Penicillium_rubens
NCBI111015	0	0	0	0	0	4	0	0	0	0	0	0	0	0	0	k__Bacteria;p__Actinomycetota;c__Actinomycetes;o__Actinomycetales;f__Actinomycetaceae;g__Actinomyces;s__Actinomyces_radicidentis
NCBI1112	0	0	0	0	1	17	0	0	0	0	0	1	0	0	0	k__Bacteria;p__Pseudomonadota;c__Alphaproteobacteria;o__Sphingomonadales;f__Erythrobacteraceae;g__Erythrobacter;s__Erythrobacter_neustonensis
NCBI1113698	8	0	0	0	0	29	0	0	0	0	0	0	0	0	0	k__Bacteria;p__Pseudomonadota;c__Alphaproteobacteria;o__Caulobacterales;f__Caulobacteraceae;g__Brevundimonas;s__Brevundimonas_sp._NBRC_101024
NCBI1114880	0	0	0	0	21	0	0	0	0	0	0	0	0	0	0	k__Bacteria;p__Pseudomonadota;c__Gammaproteobacteria;o__Aeromonadales;f__Aeromonadaceae;g__Aeromon

																as;s__Aeromonas_australiensis
NCBI1114970	0	0	0	0	0	0	56	0	0	0	0	0	0	0	0	k__Bacteria;p__Pseudomonadota;c__Gammaproteobacteria;o__Pseudomonadales;f__Pseudomonadaceae;g__Pseudomonas;s__Pseudomonas_ogarae
NCBI1115758	0	0	0	0	0	5	3	0	0	0	0	7	0	0	0	k__Bacteria;p__Bacillota;c__Clostridia;o__Eubacteriales;f__Peptostreptococcaceae;g__Romboutsia;s__Romboutsia_ilealis
NCBI1118202	0	0	0	1	0	0	0	0	1	0	0	0	0	0	0	k__Bacteria;p__Bacteroidota;c__Flavobacteriia;o__Flavobacteriales;f__Weeksellaceae;g__Cruoricaptor;s__Cruoricaptor_ignavus
NCBI113107	0	0	0	0	4	0	0	0	0	0	0	6	0	0	0	k__Bacteria;p__Bacillota;c__Bacilli;o__Lactobacillales;f__Streptococcaceae;g__Streptococcus;s__Streptococcus_australis
NCBI1131822	0	0	0	0	1	0	0	0	0	0	0	0	0	0	0	k__Bacteria;p__Verrucomicrobiota;c__Verrucomicrobiae;o__Verrucomicrobiales;f__Akkermansiaceae;g__Akkermansia;s__uncultured_Akkermansia_sp._SMG25
NCBI11320	0	0	0	0	1	1	0	0	0	0	0	0	0	0	0	k__Orthornavirae;p__Negarnaviricota;c__Insthoviricetes;o__Articulavirales;f__Orthomyxoviridae;g__Alphainfluenzavirus;s__Influenza_A_virus
NCBI1134687	0	0	0	0	27	253	0	0	0	0	0	0	0	15	0	k__Bacteria;p__Pseudomonadota;c__Gammaproteobacteria;o__Enterobacterales;f__Enterobacteriaceae;g__Klebsiella;s__Klebsiella_michiganensis
NCBI113608	0	0	0	0	0	0	0	0	0	1	0	0	0	0	0	k__Fungi;p__Ascomycota;c__Saccharomycetes;o__Saccharomycetales;f__Saccharomycetaceae;g__Tetrapisispora;s__Tetrapisispora_phaffii
NCBI1136522	0	0	0	0	0	2	0	0	0	0	0	0	0	0	0	k__Bacteria;p__;c__;o__;f__;g__;s__bacterium_AK-OB05
NCBI1137095	0	0	0	0	0	2	0	1	0	0	1	0	0	0	0	k__Bacteria;p__Cyanobacteriota;c__Cyanophyceae;o__Nostocales;f__Scytonemataceae;g__Scytonema;s__Scyto

																nema_sp._HK-05
NCBI1138189	0	0	0	0	0	1	1	0	0	0	0	0	0	0	0	k__Bacteria;p__Pseudomonadota;c__Alphaproteobacteria;o__Hyphomicrobiales;f__Rhizobiaceae;g__Rhizobium;s__Rhizobium_bangladeshense
NCBI1138194	0	0	0	0	0	1	0	0	0	0	0	0	0	0	0	k__Bacteria;p__Pseudomonadota;c__Alphaproteobacteria;o__Hyphomicrobiales;f__Rhizobiaceae;g__Rhizobium;s__Rhizobium_lentis
NCBI1138371	0	0	0	0	0	2	0	0	0	0	0	0	0	0	0	k__Bacteria;p__Pseudomonadota;c__Gammaproteobacteria;o__Pseudomonadales;f__Pseudomonadaceae;g__Pseudomonas;s__Pseudomonas_sp._L-1
NCBI1138383	0	0	0	0	0	0	0	0	3	0	0	0	0	0	0	k__Bacteria;p__Actinomycetota;c__Actinomycetes;o__Mycobacteriales;f__Mycobacteriaceae;g__Mycobacterium;s__Mycobacterium_paraintracellulare
NCBI114053	44	31	33	43	14	70	40	83	69	180	104	43	125	254	163	k__Bacteria;p__Pseudomonadota;c__Gammaproteobacteria;o__Alteromonadales;f__Pseudoalteromonadaceae;g__Pseudoalteromonas;s__uncultured_Pseudoalteromonas_sp.
NCBI114090	25	39	26	58	53	16	28	41	41	36	35	53	15	17	11	k__Bacteria;p__Bacillota;c__Bacilli;o__Lactobacillales;f__Lactobacillaceae;g__Pediococcus;s__Pediococcus_inopinatus
NCBI1141883	0	0	0	7	0	30	0	0	0	0	0	0	0	0	0	k__Bacteria;p__Pseudomonadota;c__Betaproteobacteria;o__Burkholderiales;f__Oxalobacteraceae;g__Massilia;s__Massilia_putida
NCBI114524	0	0	0	0	0	2	0	0	0	0	2	0	0	0	0	k__Fungi;p__Ascomycota;c__Saccharomycetes;o__Saccharomycetales;f__Saccharomycetaceae;g__Saccharomyces;s__Saccharomyces_kudriavzevii
NCBI114527	0	0	0	0	0	0	1	0	0	0	0	3	0	0	0	k__Bacteria;p__Bacillota;c__Clostridia;o__Eubacteriales;f__Eubacteriales_Family_XIII._Incertae_Sedis;g__Mogibacterium;s__Mogibacterium_diversum

NCBI114616	0	0	0	0	0	0	0	0	1	0	0	0	0	0	0	k__Bacteria;p__Pseudomonadota;c__Alphaproteobacteria;o__Hyphomicrobiales;f__Methylobacteriaceae;g__Methylobacterium;s__Methylobacterium_nodulans
NCBI1147042	0	0	0	0	0	0	0	0	0	0	0	10	0	0	0	k__Heunggongvirae;p__Uroviricota;c__Caudoviricetes;o__;f__Azeredovirinae;g__;s__Staphylococcus_phage_StB12
NCBI1148	0	0	0	0	2229	0	0	0	0	0	0	0	0	0	0	k__Bacteria;p__Cyanobacteriota;c__Cyanophyceae;o__Synechococcales;f__Merismopediaceae;g__Synechocystis;s__Synechocystis_sp._PCC_6803
NCBI1148157	0	0	0	0	0	4	0	0	0	0	0	14	0	0	0	k__Bacteria;p__Pseudomonadota;c__Gammaproteobacteria;o__Moraxellales;f__Moraxellaceae;g__Acinetobacter;s__Acinetobacter_oleivorans
NCBI1149133	0	1	0	0	0	1	0	0	0	0	0	0	0	0	0	k__Bacteria;p__Pseudomonadota;c__Gammaproteobacteria;o__Pseudomonadales;f__Pseudomonadaceae;g__Pseudomonas;s__Pseudomonas_furukawaii
NCBI1151742	0	0	0	0	0	0	0	0	0	0	1	0	0	0	0	k__Bacteria;p__Bacillota;c__Bacilli;o__Lactobacillales;f__Streptococcaceae;g__Lactococcus;s__Lactococcus_taiwanensis
NCBI1155384	1	5	0	16	7	28	2	4	3	61	33	45	78	56	89	k__Bacteria;p__Actinomycetota;c__Actinomycetes;o__Micrococcales;f__Micrococcaceae;g__Glutamicibacter;s__Glutamicibacter_sp._ZJUTW
NCBI115553	0	0	0	0	0	1	0	0	0	0	0	0	0	0	0	k__Bacteria;p__Pseudomonadota;c__Gammaproteobacteria;o__Oceanospirillales;f__Halomonadaceae;g__Halomonas;s__Halomonas_sulfidaeris
NCBI1156431	0	0	0	0	4	3	0	0	0	0	0	25	0	0	0	k__Bacteria;p__Bacillota;c__Bacilli;o__Lactobacillales;f__Streptococcaceae;g__Streptococcus;s__Streptococcus_ilei
NCBI115808	0	0	0	0	0	0	0	0	1	0	0	0	0	0	0	k__Bacteria;p__Pseudomonadota;c__Alphaproteobacteria;o__Hyphomicrobiales;f__Nitrobacteraceae;g__Bradyrhizobium;s__Bradyrhizobium_sp._ORS_285

NCBI1160721	1	0	1	11	0	58	0	0	0	0	0	18	0	0	0	k__Bacteria;p__Bacillota;c__Clostridia;o__Eubacteriales;f__Oscillospiraceae;g__Ruminococcus;s__Ruminococcus_bicirculans
NCBI116085	0	0	0	0	1	32	1	0	1	0	0	22	0	0	0	k__Bacteria;p__Bacillota;c__Clostridia;o__Eubacteriales;f__Lachnospiraceae;g__Coprococcus;s__Coprococcus_catus
NCBI116188	0	0	0	0	5	0	0	0	0	0	0	0	0	0	0	k__Bacteria;p__Actinomycetota;c__Actinomycetes;o__Kitasatosporales;f__Streptomycetaceae;g__Streptomyces;s__Streptomyces_coeruleorubidus
NCBI1161942	0	0	0	0	0	0	0	0	0	0	0	1	0	0	0	k__Bacteria;p__Bacillota;c__Clostridia;o__Eubacteriales;f__Oscillospiraceae;g__Ruminococcus;s__Ruminococcus_champanellensis
NCBI1164002	0	0	0	1	0	0	0	0	0	0	0	6	0	0	0	k__Bacteria;p__Actinomycetota;c__Actinomycetes;o__Mycobacteriales;f__Corynebacteriaceae;g__;s__Corynebacteriaceae_bacterium_'ARUP_UnID_227'
NCBI1169025	0	0	0	1	0	3	0	0	0	0	0	0	0	0	0	k__Bacteria;p__Actinomycetota;c__Actinomycetes;o__Kitasatosporales;f__Streptomycetaceae;g__Streptomyces;s__Streptomyces_pratensis
NCBI1170708	12	6	0	0	20	187	19	0	19	0	0	0	0	0	0	k__Bacteria;p__Pseudomonadota;c__Betaproteobacteria;o__Burkholderiales;f__Comamonadaceae;g__Comamonas;s__Comamonas_sp._KV11
NCBI117187	0	0	0	5	0	20	0	0	0	6	0	8	0	0	0	k__Fungi;p__Ascomycota;c__Sordariomycetes;o__Hypocreales;f__Nectriaceae;g__Fusarium;s__Fusarium_verticillioides
NCBI117207	2	0	1	1	3	57	2	0	2	0	0	0	0	0	0	k__Bacteria;p__Pseudomonadota;c__Alphaproteobacteria;o__Sphingomonadales;f__Sphingomonadaceae;g__Sphingopyxis;s__Sphingopyxis_alaskensis
NCBI1173061	0	0	0	0	0	4	0	0	0	0	0	0	0	0	0	k__Fungi;p__Ascomycota;c__Saccharomycetes;o__Saccharomycetales;f__Dipodascaceae;g__Geotrichum;s__Geotrichum_candidum

NCBI1173284	0	0	0	3	0	0	0	0	0	0	0	0	0	0	0	k__Bacteria;p__Pseudomonadota;c__Gammaproteobacteria;o__Pseudomonadales;f__Pseudomonadaceae;g__Pseudomonas;s__Pseudomonas_sp._R3-52-08
NCBI1173756	0	0	0	0	0	4	0	0	0	0	0	0	0	0	0	k__Heunggongvirae;p__Uroviricota;c__Caudoviricetes;o__;f__Casjensviridae;g__Chivirus;s__Chivirus_BSPM4
NCBI1174673	0	0	0	0	0	7	0	0	0	0	0	0	0	0	0	k__Fungi;p__Ascomycota;c__Pezizomycetes;o__Pezizales;f__Morchellaceae;g__Morchella;s__Morchella_importuna
NCBI1174677	0	0	0	0	0	319	0	0	0	0	0	0	0	0	0	k__Fungi;p__Ascomycota;c__Pezizomycetes;o__Pezizales;f__Morchellaceae;g__Morchella;s__Morchella_sextelata
NCBI11757	0	0	0	0	0	0	1	0	0	0	0	0	0	0	0	k__Pararnavirae;p__Artverviricota;c__Revtraviricetes;o__Ortervirales;f__Retroviridae;g__Betaretrovirus;s__Mouse_mammary_tumor_virus
NCBI1176198	0	0	0	0	0	2	0	0	0	0	0	0	0	0	0	k__Bacteria;p__Actinomycetota;c__Actinomycetes;o__Kitasatosporales;f__Streptomycetaceae;g__Streptomyces;s__Streptomyces_harbinensis
NCBI1176257	3	0	0	0	0	53	3	0	0	0	0	0	0	0	0	k__Bacteria;p__Pseudomonadota;c__Gammaproteobacteria;o__Pseudomonadales;f__Pseudomonadaceae;g__Stutzerimonas;s__[Pseudomonas]_zhaodongensis
NCBI1176536	0	0	0	0	0	4	2	0	0	1	0	0	0	0	0	k__Bacteria;p__Pseudomonadota;c__Alphaproteobacteria;o__Sphingomonadales;f__Sphingomonadaceae;g__Novosphingobium;s__Novosphingobium_ginsenosidimutans
NCBI1176649	0	2	0	4	0	2	0	0	0	0	0	0	0	0	0	k__Bacteria;p__Pseudomonadota;c__Alphaproteobacteria;o__Hyphomicrobiales;f__Rhizobiaceae;g__Agrobacterium;s__Agrobacterium_fabrum
NCBI1177574	0	0	0	1	0	1	0	1	0	0	0	2	0	0	0	k__Bacteria;p__Bacteroidota;c__Bacteroidia;o__Bacteroidales;f__Prevotellaceae;g__Prevotella;s__Prevotella_jejuni

NCBI1177712	93	111	104	309	229	239	112	65	124	333	344	461	2890	2857	3506	k__Bacteria;p__Pseudomonadota;c__Alphaproteobacteria;o__Rhodospirillales;f__Acetobacteraceae;g__Komagataeibacter;s__Komagataeibacter_medellinensis
NCBI11786	0	0	0	0	0	0	35	0	1	0	0	1	0	0	0	k__Pararnavirae;p__Artverviricota;c__Revtraviricetes;o__Ortervirales;f__Retroviridae;g__Gammaretrovirus;s__Murine_leukemia_virus
NCBI1179670	0	0	0	11	0	37	0	0	0	0	0	0	0	0	0	k__Bacteria;p__Actinomycetota;c__Actinomycetes;o__Micrococcales;f__Micrococcaceae;g__Micrococcus;s__Micrococcus_sp._KBS0714
NCBI1181499	0	0	0	0	0	12	0	0	0	0	0	0	0	0	0	k__Bacteria;p__;c__;o__;f__;g__;s__uncultured_bacterium_contig00007
NCBI1181511	0	0	0	0	0	1	0	0	0	0	0	0	0	0	0	k__Bacteria;p__;c__;o__;f__;g__;s__uncultured_bacterium_contig00021
NCBI1181518	0	0	0	0	0	19	0	0	0	0	0	0	0	0	0	k__Bacteria;p__;c__;o__;f__;g__;s__uncultured_bacterium_contig00029
NCBI1181523	0	0	0	0	0	4	0	0	0	0	0	0	0	0	0	k__Bacteria;p__;c__;o__;f__;g__;s__uncultured_bacterium_contig00034
NCBI1181524	0	0	0	0	0	7	0	0	0	0	0	0	0	0	0	k__Bacteria;p__;c__;o__;f__;g__;s__uncultured_bacterium_contig00036
NCBI1181528	0	0	0	0	0	2	0	0	0	0	0	0	0	0	0	k__Bacteria;p__;c__;o__;f__;g__;s__uncultured_bacterium_contig00040
NCBI1181548	0	0	0	0	0	1	0	0	0	0	0	0	0	0	0	k__Bacteria;p__;c__;o__;f__;g__;s__uncultured_bacterium_contig00066
NCBI1181559	0	0	0	0	0	1	0	0	0	0	0	0	0	0	0	k__Bacteria;p__;c__;o__;f__;g__;s__uncultured_bacterium_contig00086
NCBI1181561	0	0	0	0	0	5	0	0	0	0	0	0	0	0	0	k__Bacteria;p__;c__;o__;f__;g__;s__uncultured_bacterium_contig00088
NCBI1181572	0	0	0	0	0	4	0	0	0	0	0	0	0	0	0	k__Bacteria;p__;c__;o__;f__;g__;s__uncultured_bacterium_contig00106

NCBI1181590	0	0	0	0	0	0	0	0	0	0	0	1	0	0	0	k__Bacteria;p__;c__;o__;f__;g__;s__uncultured_bacterium_contig00151
NCBI1183401	30	24	10	8	87	1000	43	5	0	15	10	21	9	0	4	k__Bacteria;p__Pseudomonadota;c__Alphaproteobacteria;o__Hyphomicrobiales;f__Rhizobiaceae;g__Agrobacterium;s__Agrobacterium_fabacearum
NCBI1183410	0	0	0	0	0	258	0	0	0	0	0	0	0	0	0	k__Bacteria;p__Pseudomonadota;c__Alphaproteobacteria;o__Hyphomicrobiales;f__Rhizobiaceae;g__Agrobacterium;s__Agrobacterium_tomkonis
NCBI1183412	0	0	0	0	0	20	0	0	0	0	0	0	0	0	0	k__Bacteria;p__Pseudomonadota;c__Alphaproteobacteria;o__Hyphomicrobiales;f__Rhizobiaceae;g__Agrobacterium;s__Agrobacterium_deltaense
NCBI1183413	0	0	3	0	2	39	2	0	0	1	0	0	0	1	0	k__Bacteria;p__Pseudomonadota;c__Alphaproteobacteria;o__Hyphomicrobiales;f__Rhizobiaceae;g__Agrobacterium;s__Agrobacterium_salinitolerans
NCBI1187904	0	2	0	0	0	13	0	0	5	0	0	0	0	0	0	k__Fungi;p__Ascomycota;c__Dothideomycetes;o__Pleosporales;f__Pleosporaceae;g__Alternaria;s__Alternaria_burnsii
NCBI1188315	0	0	0	0	7	0	0	0	0	0	0	0	0	0	0	k__Bacteria;p__Actinomycetota;c__Actinomycetes;o__Kitasatosporales;f__Streptomycetaceae;g__Streptomyces;s__Streptomyces_sp._AgN23
NCBI1190813	0	0	0	0	0	0	0	0	0	0	0	10	0	0	0	k__Bacteria;p__Pseudomonadota;c__Gammaproteobacteria;o__Alteromonadales;f__Pseudoalteromonadaceae;g__Pseudoalteromonas;s__Pseudoalteromonas_shioyasakiensis
NCBI1191711	0	0	0	0	0	1	0	0	0	0	0	0	0	0	0	k__Fungi;p__Ascomycota;c__Eurotiomycetes;o__Eurotiales;f__Aspergillaceae;g__Aspergillus;s__Aspergillus_brunneoviolaceus
NCBI1192162	0	0	0	0	0	11	0	0	0	0	0	0	0	0	0	k__Bacteria;p__Pseudomonadota;c__Betaproteobacteria;o__Neisseriales;f__Chromobacteriaceae;g__Vogesella;s__Vogesella_sp._LIG4

NCBI119219	7	7	3	0	18	45	7	0	3	0	0	0	0	0	0	k__Bacteria;p__Pseudomonadota;c__Betaproteobacteria;o__Burkholderiales;f__Burkholderiaceae;g__Cupriavidus;s__Cupriavidus_metallidurans
NCBI1196081	0	0	1	0	2	0	0	0	0	0	0	2	1	0	0	k__Fungi;p__Ascomycota;c__Eurotiomycetes;o__Eurotiales;f__Trichocomaceae;g__Talaromyces;s__Talaromyces_amestolkiae
NCBI1196083	1	0	0	2	3	6	0	0	0	0	0	0	0	0	0	k__Bacteria;p__Pseudomonadota;c__Betaproteobacteria;o__Neisseriales;f__Neisseriaceae;g__Snodgrassella;s__Snodgrassella_alvi
NCBI1196095	0	0	0	0	5	1	1	0	0	0	0	0	0	0	0	k__Bacteria;p__Pseudomonadota;c__Gammaproteobacteria;o__Orbales;f__Orbaceae;g__Gilliamella;s__Gilliamella_apicola
NCBI1197717	0	0	0	0	0	7	0	0	0	0	0	0	0	0	0	k__Bacteria;p__Synergistota;c__Synergistia;o__Synergistales;f__Synergistaceae;g__Cloacibacillus;s__Cloacibacillus_porcorum
NCBI119953	0	0	0	0	0	3	0	0	0	0	0	0	0	0	0	k__Fungi;p__Ascomycota;c__Dothideomycetes;o__Pleosporales;f__Pleosporaceae;g__Alternaria;s__Alternaria_atra
NCBI120107	2	11	0	0	2	169	0	0	0	0	5	7	0	0	0	k__Bacteria;p__Pseudomonadota;c__Alphaproteobacteria;o__Sphingomonadales;f__Sphingomonadaceae;g__Sphingobium;s__Sphingobium_cloacae
NCBI1204692	0	0	0	0	0	11	0	0	0	0	0	0	0	0	0	k__Bacteria;p__Pseudomonadota;c__Betaproteobacteria;o__Burkholderiales;f__Burkholderiaceae;g__Limnobacter;s__Limnobacter_sp._2A6
NCBI1204693	0	2	2	0	0	15	0	0	0	0	0	0	0	1	0	k__Bacteria;p__Pseudomonadota;c__Betaproteobacteria;o__Burkholderiales;f__Burkholderiaceae;g__Limnobacter;s__Limnobacter_sp._2C4
NCBI1204695	1	0	0	0	0	4	0	0	0	0	0	0	0	0	0	k__Bacteria;p__Pseudomonadota;c__Betaproteobacteria;o__Burkholderiales;f__Burkholderiaceae;g__Limnobacter;s__Limnobacter_sp._CR2A4

NCBI12055	0	0	0	0	0	0	18	0	1	0	0	0	0	0	0	k__Orthornavirae;p__Kitrinoviricota;c__Tolucaviricetes;o__Tolivirales;f__Tombusviridae;g__Alphanecrovirus;s__Tobacco_necrosis_virus_A
NCBI1211	51	33	25	55	244	388	12	3	25	3	3	5	18	0	6	k__Bacteria;p__Cyanobacteriota;c__;o__;f__;g__;s__uncultured_cyanobacterium
NCBI121428	215	197	179	15	494	8950	289	30	61	28	25	68	31	34	42	k__Bacteria;p__Pseudomonadota;c__Alphaproteobacteria;o__Sphingomonadales;f__Sphingomonadaceae;g__Sphingobium;s__Sphingobium_xenophagum
NCBI1215031	6	6	2	13	6	138	2	6	1	3	1	2868	1	0	1	k__Bacteria;p__Bacillota;c__Bacilli;o__Bacillales;f__Bacillaceae;g__Neobacillus;s__Neobacillus_thermocopriae
NCBI121627	0	2	0	0	3	0	0	1	0	0	0	0	0	0	0	k__Fungi;p__Ascomycota;c__Eurotiomycetes;o__Eurotiales;f__Trichocomaceae;g__Talaromyces;s__Talaromyces_rugulosus
NCBI1216932	0	0	0	0	0	1	0	0	0	0	0	0	0	0	0	k__Bacteria;p__Bacillota;c__Clostridia;o__Eubacteriales;f__Clostridiaceae;g__Clostridium;s__Clostridium_bornimense
NCBI121719	0	5	4	0	21	372	15	2	0	0	0	7	0	0	1	k__Bacteria;p__Pseudomonadota;c__Alphaproteobacteria;o__Hyphomicrobiales;f__Stappiaceae;g__Pannonibacter;s__Pannonibacter_phragmitetus
NCBI12181	0	0	0	1	0	2	1	0	1	0	0	0	0	0	0	k__Orthornavirae;p__Kitrinoviricota;c__Alsuviricetes;o__Tymovirales;f__Alphaflexiviridae;g__Potexvirus;s__Papaya_mosaic_virus
NCBI1218493	0	0	0	0	1	3	1	0	2	0	0	0	0	0	0	k__Bacteria;p__Bacillota;c__Bacilli;o__Lactobacillales;f__Lactobacillaceae;g__Lactobacillus;s__Lactobacillus_kullabergensis
NCBI1218494	0	0	1	0	0	1	0	0	0	0	0	0	0	0	0	k__Bacteria;p__Bacillota;c__Bacilli;o__Lactobacillales;f__Lactobacillaceae;g__Lactobacillus;s__Lactobacillus_helsingborgensis
NCBI1220207	2	3	9	36	20	17	3	0	2	2	0	27	0	6	2	k__Fungi;p__Ascomycota;c__Eurotiomycetes;o__Eurotiales;f__Aspergillaceae;g__Aspergillus;s__Aspergillus_p

															uulaauensis
NCBI12204	19	15	6	35	71	96	12	8	7	2	4	2	3	5	6	k__Orthornavirae;p__Pisuviricota;c__Stelpaviricetes;o__Patatavirales;f__Potyviridae;g__Potyvirus;s__Ornithogalum_mosaic_virus
NCBI1221500	0	0	0	1	0	0	0	0	0	0	0	0	0	0	0	k__Bacteria;p__Bacillota;c__Bacilli;o__Bacillales;f__Bacillaceae;g__Fictibacillus;s__Fictibacillus_phosphorivorans
NCBI122355	0	1	0	0	0	0	0	1	0	0	3	0	0	0	0	k__Bacteria;p__Pseudomonadota;c__Gammaproteobacteria;o__Pseudomonadales;f__Pseudomonadaceae;g__Pseudomonas;s__Pseudomonas_psychrophila
NCBI1223566	0	0	0	0	1	0	0	0	0	0	0	0	0	0	0	k__Bacteria;p__Pseudomonadota;c__Alphaproteobacteria;o__Hyphomicrobiales;f__Nitrobacteraceae;g__Bradyrhizobium;s__Bradyrhizobium_sp._CCGE-LA001
NCBI122368	0	0	1	0	0	2	0	0	0	0	0	0	0	0	0	k__Fungi;p__Ascomycota;c__Dothideomycetes;o__Mycosphaerellales;f__Mycosphaerellaceae;g__Cercospora;s__Cercospora_beticola
NCBI1225769	0	0	0	0	1	3	1	0	0	0	0	0	0	0	0	k__Bacteria;p__Pseudomonadota;c__Betaproteobacteria;o__Neisseriales;f__Chromobacteriaceae;g__Paludibacterium;s__Paludibacterium_paludis
NCBI1225883	20	24	25	32	35	79	36	42	43	57	39	27	18	19	16	k__Bacteria;p__Bacillota;c__Bacilli;o__Lactobacillales;f__Lactobacillaceae;g__Oenococcus;s__uncultured_Oenococcus_sp.
NCBI1226757	0	0	0	0	0	5	0	0	0	0	0	21	0	0	0	k__Bacteria;p__Actinomycetota;c__Actinomycetes;o__Kitasatosporales;f__Streptomycetaceae;g__Streptomyces;s__Streptomyces_rapamycinicus
NCBI1229621	0	0	0	0	0	4	0	0	0	0	0	0	0	0	0	k__Bacteria;p__Bacillota;c__Clostridia;o__Eubacteriales;f__Lachnospiraceae;g__Anaerostipes;s__Anaerostipes_rhamnosivorans

NCBI1230998	0	0	0	0	0	2	0	0	0	0	0	0	0	0	0	k__Bacteria;p__Actinomycetota;c__Actinomycetes;o__Mycobacteriales;f__Corynebacteriaceae;g__Corynebacterium;s__Corynebacterium_frankenforstense
NCBI1232667	0	0	0	0	0	21	0	0	0	0	0	0	0	0	0	k__Bacteria;p__Pseudomonadota;c__Betaproteobacteria;o__Burkholderiales;f__Comamonadaceae;g__Comamonas;s__Comamonas_sp._7D-2
NCBI1233873	0	0	0	0	0	0	0	0	0	0	0	41	0	0	0	k__Bacteria;p__Bacillota;c__Bacilli;o__Bacillales;f__Bacillaceae;g__Geobacillus;s__Geobacillus_sp._GHH01
NCBI1234680	0	0	0	0	0	4	0	0	0	0	0	22	0	0	0	k__Bacteria;p__Bacillota;c__Bacilli;o__Lactobacillales;f__Streptococcaceae;g__Streptococcus;s__Streptococcus_rubneri
NCBI1235591	1	0	0	0	0	0	0	0	0	0	0	0	0	0	0	k__Bacteria;p__Pseudomonadota;c__Alphaproteobacteria;o__Hyphomicrobiales;f__;g__Pseudorhodoplanes;s__Pseudorhodoplanes_sinuspersici
NCBI123899	6	3	0	0	3	41	1	0	0	0	0	0	0	0	0	k__Bacteria;p__Pseudomonadota;c__Betaproteobacteria;o__Burkholderiales;f__Alcaligenaceae;g__Bordetella;s__Bordetella_trematum
NCBI1239458	0	0	0	0	0	4	0	0	0	0	0	0	0	0	0	k__Bacteria;p__;c__;o__;f__;g__;s__uncultured_bacterium_contig00044
NCBI1239471	0	0	0	0	0	1	0	0	0	0	0	0	0	0	0	k__Bacteria;p__;c__;o__;f__;g__;s__uncultured_bacterium_contig00096
NCBI1239475	0	0	0	0	0	0	0	0	0	0	0	1	0	0	0	k__Bacteria;p__;c__;o__;f__;g__;s__uncultured_bacterium_contig00116
NCBI1239479	0	0	0	0	0	1	0	0	0	0	0	0	0	0	0	k__Bacteria;p__;c__;o__;f__;g__;s__uncultured_bacterium_contig00122
NCBI1239482	0	0	0	0	0	28	0	0	0	0	0	0	0	0	0	k__Bacteria;p__;c__;o__;f__;g__;s__uncultured_bacterium_contig00126
NCBI1239483	0	0	0	0	0	2	0	0	0	0	0	0	0	0	0	k__Bacteria;p__;c__;o__;f__;g__;s__uncultured_bacterium_contig00128

NCBI1239486	0	0	0	0	0	2	0	0	0	0	0	0	0	0	0	k__Bacteria;p__;c__;o__;f__;g__;s__uncultured_bacterium_contig00131
NCBI1239492	0	0	0	0	0	2	0	0	0	0	0	0	0	0	0	k__Bacteria;p__;c__;o__;f__;g__;s__uncultured_bacterium_contig00148
NCBI1239493	0	0	0	0	0	2	0	0	0	0	0	0	0	0	0	k__Bacteria;p__;c__;o__;f__;g__;s__uncultured_bacterium_contig00155
NCBI1239496	0	0	0	0	0	7	0	0	0	0	0	0	0	0	0	k__Bacteria;p__;c__;o__;f__;g__;s__uncultured_bacterium_contig00164
NCBI1239502	0	0	0	0	0	2	0	0	0	0	0	0	0	0	0	k__Bacteria;p__;c__;o__;f__;g__;s__uncultured_bacterium_contig00179
NCBI1239521	0	0	0	0	0	2	0	0	0	0	0	0	0	0	0	k__Bacteria;p__;c__;o__;f__;g__;s__uncultured_bacterium_contig00232
NCBI1239532	0	0	0	0	0	2	0	0	0	0	0	0	0	0	0	k__Bacteria;p__;c__;o__;f__;g__;s__uncultured_bacterium_contig00286
NCBI1239547	0	0	0	0	0	1	0	0	0	0	0	0	0	0	0	k__Bacteria;p__;c__;o__;f__;g__;s__uncultured_bacterium_contig00362
NCBI1239554	0	0	0	0	0	1	0	0	0	0	0	0	0	0	0	k__Bacteria;p__;c__;o__;f__;g__;s__uncultured_bacterium_contig00418
NCBI1239561	0	0	0	0	0	1	0	0	0	0	0	0	0	0	0	k__Bacteria;p__;c__;o__;f__;g__;s__uncultured_bacterium_contig00449
NCBI1240598	0	0	0	0	1	1	0	0	0	0	0	0	0	0	0	k__Bacteria;p__Pseudomonadota;c__Alphaproteobacteria;o__;f__;g__;s__uncultured_proteobacterium_90H6
NCBI1241979	0	1	0	0	0	0	0	0	0	0	0	0	0	0	0	k__Bacteria;p__Bacteroidota;c__Flavobacteriia;o__Flavobacteriales;f__Weeksellaceae;g__Kaistella;s__Kaistella_carnis
NCBI12438	0	0	0	0	0	0	1	0	0	0	0	0	0	0	0	k__Pararnavirae;p__Artverviricota;c__Revtraviricetes;o__Ortervirales;f__Retroviridae;g__;s__AKT8_retrovirus
NCBI1245	0	0	0	0	0	0	0	0	0	1	0	0	0	0	0	k__Bacteria;p__Bacillota;c__Bacilli;o__Lactobacillales;f__Lactobacillaceae;g__Leuconostoc;s__Leuconostoc_mesenteroides

NCBI1246	0	0	0	1	0	0	0	0	0	0	3	0	0	0	0	k__Bacteria;p__Bacillota;c__Bacilli;o__Lactobacillales;f__Lactobacillaceae;g__Leuconostoc;s__Leuconostoc_lactis
NCBI1247	10	1	0	3	7	19	3	1	6	7	5	14	2	0	0	k__Bacteria;p__Bacillota;c__Bacilli;o__Lactobacillales;f__Lactobacillaceae;g__Oenococcus;s__Oenococcus_oeni
NCBI1249	0	0	0	1	0	2	0	0	0	0	0	0	0	0	0	k__Bacteria;p__Bacillota;c__Bacilli;o__Lactobacillales;f__Lactobacillaceae;g__Weissella;s__Weissella_paramesenteroides
NCBI12519	0	0	0	0	1	0	16	0	0	0	0	0	0	0	0	k__Pararnavirae;p__Artverviricota;c__Revtraviricetes;o__Ortervirales;f__Retroviridae;g__Intracisternal_A-particles;s__Intracisternal_A-type_particle_IAP
NCBI1254	18	18	17	40	38	1109	15	17	19	13	20	81	4	13	2	k__Bacteria;p__Bacillota;c__Bacilli;o__Lactobacillales;f__Lactobacillaceae;g__Pediococcus;s__Pediococcus_acidilactici
NCBI1255	141	128	131	203	260	100	223	258	232	370	227	281	159	191	175	k__Bacteria;p__Bacillota;c__Bacilli;o__Lactobacillales;f__Lactobacillaceae;g__Pediococcus;s__Pediococcus_pentosaceus
NCBI1256563	0	0	0	0	4	0	0	0	0	0	0	0	0	0	0	k__Bacteria;p__;c__;o__;f__;g__;s__uncultured_bacterium_A1Q1_fos_2111
NCBI1260	0	0	0	0	0	3	1	5	0	0	2	0	0	0	0	k__Bacteria;p__Bacillota;c__Tissierellia;o__Tissierellales;f__Peptoniphilaceae;g__Finegoldia;s__Finegoldia_magna
NCBI1263098	0	0	0	0	0	0	10	0	0	0	0	0	0	0	0	k__Bacteria;p__Pseudomonadota;c__Betaproteobacteria;o__Burkholderiales;f__Sutterellaceae;g__Parasutterella;s__uncultured_Parasutterella_sp.
NCBI126385	2	3	0	17	4	12	0	0	1	0	0	0	0	0	0	k__Bacteria;p__Pseudomonadota;c__Gammaproteobacteria;o__Enterobacterales;f__Morganellaceae;g__Providencia;s__Providencia_alcalifaciens
NCBI1264579	0	0	0	0	0	0	0	0	0	1	0	0	0	0	0	k__Archaea;p__Euryarchaeota;c__Halobacteria;o__Halobacteriales;f__Halococcaceae;g__Halococcus;s__Haloco

																ccus_sediminicola
NCBI126673	0	0	0	0	0	4	0	0	0	0	0	0	0	0	0	k__Bacteria;p__Actinomycetota;c__Actinomycetes;o__Mycobacteriales;f__Mycobacteriaceae;g__Mycolicibacterium;s__Mycolicibacterium_doricum
NCBI1267217	0	4	0	0	8	66	1	0	0	0	0	0	0	0	0	k__Bacteria;p__Pseudomonadota;c__Betaproteobacteria;o__Burkholderiales;f__Comamonadaceae;g__Melaminivora;s__Melaminivora_jejuensis
NCBI1267768	0	0	0	0	0	8	0	0	0	0	0	0	0	0	0	k__Bacteria;p__Pseudomonadota;c__Alphaproteobacteria;o__Rhodobacterales;f__Paracoccaceae;g__Brevirhabdus;s__Brevirhabdus_pacifica
NCBI1270	1	17	35	89	97	769	13	0	11	4	5	28	0	107	10	k__Bacteria;p__Actinomycetota;c__Actinomycetes;o__Micrococcales;f__Micrococcaceae;g__Micrococcus;s__Micrococcus_luteus
NCBI1272	0	0	0	0	0	16	0	2	0	0	0	0	0	0	0	k__Bacteria;p__Actinomycetota;c__Actinomycetes;o__Micrococcales;f__Micrococcaceae;g__Kocuria;s__Kocuria_varians
NCBI1274359	16	15	2	0	12	256	0	3	0	4	4	9	0	0	0	k__Bacteria;p__Pseudomonadota;c__Gammaproteobacteria;o__Pseudomonadales;f__Pseudomonadaceae;g__Pseudomonas;s__Pseudomonas_sihuiensis
NCBI1274631	0	0	0	0	0	1	1	0	0	0	0	0	0	0	0	k__Bacteria;p__Pseudomonadota;c__Alphaproteobacteria;o__Hyphomicrobiales;f__Nitrobacteraceae;g__Bradyrhizobium;s__Bradyrhizobium_icense
NCBI1275	0	1	7	9	17	50	5	1	2	2	1	2	0	0	0	k__Bacteria;p__Actinomycetota;c__Actinomycetes;o__Micrococcales;f__Micrococcaceae;g__Kocuria;s__Kocuria_rosea
NCBI1276	1	0	0	16	0	10	1	2	0	0	2	1	0	95	0	k__Bacteria;p__Actinomycetota;c__Actinomycetes;o__Micrococcales;f__Kytococcaceae;g__Kytococcus;s__Kytococcus_sedentarius

NCBI1279031	32	0	0	42	119	310	40	0	10	0	0	0	0	0	0	k__Bacteria;p__Pseudomonadota;c__Alphaproteobacteria;o__Hyphomicrobiales;f__Rhizobiaceae;g__Agrobacterium;s__Agrobacterium_sp._33MFTa1.1
NCBI1280	0	0	0	4	42	25	12	8	0	0	7	1304	0	0	0	k__Bacteria;p__Bacillota;c__Bacilli;o__Bacillales;f__Staphylococcaceae;g__Staphylococcus;s__Staphylococcus_aureus
NCBI1280052	0	0	0	0	0	52	0	0	0	0	0	0	0	0	0	k__Bacteria;p__Pseudomonadota;c__Gammaproteobacteria;o__Moraxellales;f__Moraxellaceae;g__Acinetobacter;s__Acinetobacter_sp._M131
NCBI1281	0	0	0	0	0	1	0	0	0	0	0	0	0	0	0	k__Bacteria;p__Bacillota;c__Bacilli;o__Bacillales;f__Staphylococcaceae;g__Staphylococcus;s__Staphylococcus_carnosus
NCBI1282	2	18	3	11	63	92	0	10	23	2	5	45	0	4	6	k__Bacteria;p__Bacillota;c__Bacilli;o__Bacillales;f__Staphylococcaceae;g__Staphylococcus;s__Staphylococcus_epidermidis
NCBI1283	0	1	0	1	0	2	2	2	0	1	0	0	0	0	0	k__Bacteria;p__Bacillota;c__Bacilli;o__Bacillales;f__Staphylococcaceae;g__Staphylococcus;s__Staphylococcus_haemolyticus
NCBI1285901	0	0	0	0	0	0	0	0	0	0	0	2	0	0	0	k__Bacteria;p__Actinomycetota;c__Actinomycetes;o__Propionibacteriales;f__Propionibacteriaceae;g__Tessaracoccus;s__Tessaracoccus_defluvii
NCBI1286180	0	0	0	0	0	1	0	0	0	0	0	0	0	0	0	k__Bacteria;p__Actinomycetota;c__Actinomycetes;o__Mycobacteriales;f__Mycobacteriaceae;g__Mycolicibacterium;s__Mycolicibacterium_sediminis
NCBI1286181	0	0	0	0	0	139	0	0	0	0	0	0	0	0	0	k__Bacteria;p__Actinomycetota;c__Actinomycetes;o__Mycobacteriales;f__Mycobacteriaceae;g__Mycolicibacterium;s__Mycolicibacterium_arabiense
NCBI128780	24	0	10	0	22	255	11	0	5	0	0	0	0	0	0	k__Bacteria;p__Pseudomonadota;c__Gammaproteobacteria;o__Xanthomonadales;f__Xanthomonadaceae;g__Stenotrophomonas;s__Stenotrophomonas_acidaminiphila

NCBI128785	72	61	45	11	173	2672	54	12	21	17	0	28	9	7	18	k__Bacteria;p__Pseudomonadota;c__Gammaproteobacteria;o__Xanthomonadales;f__Xanthomonadaceae;g__Pseudoxanthomonas;s__Pseudoxanthomonas_mexicana
NCBI1288	0	0	0	0	2	1	0	0	0	0	0	5	0	0	0	k__Bacteria;p__Bacillota;c__Bacilli;o__Bacillales;f__Staphylococcaceae;g__Staphylococcus;s__Staphylococcus_xylosus
NCBI1288121	0	0	0	0	0	0	13	0	0	0	0	1	0	0	0	k__Bacteria;p__Bacteroidota;c__Bacteroidia;o__Bacteroidales;f__Rikenellaceae;g__Alistipes;s__Alistipes_senegalensis
NCBI1288495	17	28	7	0	0	171	17	6	0	0	0	0	0	0	0	k__Bacteria;p__Pseudomonadota;c__Betaproteobacteria;o__Burkholderiales;f__Comamonadaceae;g__Diaphorobacter;s__Diaphorobacter_aerolatus
NCBI1288636	0	3	0	1	0	4	0	0	0	1	0	1	0	0	0	k__Bacteria;p__Actinomycetota;c__Actinomycetes;o__Micrococcales;f__Ornithinimicrobiaceae;g__Ornithinimicrobium;s__Ornithinimicrobium_flavum
NCBI1290	1	2	2	3	22	45	4	15	0	1	0	14	0	0	4	k__Bacteria;p__Bacillota;c__Bacilli;o__Bacillales;f__Staphylococcaceae;g__Staphylococcus;s__Staphylococcus_hominis
NCBI1292	2	0	0	0	6	2	0	1	0	0	1	1	6	0	1	k__Bacteria;p__Bacillota;c__Bacilli;o__Bacillales;f__Staphylococcaceae;g__Staphylococcus;s__Staphylococcus_warneri
NCBI129338	0	0	0	0	0	2	0	0	0	0	0	16	0	0	0	k__Bacteria;p__Bacillota;c__Bacilli;o__Bacillales;f__Bacillaceae;g__Geobacillus;s__Geobacillus_subterraneus
NCBI1294143	2	0	0	0	0	1	0	0	0	0	0	0	0	0	0	k__Bacteria;p__Pseudomonadota;c__Gammaproteobacteria;o__Pseudomonadales;f__Pseudomonadaceae;g__Pseudomonas;s__Pseudomonas_sp._ATCC_13867
NCBI1295392	0	0	0	0	0	0	0	0	0	0	0	1	0	0	0	k__Bacteria;p__Pseudomonadota;c__Gammaproteobacteria;o__Vibrionales;f__Vibrionaceae;g__Photobacterium;s__Photobacterium_gaetbulicola

NCBI1295642	0	0	0	0	0	0	0	0	0	0	0	2	0	0	0	k__Bacteria;p__Bacillota;c__Bacilli;o__Bacillales;f__Bacillaceae;g__Parageobacillus;s__Parageobacillus_genomosp._1
NCBI1296	0	1	0	0	0	1	1	0	0	0	0	0	0	0	0	k__Bacteria;p__Bacillota;c__Bacilli;o__Bacillales;f__Staphylococcaceae;g__Mammaliicoccus;s__Mammaliicoccus_sciuri
NCBI1296540	0	0	0	0	1	0	0	0	2	0	0	0	0	0	0	k__Bacteria;p__Bacillota;c__Bacilli;o__Lactobacillales;f__Lactobacillaceae;g__Paucilactobacillus;s__Paucilactobacillus_nenjiangensis
NCBI1296669	2432	3048	1491	92	4816	87818	2542	197	898	340	352	903	404	323	411	k__Bacteria;p__Pseudomonadota;c__Betaproteobacteria;o__Burkholderiales;f__;g__Aquabacterium;s__Aquabacterium_olei
NCBI129789	0	0	0	0	0	0	0	0	5	0	0	0	0	0	0	k__Archaea;p__Euryarchaeota;c__Halobacteria;o__Natrialbales;f__Natrialbaceae;g__Natrialba;s__Natrialba_aegyptia
NCBI129921	0	0	0	0	0	13	0	0	0	0	0	0	0	0	0	k__Bacteria;p__Actinomycetota;c__Actinomycetes;o__Pseudonocardiales;f__Pseudonocardiaceae;g__Amycolatopsis;s__Amycolatopsis_keratiniphila
NCBI1301032	0	0	0	0	0	7	0	0	0	0	0	0	0	0	0	k__Bacteria;p__Pseudomonadota;c__Alphaproteobacteria;o__Hyphomicrobiales;f__Rhizobiaceae;g__Rhizobium;s__Rhizobium_sp._IE4771
NCBI1302	0	2	0	2	4	1	0	0	0	0	0	8	2	0	0	k__Bacteria;p__Bacillota;c__Bacilli;o__Lactobacillales;f__Streptococcaceae;g__Streptococcus;s__Streptococcus_gordonii
NCBI1303	2	2	0	9	21	9	14	12	5	0	0	50	0	0	0	k__Bacteria;p__Bacillota;c__Bacilli;o__Lactobacillales;f__Streptococcaceae;g__Streptococcus;s__Streptococcus_oralis
NCBI1304	0	0	19	3	0	9	3	0	0	0	1	72	0	0	0	k__Bacteria;p__Bacillota;c__Bacilli;o__Lactobacillales;f__Streptococcaceae;g__Streptococcus;s__Streptococcus_salivarius

NCBI1305	0	7	0	2	0	11	0	2	0	0	0	15	0	0	0	k__Bacteria;p__Bacillota;c__Bacilli;o__Lactobacillales;f__Streptococcaceae;g__Streptococcus;s__Streptococcus_sanguinis
NCBI1306787	0	0	0	0	0	1	0	0	0	0	0	0	0	0	0	k__Bacteria;p__Pseudomonadota;c__Gammaproteobacteria;o__Oceanospirillales;f__Alcanivoracaceae;g__Isoalcanivorax;s__Isoalcanivorax_pacificus
NCBI1306993	0	0	0	0	18	0	0	0	0	0	0	0	0	0	0	k__Bacteria;p__Pseudomonadota;c__Gammaproteobacteria;o__Pseudomonadales;f__Pseudomonadaceae;g__Pseudomonas;s__Pseudomonas_soli
NCBI1307	0	0	0	1	0	16	0	0	0	0	0	1	0	0	0	k__Bacteria;p__Bacillota;c__Bacilli;o__Lactobacillales;f__Streptococcaceae;g__Streptococcus;s__Streptococcus_suis
NCBI1308	1	1	1	2	1	14	0	0	0	0	0	7	0	0	0	k__Bacteria;p__Bacillota;c__Bacilli;o__Lactobacillales;f__Streptococcaceae;g__Streptococcus;s__Streptococcus_thermophilus
NCBI1309	0	0	0	1	0	2	0	0	0	0	0	1	0	0	0	k__Bacteria;p__Bacillota;c__Bacilli;o__Lactobacillales;f__Streptococcaceae;g__Streptococcus;s__Streptococcus_mutans
NCBI1309411	0	0	0	0	0	9	0	0	0	0	0	0	0	0	0	k__Bacteria;p__Deinococcota;c__Deinococci;o__Deinococcales;f__Deinococcaceae;g__Deinococcus;s__Deinococcus_soli_(ex_Cha_et_al._2016)
NCBI1310	1	0	0	0	4	1	0	2	0	0	0	0	0	0	0	k__Bacteria;p__Bacillota;c__Bacilli;o__Lactobacillales;f__Streptococcaceae;g__Streptococcus;s__Streptococcus_sobrinus
NCBI1311	0	0	0	0	0	23	846	0	15	0	0	0	0	0	0	k__Bacteria;p__Bacillota;c__Bacilli;o__Lactobacillales;f__Streptococcaceae;g__Streptococcus;s__Streptococcus_agalactiae
NCBI131110	0	0	0	1	0	7	0	0	1	0	0	0	0	0	0	k__Bacteria;p__Actinomycetota;c__Actinomycetes;o__Actinomycetales;f__Actinomycetaceae;g__Schaalia;s__Schaalia_radingae

NCBI131111	0	3	0	0	0	0	0	0	2	0	0	1	0	0	0	k__Bacteria;p__Actinomycetota;c__Actinomycetes;o__Actinomycetales;f__Actinomycetaceae;g__Schaalia;s__Schaalia_turicensis
NCBI1313	0	0	0	0	0	0	0	0	0	0	0	4	0	0	0	k__Bacteria;p__Bacillota;c__Bacilli;o__Lactobacillales;f__Streptococcaceae;g__Streptococcus;s__Streptococcus_pneumoniae
NCBI1314	0	0	0	0	0	0	2	0	0	0	0	2	0	0	0	k__Bacteria;p__Bacillota;c__Bacilli;o__Lactobacillales;f__Streptococcaceae;g__Streptococcus;s__Streptococcus_pyogenes
NCBI131568	0	0	0	0	1	2	0	0	0	0	0	0	0	0	0	k__Bacteria;p__Actinomycetota;c__Actinomycetes;o__Kineosporiales;f__Kineosporiaceae;g__Kineococcus;s__Kineococcus_radiotolerans
NCBI1315974	3	3	3	3	15	202	6	0	0	0	3	0	0	3	0	k__Bacteria;p__Pseudomonadota;c__Alphaproteobacteria;o__Sphingomonadales;f__Sphingomonadaceae;g__Sphingobium;s__Sphingobium_sp._TKS
NCBI1316408	0	0	0	0	0	0	0	0	2	0	0	2	0	0	0	k__Bacteria;p__Bacillota;c__Bacilli;o__Lactobacillales;f__Streptococcaceae;g__Streptococcus;s__Streptococcus_sp._HSISM1
NCBI1316412	0	0	5	0	0	0	0	0	0	0	0	19	0	0	0	k__Bacteria;p__Bacillota;c__Bacilli;o__Lactobacillales;f__Streptococcaceae;g__Streptococcus;s__Streptococcus_sp._HSISS3
NCBI1318	0	0	0	0	0	0	12	0	0	0	0	0	0	0	0	k__Bacteria;p__Bacillota;c__Bacilli;o__Lactobacillales;f__Streptococcaceae;g__Streptococcus;s__Streptococcus_parasanguinis
NCBI132132	0	0	0	0	0	0	0	0	3	0	0	0	0	0	0	k__Bacteria;p__Thermodesulfobacteriota;c__Desulfovibrionia;o__Desulfovibrionales;f__Desulfomicrobiaceae;g__Desulfomicrobium;s__Desulfomicrobium_orale
NCBI1325090	0	0	0	3	2	83	2	0	0	2	2	0	9	5	21	k__Bacteria;p__Pseudomonadota;c__Alphaproteobacteria;o__Hyphomicrobiales;f__Nitrobacteraceae;g__Bradyrhizobium;s__Bradyrhizobium_guangdongense

NCBI1325095	0	0	0	0	4	7	0	0	0	0	0	0	0	0	0	k__Bacteria;p__Pseudomonadota;c__Alphaproteobacteria;o__Hyphomicrobiales;f__Nitrobacteraceae;g__Bradyrhizobium;s__Bradyrhizobium_guangzhouense
NCBI1325100	0	0	0	0	0	1	0	0	0	0	0	0	0	0	0	k__Bacteria;p__Pseudomonadota;c__Alphaproteobacteria;o__Hyphomicrobiales;f__Nitrobacteraceae;g__Bradyrhizobium;s__Bradyrhizobium_sp._CCBAU_51753
NCBI1325102	0	0	1	0	2	13	0	0	0	0	2	0	0	0	0	k__Bacteria;p__Pseudomonadota;c__Alphaproteobacteria;o__Hyphomicrobiales;f__Nitrobacteraceae;g__Bradyrhizobium;s__Bradyrhizobium_sp._CCBAU_51765
NCBI1325107	0	0	0	0	0	0	0	0	0	0	0	0	0	0	2	k__Bacteria;p__Pseudomonadota;c__Alphaproteobacteria;o__Hyphomicrobiales;f__Nitrobacteraceae;g__Bradyrhizobium;s__Bradyrhizobium_zhanjiangense
NCBI1325111	0	0	0	0	0	1	0	0	0	0	0	0	0	0	3	k__Bacteria;p__Pseudomonadota;c__Alphaproteobacteria;o__Hyphomicrobiales;f__Nitrobacteraceae;g__Bradyrhizobium;s__Bradyrhizobium_sp._CCBAU_53338
NCBI1325112	0	0	0	0	0	1	0	0	0	0	0	0	0	0	1	k__Bacteria;p__Pseudomonadota;c__Alphaproteobacteria;o__Hyphomicrobiales;f__Nitrobacteraceae;g__Bradyrhizobium;s__Bradyrhizobium_sp._CCBAU_53340
NCBI1325114	0	0	0	0	4	0	0	0	0	0	0	0	0	0	0	k__Bacteria;p__Pseudomonadota;c__Alphaproteobacteria;o__Hyphomicrobiales;f__Nitrobacteraceae;g__Bradyrhizobium;s__Bradyrhizobium_sp._CCBAU_53351
NCBI1325115	0	0	0	1	0	0	0	0	0	0	0	0	1	0	0	k__Bacteria;p__Pseudomonadota;c__Alphaproteobacteria;o__Hyphomicrobiales;f__Nitrobacteraceae;g__Bradyrhizobium;s__Bradyrhizobium_guangxiense
NCBI1325120	0	0	0	0	0	1	0	0	0	1	0	0	0	0	0	k__Bacteria;p__Pseudomonadota;c__Alphaproteobacteria;o__Hyphomicrobiales;f__Nitrobacteraceae;g__Bradyrhizobium;s__Bradyrhizobium_sp._CCBAU_53421
NCBI1325724	29	16	24	134	301	894	22	0	32	0	0	0	0	0	0	k__Bacteria;p__Pseudomonadota;c__Alphaproteobacteria;o__Caulobacterales;f__Caulobacteraceae;g__Brevundimonas;s__Brevundimonas_vancanneytii

NCBI1327635	0	0	1	0	0	1	0	0	0	0	0	0	0	0	0	k__Bacteria;p__Pseudomonadota;c__Alphaproteobacteria;o__Sphingomonadales;f__Sphingomonadaceae;g__Sphingomonas;s__Sphingomonas_psychrotolerans
NCBI1328	0	0	0	0	0	2	0	0	0	0	0	16	0	0	0	k__Bacteria;p__Bacillota;c__Bacilli;o__Lactobacillales;f__Streptococcaceae;g__Streptococcus;s__Streptococcus_anginosus
NCBI1329	0	0	0	0	0	0	1	0	0	0	0	0	0	0	0	k__Bacteria;p__Bacillota;c__Bacilli;o__Lactobacillales;f__Streptococcaceae;g__Streptococcus;s__Streptococcus_canis
NCBI1329522	0	0	0	0	1	0	0	0	1	0	0	0	0	0	0	k__Bacteria;p__;c__;o__;f__;g__;s__uncultured_bacterium_BAC10G6
NCBI1329523	0	0	0	0	2	0	0	0	2	0	0	0	0	0	0	k__Bacteria;p__;c__;o__;f__;g__;s__uncultured_bacterium_BAC25G1
NCBI1331682	0	0	0	0	27	0	0	0	0	0	0	0	0	0	0	k__Bacteria;p__Actinomycetota;c__Actinomycetes;o__Micrococcales;f__Dermabacteraceae;g__Brachybacterium;s__Brachybacterium_ginsengisoli
NCBI1332080	13	31	10	0	50	986	7	0	15	0	0	4	2	0	0	k__Bacteria;p__Pseudomonadota;c__Alphaproteobacteria;o__Sphingomonadales;f__Sphingomonadaceae;g__Sphingobium;s__Sphingobium_baderi
NCBI1333996	0	0	0	0	0	1	0	0	0	0	0	0	0	0	0	k__Bacteria;p__Pseudomonadota;c__Alphaproteobacteria;o__Hyphomicrobiales;f__Nitrobacteraceae;g__Variibacter;s__Variibacter_gotjawalensis
NCBI1334	0	0	0	0	0	0	19	0	0	0	0	0	0	0	0	k__Bacteria;p__Bacillota;c__Bacilli;o__Lactobacillales;f__Streptococcaceae;g__Streptococcus;s__Streptococcus_dysgalactiae
NCBI1335	0	0	0	0	0	5	27	1	0	0	0	0	0	0	0	k__Bacteria;p__Bacillota;c__Bacilli;o__Lactobacillales;f__Streptococcaceae;g__Streptococcus;s__Streptococcus_equinus
NCBI1335043	1	1	0	0	0	14	0	0	0	0	0	0	0	0	0	k__Bacteria;p__Pseudomonadota;c__Alphaproteobacteria;o__Hyphomicrobiales;f__Devosiaceae;g__Paradevosia;

															s__Paradevosia_shaoguanensis
NCBI1335048	0	0	0	0	2	3	0	0	0	0	0	0	0	0	0	k__Bacteria;p__Pseudomonadota;c__Alphaproteobacteria;o__Rhodobacterales;f__Paracoccaceae;g__Frigidibacter;s__Frigidibacter_mobilis
NCBI1335060	1	1	1	1	9	50	0	0	2	0	0	0	0	0	1	k__Bacteria;p__Pseudomonadota;c__Alphaproteobacteria;o__Hyphomicrobiales;f__Rhizobiaceae;g__Rhizobium;s__Rhizobium_sp._Q54
NCBI1335061	34	36	20	7	141	1511	50	10	8	14	0	63	0	8	24	k__Bacteria;p__Pseudomonadota;c__Alphaproteobacteria;o__Hyphomicrobiales;f__Rhizobiaceae;g__Pseudorhizobium;s__Pseudorhizobium_flavum
NCBI1335613	0	0	3	0	0	6	0	0	0	0	0	0	0	0	0	k__Bacteria;p__Actinomycetota;c__Coriobacteriia;o__Eggerthellales;f__Eggerthellaceae;g__Gordonibacter;s__Gordonibacter_urolithinfaciens
NCBI1337838	0	0	0	0	6	289	0	0	0	0	0	0	0	0	0	k__Bacteria;p__Pseudomonadota;c__Betaproteobacteria;o__Burkholderiales;f__Oxalobacteraceae;g__Massilia;s__Massilia_sp._UMI-21
NCBI133926	0	0	0	1	0	0	0	0	0	0	0	0	0	0	0	k__Bacteria;p__Actinomycetota;c__Coriobacteriia;o__Coriobacteriales;f__Atopobiaceae;g__Olsenella;s__Olsenella_uli
NCBI1340425	0	0	0	0	0	1	0	0	0	0	0	0	0	0	0	k__Bacteria;p__Thermodesulfobacteriota;c__Desulfuromonadia;o__Geobacterales;f__Geobacteraceae;g__Geobacter;s__Geobacter_anodireducens
NCBI1341132	0	0	0	0	1	1	0	0	0	0	0	0	0	0	0	k__Fungi;p__Ascomycota;c__Eurotiomycetes;o__Eurotiales;f__Aspergillaceae;g__Aspergillus;s__Aspergillus_welwitschiae
NCBI1343	0	0	0	0	0	0	0	0	0	0	0	2	0	0	0	k__Bacteria;p__Bacillota;c__Bacilli;o__Lactobacillales;f__Streptococcaceae;g__Streptococcus;s__Streptococcus_vestibularis

NCBI134375	0	0	0	0	0	157	0	0	0	0	0	0	0	0	0	k__Bacteria;p__Pseudomonadota;c__Betaproteobacteria;o__Burkholderiales;f__Alcaligenaceae;g__Achromobacter;s__Achromobacter_sp.
NCBI134534	3	5	0	0	6	26	2	1	0	0	0	1	0	1	0	k__Bacteria;p__Pseudomonadota;c__Gammaproteobacteria;o__Moraxellales;f__Moraxellaceae;g__Acinetobacter;s__Acinetobacter_gyllenbergii
NCBI134537	1	3	3	1	12	5	2	1	1	0	1	5	0	0	1	k__Bacteria;p__Pseudomonadota;c__Betaproteobacteria;o__Burkholderiales;f__Burkholderiaceae;g__Paraburkholderia;s__Paraburkholderia_fungorum
NCBI1348774	0	0	0	0	0	36	0	0	0	0	0	0	0	0	0	k__Bacteria;p__Pseudomonadota;c__Alphaproteobacteria;o__Sphingomonadales;f__Erythrobacteraceae;g__Croceicoccus;s__Croceicoccus_naphthovorans
NCBI1349819	0	0	0	0	0	4	0	0	0	0	0	0	0	0	0	k__Bacteria;p__Pseudomonadota;c__Alphaproteobacteria;o__Hyphomicrobiales;f__Aurantimonadaceae;g__Aureimonas;s__Aureimonas_sp._AU20
NCBI1351	0	0	8	3	0	57	9	0	0	0	0	11	0	0	1	k__Bacteria;p__Bacillota;c__Bacilli;o__Lactobacillales;f__Enterococcaceae;g__Enterococcus;s__Enterococcus_faecalis
NCBI1352	0	0	7	0	4	18	5	1	1	0	8	55	0	0	0	k__Bacteria;p__Bacillota;c__Bacilli;o__Lactobacillales;f__Enterococcaceae;g__Enterococcus;s__Enterococcus_faecium
NCBI1353	0	0	17	13	22	24	4	6	8	0	0	0	0	0	0	k__Bacteria;p__Bacillota;c__Bacilli;o__Lactobacillales;f__Enterococcaceae;g__Enterococcus;s__Enterococcus_gallinarum
NCBI1353260	0	0	0	0	0	0	0	0	0	0	1	0	0	0	0	k__Archaea;p__Nitrososphaerota;c__Nitrososphaeria;o__Nitrososphaerales;f__Nitrososphaeraceae;g__Candidatus_Nitrosocosmicus;s__Candidatus_Nitrosocosmicus_oleophilus
NCBI1353889	0	0	0	0	4	3	0	0	0	0	0	0	0	0	0	k__Bacteria;p__Pseudomonadota;c__Betaproteobacteria;o__Burkholderiales;f__Alcaligenaceae;g__Achromobact

																er;s__Achromobacter_pestifer
NCBI1353891	0	0	4	1	14	2	2	0	0	0	0	0	0	0	0	k__Bacteria;p__Pseudomonadota;c__Betaproteobacteria;o__Burkholderiales;f__Alcaligenaceae;g__Achromobacter;s__Achromobacter_deleyi
NCBI1354	0	0	1	0	0	3	1	0	0	0	0	1	0	0	0	k__Bacteria;p__Bacillota;c__Bacilli;o__Lactobacillales;f__Enterococcaceae;g__Enterococcus;s__Enterococcus_hirae
NCBI1355477	0	0	5	0	2	13	0	0	0	0	0	2	0	0	1	k__Bacteria;p__Pseudomonadota;c__Alphaproteobacteria;o__Hyphomicrobiales;f__Nitrobacteraceae;g__Bradyrhizobium;s__Bradyrhizobium_diazoefficiens
NCBI135577	0	0	0	0	0	53	0	0	0	0	0	0	9	0	0	k__Bacteria;p__Pseudomonadota;c__Gammaproteobacteria;o__Alteromonadales;f__Idiomarinaceae;g__Idiomarina;s__Idiomarina_loihiensis
NCBI1356852	1	0	0	0	2	25	0	0	0	0	0	0	1	0	0	k__Bacteria;p__Bacteroidota;c__Cytophagia;o__Cytophagales;f__Hymenobacteraceae;g__Hymenobacter;s__Hymenobacter_sp._APR13
NCBI135719	57	78	15	13	150	1628	58	6	23	20	2	2	0	7	4	k__Bacteria;p__Pseudomonadota;c__Alphaproteobacteria;o__Sphingomonadales;f__Sphingomonadaceae;g__Sphingobium;s__Sphingobium_amiense
NCBI135740	0	0	0	0	0	23	0	0	0	0	0	0	0	0	0	k__Bacteria;p__Pseudomonadota;c__Alphaproteobacteria;o__Rhodobacterales;f__Paracoccaceae;g__Paracoccus;s__Paracoccus_kondratievae
NCBI1357915	0	0	0	0	5	0	0	0	0	0	0	0	0	0	0	k__Bacteria;p__Actinomycetota;c__Actinomycetes;o__Micrococcales;f__Micrococcaceae;g__Arthrobacter;s__Arthrobacter_sp._QXT-31
NCBI1357916	0	0	0	0	0	4	0	0	0	0	0	0	0	0	0	k__Bacteria;p__Pseudomonadota;c__Alphaproteobacteria;o__Sphingomonadales;f__Sphingomonadaceae;g__Sphingopyxis;s__Sphingopyxis_sp._QXT-31
NCBI1	102	120	102	86	117	136	89	52	59	151	111	179	132	124	71	k__Bacteria;p__Bacillota;c__Bacilli;o__Lactobacillales;f

358																__Streptococcaceae;g__Lactococcus;s__Lactococcus_lactis
NCBI1359	0	0	0	0	0	0	0	0	0	0	1	1	0	0	0	k__Bacteria;p__Bacillota;c__Bacilli;o__Lactobacillales;f__Streptococcaceae;g__Lactococcus;s__Lactococcus_cremoris
NCBI136098	0	15	0	30	0	395	0	32	0	0	0	0	0	0	0	k__Bacteria;p__Pseudomonadota;c__Alphaproteobacteria;o__Hyphomicrobiales;f__Rhizobiaceae;g__Rhizobium;s__Rhizobium_sp._TCK
NCBI1363	0	0	9	4	54	11	2	0	3	0	0	2	2	0	2	k__Bacteria;p__Bacillota;c__Bacilli;o__Lactobacillales;f__Streptococcaceae;g__Lactococcus;s__Lactococcus_garvieae
NCBI1364	0	0	0	0	0	0	0	0	0	0	0	0	0	0	3	k__Bacteria;p__Bacillota;c__Bacilli;o__Lactobacillales;f__Streptococcaceae;g__Lactococcus;s__Lactococcus_piscium
NCBI1365886	0	0	0	0	0	0	0	0	1	0	0	0	0	0	0	k__Fungi;p__Ascomycota;c__Saccharomycetes;o__Saccharomycetales;f__Saccharomycetaceae;g__Zygosaccharomyces;s__Zygosaccharomyces_parabailii
NCBI1366	0	0	0	0	0	1	0	0	0	0	0	0	0	0	0	k__Bacteria;p__Bacillota;c__Bacilli;o__Lactobacillales;f__Streptococcaceae;g__Lactococcus;s__Lactococcus_raffinolactis
NCBI1368430	102	110	51	4	210	3752	113	3	54	16	23	46	13	15	14	k__Bacteria;p__Pseudomonadota;c__Alphaproteobacteria;o__Hyphomicrobiales;f__Rhizobiaceae;g__Rhizobium;s__Rhizobium_rosettiformans
NCBI136857	0	0	0	0	0	2	0	0	0	0	0	0	0	0	0	k__Bacteria;p__Actinomycetota;c__Actinomycetes;o__Mycobacteriales;f__Corynebacteriaceae;g__Corynebacterium;s__Corynebacterium_testudinoris
NCBI13689	1	2	3	4	17	853	5	0	3	0	0	1212	0	0	1	k__Bacteria;p__Pseudomonadota;c__Alphaproteobacteria;o__Sphingomonadales;f__Sphingomonadaceae;g__Sphingomonas;s__Sphingomonas_paucimobilis
NCBI1	118	143	624	152	304	574	147	119	566	214	149	477	159	97	192	k__Bacteria;p__Pseudomonadota;c__Alphaproteobacteri

3690	7	0			1	94	3									a;o__Sphingomonadales;f__Sphingomonadaceae;g__Sphingobium;s__Sphingobium_yanoikuyae
NCBI137545	0	0	0	0	0	0	1	0	0	0	0	0	0	0	0	k__Bacteria;p__Pseudomonadota;c__Gammaproteobacteria;o__Enterobacterales;f__Yersiniaceae;g__Serratia;s__Serratia_quinivorans
NCBI137591	0	0	0	0	0	1	0	0	0	0	0	2	0	0	0	k__Bacteria;p__Bacillota;c__Bacilli;o__Lactobacillales;f__Lactobacillaceae;g__Weissella;s__Weissella_cibaria
NCBI1377	0	0	239	0	2	0	0	0	0	0	0	0	0	0	0	k__Bacteria;p__Bacillota;c__Bacilli;o__Lactobacillales;f__Aerococcaceae;g__Aerococcus;s__Aerococcus_viridans
NCBI137732	0	0	0	0	0	1	0	0	0	0	0	0	0	0	0	k__Bacteria;p__Bacillota;c__Bacilli;o__Lactobacillales;f__Carnobacteriaceae;g__Granulicatella;s__Granulicatella_elegans
NCBI1379	0	0	0	0	0	0	0	0	0	0	0	9	0	0	0	k__Bacteria;p__Bacillota;c__Bacilli;o__Bacillales;f__;g__Gemella;s__Gemella_haemolysans
NCBI138118	0	0	0	0	1	5	0	0	0	0	0	0	0	0	0	k__Bacteria;p__Pseudomonadota;c__Betaproteobacteria;o__Rhodocyclales;f__Azonexaceae;g__Quatrionicoccus;s__Quatrionicoccus_australiensis
NCBI1381597	0	0	0	5	0	27	3	0	0	0	0	0	0	0	5	k__Bacteria;p__Pseudomonadota;c__Alphaproteobacteria;o__Sphingomonadales;f__Sphingomonadaceae;g__Sphingomonas;s__Sphingomonas_sp._ERG5
NCBI1382	0	0	0	0	0	0	0	3	0	0	0	1	0	0	0	k__Bacteria;p__Actinomycetota;c__Coriobacteriia;o__Coriobacteriales;f__Atopobiaceae;g__Lancefieldella;s__Lancefieldella_parvula
NCBI138278	0	1	0	0	0	0	2	0	2	0	0	1	0	0	0	k__Fungi;p__Ascomycota;c__Eurotiomycetes;o__Eurotiales;f__Aspergillaceae;g__Aspergillus;s__Aspergillus_ochraceoroseus
NCBI1383262	12305	12264	10859	49738	101243	172634	15692	3702	8706	1822	2268	3062	2336	2193	2195	k__Fungi;p__Chytridiomycota;c__Chytridiomycetes;o__Synchytriales;f__Synchytriaceae;g__Synchytrium;s__Synchytrium_taraxaci

NCBI138336	0	0	0	0	6	35	0	0	0	1	0	3	0	0	0	k__Bacteria;p__Actinomycetota;c__Actinomycetes;o__Geodermatophilales;f__Geodermatophilaceae;g__Blastococcus;s__Blastococcus_saxobsidens
NCBI1387353	0	0	0	0	0	6	0	0	0	0	0	0	0	0	0	k__Bacteria;p__Planctomycetota;c__Planctomycetia;o__Isosphaerales;f__Isosphaeraceae;g__Paludisphaera;s__Paludisphaera_borealis
NCBI1389011	0	0	0	0	5	10	0	0	0	0	0	0	0	0	0	k__Bacteria;p__Pseudomonadota;c__Alphaproteobacteria;o__Rhodobacterales;f__Roseobacteraceae;g__Sulfitobacter;s__Sulfitobacter_sp._SK025
NCBI1389192	0	0	0	0	0	0	0	0	0	0	0	0	0	5	0	k__Bacteria;p__Pseudomonadota;c__Betaproteobacteria;o__Burkholderiales;f__Burkholderiaceae;g__Cupriavidus;s__Cupriavidus_sp._USMAHM13
NCBI1389713	6	0	0	4	1	7	0	1	0	0	0	1	0	0	0	k__Bacteria;p__Actinomycetota;c__Actinomycetes;o__Mycobacteriales;f__Mycobacteriaceae;g__Mycobacterium;s__Mycobacterium_paragordonae
NCBI1390	421	363	231	1472	3295	2107	372	77	241	19	33	72	56	23	39	k__Bacteria;p__Bacillota;c__Bacilli;o__Bacillales;f__Bacillaceae;g__Bacillus;s__Bacillus_amyloliquefaciens
NCBI139021	0	1	4	0	0	4	0	0	0	0	0	0	0	0	0	k__Bacteria;p__Actinomycetota;c__Actinomycetes;o__Mycobacteriales;f__Dietziaceae;g__Dietzia;s__Dietzia_psychralcaliphila
NCBI1390395	0	0	0	0	5	11	1	0	0	0	0	0	0	0	0	k__Bacteria;p__Pseudomonadota;c__Alphaproteobacteria;o__Sphingomonadales;f__Sphingomonadaceae;g__Sphingomonas;s__Sphingomonas_sp._LK11
NCBI1391653	0	0	0	0	0	1	0	0	0	0	0	0	0	0	0	k__Bacteria;p__Myxococcota;c__Myxococcia;o__Myxococcales;f__Vulgatibacteraceae;g__Vulgatibacter;s__Vulgatibacter_incomptus
NCBI1393	0	0	0	0	0	1	0	0	0	1	0	0	0	0	0	k__Bacteria;p__Bacillota;c__Bacilli;o__Bacillales;f__Paenibacillaceae;g__Brevibacillus;s__Brevibacillus_brevis
NCBI1393545	0	0	0	0	0	1	0	0	0	0	0	0	0	0	0	k__Bacteria;p__;c__;o__;f__;g__;s__uncultured_bacterium_Contig26

NCBI1393569	0	0	0	0	0	1	0	0	0	0	0	0	0	0	0	k__Bacteria;p__;c__;o__;f__;g__;s__uncultured_bacterium_Contig4
NCBI1394	0	0	0	0	0	0	0	0	0	0	0	54	0	0	0	k__Bacteria;p__Bacillota;c__Bacilli;o__Bacillales;f__Bacillaceae;g__Geobacillus;s__[Bacillus]_caldolyticus
NCBI139438	0	0	0	1	0	0	1	0	0	0	0	3	1	0	0	k__Bacteria;p__Deferribacterota;c__Deferribacteres;o__Deferribacterales;f__Geovibrionaceae;g__Geovibrio;s__Geovibrio_thiophilus
NCBI1396	1929	2189	1779	4800	3785	4121	1067	595	1068	804	481	990	259	272	352	k__Bacteria;p__Bacillota;c__Bacilli;o__Bacillales;f__Bacillaceae;g__Bacillus;s__Bacillus_cereus
NCBI1397	0	0	0	0	0	1	0	0	0	0	0	0	0	0	0	k__Bacteria;p__Bacillota;c__Bacilli;o__Bacillales;f__Bacillaceae;g__Niallia;s__Niallia_circulans
NCBI1398	0	0	0	0	0	7	1	0	0	0	0	0	0	0	0	k__Bacteria;p__Bacillota;c__Bacilli;o__Bacillales;f__Bacillaceae;g__Weizmannia;s__Weizmannia_coagulans
NCBI1399115	0	0	1	0	8	1	0	0	0	0	0	0	0	0	0	k__Bacteria;p__Bacillota;c__Bacilli;o__Bacillales;f__;g__Exiguobacterium;s__Exiguobacterium_sp._MH3
NCBI1402	0	0	0	0	70	14	0	0	0	0	0	0	0	0	0	k__Bacteria;p__Bacillota;c__Bacilli;o__Bacillales;f__Bacillaceae;g__Bacillus;s__Bacillus_licheniformis
NCBI1402861	0	0	0	1	0	0	0	0	0	0	0	0	0	0	0	k__Bacteria;p__Bacillota;c__Bacilli;o__Bacillales;f__Bacillaceae;g__Priestia;s__Priestia_filamentosa
NCBI1404	0	0	0	10	1	9	0	0	0	0	0	0	0	0	0	k__Bacteria;p__Bacillota;c__Bacilli;o__Bacillales;f__Bacillaceae;g__Priestia;s__Priestia_megaterium
NCBI1404367	0	0	0	0	0	3	0	0	0	0	0	0	0	0	0	k__Bacteria;p__Pseudomonadota;c__Alphaproteobacteria;o__Hyphomicrobiales;f__Nitrobacteraceae;g__Bradyrhizobium;s__Bradyrhizobium_symbiodeficiens
NCBI1404768	0	0	0	0	0	2	0	0	0	0	0	0	0	0	0	k__Bacteria;p__Pseudomonadota;c__Alphaproteobacteria;o__Hyphomicrobiales;f__Nitrobacteraceae;g__Bradyrhizobium;s__Bradyrhizobium_amphicarpaeae
NCBI1404864	0	0	0	0	4	6	0	0	0	0	0	1	0	0	0	k__Bacteria;p__Pseudomonadota;c__Alphaproteobacteria;o__Hyphomicrobiales;f__Nitrobacteraceae;g__Bradyrhizobium;s__Bradyrhizobium_cosmicum

NCBI1405	4	2	2	10	26	23	9	4	6	2	0	6	3	0	0	k__Bacteria;p__Bacillota;c__Bacilli;o__Bacillales;f__Bacillaceae;g__Bacillus;s__Bacillus_mycoides
NCBI1406	1	0	2	3	0	20	1	0	0	0	2	3	0	0	1	k__Bacteria;p__Bacillota;c__Bacilli;o__Bacillales;f__Paenibacillaceae;g__Paenibacillus;s__Paenibacillus_polymyxa
NCBI1408	0	0	0	0	0	0	0	0	0	0	0	6	0	0	0	k__Bacteria;p__Bacillota;c__Bacilli;o__Bacillales;f__Bacillaceae;g__Bacillus;s__Bacillus_pumilus
NCBI1411902	0	0	0	0	0	0	0	0	0	0	0	0	0	0	8	k__Bacteria;p__Pseudomonadota;c__Alphaproteobacteria;o__Rhodobacterales;f__Paracoccaceae;g__Pacificitalea;s__Pacificitalea_manganoxidans
NCBI1419814	0	0	0	0	4	3	0	0	0	0	0	0	0	0	0	k__Bacteria;p__Bacillota;c__Bacilli;o__Lactobacillales;f__Streptococcaceae;g__Streptococcus;s__Streptococcus_sp._VT_162
NCBI1420916	0	0	0	0	0	0	0	0	0	0	2	2	0	0	0	k__Bacteria;p__Pseudomonadota;c__Gammaproteobacteria;o__Pseudomonadales;f__Marinobacteraceae;g__Marinobacter;s__Marinobacter_similis
NCBI1421	0	0	0	5	0	0	0	0	1	0	0	0	0	0	0	k__Bacteria;p__Bacillota;c__Bacilli;o__Bacillales;f__Bacillaceae;g__Lysinibacillus;s__Lysinibacillus_sphaericus
NCBI1422	0	0	0	0	0	4	0	0	0	0	0	92	0	0	0	k__Bacteria;p__Bacillota;c__Bacilli;o__Bacillales;f__Bacillaceae;g__Geobacillus;s__Geobacillus_stearothermophilus
NCBI1423	0	0	16	0	6	15	14	0	0	0	0	14	0	0	0	k__Bacteria;p__Bacillota;c__Bacilli;o__Bacillales;f__Bacillaceae;g__Bacillus;s__Bacillus_subtilis
NCBI1424339	0	0	0	6	0	0	0	0	0	0	0	0	0	0	0	k__Fungi;p__Ascomycota;c__Saccharomycetes;o__Saccharomycetales;f__Pichiaceae;g__Pichia;s__Pichia_sp._PS-4
NCBI1424810	0	0	0	0	0	1	0	0	0	0	0	0	0	0	0	k__Bacteria;p__;c__;o__;f__;g__;s__uncultured_bacterium_ctg7180000000757
NCBI1426	0	0	0	0	0	0	0	0	0	0	0	11	0	0	0	k__Bacteria;p__Bacillota;c__Bacilli;o__Bacillales;f__Bacillaceae;g__Parageobacillus;s__Parageobacillus_thermo

																glucosidasius
NCBI1428	1713	1916	1749	5200	6746	9037	1680	1017	1240	563	661	666	666	566	589	k__Bacteria;p__Bacillota;c__Bacilli;o__Bacillales;f__Bacillaceae;g__Bacillus;s__Bacillus_thuringiensis
NCBI142864	0	5	0	0	0	0	0	0	0	0	0	0	0	0	0	k__Bacteria;p__Cyanobacteriota;c__Cyanophyceae;o__Nostocales;f__Nostocaceae;g__Cylindrospermum;s__Cylindrospermum_stagnale
NCBI1428684	0	1	0	0	0	2	0	0	0	0	0	0	0	0	0	k__Bacteria;p__Bacillota;c__Bacilli;o__Bacillales;f__;g__Exiguobacterium;s__Exiguobacterium_alkaliphilum
NCBI1430884	0	0	0	0	0	194	0	0	0	0	0	0	0	0	0	k__Bacteria;p__Pseudomonadota;c__Betaproteobacteria;o__Burkholderiales;f__Sphaerotilaceae;g__Methylibium;s__Methylibium_sp._T29
NCBI143232	0	0	1	0	0	0	0	0	0	0	0	0	0	0	0	k__Fungi;p__Basidiomycota;c__Tremellomycetes;o__Trichosporonales;f__Trichosporonaceae;g__Vanrija;s__Vanrija_pseudolonga
NCBI1433513	0	0	0	1	0	0	0	0	0	0	0	0	0	0	0	k__Bacteria;p__Bacillota;c__Bacilli;o__Lactobacillales;f__Streptococcaceae;g__Streptococcus;s__Streptococcus_gwangjuense
NCBI143393	0	0	0	0	0	0	0	0	0	0	0	10	0	0	0	k__Bacteria;p__Bacillota;c__Clostridia;o__Eubacteriales;f__Eubacteriales_Family_XIII._Incertae_Sedis;g__;s__[Eubacterium]_sulci
NCBI1435607	5	2	4	3	31	190	2	0	1	0	0	18	0	0	0	k__Bacteria;p__Pseudomonadota;c__Alphaproteobacteria;o__Hyphomicrobiales;f__Rhizobiaceae;g__Rhizobium;s__Rhizobium_sp._Khangiran2
NCBI1437009	0	0	0	0	2	0	0	0	0	0	0	0	0	0	0	k__Bacteria;p__Pseudomonadota;c__Alphaproteobacteria;o__Hyphomicrobiales;f__Methylocystaceae;g__Chenggangzhangella;s__Chenggangzhangella_methanolivorans
NCBI1437360	0	0	0	0	3	1	0	0	0	1	0	0	0	0	0	k__Bacteria;p__Pseudomonadota;c__Alphaproteobacteria;o__Hyphomicrobiales;f__Nitrobacteraceae;g__Bradyrhizobium;s__Bradyrhizobium_erythrophlei

NCBI1437443	0	0	0	0	0	741	0	0	0	0	0	0	0	0	0	k__Bacteria;p__Pseudomonadota;c__Betaproteobacteria;o__Burkholderiales;f__Sphaerotilaceae;g__Methylibium;s__Methylibium_sp._T29-B
NCBI1437774	0	0	0	7	0	0	0	0	0	0	0	0	0	0	0	k__Bacteria;p__Bacillota;c__Bacilli;o__Bacillales;f__Staphylococcaceae;g__Salinicoccus;s__Salinicoccus_sp._BAB_3246
NCBI144185	0	0	0	0	0	0	0	0	0	0	0	0	0	0	258	k__Bacteria;p__Actinomycetota;c__Actinomycetes;o__Micrococcales;f__Microbacteriaceae;g__Leifsonia;s__Leifsonia_aquatica
NCBI1446467	1	0	0	0	0	42	0	0	1	0	0	0	0	0	0	k__Bacteria;p__Bacteroidota;c__Cytophagia;o__Cytophagales;f__Hymenobacteraceae;g__Hymenobacter;s__Hymenobacter_swuensis
NCBI1452	0	0	0	0	0	0	0	0	0	0	0	3	0	0	0	k__Bacteria;p__Bacillota;c__Bacilli;o__Bacillales;f__Bacillaceae;g__Bacillus;s__Bacillus_atrophaeus
NCBI1458425	0	0	1	0	0	1	0	0	0	0	0	0	0	0	0	k__Bacteria;p__Pseudomonadota;c__Betaproteobacteria;o__Burkholderiales;f__Comamonadaceae;g__Serpentinimonas;s__Serpentinimonas_raichei
NCBI1458426	0	1	0	0	2	4	0	0	0	0	0	0	0	0	0	k__Bacteria;p__Pseudomonadota;c__Betaproteobacteria;o__Burkholderiales;f__Comamonadaceae;g__Serpentinimonas;s__Serpentinimonas_maccroryi
NCBI1458492	0	0	0	0	0	0	0	0	0	0	0	16	0	0	0	k__Bacteria;p__Bacteroidota;c__Flavobacteriia;o__Flavobacteriales;f__Flavobacteriaceae;g__Myroides;s__Myroides_sp._ZB35
NCBI1462	0	0	0	0	0	12	0	0	0	0	0	776	0	0	0	k__Bacteria;p__Bacillota;c__Bacilli;o__Bacillales;f__Bacillaceae;g__Geobacillus;s__Geobacillus_kaustophilus
NCBI1463165	9	0	10	0	17	52	0	0	0	0	29	17	11	0	0	k__Bacteria;p__Pseudomonadota;c__Gammaproteobacteria;o__Enterobacterales;f__Enterobacteriaceae;g__Klebsiella;s__Klebsiella_quasipneumoniae
NCBI1463999	14	20	13	34	32	21	18	14	23	39	38	59	375	360	473	k__Fungi;p__Ascomycota;c__Leotiomycetes;o__Helotiales;f__Sclerotiniaceae;g__Botrytis;s__Botrytis_sinoallii

NCBI146474	1252	1223	1279	4231	3987	5374	18646	12842	18565	90940	85749	120875	224440	238016	258262	k__Bacteria;p__Pseudomonadota;c__Alphaproteobacteria;o__Rhodospirillales;f__Acetobacteraceae;g__Acetobacter;s__Acetobacter_orientalis
NCBI146476	89	132	101	441	688	835	353	189	271	1273	1165	1778	1274	1277	1212	k__Bacteria;p__Pseudomonadota;c__Alphaproteobacteria;o__Rhodospirillales;f__Acetobacteraceae;g__Acetobacter;s__Acetobacter_syzygii
NCBI146827	0	0	0	0	2	4	0	0	0	0	0	0	0	0	0	k__Bacteria;p__Actinomycetota;c__Actinomycetes;o__Mycobacteriales;f__Corynebacteriaceae;g__Corynebacterium;s__Corynebacterium_simulans
NCBI146939	69	34	28	0	138	991	69	5	21	9	14	27	10	10	18	k__Bacteria;p__Pseudomonadota;c__Betaproteobacteria;o__Rhodocyclales;f__Rhodocyclaceae;g__Azospira;s__Azospira_oryzae
NCBI1470621	0	0	0	0	0	0	0	0	13	0	0	0	0	0	0	k__Fungi;p__Ascomycota;c__Saccharomycetes;o__Saccharomycetales;f__Saccharomycetaceae;g__Saccharomyces;s__Saccharomyces_cerevisiae_x_Saccharomyces_eubayanus_x_Saccharomyces_uvarum
NCBI1471275	0	0	1	0	0	15	1	0	0	0	0	1	0	0	0	k__Bacteria;p__Pseudomonadota;c__Gammaproteobacteria;o__Pseudomonadales;f__Pseudomonadaceae;g__Pseudomonas;s__Pseudomonas_sp._A20
NCBI1471761	0	0	0	0	0	1	0	0	0	0	1	0	0	0	0	k__Bacteria;p__Bacillota;c__Bacilli;o__Bacillales;f__Thermoactinomycetaceae;g__Novibacillus;s__Novibacillus_thermophilus
NCBI147206	0	0	0	1	0	1	0	0	0	0	0	0	0	0	0	k__Bacteria;p__Actinomycetota;c__Coriobacteriia;o__Coriobacteriales;f__Coriobacteriaceae;g__Collinsella;s__Collinsella_stercoris
NCBI147645	5	3	0	0	26	115	6	0	0	0	0	9	6	0	0	k__Bacteria;p__Pseudomonadota;c__Alphaproteobacteria;o__Rhodobacterales;f__Paracoccaceae;g__Paracoccus;s__Paracoccus_yeei
NCBI147709	0	0	0	0	0	0	2	0	0	0	0	0	0	0	0	k__Bacteria;p__Bacillota;c__Bacilli;o__Lactobacillales;f__Carnobacteriaceae;g__Carnobacterium;s__Carnobacter

															ium_inhibens
NCBI147802	0	0	0	0	3	2	1	0	0	0	0	1	0	0	0	k__Bacteria;p__Bacillota;c__Bacilli;o__Lactobacillales;f__Lactobacillaceae;g__Lactobacillus;s__Lactobacillus_iners
NCBI1479	0	0	0	0	0	0	0	0	0	0	0	1	0	0	0	k__Bacteria;p__Bacillota;c__Bacilli;o__Bacillales;f__Bacillaceae;g__Bacillus;s__Bacillus_smithii
NCBI1479019	0	0	0	0	21	19	0	0	0	0	0	0	0	0	0	k__Bacteria;p__Pseudomonadota;c__Alphaproteobacteria;o__Hyphomicrobiales;f__Methylobacteriaceae;g__Methylobacterium;s__Methylobacterium_sp._C1
NCBI1479714	0	0	0	0	0	0	0	0	0	1	0	0	0	0	0	k__Bacteria;p__Pseudomonadota;c__Alphaproteobacteria;o__Rhodospirillales;f__Acetobacteraceae;g__Gluconobacter;s__Gluconobacter_sp._R-50361
NCBI1482074	0	0	0	0	0	1	0	0	0	0	0	0	0	0	0	k__Bacteria;p__Pseudomonadota;c__Alphaproteobacteria;o__Hyphomicrobiales;f__Pleomorphomonadaceae;g__Hartmannibacter;s__Hartmannibacter_diazotrophicus
NCBI148305	0	0	0	0	0	4	0	0	0	0	0	0	0	0	0	k__Fungi;p__Ascomycota;c__Sordariomycetes;o__Magnaporthales;f__Pyriculariaceae;g__Pyricularia;s__Pyricularia_grisea
NCBI1484109	0	0	0	0	0	0	0	0	0	0	0	0	0	1	0	k__Bacteria;p__Pseudomonadota;c__Alphaproteobacteria;o__Rhodospirillales;f__Acetobacteraceae;g__Lichenicola;s__Lichenicola_cladoniae
NCBI1484157	0	0	0	33	141	222	0	0	0	0	0	0	0	0	0	k__Bacteria;p__Pseudomonadota;c__Gammaproteobacteria;o__Enterobacterales;f__Erwiniaceae;g__Pantoea;s__Pantoea_sp._PSNIH2
NCBI148448	0	0	0	2	4	0	0	0	0	0	0	0	0	0	0	k__Archaea;p__Euryarchaeota;c__Halobacteria;o__Natrialbales;f__Natrialbaceae;g__Halobiforma;s__Halobiforma_haloterrestris
NCBI1484693	0	0	0	0	3	13	0	0	0	0	0	0	0	0	0	k__Bacteria;p__Pseudomonadota;c__Betaproteobacteria;o__Burkholderiales;f__Comamonadaceae;g__Rhodofera

																x;s__Rhodoferax_saidenbachensis
NCBI1491	49	47	35	116	220	246	66	104	33	4	27	47	8	13	32	k__Bacteria;p__Bacillota;c__Clostridia;o__Eubacteriales;f__Clostridiaceae;g__Clostridium;s__Clostridium_botulinum
NCBI1492	1	0	0	0	1	1	0	0	0	0	0	0	0	0	0	k__Bacteria;p__Bacillota;c__Clostridia;o__Eubacteriales;f__Clostridiaceae;g__Clostridium;s__Clostridium_butyricum
NCBI1493	0	0	0	0	0	0	0	0	0	0	1	0	0	0	0	k__Bacteria;p__Bacillota;c__Clostridia;o__Eubacteriales;f__Clostridiaceae;g__Clostridium;s__Clostridium_cellulovorans
NCBI1493872	2	0	0	0	0	0	0	0	0	0	0	0	0	0	0	k__Bacteria;p__Bacteroidota;c__Flavobacteriia;o__Flavobacteriales;f__Weeksellaceae;g__Chryseobacterium;s__Chryseobacterium_shandongense
NCBI1495650	0	0	0	0	2	0	0	0	0	0	0	0	0	0	0	k__Bacteria;p__Chloroflexota;c__Thermoflexia;o__Thermoflexales;f__Thermoflexaceae;g__Thermoflexus;s__Thermoflexus_hugenholtzii
NCBI1496	0	0	0	0	0	28	4	0	0	0	0	2	0	0	0	k__Bacteria;p__Bacillota;c__Clostridia;o__Eubacteriales;f__Peptostreptococcaceae;g__Clostridioides;s__Clostridioides_difficile
NCBI1496080	0	0	0	0	3	0	0	0	0	0	0	0	0	0	0	k__Bacteria;p__Actinomycetota;c__Actinomycetes;o__Micrococcales;f__Brevibacteriaceae;g__Brevibacterium;s__Brevibacterium_pigmentatum
NCBI1499686	3	4	3	0	11	109	3	2	0	0	3	3	0	1	0	k__Bacteria;p__Pseudomonadota;c__Gammaproteobacteria;o__Pseudomonadales;f__Pseudomonadaceae;g__Pseudomonas;s__Pseudomonas_saudiphocaensis
NCBI1500392	0	0	0	0	0	21	0	0	0	0	0	0	0	0	0	k__Bacteria;p__;c__;o__;f__;g__;s__uncultured_bacterium_182_02_C03
NCBI1500396	0	0	0	0	0	5	0	0	0	0	0	0	0	0	0	k__Bacteria;p__;c__;o__;f__;g__;s__uncultured_bacterium_182_09_J11

NCBI150055	0	0	0	0	0	26	0	0	0	0	0	0	0	0	0	k__Bacteria;p__Bacillota;c__Bacilli;o__Lactobacillales;f__Streptococcaceae;g__Streptococcus;s__Streptococcus_lutetiensis
NCBI1501392	0	0	0	0	0	0	1	0	0	0	0	0	0	0	0	k__Bacteria;p__Bacteroidota;c__Bacteroidia;o__Bacteroidales;f__Barnesiellaceae;g__Coprobacter;s__Coprobacter_secundus
NCBI1501662	0	0	0	0	0	0	0	0	0	0	0	2	0	0	0	k__Bacteria;p__Bacillota;c__Bacilli;o__Lactobacillales;f__Streptococcaceae;g__Streptococcus;s__Streptococcus_parasuis
NCBI1502	1	0	0	0	8	20	4	0	0	0	3	10	0	0	0	k__Bacteria;p__Bacillota;c__Clostridia;o__Eubacteriales;f__Clostridiaceae;g__Clostridium;s__Clostridium_perfringens
NCBI150396	0	0	0	0	5	50	0	0	0	0	0	0	0	0	0	k__Bacteria;p__Pseudomonadota;c__Gammaproteobacteria;o__Pseudomonadales;f__Pseudomonadaceae;g__Pseudomonas;s__Pseudomonas_sp._MT-1
NCBI1510570	14	1	0	14	8	36	1	0	0	0	0	4	16	0	0	k__Bacteria;p__Pseudomonadota;c__Gammaproteobacteria;o__Enterobacterales;f__Yersiniaceae;g__Rahnella;s__Rahnella_victoriana
NCBI1511	0	0	0	0	1	0	0	0	0	0	0	0	0	0	0	k__Bacteria;p__Bacillota;c__Clostridia;o__Eubacteriales;f__Peptostreptococcaceae;g__Acetoanaerobium;s__Acetoanaerobium_sticklandii
NCBI1513	0	0	0	0	0	0	0	0	0	0	0	1	0	0	0	k__Bacteria;p__Bacillota;c__Clostridia;o__Eubacteriales;f__Clostridiaceae;g__Clostridium;s__Clostridium_tetani
NCBI151419	0	0	0	0	0	108	0	0	0	0	0	0	0	0	0	k__Bacteria;p__Pseudomonadota;c__Alphaproteobacteria;o__Hyphomicrobiales;f__Boseaceae;g__Bosea;s__Bosea_massiliensis
NCBI1515612	3	1	4	11	12	15	1	0	0	0	0	5	0	1	1	k__Bacteria;p__Pseudomonadota;c__Alphaproteobacteria;o__Sphingomonadales;f__Sphingomonadaceae;g__Sphingopyxis;s__Sphingopyxis_fribergensis

NCBI1516059	0	0	0	0	0	2	0	0	0	0	0	0	0	0	0	k__Bacteria;p__Pseudomonadota;c__Gammaproteobacteria;o__Cellvibrionales;f__Microbulbiferaceae;g__Microbulbifer;s__Microbulbifer_sp._ALW1
NCBI1517	0	0	0	0	0	1	0	0	0	0	0	1	0	0	0	k__Bacteria;p__Bacillota;c__Clostridia;o__Thermoanaerobacterales;f__Thermoanaerobacterales_Family_III._Incertae_Sedis;g__Thermoanaerobacterium;s__Thermoanaerobacterium_thermosaccharolyticum
NCBI1517551	0	0	0	1	1	7	2	0	0	0	0	0	0	0	0	k__Bacteria;p__Pseudomonadota;c__Alphaproteobacteria;o__Sphingomonadales;f__Sphingomonadaceae;g__Sphingomonas;s__Sphingomonas_sp._HMP6
NCBI1517554	0	0	0	0	0	0	0	0	0	0	0	0	0	0	1	k__Bacteria;p__Pseudomonadota;c__Alphaproteobacteria;o__Sphingomonadales;f__Sphingomonadaceae;g__Sphingomonas;s__Sphingomonas_sp._HMP9
NCBI151783	10	5	1	0	25	476	26	0	5	0	1	8	2	2	0	k__Bacteria;p__Pseudomonadota;c__Betaproteobacteria;o__Burkholderiales;f__Burkholderiaceae;g__Cupriavidus;s__Cupriavidus_campinensis
NCBI1519377	0	0	0	0	0	0	0	0	0	0	0	5	0	0	0	k__Bacteria;p__Bacillota;c__Bacilli;o__Bacillales;f__Bacillaceae;g__Geobacillus;s__Geobacillus_sp._LC300
NCBI151985	0	0	3	0	3	47	3	0	6	0	0	0	0	0	0	k__Bacteria;p__Pseudomonadota;c__Betaproteobacteria;o__Rhodocyclales;f__Rhodocyclaceae;g__;s__uncultured_Rhodocyclaceae_bacterium
NCBI1520	113	55	76	241	128	148	44	30	64	51	19	17	3	3	9	k__Bacteria;p__Bacillota;c__Clostridia;o__Eubacteriales;f__Clostridiaceae;g__Clostridium;s__Clostridium_beijerinckii
NCBI1521	1	0	0	0	1	0	0	0	0	0	0	0	0	0	0	k__Bacteria;p__Bacillota;c__Clostridia;o__Eubacteriales;f__Oscillospiraceae;g__Ruminiclostridium;s__Ruminiclostridium_cellulolyticum
NCBI1521117	0	0	0	0	1	4	0	0	0	0	0	0	0	0	0	k__Bacteria;p__Myxococcota;c__Myxococcia;o__Myxococcales;f__Myxococcaceae;g__Corallococcus;s__Corallococcus_sp._EGB

NCBI152142	0	0	0	0	0	1	0	0	0	0	0	0	0	0	0	k__Bacteria;p__Actinomycetota;c__Actinomycetes;o__Mycobacteriales;f__Mycobacteriaceae;g__Mycolicibacterium;s__Mycolicibacterium_holsaticum
NCBI1522	0	0	17	0	0	22	0	0	0	0	0	14	0	0	0	k__Bacteria;p__Bacillota;c__Erysipelotrichia;o__Erysipelotrichales;f__Coprobacillaceae;g__Thomasclavelia;s__[Clostridium]_innocuum
NCBI152331	356	396	425	518	561	331	404	346	417	344	300	283	140	132	136	k__Bacteria;p__Bacillota;c__Bacilli;o__Lactobacillales;f__Lactobacillaceae;g__Lentilactobacillus;s__Lentilactobacillus_parabuchneri
NCBI1523415	0	2	1	0	1	14	0	0	0	2	0	2	0	0	0	k__Bacteria;p__Pseudomonadota;c__Alphaproteobacteria;o__Sphingomonadales;f__Sphingomonadaceae;g__Sphingomonas;s__Sphingomonas_sp._AAP5
NCBI152480	0	3	0	0	0	0	0	0	0	0	0	0	0	0	0	k__Bacteria;p__Pseudomonadota;c__Betaproteobacteria;o__Burkholderiales;f__Burkholderiaceae;g__Burkholderia;s__Burkholderia_ambifaria
NCBI152507	0	0	0	33	52	130	0	12	0	0	0	9	0	0	0	k__Bacteria;p__Actinomycetota;c__Actinomycetes;o__;f__;g__;s__uncultured_Actinomycetes_bacterium
NCBI152682	20	17	10	46	101	821	12	9	12	0	6	17	21	0	20	k__Bacteria;p__Pseudomonadota;c__Alphaproteobacteria;o__Sphingomonadales;f__Sphingomonadaceae;g__Sphingomonas;s__Sphingomonas_melonis
NCBI152794	0	0	0	0	0	3	0	0	0	0	0	0	0	0	0	k__Bacteria;p__Actinomycetota;c__Actinomycetes;o__Mycobacteriales;f__Corynebacteriaceae;g__Corynebacterium;s__Corynebacterium_efficiens
NCBI1528099	0	2	0	1	0	1	0	0	0	0	0	0	0	0	0	k__Bacteria;p__Actinomycetota;c__Actinomycetes;o__Mycobacteriales;f__Lawsonellaceae;g__Lawsonella;s__Lawsonella_clevelandensis
NCBI152831	19	25	12	57	76	49	22	13	48	13	32	18	49	15	55	k__Bacteria;p__Pseudomonadota;c__Gammaproteobacteria;o__Xanthomonadales;f__Xanthomonadaceae;g__Xanthomonas;s__uncultured_Xanthomonas_sp.

NCBI1530123	1	0	28	5	6	6	0	4	0	0	0	175	0	0	0	k__Bacteria;p__Pseudomonadota;c__Gammaproteobacteria;o__Moraxellales;f__Moraxellaceae;g__Acinetobacter;s__Acinetobacter_seifertii
NCBI1531	0	0	0	0	3	7	66	0	0	0	0	4	0	0	0	k__Bacteria;p__Bacillota;c__Clostridia;o__Eubacteriales;f__Lachnospiraceae;g__Enterocloster;s__Enterocloster_clostridioformis
NCBI153152	2287	2428	2479	3679	4143	10706	2938	2419	2983	2722	2193	2356	1096	1105	1084	k__Bacteria;p__Bacillota;c__Bacilli;o__Lactobacillales;f__Lactobacillaceae;g__Lactobacillus;s__uncultured_Lactobacillus_sp.
NCBI1532555	454	482	340	63	1682	18453	644	44	162	117	97	261	57	60	65	k__Bacteria;p__Pseudomonadota;c__Alphaproteobacteria;o__Caulobacterales;f__Caulobacteraceae;g__Brevundimonas;s__Brevundimonas_sp._DS20
NCBI153496	3580	3457	3600	9168	8777	8325	6181	4246	5991	30406	27277	40946	38602	40204	45147	k__Bacteria;p__Pseudomonadota;c__Alphaproteobacteria;o__Rhodospirillales;f__Acetobacteraceae;g__Kozakia;s__Kozakia_baliensis
NCBI1536772	0	0	0	2	0	0	0	0	0	0	0	0	0	0	0	k__Bacteria;p__Bacillota;c__Bacilli;o__Bacillales;f__Paenibacillaceae;g__Paenibacillus;s__Paenibacillus_sp._FSL_R7-0273
NCBI1537274	0	0	0	0	0	10	0	0	0	0	0	2	0	0	0	k__Bacteria;p__Pseudomonadota;c__Betaproteobacteria;o__Burkholderiales;f__Oxalobacteraceae;g__Janthinobacterium;s__Janthinobacterium_sp._HH102
NCBI1537400	0	0	0	0	0	4	0	0	0	0	0	0	0	0	0	k__Bacteria;p__Pseudomonadota;c__Betaproteobacteria;o__Neisseriales;f__Chromobacteriaceae;g__Aquitalea;s__Aquitalea_aquatilis
NCBI1538	0	0	0	0	7	156	0	0	0	0	0	0	0	0	0	k__Bacteria;p__Bacillota;c__Clostridia;o__Eubacteriales;f__Clostridiaceae;g__Clostridium;s__Clostridium_ljungdahlii
NCBI1538159	0	0	0	0	0	6	0	0	0	0	0	0	0	0	0	k__Bacteria;p__Pseudomonadota;c__Alphaproteobacteria;o__Hyphomicrobiales;f__Rhizobiaceae;g__Rhizobium;s__Rhizobium_hidalgonense

NCBI154046	0	0	0	0	0	0	0	0	0	0	0	1	0	0	0	k__Bacteria;p__Bacillota;c__Clostridia;o__Eubacteriales;f__Clostridiaceae;g__Hungatella;s__Hungatella_hathewayi
NCBI1541173	0	0	0	0	0	0	0	0	0	2	4	0	10	10	5	k__Bacteria;p__;c__;o__;f__;g__;s__bacterium_NJ6
NCBI1544730	0	0	0	0	0	2	0	0	0	0	0	0	0	0	0	k__Bacteria;p__Actinomycetota;c__Actinomycetes;o__Propionibacteriales;f__Kribbellaceae;g__Kribbella;s__Kribbella_qitaiheensis
NCBI1545044	0	4	3	15	7	48	0	4	3	0	0	5	0	6	0	k__Bacteria;p__Pseudomonadota;c__Alphaproteobacteria;o__Rhodobacterales;f__Paracoccaceae;g__Paracoccus;s__Paracoccus_sanguinis
NCBI1545728	0	0	0	0	0	0	1	0	0	0	0	0	0	0	0	k__Bacteria;p__Actinomycetota;c__Actinomycetes;o__Mycobacteriales;f__Mycobacteriaceae;g__Mycobacterium;s__Mycobacterium_sp._EPa45
NCBI1547	0	0	11	0	2	2	0	0	0	0	0	5	0	0	0	k__Bacteria;p__Bacillota;c__Erysipelotrichia;o__Erysipelotrichales;f__Coprobacillaceae;g__Thomasclavelia;s__Thomasclavelia_ramosa
NCBI1547448	0	0	0	0	0	3	0	0	0	0	0	1	0	0	0	k__Bacteria;p__Actinomycetota;c__Actinomycetes;o__Propionibacteriales;f__Propionibacteriaceae;g__Arachnia;s__Arachnia_rubra
NCBI1549858	1	0	0	0	9	7	1	1	0	0	0	0	0	0	0	k__Bacteria;p__Pseudomonadota;c__Alphaproteobacteria;o__Sphingomonadales;f__Sphingomonadaceae;g__Sphingomonas;s__Sphingomonas_taxi
NCBI1549949	0	0	0	0	1	1	0	0	0	0	0	0	0	0	0	k__Bacteria;p__Pseudomonadota;c__Alphaproteobacteria;o__Hyphomicrobiales;f__Nitrobacteraceae;g__Bradyrhizobium;s__Bradyrhizobium_vignae
NCBI1550024	0	0	0	1	0	28	0	1	1	0	0	22	0	0	0	k__Bacteria;p__Bacillota;c__Clostridia;o__Eubacteriales;f__Oscillospiraceae;g__Ruthenibacterium;s__Ruthenibacterium_lactatiformans

NCBI1550728	0	4	0	0	2	78	2	0	0	0	0	0	0	0	0	k__Bacteria;p__Pseudomonadota;c__Alphaproteobacteria;o__Sphingomonadales;f__Sphingomonadaceae;g__Blastomonas;s__Blastomonas_fulva
NCBI1550733	0	1	0	0	2	0	3	0	1	0	0	1	0	0	0	k__Bacteria;p__Pseudomonadota;c__Alphaproteobacteria;o__Sphingomonadales;f__Sphingomonadaceae;g__Aquisediminimonas;s__Aquisediminimonas_profunda
NCBI155077	0	0	1	0	0	0	0	0	0	0	0	0	0	0	0	k__Bacteria;p__Pseudomonadota;c__Gammaproteobacteria;o__Cellvibrionales;f__Cellvibrionaceae;g__Cellvibrio;s__Cellvibrio_japonicus
NCBI155177	0	0	0	0	0	10	0	0	0	0	0	9	0	0	0	k__Bacteria;p__Actinomycetota;c__Actinomycetes;o__Kitasatosporales;f__Streptomycetaceae;g__Streptomyces;s__Streptomyces_yatensis
NCBI1552759	0	0	0	0	0	0	0	0	0	0	0	1	0	0	0	k__Bacteria;p__Actinomycetota;c__Actinomycetes;o__Mycobacteriales;f__Mycobacteriaceae;g__Mycobacterium;s__Mycobacterium_grossiae
NCBI155322	9	25	11	39	24	50	33	10	8	10	19	0	0	0	0	k__Bacteria;p__Bacillota;c__Bacilli;o__Bacillales;f__Bacillaceae;g__Bacillus;s__Bacillus_toyonensis
NCBI155892	29	25	30	0	102	1060	25	2	17	9	0	14	2	3	3	k__Bacteria;p__Pseudomonadota;c__Alphaproteobacteria;o__Caulobacterales;f__Caulobacteraceae;g__Caulobacter;s__Caulobacter_vibrioides
NCBI1560345	0	0	0	0	1	1	0	0	0	1	0	0	1	0	0	k__Bacteria;p__Pseudomonadota;c__Alphaproteobacteria;o__Sphingomonadales;f__Sphingomonadaceae;g__Sphingomonas;s__Sphingomonas_panacis
NCBI1564114	0	0	0	0	0	2	0	0	0	0	0	0	0	0	0	k__Bacteria;p__Actinomycetota;c__Actinomycetes;o__Mycobacteriales;f__Nocardiaceae;g__Rhodococcus;s__Rhodococcus_sp._B7740
NCBI1565605	0	0	0	0	0	16	1	0	0	0	0	0	0	0	0	k__Bacteria;p__Pseudomonadota;c__Betaproteobacteria;o__Rhodocyclales;f__Rhodocyclaceae;g__Rugosibacter;s__Rugosibacter_aromaticivorans
NCBI1	0	0	0	0	0	27	0	0	0	0	0	0	0	0	0	k__Bacteria;p__Verrucomicrobiota;c__;o__;f__;g__;s__

56588																uncultured_Verrucomicrobiota_bacterium
NCBI156630	0	0	0	0	1	2	0	0	0	0	0	0	0	0	0	k__Fungi;p__Ascomycota;c__Dothideomycetes;o__Pleosporales;f__Pleosporaceae;g__Alternaria;s__Alternaria_arborescens
NCBI156977	0	0	0	0	0	0	0	3	0	0	0	0	0	0	0	k__Bacteria;p__Actinomycetota;c__Actinomycetes;o__Micrococcales;f__Microbacteriaceae;g__Microbacterium;s__Microbacterium_resistens
NCBI156978	0	0	0	0	0	0	1	0	0	0	0	0	0	0	0	k__Bacteria;p__Actinomycetota;c__Actinomycetes;o__Mycobacteriales;f__Corynebacteriaceae;g__Corynebacterium;s__Corynebacterium_imitans
NCBI1571470	0	0	0	0	0	9	0	0	0	0	0	0	0	0	0	k__Bacteria;p__Pseudomonadota;c__Alphaproteobacteria;o__Hyphomicrobiales;f__Rhizobiaceae;g__Rhizobium;s__Rhizobium_sp._ACO-34A
NCBI1573458	0	0	0	0	1	2	0	0	0	0	0	0	0	0	0	k__Heunggongvirae;p__Uroviricota;c__Caudoviricetes;o__;f__;g__;s__Pseudomonas_phage_PS-1
NCBI1573704	0	0	0	0	18	30	10	0	0	0	0	0	0	0	0	k__Bacteria;p__Pseudomonadota;c__Gammaproteobacteria;o__Pseudomonadales;f__Pseudomonadaceae;g__Pseudomonas;s__Pseudomonas_sp._R32
NCBI1574161	0	0	0	0	0	7	0	0	2	0	0	0	0	0	0	k__Bacteria;p__Pseudomonadota;c__Gammaproteobacteria;o__Enterobacterales;f__Morganellaceae;g__Proteus;s__Proteus_terrae
NCBI1575	0	0	0	1	2	0	0	0	0	2	0	0	0	0	0	k__Bacteria;p__Actinomycetota;c__Actinomycetes;o__Micrococcales;f__Microbacteriaceae;g__Leifsonia;s__Leifsonia_xyli
NCBI157782	0	0	0	3	0	0	0	0	0	0	0	0	0	0	0	k__Bacteria;p__Pseudomonadota;c__Gammaproteobacteria;o__Pseudomonadales;f__Pseudomonadaceae;g__Pseudomonas;s__Pseudomonas_parafulva
NCBI1579	32	48	28	63	62	111	47	45	57	61	26	50	14	26	24	k__Bacteria;p__Bacillota;c__Bacilli;o__Lactobacillales;f__Lactobacillaceae;g__Lactobacillus;s__Lactobacillus_acidophilus

NCBI1580	1341	1337	1299	1820	1678	526	2065	1797	1910	2287	1852	2299	895	961	921	k__Bacteria;p__Bacillota;c__Bacilli;o__Lactobacillales;f__Lactobacillaceae;g__Levilactobacillus;s__Levilactobacillus_brevis
NCBI1580596	4	0	0	0	0	43	2	0	0	0	0	0	0	0	0	k__Bacteria;p__Pseudomonadota;c__Alphaproteobacteria;o__Rhodobacterales;f__Roseobacteraceae;g__Phaeobacter;s__Phaeobacter_piscinae
NCBI158080	0	0	0	0	0	0	1	0	0	0	0	0	0	0	0	k__Bacteria;p__Pseudomonadota;c__Gammaproteobacteria;o__Oceanospirillales;f__Halomonadaceae;g__Chromohalobacter;s__Chromohalobacter_salexigens
NCBI1581	1492	1384	1423	2531	2417	1055	1541	1283	1365	1141	878	1337	452	473	553	k__Bacteria;p__Bacillota;c__Bacilli;o__Lactobacillales;f__Lactobacillaceae;g__Lentilactobacillus;s__Lentilactobacillus_buchneri
NCBI1581011	0	0	0	0	1	0	0	0	0	0	0	0	0	0	0	k__Bacteria;p__Campylobacterota;c__Epsilonproteobacteria;o__Campylobacterales;f__Sulfurospirillaceae;g__Sulfurospirillum;s__Sulfurospirillum_sp._UCH001
NCBI1582	48747	51863	54811	86999	80139	14670	81425	81996	81038	97079	83343	101514	37421	42985	44582	k__Bacteria;p__Bacillota;c__Bacilli;o__Lactobacillales;f__Lactobacillaceae;g__Lacticaseibacillus;s__Lacticaseibacillus_casei
NCBI1583	0	0	0	0	0	2	2	0	0	0	0	0	0	0	0	k__Bacteria;p__Bacillota;c__Bacilli;o__Lactobacillales;f__Lactobacillaceae;g__Weissella;s__Weissella_confusa
NCBI1583341	0	0	0	0	14	5	0	0	0	0	0	0	0	0	0	k__Bacteria;p__Pseudomonadota;c__Gammaproteobacteria;o__Pseudomonadales;f__Pseudomonadaceae;g__Pseudomonas;s__Pseudomonas_cerasi
NCBI1584	0	0	1	0	10	218	3	0	4	1	0	14	0	0	1	k__Bacteria;p__Bacillota;c__Bacilli;o__Lactobacillales;f__Lactobacillaceae;g__Lactobacillus;s__Lactobacillus_delbrueckii
NCBI158500	3	0	1	0	3	25	0	0	0	0	0	0	0	0	0	k__Bacteria;p__Pseudomonadota;c__Alphaproteobacteria;o__Sphingomonadales;f__Sphingomonadaceae;g__Novosphingobium;s__Novosphingobium_resinovorum

NCBI158627	16	10	5	108	238	419	32	7	14	2	0	4	5	0	5	k__Bacteria;p__Pseudomonadota;c__Gammaproteobacteria;o__Pseudomonadales;f__Pseudomonadaceae;g__Pseudomonas;s__Pseudomonas_graminis
NCBI1586287	0	0	0	0	0	45	0	0	0	0	0	1	0	0	0	k__Bacteria;p__Actinomycetota;c__Actinomycetes;o__Pseudonocardiales;f__Pseudonocardiaceae;g__Lentzea;s__Lentzea_guizhouensis
NCBI1587	200	213	210	285	276	203	250	281	275	440	347	409	215	236	247	k__Bacteria;p__Bacillota;c__Bacilli;o__Lactobacillales;f__Lactobacillaceae;g__Lactobacillus;s__Lactobacillus_helveticus
NCBI158751	0	0	56	41	59	1412	180	0	0	0	0	0	0	0	0	k__Bacteria;p__Pseudomonadota;c__Betaproteobacteria;o__Burkholderiales;f__Comamonadaceae;g__Acidovorax;s__uncultured_Acidovorax_sp.
NCBI158789	57	49	42	72	135	302	42	27	31	58	31	37	15	21	12	k__Bacteria;p__Deinococcota;c__Deinococci;o__Deinococcales;f__Deinococcaceae;g__Deinococcus;s__uncultured_Deinococcus_sp.
NCBI1588	81847	79674	83097	122047	123681	36233	84012	78098	85313	65565	54740	72115	21375	22569	22033	k__Bacteria;p__Bacillota;c__Bacilli;o__Lactobacillales;f__Lactobacillaceae;g__Lentilactobacillus;s__Lentilactobacillus_hilgardii
NCBI158822	0	0	0	0	6	0	0	0	0	0	0	5	0	0	0	k__Bacteria;p__Pseudomonadota;c__Gammaproteobacteria;o__Enterobacterales;f__Enterobacteriaceae;g__Cedecea;s__Cedecea_neteri
NCBI158836	17	0	75	0	102	268	59	0	0	0	0	304	0	29	0	k__Bacteria;p__Pseudomonadota;c__Gammaproteobacteria;o__Enterobacterales;f__Enterobacteriaceae;g__Enterobacter;s__Enterobacter_hormaechei
NCBI1589	648	681	645	1789	1899	232	631	737	794	659	590	842	285	278	306	k__Bacteria;p__Bacillota;c__Bacilli;o__Lactobacillales;f__Lactobacillaceae;g__Lactiplantibacillus;s__Lactiplantibacillus_pentosus
NCBI1590	88338	86076	85559	160117	145856	66857	100436	109574	116376	131979	108378	140989	63367	64586	72423	k__Bacteria;p__Bacillota;c__Bacilli;o__Lactobacillales;f__Lactobacillaceae;g__Lactiplantibacillus;s__Lactiplantibacillus_plantarum

NCBI159090	0	0	2	0	3	7	0	0	14	0	0	8	0	0	0	k__Archaea;p__Thermoproteota;c__Thermoprotei;o__Sulfolobales;f__Sulfolobaceae;g__Sulfolobus;s__uncultured_Sulfolobus_sp.
NCBI1592790	8	20	4	0	31	403	15	0	1	3	0	2	0	0	0	k__Bacteria;p__Pseudomonadota;c__Alphaproteobacteria;o__Sphingomonadales;f__Sphingomonadaceae;g__Sphingobium;s__Sphingobium_phenoxybenzoativorans
NCBI1596	0	1	1	7	108	7	3	7	3	1	1	1	0	3	0	k__Bacteria;p__Bacillota;c__Bacilli;o__Lactobacillales;f__Lactobacillaceae;g__Lactobacillus;s__Lactobacillus_gasseri
NCBI1597	957979	976119	1010970	1394426	1244275	334254	1386646	1182748	1371214	1354764	1181927	1615585	510634	531691	555669	k__Bacteria;p__Bacillota;c__Bacilli;o__Lactobacillales;f__Lactobacillaceae;g__Lacticaseibacillus;s__Lacticaseibacillus_paracasei
NCBI159733	12	23	21	54	70	96	30	25	17	9	12	16	13	15	9	k__Bacteria;p__Cyanobacteriota;c__Cyanophyceae;o__Synechococcales;f__Prochlorococcaceae;g__Prochlorococcus;s__uncultured_Prochlorococcus_sp.
NCBI1598	8	40	14	16	28	204	51	30	10	23	6	79	8	14	12	k__Bacteria;p__Bacillota;c__Bacilli;o__Lactobacillales;f__Lactobacillaceae;g__Limosilactobacillus;s__Limosilactobacillus_reuteri
NCBI1599	47	6	18	9	93	33	3	13	5	25	7	5	7	6	5	k__Bacteria;p__Bacillota;c__Bacilli;o__Lactobacillales;f__Lactobacillaceae;g__Latilactobacillus;s__Latilactobacillus_sakei
NCBI1600	128	122	135	174	188	120	121	148	146	122	95	119	31	36	42	k__Bacteria;p__Bacillota;c__Bacilli;o__Lactobacillales;f__Lactobacillaceae;g__Lactobacillus;s__Lactobacillus_acetotolerans
NCBI1601	0	0	0	0	0	1	0	0	0	0	0	0	0	0	0	k__Bacteria;p__Bacillota;c__Bacilli;o__Lactobacillales;f__Lactobacillaceae;g__Ligilactobacillus;s__Ligilactobacillus_agilis
NCBI160386	0	0	0	0	0	62	0	0	0	0	0	0	0	0	0	k__Bacteria;p__Actinomycetota;c__Actinomycetes;o__Mycobacteriales;f__Corynebacteriaceae;g__Corynebacterium;s__Corynebacterium_casei

NCBI1604	16	11	26	23	33	52	34	25	30	15	14	32	9	1	5	k__Bacteria;p__Bacillota;c__Bacilli;o__Lactobacillales;f__Lactobacillaceae;g__Lactobacillus;s__Lactobacillus_amylovorus
NCBI1605	0	0	0	0	2	3	0	0	0	0	0	1	0	0	0	k__Bacteria;p__Bacillota;c__Bacilli;o__Lactobacillales;f__Lactobacillaceae;g__Ligilactobacillus;s__Ligilactobacillus_animalis
NCBI160699	0	0	0	0	0	1	0	0	0	0	0	0	0	0	0	k__Bacteria;p__Pseudomonadota;c__Alphaproteobacteria;o__Hyphomicrobiales;f__Rhizobiaceae;g__Agrobacterium;s__Agrobacterium_larrymoorei
NCBI1607	0	0	0	0	0	0	0	1	0	1	0	1	0	0	0	k__Bacteria;p__Bacillota;c__Bacilli;o__Lactobacillales;f__Lactobacillaceae;g__Loigolactobacillus;s__Loigolactobacillus_bifermentans
NCBI160791	4	2	0	5	18	205	3	0	14	0	3	9	4	4	13	k__Bacteria;p__Pseudomonadota;c__Alphaproteobacteria;o__Sphingomonadales;f__Sphingomonadaceae;g__Rhizorhabdus;s__Rhizorhabdus_wittichii
NCBI160825	0	1	1	3	4	41	1	0	6	0	0	0	0	0	0	k__Bacteria;p__Pseudomonadota;c__Betaproteobacteria;o__Burkholderiales;f__Comamonadaceae;g__Comamonas;s__Comamonas_koreensis
NCBI160826	0	0	0	0	0	0	2	0	0	0	0	0	0	0	0	k__Bacteria;p__Actinomycetota;c__Actinomycetes;o__Propionibacteriales;f__Nocardioidaceae;g__Nocardioides;s__Nocardioides_aquaticus
NCBI1609758	1	0	0	0	0	1	0	0	0	0	0	0	0	0	0	k__Bacteria;p__Pseudomonadota;c__Alphaproteobacteria;o__Sphingomonadales;f__Sphingomonadaceae;g__Novosphingobium;s__Novosphingobium_sp._P6W
NCBI1609966	0	0	0	0	0	18	0	0	0	0	0	0	0	0	0	k__Bacteria;p__Pseudomonadota;c__Alphaproteobacteria;o__Rhodobacterales;f__Roseobacteraceae;g__Actibacterium;s__Actibacterium_sp._EMB200-NS6
NCBI1609977	0	0	0	0	0	2	0	0	0	0	0	0	0	0	0	k__Bacteria;p__Pseudomonadota;c__Alphaproteobacteria;o__Sphingomonadales;f__Sphingomonadaceae;g__Sphingomonas;s__Sphingomonas_hengshuiensis

NCBI1610	9	1	3	6	1	5	3	3	2	5	7	3	7	2	6	k__Bacteria;p__Bacillota;c__Bacilli;o__Lactobacillales;f__Lactobacillaceae;g__Loigolactobacillus;s__Loigolactobacillus_coryniformis
NCBI1610493	0	0	0	0	0	12	0	0	0	0	0	0	0	0	0	k__Bacteria;p__Actinomycetota;c__Actinomycetes;o__Propionibacteriales;f__Propionibacteriaceae;g__Tessaracoccus;s__Tessaracoccus_flavus
NCBI161154	0	0	0	0	0	1	0	0	0	0	0	0	0	0	0	k__Bacteria;p__Bacillota;c__Clostridia;o__Thermoanaerobacterales;f__Thermoanaerobacteraceae;g__Thermanaeromonas;s__Thermanaeromonas_toyohensis
NCBI1612173	0	0	0	0	2	31	2	0	0	0	0	0	0	0	0	k__Bacteria;p__Pseudomonadota;c__Alphaproteobacteria;o__Rhodospirillales;f__Azospirillaceae;g__Niveispirillum;s__Niveispirillum_cyanobacteriorum
NCBI1613	16934	17179	16475	38023	36066	45354	20647	11636	16987	16262	13861	21567	9632	9314	11032	k__Bacteria;p__Bacillota;c__Bacilli;o__Lactobacillales;f__Lactobacillaceae;g__Limosilactobacillus;s__Limosilactobacillus_fermentum
NCBI161493	0	0	0	0	0	105	0	0	0	0	0	0	0	0	0	k__Bacteria;p__Myxococcota;c__Myxococcia;o__Myxococcales;f__Anaeromyxobacteraceae;g__Anaeromyxobacter;s__Anaeromyxobacter_dehalogenans
NCBI1615674	0	2	0	4	5	4	51	0	0	0	0	0	0	0	0	k__Bacteria;p__Pseudomonadota;c__Gammaproteobacteria;o__Pseudomonadales;f__Pseudomonadaceae;g__Pseudomonas;s__Pseudomonas_lactis
NCBI1618	39	45	46	40	38	27	46	51	38	57	60	84	43	35	36	k__Bacteria;p__Bacillota;c__Bacilli;o__Lactobacillales;f__Lactobacillaceae;g__Liquorilactobacillus;s__Liquorilactobacillus_mali
NCBI161899	0	0	0	20	0	17	0	6	2	0	7	2	0	2	0	k__Bacteria;p__Actinomycetota;c__Actinomycetes;o__Mycobacteriales;f__Corynebacteriaceae;g__Corynebacterium;s__Corynebacterium_singulare
NCBI1619313	1	0	3	0	5	7	0	0	0	0	0	0	0	0	0	k__Bacteria;p__Pseudomonadota;c__Gammaproteobacteria;o__Enterobacterales;f__Erwiniaceae;g__Duffyella;s__Duffyella_gerundensis

NCBI1620215	1	0	0	2	1	0	0	0	0	0	0	0	0	0	0	k__Bacteria;p__Pseudomonadota;c__Gammaproteobacteria;o__Acidiferrobacterales;f__Acidiferrobacteraceae;g__Sulfuricaulis;s__Sulfuricaulis_limicola
NCBI1620421	0	0	0	0	0	1	0	0	0	0	0	0	0	0	0	k__Bacteria;p__Pseudomonadota;c__Alphaproteobacteria;o__Hyphomicrobiales;f__Phyllobacteriaceae;g__Hoeflea;s__Hoeflea_sp._IMCC20628
NCBI1622	5	6	0	0	11	44	49	3	8	0	0	165	0	0	3	k__Bacteria;p__Bacillota;c__Bacilli;o__Lactobacillales;f__Lactobacillaceae;g__Ligilactobacillus;s__Ligilactobacillus_murinus
NCBI1623	0	0	0	0	1	5	0	0	0	0	0	17	0	0	0	k__Bacteria;p__Bacillota;c__Bacilli;o__Lactobacillales;f__Lactobacillaceae;g__Ligilactobacillus;s__Ligilactobacillus_ruminis
NCBI1624	2	1	3	2	0	2	5	8	1	7	2	4	0	0	0	k__Bacteria;p__Bacillota;c__Bacilli;o__Lactobacillales;f__Lactobacillaceae;g__Ligilactobacillus;s__Ligilactobacillus_salivarius
NCBI162425	0	2	0	0	0	0	0	0	0	0	0	1	0	0	0	k__Fungi;p__Ascomycota;c__Eurotiomycetes;o__Eurotiales;f__Aspergillaceae;g__Aspergillus;s__Aspergillus_nidulans
NCBI162426	0	0	0	0	0	0	0	53	0	0	0	0	8	44	0	k__Bacteria;p__Actinomycetota;c__Actinomycetes;o__Micrococcales;f__Microbacteriaceae;g__Microbacterium;s__Microbacterium_hominis
NCBI162496	0	0	0	0	0	2	0	0	0	0	0	1	0	0	0	k__Bacteria;p__Actinomycetota;c__Actinomycetes;o__Micrococcales;f__Micrococcaceae;g__Glutamicibacter;s__Glutamicibacter_creatinolyticus
NCBI1625	0	0	0	1	6	2	2	2	1	0	3	1	0	0	0	k__Bacteria;p__Bacillota;c__Bacilli;o__Lactobacillales;f__Lactobacillaceae;g__Fructilactobacillus;s__Fructilactobacillus_sanfranciscensis
NCBI1628248	2	0	0	0	1	0	0	0	0	0	0	0	0	0	1	k__Bacteria;p__Bacteroidota;c__Flavobacteriia;o__Flavobacteriales;f__Weeksellaceae;g__Empedobacter;s__Empedobacter_stercoris

NCBI1629	0	0	0	0	0	2	0	0	0	0	0	0	0	0	0	k__Bacteria;p__Bacillota;c__Bacilli;o__Lactobacillales;f__Lactobacillaceae;g__Weissella;s__Weissella_viridescens
NCBI1629723	0	0	0	0	0	0	0	0	0	0	0	13	0	0	0	k__Bacteria;p__Bacillota;c__Bacilli;o__Bacillales;f__Bacillaceae;g__Geobacillus;s__Geobacillus_sp._12AMOR1
NCBI1632	6	12	9	34	27	45	12	14	12	0	12	12	10	0	3	k__Bacteria;p__Bacillota;c__Bacilli;o__Lactobacillales;f__Lactobacillaceae;g__Limosilactobacillus;s__Limosilactobacillus_oris
NCBI1632864	0	0	0	0	1	0	0	0	0	0	0	0	0	0	0	k__Bacteria;p__Planctomycetota;c__Planctomycetia;o__Planctomycetales;f__Planctomycetaceae;g__Planctomyces;s__Planctomyces_sp._SH-PL14
NCBI1633	0	0	0	0	0	6	0	0	0	0	0	1	0	0	0	k__Bacteria;p__Bacillota;c__Bacilli;o__Lactobacillales;f__Lactobacillaceae;g__Limosilactobacillus;s__Limosilactobacillus_vaginalis
NCBI1633874	39072	40157	40982	136568	105038	78132	51752	33288	49242	117000	110199	169069	1068745	1088833	1345722	k__Bacteria;p__Pseudomonadota;c__Alphaproteobacteria;o__Rhodospirillales;f__Acetobacteraceae;g__Acetobacter;s__Acetobacter_oryzifermentans
NCBI1636152	0	0	0	0	0	3	0	0	0	0	0	0	0	0	0	k__Bacteria;p__Planctomycetota;c__Planctomycetia;o__Planctomycetales;f__Planctomycetaceae;g__Planctomyces;s__Planctomyces_sp._SH-PL62
NCBI1636606	0	0	0	0	0	21	0	0	0	0	0	0	0	0	0	k__Bacteria;p__Pseudomonadota;c__Gammaproteobacteria;o__Aeromonadales;f__Aeromonadaceae;g__Aeromonas;s__Aeromonas_sp._ASNIH1
NCBI1636607	0	0	0	0	0	0	0	0	0	0	0	36	0	0	0	k__Bacteria;p__Pseudomonadota;c__Gammaproteobacteria;o__Aeromonadales;f__Aeromonadaceae;g__Aeromonas;s__Aeromonas_sp._ASNIH2
NCBI1636609	7	0	6	0	13	275	12	0	0	0	10	0	0	0	0	k__Bacteria;p__Pseudomonadota;c__Gammaproteobacteria;o__Aeromonadales;f__Aeromonadaceae;g__Aeromonas;s__Aeromonas_sp._ASNIH4

NCBI1636610	10	0	8	0	29	55	0	0	0	0	0	13	0	0	0	k__Bacteria;p__Pseudomonadota;c__Gammaproteobacteria;o__Pseudomonadales;f__Pseudomonadaceae;g__Pseudomonas;s__Pseudomonas_sp._PONIH3
NCBI1636720	0	0	0	0	0	5	0	0	0	0	0	16	0	0	0	k__Bacteria;p__Bacillota;c__Bacilli;o__Bacillales;f__Bacillaceae;g__Anoxybacillus;s__Anoxybacillus_sp._PDR2
NCBI1638223	0	0	0	0	0	2	0	0	0	0	0	0	0	0	0	k__Archaea;p__Euryarchaeota;c__Thermococci;o__Thermococcales;f__Thermococcaceae;g__Thermococcus;s__Thermococcus_sp._21S9
NCBI1638261	0	0	0	0	0	6	0	0	0	0	0	0	0	0	0	k__Archaea;p__Euryarchaeota;c__Thermococci;o__Thermococcales;f__Thermococcaceae;g__Thermococcus;s__Thermococcus_sp._M36
NCBI1638263	0	0	0	0	0	3	0	0	0	0	0	0	0	0	0	k__Archaea;p__Euryarchaeota;c__Thermococci;o__Thermococcales;f__Thermococcaceae;g__Thermococcus;s__Thermococcus_sp._MAR1
NCBI1639	0	0	1	0	3	3	51	0	3	0	0	7	0	0	0	k__Bacteria;p__Bacillota;c__Bacilli;o__Bacillales;f__Listeriaceae;g__Listeria;s__Listeria_monocytogenes
NCBI1639133	0	0	52	232	470	971	0	0	61	0	0	40	32	37	0	k__Bacteria;p__Pseudomonadota;c__Gammaproteobacteria;o__Enterobacterales;f__Enterobacteriaceae;g__Citrobacter;s__Citrobacter_portucalensis
NCBI1641402	0	2	0	0	0	7	0	0	0	0	0	0	0	0	0	k__Bacteria;p__Actinomycetota;c__Actinomycetes;o__Pseudonocardiales;f__Pseudonocardiaceae;g__Pseudonocardia;s__Pseudonocardia_sp._HH130629-09
NCBI1642	0	0	0	0	0	0	1	0	0	0	0	0	0	0	0	k__Bacteria;p__Bacillota;c__Bacilli;o__Bacillales;f__Listeriaceae;g__Listeria;s__Listeria_innocua
NCBI1643	0	0	0	0	0	0	0	0	0	0	0	1	0	0	0	k__Bacteria;p__Bacillota;c__Bacilli;o__Bacillales;f__Listeriaceae;g__Listeria;s__Listeria_welshimeri
NCBI1644131	0	0	0	0	0	1	0	0	0	0	0	0	0	0	0	k__Bacteria;p__Pseudomonadota;c__Betaproteobacteria;o__Burkholderiales;f__Oxalobacteraceae;g__Janthinobacterium;s__Janthinobacterium_sp._1_2014MBL_MicDiv

NCBI164546	0	0	1	0	2	47	0	0	0	0	0	0	0	0	0	k__Bacteria;p__Pseudomonadota;c__Betaproteobacteria;o__Burkholderiales;f__Burkholderiaceae;g__Cupriavidus;s__Cupriavidus_taiwanensis
NCBI164608	25	68	50	9	124	1373	59	15	17	0	0	26	12	8	31	k__Bacteria;p__Pseudomonadota;c__Alphaproteobacteria;o__Sphingomonadales;f__Sphingomonadaceae;g__Novosphingobium;s__Novosphingobium_sp._KA1
NCBI1646377	0	0	1	0	0	0	0	1	0	0	0	2	0	0	0	k__Bacteria;p__Pseudomonadota;c__Gammaproteobacteria;o__Enterobacterales;f__Yersiniaceae;g__Rouxiella;s__Rouxiella_badensis
NCBI1646498	0	0	1	0	0	1	0	4	0	0	0	0	0	0	0	k__Bacteria;p__Pseudomonadota;c__Gammaproteobacteria;o__Moraxellales;f__Moraxellaceae;g__Acinetobacter;s__Acinetobacter_sp._TTH0-4
NCBI164851	0	0	0	0	5	156	0	0	0	0	0	0	0	0	0	k__Bacteria;p__;c__;o__;f__;g__;s__uncultured_soil_bacterium
NCBI1648923	0	0	0	0	0	0	0	0	0	0	0	2	0	0	0	k__Bacteria;p__Bacillota;c__Bacilli;o__Bacillales;f__Bacillaceae;g__Bacillus;s__Bacillus_paralicheniformis
NCBI1649877	0	0	0	2	8	1	0	0	0	0	0	0	0	0	0	k__Bacteria;p__Pseudomonadota;c__Gammaproteobacteria;o__Pseudomonadales;f__Pseudomonadaceae;g__Pseudomonas;s__Pseudomonas_sp._CCOS_191
NCBI165179	1	0	6	2	9	119	11	9	4	0	4	231	0	0	0	k__Bacteria;p__Bacteroidota;c__Bacteroidia;o__Bacteroidales;f__Prevotellaceae;g__Prevotella;s__Prevotella_copri
NCBI165186	34	50	31	94	121	228	138	62	35	40	33	37	22	17	0	k__Bacteria;p__Bacillota;c__Clostridia;o__Eubacteriales;f__Oscillospiraceae;g__Ruminococcus;s__uncultured_Ruminococcus_sp.
NCBI165190	0	0	0	0	0	3	0	0	0	0	0	0	0	0	0	k__Bacteria;p__Actinomycetota;c__Coriobacteriia;o__Coriobacteriales;f__Coriobacteriaceae;g__Collinsella;s__uncultured_Collinsella_sp.
NCBI165192	202	178	134	181	367	905	198	79	153	134	77	114	36	48	60	k__Bacteria;p__Bacillota;c__Bacilli;o__Lactobacillales;f__Lactobacillaceae;g__Pediococcus;s__uncultured_Pedio

															coccus_sp.
NCBI1653478	0	0	0	2	0	1	0	0	0	0	0	1	0	0	0	k__Bacteria;p__Actinomycetota;c__Actinomycetes;o__Mycobacteriales;f__Nocardiaceae;g__Rhodococcus;s__Rhodococcus_sp._PBTS_1
NCBI1653480	0	0	0	0	0	7	0	0	0	0	0	0	0	0	0	k__Bacteria;p__Actinomycetota;c__Actinomycetes;o__Pseudonocardiales;f__Pseudonocardiaceae;g__Alloactinosynnema;s__Alloactinosynnema_sp._L-07
NCBI1655	0	0	5	6	13	32	0	13	0	0	1	33	0	0	0	k__Bacteria;p__Actinomycetota;c__Actinomycetes;o__Actinomycetales;f__Actinomycetaceae;g__Actinomyces;s__Actinomyces_naeslundii
NCBI1658665	21	23	9	0	66	996	30	2	11	0	6	7	3	4	1	k__Bacteria;p__Pseudomonadota;c__Betaproteobacteria;o__Burkholderiales;f__Sphaerotilaceae;g__Mitsuaria;s__Mitsuaria_sp._7
NCBI1658672	0	0	0	0	0	10	2	0	0	0	0	0	0	0	0	k__Bacteria;p__Pseudomonadota;c__Betaproteobacteria;o__Burkholderiales;f__Comamonadaceae;g__Ottowia;s__Ottowia_sp._oral_taxon_894
NCBI1660	0	1	0	1	6	26	0	0	1	0	0	123	1	0	0	k__Bacteria;p__Actinomycetota;c__Actinomycetes;o__Actinomycetales;f__Actinomycetaceae;g__Schaalia;s__Schaalia_odontolytica
NCBI166486	0	0	1	2	0	77	6	5	1	0	0	20	0	0	0	k__Bacteria;p__Bacillota;c__Clostridia;o__Eubacteriales;f__Lachnospiraceae;g__Roseburia;s__Roseburia_intestinalis
NCBI1667168	0	3	0	4	0	0	0	0	0	0	0	0	0	0	0	k__Bacteria;p__Actinomycetota;c__Actinomycetes;o__Micrococcales;f__Dermabacteraceae;g__Dermabacter;s__Dermabacter_jinjuensis
NCBI167	0	0	0	0	0	2	0	0	0	0	0	1	0	0	0	k__Bacteria;p__Spirochaetota;c__Spirochaetia;o__Spirochaetales;f__Treponemataceae;g__Treponema;s__Treponema_succinifaciens
NCBI1678128	0	0	0	0	2	4	0	0	0	0	0	0	0	0	0	k__Bacteria;p__Pseudomonadota;c__Betaproteobacteria;o__Burkholderiales;f__Comamonadaceae;g__Limnohabi

															tans;s__Limnohabitans_sp._63ED37-2
NCBI1678129	1	0	2	0	0	3	0	0	0	0	0	0	0	0	0	k__Bacteria;p__Pseudomonadota;c__Betaproteobacteria;o__Burkholderiales;f__Comamonadaceae;g__Limnohabitans;s__Limnohabitans_sp._103DPR2
NCBI1679096	0	0	0	0	0	1	0	0	0	0	0	0	0	0	0	k__Archaea;p__Euryarchaeota;c__Halobacteria;o__Halobacteriales;f__Haloarculaceae;g__Halococcoides;s__Halococcoides_cellulosivorans
NCBI1679497	0	0	0	0	2	16	2	0	0	1	0	0	0	0	0	k__Bacteria;p__Pseudomonadota;c__Alphaproteobacteria;o__Caulobacterales;f__Caulobacteraceae;g__Caulobacter;s__Caulobacter_flavus
NCBI167972	581	555	338	1122	974	2215	769	536	512	508	424	577	113	205	178	k__Bacteria;p__Bacillota;c__Bacilli;o__Lactobacillales;f__Enterococcaceae;g__Enterococcus;s__uncultured_Enterococcus_sp.
NCBI1679721	0	0	0	0	0	1	0	0	0	0	0	0	0	0	0	k__Bacteria;p__Bacillota;c__Clostridia;o__Eubacteriales;f__Lachnospiraceae;g__Herbinix;s__Herbinix_luporum
NCBI167973	178	203	226	394	436	1007	334	229	282	184	318	294	131	120	76	k__Bacteria;p__Bacillota;c__Bacilli;o__Lactobacillales;f__Streptococcaceae;g__Lactococcus;s__uncultured_Lactococcus_sp.
NCBI1680	0	1	1	0	13	1277	2	1	1	0	1	154	0	0	0	k__Bacteria;p__Actinomycetota;c__Actinomycetes;o__Bifidobacteriales;f__Bifidobacteriaceae;g__Bifidobacterium;s__Bifidobacterium_adolescentis
NCBI1681	0	0	1	0	1	440	6	0	3	0	17	46	0	0	0	k__Bacteria;p__Actinomycetota;c__Actinomycetes;o__Bifidobacteriales;f__Bifidobacteriaceae;g__Bifidobacterium;s__Bifidobacterium_bifidum
NCBI1682113	0	0	0	0	15	31	0	0	0	0	0	0	0	0	0	k__Bacteria;p__Actinomycetota;c__Actinomycetes;o__Mycobacteriales;f__Mycobacteriaceae;g__Mycobacterium;s__Mycobacterium_sp._YC-RL4
NCBI1684	0	1	0	2	1	4	0	0	0	0	0	0	0	0	0	k__Bacteria;p__Actinomycetota;c__Actinomycetes;o__Bifidobacteriales;f__Bifidobacteriaceae;g__Bifidobacteriu

															m;s__Bifidobacterium_asteroides
NCBI168471	0	3	0	0	2	128	2	0	0	0	0	0	0	0	0	k__Bacteria;p__Pseudomonadota;c__Betaproteobacteria;o__Neisseriales;f__Chromobacteriaceae;g__Laribacter;s__Laribacter_hongkongensis
NCBI1685	0	0	2	1	6	115	0	1	0	0	4	34	0	0	0	k__Bacteria;p__Actinomycetota;c__Actinomycetes;o__Bifidobacteriales;f__Bifidobacteriaceae;g__Bifidobacterium;s__Bifidobacterium_breve
NCBI1686	0	0	1	0	0	11	0	0	0	0	0	5	0	0	0	k__Bacteria;p__Actinomycetota;c__Actinomycetes;o__Bifidobacteriales;f__Bifidobacteriaceae;g__Bifidobacterium;s__Bifidobacterium_catenulatum
NCBI1686310	2	1	0	3	13	6	1	0	0	1	0	0	0	0	0	k__Bacteria;p__Pseudomonadota;c__Alphaproteobacteria;o__Hyphomicrobiales;f__Bartonellaceae;g__Bartonella;s__Bartonella_apis
NCBI1687	0	0	0	0	0	0	0	0	0	0	0	0	1	0	0	k__Bacteria;p__Actinomycetota;c__Actinomycetes;o__Bifidobacteriales;f__Bifidobacteriaceae;g__Bifidobacterium;s__Bifidobacterium_coryneforme
NCBI1689	1	0	0	0	0	10	0	0	0	0	0	3	0	0	0	k__Bacteria;p__Actinomycetota;c__Actinomycetes;o__Bifidobacteriales;f__Bifidobacteriaceae;g__Bifidobacterium;s__Bifidobacterium_dentium
NCBI1689834	0	0	0	0	0	0	0	0	1	0	0	0	0	0	0	k__Bacteria;p__Pseudomonadota;c__Acidithiobacillia;o__Acidithiobacillales;f__Acidithiobacillaceae;g__Acidithiobacillus;s__Acidithiobacillus_ferriphilus
NCBI169176	1	1	0	0	3	40	1	0	0	0	0	0	0	0	0	k__Bacteria;p__Pseudomonadota;c__Alphaproteobacteria;o__Hyphomicrobiales;f__Hyphomicrobiaceae;g__Caenibius;s__Caenibius_tardaugens
NCBI1691904	0	1	0	0	3	9	0	0	0	0	0	0	0	0	0	k__Bacteria;p__Pseudomonadota;c__Gammaproteobacteria;o__Pseudomonadales;f__Pseudomonadaceae;g__Pseudomonas;s__Pseudomonas_sediminis
NCBI1692238	0	0	0	0	0	0	0	0	0	0	0	6	0	0	0	k__Bacteria;p__Pseudomonadota;c__Gammaproteobacteria;o__Enterobacterales;f__Enterobacteriaceae;g__Entero

																bacter;s__Enterobacter_sp._FY-07
NCBI169283	0	0	0	0	0	14	0	0	0	0	0	29	0	0	0	k__Bacteria;p__Bacillota;c__Bacilli;o__Bacillales;f__Bacillaceae;g__Geobacillus;s__Geobacillus_lituanicus
NCBI169292	0	0	0	0	4	5	0	0	0	0	0	2	0	0	0	k__Bacteria;p__Actinomycetota;c__Actinomycetes;o__Mycobacteriales;f__Corynebacteriaceae;g__Corynebacterium;s__Corynebacterium_aurimucosum
NCBI1694	0	0	1	1	21	23	131	0	19	0	1	54	0	0	0	k__Bacteria;p__Actinomycetota;c__Actinomycetes;o__Bifidobacteriales;f__Bifidobacteriaceae;g__Bifidobacterium;s__Bifidobacterium_pseudolongum
NCBI169480	0	0	0	7	0	9	1	0	0	0	0	1	0	0	0	k__Bacteria;p__Actinomycetota;c__Actinomycetes;o__Micrococcales;f__Micrococcaceae;g__Rothia;s__Rothia_amarae
NCBI169669	0	0	0	12	0	5	0	0	0	0	0	0	0	0	0	k__Bacteria;p__Pseudomonadota;c__Gammaproteobacteria;o__Pseudomonadales;f__Pseudomonadaceae;g__Pseudomonas;s__Pseudomonas_extremorientalis
NCBI169679	0	0	0	1	0	0	0	0	0	0	0	0	0	0	0	k__Bacteria;p__Bacillota;c__Clostridia;o__Eubacteriales;f__Clostridiaceae;g__Clostridium;s__Clostridium_saccharobutylicum
NCBI1697	0	0	0	3	0	1	0	0	0	0	0	2	0	0	0	k__Bacteria;p__Actinomycetota;c__Actinomycetes;o__Mycobacteriales;f__Corynebacteriaceae;g__Corynebacterium;s__Corynebacterium_ammoniagenes
NCBI1699624	0	0	0	0	0	0	0	0	8	0	0	0	0	0	0	k__Bacteria;p__Pseudomonadota;c__Gammaproteobacteria;o__Moraxellales;f__Moraxellaceae;g__Psychrobacter;s__Psychrobacter_sp._P11G5
NCBI1702221	1	0	0	0	0	31	0	0	0	0	0	34	0	0	0	k__Bacteria;p__Bacillota;c__Erysipelotrichia;o__Erysipelotrichales;f__Erysipelotrichaceae;g__Faecalibaculum;s__Faecalibaculum_rodentium
NCBI1702238	0	0	0	12	10	10	19	0	0	0	10	0	0	0	0	k__Bacteria;p__Bacillota;c__Clostridia;o__Eubacteriales;f__Clostridiaceae;g__Clostridium;s__Clostridium_sp._

																MF28
NCBI1702250	0	0	0	0	0	1	0	0	0	0	0	0	0	0	0	k__Bacteria;p__Pseudomonadota;c__Gammaproteobacteria;o__Pseudomonadales;f__Pseudomonadaceae;g__Pseudomonas;s__Pseudomonas_sp._IB20
NCBI1702325	0	0	0	0	0	37	0	0	0	0	0	211	0	0	0	k__Bacteria;p__Pseudomonadota;c__Alphaproteobacteria;o__Hyphomicrobiales;f__Chelatococcaceae;g__Chelatococcus;s__Chelatococcus_sp._CO-6
NCBI1703	2	1	0	0	0	22	0	2	0	0	0	0	0	0	0	k__Bacteria;p__Actinomycetota;c__Actinomycetes;o__Micrococcales;f__Brevibacteriaceae;g__Brevibacterium;s__Brevibacterium_linens
NCBI1703338	17	25	11	0	53	783	22	2	17	0	0	6	0	10	4	k__Bacteria;p__Pseudomonadota;c__Alphaproteobacteria;o__Sphingomonadales;f__Erythrobacteraceae;g__Croceicoccus;s__Croceicoccus_sp._Ery15
NCBI1703340	0	0	0	0	0	5	0	0	0	0	0	0	0	0	0	k__Bacteria;p__Pseudomonadota;c__Alphaproteobacteria;o__Sphingomonadales;f__Erythrobacteraceae;g__Croceicoccus;s__Croceicoccus_sp._Ery5
NCBI1704499	2	1	6	0	10	25	2	0	0	1	0	2	1	0	1	k__Bacteria;p__Pseudomonadota;c__Gammaproteobacteria;o__Methylococcales;f__Methylococcaceae;g__Methylovulum;s__Methylovulum_psychrotolerans
NCBI1705	0	0	0	0	2	4	1	1	0	0	0	30	0	0	0	k__Bacteria;p__Actinomycetota;c__Actinomycetes;o__Mycobacteriales;f__Corynebacteriaceae;g__Corynebacterium;s__Corynebacterium_stationis
NCBI1705310	0	0	0	0	0	0	0	0	0	0	0	3	0	0	0	k__Bacteria;p__Pseudomonadota;c__Betaproteobacteria;o__Burkholderiales;f__Burkholderiaceae;g__Burkholderia;s__Burkholderia_sp._IDO3
NCBI170573	0	0	0	0	1	2	1	0	0	0	0	1	0	0	0	k__Bacteria;p__Bacillota;c__Bacilli;o__Bacillales;f__Staphylococcaceae;g__Staphylococcus;s__Staphylococcus_pettenkoferi

NCBI1707700	0	0	0	0	42	0	0	0	0	0	0	0	0	0	0	k__Fungi;p__Ascomycota;c__Dothideomycetes;o__Cladosporiales;f__Cladosporiaceae;g__Cladosporium;s__Cladosporium_sp.
NCBI1707785	0	0	0	0	18	0	0	0	0	0	0	0	0	0	0	k__Bacteria;p__Pseudomonadota;c__Betaproteobacteria;o__Burkholderiales;f__Oxalobacteraceae;g__Massilia;s__Massilia_sp._WG5
NCBI1708541	1	1	1	9	12	0	1	0	0	0	0	22	0	0	0	k__Fungi;p__Basidiomycota;c__Wallemiomycetes;o__Wallemiales;f__Wallemiaceae;g__Wallemia;s__Wallemia_mellicola
NCBI1710	0	0	0	0	0	34	0	0	0	0	1	0	0	0	0	k__Bacteria;p__Actinomycetota;c__Actinomycetes;o__Micrococcales;f__Promicromonosporaceae;g__Cellulosimicrobium;s__Cellulosimicrobium_cellulans
NCBI1712675	0	0	1	0	0	25	0	0	0	0	0	5	0	0	0	k__Bacteria;p__Bacillota;c__Erysipelotrichia;o__Erysipelotrichales;f__Turicibacteraceae;g__Turicibacter;s__Turicibacter_sp._H121
NCBI1714373	0	0	0	0	0	6	0	0	0	0	0	0	0	0	0	k__Bacteria;p__Actinomycetota;c__Actinomycetes;o__Micrococcales;f__Microbacteriaceae;g__Microbacterium;s__Microbacterium_sp._No._7
NCBI1715720	0	0	0	0	0	1	0	0	0	0	0	0	0	0	0	k__Bacteria;p__Pseudomonadota;c__Betaproteobacteria;o__Burkholderiales;f__Comamonadaceae;g__Diaphorobacter;s__Diaphorobacter_ruginosibacter
NCBI1717	0	0	0	0	24	39	0	0	0	10	0	0	0	0	0	k__Bacteria;p__Actinomycetota;c__Actinomycetes;o__Mycobacteriales;f__Corynebacteriaceae;g__Corynebacterium;s__Corynebacterium_diphtheriae
NCBI1718	0	0	0	26	0	13	0	0	11	0	0	24	0	0	0	k__Bacteria;p__Actinomycetota;c__Actinomycetes;o__Mycobacteriales;f__Corynebacteriaceae;g__Corynebacterium;s__Corynebacterium_glutamicum
NCBI172042	0	7	2	4	1	0	1	0	0	0	0	3	0	1	2	k__Bacteria;p__Actinomycetota;c__Actinomycetes;o__Micrococcales;f__Micrococcaceae;g__Rothia;s__Rothia_aeria

NCBI172043	0	0	0	1	23	34	1	0	6	0	0	1	0	0	0	k__Bacteria;p__Pseudomonadota;c__Alphaproteobacteria;o__Caulobacterales;f__Caulobacteraceae;g__Brevundimonas;s__Brevundimonas_nasdae
NCBI172045	0	0	0	0	0	3	0	0	0	0	0	0	0	0	0	k__Bacteria;p__Bacteroidota;c__Flavobacteriia;o__Flavobacteriales;f__Weeksellaceae;g__Elizabethkingia;s__Elizabethkingia_miricola
NCBI1721	0	0	0	0	2	3	3	0	0	0	0	0	0	0	0	k__Bacteria;p__Actinomycetota;c__Actinomycetes;o__Mycobacteriales;f__Corynebacteriaceae;g__Corynebacterium;s__Corynebacterium_callunae
NCBI1725	5	1	0	7	15	171	0	1	0	0	0	14	0	31	0	k__Bacteria;p__Actinomycetota;c__Actinomycetes;o__Mycobacteriales;f__Corynebacteriaceae;g__Corynebacterium;s__Corynebacterium_xerosis
NCBI1727	3	0	0	1	0	58	0	0	0	0	0	0	0	0	0	k__Bacteria;p__Actinomycetota;c__Actinomycetes;o__Mycobacteriales;f__Corynebacteriaceae;g__Corynebacterium;s__Corynebacterium_variabile
NCBI172827	0	0	1	0	0	2	0	0	0	0	0	0	0	0	0	k__Bacteria;p__Deinococcota;c__Deinococci;o__Thermales;f__Thermaceae;g__Meiothermus;s__Meiothermus_taiwanensis
NCBI173262	341	444	461	777	983	2467	518	428	526	360	242	355	185	229	106	k__Bacteria;p__Bacillota;c__Bacilli;o__Lactobacillales;f__Lactobacillaceae;g__Leuconostoc;s__uncultured_Leuconostoc_sp.
NCBI1735038	0	0	0	0	0	1	0	0	0	0	0	0	0	0	0	k__Bacteria;p__Pseudomonadota;c__Betaproteobacteria;o__Rhodocyclales;f__Rhodocyclaceae;g__Oryzomicrobium;s__Oryzomicrobium_terrae
NCBI173560	0	0	0	0	0	50	0	0	0	0	0	0	0	0	0	k__Bacteria;p__Actinomycetota;c__Actinomycetes;o__Pseudonocardiales;f__Pseudonocardiaceae;g__Saccharothrix;s__Saccharothrix_algeriensis
NCBI1737424	0	0	0	0	0	27	0	0	0	0	0	0	0	0	0	k__Bacteria;p__Bacillota;c__Clostridia;o__Eubacteriales;f__Lachnospiraceae;g__Blautia;s__Blautia_massiliensis

NCBI173971	0	0	0	0	0	90	0	0	0	0	0	0	0	0	0	k__Bacteria;p__Pseudomonadota;c__Gammaproteobacteria;o__Oceanospirillales;f__Halomonadaceae;g__Halomonas;s__uncultured_Halomonas_sp.
NCBI173974	50	41	56	97	104	411	83	44	12	41	43	32	177	49	81	k__Bacteria;p__Pseudomonadota;c__Gammaproteobacteria;o__Alteromonadales;f__Psychromonadaceae;g__Psychromonas;s__uncultured_Psychromonas_sp.
NCBI1741	0	0	0	2	0	0	0	0	0	0	0	0	0	0	0	k__Bacteria;p__Thermodesulfobacteriota;c__Thermodesulfobacteria;o__Thermodesulfobacteriales;f__Thermodesulfobacteriaceae;g__Thermodesulfobacterium;s__Thermodesulfobacterium_commune
NCBI1742359	0	0	0	0	2	0	0	0	0	0	0	0	0	0	0	k__Bacteria;p__Bacillota;c__Bacilli;o__Bacillales;f__Bacillaceae;g__Bacillus;s__Bacillus_dafuensis
NCBI174390	54	58	47	155	222	457	29	63	83	49	64	51	25	16	39	k__Bacteria;p__Spirochaetota;c__Spirochaetia;o__Spirochaetales;f__Spirochaetaceae;g__Spirochaeta;s__uncultured_Spirochaeta_sp.
NCBI1744	0	0	0	1	0	0	0	0	0	0	0	0	0	0	0	k__Bacteria;p__Actinomycetota;c__Actinomycetes;o__Propionibacteriales;f__Propionibacteriaceae;g__Propionibacterium;s__Propionibacterium_freudenreichii
NCBI1745854	0	0	0	0	0	1	0	0	0	0	0	0	0	0	0	k__Bacteria;p__Pseudomonadota;c__Alphaproteobacteria;o__Hyphomicrobiales;f__Xanthobacteraceae;g__Ancylobacter;s__Ancylobacter_pratisalsi
NCBI1747	170	112	130	1249	554	3193	97	228	108	25	93	587	29	32	129	k__Bacteria;p__Actinomycetota;c__Actinomycetes;o__Propionibacteriales;f__Propionibacteriaceae;g__Cutibacterium;s__Cutibacterium_acnes
NCBI1748	0	0	0	0	0	16	0	0	0	0	0	1	0	0	0	k__Bacteria;p__Actinomycetota;c__Actinomycetes;o__Propionibacteriales;f__Propionibacteriaceae;g__Acidipropionibacterium;s__Acidipropionibacterium_acidipropionici
NCBI1750	0	0	0	1	0	1	0	3	0	0	0	5	0	0	0	k__Bacteria;p__Actinomycetota;c__Actinomycetes;o__Propionibacteriales;f__Propionibacteriaceae;g__Arachnia;

																s__Arachnia_propionica
NCBI1752064	0	0	0	0	0	1	0	0	0	0	0	0	0	0	0	k__Bacteria;p__Cyanobacteriota;c__Cyanophyceae;o__Pseudanabaenales;f__Leptolyngbyaceae;g__Leptolyngbya;s__Leptolyngbya_sp._NIES-3755
NCBI1752398	1	1	2	0	6	56	3	0	0	2	0	3	0	0	0	k__Bacteria;p__Pseudomonadota;c__Alphaproteobacteria;o__Hyphomicrobiales;f__Rhizobiaceae;g__Ensifer;s__Ensifer_alkalisoli
NCBI175243	0	0	0	0	0	0	0	4	0	0	0	0	0	0	0	k__Fungi;p__Ascomycota;c__;o__;f__;g__;s__uncultured_Ascomycota
NCBI175245	4116	3539	2817	16263	38468	57251	5828	868	2248	382	278	840	357	281	393	k__Fungi;p__;c__;o__;f__;g__;s__uncultured_fungus
NCBI1755504	0	0	0	0	0	3	2	0	0	0	0	0	0	0	0	k__Bacteria;p__Pseudomonadota;c__Gammaproteobacteria;o__Pseudomonadales;f__Pseudomonadaceae;g__Pseudomonas;s__Pseudomonas_sp._DY-1
NCBI1756988	0	0	0	0	0	4	0	0	0	0	0	0	0	0	0	k__Bacteria;p__Pseudomonadota;c__Alphaproteobacteria;o__Hyphomicrobiales;f__Phyllobacteriaceae;g__Nitratireductor;s__Nitratireductor_sp._OM-1
NCBI1756993	0	0	0	0	0	13	0	0	0	0	10	0	0	11	0	k__Bacteria;p__Pseudomonadota;c__Gammaproteobacteria;o__Enterobacterales;f__Enterobacteriaceae;g__Phytobacter;s__Phytobacter_sp._SCO41
NCBI1758179	0	0	21	0	0	0	0	0	0	0	0	0	0	0	0	k__Bacteria;p__Pseudomonadota;c__Gammaproteobacteria;o__Aeromonadales;f__Aeromonadaceae;g__Aeromonas;s__Aeromonas_sp._ASNIH5
NCBI1758189	0	0	0	0	0	1	0	0	0	0	0	2	0	0	0	k__Bacteria;p__Pseudomonadota;c__Gammaproteobacteria;o__Moraxellales;f__Moraxellaceae;g__Acinetobacter;s__Acinetobacter_sp._ACNIH2
NCBI1758194	0	0	0	0	0	4	0	0	0	0	0	5	0	0	0	k__Bacteria;p__Pseudomonadota;c__Betaproteobacteria;o__Burkholderiales;f__Alcaligenaceae;g__Achromobacter;s__Achromobacter_sp._AONIH1

NCBI1759059	0	0	0	0	0	2	0	0	0	0	0	0	0	0	0	k__Bacteria;p__Pseudomonadota;c__Alphaproteobacteria;o__Hyphomonadales;f__Hyphomonadaceae;g__Candidatus_Viadribacter;s__Candidatus_Viadribacter_manganicus
NCBI1759399	0	0	0	0	0	0	0	0	0	0	0	4	0	0	0	k__Bacteria;p__Bacillota;c__Bacilli;o__Lactobacillales;f__Streptococcaceae;g__Streptococcus;s__Streptococcus_sp._A12
NCBI176275	0	0	0	0	0	2	0	0	0	0	0	0	0	0	0	k__Fungi;p__Ascomycota;c__Sordariomycetes;o__Hypocreales;f__Cordycipitaceae;g__Beauveria;s__Beauveria_bassiana
NCBI1763535	4	3	5	0	18	49	1	0	1	0	0	0	0	0	1	k__Bacteria;p__Pseudomonadota;c__Betaproteobacteria;o__Burkholderiales;f__Comamonadaceae;g__Hydrogenophaga;s__Hydrogenophaga_crassostreae
NCBI1763828	0	0	0	0	0	2	0	0	0	0	0	0	0	0	0	k__Bacteria;p__Pseudomonadota;c__Alphaproteobacteria;o__Sphingomonadales;f__Sphingomonadaceae;g__Hankyongella;s__Hankyongella_ginsenosidimutans
NCBI1763998	0	2	0	0	1	3	1	0	0	0	1	0	0	0	0	k__Bacteria;p__Pseudomonadota;c__Gammaproteobacteria;o__Chromatiales;f__Chromatiaceae;g__Rheinheimera;s__Rheinheimera_sp._F8
NCBI1764	0	0	0	0	0	0	0	0	0	0	0	1	0	0	0	k__Bacteria;p__Actinomycetota;c__Actinomycetes;o__Mycobacteriales;f__Mycobacteriaceae;g__Mycobacterium;s__Mycobacterium_avium
NCBI1765049	362	520	189	10	845	8320	474	23	141	72	64	196	86	23	32	k__Bacteria;p__Pseudomonadota;c__Betaproteobacteria;o__Rhodocyclales;f__Rhodocyclaceae;g__Azospira;s__Azospira_sp._I09
NCBI1765967	1	1	0	0	0	1	0	0	0	0	0	0	0	0	0	k__Bacteria;p__Pseudomonadota;c__Gammaproteobacteria;o__Chromatiales;f__Ectothiorhodospiraceae;g__Acidihalobacter;s__Acidihalobacter_ferrooxydans
NCBI1766	0	0	0	0	0	0	0	0	0	0	0	6	0	0	0	k__Bacteria;p__Actinomycetota;c__Actinomycetes;o__Mycobacteriales;f__Mycobacteriaceae;g__Mycolicibacte

															rium;s__Mycolicibacterium_fortuitum
NCBI1767	0	0	0	0	0	75	0	0	0	0	0	2	0	0	0	k__Bacteria;p__Actinomycetota;c__Actinomycetes;o__Mycobacteriales;f__Mycobacteriaceae;g__Mycobacterium;s__Mycobacterium_intracellulare
NCBI1768	0	0	0	0	0	45	0	0	0	0	0	4	0	0	0	k__Bacteria;p__Actinomycetota;c__Actinomycetes;o__Mycobacteriales;f__Mycobacteriaceae;g__Mycobacterium;s__Mycobacterium_kansasii
NCBI1768108	0	0	0	0	0	2	0	0	0	0	0	0	0	0	0	k__Bacteria;p__Deinococcota;c__Deinococci;o__Deinococcales;f__Deinococcaceae;g__Deinococcus;s__Deinococcus_actinosclerus
NCBI1768242	0	0	0	0	2	35	1	0	0	0	1	0	0	0	0	k__Bacteria;p__Pseudomonadota;c__Betaproteobacteria;o__Burkholderiales;f__;g__Paucibacter;s__Paucibacter_sp._KCTC_42545
NCBI1773	0	0	0	0	0	0	172	0	0	0	0	0	0	0	0	k__Bacteria;p__Actinomycetota;c__Actinomycetes;o__Mycobacteriales;f__Mycobacteriaceae;g__Mycobacterium;s__Mycobacterium_tuberculosis
NCBI1774099	0	0	0	0	2	0	0	0	0	0	0	0	0	0	0	k__Fungi;p__Ascomycota;c__Eurotiomycetes;o__Chaetothyriales;f__Chaetothyriaceae;g__Ceramothyrium;s__Ceramothyrium_longivolcaniforme
NCBI1774216	0	0	5	0	0	0	0	0	0	0	0	6	0	0	0	k__Bacteria;p__Actinomycetota;c__Actinomycetes;o__Propionibacteriales;f__Nocardioidaceae;g__Nocardioides;s__Nocardioides_rotundus
NCBI1775880	0	0	0	1	0	0	0	0	1	0	0	1	0	0	0	k__Bacteria;p__Actinomycetota;c__Actinomycetes;o__Micrococcales;f__Micrococcaceae;g__Glutamicibacter;s__Glutamicibacter_mishrai
NCBI1776742	0	0	1	0	1	8	0	0	0	0	0	2	0	0	0	k__Bacteria;p__Pseudomonadota;c__Gammaproteobacteria;o__Moraxellales;f__Moraxellaceae;g__Acinetobacter;s__Acinetobacter_vivianii
NCBI1776995	0	0	0	0	0	1	0	0	0	0	0	0	0	0	0	k__Bacteria;p__;c__;o__;f__;g__;s__uncultured_bacterium_UPO78

NCBI1778540	0	0	0	1	0	0	0	0	0	0	0	0	0	0	0	k__Bacteria;p__Pseudomonadota;c__Gammaproteobacteria;o__Enterobacterales;f__Pectobacteriaceae;g__Dickeya;s__Dickeya_fangzhongdai
NCBI1778675	0	0	0	0	0	9	0	0	0	0	0	0	0	0	0	k__Bacteria;p__Pseudomonadota;c__Betaproteobacteria;o__Neisseriales;f__Chromobacteriaceae;g__Chromobacterium;s__Chromobacterium_rhizoryzae
NCBI178339	0	0	1	0	0	6	0	0	0	0	0	3	0	0	0	k__Bacteria;p__Actinomycetota;c__Actinomycetes;o__Actinomycetales;f__Actinomycetaceae;g__Pauljensenia;s__Pauljensenia_hongkongensis
NCBI1784719	0	1	0	0	0	126	0	6	0	17	7	2	24	4	39	k__Bacteria;p__Actinomycetota;c__Actinomycetes;o__Micrococcales;f__Microbacteriaceae;g__Leucobacter;s__Leucobacter_triazinivorans
NCBI1785128	0	0	0	0	0	16	0	0	0	0	0	0	0	0	0	k__Bacteria;p__Pseudomonadota;c__Gammaproteobacteria;o__Moraxellales;f__Moraxellaceae;g__Acinetobacter;s__Acinetobacter_lactucae
NCBI1788	0	0	0	0	0	0	0	2	0	0	0	0	0	0	0	k__Bacteria;p__Actinomycetota;c__Actinomycetes;o__Mycobacteriales;f__Mycobacteriaceae;g__Mycolicibacter;s__Mycolicibacter_terrae
NCBI1788301	0	0	0	0	0	1	0	0	0	0	0	0	0	0	0	k__Bacteria;p__Pseudomonadota;c__Gammaproteobacteria;o__Pseudomonadales;f__Pseudomonadaceae;g__Pseudomonas;s__Pseudomonas_versuta
NCBI179111	214	250	172	303	229	726	206	198	285	179	186	130	172	214	263	k__Bacteria;p__Pseudomonadota;c__Gammaproteobacteria;o__Oceanospirillales;f__Oceanospirillaceae;g__Marinomonas;s__uncultured_Marinomonas_sp.
NCBI1792	0	0	3	0	0	0	0	0	0	0	0	0	0	0	0	k__Bacteria;p__Actinomycetota;c__Actinomycetes;o__Mycobacteriales;f__Mycobacteriaceae;g__Mycolicibacterium;s__Mycolicibacterium_chitae
NCBI1792307	0	0	0	0	2	4	0	0	0	0	0	0	0	0	0	k__Bacteria;p__Pseudomonadota;c__Alphaproteobacteria;o__Hyphomicrobiales;f__Boseaceae;g__Bosea;s__Bosea_sp._PAMC_26642

NCBI1794	0	0	0	0	5	0	0	0	0	0	0	0	0	0	0	k__Bacteria;p__Actinomycetota;c__Actinomycetes;o__Mycobacteriales;f__Mycobacteriaceae;g__Mycolicibacterium;s__Mycolicibacterium_gadium
NCBI1795	0	0	0	0	0	3	0	0	0	0	0	0	0	0	0	k__Bacteria;p__Actinomycetota;c__Actinomycetes;o__Mycobacteriales;f__Mycobacteriaceae;g__Mycolicibacterium;s__Mycolicibacterium_neoaurum
NCBI1795053	0	0	0	0	90	195	0	0	0	0	0	0	0	0	0	k__Bacteria;p__Actinomycetota;c__Actinomycetes;o__Micrococcales;f__Microbacteriaceae;g__Microbacterium;s__Microbacterium_sp._PAMC_28756
NCBI1795631	0	0	0	0	1	5	0	0	0	0	0	0	0	0	0	k__Bacteria;p__Pseudomonadota;c__Betaproteobacteria;o__Burkholderiales;f__Comamonadaceae;g__Variovorax;s__Variovorax_sp._PAMC_28711
NCBI179636	190	168	22	0	91	3606	158	0	49	77	0	23	74	0	5	k__Bacteria;p__Pseudomonadota;c__Betaproteobacteria;o__Burkholderiales;f__Comamonadaceae;g__Alicycliphilus;s__Alicycliphilus_denitrificans
NCBI1796606	0	0	0	0	0	27	0	0	0	0	0	0	0	0	0	k__Bacteria;p__Pseudomonadota;c__Betaproteobacteria;o__Burkholderiales;f__Burkholderiaceae;g__Cupriavidus;s__Cupriavidus_nantongensis
NCBI1796613	0	0	0	0	75	2	571	0	68	0	0	8	0	0	12	k__Bacteria;p__Bacteroidota;c__Bacteroidia;o__Bacteroidales;f__Bacteroidaceae;g__Bacteroides;s__Bacteroides_caecimuris
NCBI1796616	0	0	10	0	0	359	3	0	0	0	0	18	0	0	0	k__Bacteria;p__Bacillota;c__Clostridia;o__Eubacteriales;f__Lachnospiraceae;g__Blautia;s__Blautia_pseudococcoides
NCBI1796635	0	1	0	0	0	0	7	0	0	0	0	7	0	0	0	k__Bacteria;p__Bacillota;c__Erysipelotrichia;o__Erysipelotrichales;f__Erysipelotrichaceae;g__Longicatena;s__Longicatena_caecimuris
NCBI1796646	0	0	0	0	137	1	1018	0	104	0	0	12	0	0	2	k__Bacteria;p__Bacteroidota;c__Bacteroidia;o__Bacteroidales;f__Muribaculaceae;g__Muribaculum;s__Muribaculum_intestinale

NCBI1797	0	2	0	0	0	5	0	0	0	0	0	1	0	0	0	k__Bacteria;p__Actinomycetota;c__Actinomycetes;o__Mycobacteriales;f__Mycobacteriaceae;g__Mycolicibacterium;s__Mycolicibacterium_thermoresistibile
NCBI179838	1342	1595	1805	2343	4072	5256	1752	998	1336	570	814	797	154	480	303	k__Bacteria;p__Bacillota;c__Bacilli;o__Lactobacillales;f__Lactobacillaceae;g__Lentilactobacillus;s__Lentilactobacillus_diolivorans
NCBI179879	0	0	0	0	4	6	0	0	0	0	0	2	0	0	0	k__Bacteria;p__Pseudomonadota;c__Betaproteobacteria;o__Burkholderiales;f__Burkholderiaceae;g__Burkholderia;s__Burkholderia_anthina
NCBI180282	0	54	38	217	586	495	0	0	0	0	0	40	0	0	0	k__Bacteria;p__Pseudomonadota;c__Betaproteobacteria;o__Burkholderiales;f__Comamonadaceae;g__Delftia;s__Delftia_tsuruhatensis
NCBI1804	0	0	123	0	0	0	0	0	0	0	0	0	0	0	0	k__Bacteria;p__Actinomycetota;c__Actinomycetes;o__Mycobacteriales;f__Mycobacteriaceae;g__Mycolicibacterium;s__Mycolicibacterium_gilvum
NCBI1804990	0	0	0	0	0	5	0	0	0	0	0	0	0	0	0	k__Bacteria;p__Actinomycetota;c__Actinomycetes;o__Micrococcales;f__Microbacteriaceae;g__Microcella;s__Microcella_flavibacter
NCBI1805933	0	0	5	0	6	20	0	0	0	0	0	0	0	0	0	k__Bacteria;p__Pseudomonadota;c__Gammaproteobacteria;o__Enterobacterales;f__Yersiniaceae;g__Rahnella;s__Rahnella_sp._ERMR1:05
NCBI1809055	0	0	0	0	0	0	0	0	0	0	0	0	0	0	98	k__Bacteria;p__Pseudomonadota;c__Gammaproteobacteria;o__Moraxellales;f__Moraxellaceae;g__Acinetobacter;s__Acinetobacter_sp._DUT-2
NCBI1809410	0	0	0	0	0	1	0	0	0	0	0	0	0	0	0	k__Bacteria;p__Pseudomonadota;c__Betaproteobacteria;o__Burkholderiales;f__Oxalobacteraceae;g__Herminiimonas;s__Herminiimonas_arsenitoxidans
NCBI180957	0	0	0	0	0	2	0	0	0	0	0	0	0	0	0	k__Bacteria;p__Pseudomonadota;c__Gammaproteobacteria;o__Enterobacterales;f__Pectobacteriaceae;g__Pectobacterium;s__Pectobacterium_brasiliense

NCBI1810	0	0	0	0	0	1	0	0	0	0	0	0	0	0	0	k__Bacteria;p__Actinomycetota;c__Actinomycetes;o__Mycobacteriales;f__Mycobacteriaceae;g__Mycolicibacterium;s__Mycolicibacterium_vaccae
NCBI1810504	0	0	0	0	1	1	0	0	0	1	0	0	0	0	0	k__Bacteria;p__Pseudomonadota;c__Gammaproteobacteria;o__Immundisolibacterales;f__Immundisolibacteraceae;g__Immundisolibacter;s__Immundisolibacter_cernigliae
NCBI1810919	0	0	0	0	1	0	0	0	0	0	0	0	0	0	0	k__Fungi;p__Ascomycota;c__Eurotiomycetes;o__Eurotiales;f__Aspergillaceae;g__Aspergillus;s__Aspergillus_mulundensis
NCBI1812935	0	0	0	0	12	0	6	0	0	0	0	0	0	0	8	k__Bacteria;p__Pseudomonadota;c__Gammaproteobacteria;o__Enterobacterales;f__Enterobacteriaceae;g__Enterobacter;s__Enterobacter_roggenkampii
NCBI1813182	0	0	0	0	0	0	0	0	0	0	0	11	0	0	0	k__Bacteria;p__Bacillota;c__Bacilli;o__Bacillales;f__Bacillaceae;g__Geobacillus;s__Geobacillus_sp._JS12
NCBI1813451	8	7	4	1	35	524	21	0	5	3	2	3	1	0	4	k__Bacteria;p__Pseudomonadota;c__Alphaproteobacteria;o__Hyphomicrobiales;f__Rhizobiaceae;g__Peteryoungia;s__Peteryoungia_desertarenae
NCBI1813611	0	0	0	3	11	0	0	0	0	0	0	0	0	0	0	k__Bacteria;p__Bacteroidota;c__Flavobacteriia;o__Flavobacteriales;f__Weeksellaceae;g__Chryseobacterium;s__Chryseobacterium_cucumeris
NCBI181487	0	0	0	0	0	0	0	0	0	0	0	0	0	1	0	k__Bacteria;p__Actinomycetota;c__Actinomycetes;o__Actinomycetales;f__Actinomycetaceae;g__Schaalia;s__Schaalia_cardiffensis
NCBI1815975	0	0	0	0	0	3	0	0	0	0	0	0	0	0	0	k__Heunggongvirae;p__Uroviricota;c__Caudoviricetes;o__;f__;g__Jamesmcgillvirus;s__Pseudomonas_phage_PaMx35
NCBI1817405	0	0	0	0	3	0	0	0	0	0	0	0	0	0	0	k__Bacteria;p__Bacillota;c__Bacilli;o__Bacillales;f__Staphylococcaceae;g__Abyssicoccus;s__Abyssicoccus_albus

NCBI1817965	0	0	0	0	0	6	0	0	0	0	0	0	0	0	0	k__Bacteria;p__Pseudomonadota;c__Alphaproteobacteria;o__Rhodospirillales;f__Azospirillaceae;g__Skermanella;s__Skermanella_rosea
NCBI182096	0	0	0	0	0	1	0	0	0	0	0	1	0	0	0	k__Fungi;p__Ascomycota;c__Eurotiomycetes;o__Eurotiales;f__Aspergillaceae;g__Aspergillus;s__Aspergillus_chevalieri
NCBI1821625	0	0	0	0	0	0	0	0	5	0	0	0	0	0	0	k__Bacteria;p__Actinomycetota;c__Actinomycetes;o__Kitasatosporales;f__Streptomycetaceae;g__Streptomyces;s__Streptomyces_sp._VN1
NCBI182262	0	0	0	0	0	0	0	0	0	0	0	5	0	0	0	k__Bacteria;p__Pseudomonadota;c__Gammaproteobacteria;o__Alteromonadales;f__Alteromonadaceae;g__Agarivorans;s__Agarivorans_albus
NCBI1824	0	0	0	0	15	8	0	0	0	0	0	0	0	0	0	k__Bacteria;p__Actinomycetota;c__Actinomycetes;o__Mycobacteriales;f__Nocardiaceae;g__Nocardia;s__Nocardia_asteroides
NCBI1825976	0	0	0	0	1	5	0	0	0	0	0	0	0	0	0	k__Bacteria;p__Pseudomonadota;c__Alphaproteobacteria;o__Hyphomicrobiales;f__Rhizobiaceae;g__Neorhizobium;s__Neorhizobium_sp._NCHU2750
NCBI182640	0	1	0	0	0	269	0	0	0	0	0	0	0	0	0	k__Bacteria;p__Actinomycetota;c__Actinomycetes;o__Propionibacteriales;f__Kribbellaceae;g__Kribbella;s__Kribbella_flavida
NCBI1826607	0	0	0	0	0	3	0	0	0	0	0	0	0	0	0	k__Bacteria;p__Pseudomonadota;c__Alphaproteobacteria;o__Rhodobacterales;f__Paracoccaceae;g__Silicimonas;s__Silicimonas_algicola
NCBI182710	0	0	0	0	0	1	0	0	8	0	0	0	0	0	0	k__Bacteria;p__Bacillota;c__Bacilli;o__Bacillales;f__Bacillaceae;g__Oceanobacillus;s__Oceanobacillus_iheyensis
NCBI1827285	20	25	3	33	57	25	21	2	10	9	2	46	2	6	0	k__Bacteria;p__Pseudomonadota;c__Gammaproteobacteria;o__Moraxellales;f__Moraxellaceae;g__Acinetobacter;s__Acinetobacter_sp._MYb10

NCBI1827469	20	17	6	2	57	653	34	6	2	0	0	8	0	0	0	k__Bacteria;p__Pseudomonadota;c__Alphaproteobacteria;o__Caulobacterales;f__Caulobacteraceae;g__Brevundimonas;s__Brevundimonas_sp._GW460-12-10-14-LB2
NCBI1827580	0	0	0	0	0	0	0	0	0	0	0	0	0	0	1	k__Bacteria;p__Actinomycetota;c__Actinomycetes;o__Kitasatosporales;f__Streptomycetaceae;g__Streptomyces;s__Streptomyces_nigra
NCBI1828	1	1	0	2	1	17	1	2	1	1	1	7	0	0	1	k__Bacteria;p__Actinomycetota;c__Actinomycetes;o__Mycobacteriales;f__Nocardiaceae;g__Rhodococcus;s__Rhodococcus_fascians
NCBI1829	0	3	0	0	0	4	0	0	0	0	0	0	0	0	0	k__Bacteria;p__Actinomycetota;c__Actinomycetes;o__Mycobacteriales;f__Nocardiaceae;g__Rhodococcus;s__Rhodococcus_rhodochrous
NCBI1830	0	0	0	1	0	6	3	0	0	0	0	3	0	0	0	k__Bacteria;p__Actinomycetota;c__Actinomycetes;o__Mycobacteriales;f__Nocardiaceae;g__Rhodococcus;s__Rhodococcus_ruber
NCBI1833	0	0	0	4	0	0	0	0	0	0	0	0	0	0	0	k__Bacteria;p__Actinomycetota;c__Actinomycetes;o__Mycobacteriales;f__Nocardiaceae;g__Rhodococcus;s__Rhodococcus_erythropolis
NCBI1835702	0	0	0	0	0	1	0	0	0	0	0	0	0	0	0	k__Fungi;p__Ascomycota;c__Eurotiomycetes;o__Eurotiales;f__Aspergillaceae;g__Penicillium;s__Penicillium_arizonense
NCBI1836	0	0	0	2	4	0	0	0	0	0	0	0	0	0	0	k__Bacteria;p__Actinomycetota;c__Actinomycetes;o__Pseudonocardiales;f__Pseudonocardiaceae;g__Saccharopolyspora;s__Saccharopolyspora_erythraea
NCBI1839799	0	0	0	0	0	5	0	0	0	0	0	0	0	0	0	k__Bacteria;p__Bacillota;c__Bacilli;o__Lactobacillales;f__Streptococcaceae;g__Streptococcus;s__Streptococcus_sp._FDAARGOS_192
NCBI1841863	0	0	0	0	0	1	0	0	0	0	0	0	0	0	0	k__Bacteria;p__Actinomycetota;c__Coriobacteriia;o__Eggerthellales;f__Eggerthellaceae;g__Gordonibacter;s__Gordonibacter_massiliensis

NCBI1842533	6	0	0	0	9	151	3	0	0	2	0	0	0	7	0	k__Bacteria;p__Pseudomonadota;c__Betaproteobacteria;o__Burkholderiales;f__Comamonadaceae;g__Acidovorax;s__Acidovorax_sp._RAC01
NCBI1842534	1	0	0	0	0	15	0	0	0	1	0	0	0	0	0	k__Bacteria;p__Pseudomonadota;c__Alphaproteobacteria;o__Hyphomicrobiales;f__Rhizobiaceae;g__Sinorhizobium;s__Sinorhizobium_sp._RAC02
NCBI1842535	8	5	4	0	31	276	11	0	1	0	0	2	1	1	0	k__Bacteria;p__Pseudomonadota;c__Alphaproteobacteria;o__Sphingomonadales;f__Sphingomonadaceae;g__Blastomonas;s__Blastomonas_sp._RAC04
NCBI1842536	1	3	0	0	22	201	3	1	3	0	0	0	1	0	0	k__Bacteria;p__Pseudomonadota;c__Alphaproteobacteria;o__Hyphomicrobiales;f__Rhizobiaceae;g__Agrobacterium;s__Agrobacterium_sp._RAC06
NCBI1842537	0	4	0	0	0	8	0	0	1	0	0	0	0	0	0	k__Bacteria;p__Pseudomonadota;c__Betaproteobacteria;o__Burkholderiales;f__Comamonadaceae;g__Hydrogenophaga;s__Hydrogenophaga_sp._RAC07
NCBI1842539	33	37	44	7	83	1034	32	0	20	9	7	10	6	6	0	k__Bacteria;p__Pseudomonadota;c__Alphaproteobacteria;o__Hyphomicrobiales;f__Boseaceae;g__Bosea;s__Bosea_sp._RAC05
NCBI1842540	0	0	1	2	8	5	0	0	1	2	0	0	0	0	0	k__Bacteria;p__Pseudomonadota;c__Betaproteobacteria;o__Nitrosomonadales;f__Sterolibacteriaceae;g__Methyloversatilis;s__Methyloversatilis_sp._RAC08
NCBI1842727	0	0	0	2	1	13	0	0	1	0	0	0	0	0	0	k__Bacteria;p__Pseudomonadota;c__Betaproteobacteria;o__Burkholderiales;f__Comamonadaceae;g__Rhodoferax;s__Rhodoferax_koreense
NCBI1843368	7	1	13	0	21	202	11	1	0	6	3	4	1	1	3	k__Bacteria;p__Pseudomonadota;c__Alphaproteobacteria;o__Sphingomonadales;f__Sphingomonadaceae;g__Sphingobium;s__Sphingobium_sp._RAC03
NCBI1844999	0	0	0	0	0	0	47	0	0	0	0	0	0	0	0	k__Bacteria;p__Bacillota;c__Bacilli;o__Bacillales;f__Listeriaceae;g__Listeria;s__Listeria_sp._PSOL-1

NCBI1849015	0	0	0	0	2	0	0	0	0	0	0	0	0	0	k__Bacteria;p__Campylobacterota;c__Epsilonproteobacteria;o__Campylobacterales;f__Arcobacteraceae;g__Arcobacter;s__Arcobacter_acticola
NCBI1849032	0	0	0	0	0	0	0	0	6	0	0	0	0	0	0	k__Bacteria;p__Actinomycetota;c__Actinomycetes;o__Micrococcales;f__Micrococcaceae;g__Arthrobacter;s__Arthrobacter_sp._U41
NCBI184914	0	0	0	0	0	47	0	0	0	0	0	0	0	0	0	k__Bacteria;p__Myxococcota;c__Myxococcia;o__Myxococcales;f__Myxococcaceae;g__Corallococcus;s__Corallococcus_coralloides
NCBI1849154	0	23	0	30	0	0	0	0	0	0	0	0	0	0	0	k__Fungi;p__Ascomycota;c__Saccharomycetes;o__Saccharomycetales;f__Phaffomycetaceae;g__Cyberlindnera;s__Cyberlindnera_sp._RODW5
NCBI1849986	0	0	0	8	5	15	6	0	0	0	0	0	0	0	0	k__Bacteria;p__Pseudomonadota;c__Gammaproteobacteria;o__Pseudomonadales;f__Pseudomonadaceae;g__Pseudomonas;s__Pseudomonas_sp._JNBP_4382
NCBI1850238	4	6	0	0	26	152	7	0	1	1	0	0	2	0	0	k__Bacteria;p__Pseudomonadota;c__Alphaproteobacteria;o__Sphingomonadales;f__Sphingomonadaceae;g__Rhizorhabdus;s__Rhizorhabdus_dicambivorans
NCBI1850250	0	0	0	0	0	8	0	0	0	0	0	0	0	0	0	k__Bacteria;p__Pseudomonadota;c__Alphaproteobacteria;o__Rhodobacterales;f__Paracoccaceae;g__Rhodobacter;s__Rhodobacter_sp._LPB0142
NCBI1850374	0	0	0	0	0	1	0	0	0	0	0	0	0	0	0	k__Bacteria;p__Pseudomonadota;c__Alphaproteobacteria;o__Hyphomicrobiales;f__Xanthobacteraceae;g__Ancylobacter;s__Ancylobacter_sp._TS-1
NCBI1851395	0	0	0	0	0	3	0	0	0	0	0	0	0	0	0	k__Bacteria;p__Actinomycetota;c__Actinomycetes;o__Actinomycetales;f__Actinomycetaceae;g__Actinomyces;s__Actinomyces_sp._Chiba101
NCBI1851514	0	0	0	0	0	0	1	0	0	0	0	0	0	0	0	k__Bacteria;p__Pseudomonadota;c__Gammaproteobacteria;o__Enterobacterales;f__Enterobacteriaceae;g__Scandinavium;s__Scandinavium_goeteborgense

NCBI1852	0	0	0	0	18	6	0	0	0	0	0	2	0	0	0	k__Bacteria;p__Actinomycetota;c__Actinomycetes;o__Pseudonocardiales;f__Pseudonocardiaceae;g__Saccharomonospora;s__Saccharomonospora_viridis
NCBI1852374	0	0	2	0	0	0	0	0	0	0	0	0	0	0	0	k__Bacteria;p__Bacillota;c__Tissierellia;o__;f__;g__Ezakiella;s__Ezakiella_massiliensis
NCBI1852377	0	0	0	0	0	0	0	0	0	0	0	16	0	0	0	k__Bacteria;p__Actinomycetota;c__Actinomycetes;o__Actinomycetales;f__Actinomycetaceae;g__Actinomyces;s__Actinomyces_pacaensis
NCBI1853130	0	0	0	0	0	0	0	0	0	0	0	1	0	0	0	k__Bacteria;p__Pseudomonadota;c__Gammaproteobacteria;o__Pseudomonadales;f__Pseudomonadaceae;g__Pseudomonas;s__Pseudomonas_silesiensis
NCBI1853487	0	0	0	0	0	67	0	0	0	0	0	15	0	0	0	k__Bacteria;p__Pseudomonadota;c__Alphaproteobacteria;o__Hyphomicrobiales;f__Rhizobiaceae;g__Rhizobium;s__Rhizobium_sp._OTB26
NCBI1854574	0	0	0	0	0	5	0	0	0	0	0	0	0	0	0	k__Bacteria;p__Actinomycetota;c__Actinomycetes;o__Kitasatosporales;f__Streptomycetaceae;g__Streptomyces;s__Streptomyces_huasconensis
NCBI1855331	12	2	7	41	60	167	14	1	6	0	0	14	2	0	0	k__Bacteria;p__Pseudomonadota;c__Gammaproteobacteria;o__Pseudomonadales;f__Pseudomonadaceae;g__Pseudomonas;s__Pseudomonas_sp._A214
NCBI1855519	0	0	0	0	1	14	0	0	0	0	0	0	0	0	0	k__Bacteria;p__Pseudomonadota;c__Alphaproteobacteria;o__Sphingomonadales;f__Sphingomonadaceae;g__Sphingobium;s__Sphingobium_sp._EP60837
NCBI1855875	0	0	0	0	0	0	0	0	0	0	0	0	0	1	0	k__Bacteria;p__Pseudomonadota;c__Gammaproteobacteria;o__Chromatiales;f__Ectothiorhodospiraceae;g__Spiribacter;s__Spiribacter_roseus
NCBI1855912	0	0	0	0	1	0	0	0	0	0	0	0	0	0	0	k__Bacteria;p__Acidobacteriota;c__Vicinamibacteria;o__Vicinamibacterales;f__Vicinamibacteraceae;g__Luteitalea;s__Luteitalea_pratensis

NCBI1856685	1	0	1	0	0	3	3	0	0	0	0	0	0	0	0	k__Bacteria;p__Pseudomonadota;c__Gammaproteobacteria;o__Pseudomonadales;f__Pseudomonadaceae;g__Pseudomonas;s__Pseudomonas_sp._TCU-HL1
NCBI1858609	34	21	20	23	57	789	35	8	0	13	0	21	0	0	40	k__Bacteria;p__Pseudomonadota;c__Betaproteobacteria;o__Burkholderiales;f__Comamonadaceae;g__Acidovorax;s__Acidovorax_sp._T1
NCBI1861	0	0	0	0	0	7703	0	0	0	0	0	0	0	0	0	k__Bacteria;p__Actinomycetota;c__Actinomycetes;o__Geodermatophilales;f__Geodermatophilaceae;g__Geodermatophilus;s__Geodermatophilus_obscurus
NCBI1866325	0	0	8	0	0	60	0	0	0	0	0	0	0	0	0	k__Bacteria;p__Pseudomonadota;c__Alphaproteobacteria;o__Sphingomonadales;f__Sphingomonadaceae;g__Sphingopyxis;s__Sphingopyxis_sp._MG
NCBI1867715	0	1	0	0	1	4	0	0	0	0	0	0	0	0	0	k__Bacteria;p__Pseudomonadota;c__Alphaproteobacteria;o__Hyphomicrobiales;f__Boseaceae;g__Bosea;s__Bosea_sp._Tri-49
NCBI1867719	0	0	0	0	0	1	0	0	0	0	0	0	0	0	0	k__Bacteria;p__Pseudomonadota;c__Alphaproteobacteria;o__Hyphomicrobiales;f__Phyllobacteriaceae;g__Phyllobacterium;s__Phyllobacterium_zundukense
NCBI1868589	0	0	0	0	0	4	0	0	0	0	0	0	0	0	0	k__Bacteria;p__Pseudomonadota;c__Alphaproteobacteria;o__Hyphomicrobiales;f__Phreatobacteraceae;g__Phreatobacter;s__Phreatobacter_cathodiphilus
NCBI1868793	1	0	1	0	0	2	0	0	3	0	0	0	0	0	0	k__Bacteria;p__Bacillota;c__Bacilli;o__Lactobacillales;f__Carnobacteriaceae;g__Jeotgalibaca;s__Jeotgalibaca_porci
NCBI1869170	0	753	0	0	0	2471	497	0	0	0	0	0	0	0	0	k__Bacteria;p__Pseudomonadota;c__Alphaproteobacteria;o__Hyphomicrobiales;f__Rhizobiaceae;g__Rhizobium;s__Rhizobium_sp._S41
NCBI1869227	0	3	0	0	4	27	0	0	0	0	0	4	0	0	0	k__Bacteria;p__;c__;o__;f__;g__;s__bacterium

NCBI1870984	0	0	0	0	0	0	0	0	0	0	0	1	0	0	0	k__Bacteria;p__Bacillota;c__Tissierellia;o__Tissierellales;f__Peptoniphilaceae;g__Anaerococcus;s__Anaerococcus_mediterraneensis
NCBI1871021	0	0	0	0	1	5	1	0	0	0	0	1	0	0	0	k__Bacteria;p__Bacillota;c__Clostridia;o__Eubacteriales;f__Lachnospiraceae;g__Lachnoclostridium;s__Lachnoclostridium_phocaeense
NCBI1871022	0	0	0	0	0	3	0	0	0	0	0	0	0	0	0	k__Bacteria;p__Actinomycetota;c__Coriobacteriia;o__Coriobacteriales;f__Atopobiaceae;g__Parolsenella;s__Parolsenella_massiliensis
NCBI1871086	0	0	0	0	2	3	0	0	0	0	0	3	0	0	0	k__Bacteria;p__Pseudomonadota;c__Alphaproteobacteria;o__Caulobacterales;f__Caulobacteraceae;g__Brevundimonas;s__Brevundimonas_sp.
NCBI187304	0	8	0	0	10	105	4	0	0	0	0	0	0	0	0	k__Bacteria;p__Pseudomonadota;c__Alphaproteobacteria;o__Hyphomicrobiales;f__Stappiaceae;g__Roseibium;s__Roseibium_aggregatum
NCBI187327	1	0	0	0	0	1	0	0	0	0	0	1	0	0	0	k__Bacteria;p__Bacillota;c__Negativicutes;o__Acidaminococcales;f__Acidaminococcaceae;g__Acidaminococcus;s__Acidaminococcus_intestini
NCBI1873497	60	0	0	173	82	127	0	0	0	0	0	0	0	0	0	k__Bacteria;p__Pseudomonadota;c__Gammaproteobacteria;o__Enterobacterales;f__Yersiniaceae;g__Rahnella;s__Rahnella_sp.
NCBI1873960	0	0	0	0	0	0	3	0	0	0	0	0	0	0	0	k__Fungi;p__Ascomycota;c__Dothideomycetes;o__Mycosphaerellales;f__Mycosphaerellaceae;g__Pseudocercospora;s__Pseudocercospora_fijiensis
NCBI187400	0	2	0	0	3	26	0	0	0	0	0	0	0	0	0	k__Bacteria;p__Pseudomonadota;c__Alphaproteobacteria;o__Rhodobacterales;f__Paracoccaceae;g__Paracoccus;s__Paracoccus_zeaxanthinifaciens
NCBI1874061	3	1	1	0	16	132	5	0	5	0	0	0	0	1	4	k__Bacteria;p__Pseudomonadota;c__Alphaproteobacteria;o__Sphingomonadales;f__Sphingomonadaceae;g__Sphingopyxis;s__Sphingopyxis_sp._EG6

NCBI187452	1842	1872	1450	3477	3394	550	1492	2100	2318	2047	1835	2485	838	709	1029	k__Bacteria;p__Bacillota;c__Bacilli;o__Lactobacillales;f__Lactobacillaceae;g__Pediococcus;s__Pediococcus_claussenii
NCBI187493	0	0	0	0	0	1	0	0	0	0	0	0	0	0	0	k__Bacteria;p__Pseudomonadota;c__Gammaproteobacteria;o__Oceanospirillales;f__Oceanospirillaceae;g__Thalassolituus;s__Thalassolituus_oleivorans
NCBI1876758	595	404	509	1728	2152	2879	897	323	642	2350	2046	3766	3747	4226	4845	k__Bacteria;p__Pseudomonadota;c__Alphaproteobacteria;o__Rhodospirillales;f__Acetobacteraceae;g__Gluconobacter;s__Gluconobacter_sp.
NCBI1877	0	0	0	0	0	176	2	0	0	0	0	0	0	0	0	k__Bacteria;p__Actinomycetota;c__Actinomycetes;o__Micromonosporales;f__Micromonosporaceae;g__Micromonospora;s__Micromonospora_echinospora
NCBI1879050	0	0	0	0	0	0	0	0	0	1	0	4	0	0	0	k__Bacteria;p__Pseudomonadota;c__Gammaproteobacteria;o__Moraxellales;f__Moraxellaceae;g__Acinetobacter;s__Acinetobacter_wuhouensis
NCBI1881016	0	0	0	0	0	1	0	0	0	0	0	0	0	0	0	k__Bacteria;p__Pseudomonadota;c__Betaproteobacteria;o__Burkholderiales;f__Alcaligenaceae;g__Achromobacter;s__Achromobacter_sp._MFA1_R4
NCBI1882222	0	0	0	0	0	5	5	0	0	0	0	0	0	0	0	k__Bacteria;p__Pseudomonadota;c__Alphaproteobacteria;o__Hyphomicrobiales;f__Brucellaceae;g__Ochrobactrum;s__Ochrobactrum_sp._PW1
NCBI1882682	0	0	0	0	1	86	0	0	0	0	0	1	0	0	0	k__Bacteria;p__Pseudomonadota;c__Alphaproteobacteria;o__Hyphomicrobiales;f__Methylobacteriaceae;g__Microvirga;s__Microvirga_ossetica
NCBI1882747	0	0	0	2	0	54	0	0	0	1	1	0	3	0	13	k__Bacteria;p__Pseudomonadota;c__Alphaproteobacteria;o__Hyphomicrobiales;f__Nitrobacteraceae;g__Afipia;s__Afipia_sp._GAS231
NCBI1882749	0	0	0	0	0	4	0	0	0	0	0	0	0	0	0	k__Bacteria;p__Verrucomicrobiota;c__Opitutae;o__Opitutales;f__Opitutaceae;g__Opitutus;s__Opitutus_sp._GAS368

NCBI1884907	1	0	0	0	25	0	1	0	0	0	0	0	0	0	8	k__Bacteria;p__Actinomycetota;c__Actinomycetes;o__Candidatus_Nanopelagicales;f__Candidatus_Nanopelagicaceae;g__Candidatus_Planktophila;s__Candidatus_Planktophila_vernalis
NCBI1884913	0	0	0	3	0	0	0	0	0	0	0	0	0	0	0	k__Bacteria;p__Actinomycetota;c__Actinomycetes;o__Candidatus_Nanopelagicales;f__Candidatus_Nanopelagicaceae;g__Candidatus_Planktophila;s__Candidatus_Planktophila_lacus
NCBI1888	0	0	0	0	0	4	0	0	0	0	0	0	0	2	0	k__Bacteria;p__Actinomycetota;c__Actinomycetes;o__Kitasatosporales;f__Streptomycetaceae;g__Streptomyces;s__Streptomyces_albus
NCBI1890302	0	0	0	0	0	4	0	0	0	0	4	0	0	0	0	k__Bacteria;p__Bacillota;c__Bacilli;o__Bacillales;f__Bacillaceae;g__Bacillus;s__Bacillus_wiedmannii
NCBI1891238	0	0	0	0	0	1	0	0	0	0	0	0	0	0	0	k__Bacteria;p__Pseudomonadota;c__Betaproteobacteria;o__Burkholderiales;f__;g__;s__Burkholderiales_bacterium
NCBI1891279	0	0	0	0	1	0	0	0	0	0	0	0	0	0	0	k__Bacteria;p__Pseudomonadota;c__Alphaproteobacteria;o__Rhodospirillales;f__Thalassospiraceae;g__Thalassospira;s__Thalassospira_indica
NCBI1891767	0	0	0	0	0	0	2	0	0	0	0	0	0	0	0	k__Shotokuvirae;p__Cossaviricota;c__Papovaviricetes;o__Sepolyvirales;f__Polyomaviridae;g__Betapolyomavirus;s__Betapolyomavirus_macacae
NCBI1892	0	0	0	6	5	0	6	6	0	12	14	53	26	29	32	k__Bacteria;p__Actinomycetota;c__Actinomycetes;o__Kitasatosporales;f__Streptomycetaceae;g__Streptomyces;s__Streptomyces_anulatus
NCBI1892855	0	0	0	0	0	2	0	0	0	0	0	0	0	0	0	k__Bacteria;p__Pseudomonadota;c__Alphaproteobacteria;o__Sphingomonadales;f__Sphingosinicellaceae;g__Sphingosinicella;s__Sphingosinicella_sp._BN140058
NCBI189426	1	0	0	0	0	1	0	0	0	0	0	0	0	0	0	k__Bacteria;p__Bacillota;c__Bacilli;o__Bacillales;f__Paenibacillaceae;g__Paenibacillus;s__Paenibacillus_odorife

															r
NCBI1896164	0	0	0	0	0	0	0	0	0	0	0	2	0	0	0	k__Bacteria;p__Pseudomonadota;c__Gammaproteobacteria;o__Xanthomonadales;f__Xanthomonadaceae;g__Luteimonas;s__Luteimonas_sp._JM171
NCBI1896196	5	2	4	0	10	128	4	0	0	2	0	1	0	2	1	k__Bacteria;p__Pseudomonadota;c__Alphaproteobacteria;o__Sphingomonadales;f__Erythrobacteraceae;g__Porphyrobacter;s__Porphyrobacter_sp._LM_6
NCBI189668	325	204	144	49	60	142	149	326	283	383	290	414	197	195	199	k__Bacteria;p__Bacillota;c__Bacilli;o__Bacillales;f__Staphylococcaceae;g__Staphylococcus;s__uncultured_Staphylococcus_sp.
NCBI1897061	0	0	0	0	0	3	0	0	0	0	0	0	0	0	0	k__Bacteria;p__Actinomycetota;c__Actinomycetes;o__Micrococcales;f__Microbacteriaceae;g__Cryobacterium;s__Cryobacterium_sp._SO1
NCBI1898103	0	19	0	0	0	213	0	0	0	0	0	0	0	0	0	k__Bacteria;p__Pseudomonadota;c__Betaproteobacteria;o__Rhodocyclales;f__Rhodocyclaceae;g__;s__Rhodocyclaceae_bacterium
NCBI1898104	0	0	0	0	0	1	0	0	0	1	0	0	0	0	0	k__Bacteria;p__Bacteroidota;c__;o__;f__;g__;s__Bacteroidota_bacterium
NCBI1898203	0	0	0	0	0	19	0	0	0	0	0	49	0	0	0	k__Bacteria;p__Bacillota;c__Clostridia;o__Eubacteriales;f__Lachnospiraceae;g__;s__Lachnospiraceae_bacterium
NCBI1898207	0	0	0	2	3	21	0	1	0	0	0	14	0	0	1	k__Bacteria;p__Bacillota;c__Clostridia;o__Eubacteriales;f__;g__;s__Clostridiales_bacterium
NCBI189834	0	0	0	0	0	1	0	0	0	0	0	0	0	0	0	k__Bacteria;p__Pseudomonadota;c__Gammaproteobacteria;o__Pasteurellales;f__Pasteurellaceae;g__Actinobacillus;s__Actinobacillus_porcitonsillarum
NCBI1898684	0	0	0	0	0	36	3	0	0	0	0	0	0	0	0	k__Bacteria;p__Pseudomonadota;c__Gammaproteobacteria;o__Pseudomonadales;f__Pseudomonadaceae;g__Pseudomonas;s__Pseudomonas_sp._LPH1

NCBI190148	0	0	0	0	1	5	0	0	0	0	0	0	0	0	0	k__Bacteria;p__Pseudomonadota;c__Alphaproteobacteria;o__Hyphomicrobiales;f__Nitrobacteraceae;g__Bradyrhizobium;s__Bradyrhizobium_paxllaeri
NCBI1902136	0	0	0	0	0	6	0	2	0	0	0	4	0	0	0	k__Bacteria;p__Bacillota;c__Bacilli;o__Lactobacillales;f__Streptococcaceae;g__Streptococcus;s__Streptococcus_sp._NPS_308
NCBI1903058	0	0	0	0	1	5	0	0	0	0	0	0	1	0	1	k__Bacteria;p__Verrucomicrobiota;c__Spartobacteria;o__;f__;g__Candidatus_Xiphinematobacter;s__Candidatus_Xiphinematobacter_sp.
NCBI1903186	0	0	0	3	0	0	0	0	0	0	0	2	0	0	0	k__Bacteria;p__Actinomycetota;c__Actinomycetes;o__Micrococcales;f__Dermabacteraceae;g__Brachybacterium;s__Brachybacterium_sp._P6-10-X1
NCBI1904441	0	0	0	0	0	6	0	0	0	0	0	0	0	0	0	k__Bacteria;p__Pseudomonadota;c__Alphaproteobacteria;o__Rhodobacterales;f__Paracoccaceae;g__;s__Paracoccaceae_bacterium
NCBI1904944	1	2	4	14	11	34	4	2	6	0	0	4	0	1	0	k__Bacteria;p__Pseudomonadota;c__Gammaproteobacteria;o__Xanthomonadales;f__Xanthomonadaceae;g__Stenotrophomonas;s__Stenotrophomonas_sp._LM091
NCBI1905847	0	0	0	1	3	2	1	0	0	0	0	0	0	0	0	k__Bacteria;p__Actinomycetota;c__Actinomycetes;o__Micrococcales;f__Microbacteriaceae;g__Curtobacterium;s__Curtobacterium_sp._BH-2-1-1
NCBI1906	0	0	0	0	0	41	0	0	0	0	0	0	0	0	0	k__Bacteria;p__Actinomycetota;c__Actinomycetes;o__Kitasatosporales;f__Streptomycetaceae;g__Streptomyces;s__Streptomyces_fradiae
NCBI1906272	0	0	0	0	0	0	0	0	0	0	0	1	0	0	0	k__Bacteria;p__Bacillota;c__Bacilli;o__Bacillales;f__Paenibacillaceae;g__Paenibacillus;s__Paenibacillus_sp._JZ16
NCBI1906273	0	0	0	0	0	57	0	0	0	0	0	0	0	0	0	k__Bacteria;p__Actinomycetota;c__Actinomycetes;o__Micrococcales;f__Promicromonosporaceae;g__Cellulosimicrobium;s__Cellulosimicrobium_sp._JZ28

NCBI1906274	0	0	0	0	0	0	0	0	0	0	0	1	0	0	0	k__Bacteria;p__Actinomycetota;c__Actinomycetes;o__Micrococcales;f__Microbacteriaceae;g__Microbacterium;s__Microbacterium_sp._JZ31
NCBI1906741	0	1	0	0	0	2	0	1	0	0	0	0	0	0	0	k__Bacteria;p__Pseudomonadota;c__Betaproteobacteria;o__Neisseriales;f__Chromobacteriaceae;g__Jeongeupia;s__Jeongeupia_sp._USM3
NCBI1906742	0	0	0	0	0	6	0	0	0	0	1	0	1	2	0	k__Bacteria;p__Actinomycetota;c__Actinomycetes;o__Micrococcales;f__Microbacteriaceae;g__Microbacterium;s__Microbacterium_sp._BH-3-3-3
NCBI1907	0	0	0	0	0	2	0	0	0	0	0	0	0	0	0	k__Bacteria;p__Actinomycetota;c__Actinomycetes;o__Kitasatosporales;f__Streptomycetaceae;g__Streptomyces;s__Streptomyces_glaucescens
NCBI190721	10	3	6	14	23	40	20	6	3	0	0	9	9	0	4	k__Bacteria;p__Pseudomonadota;c__Betaproteobacteria;o__Burkholderiales;f__Burkholderiaceae;g__Ralstonia;s__Ralstonia_insidiosa
NCBI1908	0	0	0	0	143	0	0	0	0	0	0	0	0	0	0	k__Bacteria;p__Actinomycetota;c__Actinomycetes;o__Kitasatosporales;f__Streptomycetaceae;g__Streptomyces;s__Streptomyces_globisporus
NCBI190893	1	0	3	1	5	2	0	0	0	1	0	0	0	0	0	k__Bacteria;p__Pseudomonadota;c__Gammaproteobacteria;o__Vibrionales;f__Vibrionaceae;g__Vibrio;s__Vibrio_coralliilyticus
NCBI190966	0	0	0	0	0	1	0	0	0	0	0	0	0	0	0	k__Bacteria;p__;c__;o__;f__;g__;s__uncultured_pig_faeces_bacterium
NCBI190975	0	0	0	0	0	0	0	2	0	0	0	0	0	0	0	k__Archaea;p__Euryarchaeota;c__Methanobacteria;o__Methanobacteriales;f__Methanobacteriaceae;g__Methanobrevibacter;s__Methanobrevibacter_thaueri
NCBI190977	0	0	0	0	0	6	0	0	0	0	0	0	0	0	0	k__Archaea;p__Euryarchaeota;c__Methanobacteria;o__Methanobacteriales;f__Methanobacteriaceae;g__Methanobrevibacter;s__Methanobrevibacter_wolinii

NCBI1911586	0	0	0	0	0	1	0	0	0	0	0	0	0	0	0	k__Bacteria;p__Bacillota;c__Bacilli;o__Lactobacillales;f__Carnobacteriaceae;g__Marinilactibacillus;s__Marinilactibacillus_sp._15R
NCBI1912891	0	0	0	0	0	58	0	0	0	0	0	0	0	0	0	k__Bacteria;p__Pseudomonadota;c__Alphaproteobacteria;o__Sphingomonadales;f__Sphingomonadaceae;g__Sphingobium;s__Sphingobium_sp.
NCBI191390	0	0	0	0	0	3	0	0	0	0	0	0	0	0	0	k__Bacteria;p__Pseudomonadota;c__Gammaproteobacteria;o__Pseudomonadales;f__Pseudomonadaceae;g__Pseudomonas;s__Pseudomonas_palleroniana
NCBI1913989	0	0	0	0	0	0	0	0	0	0	0	1	0	0	0	k__Bacteria;p__Pseudomonadota;c__Gammaproteobacteria;o__;f__;g__;s__Gammaproteobacteria_bacterium
NCBI1914471	0	0	0	0	0	8	0	0	2	0	0	0	0	0	0	k__Bacteria;p__Pseudomonadota;c__Betaproteobacteria;o__Nitrosomonadales;f__Thiobacillaceae;g__Sulfuritortus;s__Sulfuritortus_calidifontis
NCBI1914525	79	99	46	10	151	2583	78	14	41	18	8	37	21	7	19	k__Bacteria;p__Pseudomonadota;c__Alphaproteobacteria;o__Sphingomonadales;f__Sphingomonadaceae;g__Sphingopyxis;s__Sphingopyxis_sp._FD7
NCBI1914541	10	15	10	0	122	669	32	0	0	0	0	27	9	0	0	k__Bacteria;p__Pseudomonadota;c__Alphaproteobacteria;o__Hyphomicrobiales;f__Rhizobiaceae;g__Rhizobium;s__Rhizobium_sp._Y9
NCBI1914861	4	1	2	8	15	58	2	1	0	0	0	0	0	0	0	k__Bacteria;p__Pseudomonadota;c__Gammaproteobacteria;o__Enterobacterales;f__Enterobacteriaceae;g__Enterobacter;s__Enterobacter_sp._SA187
NCBI191495	0	0	0	0	0	3	0	0	0	0	0	0	0	0	0	k__Bacteria;p__Actinomycetota;c__Thermoleophilia;o__Solirubrobacterales;f__Conexibacteraceae;g__Conexibacter;s__Conexibacter_woesei
NCBI1915078	0	0	0	0	0	4	0	0	0	0	0	0	0	0	0	k__Bacteria;p__Pseudomonadota;c__Alphaproteobacteria;o__Rhodobacterales;f__Paracoccaceae;g__Thioclava;s__Thioclava_nitratireducens

NCBI191610	0	1	0	0	0	2	0	0	0	0	0	0	0	0	0	k__Bacteria;p__Actinomycetota;c__Actinomycetes;o__Mycobacteriales;f__Corynebacteriaceae;g__Corynebacterium;s__Corynebacterium_atypicum
NCBI1916917	0	0	0	0	0	10	0	0	0	0	0	0	0	0	0	k__Bacteria;p__Actinomycetota;c__Actinomycetes;o__Micrococcales;f__Microbacteriaceae;g__Microbacterium;s__Microbacterium_sp._1.5R
NCBI1917218	0	1	0	0	1	6	0	0	0	0	0	0	0	0	0	k__Bacteria;p__Pseudomonadota;c__Betaproteobacteria;o__Rhodocyclales;f__Azonexaceae;g__Dechloromonas;s__Dechloromonas_sp.
NCBI1918613	0	0	0	0	0	0	31	0	0	0	0	0	0	0	0	k__Bacteria;p__Bacteroidota;c__Bacteroidia;o__Bacteroidales;f__Muribaculaceae;g__Muribaculum;s__uncultured_Muribaculum_sp.
NCBI192	3	2	4	14	17	21	6	0	5	0	0	0	0	0	0	k__Bacteria;p__Pseudomonadota;c__Alphaproteobacteria;o__Rhodospirillales;f__Azospirillaceae;g__Azospirillum;s__Azospirillum_brasilense
NCBI192010	0	0	0	0	0	0	2	0	0	0	0	0	0	0	0	k__Fungi;p__Ascomycota;c__Sordariomycetes;o__Hypocreales;f__Nectriaceae;g__Fusarium;s__Fusarium_mangiferae
NCBI1920114	0	0	0	0	0	0	0	0	0	0	0	0	22	0	0	k__Bacteria;p__Pseudomonadota;c__Gammaproteobacteria;o__Enterobacterales;f__Enterobacteriaceae;g__Leclercia;s__Leclercia_sp._LSNIH1
NCBI1920116	0	0	0	0	0	0	0	0	0	0	0	111	0	0	0	k__Bacteria;p__Pseudomonadota;c__Gammaproteobacteria;o__Enterobacterales;f__Enterobacteriaceae;g__Leclercia;s__Leclercia_sp._LSNIH3
NCBI1920191	0	0	0	0	18	49	0	0	0	0	0	0	0	0	0	k__Bacteria;p__Pseudomonadota;c__Betaproteobacteria;o__Burkholderiales;f__Comamonadaceae;g__Delftia;s__Delftia_sp._HK171
NCBI1921421	0	0	0	0	0	0	0	0	0	0	0	3	0	0	0	k__Bacteria;p__Bacillota;c__Bacilli;o__Bacillales;f__Bacillaceae;g__Geobacillus;s__Geobacillus_genomosp._3

NCBI1921510	0	2	0	0	6	2	0	0	0	0	0	0	0	0	0	k__Bacteria;p__Pseudomonadota;c__Alphaproteobacteria;o__Sphingomonadales;f__Sphingomonadaceae;g__Tardibacter;s__Tardibacter_chloracetimidivorans
NCBI1926290	0	0	0	0	0	20	0	0	0	0	0	0	0	0	16	k__Bacteria;p__Actinomycetota;c__Actinomycetes;o__Micrococcales;f__Microbacteriaceae;g__Cryobacterium;s__Cryobacterium_sp.
NCBI1926868	1	1	2	0	1	29	0	0	0	0	0	0	0	0	0	k__Bacteria;p__Pseudomonadota;c__Betaproteobacteria;o__Burkholderiales;f__Comamonadaceae;g__Acidovorax;s__Acidovorax_monticola
NCBI192812	0	0	0	0	0	4	0	0	0	0	0	4	0	0	0	k__Bacteria;p__Pseudomonadota;c__Alphaproteobacteria;o__Sphingomonadales;f__Erythrobacteraceae;g__Qipengyuania;s__Qipengyuania_flava
NCBI1928330	0	0	0	0	0	1	0	0	0	0	0	0	0	0	0	k__Heunggongvirae;p__Uroviricota;c__Caudoviricetes;o__;f__Tybeckvirinae;g__Maenadvirus;s__Lactobacillus_phage_P2
NCBI192843	0	0	0	0	0	1	0	0	0	0	0	0	0	0	0	k__Bacteria;p__Pseudomonadota;c__Betaproteobacteria;o__Burkholderiales;f__Comamonadaceae;g__Rhodoferax;s__Rhodoferax_ferrireducens
NCBI1928706	0	0	0	0	6	0	0	4	0	0	0	0	0	0	0	k__Fungi;p__Basidiomycota;c__Microbotryomycetes;o__Sporidiobolales;f__Sporidiobolaceae;g__Sporobolomyces;s__Sporobolomyces_sp.
NCBI1930593	0	0	0	0	0	0	0	0	0	0	0	1	0	0	0	k__Bacteria;p__Candidatus_Omnitrophica;c__;o__;f__;g__Candidatus_Velamenicoccus;s__Candidatus_Velamenicoccus_archaeovorus
NCBI1933220	0	0	0	2	0	0	0	0	0	0	0	0	0	0	0	k__Bacteria;p__Bacteroidota;c__Sphingobacteriia;o__Sphingobacteriales;f__Sphingobacteriaceae;g__Sphingobacterium;s__Sphingobacterium_sp._B29
NCBI1938334	0	0	0	0	13	0	0	0	0	0	0	0	0	0	0	k__Bacteria;p__Actinomycetota;c__Actinomycetes;o__Micrococcales;f__Microbacteriaceae;g__Microbacterium;s__Microbacterium_sp._TPU_3598

NCBI1938604	0	0	0	0	0	1	0	0	0	0	0	0	0	0	0	k__Bacteria;p__Pseudomonadota;c__Betaproteobacteria;o__Neisseriales;f__Chromobacteriaceae;g__Aquaspirillum;s__Aquaspirillum_sp._LM1
NCBI1938605	0	1	0	1	0	4	0	0	0	0	0	0	0	0	0	k__Bacteria;p__Pseudomonadota;c__Alphaproteobacteria;o__Caulobacterales;f__Caulobacteraceae;g__Brevundimonas;s__Brevundimonas_sp._LM2
NCBI1938607	0	0	0	0	1	1	0	0	0	0	0	0	0	0	0	k__Bacteria;p__Pseudomonadota;c__Alphaproteobacteria;o__Sphingomonadales;f__Sphingomonadaceae;g__Sphingomonas;s__Sphingomonas_sp._LM7
NCBI1940610	0	0	0	2	0	82	0	2	0	10	7	3	11	7	28	k__Bacteria;p__Pseudomonadota;c__Alphaproteobacteria;o__Hyphomicrobiales;f__Phreatobacteraceae;g__Phreatobacter;s__Phreatobacter_stygius
NCBI1940636	0	32	0	0	0	0	0	0	0	0	0	44	0	0	0	k__Bacteria;p__Pseudomonadota;c__Gammaproteobacteria;o__Pseudomonadales;f__Pseudomonadaceae;g__Pseudomonas;s__Pseudomonas_shirazica
NCBI1940690	0	0	0	0	0	1	0	0	0	0	0	0	0	0	0	k__Bacteria;p__Pseudomonadota;c__Gammaproteobacteria;o__Alteromonadales;f__Idiomarinaceae;g__Idiomarina;s__Idiomarina_andamanensis
NCBI1940762	0	0	0	0	0	1	0	0	0	0	0	0	0	0	0	k__Bacteria;p__Cyanobacteriota;c__Cyanophyceae;o__Nostocales;f__;g__;s__Nostocales_cyanobacterium_HT-58-2
NCBI1940789	0	0	0	157	0	0	0	0	0	0	0	0	0	0	0	k__Bacteria;p__Bacillota;c__Bacilli;o__Lactobacillales;f__Streptococcaceae;g__Lactococcus;s__Lactococcus_petauri
NCBI1945662	2	1	0	0	0	31	3	0	0	0	0	0	0	2	0	k__Bacteria;p__Pseudomonadota;c__Alphaproteobacteria;o__Rhodobacterales;f__Paracoccaceae;g__Paracoccus;s__Paracoccus_contaminans
NCBI195	0	0	0	0	0	0	0	0	0	0	0	1	0	0	0	k__Bacteria;p__Campylobacterota;c__Epsilonproteobacteria;o__Campylobacterales;f__Campylobacteraceae;g__Campylobacter;s__Campylobacter_coli

NCBI1950	0	0	0	0	0	2	0	0	0	0	0	0	0	0	0	k__Bacteria;p__Actinomycetota;c__Actinomycetes;o__Kitasatosporales;f__Streptomycetaceae;g__Streptomyces;s__Streptomyces_peucetius
NCBI195105	0	0	0	12	2	56	3	0	0	0	0	0	0	0	0	k__Bacteria;p__Pseudomonadota;c__Alphaproteobacteria;o__Rhodobacterales;f__Paracoccaceae;g__Haematobacter;s__Haematobacter_massiliensis
NCBI196024	0	0	0	0	0	0	0	0	0	0	0	11	0	0	0	k__Bacteria;p__Pseudomonadota;c__Gammaproteobacteria;o__Aeromonadales;f__Aeromonadaceae;g__Aeromonas;s__Aeromonas_dhakensis
NCBI1961362	3	2	5	2	6	24	2	0	3	5	0	7	0	0	0	k__Bacteria;p__Pseudomonadota;c__Alphaproteobacteria;o__Sphingomonadales;f__Sphingomonadaceae;g__Sphingomonas;s__Sphingomonas_sp._NIC1
NCBI196162	0	0	0	0	0	9	0	0	0	0	0	0	0	0	0	k__Bacteria;p__Actinomycetota;c__Actinomycetes;o__Propionibacteriales;f__Nocardioidaceae;g__Nocardioides;s__Nocardioides_sp._JS614
NCBI196180	0	0	0	3	0	15	0	0	0	0	0	270	0	0	0	k__Bacteria;p__Bacillota;c__Bacilli;o__Bacillales;f__Bacillaceae;g__Anoxybacillus;s__Anoxybacillus_kamchatkensis
NCBI1963024	0	0	0	0	0	0	0	0	0	0	0	8	0	0	0	k__Bacteria;p__Bacillota;c__Bacilli;o__Bacillales;f__Bacillaceae;g__Geobacillus;s__Geobacillus_sp._44C
NCBI1963025	0	0	0	0	0	0	0	0	0	0	0	2	0	0	0	k__Bacteria;p__Bacillota;c__Bacilli;o__Bacillales;f__Bacillaceae;g__Geobacillus;s__Geobacillus_sp._46C-IIa
NCBI1963026	0	0	0	0	0	0	0	0	0	0	0	16	0	0	0	k__Bacteria;p__Bacillota;c__Bacilli;o__Bacillales;f__Bacillaceae;g__Geobacillus;s__Geobacillus_sp._47C-IIb
NCBI1969806	0	0	0	0	0	1	0	0	0	0	0	0	0	0	0	k__Bacteria;p__Pseudomonadota;c__Alphaproteobacteria;o__Rhodospirillales;f__Acetobacteraceae;g__Acidibrevibacterium;s__Acidibrevibacterium_fodinaquatile
NCBI1969821	0	1	0	0	0	16	1	0	1	0	0	0	0	0	0	k__Bacteria;p__Pseudomonadota;c__Alphaproteobacteria;o__Hyphomicrobiales;f__Rhizobiaceae;g__Ciceribacter;s__Ciceribacter_thiooxidans

NCBI197	0	0	0	0	0	4	0	0	0	0	0	7	0	0	0	k__Bacteria;p__Campylobacterota;c__Epsilonproteobacteria;o__Campylobacterales;f__Campylobacteraceae;g__Campylobacter;s__Campylobacter_jejuni
NCBI1970093	0	0	0	0	0	25	0	0	0	0	0	1	0	0	0	k__Bacteria;p__Bacillota;c__Clostridia;o__Eubacteriales;f__Clostridiaceae;g__Clostridium;s__Clostridium_sp._001
NCBI197614	0	0	46	0	0	0	0	0	0	0	0	0	0	0	0	k__Bacteria;p__Bacillota;c__Bacilli;o__Lactobacillales;f__Streptococcaceae;g__Streptococcus;s__Streptococcus_pasteurianus
NCBI1977088	0	0	0	0	0	5	0	0	0	0	0	0	0	0	0	k__Bacteria;p__Actinomycetota;c__Actinomycetes;o__Kitasatosporales;f__Streptomycetaceae;g__Streptomyces;s__Streptomyces_ficellus
NCBI1977998	0	0	0	0	1	0	0	0	0	0	0	3	0	0	0	k__Heunggongvirae;p__Uroviricota;c__Caudoviricetes;o__;f__;g__;s__Phage_FAKO05_000032F
NCBI1979401	0	0	0	0	0	1	0	0	0	0	0	0	0	0	0	k__Bacteria;p__Pseudomonadota;c__Alphaproteobacteria;o__Rhodobacterales;f__Roseobacteraceae;g__Thalassobius;s__Thalassobius_sp.
NCBI1979527	5	0	8	23	23	174	0	2	1	0	0	15	0	0	2	k__Bacteria;p__Actinomycetota;c__Actinomycetes;o__Mycobacteriales;f__Corynebacteriaceae;g__Corynebacterium;s__Corynebacterium_kefirresidentii
NCBI1980001	0	0	0	0	0	27	0	0	0	0	0	0	0	0	0	k__Bacteria;p__Actinomycetota;c__Actinomycetes;o__Micrococcales;f__Promicromonosporaceae;g__Cellulosimicrobium;s__Cellulosimicrobium_sp._TH-20
NCBI198107	0	0	0	0	0	1	0	0	0	0	0	0	0	0	0	k__Bacteria;p__Pseudomonadota;c__Betaproteobacteria;o__Rhodocyclales;f__Zoogloeaceae;g__Azoarcus;s__Azoarcus_sp._CIB
NCBI1982626	0	0	0	0	0	0	0	0	0	0	0	6	0	0	0	k__Bacteria;p__Bacillota;c__Erysipelotrichia;o__Erysipelotrichales;f__Erysipelotrichaceae;g__Faecalibacillus;s__Faecalibacillus_intestinalis

NCBI198467	0	0	0	0	0	0	0	0	0	0	0	45	0	0	0	k__Bacteria;p__Bacillota;c__Bacilli;o__Bacillales;f__Bacillaceae;g__Anoxybacillus;s__Anoxybacillus_gonensis
NCBI1985873	0	0	0	0	3	7	2	0	0	0	0	0	0	0	0	k__Bacteria;p__Pseudomonadota;c__Betaproteobacteria;o__Nitrosomonadales;f__Sulfuricellaceae;g__Sulfuriferula;s__Sulfuriferula_sp._AH1
NCBI198618	0	2	0	1	4	13	0	0	0	0	0	0	0	0	0	k__Bacteria;p__Pseudomonadota;c__Gammaproteobacteria;o__Pseudomonadales;f__Pseudomonadaceae;g__Pseudomonas;s__Pseudomonas_umsongensis
NCBI1986952	0	0	0	0	1	0	0	0	0	0	0	0	0	0	0	k__Bacteria;p__Bacteroidota;c__Sphingobacteriia;o__Sphingobacteriales;f__Sphingobacteriaceae;g__;s__Sphingobacteriaceae_bacterium_GW460-11-11-14-LB5
NCBI1987369	0	1	1	1	1	0	3	3	0	2	1	1	0	1	0	k__Fungi;p__Ascomycota;c__Saccharomycetes;o__Saccharomycetales;f__Saccharomycetaceae;g__Saccharomyces;s__Saccharomyces_jurei
NCBI199	0	0	0	0	0	1	0	0	0	0	0	0	0	0	0	k__Bacteria;p__Campylobacterota;c__Epsilonproteobacteria;o__Campylobacterales;f__Campylobacteraceae;g__Campylobacter;s__Campylobacter_concisus
NCBI199592	0	0	0	0	39	1292	0	0	0	362	182	0	398	143	790	k__Bacteria;p__Actinomycetota;c__Actinomycetes;o__Micrococcales;f__Microbacteriaceae;g__Microbacterium;s__Microbacterium_paraoxydans
NCBI2001	0	0	0	0	0	1	0	0	0	0	0	0	0	0	0	k__Bacteria;p__Actinomycetota;c__Actinomycetes;o__Streptosporangiales;f__Streptosporangiaceae;g__Streptosporangium;s__Streptosporangium_roseum
NCBI2003121	0	0	0	0	2	7	0	0	0	0	0	0	0	0	0	k__Bacteria;p__Bacteroidota;c__Sphingobacteriia;o__Sphingobacteriales;f__Sphingobacteriaceae;g__Sphingobacterium;s__Sphingobacterium_sp._G1-14
NCBI2003315	0	0	0	0	0	10	0	0	0	0	0	0	0	0	0	k__Bacteria;p__Pseudomonadota;c__Alphaproteobacteria;o__Sphingomonadales;f__Erythrobacteraceae;g__Porphyrobacter;s__Porphyrobacter_sp._CACIAM_03H1

NCBI200451	1	0	0	0	5	6	0	0	0	0	0	0	0	0	0	k__Bacteria;p__Pseudomonadota;c__Gammaproteobacteria;o__Pseudomonadales;f__Pseudomonadaceae;g__Pseudomonas;s__Pseudomonas_poae
NCBI200452	0	0	2	0	0	0	0	0	0	0	0	0	0	0	0	k__Bacteria;p__Pseudomonadota;c__Gammaproteobacteria;o__Pseudomonadales;f__Pseudomonadaceae;g__Pseudomonas;s__Pseudomonas_congelans
NCBI2004644	17	12	6	0	5	31	4	0	7	0	2	3	0	0	7	k__Bacteria;p__Pseudomonadota;c__Gammaproteobacteria;o__Moraxellales;f__Moraxellaceae;g__Acinetobacter;s__Acinetobacter_sp._WCHA45
NCBI2004646	0	0	0	0	3	0	0	0	1	0	0	2	3	0	0	k__Bacteria;p__Pseudomonadota;c__Gammaproteobacteria;o__Moraxellales;f__Moraxellaceae;g__Acinetobacter;s__Acinetobacter_sp._WCHA55
NCBI2004647	1	0	0	0	0	0	0	0	0	0	0	0	0	0	0	k__Bacteria;p__Pseudomonadota;c__Gammaproteobacteria;o__Moraxellales;f__Moraxellaceae;g__Acinetobacter;s__Acinetobacter_sp._WCHAc010052
NCBI2004650	1	0	0	0	0	0	0	0	0	0	0	0	0	0	0	k__Bacteria;p__Pseudomonadota;c__Gammaproteobacteria;o__Moraxellales;f__Moraxellaceae;g__Acinetobacter;s__Acinetobacter_chinensis
NCBI2004710	22	55	102	0	65	39	42	8	28	28	14	17	10	8	11	k__Bacteria;p__Bacteroidota;c__Flavobacteriia;o__Flavobacteriales;f__Weeksellaceae;g__Cloacibacterium;s__Cloacibacterium_caeni
NCBI2005046	0	0	0	0	0	2	0	0	0	0	0	0	0	0	0	k__Bacteria;p__Pseudomonadota;c__Gammaproteobacteria;o__Xanthomonadales;f__Xanthomonadaceae;g__Stenotrophomonas;s__Stenotrophomonas_sp._WZN-1
NCBI2005464	0	0	0	0	0	12	0	0	0	0	0	0	0	0	0	k__Bacteria;p__Cyanobacteriota;c__Cyanophyceae;o__Nostocales;f__Scytonemataceae;g__Scytonema;s__Scytonema_sp._NIES-4073
NCBI2005884	0	0	0	0	0	9	0	0	0	0	0	0	0	0	0	k__Bacteria;p__Pseudomonadota;c__Betaproteobacteria;o__Rhodocyclales;f__Zoogloeaceae;g__Thauera;s__Thauera_sp._K11

NCBI2006115	0	1	0	0	0	0	0	0	0	0	0	0	0	0	0	k__Bacteria;p__Pseudomonadota;c__Gammaproteobacteria;o__Moraxellales;f__Moraxellaceae;g__Acinetobacter;s__Acinetobacter_piscicola
NCBI2009329	0	0	0	0	0	0	5	0	0	0	0	0	0	0	0	k__Bacteria;p__Pseudomonadota;c__Alphaproteobacteria;o__Rhodobacterales;f__Roseobacteraceae;g__Sagittula;s__Sagittula_sp._P11
NCBI2010829	0	0	0	0	0	49	0	0	0	0	0	0	0	0	0	k__Bacteria;p__Pseudomonadota;c__Gammaproteobacteria;o__Xanthomonadales;f__Rhodanobacteraceae;g__Aerosticca;s__Aerosticca_soli
NCBI2010972	0	0	0	0	0	2	0	0	0	0	0	0	0	0	0	k__Bacteria;p__Pseudomonadota;c__Alphaproteobacteria;o__Caulobacterales;f__Caulobacteraceae;g__Caulobacter;s__Caulobacter_rhizosphaerae
NCBI2011159	0	0	0	0	0	2	0	0	0	0	0	0	0	0	0	k__Bacteria;p__Pseudomonadota;c__Alphaproteobacteria;o__Sphingomonadales;f__Erythrobacteraceae;g__Erythrobacter;s__Erythrobacter_sp._KY5
NCBI2011922	0	0	0	0	2	0	0	0	0	0	0	0	0	0	0	k__Fungi;p__Chytridiomycota;c__Chytridiomycetes;o__Rhizophydiales;f__;g__;s__Rhizophydiales_sp.
NCBI2014	0	2	0	0	0	9	0	0	0	0	0	0	0	0	0	k__Bacteria;p__Actinomycetota;c__Actinomycetes;o__Streptosporangiales;f__Nocardiopsaceae;g__Nocardiopsis;s__Nocardiopsis_dassonvillei
NCBI2014534	0	4	0	1	13	10	0	3	0	0	0	0	0	0	0	k__Bacteria;p__Actinomycetota;c__Actinomycetes;o__Micrococcales;f__Microbacteriaceae;g__Microbacterium;s__Microbacterium_sp._PM5
NCBI2015316	0	0	0	2	6	10	4	0	2	0	0	0	0	0	0	k__Bacteria;p__Pseudomonadota;c__Alphaproteobacteria;o__Hyphomicrobiales;f__Boseaceae;g__Bosea;s__Bosea_sp._AS-1
NCBI2016196	0	0	0	0	0	2	0	0	0	0	0	0	0	0	0	k__Bacteria;p__Pseudomonadota;c__Alphaproteobacteria;o__Hyphomonadales;f__Hyphomonadaceae;g__Hyphomonas;s__Hyphomonas_sp._KY3

NCBI2017485	0	0	0	0	0	5	0	0	0	0	0	0	0	0	0	k__Bacteria;p__Actinomycetota;c__Actinomycetes;o__Micrococcales;f__Dermabacteraceae;g__Brachybacterium;s__Brachybacterium_avium
NCBI2017486	0	0	6	7	27	234	0	0	0	0	0	7	0	0	0	k__Bacteria;p__Actinomycetota;c__Actinomycetes;o__Propionibacteriales;f__Nocardioidaceae;g__Nocardioides;s__Nocardioides_sp._S5
NCBI2018067	0	0	0	0	8	15	3	0	0	0	0	0	0	0	0	k__Bacteria;p__Pseudomonadota;c__Gammaproteobacteria;o__Pseudomonadales;f__Pseudomonadaceae;g__Pseudomonas;s__Pseudomonas_sp._FDAARGOS_380
NCBI2018305	0	0	0	0	0	0	0	1	0	0	0	0	0	0	0	k__Bacteria;p__Pseudomonadota;c__Gammaproteobacteria;o__Alteromonadales;f__Shewanellaceae;g__Shewanella;s__Shewanella_bicestrii
NCBI2020312	0	0	0	0	1	7	0	0	0	0	0	0	0	0	0	k__Bacteria;p__Pseudomonadota;c__Alphaproteobacteria;o__Hyphomicrobiales;f__Rhizobiaceae;g__Rhizobium;s__Rhizobium_sp._CIAT894
NCBI2020313	0	0	6	0	0	0	0	0	0	0	0	0	0	0	0	k__Bacteria;p__Pseudomonadota;c__Alphaproteobacteria;o__Hyphomicrobiales;f__Rhizobiaceae;g__Rhizobium;s__Rhizobium_sp._TAL182
NCBI2020412	0	0	0	0	6	57	2	0	0	0	0	5	0	0	0	k__Bacteria;p__Pseudomonadota;c__Alphaproteobacteria;o__Hyphomicrobiales;f__Boseaceae;g__Bosea;s__Bosea_sp._ANAM02
NCBI2020486	0	0	0	0	11	9	0	0	0	0	0	0	0	0	0	k__Bacteria;p__Actinomycetota;c__Actinomycetes;o__Micrococcales;f__Micrococcaceae;g__Arthrobacter;s__Arthrobacter_sp._YN
NCBI2021379	0	0	0	0	2	0	0	0	0	0	0	0	0	0	0	k__Bacteria;p__Pseudomonadota;c__Alphaproteobacteria;o__Hyphomicrobiales;f__Xanthobacteraceae;g__;s__Xanthobacteraceae_bacterium
NCBI2021862	0	0	0	0	0	22	0	0	0	0	0	0	0	0	0	k__Bacteria;p__Pseudomonadota;c__Alphaproteobacteria;o__Hyphomicrobiales;f__Stappiaceae;g__Labrenzia;s__Labrenzia_sp._VG12

NCBI2023229	0	0	1	0	0	13	0	0	0	0	0	0	0	0	0	k__Bacteria;p__Pseudomonadota;c__Alphaproteobacteria;o__Sphingomonadales;f__Erythrobacteraceae;g__Porphyrobacter;s__Porphyrobacter_sp._HT-58-2
NCBI2024265	0	0	0	0	0	1	0	0	0	0	0	0	0	0	0	k__Heunggongvirae;p__Uroviricota;c__Caudoviricetes;o__;f__;g__;s__Agrobacterium_phage_Atu_ph08
NCBI2025658	0	0	0	0	0	2	4	0	0	0	0	3	1	0	0	k__Bacteria;p__Pseudomonadota;c__Gammaproteobacteria;o__Pseudomonadales;f__Pseudomonadaceae;g__Pseudomonas;s__Pseudomonas_sp._NS1(2017)
NCBI2025876	0	0	0	0	0	37	0	0	0	0	0	0	0	0	0	k__Bacteria;p__Bacteroidota;c__Bacteroidia;o__Bacteroidales;f__Tannerellaceae;g__Parabacteroides;s__Parabacteroides_sp._CT06
NCBI2026	0	0	0	2	2	0	1	0	0	0	0	0	0	0	5	k__Bacteria;p__Bacillota;c__Bacilli;o__Bacillales;f__Thermoactinomycetaceae;g__Thermoactinomyces;s__Thermoactinomyces_vulgaris
NCBI2026186	79	0	38	59	64	199	18	0	0	51	0	0	9	0	19	k__Bacteria;p__Bacillota;c__Bacilli;o__Bacillales;f__Bacillaceae;g__Bacillus;s__Bacillus_paranthracis
NCBI2026188	0	0	0	0	0	53	0	0	0	0	0	0	0	0	0	k__Bacteria;p__Bacillota;c__Bacilli;o__Bacillales;f__Bacillaceae;g__Bacillus;s__Bacillus_tropicus
NCBI2026189	0	0	0	0	1	3	0	0	1	0	0	0	0	0	0	k__Bacteria;p__Bacillota;c__Bacilli;o__Bacillales;f__Bacillaceae;g__Bacillus;s__Bacillus_albus
NCBI2026191	0	0	2	12	3	9	2	1	0	2	0	6	0	0	0	k__Bacteria;p__Bacillota;c__Bacilli;o__Bacillales;f__Bacillaceae;g__Bacillus;s__Bacillus_luti
NCBI2026199	0	0	0	0	0	5	0	0	0	0	0	0	0	0	0	k__Bacteria;p__Pseudomonadota;c__Betaproteobacteria;o__Burkholderiales;f__Burkholderiaceae;g__Paraburkholderia;s__Paraburkholderia_aromaticivorans
NCBI202669	47	69	54	95	114	411	59	39	46	56	64	34	36	20	25	k__Bacteria;p__Bacillota;c__Bacilli;o__Bacillales;f__;g__Exiguobacterium;s__uncultured_Exiguobacterium_sp.
NCBI2026716	0	0	0	0	0	0	0	0	0	0	0	1	0	0	0	k__Bacteria;p__Candidatus_Campbellbacteria;c__;o__;f__;g__;s__Candidatus_Campbellbacteria_bacterium

NCBI2026720	0	0	0	0	0	4	0	0	0	0	0	0	0	0	0	k__Bacteria;p__Candidatus_Saccharibacteria;c__;o__;f__;g__;s__Candidatus_Saccharibacteria_bacterium
NCBI2026724	1	0	0	0	0	0	0	0	0	0	0	0	0	0	0	k__Bacteria;p__Chloroflexota;c__;o__;f__;g__;s__Chloroflexota_bacterium
NCBI2026742	0	0	0	0	0	1	0	0	0	0	0	0	0	0	0	k__Bacteria;p__Gemmatimonadota;c__;o__;f__;g__;s__Gemmatimonadota_bacterium
NCBI2026777	0	0	0	0	5	3	0	0	0	0	0	0	0	0	0	k__Bacteria;p__Planctomycetota;c__Phycisphaerae;o__Phycisphaerales;f__Phycisphaeraceae;g__;s__Phycisphaeraceae_bacterium
NCBI2026786	0	0	0	0	3	8	0	0	0	0	0	0	0	0	0	k__Bacteria;p__Pseudomonadota;c__Alphaproteobacteria;o__Rhodospirillales;f__;g__;s__Rhodospirillales_bacterium
NCBI2026804	0	0	0	1	0	0	0	0	0	0	0	0	0	0	0	k__Bacteria;p__Candidatus_Woesebacteria;c__;o__;f__;g__;s__Candidatus_Woesebacteria_bacterium
NCBI2027405	0	0	0	0	0	5	0	0	0	0	0	0	0	0	0	k__Bacteria;p__Pseudomonadota;c__Betaproteobacteria;o__Rhodocyclales;f__Zoogloeaceae;g__Azoarcus;s__Azoarcus_sp._DD4
NCBI2027919	0	0	0	45	0	44	0	0	0	0	0	0	0	0	0	k__Bacteria;p__Pseudomonadota;c__Gammaproteobacteria;o__Enterobacterales;f__Enterobacteriaceae;g__Enterobacter;s__Enterobacter_cloacae_complex_sp.
NCBI2028345	0	0	1	0	0	0	0	0	0	0	0	0	0	0	0	k__Bacteria;p__Pseudomonadota;c__Betaproteobacteria;o__Burkholderiales;f__Alcaligenaceae;g__Pusillimonas;s__Pusillimonas_thiosulfatoxidans
NCBI202954	0	0	0	1	2	9	3	0	0	1	0	1	0	0	0	k__Bacteria;p__Pseudomonadota;c__Gammaproteobacteria;o__Moraxellales;f__Moraxellaceae;g__Acinetobacter;s__Acinetobacter_tandoii
NCBI202956	0	0	0	0	0	6	0	0	2	0	0	0	0	0	0	k__Bacteria;p__Pseudomonadota;c__Gammaproteobacteria;o__Moraxellales;f__Moraxellaceae;g__Acinetobacter;s__Acinetobacter_towneri

NCBI2029752	7	10	9	27	50	167	17	1	2	0	1	1	1	0	1	k__Fungi;p__Ascomycota;c__Sordariomycetes;o__Diaporthales;f__Cryphonectriaceae;g__Chrysoporthe;s__Chrysoporthe_puriensis
NCBI2029849	0	0	0	0	0	2	0	0	0	0	0	0	0	0	0	k__Bacteria;p__Pseudomonadota;c__Alphaproteobacteria;o__Maricaulales;f__Maricaulaceae;g__Marinicauda;s__Marinicauda_algicola
NCBI2029986	0	0	0	0	0	5	0	0	0	0	0	0	0	0	0	k__Bacteria;p__Pseudomonadota;c__Gammaproteobacteria;o__Alteromonadales;f__Shewanellaceae;g__Shewanella;s__Shewanella_sp._WE21
NCBI2030806	0	0	0	0	0	15	0	0	0	0	0	0	0	0	0	k__Bacteria;p__Pseudomonadota;c__Betaproteobacteria;o__Burkholderiales;f__Burkholderiaceae;g__;s__Burkholderiaceae_bacterium
NCBI203192	108	192	84	10	335	6324	192	12	20	30	31	38	15	32	25	k__Bacteria;p__Pseudomonadota;c__Gammaproteobacteria;o__Pseudomonadales;f__Pseudomonadaceae;g__Stutzerimonas;s__Stutzerimonas_chloritidismutans
NCBI2033	0	0	0	0	1	12	0	0	0	11	1	0	4	3	5	k__Bacteria;p__Actinomycetota;c__Actinomycetes;o__Micrococcales;f__Microbacteriaceae;g__Microbacterium;s__Microbacterium_testaceum
NCBI2033435	0	0	0	1	0	2	0	0	0	0	0	0	0	0	0	k__Bacteria;p__Pseudomonadota;c__Alphaproteobacteria;o__Rhodobacterales;f__Paracoccaceae;g__;s__Rhodobacteraceae_bacterium_QY30
NCBI2035	0	0	2	0	147	45	1998	6	63	0	7	55	0	3	0	k__Bacteria;p__Actinomycetota;c__Actinomycetes;o__Micrococcales;f__Microbacteriaceae;g__Curtobacterium;s__Curtobacterium_flaccumfaciens
NCBI204039	0	0	0	0	0	3	0	0	0	0	0	0	0	0	0	k__Bacteria;p__Pseudomonadota;c__Gammaproteobacteria;o__Enterobacterales;f__Pectobacteriaceae;g__Dickeya;s__Dickeya_dianthicola
NCBI2040586	5	0	5	0	0	0	0	0	0	0	0	0	0	0	0	k__Bacteria;p__Pseudomonadota;c__Gammaproteobacteria;o__Xanthomonadales;f__Xanthomonadaceae;g__Stenotrophomonas;s__Stenotrophomonas_sp._Pemsol

NCBI2041	0	0	0	0	0	0	5	0	0	0	0	1	0	0	0	k__Bacteria;p__Actinomycetota;c__Actinomycetes;o__Propionibacteriales;f__Nocardioidaceae;g__Aeromicrobium;s__Aeromicrobium_erythreum
NCBI2041044	0	0	0	0	0	14	0	0	0	0	0	0	0	0	0	k__Bacteria;p__Bacillota;c__Clostridia;o__Eubacteriales;f__Eubacteriaceae;g__Eubacterium;s__Eubacterium_maltosivorans
NCBI2045202	0	16	0	29	19	69	0	14	0	0	0	0	0	0	13	k__Bacteria;p__Pseudomonadota;c__Gammaproteobacteria;o__Enterobacterales;f__Enterobacteriaceae;g__;s__Enterobacteriaceae_bacterium_A-F18
NCBI2045208	0	0	0	0	0	0	0	2	0	0	0	0	0	0	0	k__Bacteria;p__Pseudomonadota;c__Betaproteobacteria;o__Burkholderiales;f__Oxalobacteraceae;g__Massilia;s__Massilia_violaceinigra
NCBI2045451	0	0	0	0	0	0	10	0	0	0	0	0	0	0	0	k__Bacteria;p__Pseudomonadota;c__Gammaproteobacteria;o__Xanthomonadales;f__Xanthomonadaceae;g__Stenotrophomonas;s__Stenotrophomonas_indicatrix
NCBI2047	2	0	0	4	13	11	0	0	0	0	0	7	0	0	0	k__Bacteria;p__Actinomycetota;c__Actinomycetes;o__Micrococcales;f__Micrococcaceae;g__Rothia;s__Rothia_dentocariosa
NCBI204773	0	0	0	0	0	3	0	0	0	0	0	0	0	0	0	k__Bacteria;p__Pseudomonadota;c__Betaproteobacteria;o__Burkholderiales;f__Oxalobacteraceae;g__Herminiimonas;s__Herminiimonas_arsenicoxydans
NCBI2048283	0	0	0	0	0	1	0	0	0	0	0	0	0	0	0	k__Bacteria;p__Pseudomonadota;c__Alphaproteobacteria;o__Rhodospirillales;f__Thalassospiraceae;g__Thalassospira;s__Thalassospira_marina
NCBI2048897	0	0	0	0	0	2	0	0	0	0	0	0	0	0	0	k__Bacteria;p__Pseudomonadota;c__Alphaproteobacteria;o__Hyphomicrobiales;f__Rhizobiaceae;g__Rhizobium;s__Rhizobium_sp._NXC24
NCBI2048898	0	0	0	4	11	0	1	0	0	0	0	3	0	0	0	k__Bacteria;p__Actinomycetota;c__Actinomycetes;o__Micrococcales;f__Microbacteriaceae;g__Microbacterium;s__Microbacterium_sp._Y-01

NCBI2049589	0	0	0	0	1	1	0	0	0	0	0	0	0	0	0	k__Bacteria;p__Pseudomonadota;c__Gammaproteobacteria;o__Pseudomonadales;f__Pseudomonadaceae;g__Pseudomonas;s__Pseudomonas_sp._HLS-6
NCBI2051553	0	1	0	0	7	13	0	0	0	2	0	0	0	0	0	k__Bacteria;p__Pseudomonadota;c__Alphaproteobacteria;o__Hyphomicrobiales;f__Methylobacteriaceae;g__Methylobacterium;s__Methylobacterium_currus
NCBI2052164	0	0	0	0	0	12	0	0	0	0	0	0	0	0	0	k__Bacteria;p__Planctomycetota;c__Planctomycetia;o__Gemmatales;f__Gemmataceae;g__;s__Gemmataceae_bacterium
NCBI2052166	0	0	0	0	0	0	1	0	0	0	0	0	0	0	0	k__Bacteria;p__Candidatus_Melainabacteria;c__;o__;f__;g__;s__Candidatus_Melainabacteria_bacterium
NCBI2052180	0	0	0	0	0	1	0	0	0	0	0	0	0	0	0	k__Bacteria;p__Planctomycetota;c__Phycisphaerae;o__Phycisphaerales;f__;g__;s__Phycisphaerales_bacterium
NCBI2052181	0	0	0	0	0	10	0	0	0	0	0	0	0	0	0	k__Bacteria;p__Planctomycetota;c__Planctomycetia;o__;f__;g__;s__Planctomycetia_bacterium
NCBI2053287	0	0	2	0	0	0	0	0	0	0	0	7	0	0	0	k__Bacteria;p__Pseudomonadota;c__Gammaproteobacteria;o__Moraxellales;f__Moraxellaceae;g__Acinetobacter;s__Acinetobacter_pseudolwoffii
NCBI2053832	0	0	0	0	35	0	7	0	0	0	0	0	0	4	0	k__Bacteria;p__Bacillota;c__Bacilli;o__Bacillales;f__Bacillaceae;g__Bacillus;s__Bacillus_sp._HBCD-sjtu
NCBI2054	0	0	0	0	0	1	0	0	0	0	0	4	0	0	0	k__Bacteria;p__Actinomycetota;c__Actinomycetes;o__Mycobacteriales;f__Gordoniaceae;g__Gordonia;s__Gordonia_bronchialis
NCBI2054914	0	0	0	0	0	0	4	0	0	0	0	0	0	0	0	k__Bacteria;p__Pseudomonadota;c__Gammaproteobacteria;o__Pseudomonadales;f__Pseudomonadaceae;g__Pseudomonas;s__Pseudomonas_sp._02C_26
NCBI2054919	0	0	0	0	0	1	0	0	0	0	0	0	0	0	0	k__Bacteria;p__Pseudomonadota;c__Gammaproteobacteria;o__Pseudomonadales;f__Pseudomonadaceae;g__Pseudomonas;s__Pseudomonas_sp._S09G_359
NCBI2	0	0	0	0	0	6	0	0	0	0	0	0	0	0	0	k__Bacteria;p__Actinomycetota;c__Actinomycetes;o__

055																Mycobacteriales;f__Gordoniaceae;g__Gordonia;s__Gordonia_terrae
NCBI2055955	0	0	0	0	0	22	4	0	0	0	0	0	0	0	0	k__Bacteria;p__Pseudomonadota;c__Alphaproteobacteria;o__Sphingomonadales;f__Erythrobacteraceae;g__Tsuneonella;s__Tsuneonella_flava
NCBI2056231	1222	1211	1025	7054	14841	22779	2151	457	1000	165	127	278	341	287	331	k__Bacteria;p__Pseudomonadota;c__Gammaproteobacteria;o__Pseudomonadales;f__Pseudomonadaceae;g__Pseudomonas;s__Pseudomonas_qingdaonensis
NCBI2057	0	0	0	0	0	0	0	0	0	0	0	1	0	0	0	k__Bacteria;p__Thermomicrobiota;c__Thermomicrobia;o__Sphaerobacterales;f__Sphaerobacteraceae;g__Sphaerobacter;s__Sphaerobacter_thermophilus
NCBI2057026	2	0	1	0	2	0	1	2	0	0	0	43	0	0	1	k__Bacteria;p__Pseudomonadota;c__Gammaproteobacteria;o__Enterobacterales;f__Enterobacteriaceae;g__Enterobacter;s__Enterobacter_sp._EA-1
NCBI2057741	0	1	0	2	5	11	1	0	0	0	0	0	0	1	0	k__Bacteria;p__Pseudomonadota;c__Alphaproteobacteria;o__Hyphomicrobiales;f__Nitrobacteraceae;g__Bradyrhizobium;s__Bradyrhizobium_sp._SK17
NCBI2057791	11	3	3	31	18	20	2	2	0	0	0	0	0	1	3	k__Bacteria;p__Bacillota;c__Bacilli;o__Lactobacillales;f__Enterococcaceae;g__Enterococcus;s__Enterococcus_sp._CR-Ec1
NCBI2058136	0	0	0	2	0	0	0	0	0	0	0	0	0	0	0	k__Bacteria;p__Bacillota;c__Bacilli;o__Bacillales;f__Planococcaceae;g__Planococcus;s__Planococcus_sp._MB-3u-03
NCBI2058152	24	0	0	0	0	508	0	0	0	0	0	0	0	0	0	k__Bacteria;p__Pseudomonadota;c__Gammaproteobacteria;o__Enterobacterales;f__Enterobacteriaceae;g__Klebsiella;s__Klebsiella_grimontii
NCBI205844	39	48	14	0	112	1042	52	6	9	9	0	9	0	5	0	k__Bacteria;p__Pseudomonadota;c__Alphaproteobacteria;o__Sphingomonadales;f__Sphingomonadaceae;g__Novosphingobium;s__Novosphingobium_pentaromativorans

NCBI2060307	0	0	0	4	11	0	0	0	0	0	0	0	0	0	0	k__Bacteria;p__Bacillota;c__Bacilli;o__Lactobacillales;f__Enterococcaceae;g__Enterococcus;s__Enterococcus_sp._FDAARGOS_375
NCBI2060312	0	0	0	0	0	2	0	0	0	0	0	0	0	0	0	k__Bacteria;p__Pseudomonadota;c__Alphaproteobacteria;o__Sphingomonadales;f__Erythrobacteraceae;g__Altererythrobacter;s__Altererythrobacter_sp._B11
NCBI2060726	0	3	0	0	1	29	0	0	0	0	0	0	0	0	0	k__Bacteria;p__Pseudomonadota;c__Alphaproteobacteria;o__Hyphomicrobiales;f__Rhizobiaceae;g__Neorhizobium;s__Neorhizobium_sp._SOG26
NCBI206506	0	0	0	0	0	3	0	0	0	0	0	0	0	0	0	k__Bacteria;p__Pseudomonadota;c__Betaproteobacteria;o__Burkholderiales;f__Alcaligenaceae;g__Kerstersia;s__Kerstersia_gyiorum
NCBI2065379	0	0	0	0	0	0	2	0	0	0	0	0	0	0	0	k__Bacteria;p__Pseudomonadota;c__Alphaproteobacteria;o__Rhodobacterales;f__Paracoccaceae;g__Paracoccus;s__Paracoccus_jeotgali
NCBI2067415	0	0	1	0	1	1	0	0	0	0	0	0	0	0	0	k__Bacteria;p__Pseudomonadota;c__Alphaproteobacteria;o__Sphingomonadales;f__Erythrobacteraceae;g__Altererythrobacter;s__Altererythrobacter_sp._TH136
NCBI2067957	9	5	0	69	22	0	0	0	0	0	0	0	6	0	0	k__Bacteria;p__Pseudomonadota;c__Alphaproteobacteria;o__Hyphomicrobiales;f__Methylobacteriaceae;g__Methylobacterium;s__Methylobacterium_sp._DM1
NCBI2068655	0	0	0	0	0	1	0	0	0	0	0	0	0	0	0	k__Bacteria;p__Bacillota;c__Clostridia;o__Eubacteriales;f__Lachnospiraceae;g__Novisyntrophococcus;s__Novisyntrophococcus_fermenticellae
NCBI2070347	0	0	0	0	8	0	0	0	0	0	0	0	0	0	0	k__Bacteria;p__Actinomycetota;c__Actinomycetes;o__Micrococcales;f__Microbacteriaceae;g__Agrococcus;s__Agrococcus_sp._SGAir0287
NCBI2070348	0	0	0	0	0	27	4	0	0	0	0	0	0	0	0	k__Bacteria;p__Actinomycetota;c__Actinomycetes;o__Micrococcales;f__Microbacteriaceae;g__Microbacterium;s__Microbacterium_sp._SGAir0570

NCBI2070537	0	0	0	0	0	3	0	0	0	0	0	0	0	0	0	k__Bacteria;p__Pseudomonadota;c__Alphaproteobacteria;o__Rhodospirillales;f__Acetobacteraceae;g__Commensalibacter;s__Commensalibacter_sp._ESL0284
NCBI2071710	0	0	0	0	0	0	0	1	0	0	0	0	0	0	0	k__Bacteria;p__Pseudomonadota;c__Gammaproteobacteria;o__Enterobacterales;f__Enterobacteriaceae;g__Enterobacter;s__Enterobacter_sichuanensis
NCBI2072025	0	0	0	0	1	0	0	0	0	0	0	0	0	0	0	k__Bacteria;p__Bacillota;c__Bacilli;o__Bacillales;f__Bacillaceae;g__Lysinibacillus;s__Lysinibacillus_sp._YS11
NCBI2072413	0	0	0	0	0	0	0	0	1	0	0	0	0	0	0	k__Bacteria;p__Pseudomonadota;c__Gammaproteobacteria;o__Xanthomonadales;f__Xanthomonadaceae;g__Stenotrophomonas;s__Stenotrophomonas_sp._SAU14A_NAIMI4_5
NCBI2072420	0	0	0	0	0	7	0	0	0	0	0	0	0	0	1	k__Bacteria;p__Pseudomonadota;c__Alphaproteobacteria;o__Hyphomicrobiales;f__Nitrobacteraceae;g__;s__Bradyrhizobiaceae_bacterium
NCBI2072503	0	0	0	0	0	1	0	0	0	0	0	0	0	0	0	k__Bacteria;p__Actinomycetota;c__Actinomycetes;o__Pseudonocardiales;f__Pseudonocardiaceae;g__Actinoalloteichus;s__Actinoalloteichus_sp._AHMU_CJ021
NCBI2072936	0	0	0	0	0	4	0	0	0	0	0	0	0	0	0	k__Bacteria;p__Pseudomonadota;c__Alphaproteobacteria;o__Sphingomonadales;f__Sphingomonadaceae;g__Sphingobium;s__Sphingobium_sp._SCG-1
NCBI207340	0	0	0	0	6	11	0	0	0	0	0	0	0	0	0	k__Bacteria;p__Pseudomonadota;c__Alphaproteobacteria;o__Rhodospirillales;f__Acetobacteraceae;g__Roseomonas;s__Roseomonas_mucosa
NCBI2074	0	0	0	3	0	9	0	0	0	0	0	1	0	0	0	k__Bacteria;p__Actinomycetota;c__Actinomycetes;o__Pseudonocardiales;f__Pseudonocardiaceae;g__Pseudonocardia;s__Pseudonocardia_autotrophica
NCBI2077200	3	0	0	0	10	20	0	0	0	0	0	0	0	0	0	k__Archaea;p__Euryarchaeota;c__Halobacteria;o__Haloferacales;f__Haloferacaceae;g__Haloferax;s__Haloferax_sp._Atlit-24N

NCBI207745	16	11	0	0	24	223	15	0	5	0	0	0	6	0	0	k__Bacteria;p__Pseudomonadota;c__Betaproteobacteria;o__Burkholderiales;f__Comamonadaceae;g__Variovorax;s__Variovorax_sp._WDL1
NCBI2079806	1	0	0	0	0	11	0	0	0	0	0	0	0	0	0	k__Bacteria;p__Pseudomonadota;c__Gammaproteobacteria;o__Pseudomonadales;f__Pseudomonadaceae;g__;s__Pseudomonadaceae_bacterium_SI-3
NCBI2080419	0	1	0	0	1	1	1	0	0	0	0	0	0	0	0	k__Bacteria;p__Deinococcota;c__Deinococci;o__Deinococcales;f__Deinococcaceae;g__Deinococcus;s__Deinococcus_sp._NW-56
NCBI2080469	13	0	0	0	19	24	0	6	0	0	0	0	0	0	0	k__Bacteria;p__Pseudomonadota;c__Betaproteobacteria;o__Rhodocyclales;f__Zoogloeaceae;g__;s__Zoogloeaceae_bacteirum_Par-f-2
NCBI2081702	0	0	0	0	0	3	0	0	0	0	0	0	0	0	0	k__Bacteria;p__Actinomycetota;c__Actinomycetes;o__Actinomycetales;f__Actinomycetaceae;g__Actinomyces;s__Actinomyces_sp._oral_taxon_897
NCBI2081703	0	0	0	0	0	3	0	0	0	0	0	1	0	0	0	k__Bacteria;p__Bacillota;c__Clostridia;o__Eubacteriales;f__Peptostreptococcaceae;g__;s__Peptostreptococcaceae_bacterium_oral_taxon_929
NCBI2082188	13	33	13	0	64	958	18	0	9	11	4	4	2	1	6	k__Bacteria;p__Pseudomonadota;c__Alphaproteobacteria;o__Sphingomonadales;f__Sphingomonadaceae;g__Sphingobium;s__Sphingobium_sp._YG1
NCBI208223	0	0	0	2	1	0	3	0	0	0	0	1	0	0	0	k__Bacteria;p__Pseudomonadota;c__Gammaproteobacteria;o__Enterobacterales;f__Enterobacteriaceae;g__Kosakonia;s__Kosakonia_cowanii
NCBI208224	0	0	0	10	0	11	0	0	0	0	0	0	0	0	0	k__Bacteria;p__Pseudomonadota;c__Gammaproteobacteria;o__Enterobacterales;f__Enterobacteriaceae;g__Enterobacter;s__Enterobacter_kobei
NCBI2082386	0	0	0	0	0	12	0	0	0	0	0	0	0	0	0	k__Bacteria;p__Pseudomonadota;c__Betaproteobacteria;o__Burkholderiales;f__Sphaerotilaceae;g__Methylibium;s__Methylibium_sp._Pch-M

NCBI2082387	0	0	0	0	0	5	0	0	0	0	0	0	0	0	0	k__Bacteria;p__Pseudomonadota;c__Alphaproteobacteria;o__Hyphomicrobiales;f__Phyllobacteriaceae;g__Mesorhizobium;s__Mesorhizobium_sp._Pch-S
NCBI2083052	0	10	8	10	51	57	0	0	0	0	0	0	0	0	0	k__Bacteria;p__Pseudomonadota;c__Gammaproteobacteria;o__Pseudomonadales;f__Pseudomonadaceae;g__Pseudomonas;s__Pseudomonas_sp._SWI36
NCBI208479	1	0	6	0	2	29	50	0	0	0	0	18	0	0	0	k__Bacteria;p__Bacillota;c__Clostridia;o__Eubacteriales;f__Lachnospiraceae;g__Enterocloster;s__Enterocloster_bolteae
NCBI208544	0	0	0	0	0	395	0	0	0	0	0	0	0	0	0	k__Bacteria;p__Pseudomonadota;c__Betaproteobacteria;o__Burkholderiales;f__;g__;s__uncultured_Burkholderiales_bacterium
NCBI2086471	0	0	0	0	0	1	0	0	0	0	0	0	0	0	0	k__Bacteria;p__Bacteroidota;c__Cytophagia;o__Cytophagales;f__Hymenobacteraceae;g__Adhaeribacter;s__Adhaeribacter_swui
NCBI2086584	0	0	0	0	0	1	0	0	0	0	0	0	0	0	0	k__Bacteria;p__Bacillota;c__Clostridia;o__Eubacteriales;f__;g__Massilistercora;s__Massilistercora_timonensis
NCBI2093742	0	0	0	0	0	0	2	0	0	0	0	0	0	0	0	k__Bacteria;p__Bacillota;c__Clostridia;o__Eubacteriales;f__Lachnospiraceae;g__;s__Lachnospiraceae_bacterium_KM106-2
NCBI2093856	0	1	0	0	1	0	0	0	0	0	0	4	0	0	0	k__Bacteria;p__Bacteroidota;c__Bacteroidia;o__Bacteroidales;f__Odoribacteraceae;g__Butyricimonas;s__Butyricimonas_faecalis
NCBI2093857	0	0	0	0	0	27	75	0	0	0	0	5	0	0	0	k__Bacteria;p__Bacillota;c__Clostridia;o__Eubacteriales;f__Oscillospiraceae;g__Dysosmobacter;s__Dysosmobacter_welbionis
NCBI2094242	0	0	0	0	1	0	2	1	0	0	0	0	0	0	0	k__Bacteria;p__Lentisphaerota;c__Lentisphaeria;o__Victivallales;f__;g__;s__Victivallales_bacterium_CCUG_44730

NCBI2099675	0	0	0	0	0	3	0	0	0	0	0	0	1	0	2	k__Bacteria;p__Bacteroidota;c__Cytophagia;o__Cytophagales;f__Cyclobacteriaceae;g__;s__Cyclobacteriaceae_bacterium
NCBI2099789	0	0	1	0	3	0	2	0	0	1	0	0	0	1	1	k__Bacteria;p__Bacillota;c__Bacilli;o__Lactobacillales;f__Lactobacillaceae;g__Lactobacillus;s__Lactobacillus_sp._CBA3606
NCBI210	0	0	0	0	0	7	0	0	0	0	0	0	0	0	0	k__Bacteria;p__Campylobacterota;c__Epsilonproteobacteria;o__Campylobacterales;f__Helicobacteraceae;g__Helicobacter;s__Helicobacter_pylori
NCBI2100421	0	1	0	0	0	11	1	0	0	0	0	0	0	0	0	k__Heunggongvirae;p__Uroviricota;c__Caudoviricetes;o__;f__;g__;s__uncultured_Caudovirales_phage
NCBI2100821	3084	3256	3205	4609	4127	1260	4577	3792	4509	4445	4091	5321	1704	1728	1794	k__Bacteria;p__Bacillota;c__Bacilli;o__Lactobacillales;f__Lactobacillaceae;g__Lacticaseibacillus;s__Lacticaseibacillus_chiayiensis
NCBI2104	2	0	0	0	9	973	1	0	0	0	0	3	0	0	0	k__Bacteria;p__Mycoplasmatota;c__;o__Mycoplasmoidales;f__Metamycoplasmataceae;g__Mycoplasmoides;s__Mycoplasmoides_pneumoniae
NCBI2107999	61	39	64	80	69	188	68	46	87	110	76	93	35	35	24	k__Bacteria;p__Bacillota;c__Bacilli;o__Lactobacillales;f__Lactobacillaceae;g__Lactobacillus;s__Lactobacillus_paragasseri
NCBI2108445	0	0	0	0	0	3	0	0	0	0	0	0	0	0	0	k__Bacteria;p__Pseudomonadota;c__Alphaproteobacteria;o__Hyphomicrobiales;f__Phyllobacteriaceae;g__Mesorhizobium;s__Mesorhizobium_sp._DCY119
NCBI2109685	0	0	0	0	0	13	0	0	0	0	0	3	0	0	0	k__Bacteria;p__Actinomycetota;c__Coriobacteriia;o__Coriobacteriales;f__Atopobiaceae;g__Olsenella;s__Olsenella_sp._GAM18
NCBI2109687	0	0	0	0	0	29	0	0	0	0	0	24	0	0	0	k__Bacteria;p__Bacillota;c__Clostridia;o__Eubacteriales;f__Oscillospiraceae;g__Oscillibacter;s__Oscillibacter_sp._PEA192
NCBI2	0	0	0	0	0	27	0	0	0	0	0	2	0	0	0	k__Bacteria;p__Bacillota;c__Clostridia;o__Eubacteriales

109688															;f__;g__;s__Clostridiales_bacterium_CCNA10
NCBI2 109690	0	0	4	0	9	2	4	2	0	0	0	0	0	0	0	k__Bacteria;p__Bacillota;c__Clostridia;o__Eubacteriales;f__Lachnospiraceae;g__;s__Lachnospiraceae_bacterium_Choco86
NCBI2 109691	0	0	7	0	0	7	1	3	0	0	0	35	1	0	0	k__Bacteria;p__Bacillota;c__Clostridia;o__Eubacteriales;f__Lachnospiraceae;g__;s__Lachnospiraceae_bacterium_GAM79
NCBI2 109692	0	0	0	0	0	0	0	0	0	0	0	1	0	0	0	k__Bacteria;p__Bacillota;c__Erysipelotrichia;o__Erysipelotrichales;f__Erysipelotrichaceae;g__;s__Erysipelotrichaceae_bacterium_GAM147
NCBI2 109913	0	0	0	0	2	10	0	0	0	0	0	2	0	0	0	k__Bacteria;p__Pseudomonadota;c__Betaproteobacteria;o__Burkholderiales;f__Comamonadaceae;g__Melaminivora;s__Melaminivora_suipulveris
NCBI2 109914	0	0	0	0	1	2	0	0	0	0	0	0	0	0	0	k__Bacteria;p__Pseudomonadota;c__Betaproteobacteria;o__Burkholderiales;f__Comamonadaceae;g__Ottowia;s__Ottowia_oryzae
NCBI2 109915	1	0	0	0	0	6	0	0	0	0	0	0	0	0	0	k__Bacteria;p__Pseudomonadota;c__Betaproteobacteria;o__Burkholderiales;f__Comamonadaceae;g__Simplicispira;s__Simplicispira_suum
NCBI2 116657	0	0	0	0	0	2	0	0	0	0	0	0	0	0	0	k__Bacteria;p__Pseudomonadota;c__Betaproteobacteria;o__Burkholderiales;f__Comamonadaceae;g__Pulveribacter;s__Pulveribacter_suum
NCBI2 12362	0	0	0	0	0	0	2	0	0	0	0	0	0	0	0	k__Bacteria;p__Cyanobacteriota;c__Cyanophyceae;o__Nostocales;f__Nostocaceae;g__Nostoc;s__Nostoc_paludosum
NCBI2 126319	0	0	0	0	0	36	0	0	0	0	0	0	0	0	0	k__Bacteria;p__Pseudomonadota;c__Betaproteobacteria;o__Burkholderiales;f__Comamonadaceae;g__Variovorax;s__Variovorax_sp._PMC12
NCBI2 126321	0	0	0	4	0	1	0	0	0	0	0	0	0	0	0	k__Bacteria;p__Pseudomonadota;c__Gammaproteobacteria;o__Enterobacterales;f__Yersiniaceae;g__Nissabacter;

																s__Nissabacter_sp._SGAir0207
NCBI213	0	0	0	0	0	0	0	0	0	0	0	3	0	0	0	k__Bacteria;p__Campylobacterota;c__Epsilonproteobacteria;o__Campylobacterales;f__Helicobacteraceae;g__Helicobacter;s__Helicobacter_cinaedi
NCBI2133760	0	0	0	0	1	0	0	0	0	0	0	0	0	0	0	k__Heunggongvirae;p__Uroviricota;c__Caudoviricetes;o__;f__;g__;s__Siphoviridae_environmental_samples
NCBI213419	0	0	0	4	0	0	0	0	0	0	0	59	0	0	0	k__Bacteria;p__Bacillota;c__Bacilli;o__Bacillales;f__Bacillaceae;g__Geobacillus;s__Geobacillus_zalihae
NCBI2136173	0	0	0	2	0	0	0	0	0	0	0	0	0	0	0	k__Bacteria;p__Actinomycetota;c__Actinomycetes;o__Kitasatosporales;f__Streptomycetaceae;g__Streptomyces;s__Streptomyces_sp._So13.3
NCBI2144175	0	0	0	0	0	0	0	0	0	0	0	0	0	1	0	k__Bacteria;p__Bacillota;c__Negativicutes;o__Veillonellales;f__Veillonellaceae;g__Megasphaera;s__Megasphaera_stantonii
NCBI214856	0	0	0	0	0	2	9	0	0	0	0	4	0	0	0	k__Bacteria;p__Bacteroidota;c__Bacteroidia;o__Bacteroidales;f__Rikenellaceae;g__Alistipes;s__Alistipes_finegoldii
NCBI215221	1725	1667	1754	5488	4158	3743	2536	1685	2120	9044	8152	12023	34815	34180	42094	k__Bacteria;p__Pseudomonadota;c__Alphaproteobacteria;o__Rhodospirillales;f__Acetobacteraceae;g__Komagataeibacter;s__Komagataeibacter_rhaeticus
NCBI2153354	0	0	0	0	1	0	0	0	0	0	0	0	0	0	0	k__Bacteria;p__Pseudomonadota;c__Gammaproteobacteria;o__Enterobacterales;f__Enterobacteriaceae;g__Klebsiella;s__Klebsiella_huaxiensis
NCBI2153385	0	0	0	0	0	0	2	0	0	0	0	0	0	0	0	k__Bacteria;p__Pseudomonadota;c__Gammaproteobacteria;o__Enterobacterales;f__Enterobacteriaceae;g__Lelliottia;s__Lelliottia_sp._WB101
NCBI215580	0	0	0	0	0	9	0	0	0	0	0	0	0	0	0	k__Bacteria;p__Pseudomonadota;c__Betaproteobacteria;o__Burkholderiales;f__Sphaerotilaceae;g__Caldimonas;s__Caldimonas_thermodepolymerans

NCBI216142	1	0	0	3	0	0	0	0	0	0	0	0	0	0	0	k__Bacteria;p__Pseudomonadota;c__Gammaproteobacteria;o__Pseudomonadales;f__Pseudomonadaceae;g__Pseudomonas;s__Pseudomonas_rhizosphaerae
NCBI216465	0	2	0	0	3	11	0	0	0	0	0	0	0	0	0	k__Bacteria;p__Pseudomonadota;c__Betaproteobacteria;o__Burkholderiales;f__Comamonadaceae;g__Polaromonas;s__Polaromonas_naphthalenivorans
NCBI216778	11	18	12	98	128	193	13	6	10	3	1	3	12	5	7	k__Bacteria;p__Pseudomonadota;c__Gammaproteobacteria;o__Xanthomonadales;f__Xanthomonadaceae;g__Stenotrophomonas;s__Stenotrophomonas_rhizophila
NCBI216816	4	0	19	6	6	1011	9	4	0	0	57	381	3	0	0	k__Bacteria;p__Actinomycetota;c__Actinomycetes;o__Bifidobacteriales;f__Bifidobacteriaceae;g__Bifidobacterium;s__Bifidobacterium_longum
NCBI2171623	0	0	2	0	0	4	0	0	0	0	0	0	0	0	0	k__Bacteria;p__Actinomycetota;c__Actinomycetes;o__Micrococcales;f__Beutenbergiaceae;g__Miniimonas;s__Miniimonas_sp._S16
NCBI217203	0	0	0	1	0	0	14	1	0	0	0	0	0	0	0	k__Bacteria;p__Pseudomonadota;c__Betaproteobacteria;o__Burkholderiales;f__Alcaligenaceae;g__Achromobacter;s__Achromobacter_spanius
NCBI2172549	0	0	0	0	0	31	0	0	0	0	0	0	0	0	0	k__Bacteria;p__Planctomycetota;c__Candidatus_Brocadiia;o__Candidatus_Brocadiales;f__Candidatus_Brocadiaceae;g__Candidatus_Brocadia;s__Candidatus_Brocadia_sp.
NCBI2173	0	0	0	0	0	42	0	0	0	0	0	3	0	0	0	k__Archaea;p__Euryarchaeota;c__Methanobacteria;o__Methanobacteriales;f__Methanobacteriaceae;g__Methanobrevibacter;s__Methanobrevibacter_smithii
NCBI2182384	0	0	0	0	0	3	0	0	0	0	0	0	0	0	0	k__Bacteria;p__Pseudomonadota;c__Alphaproteobacteria;o__Sphingomonadales;f__Erythrobacteraceae;g__Erythrobacter;s__Erythrobacter_aureus
NCBI218284	0	0	0	0	0	0	0	0	2	1	0	0	0	0	0	k__Bacteria;p__Bacillota;c__Bacilli;o__Bacillales;f__Bacillaceae;g__Rossellomorea;s__Rossellomorea_vietname

																nsis
NCBI2183896	0	0	0	0	4	0	0	0	0	0	0	0	0	0	0	k__Bacteria;p__Bacteroidota;c__Flavobacteriia;o__Flavobacteriales;f__Flavobacteriaceae;g__Flavobacterium;s__Flavobacterium_crocinum
NCBI2184519	17	11	19	1	60	508	44	0	6	0	0	5	0	0	5	k__Bacteria;p__Pseudomonadota;c__Betaproteobacteria;o__Burkholderiales;f__Comamonadaceae;g__Hydrogenophaga;s__Hydrogenophaga_sp._NH-16
NCBI2185111	37	0	115	0	152	2282	39	0	0	0	0	0	0	0	0	k__Bacteria;p__Pseudomonadota;c__Alphaproteobacteria;o__Sphingomonadales;f__Sphingomonadaceae;g__Sphingobium;s__Sphingobium_sp._LF-16
NCBI218936	0	0	0	0	0	2	0	0	0	0	0	0	0	0	0	k__Bacteria;p__Pseudomonadota;c__Gammaproteobacteria;o__Aeromonadales;f__Aeromonadaceae;g__Aeromonas;s__Aeromonas_simiae
NCBI2202151	0	0	0	0	1	0	0	0	0	0	0	0	0	0	0	k__Bacteria;p__Aquificota;c__;o__;f__;g__;s__Aquificae_bacterium
NCBI2202644	0	0	0	0	0	0	3	0	0	0	0	0	0	0	0	k__Sangervirae;p__Phixviricota;c__Malgrandaviricetes;o__Petitvirales;f__Microviridae;g__;s__Microviridae_sp.
NCBI2202734	0	0	0	0	0	1	0	0	0	0	0	0	0	0	0	k__Bacteria;p__Bacteroidota;c__Saprospiria;o__Saprospirales;f__Saprospiraceae;g__;s__Saprospiraceae_bacterium
NCBI2202825	0	0	0	0	1	0	0	0	0	0	0	0	0	0	0	k__Bacteria;p__Pseudomonadota;c__Alphaproteobacteria;o__Hyphomicrobiales;f__Methylobacteriaceae;g__Methylobacterium;s__Methylobacterium_durans
NCBI2202826	0	0	1	0	0	0	0	0	0	0	0	0	0	0	0	k__Bacteria;p__Pseudomonadota;c__Alphaproteobacteria;o__Hyphomicrobiales;f__Methylobacteriaceae;g__Methylobacterium;s__Methylobacterium_sp._17Sr1-1
NCBI2202827	0	0	0	0	0	0	1	0	0	0	0	0	0	0	0	k__Bacteria;p__Pseudomonadota;c__Alphaproteobacteria;o__Hyphomicrobiales;f__Methylobacteriaceae;g__Methylobacterium;s__Methylobacterium_terrae

NCBI2202828	0	1	0	54	1	25	2	1	0	1	0	0	0	0	0	k__Bacteria;p__Pseudomonadota;c__Alphaproteobacteria;o__Hyphomicrobiales;f__Methylobacteriaceae;g__Methylobacterium;s__Methylobacterium_radiodurans
NCBI2203895	0	0	0	0	0	0	0	0	0	0	0	2	0	0	0	k__Bacteria;p__Pseudomonadota;c__Gammaproteobacteria;o__Moraxellales;f__Moraxellaceae;g__Psychrobacter;s__Psychrobacter_sp._YP14
NCBI220672	0	0	0	0	0	1	0	0	0	0	0	0	0	0	0	k__Fungi;p__Ascomycota;c__Dothideomycetes;o__Pleosporales;f__Leptosphaeriaceae;g__Plenodomus;s__Plenodomus_biglobosus
NCBI221027	0	0	0	0	1	0	0	0	0	0	0	0	0	0	0	k__Bacteria;p__Spirochaetota;c__Spirochaetia;o__Spirochaetales;f__Treponemataceae;g__Treponema;s__Treponema_putidum
NCBI2211108	0	0	0	0	0	1	0	0	0	0	0	0	0	0	0	k__Bacteria;p__Pseudomonadota;c__Betaproteobacteria;o__Nitrosomonadales;f__Sterolibacteriaceae;g__;s__Sterolibacteriaceae_bacterium_J5B
NCBI2211160	0	0	0	0	17	59	0	0	0	0	0	0	0	0	0	k__Bacteria;p__Pseudomonadota;c__Gammaproteobacteria;o__Xanthomonadales;f__Xanthomonadaceae;g__Stenotrophomonas;s__Stenotrophomonas_sp._pho
NCBI2212991	0	0	0	0	0	1	0	0	0	0	0	0	0	0	0	k__Bacteria;p__Bacillota;c__Clostridia;o__Eubacteriales;f__Clostridiaceae;g__Clostridium;s__Clostridium_sp._AWRP
NCBI2217832	0	0	0	0	0	0	0	8	0	0	0	0	0	0	0	k__Bacteria;p__Bacillota;c__Bacilli;o__Bacillales;f__Bacillaceae;g__Bacillus;s__Bacillus_sp._JAS24-2
NCBI2217867	0	0	0	0	0	0	2	0	0	0	0	0	0	0	0	k__Bacteria;p__Pseudomonadota;c__Gammaproteobacteria;o__Pseudomonadales;f__Pseudomonadaceae;g__Pseudomonas;s__Pseudomonas_sp._SGAir0191
NCBI2219057	0	0	0	0	0	1	0	0	0	0	0	0	0	0	0	k__Bacteria;p__Pseudomonadota;c__Gammaproteobacteria;o__Pseudomonadales;f__Pseudomonadaceae;g__Pseudomonas;s__Pseudomonas_sp._LG1E9

NCBI2219103	0	0	0	1	0	8	0	0	0	1	0	6	0	0	0	k__Loebvirae;p__Hofneiviricota;c__Faserviricetes;o__Tubulavirales;f__Inoviridae;g__;s__Inoviridae_sp.
NCBI2219225	0	0	41	0	0	0	0	0	0	0	0	0	0	0	0	k__Bacteria;p__Pseudomonadota;c__Gammaproteobacteria;o__Pseudomonadales;f__Pseudomonadaceae;g__Pseudomonas;s__Pseudomonas_asiatica
NCBI2219696	1	6	0	8	13	34	16	0	0	12	0	5	1	12	26	k__Bacteria;p__Pseudomonadota;c__Alphaproteobacteria;o__Sphingomonadales;f__Sphingomonadaceae;g__Sphingomonas;s__Sphingomonas_sp._FARSPH
NCBI2220096	0	0	0	0	0	1	0	0	0	0	0	0	0	0	0	k__Bacteria;p__Pseudomonadota;c__Alphaproteobacteria;o__Rhodospirillales;f__Azospirillaceae;g__Indioceanicola;s__Indioceanicola_profundi
NCBI2231055	0	0	0	0	0	1	0	0	0	0	0	0	0	0	0	k__Bacteria;p__Pseudomonadota;c__Betaproteobacteria;o__Rhodocyclales;f__Azonexaceae;g__Dechloromonas;s__Dechloromonas_sp._HYN0024
NCBI223192	0	0	0	1	0	0	0	0	0	0	0	0	0	0	0	k__Fungi;p__Ascomycota;c__Sordariomycetes;o__Togniniales;f__Togniniaceae;g__Phaeoacremonium;s__Phaeoacremonium_minimum
NCBI223919	0	30	0	15	12	25	0	0	0	0	0	0	0	0	0	k__Bacteria;p__Bacillota;c__Clostridia;o__Eubacteriales;f__Clostridiaceae;g__Clostridium;s__Clostridium_diolis
NCBI223967	6	0	5	20	46	55	1	0	0	0	0	0	0	0	0	k__Bacteria;p__Pseudomonadota;c__Alphaproteobacteria;o__Hyphomicrobiales;f__Methylobacteriaceae;g__Methylorubrum;s__Methylorubrum_populi
NCBI224719	0	0	0	0	0	0	0	0	0	0	0	2	0	0	0	k__Archaea;p__Euryarchaeota;c__Methanobacteria;o__Methanobacteriales;f__Methanobacteriaceae;g__Methanobrevibacter;s__Methanobrevibacter_sp._AbM4
NCBI2249356	0	0	0	0	0	0	2	0	0	1	0	0	0	1	0	k__Bacteria;p__Bacteroidota;c__Flavobacteriia;o__Flavobacteriales;f__Flavobacteriaceae;g__Flavobacterium;s__Flavobacterium_fluviale
NCBI2252	2	0	5	0	3	0	1	0	0	0	0	1	0	0	0	k__Archaea;p__Euryarchaeota;c__Halobacteria;o__Haloferacales;f__Haloferacaceae;g__Haloferax;s__Haloferax

															_mediterranei
NCBI2259623	113	104	120	137	145	115	113	76	83	52	40	87	26	43	34	k__Bacteria;p__Bacillota;c__Bacilli;o__Lactobacillales;f__Lactobacillaceae;g__Oenococcus;s__Oenococcus_sp._UCMA_16435
NCBI225991	1891	2225	1162	270	5259	100994	2363	195	768	540	410	913	323	350	437	k__Bacteria;p__Pseudomonadota;c__Betaproteobacteria;o__Burkholderiales;f__Comamonadaceae;g__Comamonas;s__Comamonas_aquatica
NCBI225992	5	1	3	0	12	243	8	0	0	0	0	19	0	2	0	k__Bacteria;p__Pseudomonadota;c__Betaproteobacteria;o__Burkholderiales;f__Comamonadaceae;g__Comamonas;s__Comamonas_kerstersii
NCBI2267833	1233	998	694	204	3412	38377	1147	53	431	128	113	598	150	116	118	k__Bacteria;p__Pseudomonadota;c__Alphaproteobacteria;o__Hyphomicrobiales;f__Rhizobiaceae;g__Rhizobium;s__Rhizobium_oryzihabitans
NCBI2268461	0	0	0	1	0	0	0	0	0	0	0	0	0	0	0	k__Bacteria;p__Actinomycetota;c__Actinomycetes;o__Micrococcales;f__Microbacteriaceae;g__Microbacterium;s__Microbacterium_sp._ABRD_28
NCBI227322	40	89	115	115	131	384	78	41	94	95	37	71	13	6	19	k__Bacteria;p__Bacillota;c__Bacilli;o__Bacillales;f__Paenibacillaceae;g__Paenibacillus;s__uncultured_Paenibacillus_sp.
NCBI227942	0	0	0	0	0	0	0	0	0	0	0	1	0	0	0	k__Bacteria;p__Bacillota;c__Bacilli;o__Lactobacillales;f__Lactobacillaceae;g__Limosilactobacillus;s__Limosilactobacillus_gastricus
NCBI2282124	0	0	0	13	12	47	0	0	0	0	0	0	0	0	0	k__Bacteria;p__Pseudomonadota;c__Gammaproteobacteria;o__Xanthomonadales;f__Xanthomonadaceae;g__Stenotrophomonas;s__Stenotrophomonas_sp._ASS1
NCBI2282130	0	0	0	0	0	2	0	0	0	0	0	0	0	0	0	k__Archaea;p__Euryarchaeota;c__Halobacteria;o__Haloferacales;f__Haloferacaceae;g__Halobellus;s__Halobellus_sp._Atlit-31R
NCBI228229	1	11	6	9	6	5	7	6	6	7	5	3	2	0	3	k__Bacteria;p__Bacillota;c__Bacilli;o__Lactobacillales;f__Lactobacillaceae;g__Ligilactobacillus;s__Ligilactobaci

																llus_saerimneri
NCBI2282309	0	0	0	5	0	7	0	0	0	0	0	0	0	0	0	k__Bacteria;p__Pseudomonadota;c__Gammaproteobacteria;o__Enterobacterales;f__Enterobacteriaceae;g__Leclercia;s__Leclercia_sp._W17
NCBI2282310	0	0	0	0	3	10	0	0	0	0	0	0	0	0	0	k__Bacteria;p__Pseudomonadota;c__Gammaproteobacteria;o__Enterobacterales;f__Enterobacteriaceae;g__Leclercia;s__Leclercia_sp._W6
NCBI2282475	0	0	0	1	0	23	0	0	0	0	0	0	0	0	0	k__Bacteria;p__Pseudomonadota;c__Betaproteobacteria;o__Burkholderiales;f__Alcaligenaceae;g__Achromobacter;s__Achromobacter_sp._B7
NCBI2282656	0	0	0	0	0	0	0	0	0	0	0	0	89	0	0	k__Bacteria;p__Actinomycetota;c__Actinomycetes;o__Micrococcales;f__Microbacteriaceae;g__Humibacter;s__Humibacter_sp._BT305
NCBI228899	0	0	0	0	0	0	0	1	0	0	0	0	0	0	0	k__Bacteria;p__Bacillota;c__Bacilli;o__Bacillales;f__Bacillaceae;g__Peribacillus;s__Peribacillus_asahii
NCBI2291939	14	56	35	7	111	1964	79	19	28	0	27	9	14	0	25	k__Bacteria;p__Pseudomonadota;c__Alphaproteobacteria;o__Hyphomicrobiales;f__Rhizobiaceae;g__Rhizobium;s__Rhizobium_sp._ZX09
NCBI2292255	2	0	1	1	2	1	1	0	0	0	1	2	0	0	0	k__Bacteria;p__Bacillota;c__Bacilli;o__Lactobacillales;f__Lactobacillaceae;g__Levilactobacillus;s__Levilactobacillus_suantsaii
NCBI2293	1	0	0	0	1	0	0	0	0	0	0	0	0	0	0	k__Bacteria;p__Thermodesulfobacteriota;c__Desulfobacteria;o__Desulfobacterales;f__Desulfobacteraceae;g__Desulfobacter;s__Desulfobacter_postgatei
NCBI229731	0	0	0	0	10	10	0	0	0	1	0	0	0	0	0	k__Archaea;p__Euryarchaeota;c__Halobacteria;o__Natrialbales;f__Natrialbaceae;g__Halobiforma;s__Halobiforma_lacisalsi
NCBI230089	0	0	0	0	0	1	0	0	0	0	0	0	0	0	0	k__Bacteria;p__Pseudomonadota;c__Gammaproteobacteria;o__Enterobacterales;f__Morganellaceae;g__Photorha

															bdus;s__Photorhabdus_thracensis
NCBI2303331	0	0	0	0	0	2	0	0	0	0	0	0	0	0	0	k__Bacteria;p__Pseudomonadota;c__Gammaproteobacteria;o__Nevskiales;f__Sinobacteraceae;g__Solimonas;s__Solimonas_sp._K1W22B-7
NCBI2303332	0	0	1	0	0	0	0	0	0	0	0	0	0	0	0	k__Bacteria;p__Pseudomonadota;c__Gammaproteobacteria;o__Cellvibrionales;f__Cellvibrionaceae;g__Cellvibrio;s__Cellvibrio_sp._KY-GH-1
NCBI230361	0	0	0	0	0	1	0	0	0	0	0	0	0	0	0	k__Archaea;p__Euryarchaeota;c__Methanobacteria;o__Methanobacteriales;f__Methanobacteriaceae;g__Methanobrevibacter;s__Methanobrevibacter_millerae
NCBI2303750	0	0	0	0	18	0	0	0	0	0	0	0	0	0	0	k__Bacteria;p__Pseudomonadota;c__Gammaproteobacteria;o__Xanthomonadales;f__Xanthomonadaceae;g__Stenotrophomonas;s__Stenotrophomonas_sp._G4
NCBI2304600	0	0	0	0	0	2	0	0	0	0	0	0	0	0	0	k__Bacteria;p__Pseudomonadota;c__Alphaproteobacteria;o__Hyphomicrobiales;f__Breoghaniaceae;g__Breoghania;s__Breoghania_sp._L-A4
NCBI231049	781	722	663	1081	1107	263	792	788	1031	910	905	1089	360	322	370	k__Bacteria;p__Bacillota;c__Bacilli;o__Lactobacillales;f__Lactobacillaceae;g__Furfurilactobacillus;s__Furfurilactobacillus_rossiae
NCBI231269	0	3	0	0	2	0	0	0	0	0	0	1	0	0	0	k__Fungi;p__Ascomycota;c__Sordariomycetes;o__Hypocreales;f__Nectriaceae;g__Fusarium;s__Fusarium_coffeatum
NCBI2319844	0	0	1	0	0	15	0	0	0	0	1	0	0	0	3	k__Bacteria;p__Pseudomonadota;c__Alphaproteobacteria;o__Sphingomonadales;f__Sphingomonadaceae;g__Sphingomonas;s__Sphingomonas_paeninsulae
NCBI2320270	0	0	0	0	5	1	0	0	0	0	0	0	0	0	0	k__Bacteria;p__Pseudomonadota;c__Gammaproteobacteria;o__Pseudomonadales;f__Pseudomonadaceae;g__Pseudomonas;s__Pseudomonas_sp._DG56-2

NCBI2320868	74	81	66	175	183	191	41	29	48	21	12	16	14	7	9	k__Bacteria;p__Bacillota;c__Clostridia;o__Eubacteriales;f__Clostridiaceae;g__Clostridium;s__Clostridium_manihotivorum
NCBI2321230	0	0	0	1	1	1	0	0	0	0	0	0	0	0	1	k__Archaea;p__Euryarchaeota;c__Halobacteria;o__Halobacteriales;f__Halococcaceae;g__Halococcus;s__Halococcus_sp._IIIV-5B
NCBI232721	233	268	0	0	318	3888	194	36	0	0	64	0	0	0	0	k__Bacteria;p__Pseudomonadota;c__Betaproteobacteria;o__Burkholderiales;f__Comamonadaceae;g__Acidovorax;s__Acidovorax_sp._JS42
NCBI2338073	0	0	0	0	1	0	0	0	0	0	0	0	0	0	0	k__Bacteria;p__Pseudomonadota;c__Gammaproteobacteria;o__Enterobacterales;f__Yersiniaceae;g__Serratia;s__Serratia_inhibens
NCBI2341112	1	0	3	0	0	2	0	0	0	0	0	0	0	0	0	k__Bacteria;p__Pseudomonadota;c__Alphaproteobacteria;o__Hyphomicrobiales;f__Rhizobiaceae;g__Georhizobium;s__Georhizobium_profundi
NCBI2350	0	0	0	3	1	3	0	1	0	0	0	0	0	0	0	k__Bacteria;p__Bacteroidota;c__Saprospiria;o__Saprospirales;f__Haliscomenobacteraceae;g__Haliscomenobacter;s__Haliscomenobacter_hydrossis
NCBI2358187	0	0	0	0	0	10	0	0	0	0	0	0	0	0	0	k__Bacteria;p__Pseudomonadota;c__Gammaproteobacteria;o__Alteromonadales;f__Alteromonadaceae;g__Alteromonas;s__Alteromonas_sp._76-1
NCBI236753	0	0	0	0	0	0	0	0	0	0	0	0	1	0	0	k__Bacteria;p__Bacillota;c__Clostridia;o__Eubacteriales;f__Oscillospiraceae;g__Fastidiosipila;s__Fastidiosipila_sanguinis
NCBI2371	1	0	0	0	0	0	0	0	0	0	0	0	0	0	0	k__Bacteria;p__Pseudomonadota;c__Gammaproteobacteria;o__Xanthomonadales;f__Xanthomonadaceae;g__Xylella;s__Xylella_fastidiosa
NCBI237258	29	4	24	0	38	8	14	7	5	0	7	10	6	4	3	k__Bacteria;p__Bacteroidota;c__Flavobacteriia;o__Flavobacteriales;f__Weeksellaceae;g__Cloacibacterium;s__Cloacibacterium_normanense

NCBI237610	4	5	2	15	41	53	5	0	1	0	0	0	0	0	0	k__Bacteria;p__Pseudomonadota;c__Gammaproteobacteria;o__Pseudomonadales;f__Pseudomonadaceae;g__Pseudomonas;s__Pseudomonas_psychrotolerans
NCBI238015	1081	1070	1010	1515	1560	2687	1227	1190	1257	1189	1027	985	438	495	432	k__Bacteria;p__Bacillota;c__Bacilli;o__Lactobacillales;f__Lactobacillaceae;g__Liquorilactobacillus;s__Liquorilactobacillus_vini
NCBI239364	0	0	3	7	0	22	1	1	0	0	0	47	0	0	0	k__Viruses;p__;c__;o__;f__;g__;s__uncultured_human_fecal_virus
NCBI239935	0	0	1	0	71	208	446	6	47	0	3	38	0	0	0	k__Bacteria;p__Verrucomicrobiota;c__Verrucomicrobiae;o__Verrucomicrobiales;f__Akkermansiaceae;g__Akkermansia;s__Akkermansia_muciniphila
NCBI24	1	0	4	0	0	30	3	0	2	2	0	0	0	0	0	k__Bacteria;p__Pseudomonadota;c__Gammaproteobacteria;o__Alteromonadales;f__Shewanellaceae;g__Shewanella;s__Shewanella_putrefaciens
NCBI240427	14	8	19	21	32	18	14	12	16	11	13	23	9	10	12	k__Bacteria;p__Bacillota;c__Bacilli;o__Lactobacillales;f__Lactobacillaceae;g__Secundilactobacillus;s__Secundilactobacillus_paracollinoides
NCBI240521	0	2	1	0	11	107	5	0	0	0	0	0	0	0	0	k__Bacteria;p__Pseudomonadota;c__Alphaproteobacteria;o__Hyphomicrobiales;f__Rhizobiaceae;g__Rhizobium;s__Rhizobium_daejeonense
NCBI241622	0	0	0	0	4	0	0	4	0	0	0	0	0	0	0	k__Fungi;p__Mucoromycota;c__;o__;f__;g__;s__uncultured_Glomeromycotina
NCBI2419774	0	0	0	0	0	3	0	0	0	0	0	0	0	0	0	k__Bacteria;p__Actinomycetota;c__Actinomycetes;o__Micrococcales;f__Microbacteriaceae;g__Protaetiibacter;s__Protaetiibacter_intestinalis
NCBI2419781	0	0	0	0	0	1	0	0	0	0	0	0	0	0	0	k__Archaea;p__Euryarchaeota;c__Halobacteria;o__Natrialbales;f__Natrialbaceae;g__Salinadaptatus;s__Salinadaptatus_halalkaliphilus
NCBI2420199	0	0	0	0	0	6	0	0	0	0	0	0	0	0	0	k__Fungi;p__Basidiomycota;c__Agaricomycetes;o__Polyporales;f__Meruliaceae;g__Hydnophlebia;s__Hydnophl

															ebia_sp.
NCBI242606	0	0	0	7	5	8	0	0	0	0	0	0	0	0	0	k__Bacteria;p__Pseudomonadota;c__Gammaproteobacteria;o__Xanthomonadales;f__Rhodanobacteraceae;g__Luteibacter;s__Luteibacter_rhizovicinus
NCBI244128	97	127	85	172	181	327	141	174	144	142	115	114	56	66	48	k__Bacteria;p__Bacillota;c__Bacilli;o__Lactobacillales;f__Lactobacillaceae;g__Lactobacillus;s__Lactobacillus_sp._MONT4
NCBI244366	0	0	0	0	25	172	0	18	0	0	0	0	0	0	0	k__Bacteria;p__Pseudomonadota;c__Gammaproteobacteria;o__Enterobacterales;f__Enterobacteriaceae;g__Klebsiella;s__Klebsiella_variicola
NCBI244734	0	0	2	2	8	44	2	0	5	0	0	9	1	0	1	k__Bacteria;p__Pseudomonadota;c__Alphaproteobacteria;o__Hyphomicrobiales;f__Nitrobacteraceae;g__Bradyrhizobium;s__Bradyrhizobium_betae
NCBI245014	0	0	0	0	0	5	0	1	0	0	0	12	0	0	0	k__Bacteria;p__Bacillota;c__Clostridia;o__Eubacteriales;f__;g__;s__butyrate-producing_bacterium_SS3/4
NCBI245174	2	0	0	0	0	0	0	0	0	0	0	0	0	0	0	k__Fungi;p__Basidiomycota;c__Wallemiomycetes;o__Wallemiales;f__Wallemiaceae;g__Wallemia;s__Wallemia_ichthyophaga
NCBI245188	7	13	6	0	28	266	20	3	0	0	0	0	0	0	0	k__Bacteria;p__Pseudomonadota;c__Alphaproteobacteria;o__Rhodobacterales;f__Paracoccaceae;g__Yoonia;s__Yoonia_vestfoldensis
NCBI246	0	0	0	0	0	15	0	0	0	0	0	0	0	0	0	k__Bacteria;p__Bacteroidota;c__Flavobacteriia;o__Flavobacteriales;f__Weeksellaceae;g__Chryseobacterium;s__Chryseobacterium_balustinum
NCBI246787	0	0	1	0	0	1	1	0	0	0	0	7	0	0	0	k__Bacteria;p__Bacteroidota;c__Bacteroidia;o__Bacteroidales;f__Bacteroidaceae;g__Bacteroides;s__Bacteroides_cellulosilyticus
NCBI247	0	0	1	2	1	2	0	0	0	0	0	0	1	0	0	k__Bacteria;p__Bacteroidota;c__Flavobacteriia;o__Flavobacteriales;f__Weeksellaceae;g__Empedobacter;s__Empedobacter_brevis

NCBI247480	0	0	0	0	0	0	0	0	0	0	0	1	0	0	0	k__Bacteria;p__Bacillota;c__Bacilli;o__Bacillales;f__Bacillaceae;g__Anoxybacillus;s__Anoxybacillus_caldiproteolyticus
NCBI2478662	13	24	15	0	47	667	20	2	7	3	0	7	0	2	2	k__Bacteria;p__Pseudomonadota;c__Betaproteobacteria;o__Burkholderiales;f__Comamonadaceae;g__Acidovorax;s__Acidovorax_sp._1608163
NCBI2478912	0	0	0	0	1	0	0	0	0	0	0	0	0	0	0	k__Bacteria;p__Pseudomonadota;c__Alphaproteobacteria;o__Rhodospirillales;f__Acetobacteraceae;g__Commensalibacter;s__Commensalibacter_sp._AMU001
NCBI2478954	0	0	0	0	1	7	0	0	0	0	0	14	0	0	0	k__Bacteria;p__Bacillota;c__Erysipelotrichia;o__Erysipelotrichales;f__Coprobacillaceae;g__Catenibacterium;s__Catenibacterium_sp._co_0103
NCBI2479767	0	0	0	0	0	22	0	0	0	0	0	31	0	0	0	k__Bacteria;p__Bacillota;c__Clostridia;o__Eubacteriales;f__Lachnospiraceae;g__Blautia;s__Blautia_sp._SC05B48
NCBI2480625	0	0	0	0	3	0	0	0	0	0	0	0	0	0	0	k__Bacteria;p__Actinomycetota;c__Actinomycetes;o__Micrococcales;f__Microbacteriaceae;g__Plantibacter;s__Plantibacter_sp._PA-3-X8
NCBI2483401	0	0	0	2	0	2	0	0	0	0	0	0	0	0	0	k__Bacteria;p__Actinomycetota;c__Actinomycetes;o__Micrococcales;f__Microbacteriaceae;g__Microbacterium;s__Microbacterium_sp._10M-3C3
NCBI2487072	0	0	1	4	5	58	0	0	0	0	0	0	0	0	0	k__Bacteria;p__Bacteroidota;c__Flavobacteriia;o__Flavobacteriales;f__Weeksellaceae;g__Epilithonimonas;s__Epilithonimonas_vandammei
NCBI2487150	0	0	0	15	78	24	0	0	18	0	0	9	0	0	0	k__Bacteria;p__Pseudomonadota;c__Gammaproteobacteria;o__Enterobacterales;f__Enterobacteriaceae;g__Metakosakonia;s__Metakosakonia_sp._MRY16-398
NCBI2488560	0	0	0	0	0	0	1	0	0	0	0	0	0	0	0	k__Bacteria;p__Pseudomonadota;c__Betaproteobacteria;o__Burkholderiales;f__Alcaligenaceae;g__Pigmentiphaga;s__Pigmentiphaga_sp._H8

NCBI2488639	0	0	2	0	0	10	0	0	0	0	0	0	0	0	0	k__Bacteria;p__Pseudomonadota;c__Gammaproteobacteria;o__Enterobacterales;f__Pectobacteriaceae;g__Pectobacterium;s__Pectobacterium_versatile
NCBI248903	0	0	0	0	1	2	0	0	0	0	0	0	0	0	0	k__Bacteria;p__Bacillota;c__Bacilli;o__Bacillales;f__Paenibacillaceae;g__Paenibacillus;s__Paenibacillus_xylanilyticus
NCBI2489212	0	2	0	0	0	8	0	0	0	0	0	0	0	0	0	k__Bacteria;p__Actinomycetota;c__Actinomycetes;o__Micrococcales;f__Microbacteriaceae;g__Microbacterium;s__Microbacterium_sp._RG1
NCBI249058	0	0	0	0	0	1	0	0	0	0	0	0	0	0	0	k__Bacteria;p__Actinomycetota;c__Actinomycetes;o__Mycobacteriales;f__Gordoniaceae;g__Gordonia;s__Gordonia_otitidis
NCBI2490939	0	0	0	0	0	16	0	0	0	0	0	0	0	0	0	k__Bacteria;p__Cyanobacteriota;c__Cyanophyceae;o__Nostocales;f__Nostocaceae;g__Anabaena;s__Anabaena_sp._YBS01
NCBI2492396	1	0	0	0	0	2	0	0	0	0	0	1	0	0	0	k__Bacteria;p__Pseudomonadota;c__Gammaproteobacteria;o__Enterobacterales;f__Enterobacteriaceae;g__Kosakonia;s__Kosakonia_sp._CCTCC_M2018092
NCBI2492837	0	0	4	0	0	36	2	0	0	0	0	0	0	0	0	k__Bacteria;p__Pseudomonadota;c__Alphaproteobacteria;o__Sphingomonadales;f__Sphingomonadaceae;g__Sphingomonas;s__Sphingomonas_sp._C8-2
NCBI2493093	4	0	0	0	0	11	0	0	0	0	0	0	0	0	1	k__Bacteria;p__Pseudomonadota;c__Alphaproteobacteria;o__Hyphomicrobiales;f__Nitrobacteraceae;g__Bradyrhizobium;s__Bradyrhizobium_sp._LCT2
NCBI2494234	0	0	0	0	0	1	0	0	0	0	0	0	0	0	0	k__Bacteria;p__Pseudomonadota;c__Betaproteobacteria;o__Burkholderiales;f__Sutterellaceae;g__Sutterella;s__Sutterella_megalosphaeroides
NCBI2494374	1	0	1	0	2	5	1	0	0	0	0	0	0	0	0	k__Bacteria;p__Pseudomonadota;c__Alphaproteobacteria;o__Rhodobacterales;f__Paracoccaceae;g__Tabrizicola;s__Tabrizicola_piscis

NCBI2494701	0	0	0	0	0	0	0	0	0	0	0	3	0	0	0	k__Bacteria;p__Pseudomonadota;c__Gammaproteobacteria;o__Enterobacterales;f__Enterobacteriaceae;g__Enterobacter;s__Enterobacter_chengduensis
NCBI2496847	0	0	0	0	0	20	0	0	0	0	0	0	0	0	0	k__Bacteria;p__Pseudomonadota;c__Betaproteobacteria;o__Nitrosomonadales;f__Sterolibacteriaceae;g__;s__Sterolibacteriaceae_bacterium_M52
NCBI2496867	0	0	0	0	0	0	0	0	0	0	0	2	0	0	0	k__Bacteria;p__Actinomycetota;c__Actinomycetes;o__Actinomycetales;f__Actinomycetaceae;g__Flaviflexus;s__Flaviflexus_ciconiae
NCBI2497860	0	0	0	0	0	3	0	0	0	0	0	0	0	0	0	k__Bacteria;p__Bacillota;c__Clostridia;o__Eubacteriales;f__Cellulosilyticaceae;g__Cellulosilyticum;s__Cellulosilyticum_sp._WCF-2
NCBI2497863	0	0	0	0	0	1	0	0	0	0	0	0	0	0	0	k__Bacteria;p__Pseudomonadota;c__Betaproteobacteria;o__Burkholderiales;f__Oxalobacteraceae;g__Janthinobacterium;s__Janthinobacterium_sp._17J80-10
NCBI2498451	1	4	2	0	2	23	0	0	1	0	0	1	1	1	0	k__Bacteria;p__Pseudomonadota;c__Gammaproteobacteria;o__Chromatiales;f__Chromatiaceae;g__Rheinheimera;s__Rheinheimera_mangrovi
NCBI2498848	0	0	0	0	0	0	0	0	0	0	3	0	0	0	0	k__Bacteria;p__Pseudomonadota;c__Gammaproteobacteria;o__Pseudomonadales;f__Pseudomonadaceae;g__Pseudomonas;s__Pseudomonas_sp._MPC6
NCBI2499144	0	0	0	0	0	16	0	0	0	0	0	0	0	0	0	k__Bacteria;p__Pseudomonadota;c__Alphaproteobacteria;o__Hyphomicrobiales;f__Devosiaceae;g__Devosia;s__Devosia_sp._1566
NCBI2499213	0	0	0	0	4	0	0	0	0	0	0	0	3	1	0	k__Bacteria;p__Bacillota;c__Bacilli;o__Bacillales;f__Bacillaceae;g__Bacillus;s__Bacillus_sp._BD59S
NCBI2500532	14	12	12	86	80	192	15	4	5	1	6	2	0	17	10	k__Bacteria;p__Pseudomonadota;c__Alphaproteobacteria;o__Rhodobacterales;f__Paracoccaceae;g__Paracoccus;s__Paracoccus_sp._Arc7-R13

NCBI2500548	1358	1512	1431	3832	3162	2845	2001	1378	2048	6098	6042	8770	41144	40900	49782	k__Bacteria;p__Pseudomonadota;c__Alphaproteobacteria;o__Rhodospirillales;f__Acetobacteraceae;g__Acetobacter;s__Acetobacter_oryzoeni
NCBI2502779	0	0	5	0	0	0	0	0	0	0	0	0	0	0	0	k__Bacteria;p__Bacteroidota;c__Chitinophagia;o__Chitinophagales;f__Chitinophagaceae;g__Pseudocnuella;s__Pseudocnuella_soli
NCBI2502843	0	0	0	1	0	5	0	0	0	0	0	3	0	0	0	k__Bacteria;p__Pseudomonadota;c__Alphaproteobacteria;o__Sphingomonadales;f__Erythrobacteraceae;g__Erythrobacter;s__Erythrobacter_sp._HKB08
NCBI2502979	0	0	0	0	0	2	0	0	0	0	0	0	0	0	0	k__Bacteria;p__Pseudomonadota;c__Gammaproteobacteria;o__Pseudomonadales;f__Pseudomonadaceae;g__Pseudomonas;s__Pseudomonas_khazarica
NCBI2507539	0	0	0	3	0	0	0	0	0	0	0	0	0	0	0	k__Bacteria;p__Bacillota;c__Bacilli;o__Bacillales;f__Thermoactinomycetaceae;g__Thermoactinomyces;s__Thermoactinomyces_sp._YT06
NCBI2508168	1	0	0	0	0	0	0	0	0	0	0	0	0	0	0	k__Bacteria;p__Pseudomonadota;c__Gammaproteobacteria;o__Xanthomonadales;f__Xanthomonadaceae;g__Luteimonas;s__Luteimonas_sp._YGD11-2
NCBI2509614	0	0	0	0	1	1	0	0	0	0	0	0	0	0	0	k__Bacteria;p__Pseudomonadota;c__Betaproteobacteria;o__Burkholderiales;f__Comamonadaceae;g__Rhodoferax;s__Rhodoferax_sediminis
NCBI251701	0	0	0	0	0	10	0	0	0	0	0	0	0	0	0	k__Bacteria;p__Pseudomonadota;c__Gammaproteobacteria;o__Pseudomonadales;f__Pseudomonadaceae;g__Pseudomonas;s__Pseudomonas_syringae_group_genomosp._3
NCBI2517899	0	0	0	0	0	9	0	0	0	0	0	0	0	0	0	k__Bacteria;p__Pseudomonadota;c__Gammaproteobacteria;o__Moraxellales;f__Moraxellaceae;g__Psychrobacter;s__Psychrobacter_sp._KH172YL61
NCBI2518343	0	0	0	2	10	92	15	0	3	0	0	0	1	0	1	k__Bacteria;p__Pseudomonadota;c__Betaproteobacteria;o__Burkholderiales;f__Comamonadaceae;g__Acidovora

																x;s__Acidovorax_sp._JMULE5
NCBI2518371	10	0	0	0	0	16	0	0	0	0	0	0	0	0	0	k__Bacteria;p__Actinomycetota;c__Actinomycetes;o__Propionibacteriales;f__Nocardioidaceae;g__Nocardioides;s__Nocardioides_seonyuensis
NCBI2518644	0	0	0	0	1	3	0	0	0	0	0	0	0	0	0	k__Bacteria;p__Pseudomonadota;c__Gammaproteobacteria;o__Pseudomonadales;f__Pseudomonadaceae;g__Pseudomonas;s__Pseudomonas_tructae
NCBI2518971	0	0	0	0	449	10	3062	0	261	0	0	13	0	0	6	k__Bacteria;p__Bacteroidota;c__Bacteroidia;o__Bacteroidales;f__Muribaculaceae;g__Duncaniella;s__Duncaniella_dubosii
NCBI2527974	0	0	0	0	0	14	0	0	0	0	0	0	0	0	0	k__Bacteria;p__Planctomycetota;c__Planctomycetia;o__Isosphaerales;f__Isosphaeraceae;g__Tautonia;s__Tautonia_plasticadhaerens
NCBI2528021	0	0	0	0	2	0	0	0	0	0	0	0	0	0	0	k__Bacteria;p__Planctomycetota;c__Planctomycetia;o__Pirellulales;f__Pirellulaceae;g__Anatilimnocola;s__Anatilimnocola_aggregata
NCBI2528023	0	0	0	0	0	22	0	0	0	0	0	0	0	0	0	k__Bacteria;p__Planctomycetota;c__Planctomycetia;o__Gemmatales;f__Gemmataceae;g__Urbifossiella;s__Urbifossiella_limnaea
NCBI2528203	0	0	0	0	0	15	0	0	18	0	0	7	0	0	0	k__Bacteria;p__Bacteroidota;c__Bacteroidia;o__Bacteroidales;f__Bacteroidaceae;g__Bacteroides;s__Bacteroides_sp._A1C1
NCBI2528642	0	0	0	0	0	2	0	0	0	0	0	0	0	0	0	k__Bacteria;p__Pseudomonadota;c__Alphaproteobacteria;o__Hyphomicrobiales;f__Lichenihabitantaceae;g__Lichenihabitans;s__Lichenihabitans_psoromatis
NCBI2528964	0	0	0	0	0	1	0	0	1	0	0	0	0	0	0	k__Bacteria;p__Pseudomonadota;c__Alphaproteobacteria;o__Hyphomicrobiales;f__Xanthobacteraceae;g__Xanthobacter;s__Xanthobacter_dioxanivorans

NCBI2530390	0	0	0	0	223	12	1314	0	123	0	0	13	0	0	1	k__Bacteria;p__Bacteroidota;c__Bacteroidia;o__Bacteroidales;f__Muribaculaceae;g__Muribaculum;s__Muribaculum_gordoncarteri
NCBI253237	16	0	6	0	81	461	71	0	0	0	0	21	0	23	0	k__Bacteria;p__Pseudomonadota;c__Gammaproteobacteria;o__Pseudomonadales;f__Pseudomonadaceae;g__Pseudomonas;s__Pseudomonas_sp._phDV1
NCBI254	1	0	0	0	0	9	0	0	0	0	0	0	0	0	0	k__Bacteria;p__Bacteroidota;c__Flavobacteriia;o__Flavobacteriales;f__Weeksellaceae;g__Chryseobacterium;s__Chryseobacterium_indoltheticum
NCBI254436	116	194	90	310	362	703	242	140	128	577	514	700	4388	4120	4637	k__Bacteria;p__Pseudomonadota;c__Alphaproteobacteria;o__Rhodospirillales;f__Acetobacteraceae;g__Gluconacetobacter;s__uncultured_Gluconacetobacter_sp.
NCBI2545632	0	0	0	0	0	1	0	0	0	0	0	0	0	0	0	k__Bacteria;p__Pseudomonadota;c__Gammaproteobacteria;o__Chromatiales;f__Chromatiaceae;g__Rheinheimera;s__Rheinheimera_sp._D18
NCBI2545797	0	0	0	0	0	0	0	0	0	0	0	9	0	0	0	k__Bacteria;p__Pseudomonadota;c__Gammaproteobacteria;o__Moraxellales;f__Moraxellaceae;g__Acinetobacter;s__Acinetobacter_sp._FDAARGOS_724
NCBI2545798	0	0	0	0	0	1	0	0	0	0	0	0	0	0	0	k__Bacteria;p__Pseudomonadota;c__Gammaproteobacteria;o__Enterobacterales;f__Enterobacteriaceae;g__Cedecea;s__Cedecea_sp._FDAARGOS_727
NCBI2547601	0	0	0	0	0	9	4	0	0	0	0	8	0	0	0	k__Bacteria;p__Pseudomonadota;c__Alphaproteobacteria;o__Sphingomonadales;f__Erythrobacteraceae;g__Porphyrobacter;s__Porphyrobacter_sp._YT40
NCBI2548116	0	0	0	0	0	3	0	0	0	0	0	0	0	0	0	k__Heunggongvirae;p__Uroviricota;c__Caudoviricetes;o__;f__;g__;s__Streptococcus_phage_Javan346
NCBI255045	0	0	0	0	0	3	0	0	0	0	0	0	0	0	1	k__Bacteria;p__Pseudomonadota;c__Alphaproteobacteria;o__Hyphomicrobiales;f__Nitrobacteraceae;g__Bradyrhizobium;s__Bradyrhizobium_canariense

NCBI2559073	0	1	1	6	10	53	12	2	1	0	1	4	0	1	1	k__Bacteria;p__Actinomycetota;c__Actinomycetes;o__Propionibacteriales;f__Propionibacteriaceae;g__Cutibacterium;s__Cutibacterium_modestum
NCBI2559074	0	0	0	0	0	4	0	0	0	0	0	0	0	0	0	k__Bacteria;p__Pseudomonadota;c__Gammaproteobacteria;o__Pseudomonadales;f__Pseudomonadaceae;g__Pseudomonas;s__Pseudomonas_sp._S150
NCBI2559573	0	0	0	0	0	0	0	0	0	1	0	0	0	0	0	k__Archaea;p__Euryarchaeota;c__Halobacteria;o__Halobacteriales;f__Haloarculaceae;g__Halorhabdus;s__Halorhabdus_amylolytica
NCBI256	0	0	0	0	0	2	0	0	0	0	0	0	0	0	0	k__Bacteria;p__Bacteroidota;c__Flavobacteriia;o__Flavobacteriales;f__Flavobacteriaceae;g__Myroides;s__Myroides_odoratus
NCBI2560053	1	0	0	0	1	0	0	0	0	0	0	0	0	0	0	k__Bacteria;p__Pseudomonadota;c__Alphaproteobacteria;o__Rhodobacterales;f__Paracoccaceae;g__Paracoccus;s__Paracoccus_liaowanqingii
NCBI2560058	349	395	256	73	1269	15320	503	25	180	74	69	160	33	36	73	k__Bacteria;p__Pseudomonadota;c__Alphaproteobacteria;o__Caulobacterales;f__Caulobacteraceae;g__Brevundimonas;s__Brevundimonas_sp._Bb-A
NCBI2561924	0	1	0	0	1	17	0	0	0	0	1	1	0	0	0	k__Bacteria;p__Pseudomonadota;c__Alphaproteobacteria;o__Caulobacterales;f__Caulobacteraceae;g__Brevundimonas;s__Brevundimonas_sp._MF30-B
NCBI2562284	0	0	0	0	0	1	0	0	0	0	0	0	0	0	0	k__Bacteria;p__Pseudomonadota;c__Alphaproteobacteria;o__Hyphomicrobiales;f__Xanthobacteraceae;g__Pseudolabrys;s__Pseudolabrys_sp._FHR47
NCBI2562582	7	15	3	0	9	262	6	0	9	2	4	0	0	0	0	k__Bacteria;p__Pseudomonadota;c__Alphaproteobacteria;o__Caulobacterales;f__Caulobacteraceae;g__Brevundimonas;s__Brevundimonas_sp._'scallop'
NCBI2563600	0	0	0	0	0	0	0	0	0	2	0	0	0	0	0	k__Archaea;p__Nitrososphaerota;c__Nitrososphaeria;o__Nitrososphaerales;f__Nitrososphaeraceae;g__Candidatus_Nitrosocosmicus;s__Candidatus_Nitrosocosmicus_sp._

															SS
NCBI2564099	0	0	0	0	0	1	0	0	0	0	0	0	0	0	0	k__Bacteria;p__Bacillota;c__Clostridia;o__Eubacteriales;f__Oscillospiraceae;g__Ruminococcus;s__Ruminococcus_bovis
NCBI2565368	0	0	0	0	0	2	95	0	0	0	5	0	0	0	0	k__Bacteria;p__Pseudomonadota;c__Gammaproteobacteria;o__Pseudomonadales;f__Pseudomonadaceae;g__Pseudomonas;s__Pseudomonas_atacamensis
NCBI2565554	43	102	21	22	99	2745	98	9	30	46	43	111	106	107	167	k__Bacteria;p__Pseudomonadota;c__Alphaproteobacteria;o__Sphingomonadales;f__Sphingomonadaceae;g__Sphingobium;s__Sphingobium_sp._PAMC28499
NCBI2565555	0	0	0	0	2	0	0	0	0	0	0	0	0	0	0	k__Bacteria;p__Pseudomonadota;c__Alphaproteobacteria;o__Sphingomonadales;f__Sphingomonadaceae;g__Sphingomonas;s__Sphingomonas_sp._PAMC26645
NCBI2565556	0	0	0	0	0	1	0	0	0	0	0	0	0	0	0	k__Bacteria;p__Pseudomonadota;c__Alphaproteobacteria;o__Sphingomonadales;f__Sphingomonadaceae;g__Sphingopyxis;s__Sphingopyxis_sp._PAMC25046
NCBI2565558	0	0	0	0	0	3	1	0	0	0	0	0	0	0	0	k__Bacteria;p__Pseudomonadota;c__Betaproteobacteria;o__Burkholderiales;f__Comamonadaceae;g__Hydrogenophaga;s__Hydrogenophaga_sp._PAMC20947
NCBI256618	0	0	0	0	2	5	0	0	0	0	0	0	0	0	0	k__Bacteria;p__Pseudomonadota;c__Alphaproteobacteria;o__Hyphomicrobiales;f__Parvibaculaceae;g__Parvibaculum;s__Parvibaculum_lavamentivorans
NCBI256701	0	0	0	3	0	4	0	0	6	0	0	0	0	0	0	k__Bacteria;p__Actinomycetota;c__Actinomycetes;o__Micrococcales;f__Micrococcaceae;g__Glutamicibacter;s__Glutamicibacter_arilaitensis
NCBI2567881	0	0	0	0	0	5	0	0	0	0	0	0	0	0	0	k__Bacteria;p__Actinomycetota;c__Actinomycetes;o__Micrococcales;f__Micrococcaceae;g__Citricoccus;s__Citricoccus_sp._SGAir0253

NCBI2567941	0	0	0	0	1	0	0	0	0	0	0	0	0	0	0	k__Bacteria;p__Bacillota;c__Bacilli;o__Bacillales;f__Bacillaceae;g__Metabacillus;s__Metabacillus_sediminilitoris
NCBI2571029	0	0	5	5	0	15	0	0	0	0	0	2	0	9	0	k__Bacteria;p__Actinomycetota;c__Actinomycetes;o__Micrococcales;f__Dermabacteraceae;g__Brachybacterium;s__Brachybacterium_sp._SGAir0954
NCBI2571115	0	0	0	0	11	0	3	0	0	0	0	0	0	0	0	k__Bacteria;p__Pseudomonadota;c__Gammaproteobacteria;o__Xanthomonadales;f__Xanthomonadaceae;g__Pseudoxanthomonas;s__Pseudoxanthomonas_sp._X-1
NCBI2571749	13	4	11	9	17	227	1	2	4	0	0	5	7	0	5	k__Bacteria;p__Pseudomonadota;c__Alphaproteobacteria;o__Sphingomonadales;f__Sphingomonadaceae;g__Novosphingobium;s__Novosphingobium_sp._EMRT-2
NCBI2572036	2	1	1	0	4	8	1	0	0	5	0	1	1	0	0	k__Bacteria;p__Pseudomonadota;c__Alphaproteobacteria;o__Hyphomicrobiales;f__Beijerinckiaceae;g__;s__Beijerinckiaceae_bacterium_RH_AL1
NCBI257438	778	753	818	1847	1819	1678	1298	821	1186	7045	6231	9805	9388	9633	11894	k__Bacteria;p__Pseudomonadota;c__Alphaproteobacteria;o__Rhodospirillales;f__Acetobacteraceae;g__Gluconobacter;s__Gluconobacter_thailandicus
NCBI2575375	0	0	8	8	14	8	1	0	0	0	1	0	0	0	0	k__Bacteria;p__Pseudomonadota;c__Gammaproteobacteria;o__Enterobacterales;f__Erwiniaceae;g__Pantoea;s__Pantoea_sp._SO10
NCBI2575923	0	0	0	4	3	45	2	0	0	0	0	1	0	67	0	k__Bacteria;p__Actinomycetota;c__Actinomycetes;o__Micrococcales;f__Brevibacteriaceae;g__Brevibacterium;s__Brevibacterium_sp._CS2
NCBI2576376	0	0	0	2	0	0	0	0	0	0	0	0	0	0	0	k__Bacteria;p__Bacillota;c__Bacilli;o__Lactobacillales;f__Streptococcaceae;g__Streptococcus;s__Streptococcus_sp._1643
NCBI2576756	0	0	0	0	0	1	0	0	0	0	0	0	0	0	0	k__Bacteria;p__Bacillota;c__Clostridia;o__Eubacteriales;f__Oscillospiraceae;g__Caproicibacter;s__Caproicibacter_fermentans

NCBI2576841	0	0	0	0	0	1	0	0	0	0	0	0	0	0	0	k__Bacteria;p__Pseudomonadota;c__Gammaproteobacteria;o__Oceanospirillales;f__Halomonadaceae;g__Halomonas;s__Halomonas_sp._PA16-9
NCBI2576891	0	0	0	0	3	0	0	0	0	0	0	0	0	0	0	k__Bacteria;p__Verrucomicrobiota;c__Opitutae;o__Opitutales;f__Opitutaceae;g__Nibricoccus;s__Nibricoccus_aquaticus
NCBI257708	0	0	1	0	0	1	0	0	0	0	0	0	0	0	0	k__Bacteria;p__Pseudomonadota;c__Alphaproteobacteria;o__Rhodospirillales;f__Acetobacteraceae;g__Roseomonas;s__Roseomonas_gilardii
NCBI2579248	0	0	0	0	0	1	0	0	0	0	0	0	0	0	0	k__Bacteria;p__Pseudomonadota;c__Alphaproteobacteria;o__Hyphomicrobiales;f__Rhizobiaceae;g__Agrobacterium;s__Agrobacterium_sp._CGMCC_11546
NCBI2579977	8	14	13	0	62	593	16	5	1	0	0	24	0	1	1	k__Bacteria;p__Pseudomonadota;c__Alphaproteobacteria;o__Caulobacterales;f__Caulobacteraceae;g__Brevundimonas;s__Brevundimonas_sp._SGAir0440
NCBI258	0	0	0	0	0	2	1	0	0	0	0	0	0	0	0	k__Bacteria;p__Bacteroidota;c__Sphingobacteriia;o__Sphingobacteriales;f__Sphingobacteriaceae;g__Sphingobacterium;s__Sphingobacterium_spiritivorum
NCBI2580515	1	1	0	0	2	22	0	0	0	0	1	0	0	0	0	k__Bacteria;p__Pseudomonadota;c__Alphaproteobacteria;o__Hyphomicrobiales;f__Rhizobiaceae;g__Agrobacterium;s__Agrobacterium_sp._T29
NCBI258224	0	0	0	0	0	5	0	0	0	0	0	0	0	0	0	k__Bacteria;p__Actinomycetota;c__Actinomycetes;o__Mycobacteriales;f__Corynebacteriaceae;g__Corynebacterium;s__Corynebacterium_resistens
NCBI2582419	0	0	0	0	2	0	0	0	0	0	0	0	0	0	0	k__Bacteria;p__Bacillota;c__Negativicutes;o__Veillonellales;f__Veillonellaceae;g__Dialister;s__Dialister_hominis
NCBI2582905	0	0	0	0	0	12	0	0	0	0	0	0	0	0	0	k__Bacteria;p__Actinomycetota;c__Actinomycetes;o__Propionibacteriales;f__Nocardioidaceae;g__Nocardioides;s__Nocardioides_sp._S-1144

NCBI2582914	0	2	0	0	0	61	4	0	0	0	0	0	0	0	0	k__Bacteria;p__Pseudomonadota;c__Betaproteobacteria;o__Burkholderiales;f__Alcaligenaceae;g__Alcaligenes;s__Alcaligenes_ammonioxydans
NCBI2583231	0	0	0	0	0	2	0	0	0	0	0	0	0	0	0	k__Bacteria;p__Pseudomonadota;c__Alphaproteobacteria;o__Hyphomicrobiales;f__Rhizobiaceae;g__Rhizobium;s__Rhizobium_indicum
NCBI2583377	0	0	1	0	0	2	0	0	0	0	0	0	0	0	0	k__Bacteria;p__Bacillota;c__Bacilli;o__Bacillales;f__Paenibacillaceae;g__Saccharibacillus;s__Saccharibacillus_brassicae
NCBI2584940	0	0	0	0	2	0	0	0	0	0	0	0	0	0	0	k__Bacteria;p__Bacteroidota;c__Cytophagia;o__Cytophagales;f__Hymenobacteraceae;g__Hymenobacter;s__Hymenobacter_sp._DG01
NCBI2584943	0	0	0	0	0	0	0	0	0	0	0	11	0	0	0	k__Bacteria;p__Bacillota;c__Erysipelotrichia;o__Erysipelotrichales;f__Erysipelotrichaceae;g__Longibaculum;s__Longibaculum_sp._KGMB06250
NCBI2584944	0	0	0	0	0	0	0	0	0	0	0	1	0	0	0	k__Bacteria;p__Pseudomonadota;c__Betaproteobacteria;o__Burkholderiales;f__Sutterellaceae;g__Sutterella;s__Sutterella_faecalis
NCBI2585118	0	0	0	0	1	15	12	0	7	0	0	18	0	0	0	k__Bacteria;p__Bacteroidota;c__Bacteroidia;o__Bacteroidales;f__Rikenellaceae;g__Alistipes;s__Alistipes_communis
NCBI2585119	0	0	0	0	0	0	1	0	0	0	0	0	0	0	0	k__Bacteria;p__Bacteroidota;c__Bacteroidia;o__Bacteroidales;f__Rikenellaceae;g__Alistipes;s__Alistipes_dispar
NCBI2587412	0	0	1	0	0	0	0	1	1	2	1	1	2	0	0	k__Fungi;p__Ascomycota;c__Sordariomycetes;o__Sordariales;f__Podosporaceae;g__Podospora;s__Podospora_anserina
NCBI2587855	0	0	0	0	4	34	0	0	0	0	0	0	0	0	0	k__Bacteria;p__Pseudomonadota;c__Alphaproteobacteria;o__Rhodobacterales;f__Roseobacteraceae;g__Sulfitobacter;s__Sulfitobacter_sp._THAF37

NCBI2587856	0	0	0	0	0	0	0	0	0	0	0	3	0	0	0	k__Bacteria;p__Pseudomonadota;c__Gammaproteobacteria;o__Cellvibrionales;f__Microbulbiferaceae;g__Microbulbifer;s__Microbulbifer_sp._THAF38
NCBI2588519	1	5	1	3	1	0	161	0	0	0	0	0	0	0	0	k__Heunggongvirae;p__Uroviricota;c__Caudoviricetes;o__;f__;g__;s__Aeromonas_phage_4_L372X
NCBI2588534	0	0	0	1	0	0	0	0	0	0	0	0	0	0	0	k__Bacteria;p__Pseudomonadota;c__Betaproteobacteria;o__Nitrosomonadales;f__Methylophilaceae;g__Methylophilus;s__Methylophilus_medardicus
NCBI2588535	0	0	0	0	1	0	0	0	0	0	0	0	0	0	0	k__Bacteria;p__Pseudomonadota;c__Betaproteobacteria;o__Nitrosomonadales;f__Methylophilaceae;g__Candidatus_Methylopumilus;s__Candidatus_Methylopumilus_rimovensis
NCBI2589076	0	1	1	3	1	5	2	0	0	0	0	1	0	0	0	k__Bacteria;p__Pseudomonadota;c__Alphaproteobacteria;o__Rhodobacterales;f__Paracoccaceae;g__Paracoccus;s__Paracoccus_sp._AK26
NCBI2589974	0	0	0	0	0	16	0	0	0	0	0	0	0	0	0	k__Bacteria;p__Pseudomonadota;c__Alphaproteobacteria;o__Hyphomicrobiales;f__Phyllobacteriaceae;g__Mesorhizobium;s__Mesorhizobium_sp._B2-1-1
NCBI259	0	0	0	0	4	1	0	0	0	0	0	0	0	0	0	k__Bacteria;p__Bacteroidota;c__Sphingobacteriia;o__Sphingobacteriales;f__Sphingobacteriaceae;g__Sphingobacterium;s__Sphingobacterium_thalpophilum
NCBI259059	2098	2020	2040	1787	1624	1368	3103	2799	3147	4922	3907	4641	2578	2450	2445	k__Bacteria;p__Bacillota;c__Bacilli;o__Lactobacillales;f__Lactobacillaceae;g__Liquorilactobacillus;s__Liquorilactobacillus_satsumensis
NCBI2590777	0	0	0	0	0	18	0	0	0	0	0	0	0	0	0	k__Bacteria;p__Pseudomonadota;c__Alphaproteobacteria;o__Hyphomicrobiales;f__Rhizobiaceae;g__Rhizobium;s__Rhizobium_sp._NIBRBAC000502774
NCBI2590869	0	0	0	0	0	11	0	0	0	0	0	2	0	0	0	k__Bacteria;p__Pseudomonadota;c__Betaproteobacteria;o__Burkholderiales;f__Oxalobacteraceae;g__Janthinobacterium;s__Janthinobacterium_tructae

NCBI2591109	0	0	0	0	0	6	0	0	0	0	0	0	0	0	0	k__Bacteria;p__Pseudomonadota;c__Betaproteobacteria;o__;f__Casimicrobiaceae;g__Casimicrobium;s__Casimicrobium_huifangae
NCBI2591145	0	0	0	1	13	5	0	0	0	0	0	0	0	0	0	k__Bacteria;p__Actinomycetota;c__Actinomycetes;o__Micrococcales;f__Cellulomonadaceae;g__Cellulomonas;s__Cellulomonas_sp._Y8
NCBI2591463	0	0	1	2	0	6	1	0	0	0	0	0	0	0	0	k__Bacteria;p__Pseudomonadota;c__Alphaproteobacteria;o__Caulobacterales;f__Caulobacteraceae;g__Brevundimonas;s__Brevundimonas_sp._M20
NCBI2592655	209	248	174	675	504	467	292	219	280	824	698	1064	5679	5420	6906	k__Bacteria;p__Pseudomonadota;c__Alphaproteobacteria;o__Rhodospirillales;f__Acetobacteraceae;g__Acetobacter;s__Acetobacter_vaccinii
NCBI259315	0	13	0	0	0	0	0	5	11	0	0	12	0	0	0	k__Bacteria;p__Bacillota;c__Clostridia;o__Eubacteriales;f__Oscillospiraceae;g__Faecalibacterium;s__uncultured_Faecalibacterium_sp.
NCBI2594003	0	0	0	0	0	1	0	0	0	0	0	0	0	0	0	k__Bacteria;p__Pseudomonadota;c__Alphaproteobacteria;o__Rhodospirillales;f__Rhodospirillaceae;g__Ferrovibrio;s__Ferrovibrio_terrae
NCBI2594269	0	0	0	0	0	1	0	0	0	0	0	0	0	0	0	k__Bacteria;p__Bacteroidota;c__Flavobacteriia;o__Flavobacteriales;f__Weeksellaceae;g__Chryseobacterium;s__Chryseobacterium_sp._SNU_WT5
NCBI2594789	0	0	0	0	0	2	0	0	0	0	0	0	0	0	0	k__Bacteria;p__Bacillota;c__Clostridia;o__Eubacteriales;f__Lachnospiraceae;g__;s__Lachnospiraceae_bacterium_KGMB03038
NCBI2594913	3	0	2	8	6	36	0	0	1	0	0	5	0	0	0	k__Bacteria;p__Actinomycetota;c__Actinomycetes;o__Mycobacteriales;f__Corynebacteriaceae;g__Corynebacterium;s__Corynebacterium_sanguinis
NCBI2596913	0	0	0	0	4	7	1	0	0	0	0	0	0	0	0	k__Bacteria;p__Pseudomonadota;c__Alphaproteobacteria;o__Sphingomonadales;f__Sphingomonadaceae;g__Sphingomonas;s__Sphingomonas_sp._NBWT7

NCBI2596916	0	0	0	0	0	0	1	0	0	0	2	0	0	0	0	k__Bacteria;p__Actinomycetota;c__Actinomycetes;o__Micrococcales;f__Microbacteriaceae;g__Frigoribacterium;s__Frigoribacterium_sp._NBH87
NCBI2596920	0	0	0	0	0	2	0	0	0	0	0	0	0	0	0	k__Bacteria;p__Actinomycetota;c__Actinomycetes;o__Geodermatophilales;f__Geodermatophilaceae;g__;s__Geodermatophilaceae_bacterium_NBWT11
NCBI2596949	13	0	20	14	82	118	10	0	11	9	0	0	10	0	0	k__Bacteria;p__Pseudomonadota;c__Gammaproteobacteria;o__Enterobacterales;f__Enterobacteriaceae;g__Enterobacter;s__Enterobacter_sp._E76
NCBI2597701	0	0	0	0	0	7	0	0	0	0	0	0	0	0	0	k__Bacteria;p__Pseudomonadota;c__Betaproteobacteria;o__Burkholderiales;f__Comamonadaceae;g__Comamonas;s__Comamonas_sp._NLF-7-7
NCBI2597770	0	0	0	0	23	24	0	0	8	0	0	0	0	0	0	k__Bacteria;p__Pseudomonadota;c__Gammaproteobacteria;o__Pseudomonadales;f__Pseudomonadaceae;g__Pseudomonas;s__Pseudomonas_sp._BJP69
NCBI2598457	0	0	0	0	0	2	0	0	0	0	0	0	0	0	0	k__Bacteria;p__Bacillota;c__Bacilli;o__Lactobacillales;f__Streptococcaceae;g__Streptococcus;s__Streptococcus_sp._KS_6
NCBI2599293	0	0	0	0	0	25	0	0	0	0	0	0	7	0	4	k__Bacteria;p__Actinomycetota;c__Actinomycetes;o__Micrococcales;f__Microbacteriaceae;g__Humibacter;s__Humibacter_ginsenosidimutans
NCBI2599297	0	0	0	0	2	35	1	0	0	0	0	0	0	0	0	k__Bacteria;p__Pseudomonadota;c__Alphaproteobacteria;o__Sphingomonadales;f__Sphingomonadaceae;g__Sphingomonas;s__Sphingomonas_suaedae
NCBI259951	3	0	3	0	0	0	0	2	7	8	6	7	11	0	0	k__Bacteria;p__Cyanobacteriota;c__Cyanophyceae;o__Oscillatoriales;f__Cyanothecaceae;g__Cyanothece;s__uncultured_Cyanothece_sp.
NCBI2599640	0	0	0	0	0	2	0	0	0	0	0	0	0	0	0	k__Bacteria;p__Pseudomonadota;c__Alphaproteobacteria;o__Hyphomicrobiales;f__Boseaceae;g__Bosea;s__Bosea_sp._F3-2

NCBI260090	0	0	0	0	0	12	0	0	0	0	0	0	0	0	0	k__Fungi;p__Mucoromycota;c__Glomeromycetes;o__Glomerales;f__Glomeraceae;g__;s__uncultured_glomeraceous_AM_fungus
NCBI2601122	0	0	0	0	0	0	1	0	0	0	0	0	0	0	0	k__Bacteria;p__Pseudomonadota;c__Gammaproteobacteria;o__Moraxellales;f__Moraxellaceae;g__Acinetobacter;s__Acinetobacter_sp._YH12138
NCBI2601609	0	0	0	0	1	0	0	0	0	0	0	0	0	0	0	k__Heunggongvirae;p__Uroviricota;c__Caudoviricetes;o__;f__;g__;s__Bordetella_phage_vB_BbrS_PHB09
NCBI2602016	0	0	0	0	0	2	0	0	0	0	0	0	1	0	0	k__Bacteria;p__Pseudomonadota;c__Alphaproteobacteria;o__Rhodospirillales;f__Rhodospirillaceae;g__Hypericibacter;s__Hypericibacter_adhaerens
NCBI2603206	0	0	0	1	0	0	0	0	0	0	0	0	0	0	0	k__Bacteria;p__Actinomycetota;c__Actinomycetes;o__Micrococcales;f__Ruaniaceae;g__Ruania;s__Ruania_zhangjianzhongii
NCBI2603276	0	0	0	1	1	0	0	0	0	0	0	0	0	0	0	k__Bacteria;p__Pseudomonadota;c__Alphaproteobacteria;o__Hyphomicrobiales;f__Methylobacteriaceae;g__Methylobacterium;s__Methylobacterium_sp._WL1
NCBI2603277	53	47	16	5	110	1762	68	1	11	12	11	31	10	4	15	k__Bacteria;p__Pseudomonadota;c__Alphaproteobacteria;o__Hyphomicrobiales;f__Rhizobiaceae;g__Rhizobium;s__Rhizobium_sp._WL3
NCBI260364	0	0	0	0	0	0	0	0	0	0	0	1	0	0	0	k__Bacteria;p__Pseudomonadota;c__Gammaproteobacteria;o__Alteromonadales;f__Shewanellaceae;g__Shewanella;s__Shewanella_marisflavi
NCBI2604832	0	0	0	0	0	7	0	0	1	0	0	0	0	0	0	k__Bacteria;p__Pseudomonadota;c__Gammaproteobacteria;o__Pseudomonadales;f__Pseudomonadaceae;g__Pseudomonas;s__Pseudomonas_lalkuanensis
NCBI2605424	0	0	0	3	2	0	0	0	0	0	0	0	0	0	3	k__Bacteria;p__Pseudomonadota;c__Gammaproteobacteria;o__Pseudomonadales;f__Pseudomonadaceae;g__Pseudomonas;s__Pseudomonas_sp._J380

NCBI2605946	0	0	0	0	0	8	0	0	0	0	0	0	0	0	0	k__Bacteria;p__Pseudomonadota;c__Alphaproteobacteria;o__Rhodobacterales;f__Paracoccaceae;g__Pukyongiella;s__Pukyongiella_litopenaei
NCBI2606451	0	0	0	0	0	9	0	0	0	0	0	0	0	0	0	k__Bacteria;p__Actinomycetota;c__Actinomycetes;o__Micrococcales;f__Microbacteriaceae;g__Microbacterium;s__Microbacterium_sp._1S1
NCBI2606626	0	0	0	0	1	1	3	0	0	0	1	2	0	0	0	k__Bacteria;p__Bacteroidota;c__Bacteroidia;o__Bacteroidales;f__Muribaculaceae;g__Sodaliphilus;s__Sodaliphilus_pleomorphus
NCBI2609668	0	1	0	0	0	14	1	0	0	0	0	0	3	0	0	k__Bacteria;p__Pseudomonadota;c__Gammaproteobacteria;o__Moraxellales;f__Moraxellaceae;g__Acinetobacter;s__Acinetobacter_sp._C16S1
NCBI2610895	0	0	2	0	0	9	0	0	0	0	0	0	0	0	0	k__Bacteria;p__Bacillota;c__Clostridia;o__Eubacteriales;f__;g__Flintibacter;s__Flintibacter_sp._KGMB00164
NCBI2610896	0	0	0	0	2	0	0	0	0	0	0	12	0	0	0	k__Bacteria;p__Bacillota;c__Bacilli;o__Lactobacillales;f__Streptococcaceae;g__Streptococcus;s__Streptococcus_sp._LPB0220
NCBI261098	0	0	0	0	0	1	0	0	0	0	0	0	0	0	0	k__Fungi;p__Chytridiomycota;c__Chytridiomycetes;o__Chytridiales;f__Chytridiaceae;g__Phlyctochytrium;s__Phlyctochytrium_planicorne
NCBI2613334	0	0	0	0	2	0	0	0	0	0	0	0	0	0	0	k__Bacteria;p__Bacillota;c__Bacilli;o__Bacillales;f__Bacillaceae;g__Anoxybacillus;s__Anoxybacillus_sediminis
NCBI2613770	0	0	1	0	0	5	0	0	0	0	0	0	0	0	0	k__Bacteria;p__Pseudomonadota;c__Alphaproteobacteria;o__Hyphomicrobiales;f__Rhizobiaceae;g__Rhizobium;s__Rhizobium_sp._BG4
NCBI2614442	0	0	0	0	0	0	0	0	0	0	0	2	0	0	0	k__Bacteria;p__Pseudomonadota;c__Gammaproteobacteria;o__Pseudomonadales;f__Pseudomonadaceae;g__Pseudomonas;s__Pseudomonas_sp._LPB0260
NCBI262209	2	0	0	8	0	8	0	0	0	0	0	0	0	0	0	k__Bacteria;p__Actinomycetota;c__Actinomycetes;o__Micrococcales;f__Intrasporangiaceae;g__Janibacter;s__J

																anibacter_melonis
NCBI262404	0	0	0	0	0	6	0	0	2	0	0	0	0	0	0	k__Bacteria;p__Bacillota;c__Clostridia;o__Halanaerobiales;f__Halanaerobiaceae;g__Halanaerobium;s__uncultured_Halanaerobium_sp.
NCBI264951	0	0	0	0	0	1	0	0	0	0	0	0	0	0	0	k__Fungi;p__Ascomycota;c__Eurotiomycetes;o__Eurotiales;f__Thermoascaceae;g__Paecilomyces;s__Paecilomyces_variotii
NCBI2650157	0	0	1	0	0	13	2	0	0	0	0	7	0	0	0	k__Bacteria;p__Bacteroidota;c__Bacteroidia;o__Bacteroidales;f__Bacteroidaceae;g__Bacteroides;s__Bacteroides_zhangwenhongii
NCBI2650158	0	0	0	0	0	1	2	0	0	0	0	0	0	0	0	k__Bacteria;p__Bacteroidota;c__Bacteroidia;o__Bacteroidales;f__Bacteroidaceae;g__Bacteroides;s__Bacteroides_luhongzhouii
NCBI2651048	0	0	0	0	0	8	0	0	0	0	0	0	0	0	0	k__Bacteria;p__Pseudomonadota;c__Gammaproteobacteria;o__Pseudomonadales;f__Pseudomonadaceae;g__Pseudomonas;s__Pseudomonas_sp._CFA
NCBI2651974	1	1	0	0	1	44	2	0	0	0	0	0	0	0	0	k__Bacteria;p__Pseudomonadota;c__Betaproteobacteria;o__Burkholderiales;f__Comamonadaceae;g__Hydrogenophaga;s__Hydrogenophaga_sp._BPS33
NCBI2652173	0	0	0	0	0	0	0	0	1	0	0	0	0	0	0	k__Bacteria;p__Nitrospirota;c__Nitrospiria;o__Nitrospirales;f__Nitrospiraceae;g__Nitrospira;s__Candidatus_Nitrospira_kreftii
NCBI2653203	0	1	0	0	0	10	0	0	0	0	0	0	0	1	0	k__Bacteria;p__Pseudomonadota;c__Alphaproteobacteria;o__Sphingomonadales;f__Sphingomonadaceae;g__Sphingomonas;s__Sphingomonas_sp._CL5.1
NCBI2653851	0	0	0	1	0	310	0	0	0	0	0	0	0	0	0	k__Bacteria;p__Actinomycetota;c__Rubrobacteria;o__Rubrobacterales;f__Rubrobacteraceae;g__Rubrobacter;s__Rubrobacter_tropicus

NCBI2653852	0	0	0	0	0	11	0	0	0	0	0	0	0	0	0	k__Bacteria;p__Actinomycetota;c__Rubrobacteria;o__Rubrobacterales;f__Rubrobacteraceae;g__Rubrobacter;s__Rubrobacter_marinus
NCBI2654218	2	1	0	0	5	16	0	0	0	0	0	0	0	0	0	k__Bacteria;p__Pseudomonadota;c__Betaproteobacteria;o__Rhodocyclales;f__Azonexaceae;g__Dechloromonas;s__Dechloromonas_sp._TW-R-39-2
NCBI2654982	0	0	0	0	1	0	0	0	0	0	0	0	0	0	0	k__Bacteria;p__Pseudomonadota;c__Betaproteobacteria;o__Burkholderiales;f__Burkholderiaceae;g__Paraburkholderia;s__Paraburkholderia_atlantica
NCBI265959	781	799	866	2437	2008	1583	1150	760	1078	3834	3690	5547	16636	15981	19737	k__Bacteria;p__Pseudomonadota;c__Alphaproteobacteria;o__Rhodospirillales;f__Acetobacteraceae;g__Komagataeibacter;s__Komagataeibacter_saccharivorans
NCBI265960	13	28	26	280	332	331	31	43	31	112	89	138	164	178	227	k__Bacteria;p__Pseudomonadota;c__Alphaproteobacteria;o__Rhodospirillales;f__Acetobacteraceae;g__Komagataeibacter;s__Komagataeibacter_nataicola
NCBI2660750	0	24	0	0	0	0	0	0	0	0	0	0	0	0	0	k__Bacteria;p__Bacillota;c__Bacilli;o__Bacillales;f__Bacillaceae;g__Bacillus;s__Bacillus_sp._A260
NCBI2662033	0	0	0	9	14	10	2	0	0	0	0	0	0	0	3	k__Bacteria;p__Pseudomonadota;c__Gammaproteobacteria;o__Pseudomonadales;f__Pseudomonadaceae;g__Pseudomonas;s__Pseudomonas_sp._NY5710
NCBI2662034	0	0	0	0	0	22	0	0	0	0	0	0	0	0	0	k__Bacteria;p__Pseudomonadota;c__Gammaproteobacteria;o__Pseudomonadales;f__Pseudomonadaceae;g__Pseudomonas;s__Pseudomonas_sp._13159349
NCBI2662179	2	9	4	37	92	153	6	0	5	0	0	0	3	0	1	k__Bacteria;p__Pseudomonadota;c__Gammaproteobacteria;o__Enterobacterales;f__Enterobacteriaceae;g__;s__Enterobacteriaceae_bacterium_Kacie_13
NCBI2662362	0	0	0	0	0	0	2	0	0	0	0	0	0	0	0	k__Bacteria;p__Pseudomonadota;c__Gammaproteobacteria;o__Moraxellales;f__Moraxellaceae;g__Acinetobacter;s__Acinetobacter_wanghuae

NCBI2662363	0	0	0	0	0	0	0	0	0	0	0	2	0	0	0	k__Bacteria;p__Bacteroidota;c__Bacteroidia;o__Bacteroidales;f__Rikenellaceae;g__Alistipes;s__Alistipes_sp._dk3624
NCBI2663009	0	0	0	0	0	0	1	2	0	0	0	0	0	0	0	k__Bacteria;p__Fusobacteriota;c__Fusobacteriia;o__Fusobacteriales;f__Fusobacteriaceae;g__Fusobacterium;s__Fusobacterium_pseudoperiodonticum
NCBI2663121	0	0	0	0	0	1	0	0	0	0	0	0	0	0	0	k__Bacteria;p__Actinomycetota;c__Actinomycetes;o__Mycobacteriales;f__Nocardiaceae;g__Rhodococcus;s__Rhodococcus_sp._WAY2
NCBI2663857	0	0	4	0	0	0	0	0	0	0	0	0	0	0	0	k__Bacteria;p__Actinomycetota;c__Actinomycetes;o__Propionibacteriales;f__Nocardioidaceae;g__Nocardioides;s__Nocardioides_sp._zg-579
NCBI2664893	74	86	43	0	192	2948	88	5	27	12	18	33	18	16	11	k__Bacteria;p__Pseudomonadota;c__Alphaproteobacteria;o__Hyphomicrobiales;f__Rhizobiaceae;g__Agrobacterium;s__Agrobacterium_sp._MA01
NCBI2665642	0	0	0	2	1	53	0	0	0	0	0	0	0	0	0	k__Bacteria;p__Actinomycetota;c__Actinomycetes;o__Pseudonocardiales;f__Pseudonocardiaceae;g__Allosaccharopolyspora;s__Allosaccharopolyspora_coralli
NCBI2666139	0	0	0	0	0	0	0	0	0	0	0	13	0	0	0	k__Bacteria;p__Pseudomonadota;c__Alphaproteobacteria;o__Hyphomicrobiales;f__Phyllobacteriaceae;g__Aminobacter;s__Aminobacter_sp._MDW-2
NCBI266831	0	0	0	0	0	51	0	0	0	0	0	0	0	0	0	k__Bacteria;p__Pseudomonadota;c__Betaproteobacteria;o__Burkholderiales;f__Comamonadaceae;g__Diaphorobacter;s__Diaphorobacter_sp._PCA039
NCBI267128	5	0	0	0	0	75	0	0	0	0	0	0	0	0	0	k__Bacteria;p__Pseudomonadota;c__Alphaproteobacteria;o__Sphingomonadales;f__Sphingomonadaceae;g__Sphingopyxis;s__Sphingopyxis_granuli
NCBI267212	0	1	0	0	0	0	1	0	0	0	0	0	0	0	0	k__Bacteria;p__Pseudomonadota;c__Betaproteobacteria;o__Neisseriales;f__Neisseriaceae;g__Neisseria;s__Neisseria_bacilliformis

NCBI2675225	0	0	0	0	0	2	0	0	0	0	0	0	0	0	0	k__Bacteria;p__Pseudomonadota;c__Alphaproteobacteria;o__Sphingomonadales;f__Sphingomonadaceae;g__Novosphingobium;s__Novosphingobium_sp._Gsoil_351
NCBI2675378	0	0	0	0	0	0	0	0	0	0	190	22	0	0	0	k__Bacteria;p__Pseudomonadota;c__Gammaproteobacteria;o__Enterobacterales;f__Erwiniaceae;g__Erwinia;s__Erwinia_sp._E602
NCBI2676062	0	0	0	0	0	0	0	0	0	0	0	1	0	0	0	k__Bacteria;p__Bacillota;c__Erysipelotrichia;o__Erysipelotrichales;f__Erysipelotrichaceae;g__;s__Erysipelotrichaceae_bacterium_66202529
NCBI2676077	2	2	2	2	12	182	7	0	0	0	2	2	0	0	0	k__Bacteria;p__Pseudomonadota;c__Alphaproteobacteria;o__Sphingomonadales;f__Sphingomonadaceae;g__Sphingobium;s__Sphingobium_sp._CAP-1
NCBI2676834	0	0	0	0	28	10	0	0	0	0	0	0	0	0	0	k__Bacteria;p__Pseudomonadota;c__Gammaproteobacteria;o__Enterobacterales;f__Erwiniaceae;g__Pantoea;s__Pantoea_jilinensis
NCBI2676868	0	0	0	0	0	1	1	0	0	0	0	0	0	1	6	k__Bacteria;p__Bacteroidota;c__Chitinophagia;o__Chitinophagales;f__Chitinophagaceae;g__Phnomibacter;s__Phnomibacter_ginsenosidimutans
NCBI267818	20	19	24	47	49	31	31	27	12	15	14	19	2	2	5	k__Bacteria;p__Bacillota;c__Bacilli;o__Lactobacillales;f__Lactobacillaceae;g__Lactobacillus;s__Lactobacillus_kefiranofaciens
NCBI2681308	0	0	0	7	6	7	7	0	0	0	0	0	0	0	0	k__Bacteria;p__Pseudomonadota;c__Gammaproteobacteria;o__Enterobacterales;f__Enterobacteriaceae;g__Leclercia;s__Leclercia_sp._119287
NCBI2681547	0	0	0	0	0	0	0	0	0	0	0	0	0	1	0	k__Bacteria;p__Pseudomonadota;c__Gammaproteobacteria;o__Cellvibrionales;f__Microbulbiferaceae;g__Microbulbifer;s__Microbulbifer_sp._SH-1
NCBI2681552	0	0	0	0	0	5	0	0	0	0	0	0	0	0	0	k__Bacteria;p__Pseudomonadota;c__Betaproteobacteria;o__Burkholderiales;f__Comamonadaceae;g__Variovorax;s__Variovorax_sp._RKNM96

NCBI2681674	0	0	0	0	0	0	0	0	0	0	0	9	0	0	0	k__Heunggongvirae;p__Uroviricota;c__Caudoviricetes;o__;f__Guernseyvirinae;g__Kagunavirus;s__Escherichia_phage_vB_EcoS_XY1
NCBI2682455	0	0	0	0	0	1	0	2	0	0	0	0	0	0	0	k__Bacteria;p__Bacillota;c__Negativicutes;o__Veillonellales;f__Veillonellaceae;g__Veillonella;s__Veillonella_sp._S12025-13
NCBI2682456	0	0	0	0	0	0	0	0	0	0	0	5	0	0	0	k__Bacteria;p__Bacillota;c__Negativicutes;o__Veillonellales;f__Veillonellaceae;g__Veillonella;s__Veillonella_nakazawae
NCBI2682487	0	26	23	281	378	460	0	0	0	0	0	22	0	0	0	k__Bacteria;p__Pseudomonadota;c__Gammaproteobacteria;o__Xanthomonadales;f__Xanthomonadaceae;g__Stenotrophomonas;s__Stenotrophomonas_sp._SXG-1
NCBI2686385	0	0	0	0	0	1	0	0	0	0	0	0	0	0	0	k__Heunggongvirae;p__Uroviricota;c__Caudoviricetes;o__;f__;g__;s__Lactobacillus_phage_JNU_P7
NCBI2686386	5	5	0	0	0	10	10	15	14	35	23	5	0	0	0	k__Heunggongvirae;p__Uroviricota;c__Caudoviricetes;o__;f__;g__Sukhumvitvirus;s__Lactobacillus_phage_JNU_P9
NCBI2690380	0	0	0	0	0	0	0	0	0	0	0	0	0	10	0	k__Bacteria;p__Bacillota;c__Bacilli;o__Lactobacillales;f__Lactobacillaceae;g__Lactobacillus;s__Lactobacillus_sp._JM1
NCBI2691571	0	0	0	0	0	1	0	0	0	0	0	0	0	0	0	k__Bacteria;p__Pseudomonadota;c__Gammaproteobacteria;o__Xanthomonadales;f__Xanthomonadaceae;g__Stenotrophomonas;s__Stenotrophomonas_sp._364
NCBI2691580	0	0	0	0	0	4	0	0	0	0	0	0	0	0	0	k__Bacteria;p__Bacteroidota;c__Bacteroidia;o__Bacteroidales;f__Prevotellaceae;g__Prevotella;s__Prevotella_sp._Rep29
NCBI269660	0	8	0	21	14	90	4	0	0	0	0	22	7	0	6	k__Bacteria;p__Pseudomonadota;c__Alphaproteobacteria;o__Hyphomicrobiales;f__Methylobacteriaceae;g__Methylobacterium;s__Methylobacterium_brachiatum

NCBI2697019	0	0	0	0	0	2	0	0	0	0	0	0	0	0	0	k__Bacteria;p__Pseudomonadota;c__Gammaproteobacteria;o__Enterobacterales;f__Morganellaceae;g__Proteus;s__Proteus_sp._ZN5
NCBI2697023	2	2	0	0	7	24	2	0	0	0	0	0	0	5	0	k__Bacteria;p__Pseudomonadota;c__Gammaproteobacteria;o__Pseudomonadales;f__Pseudomonadaceae;g__Pseudomonas;s__Pseudomonas_sp._AN-B15
NCBI2697032	0	0	0	0	0	5	0	0	0	0	0	0	0	0	0	k__Bacteria;p__Pseudomonadota;c__Betaproteobacteria;o__Burkholderiales;f__;g__Xylophilus;s__Xylophilus_rhododendri
NCBI2697565	0	0	0	2	0	1	0	0	1	0	0	0	0	0	0	k__Bacteria;p__Actinomycetota;c__Actinomycetes;o__Micrococcales;f__Brevibacteriaceae;g__Brevibacterium;s__Brevibacterium_limosum
NCBI2698673	11	2	2	0	11	186	13	0	0	2	0	5	0	0	0	k__Bacteria;p__Pseudomonadota;c__Alphaproteobacteria;o__Sphingomonadales;f__Sphingomonadaceae;g__Novosphingobium;s__Novosphingobium_decolorationis
NCBI2698679	0	0	0	0	0	1	0	0	0	0	0	0	0	0	0	k__Bacteria;p__Pseudomonadota;c__Alphaproteobacteria;o__Sphingomonadales;f__Sphingomonadaceae;g__Sphingomonas;s__Sphingomonas_changnyeongensis
NCBI2698682	0	0	0	1	0	1	0	0	0	0	0	0	0	0	0	k__Bacteria;p__Pseudomonadota;c__Gammaproteobacteria;o__Xanthomonadales;f__Xanthomonadaceae;g__Lysobacter;s__Lysobacter_oculi
NCBI2698684	0	0	0	0	0	9	0	0	0	0	0	3	0	0	0	k__Bacteria;p__Pseudomonadota;c__Gammaproteobacteria;o__Nevskiales;f__Sinobacteraceae;g__Sinimarinibacterium;s__Sinimarinibacterium_sp._NLF-5-8
NCBI2698900	0	2	0	0	0	0	0	0	0	0	0	0	0	0	0	k__Bacteria;p__Actinomycetota;c__Actinomycetes;o__Mycobacteriales;f__Gordoniaceae;g__Gordonia;s__Gordonia_sp._JH63
NCBI2702	0	0	1	0	0	7	0	0	0	0	0	2	0	0	2	k__Bacteria;p__Actinomycetota;c__Actinomycetes;o__Bifidobacteriales;f__Bifidobacteriaceae;g__Gardnerella;s__Gardnerella_vaginalis

NCBI270351	2	0	0	4	6	45	4	0	1	0	0	3	0	0	6	k__Bacteria;p__Pseudomonadota;c__Alphaproteobacteria;o__Hyphomicrobiales;f__Methylobacteriaceae;g__Methylobacterium;s__Methylobacterium_aquaticum
NCBI2703885	0	0	0	11	19	0	0	0	0	0	0	0	0	0	0	k__Bacteria;p__Pseudomonadota;c__Gammaproteobacteria;o__Enterobacterales;f__Yersiniaceae;g__Rahnella;s__Rahnella_aceris
NCBI2704465	0	0	0	0	0	3	0	0	0	0	0	0	0	0	0	k__Bacteria;p__Bacteroidota;c__Cytophagia;o__Cytophagales;f__Cytophagaceae;g__Rhodocytophaga;s__Rhodocytophaga_rosea
NCBI2705472	1	2	0	3	11	47	0	0	1	0	0	0	0	0	0	k__Bacteria;p__Pseudomonadota;c__Gammaproteobacteria;o__Pseudomonadales;f__Pseudomonadaceae;g__Pseudomonas;s__Pseudomonas_sp._MTM4
NCBI2706126	0	0	0	0	1	3	0	0	0	0	0	1	0	0	0	k__Bacteria;p__Pseudomonadota;c__Gammaproteobacteria;o__Pseudomonadales;f__Pseudomonadaceae;g__Pseudomonas;s__Pseudomonas_sp._OIL-1
NCBI270673	0	0	0	0	0	18	0	0	0	0	0	0	0	0	0	k__Heunggongvirae;p__Uroviricota;c__Caudoviricetes;o__;f__;g__Jamesmcgillvirus;s__Pseudomonas_phage_PaP2
NCBI2707299	0	0	0	0	0	1	0	0	0	0	0	1	0	0	0	k__Bacteria;p__Actinomycetota;c__Coriobacteriia;o__Eggerthellales;f__Eggerthellaceae;g__Adlercreutzia;s__Adlercreutzia_hattorii
NCBI2708117	0	0	0	1	0	0	0	1	0	0	0	0	0	0	0	k__Bacteria;p__Bacteroidota;c__Flavobacteriia;o__Flavobacteriales;f__Flavobacteriaceae;g__Capnocytophaga;s__Capnocytophaga_endodontalis
NCBI2708348	0	0	3	0	0	0	0	0	0	0	0	0	0	0	0	k__Bacteria;p__Pseudomonadota;c__Gammaproteobacteria;o__Moraxellales;f__Moraxellaceae;g__Acinetobacter;s__Acinetobacter_sp._WY4
NCBI2708351	0	0	0	0	0	60	9	0	0	0	0	22	0	0	0	k__Bacteria;p__Pseudomonadota;c__Alphaproteobacteria;o__Hyphomicrobiales;f__Brucellaceae;g__Ochrobactrum;s__Ochrobactrum_sp._WY7

NCBI2708352	0	0	0	0	0	1379	0	0	0	0	0	0	0	495	0	k__Bacteria;p__Pseudomonadota;c__Betaproteobacteria;o__Burkholderiales;f__Comamonadaceae;g__Delftia;s__Delftia_sp._WY8
NCBI2708539	0	0	0	0	3	4	0	0	0	0	0	0	0	0	0	k__Bacteria;p__Pseudomonadota;c__Alphaproteobacteria;o__Caulobacterales;f__Caulobacteraceae;g__Caulobacter;s__Caulobacter_soli
NCBI2709410	0	0	0	0	0	1	0	0	0	0	0	0	0	0	0	k__Bacteria;p__Bacillota;c__Clostridia;o__Eubacteriales;f__Lachnospiraceae;g__Chordicoccus;s__Chordicoccus_furentiruminis
NCBI271	0	0	0	0	0	0	0	0	0	0	0	1	0	0	0	k__Bacteria;p__Deinococcota;c__Deinococci;o__Thermales;f__Thermaceae;g__Thermus;s__Thermus_aquaticus
NCBI2711215	0	0	0	0	0	1	0	0	0	0	0	0	0	0	0	k__Bacteria;p__Pseudomonadota;c__Alphaproteobacteria;o__Sphingomonadales;f__Sphingomonadaceae;g__Sphingosinithalassobacter;s__Sphingosinithalassobacter_tenebrarum
NCBI2712223	0	0	0	0	0	12	0	0	0	0	0	0	0	0	0	k__Bacteria;p__Actinomycetota;c__Actinomycetes;o__Propionibacteriales;f__Nocardioidaceae;g__Nocardioides;s__Nocardioides_anomalus
NCBI2712698	0	1	1	1	3	22	0	0	0	0	0	0	0	1	0	k__Bacteria;p__Pseudomonadota;c__Alphaproteobacteria;o__Hyphomicrobiales;f__Phyllobacteriaceae;g__Salaquimonas;s__Salaquimonas_pukyongi
NCBI2713573	0	0	0	0	1	1	0	0	0	0	0	0	0	0	0	k__Bacteria;p__Bacteroidota;c__Sphingobacteriia;o__Sphingobacteriales;f__Sphingobacteriaceae;g__Sphingobacterium;s__Sphingobacterium_sp._DR205
NCBI2714110	0	0	0	0	0	2	0	0	0	0	0	0	0	0	0	k__Bacteria;p__Pseudomonadota;c__Gammaproteobacteria;o__Moraxellales;f__Moraxellaceae;g__Acinetobacter;s__Acinetobacter_sp._Marseille-Q1620
NCBI271420	0	0	0	0	0	10	0	0	0	0	0	0	0	0	0	k__Bacteria;p__Pseudomonadota;c__Gammaproteobacteria;o__Pseudomonadales;f__Pseudomonadaceae;g__Stutzerimonas;s__Stutzerimonas_xanthomarina

NCBI2714353	0	0	0	0	0	1	0	0	0	0	0	0	0	0	0	k__Bacteria;p__Bacillota;c__Clostridia;o__Eubacteriales;f__Oscillospiraceae;g__Vescimonas;s__Vescimonas_fastidiosa
NCBI2714355	0	0	1	3	0	72	4	0	0	1	0	16	0	0	0	k__Bacteria;p__Bacillota;c__Clostridia;o__Eubacteriales;f__Oscillospiraceae;g__Vescimonas;s__Vescimonas_coprocola
NCBI2714358	0	0	0	0	1	1	0	0	0	0	0	2	0	0	0	k__Bacteria;p__Bacillota;c__Clostridia;o__Eubacteriales;f__Oscillospiraceae;g__Pusillibacter;s__Pusillibacter_faecalis
NCBI2714923	0	0	0	0	0	13	0	0	0	0	0	0	0	0	0	k__Bacteria;p__Pseudomonadota;c__Betaproteobacteria;o__Burkholderiales;f__Comamonadaceae;g__Acidovorax;s__Acidovorax_sp._HDW3
NCBI2714924	3	9	2	0	9	178	6	0	3	0	0	0	0	0	4	k__Bacteria;p__Pseudomonadota;c__Betaproteobacteria;o__Burkholderiales;f__Comamonadaceae;g__Diaphorobacter;s__Diaphorobacter_sp._HDW4A
NCBI2714925	0	0	0	0	0	1	0	0	0	0	0	0	0	0	0	k__Bacteria;p__Pseudomonadota;c__Betaproteobacteria;o__Burkholderiales;f__Comamonadaceae;g__Diaphorobacter;s__Diaphorobacter_sp._HDW4B
NCBI2714938	0	0	0	13	0	0	0	0	0	0	0	0	0	0	0	k__Bacteria;p__Actinomycetota;c__Actinomycetes;o__Propionibacteriales;f__Nocardioidaceae;g__Nocardioides;s__Nocardioides_piscis
NCBI2714939	0	0	2	0	0	9	0	0	0	0	1	0	0	0	0	k__Bacteria;p__Actinomycetota;c__Actinomycetes;o__Propionibacteriales;f__Nocardioidaceae;g__Nocardioides;s__Nocardioides_sp._HDW12B
NCBI2714948	0	0	0	0	0	2	0	0	0	1	0	0	0	0	0	k__Bacteria;p__Pseudomonadota;c__Gammaproteobacteria;o__Vibrionales;f__Vibrionaceae;g__Vibrio;s__Vibrio_sp._HDW18
NCBI2714951	0	0	0	0	0	1	0	0	0	0	0	0	0	0	0	k__Bacteria;p__Pseudomonadota;c__Gammaproteobacteria;o__Enterobacterales;f__Enterobacteriaceae;g__Leclercia;s__Leclercia_sp._29361

NCBI2714952	0	7	0	0	1	54	0	0	0	0	0	1	0	0	2	k__Bacteria;p__Pseudomonadota;c__Betaproteobacteria;o__Burkholderiales;f__Comamonadaceae;g__Kinneretia;s__Kinneretia_sp._DAIF2
NCBI2715212	0	0	0	0	6	3	30	1	7	0	0	1	0	0	0	k__Bacteria;p__Bacteroidota;c__Bacteroidia;o__Bacteroidales;f__Bacteroidaceae;g__Bacteroides;s__Bacteroides_faecium
NCBI2715959	8	7	6	2	15	253	8	0	7	0	9	6	0	1	2	k__Bacteria;p__Pseudomonadota;c__Alphaproteobacteria;o__Hyphomicrobiales;f__Rhizobiaceae;g__Shinella;s__Shinella_sp._PSBB067
NCBI2715960	40	45	42	60	130	685	55	9	14	39	23	50	59	48	110	k__Bacteria;p__Pseudomonadota;c__Alphaproteobacteria;o__Hyphomicrobiales;f__Nitrobacteraceae;g__Bradyrhizobium;s__Bradyrhizobium_sp._PSBB068
NCBI2716225	5	5	0	0	8	102	15	0	2	0	4	6	0	0	0	k__Bacteria;p__Pseudomonadota;c__Betaproteobacteria;o__Burkholderiales;f__Comamonadaceae;g__Hydrogenophaga;s__Hydrogenophaga_crocea
NCBI2718	1	0	0	0	0	0	0	0	0	0	0	0	0	0	0	k__Bacteria;p__Pseudomonadota;c__Gammaproteobacteria;o__Cardiobacteriales;f__Cardiobacteriaceae;g__Cardiobacterium;s__Cardiobacterium_hominis
NCBI271865	0	1	0	0	2	3	9	0	0	0	0	0	0	0	0	k__Bacteria;p__Pseudomonadota;c__Alphaproteobacteria;o__Hyphomicrobiales;f__Brucellaceae;g__Brucella;s__[Ochrobactrum]_quorumnocens
NCBI271881	4	0	0	0	0	0	0	0	0	0	0	0	0	0	0	k__Bacteria;p__Bacillota;c__Bacilli;o__Lactobacillales;f__Lactobacillaceae;g__Lactiplantibacillus;s__Lactiplantibacillus_argentoratensis
NCBI2719588	0	0	0	2	0	2	0	0	0	0	0	0	0	0	0	k__Bacteria;p__Actinomycetota;c__Actinomycetes;o__Micrococcales;f__Micrococcaceae;g__Kocuria;s__Kocuria_sp._KD4
NCBI2720029	0	0	0	7	0	0	0	0	0	0	0	0	0	0	0	k__Bacteria;p__Pseudomonadota;c__Gammaproteobacteria;o__Enterobacterales;f__Enterobacteriaceae;g__Enterobacter;s__Enterobacter_sp._DNB-S2

NCBI272131	0	0	0	0	0	3	0	0	0	0	0	0	0	0	0	k__Bacteria;p__Cyanobacteriota;c__Cyanophyceae;o__Nostocales;f__Nostocaceae;g__Nostoc;s__Nostoc_punctiforme
NCBI2722752	0	0	0	0	7	41	0	0	0	0	0	0	0	0	0	k__Bacteria;p__Actinomycetota;c__Acidimicrobiia;o__Acidimicrobiales;f__Iamiaceae;g__Iamia;s__Iamia_sp._SCSIO_61187
NCBI2724122	0	0	0	0	10	0	0	0	0	0	0	0	0	0	0	k__Bacteria;p__Pseudomonadota;c__Gammaproteobacteria;o__Xanthomonadales;f__Xanthomonadaceae;g__Xanthomonas;s__Xanthomonas_sp._SS
NCBI2724123	0	0	0	0	5	6	0	0	0	0	0	0	0	0	0	k__Bacteria;p__Pseudomonadota;c__Gammaproteobacteria;o__Xanthomonadales;f__Xanthomonadaceae;g__Xanthomonas;s__Xanthomonas_sp._SI
NCBI2724468	0	0	0	1	0	0	9	0	0	0	0	0	0	0	0	k__Bacteria;p__Pseudomonadota;c__Gammaproteobacteria;o__Enterobacterales;f__Enterobacteriaceae;g__Enterobacter;s__Enterobacter_sp._JUb54
NCBI2724470	0	0	0	0	2	4	0	0	0	0	0	0	0	0	0	k__Bacteria;p__Pseudomonadota;c__Gammaproteobacteria;o__Pseudomonadales;f__Pseudomonadaceae;g__Pseudomonas;s__Pseudomonas_sp._BIGb0427
NCBI2724619	0	0	4	0	1	0	0	0	0	0	0	0	1	0	0	k__Bacteria;p__Bacteroidota;c__Flavobacteriia;o__Flavobacteriales;f__Weeksellaceae;g__Chryseobacterium;s__Chryseobacterium_sp._NEB161
NCBI2725477	2	0	0	0	0	3	0	0	0	0	0	0	0	0	0	k__Bacteria;p__Pseudomonadota;c__Gammaproteobacteria;o__Pseudomonadales;f__Pseudomonadaceae;g__Pseudomonas;s__Pseudomonas_tohonis
NCBI2725666	0	0	0	0	6	44	4	0	0	1	0	0	0	2	3	k__Bacteria;p__Pseudomonadota;c__Alphaproteobacteria;o__Hyphomicrobiales;f__Phyllobacteriaceae;g__Mesorhizobium;s__Mesorhizobium_terrae
NCBI2726427	3	34	7	0	31	637	12	0	3	0	0	6	5	0	3	k__Bacteria;p__Pseudomonadota;c__Alphaproteobacteria;o__Hyphomicrobiales;f__Brucellaceae;g__Ochrobactrum;s__Ochrobactrum_sp._MT180101

NCBI2726956	0	0	0	0	0	2	0	0	0	0	0	0	0	0	0	k__Bacteria;p__Pseudomonadota;c__Gammaproteobacteria;o__Pseudomonadales;f__Pseudomonadaceae;g__Pseudomonas;s__Pseudomonas_sp._MSPm1
NCBI2728020	0	0	0	0	0	19	0	0	0	0	0	0	0	0	0	k__Bacteria;p__Pseudomonadota;c__Betaproteobacteria;o__Burkholderiales;f__Oxalobacteraceae;g__Massilia;s__Massilia_forsythiae
NCBI2728827	0	0	0	0	8	0	0	0	0	0	0	0	0	0	0	k__Bacteria;p__Actinomycetota;c__Actinomycetes;o__Micromonosporales;f__Micromonosporaceae;g__Catellatospora;s__Catellatospora_sp._IY07-71
NCBI2728875	0	0	0	0	0	2	0	0	0	0	0	0	0	0	0	k__Bacteria;p__Pseudomonadota;c__Alphaproteobacteria;o__Rhodospirillales;f__Rhodospirillaceae;g__;s__Rhodospirillaceae_bacterium_B3
NCBI27291	50465	55578	51542	36875	29563	16265	42319	46857	48322	38272	31722	33472	9715	9491	9394	k__Fungi;p__Ascomycota;c__Saccharomycetes;o__Saccharomycetales;f__Saccharomycetaceae;g__Saccharomyces;s__Saccharomyces_paradoxus
NCBI27292	552	474	437	233	312	62	281	572	360	692	435	390	285	92	203	k__Fungi;p__Ascomycota;c__Saccharomycetes;o__Saccharomycetales;f__Saccharomycetaceae;g__Saccharomyces;s__Saccharomyces_pastorianus
NCBI2729423	0	0	0	0	0	2	0	0	0	0	0	0	0	0	0	k__Bacteria;p__Pseudomonadota;c__Gammaproteobacteria;o__Pseudomonadales;f__Pseudomonadaceae;g__Pseudomonas;s__Pseudomonas_sp._SK
NCBI2730360	0	0	0	0	0	0	1	0	0	0	0	0	0	0	0	k__Bacteria;p__Pseudomonadota;c__Gammaproteobacteria;o__Oceanospirillales;f__Halomonadaceae;g__Halomonas;s__Halomonas_sp._PGE1
NCBI2731680	0	0	0	0	0	40	0	0	0	0	0	0	0	0	0	k__Bacteria;p__Actinomycetota;c__Actinomycetes;o__Micrococcales;f__Promicromonosporaceae;g__Cellulosimicrobium;s__Cellulosimicrobium_sp._72-3
NCBI2731681	0	0	0	0	1	0	0	0	0	0	0	6	0	0	0	k__Bacteria;p__Pseudomonadota;c__Gammaproteobacteria;o__Pseudomonadales;f__Pseudomonadaceae;g__Pseudomonas;s__Pseudomonas_campi

NCBI2731755	1	0	0	0	0	0	0	0	0	0	0	0	0	0	0	k__Bacteria;p__Pseudomonadota;c__Gammaproteobacteria;o__Cellvibrionales;f__Cellvibrionaceae;g__Teredinibacter;s__Teredinibacter_haidensis
NCBI2732163	1982	2159	1294	131	4960	36743	2472	172	909	369	332	946	323	219	337	k__Bacteria;p__Pseudomonadota;c__Betaproteobacteria;o__Burkholderiales;f__Burkholderiaceae;g__Limnobacter;s__Limnobacter_sp._SAORIC-580
NCBI27322	3	1	0	0	0	0	0	0	0	0	0	0	0	0	0	k__Fungi;p__Ascomycota;c__Saccharomycetes;o__Saccharomycetales;f__Metschnikowiaceae;g__Metschnikowia;s__Metschnikowia_bicuspidata
NCBI2732511	0	1	0	0	0	6	0	0	0	0	0	0	0	0	0	k__Bacteria;p__Pseudomonadota;c__Betaproteobacteria;o__Burkholderiales;f__Comamonadaceae;g__Ramlibacter;s__Ramlibacter_sp._H242
NCBI2733662	0	0	0	0	0	0	0	0	0	0	1	0	0	0	0	k__Heunggongvirae;p__Uroviricota;c__Caudoviricetes;o__;f__Autographiviridae;g__Pijolavirus;s__Pijolavirus_PspYZU08
NCBI273371	0	0	0	0	1	0	0	0	0	0	0	8	0	0	0	k__Fungi;p__Ascomycota;c__Saccharomycetes;o__Saccharomycetales;f__Debaryomycetaceae;g__Candida;s__Candida_orthopsilosis
NCBI27338	63	62	58	50	21	16	35	36	31	26	37	42	0	7	29	k__Fungi;p__Ascomycota;c__Sordariomycetes;o__Sordariales;f__Sordariaceae;g__Sordaria;s__Sordaria_fimicola
NCBI273384	0	0	0	3	0	0	0	0	0	0	0	1	0	0	0	k__Bacteria;p__Actinomycetota;c__Actinomycetes;o__Micrococcales;f__Brevibacteriaceae;g__Brevibacterium;s__Brevibacterium_aurantiacum
NCBI2733866	0	0	0	0	1	0	0	0	0	0	0	0	0	0	0	k__Bacteria;p__Pseudomonadota;c__Gammaproteobacteria;o__Moraxellales;f__Moraxellaceae;g__Psychrobacter;s__Psychrobacter_sp._KCTC_72983
NCBI2733867	0	1	0	0	0	17	1	0	0	0	0	0	0	0	1	k__Bacteria;p__Pseudomonadota;c__Alphaproteobacteria;o__Sphingomonadales;f__Sphingomonadaceae;g__Sphingobium;s__Sphingobium_sp._KCTC_72723

NCBI2734109	1	1	0	1	0	3	1	0	0	0	0	0	0	0	0	k__Heunggongvirae;p__Uroviricota;c__Caudoviricetes;o__;f__;g__Goslarvirus;s__Goslarvirus_goslar
NCBI2735134	0	0	0	0	0	1	0	0	0	0	0	0	0	1	0	k__Bacteria;p__Pseudomonadota;c__Alphaproteobacteria;o__Sphingomonadales;f__Sphingomonadaceae;g__Sphingomonas;s__Sphingomonas_sp._AP4-R1
NCBI2735136	0	0	0	1	1	2	0	0	0	0	0	1	0	0	0	k__Bacteria;p__Actinomycetota;c__Actinomycetes;o__Mycobacteriales;f__Corynebacteriaceae;g__Corynebacterium;s__Corynebacterium_wankanglinii
NCBI2735528	0	0	0	0	0	0	1	0	0	0	0	0	0	0	0	k__Bacteria;p__Pseudomonadota;c__Alphaproteobacteria;o__Hyphomicrobiales;f__Rhizobiaceae;g__Agrobacterium;s__Agrobacterium_vaccinii
NCBI2735554	540	382	98	0	364	4731	312	25	0	0	0	0	0	0	0	k__Bacteria;p__Pseudomonadota;c__Betaproteobacteria;o__Burkholderiales;f__Comamonadaceae;g__Diaphorobacter;s__Diaphorobacter_sp._JS3050
NCBI2735906	2	0	0	0	0	2	0	0	1	0	0	0	0	0	0	k__Bacteria;p__Pseudomonadota;c__Gammaproteobacteria;o__Pseudomonadales;f__Pseudomonadaceae;g__Pseudomonas;s__Pseudomonas_sp._B11D7D
NCBI2736757	0	0	0	0	0	13	0	0	0	0	0	0	0	0	0	k__Bacteria;p__Actinomycetota;c__Actinomycetes;o__Propionibacteriales;f__Nocardioidaceae;g__Nocardioides;s__Nocardioides_campestrisoli
NCBI2738409	0	0	0	0	0	7	0	0	0	0	0	0	0	0	0	k__Bacteria;p__Actinomycetota;c__Actinomycetes;o__Mycobacteriales;f__Mycobacteriaceae;g__Mycobacterium;s__Mycobacterium_ostraviense
NCBI2738979	47	67	47	330	414	246	37	31	30	14	11	23	17	15	23	k__Bacteria;p__Bacillota;c__Bacilli;o__Bacillales;f__Paenibacillaceae;g__Brevibacillus;s__Brevibacillus_sp._HD3.3A
NCBI2739433	0	0	0	0	0	4	0	0	0	0	0	0	0	0	0	k__Bacteria;p__Pseudomonadota;c__Alphaproteobacteria;o__Sphingomonadales;f__Erythrobacteraceae;g__Erythrobacter;s__Erythrobacter_mangrovi

NCBI2739434	0	0	0	0	0	7	2	0	0	0	0	0	0	0	0	k__Bacteria;p__Pseudomonadota;c__Betaproteobacteria;o__Neisseriales;f__Chromobacteriaceae;g__Chitinibacter;s__Chitinibacter_bivalviorum
NCBI274	0	0	0	0	1	8	0	0	0	0	0	0	0	0	0	k__Bacteria;p__Deinococcota;c__Deinococci;o__Thermales;f__Thermaceae;g__Thermus;s__Thermus_thermophilus
NCBI2741499	0	0	0	0	0	0	0	0	0	0	0	2	0	0	0	k__Bacteria;p__Pseudomonadota;c__Gammaproteobacteria;o__Enterobacterales;f__Yersiniaceae;g__Serratia;s__Serratia_surfactantfaciens
NCBI2741720	189	306	87	11	410	2193	196	12	69	31	26	89	37	17	37	k__Bacteria;p__Pseudomonadota;c__Betaproteobacteria;o__Burkholderiales;f__Comamonadaceae;g__Rhodoferax;s__Rhodoferax_sp._BAB1
NCBI2742128	0	0	0	0	0	0	0	0	1	0	0	0	0	0	0	k__Bacteria;p__Actinomycetota;c__Actinomycetes;o__Streptosporangiales;f__Thermomonosporaceae;g__Actinomadura;s__Actinomadura_sp._NAK00032
NCBI2742137	0	0	0	0	0	0	0	0	0	0	0	1	0	0	0	k__Bacteria;p__Actinomycetota;c__Actinomycetes;o__Kitasatosporales;f__Streptomycetaceae;g__Streptomyces;s__Streptomyces_sp._NA02950
NCBI2742204	0	0	0	0	0	1	0	0	0	0	0	0	0	0	0	k__Bacteria;p__Pseudomonadota;c__Alphaproteobacteria;o__Hyphomicrobiales;f__Hyphomicrobiaceae;g__Methyloligella;s__Methyloligella_sp._GL2
NCBI2742619	0	0	2	0	0	0	0	0	0	0	0	0	0	0	0	k__Bacteria;p__Pseudomonadota;c__Gammaproteobacteria;o__Enterobacterales;f__Enterobacteriaceae;g__Enterobacter;s__Enterobacter_sp._RHB15-C17
NCBI2743	0	0	0	0	0	3	0	0	0	0	0	0	0	0	0	k__Bacteria;p__Pseudomonadota;c__Gammaproteobacteria;o__Pseudomonadales;f__Marinobacteraceae;g__Marinobacter;s__Marinobacter_nauticus
NCBI2743470	13	0	3	15	42	231	9	0	3	4	8	0	17	4	0	k__Bacteria;p__Pseudomonadota;c__Betaproteobacteria;o__Burkholderiales;f__Comamonadaceae;g__Acidovorax;s__Acidovorax_antarcticus

NCBI2743575	0	0	3	0	4	4	0	0	0	0	0	0	0	0	0	k__Bacteria;p__Pseudomonadota;c__Gammaproteobacteria;o__Moraxellales;f__Moraxellaceae;g__Acinetobacter;s__Acinetobacter_sp._NEB_394
NCBI2745197	0	0	0	0	0	2	0	0	0	0	0	0	0	0	0	k__Bacteria;p__Bacteroidota;c__Cytophagia;o__Cytophagales;f__Hymenobacteraceae;g__Adhaeribacter;s__Adhaeribacter_radiodurans
NCBI2745198	0	0	2	1	0	0	0	0	0	0	0	0	0	0	0	k__Bacteria;p__Bacillota;c__Bacilli;o__Bacillales;f__Bacillaceae;g__Metabacillus;s__Metabacillus_sp._KUDC1714
NCBI2745503	0	0	0	0	0	2	25	0	0	0	0	0	0	0	0	k__Bacteria;p__Pseudomonadota;c__Gammaproteobacteria;o__Pseudomonadales;f__Pseudomonadaceae;g__Pseudomonas;s__Pseudomonas_iranensis
NCBI2745514	0	0	0	3	2	3	2	0	0	0	0	0	0	0	0	k__Bacteria;p__Pseudomonadota;c__Gammaproteobacteria;o__Pseudomonadales;f__Pseudomonadaceae;g__Pseudomonas;s__Pseudomonas_salmasensis
NCBI2745518	0	0	0	0	0	1	0	0	0	0	0	0	0	0	0	k__Bacteria;p__Pseudomonadota;c__Gammaproteobacteria;o__Pseudomonadales;f__Pseudomonadaceae;g__Pseudomonas;s__Pseudomonas_tritici
NCBI2745519	5	11	0	19	89	176	18	1	6	0	0	4	0	2	1	k__Bacteria;p__Pseudomonadota;c__Gammaproteobacteria;o__Pseudomonadales;f__Pseudomonadaceae;g__Pseudomonas;s__Pseudomonas_sp._OE_28.3
NCBI2745590	0	0	0	3	0	7	0	0	0	0	0	0	0	0	0	k__Bacteria;p__Pseudomonadota;c__Alphaproteobacteria;o__Rhodobacterales;f__Paracoccaceae;g__;s__Rhodobacteraceae_bacterium_SC52
NCBI2747968	0	0	0	0	0	1	0	0	0	0	0	4	0	0	0	k__Fungi;p__Ascomycota;c__Sordariomycetes;o__Hypocreales;f__Nectriaceae;g__Fusarium;s__Fusarium_vanettenii
NCBI2748080	1	0	0	0	0	0	0	0	0	0	0	0	0	0	0	k__Bacteria;p__Pseudomonadota;c__Gammaproteobacteria;o__Pseudomonadales;f__Pseudomonadaceae;g__Pseudomonas;s__Pseudomonas_sp._ABC1

NCBI2749807	0	0	0	0	0	9	0	0	0	0	0	0	0	0	0	k__Bacteria;p__Pseudomonadota;c__Gammaproteobacteria;o__Pseudomonadales;f__Pseudomonadaceae;g__Pseudomonas;s__Pseudomonas_sp._Y39-6
NCBI2749991	0	0	0	0	14	0	0	0	0	0	0	0	0	0	0	k__Bacteria;p__Actinomycetota;c__Actinomycetes;o__Mycobacteriales;f__Nocardiaceae;g__Nocardia;s__Nocardia_gipuzkoensis
NCBI2749999	0	0	0	0	0	5	0	0	0	0	0	1	0	0	0	k__Bacteria;p__Pseudomonadota;c__Gammaproteobacteria;o__Pseudomonadales;f__Pseudomonadaceae;g__Pseudomonas;s__Pseudomonas_sp._RtIB026
NCBI2750025	0	0	0	0	11	0	0	0	0	0	0	0	0	0	0	k__Bacteria;p__Actinomycetota;c__Actinomycetes;o__Kitasatosporales;f__Streptomycetaceae;g__Streptomyces;s__Streptomyces_sp._CB00271
NCBI2750669	0	0	0	0	0	0	0	0	0	0	0	1	0	0	0	k__Archaea;p__Euryarchaeota;c__Halobacteria;o__Halobacteriales;f__Haloarculaceae;g__Natronomonas;s__Natronomonas_sp._LN261
NCBI2751170	0	0	0	0	0	0	0	0	0	0	0	2	0	0	0	k__Bacteria;p__Cyanobacteriota;c__Cyanophyceae;o__Synechococcales;f__Synechococcaceae;g__Synechococcus;s__Synechococcus_sp._LTW-R
NCBI2752316	0	0	0	0	1	2	0	0	0	0	0	0	0	0	0	k__Bacteria;p__Pseudomonadota;c__Betaproteobacteria;o__Burkholderiales;f__Comamonadaceae;g__Rhodoferax;s__Rhodoferax_sp._AJA081-3
NCBI2752515	0	0	0	0	0	2	0	0	0	0	0	0	0	0	0	k__Bacteria;p__Pseudomonadota;c__Alphaproteobacteria;o__Caulobacterales;f__Caulobacteraceae;g__Brevundimonas;s__Brevundimonas_sp._AJA228-03
NCBI2753607	2	0	0	1	2	43	2	0	0	1	0	1	0	0	0	k__Bacteria;p__Pseudomonadota;c__Betaproteobacteria;o__Burkholderiales;f__;g__Rhizobacter;s__Rhizobacter_sp._AJA081-3
NCBI2754044	0	0	0	0	0	0	0	0	0	0	1	0	0	0	0	k__Bacteria;p__Bacillota;c__Negativicutes;o__Selenomonadales;f__Selenomonadaceae;g__Selenomonas;s__Selenomonas_timonae

NCBI2754694	0	0	0	37	13	0	0	0	0	0	0	6	0	0	0	k__Bacteria;p__Bacteroidota;c__Flavobacteriia;o__Flavobacteriales;f__Weeksellaceae;g__Chryseobacterium;s__Chryseobacterium_manosquense
NCBI2754725	0	0	0	0	0	0	1	0	0	0	0	0	0	0	0	k__Bacteria;p__Actinomycetota;c__Actinomycetes;o__Mycobacteriales;f__Corynebacteriaceae;g__Corynebacterium;s__Corynebacterium_incognita
NCBI2755035	0	0	0	0	0	1	0	0	0	0	0	0	0	0	0	k__Bacteria;p__Deinococcota;c__Deinococci;o__Deinococcales;f__Deinococcaceae;g__Deinococcus;s__Deinococcus_sp._D7000
NCBI2755405	0	0	0	0	0	0	25	0	0	0	0	0	0	0	0	k__Bacteria;p__Bacteroidota;c__Bacteroidia;o__Bacteroidales;f__Bacteroidaceae;g__Bacteroides;s__Bacteroides_sp._CACC_737
NCBI2756	0	0	0	0	0	0	0	1	0	0	0	0	0	0	0	k__Bacteria;p__Bacillota;c__Bacilli;o__Bacillales;f__Listeriaceae;g__Brochothrix;s__Brochothrix_thermosphacta
NCBI2758041	0	0	0	0	0	1	0	0	0	0	0	0	0	0	0	k__Bacteria;p__Pseudomonadota;c__Alphaproteobacteria;o__Hyphomicrobiales;f__Aurantimonadaceae;g__Aureimonas;s__Aureimonas_mangrovi
NCBI2758440	0	0	0	2	0	6	0	0	0	0	0	0	0	0	0	k__Bacteria;p__Actinomycetota;c__Actinomycetes;o__Actinomycetales;f__Actinomycetaceae;g__Changpingibacter;s__Changpingibacter_yushuensis
NCBI2758710	0	0	0	0	0	1	0	0	0	0	0	0	0	0	0	k__Bacteria;p__Actinomycetota;c__Actinomycetes;o__Mycobacteriales;f__Gordoniaceae;g__Gordonia;s__Gordonia_jinghuaiqii
NCBI2759167	0	0	0	0	0	0	0	0	0	0	0	7	0	0	0	k__Bacteria;p__Actinomycetota;c__Actinomycetes;o__Micrococcales;f__Dermabacteraceae;g__Brachybacterium;s__Brachybacterium_sp._Z12
NCBI2759526	0	0	0	0	1	0	0	0	0	0	0	0	0	0	0	k__Bacteria;p__Pseudomonadota;c__Alphaproteobacteria;o__Sphingomonadales;f__Sphingomonadaceae;g__Sphingomonas;s__Sphingomonas_aliaeris

NCBI2760307	0	0	0	0	0	0	0	1	0	0	0	0	0	0	0	k__Bacteria;p__Pseudomonadota;c__Alphaproteobacteria;o__Rhodobacterales;f__Paracoccaceae;g__Paracoccus;s__Paracoccus_sp._MC1862
NCBI2761047	0	0	1	8	12	32	0	0	1	0	0	0	0	2	0	k__Bacteria;p__Actinomycetota;c__Actinomycetes;o__Micrococcales;f__Intrasporangiaceae;g__Janibacter;s__Janibacter_sp._YB324
NCBI2761102	0	2	0	0	0	0	0	0	0	0	0	0	0	0	0	k__Archaea;p__Euryarchaeota;c__Halobacteria;o__Haloferacales;f__Haloferacaceae;g__Halobellus;s__Halobellus_ruber
NCBI2762277	0	0	0	0	0	0	0	0	0	1	0	0	0	0	0	k__Bacteria;p__Pseudomonadota;c__Alphaproteobacteria;o__Rhodospirillales;f__Acetobacteraceae;g__Entomobacter;s__Entomobacter_blattae
NCBI2762285	3	3	0	10	1	25	5	0	3	0	0	0	0	0	0	k__Heunggongvirae;p__Uroviricota;c__Caudoviricetes;o__;f__;g__;s__Stenotrophomonas_phage_phiSHP3
NCBI2762331	0	0	0	0	0	0	0	0	0	0	0	1	0	0	0	k__Bacteria;p__Actinomycetota;c__Actinomycetes;o__Micrococcales;f__Dermacoccaceae;g__Dermacoccus;s__Dermacoccus_sp._PAMC28757
NCBI2762729	0	0	0	0	0	2	0	0	0	0	0	0	0	0	0	k__Bacteria;p__Pseudomonadota;c__Alphaproteobacteria;o__Sphingomonadales;f__Sphingomonadaceae;g__Novosphingopyxis;s__Novosphingopyxis_iocasae
NCBI2763008	0	0	0	0	0	34	0	0	0	0	0	0	0	0	0	k__Bacteria;p__Actinomycetota;c__Actinomycetes;o__Propionibacteriales;f__Nocardioidaceae;g__Nocardioides;s__Nocardioides_sp._zg-1228
NCBI2763010	0	0	0	0	0	5	0	1	0	0	14	3	0	0	0	k__Bacteria;p__Actinomycetota;c__Actinomycetes;o__Mycobacteriales;f__Corynebacteriaceae;g__Corynebacterium;s__Corynebacterium_lujinxingii
NCBI2763022	0	0	2	0	0	0	15	3	0	0	0	0	0	0	0	k__Bacteria;p__Bacteroidota;c__Bacteroidia;o__Bacteroidales;f__Bacteroidaceae;g__Bacteroides;s__Bacteroides_sp._M10

NCBI2763056	0	0	0	0	0	0	1	0	0	0	0	0	0	0	0	k__Bacteria;p__Bacillota;c__Clostridia;o__Eubacteriales;f__Oscillospiraceae;g__Oscillibacter;s__Oscillibacter_hominis
NCBI2763257	0	8	0	3	0	149	1	10	0	34	13	0	49	22	58	k__Bacteria;p__Actinomycetota;c__Actinomycetes;o__Micrococcales;f__Microbacteriaceae;g__Microbacterium;s__Microbacterium_sp._YJN-G
NCBI2763667	0	0	0	0	0	8	0	0	0	0	0	1	0	0	0	k__Bacteria;p__Bacillota;c__Clostridia;o__Eubacteriales;f__Lachnospiraceae;g__Wansuia;s__Wansuia_hejianensis
NCBI2763670	0	0	1	0	0	8	0	0	0	0	0	8	0	0	0	k__Bacteria;p__Bacillota;c__Clostridia;o__Eubacteriales;f__Lachnospiraceae;g__Wujia;s__Wujia_chipingensis
NCBI2763672	0	0	0	3	0	14	0	1	0	0	0	11	0	0	0	k__Bacteria;p__Bacillota;c__Clostridia;o__Eubacteriales;f__Lachnospiraceae;g__Simiaoa;s__Simiaoa_sunii
NCBI2764325	0	0	0	0	1	1	0	0	0	0	0	2	0	0	0	k__Bacteria;p__Bacillota;c__Clostridia;o__Eubacteriales;f__Eubacteriaceae;g__Eubacterium;s__Eubacterium_hominis
NCBI2768039	0	0	0	0	0	0	0	0	11	0	0	0	0	0	0	k__Bacteria;p__Bacteroidota;c__Bacteroidia;o__Bacteroidales;f__Muribaculaceae;g__Duncaniella;s__uncultured_Duncaniella_sp.
NCBI2768067	0	0	0	0	0	4	0	0	0	0	0	0	0	0	0	k__Bacteria;p__Actinomycetota;c__Actinomycetes;o__Kitasatosporales;f__Streptomycetaceae;g__Streptomyces;s__Streptomyces_sp._JCM17656
NCBI2768161	0	0	0	0	0	1	0	0	0	0	0	0	0	0	0	k__Bacteria;p__Pseudomonadota;c__Alphaproteobacteria;o__Rhodospirillales;f__Acetobacteraceae;g__Roseomonas;s__Roseomonas_marmotae
NCBI2768162	1	0	0	0	0	1	0	0	0	0	0	0	0	0	0	k__Bacteria;p__Pseudomonadota;c__Alphaproteobacteria;o__Rhodospirillales;f__Acetobacteraceae;g__Roseomonas;s__Roseomonas_haemaphysalidis
NCBI2768165	0	0	0	0	9	0	0	0	0	0	0	0	0	0	0	k__Bacteria;p__Pseudomonadota;c__Gammaproteobacteria;o__Enterobacterales;f__Erwiniaceae;g__Pantoea;s__P

																antoea_sp._MT58
NCBI2769358	131	139	169	196	179	38	210	202	223	200	170	260	57	95	97	k__Heunggongvirae;p__Uroviricota;c__Caudoviricetes;o__;f__Herelleviridae;g__Mooreparkvirus;s__Lactobacillus_phage_Lbab1
NCBI2769491	0	0	0	0	0	8	0	0	0	0	0	0	0	0	0	k__Bacteria;p__Pseudomonadota;c__Betaproteobacteria;o__Burkholderiales;f__Oxalobacteraceae;g__Massilia;s__Massilia_sp._LPB0304
NCBI2770551	0	1	0	0	0	0	0	0	0	0	0	0	0	0	0	k__Bacteria;p__Actinomycetota;c__Actinomycetes;o__Micrococcales;f__Dermacoccaceae;g__Yimella;s__Yimella_sp._cx-51
NCBI2771360	0	0	0	0	0	6	0	0	0	0	0	0	0	0	0	k__Bacteria;p__Pseudomonadota;c__Betaproteobacteria;o__Burkholderiales;f__Burkholderiaceae;g__Cupriavidus;s__Cupriavidus_sp._ISTL7
NCBI2771436	0	0	0	0	0	39	0	0	0	0	0	0	0	0	0	k__Bacteria;p__Pseudomonadota;c__Gammaproteobacteria;o__Xanthomonadales;f__Xanthomonadaceae;g__Thermomonas;s__Thermomonas_sp._XSG
NCBI2772095	0	0	0	0	0	3	0	0	0	0	0	0	0	0	0	k__Heunggongvirae;p__Uroviricota;c__Caudoviricetes;o__Crassvirales;f__Suoliviridae;g__Buhlduvirus;s__Buhlduvirus_animalis
NCBI2772254	0	0	2	0	0	0	0	0	0	0	0	0	0	0	0	k__Bacteria;p__Pseudomonadota;c__Gammaproteobacteria;o__Moraxellales;f__Moraxellaceae;g__Psychrobacter;s__Psychrobacter_sp._28M-43
NCBI2772401	0	0	0	0	2	1	0	0	0	0	0	2	0	0	0	k__Bacteria;p__Actinomycetota;c__Actinomycetes;o__Micrococcales;f__Micrococcaceae;g__Pseudarthrobacter;s__Pseudarthrobacter_sp._BIM_B-2242
NCBI2772483	0	0	0	1	0	0	0	0	0	0	0	0	0	0	0	k__Bacteria;p__Bacteroidota;c__Cytophagia;o__Cytophagales;f__Cyclobacteriaceae;g__Algoriphagus;s__Algoriphagus_sp._Y33

NCBI2772558	0	0	0	0	2	63	0	1	1	0	0	2	0	0	0	k__Bacteria;p__Pseudomonadota;c__Gammaproteobacteria;o__Pseudomonadales;f__Pseudomonadaceae;g__Pseudomonas;s__Pseudomonas_paralcaligenes
NCBI2774151	0	0	0	0	2	4	0	0	0	0	0	0	0	0	0	k__Bacteria;p__Planctomycetota;c__Planctomycetia;o__Gemmatales;f__Gemmataceae;g__Frigoriglobus;s__Frigoriglobus_tundricola
NCBI2774189	3	6	1	21	35	122	11	0	1	0	0	3	0	0	1	k__Bacteria;p__Pseudomonadota;c__Alphaproteobacteria;o__Caulobacterales;f__Caulobacteraceae;g__Brevundimonas;s__Brevundimonas_pondensis
NCBI2774190	0	0	0	0	0	5	0	0	0	0	0	0	0	0	0	k__Bacteria;p__Pseudomonadota;c__Alphaproteobacteria;o__Caulobacterales;f__Caulobacteraceae;g__Brevundimonas;s__Brevundimonas_goettingensis
NCBI2774459	0	0	0	0	0	3	0	0	0	0	0	0	0	0	0	k__Bacteria;p__Pseudomonadota;c__Gammaproteobacteria;o__Pseudomonadales;f__Pseudomonadaceae;g__Pseudomonas;s__Pseudomonas_sp._IzPS59
NCBI2774562	0	2	0	0	6	83	5	0	4	0	0	0	0	0	0	k__Bacteria;p__Pseudomonadota;c__Alphaproteobacteria;o__Hyphomicrobiales;f__Phyllobacteriaceae;g__Aminobacter;s__Aminobacter_sp._SR38
NCBI2774873	0	0	0	0	0	0	0	0	0	0	0	4	0	0	0	k__Bacteria;p__Pseudomonadota;c__Gammaproteobacteria;o__Pseudomonadales;f__Pseudomonadaceae;g__Pseudomonas;s__Pseudomonas_sp._ADPe
NCBI2775420	0	0	0	0	0	4	0	0	0	0	0	0	0	0	0	k__Bacteria;p__Pseudomonadota;c__Alphaproteobacteria;o__Rhodospirillales;f__Azospirillaceae;g__Skermanella;s__Skermanella_sp._TT6
NCBI2775920	0	0	0	0	0	445	65	0	0	0	0	0	0	0	0	k__Bacteria;p__Pseudomonadota;c__Gammaproteobacteria;o__Xanthomonadales;f__Xanthomonadaceae;g__Stenotrophomonas;s__Stenotrophomonas_sp._CW117
NCBI2777475	0	0	0	3	0	0	0	0	0	0	0	0	0	0	0	k__Bacteria;p__Pseudomonadota;c__Alphaproteobacteria;o__Hyphomicrobiales;f__Phyllobacteriaceae;g__Mesorhizobium;s__Mesorhizobium_sp._J8

NCBI2777556	0	0	0	0	0	2	0	0	0	0	0	0	0	0	0	k__Bacteria;p__Actinomycetota;c__Actinomycetes;o__Micrococcales;f__Brevibacteriaceae;g__Brevibacterium;s__Brevibacterium_sp._SMBL_HHYL_HB1
NCBI2777984	0	0	0	0	0	0	0	0	0	0	0	1	0	0	0	k__Bacteria;p__Bacillota;c__Bacilli;o__Bacillales;f__Paenibacillaceae;g__Paenibacillus;s__Paenibacillus_sp._JNUCC-32
NCBI2778055	0	0	0	0	0	0	0	0	0	0	0	90	0	0	0	k__Bacteria;p__Pseudomonadota;c__Gammaproteobacteria;o__Aeromonadales;f__Aeromonadaceae;g__Aeromonas;s__Aeromonas_sp._FDAARGOS_1406
NCBI2778064	0	0	0	5	0	0	0	0	0	0	0	3	0	3	0	k__Bacteria;p__Pseudomonadota;c__Gammaproteobacteria;o__Aeromonadales;f__Aeromonadaceae;g__Aeromonas;s__Aeromonas_sp._FDAARGOS_1415
NCBI2778068	0	0	0	0	0	0	0	0	0	0	0	15	0	0	0	k__Bacteria;p__Pseudomonadota;c__Gammaproteobacteria;o__Aeromonadales;f__Aeromonadaceae;g__Aeromonas;s__Aeromonas_sp._FDAARGOS_1419
NCBI2779362	0	0	0	0	0	0	0	0	0	0	0	1	0	0	0	k__Bacteria;p__Bacteroidota;c__Flavobacteriia;o__Flavobacteriales;f__Flavobacteriaceae;g__Mesoflavibacter;s__Mesoflavibacter_sp._SCSIO_43206
NCBI2780074	70	133	69	5	286	3070	94	4	46	17	13	41	17	18	15	k__Bacteria;p__Pseudomonadota;c__Alphaproteobacteria;o__Sphingomonadales;f__Sphingomonadaceae;g__Novosphingobium;s__Novosphingobium_sp._ES2-1
NCBI2781367	0	0	0	0	3	2	0	0	0	0	0	0	0	0	0	k__Heunggongvirae;p__Uroviricota;c__Caudoviricetes;o__;f__;g__;s__Bacteriophage_sp._438212
NCBI2781735	0	0	0	0	0	147	0	0	0	0	0	0	0	0	0	k__Bacteria;p__Actinomycetota;c__Actinomycetes;o__Pseudonocardiales;f__Pseudonocardiaceae;g__Saccharothrix;s__Saccharothrix_sp._6-C
NCBI2781976	0	0	0	0	0	0	78	0	0	0	0	0	0	0	0	k__Bacteria;p__Pseudomonadota;c__Gammaproteobacteria;o__Moraxellales;f__Moraxellaceae;g__Acinetobacter;s__Acinetobacter_sp._Ac-14

NCBI2782166	0	0	0	1	0	0	0	0	0	0	0	0	0	0	0	k__Bacteria;p__Actinomycetota;c__Actinomycetes;o__Micrococcales;f__Microbacteriaceae;g__Microbacterium;s__Microbacterium_sp._A18JL241
NCBI2782167	0	0	0	0	0	66	0	0	0	40	2	0	7	6	22	k__Bacteria;p__Actinomycetota;c__Actinomycetes;o__Micrococcales;f__Microbacteriaceae;g__Microbacterium;s__Microbacterium_luteum
NCBI2782168	0	0	0	0	0	24	0	0	0	0	0	0	0	0	0	k__Bacteria;p__Actinomycetota;c__Actinomycetes;o__Micrococcales;f__Microbacteriaceae;g__Microbacterium;s__Microbacterium_atlanticum
NCBI2783796	0	0	0	0	0	5	0	0	0	0	0	0	0	0	0	k__Bacteria;p__Bacillota;c__Bacilli;o__Bacillales;f__;g__Exiguobacterium;s__Exiguobacterium_sp._PBE
NCBI2785056	0	0	0	0	0	17	0	0	0	0	0	0	0	0	0	k__Bacteria;p__Pseudomonadota;c__Alphaproteobacteria;o__Hyphomicrobiales;f__Rhizobiaceae;g__Rhizobium;s__Rhizobium_sp._007
NCBI2785531	0	0	0	0	0	0	15	0	0	0	0	0	0	0	0	k__Bacteria;p__Bacteroidota;c__Bacteroidia;o__Bacteroidales;f__Bacteroidaceae;g__Bacteroides;s__Bacteroides_sp._HF-162
NCBI2789216	0	0	0	0	0	1	0	0	0	0	0	0	0	0	0	k__Bacteria;p__Pseudomonadota;c__Alphaproteobacteria;o__Hyphomicrobiales;f__Xanthobacteraceae;g__Labrys;s__Labrys_sp._KNU-23
NCBI2789327	3	1	0	0	4	51	1	0	0	0	0	2	0	0	0	k__Bacteria;p__Pseudomonadota;c__Alphaproteobacteria;o__Sphingomonadales;f__Sphingomonadaceae;g__Sphingobium;s__Sphingobium_sp._Cam5-1
NCBI2789424	0	0	0	0	4	0	0	0	0	0	0	0	0	0	0	k__Bacteria;p__Actinomycetota;c__Actinomycetes;o__Actinomycetales;f__Actinomycetaceae;g__Actinomyces;s__Actinomyces_sp._HMT897
NCBI2789425	0	0	0	6	10	9	1	27	0	0	0	0	0	0	0	k__Bacteria;p__Actinomycetota;c__Actinomycetes;o__Actinomycetales;f__Actinomycetaceae;g__Actinomyces;s__Actinomyces_sp._HMT_175

NCBI2789856	0	0	0	0	0	1	0	0	0	0	0	0	0	0	0	k__Bacteria;p__Pseudomonadota;c__Alphaproteobacteria;o__Rhodobacterales;f__Paracoccaceae;g__Pontivivens;s__Pontivivens_ytuae
NCBI2789857	0	0	0	0	0	0	0	0	0	0	0	1	0	0	0	k__Bacteria;p__Cyanobacteriota;c__Cyanophyceae;o__Synechococcales;f__Synechococcaceae;g__Synechococcus;s__Synechococcus_sp._CBW1107
NCBI2792224	160	314	111	0	487	6258	179	0	180	0	0	77	0	81	0	k__Bacteria;p__Pseudomonadota;c__Betaproteobacteria;o__Burkholderiales;f__Comamonadaceae;g__Diaphorobacter;s__Diaphorobacter_sp._JS3051
NCBI2795031	0	0	0	1	11	1	0	0	0	0	0	8	0	0	0	k__Bacteria;p__Actinomycetota;c__Actinomycetes;o__Mycobacteriales;f__Nocardiaceae;g__Rhodococcus;s__Rhodococcus_sp._P-2
NCBI2795216	21	13	26	79	66	104	44	49	49	275	244	390	226	251	271	k__Bacteria;p__Pseudomonadota;c__Alphaproteobacteria;o__Hyphomicrobiales;f__Rhizobiaceae;g__Rhizobium;s__Rhizobium_sp._AB2/73
NCBI2795690	0	0	0	0	0	2	0	0	0	0	0	0	0	0	0	k__Bacteria;p__Pseudomonadota;c__Alphaproteobacteria;o__Hyphomicrobiales;f__Kaistiaceae;g__Kaistia;s__Kaistia_sp._32K
NCBI2796366	0	0	0	0	0	2	0	1	0	0	0	0	0	0	0	k__Bacteria;p__Pseudomonadota;c__Betaproteobacteria;o__Burkholderiales;f__Comamonadaceae;g__Comamonas;s__Comamonas_fluminis
NCBI2798724	9	15	16	1	42	393	12	1	5	0	3	8	1	0	3	k__Bacteria;p__Pseudomonadota;c__Alphaproteobacteria;o__Sphingomonadales;f__Erythrobacteraceae;g__Croceicoccus;s__Croceicoccus_sp._YJ47
NCBI2799326	0	0	0	0	0	0	0	0	0	0	0	1	0	0	0	k__Bacteria;p__Pseudomonadota;c__Gammaproteobacteria;o__Xanthomonadales;f__Xanthomonadaceae;g__Luteimonas;s__Luteimonas_sp._MC1750
NCBI2799560	0	0	0	0	0	1	0	0	0	0	0	0	0	0	0	k__Bacteria;p__Bacillota;c__Clostridia;o__Eubacteriales;f__Oscillospiraceae;g__;s__Ruminococcaceae_bacterium_BL-4

NCBI280	14	8	5	0	30	434	13	0	1	2	1	3	1	1	0	k__Bacteria;p__Pseudomonadota;c__Alphaproteobacteria;o__Hyphomicrobiales;f__Xanthobacteraceae;g__Xanthobacter;s__Xanthobacter_autotrophicus
NCBI2800128	34	46	36	33	38	8	57	50	37	122	63	81	66	81	64	k__Bacteria;p__Bacillota;c__Bacilli;o__Lactobacillales;f__Carnobacteriaceae;g__Carnobacterium;s__Carnobacterium_sp._CS13
NCBI2800818	0	0	0	0	1	32	0	0	0	0	0	0	0	0	0	k__Bacteria;p__Pseudomonadota;c__Alphaproteobacteria;o__Caulobacterales;f__Caulobacteraceae;g__Brevundimonas;s__Brevundimonas_vitisensis
NCBI2801342	11	0	56	92	29	29	19	0	0	0	0	11	0	0	0	k__Bacteria;p__Bacillota;c__Bacilli;o__Bacillales;f__Bacillaceae;g__Bacillus;s__Bacillus_sp._TK-2
NCBI28025	0	0	0	0	0	16	1	2	0	0	0	6	0	0	0	k__Bacteria;p__Actinomycetota;c__Actinomycetes;o__Bifidobacteriales;f__Bifidobacteriaceae;g__Bifidobacterium;s__Bifidobacterium_animalis
NCBI28026	0	0	3	15	3	333	2	0	1	0	2	222	0	0	2	k__Bacteria;p__Actinomycetota;c__Actinomycetes;o__Bifidobacteriales;f__Bifidobacteriaceae;g__Bifidobacterium;s__Bifidobacterium_pseudocatenulatum
NCBI2802971	0	0	0	0	1	0	0	0	0	0	0	0	0	0	0	k__Bacteria;p__Acidobacteriota;c__Vicinamibacteria;o__Vicinamibacterales;f__Vicinamibacteraceae;g__Luteitalea;s__Luteitalea_sp._TBR-22
NCBI28031	0	2	3	8	6	13	0	0	0	0	1	0	0	3	1	k__Bacteria;p__Bacillota;c__Bacilli;o__Bacillales;f__Bacillaceae;g__Lysinibacillus;s__Lysinibacillus_fusiformis
NCBI28035	1	0	0	0	0	0	0	0	0	0	0	0	0	0	0	k__Bacteria;p__Bacillota;c__Bacilli;o__Bacillales;f__Staphylococcaceae;g__Staphylococcus;s__Staphylococcus_lugdunensis
NCBI28037	1	3	7	9	10	1	0	0	0	0	1	6	0	0	0	k__Bacteria;p__Bacillota;c__Bacilli;o__Lactobacillales;f__Streptococcaceae;g__Streptococcus;s__Streptococcus_mitis
NCBI2803784	0	0	0	0	2	1	1	0	0	0	0	0	0	0	0	k__Bacteria;p__Pseudomonadota;c__Alphaproteobacteria;o__Caulobacterales;f__Caulobacteraceae;g__Phenylob

															acterium;s__Phenylobacterium_glaciei
NCBI28038	31	35	44	63	57	80	52	53	58	45	52	68	16	12	25	k__Bacteria;p__Bacillota;c__Bacilli;o__Lactobacillales;f__Lactobacillaceae;g__Latilactobacillus;s__Latilactobacillus_curvatus
NCBI28042	0	0	0	0	0	6	0	0	0	0	0	0	0	0	0	k__Bacteria;p__Actinomycetota;c__Actinomycetes;o__Pseudonocardiales;f__Pseudonocardiaceae;g__Saccharopolyspora;s__Saccharopolyspora_rectivirgula
NCBI2804761	4	4	0	28	34	56	6	1	1	0	0	0	0	0	0	k__Bacteria;p__Pseudomonadota;c__Gammaproteobacteria;o__Pseudomonadales;f__Pseudomonadaceae;g__Pseudomonas;s__Pseudomonas_sp._15A4
NCBI2806251	0	1	0	1	0	0	0	0	1	0	0	0	0	0	0	k__Bacteria;p__Actinomycetota;c__Actinomycetes;o__Streptosporangiales;f__Nocardiopsaceae;g__Nocardiopsis;s__Nocardiopsis_sp._BM-2018
NCBI2806262	6	2	8	0	19	184	1	0	0	0	3	3	0	0	0	k__Bacteria;p__Pseudomonadota;c__Betaproteobacteria;o__Burkholderiales;f__Comamonadaceae;g__Hydrogenophaga;s__Hydrogenophaga_sp._YM1
NCBI28068	2	1	2	0	8	119	1	0	0	0	0	0	0	0	0	k__Bacteria;p__Pseudomonadota;c__Betaproteobacteria;o__Burkholderiales;f__Sphaerotilaceae;g__Rubrivivax;s__Rubrivivax_gelatinosus
NCBI2807096	0	2	0	2	0	6	0	0	0	0	0	0	0	0	0	k__Bacteria;p__Pseudomonadota;c__Alphaproteobacteria;o__Rhodobacterales;f__Paracoccaceae;g__Neotabrizicola;s__Neotabrizicola_shimadae
NCBI2807101	0	0	0	0	0	46	0	0	0	0	0	0	0	0	0	k__Bacteria;p__Pseudomonadota;c__Alphaproteobacteria;o__Hyphomicrobiales;f__Methylobacteriaceae;g__Microvirga;s__Microvirga_sp._VF16
NCBI28090	20	9	40	12	9	124	8	0	0	3	3	26	0	0	8	k__Bacteria;p__Pseudomonadota;c__Gammaproteobacteria;o__Moraxellales;f__Moraxellaceae;g__Acinetobacter;s__Acinetobacter_lwoffii
NCBI2	1	0	0	1	0	17	0	0	0	0	0	2	0	0	0	k__Bacteria;p__Pseudomonadota;c__Betaproteobacteria;

8095																o__Burkholderiales;f__Burkholderiaceae;g__Burkholderia;s__Burkholderia_gladioli
NCBI28097	0	8	0	0	0	0	0	0	0	0	0	0	0	0	0	k__Bacteria;p__Pseudomonadota;c__Betaproteobacteria;o__Burkholderiales;f__Burkholderiaceae;g__Ralstonia;s__Ralstonia_syzygii
NCBI28099	0	0	0	0	2	1	0	0	0	0	0	0	0	0	0	k__Bacteria;p__Pseudomonadota;c__Alphaproteobacteria;o__Hyphomicrobiales;f__Rhizobiaceae;g__Agrobacterium;s__Agrobacterium_rubi
NCBI2810070	0	0	0	0	3	0	0	0	0	0	0	0	0	0	0	k__Bacteria;p__Pseudomonadota;c__Gammaproteobacteria;o__Moraxellales;f__Moraxellaceae;g__Acinetobacter;s__Acinetobacter_sp._Colony158
NCBI2810513	0	0	0	0	0	0	0	0	0	0	0	1	0	0	0	k__Bacteria;p__Actinomycetota;c__Actinomycetes;o__Micrococcales;f__Microbacteriaceae;g__Agrococcus;s__Agrococcus_sp._Marseille-Q4369
NCBI28111	0	0	1	0	1	9	5	0	0	0	0	3	0	0	0	k__Bacteria;p__Bacteroidota;c__Bacteroidia;o__Bacteroidales;f__Bacteroidaceae;g__Bacteroides;s__Bacteroides_eggerthii
NCBI28112	0	0	0	0	0	0	0	0	0	0	0	1	0	0	0	k__Bacteria;p__Bacteroidota;c__Bacteroidia;o__Bacteroidales;f__Tannerellaceae;g__Tannerella;s__Tannerella_forsythia
NCBI28113	0	0	0	0	0	0	0	0	0	0	0	4	0	0	0	k__Bacteria;p__Bacteroidota;c__Bacteroidia;o__Bacteroidales;f__Bacteroidaceae;g__Bacteroides;s__Bacteroides_heparinolyticus
NCBI2811423	1	2	0	0	1	39	1	0	0	0	0	0	0	0	0	k__Bacteria;p__Pseudomonadota;c__Alphaproteobacteria;o__Hyphomicrobiales;f__Rhizobiaceae;g__Ensifer;s__Ensifer_sp._PDNC004
NCBI2811425	0	0	0	3	0	30	0	0	0	0	0	0	0	0	0	k__Bacteria;p__Pseudomonadota;c__Betaproteobacteria;o__Burkholderiales;f__Comamonadaceae;g__Variovorax;s__Variovorax_sp._PDNC026
NCBI2	4	0	15	0	6	22	157	0	3	0	0	43	0	0	0	k__Bacteria;p__Bacteroidota;c__Bacteroidia;o__Bacteroi

8116																dales;f__Bacteroidaceae;g__Bacteroides;s__Bacteroides_ovatus
NCBI28118	0	0	0	1	0	3	2	0	0	0	0	5	0	0	0	k__Bacteria;p__Bacteroidota;c__Bacteroidia;o__Bacteroidales;f__Odoribacteraceae;g__Odoribacter;s__Odoribacter_splanchnicus
NCBI2812000	0	1	0	1	5	2	1	1	0	0	0	0	0	0	0	k__Bacteria;p__Pseudomonadota;c__Gammaproteobacteria;o__Pseudomonadales;f__Pseudomonadaceae;g__Pseudomonas;s__Pseudomonas_sp._SDM007
NCBI28123	0	0	2	2	0	12	0	0	0	0	0	0	0	0	0	k__Bacteria;p__Bacteroidota;c__Bacteroidia;o__Bacteroidales;f__Porphyromonadaceae;g__Porphyromonas;s__Porphyromonas_asaccharolytica
NCBI28124	1	0	2	1	0	0	0	0	0	0	0	0	0	0	0	k__Bacteria;p__Bacteroidota;c__Bacteroidia;o__Bacteroidales;f__Porphyromonadaceae;g__Porphyromonas;s__Porphyromonas_endodontalis
NCBI2812560	0	0	0	0	0	41	0	0	0	0	0	0	0	0	0	k__Bacteria;p__Actinomycetota;c__Thermoleophilia;o__Solirubrobacterales;f__Conexibacteraceae;g__Conexibacter;s__Conexibacter_sp._SYSU_D00693
NCBI2812658	0	3	0	0	0	0	0	0	0	0	0	0	0	0	0	k__Bacteria;p__Pseudomonadota;c__Alphaproteobacteria;o__Rhodobacterales;f__Paracoccaceae;g__Paracoccus;s__Paracoccus_methylovorus
NCBI28127	0	0	0	3	0	5	0	1	0	0	0	2	0	0	0	k__Bacteria;p__Bacteroidota;c__Bacteroidia;o__Bacteroidales;f__Prevotellaceae;g__Hoylesella;s__Hoylesella_buccalis
NCBI281274	47	103	61	196	134	577	162	94	135	57	107	109	43	24	18	k__Bacteria;p__Bacillota;c__Clostridia;o__Eubacteriales;f__Desulfotomaculaceae;g__Pelotomaculum;s__uncultured_Pelotomaculum_sp.
NCBI28129	1	0	0	0	0	3	0	0	0	0	0	0	0	0	0	k__Bacteria;p__Bacteroidota;c__Bacteroidia;o__Bacteroidales;f__Prevotellaceae;g__Prevotella;s__Prevotella_denticola
NCBI2	0	0	0	0	0	0	8	4	2	0	0	5	0	0	0	k__Bacteria;p__Bacteroidota;c__Bacteroidia;o__Bacteroi

8131																dales;f__Prevotellaceae;g__Prevotella;s__Prevotella_intermedia
NCBI28132	2	0	2	9	7	12	0	42	0	0	0	20	1	0	0	k__Bacteria;p__Bacteroidota;c__Bacteroidia;o__Bacteroidales;f__Prevotellaceae;g__Prevotella;s__Prevotella_melaninogenica
NCBI28133	0	0	0	0	0	0	0	0	0	0	0	1	0	0	0	k__Bacteria;p__Bacteroidota;c__Bacteroidia;o__Bacteroidales;f__Prevotellaceae;g__Prevotella;s__Prevotella_nigrescens
NCBI2813371	0	0	0	0	0	3	0	0	0	0	0	0	0	0	0	k__Bacteria;p__Bacillota;c__Clostridia;o__Eubacteriales;f__Peptostreptococcaceae;g__Terrisporobacter;s__Terrisporobacter_hibernicus
NCBI28135	0	0	0	0	1	0	0	4	0	0	0	1	0	0	0	k__Bacteria;p__Bacteroidota;c__Bacteroidia;o__Bacteroidales;f__Prevotellaceae;g__Prevotella;s__Prevotella_oris
NCBI281362	0	0	2	0	0	4	0	0	0	0	0	0	0	0	0	k__Bacteria;p__Pseudomonadota;c__Betaproteobacteria;o__Rhodocyclales;f__Azonexaceae;g__Dechloromonas;s__Dechloromonas_denitrificans
NCBI28137	0	0	2	1	0	0	0	0	0	0	0	1	0	0	0	k__Bacteria;p__Bacteroidota;c__Bacteroidia;o__Bacteroidales;f__Prevotellaceae;g__Prevotella;s__Prevotella_veroralis
NCBI2813778	63	40	29	0	76	1401	36	3	10	6	2	8	11	1	2	k__Bacteria;p__Pseudomonadota;c__Alphaproteobacteria;o__Caulobacterales;f__Caulobacteraceae;g__Brevundimonas;s__Brevundimonas_sp._CS1
NCBI2813780	0	0	0	1	0	0	0	0	0	0	0	0	0	0	0	k__Bacteria;p__Pseudomonadota;c__Betaproteobacteria;o__Burkholderiales;f__Alcaligenaceae;g__Alcaligenes;s__Alcaligenes_sp._SORT26
NCBI28141	0	0	0	0	0	21	0	0	0	0	0	17	0	0	0	k__Bacteria;p__Pseudomonadota;c__Gammaproteobacteria;o__Enterobacterales;f__Enterobacteriaceae;g__Cronobacter;s__Cronobacter_sakazakii
NCBI2814292	0	0	0	0	0	1	0	0	0	0	0	0	0	0	0	k__Bacteria;p__Pseudomonadota;c__Gammaproteobacteria;o__Alteromonadales;f__Shewanellaceae;g__Shewane

																lla;s__Shewanella_cyperi
NCBI28151	0	0	0	0	0	1	0	0	0	0	0	0	0	0	0	k__Bacteria;p__Pseudomonadota;c__Gammaproteobacteria;o__Enterobacterales;f__Yersiniaceae;g__Serratia;s__Serratia_proteamaculans
NCBI2815358	0	0	0	0	0	104	0	0	0	0	0	5	0	1	0	k__Bacteria;p__Pseudomonadota;c__Gammaproteobacteria;o__Enterobacterales;f__Enterobacteriaceae;g__Leclercia;s__Leclercia_pneumoniae
NCBI2815360	65	78	18	5	141	1930	57	4	36	19	5	72	9	9	11	k__Bacteria;p__Pseudomonadota;c__Alphaproteobacteria;o__Hyphomicrobiales;f__Rhizobiaceae;g__Rhizobium;s__Rhizobium_sp._X9
NCBI2815720	0	0	0	0	0	0	5	0	0	0	0	0	0	0	0	k__Bacteria;p__Pseudomonadota;c__Gammaproteobacteria;o__Pseudomonadales;f__Pseudomonadaceae;g__Pseudomonas;s__Pseudomonas_germanica
NCBI2815936	0	0	0	1	0	1	0	0	0	0	0	3	4	0	0	k__Bacteria;p__Pseudomonadota;c__Gammaproteobacteria;o__Pseudomonadales;f__Pseudomonadaceae;g__Pseudomonas;s__Pseudomonas_sp._PP3
NCBI2816454	1	5	3	0	9	133	1	1	5	0	0	0	0	1	0	k__Bacteria;p__Pseudomonadota;c__Alphaproteobacteria;o__Hyphomicrobiales;f__Aurantimonadaceae;g__Aureimonas;s__Aureimonas_sp._OT7
NCBI2816911	22	0	11	0	107	0	0	0	0	0	0	0	0	0	0	k__Bacteria;p__Bacillota;c__Bacilli;o__Lactobacillales;f__Streptococcaceae;g__Lactococcus;s__Lactococcus_sp._LG592
NCBI2816950	0	0	0	0	0	10	0	0	0	1	0	0	0	0	0	k__Bacteria;p__Pseudomonadota;c__Betaproteobacteria;o__Burkholderiales;f__Comamonadaceae;g__Ottowia;s__Ottowia_testudinis
NCBI28198	0	0	0	0	1	0	0	0	0	0	0	0	0	0	0	k__Bacteria;p__Campylobacterota;c__Epsilonproteobacteria;o__Campylobacterales;f__Arcobacteraceae;g__Aliarcobacter;s__Aliarcobacter_cryaerophilus

NCBI2819999	0	0	0	0	0	24	0	0	0	0	0	0	0	0	0	k__Bacteria;p__Pseudomonadota;c__Alphaproteobacteria;o__Hyphomicrobiales;f__Rhizobiaceae;g__Rhizobium;s__Rhizobium_sp._L51/94
NCBI2820270	0	0	0	0	1	0	0	0	0	0	0	1	0	0	0	k__Bacteria;p__Bacteroidota;c__Flavobacteriia;o__Flavobacteriales;f__Weeksellaceae;g__Kaistella;s__Candidatus_Kaistella_beijingensis
NCBI2820673	0	0	0	3	0	0	0	0	0	0	0	0	0	0	0	k__Bacteria;p__Actinomycetota;c__Actinomycetes;o__Micrococcales;f__Microbacteriaceae;g__Pseudoclavibacter;s__Pseudoclavibacter_triregionum
NCBI28212	0	0	0	0	0	38	11	0	0	0	0	0	0	0	0	k__Bacteria;p__Pseudomonadota;c__Alphaproteobacteria;o__Sphingomonadales;f__Sphingomonadaceae;g__Sphingomonas;s__Sphingomonas_adhaesiva
NCBI28214	0	0	0	0	19	145	0	0	0	0	0	0	0	0	0	k__Bacteria;p__Pseudomonadota;c__Alphaproteobacteria;o__Sphingomonadales;f__Sphingomonadaceae;g__Sphingomonas;s__Sphingomonas_sp.
NCBI2822368	0	0	0	1	0	0	0	0	0	0	0	0	0	0	0	k__Bacteria;p__Pseudomonadota;c__Gammaproteobacteria;o__Xanthomonadales;f__Xanthomonadaceae;g__Lysobacter;s__Lysobacter_luteus
NCBI2822760	0	0	0	0	0	2	0	0	0	0	0	0	0	0	0	k__Bacteria;p__Pseudomonadota;c__Betaproteobacteria;o__Burkholderiales;f__Comamonadaceae;g__Rhodoferax;s__Rhodoferax_sp._PAMC_29310
NCBI2823316	0	0	0	0	0	3	0	0	0	0	0	3	0	0	1	k__Bacteria;p__Bacillota;c__Clostridia;o__Eubacteriales;f__Lachnospiraceae;g__Faecalicatena;s__Faecalicatena_sp._Marseille-Q4148
NCBI2823317	0	0	0	0	0	4	0	0	0	0	0	2	0	0	0	k__Bacteria;p__Bacillota;c__Negativicutes;o__Acidaminococcales;f__Acidaminococcaceae;g__Phascolarctobacterium;s__Phascolarctobacterium_sp._Marseille-Q4147
NCBI2823564	3	0	0	0	0	0	0	0	0	0	0	0	0	0	0	k__Heunggongvirae;p__Uroviricota;c__Caudoviricetes;o__;f__;g__;s__Siphoviridae_sp._ct5tj9

NCBI2823693	0	0	0	0	2	6	0	0	0	0	0	1	0	0	0	k__Bacteria;p__Pseudomonadota;c__Alphaproteobacteria;o__Caulobacterales;f__Caulobacteraceae;g__Caulobacter;s__Caulobacter_sp._S6
NCBI2823807	0	0	0	0	0	1	0	0	0	0	0	0	0	0	0	k__Bacteria;p__Pseudomonadota;c__Alphaproteobacteria;o__Hyphomicrobiales;f__Nitrobacteraceae;g__Bradyrhizobium;s__Bradyrhizobium_barranii
NCBI2823875	0	0	0	0	0	2	0	0	0	0	0	0	0	0	0	k__Bacteria;p__Pseudomonadota;c__Gammaproteobacteria;o__Pseudomonadales;f__Pseudomonadaceae;g__Pseudomonas;s__Pseudomonas_sp._Tri1
NCBI282402	0	0	0	3	0	0	0	0	0	0	0	1	0	0	0	k__Bacteria;p__Bacteroidota;c__Bacteroidia;o__Bacteroidales;f__Prevotellaceae;g__Prevotella;s__Prevotella_multiformis
NCBI2824561	0	0	0	0	1	0	0	0	0	0	0	0	0	0	0	k__Bacteria;p__Verrucomicrobiota;c__Verrucomicrobiae;o__Verrucomicrobiales;f__Verrucomicrobiaceae;g__Luteolibacter;s__Luteolibacter_ambystomatis
NCBI2825044	0	0	0	0	0	0	0	0	0	0	0	1	0	0	0	k__Heunggongvirae;p__Uroviricota;c__Caudoviricetes;o__;f__;g__;s__Myoviridae_sp._ctAys2
NCBI2825413	0	0	0	0	0	0	0	0	0	0	0	1	0	0	0	k__Heunggongvirae;p__Uroviricota;c__Caudoviricetes;o__;f__;g__;s__Siphoviridae_sp._ctGa111
NCBI2825586	0	0	0	0	0	1	0	0	0	0	0	0	0	0	0	k__Heunggongvirae;p__Uroviricota;c__Caudoviricetes;o__;f__;g__;s__Siphoviridae_sp._ctdmY20
NCBI2825595	0	0	0	0	0	1	0	0	0	0	0	0	0	0	0	k__Heunggongvirae;p__Uroviricota;c__Caudoviricetes;o__;f__;g__;s__Siphoviridae_sp._ctf8W5
NCBI2826375	0	0	0	0	0	0	0	0	0	0	0	1	0	0	0	k__Heunggongvirae;p__Uroviricota;c__Caudoviricetes;o__;f__;g__;s__Siphoviridae_sp._ctYJD4
NCBI2826461	0	0	0	0	0	0	0	0	0	0	0	2	0	0	0	k__Heunggongvirae;p__Uroviricota;c__Caudoviricetes;o__;f__;g__;s__Siphoviridae_sp._ctoWO12
NCBI2826479	0	0	0	0	0	7	0	0	0	0	0	0	0	0	0	k__Heunggongvirae;p__Uroviricota;c__Caudoviricetes;o__;f__;g__;s__Siphoviridae_sp._ctrgt10

NCBI2826786	0	0	0	0	0	5	0	0	0	0	0	0	0	0	0	k__Heunggongvirae;p__Uroviricota;c__Caudoviricetes;o__;f__;g__;s__Caudovirales_sp._ctt3K6
NCBI2826993	0	0	0	0	0	1	0	0	0	0	0	0	0	0	0	k__Bacteria;p__Pseudomonadota;c__Alphaproteobacteria;o__Hyphomicrobiales;f__Aurantimonadaceae;g__Aureimonas;s__Aureimonas_sp._SA4125
NCBI2827673	0	0	0	0	0	0	0	0	0	0	0	1	0	0	0	k__Heunggongvirae;p__Uroviricota;c__Caudoviricetes;o__;f__;g__;s__Myoviridae_sp._ctIty1
NCBI2827714	0	0	0	0	0	0	0	0	0	0	0	1	0	0	0	k__Heunggongvirae;p__Uroviricota;c__Caudoviricetes;o__;f__;g__;s__Myoviridae_sp._ctu6J18
NCBI2827803	0	0	0	0	0	0	1	0	0	0	0	0	0	0	0	k__Heunggongvirae;p__Uroviricota;c__Caudoviricetes;o__;f__;g__;s__Siphoviridae_sp._ctBLh2
NCBI2827834	0	0	0	0	2	0	2	0	0	0	0	0	0	0	0	k__Heunggongvirae;p__Uroviricota;c__Caudoviricetes;o__;f__;g__;s__Siphoviridae_sp._ctJ0s2
NCBI2828206	0	0	0	0	0	0	0	0	0	0	0	4	0	0	0	k__Heunggongvirae;p__Uroviricota;c__Caudoviricetes;o__;f__;g__;s__Siphoviridae_sp._cta6m1
NCBI2830657	0	0	0	1	1	2	0	0	0	0	0	2	0	0	0	k__Bacteria;p__Bacillota;c__Clostridia;o__Eubacteriales;f__Clostridiaceae;g__;s__Clostridiaceae_bacterium_Marseille-Q4143
NCBI2830659	0	0	0	0	0	24	0	2	0	0	1	6	0	0	0	k__Bacteria;p__Bacillota;c__Clostridia;o__Eubacteriales;f__Clostridiaceae;g__;s__Clostridiaceae_bacterium_Marseille-Q4149
NCBI2830668	0	0	0	0	0	25	1	0	0	0	0	6	0	0	0	k__Bacteria;p__Actinomycetota;c__Coriobacteriia;o__Coriobacteriales;f__Atopobiaceae;g__;s__Atopobiaceae_bacterium_P1
NCBI2830996	0	0	0	4	0	0	0	0	0	0	0	0	0	0	0	k__Bacteria;p__Actinomycetota;c__Actinomycetes;o__Micrococcales;f__Micrococcaceae;g__Arthrobacter;s__Arthrobacter_sp._StoSoilB22
NCBI2831613	0	0	0	1	0	114	0	0	1	13	4	1	16	6	11	k__Heunggongvirae;p__Uroviricota;c__Caudoviricetes;o__;f__Herelleviridae;g__;s__Herelleviridae_sp.

NCBI2831617	0	0	0	0	0	0	1	0	0	0	0	0	0	0	0	k__Heunggongvirae;p__Uroviricota;c__Caudoviricetes;o__Crassvirales;f__;g__;s__CrAss-like_virus_sp.
NCBI2831970	0	0	0	0	1	0	0	0	0	0	0	0	0	0	0	k__Bacteria;p__Actinomycetota;c__Actinomycetes;o__Streptosporangiales;f__Nocardiopsaceae;g__Nocardiopsis;s__Nocardiopsis_eucommiae
NCBI2834406	6	1	1	4	44	72	4	0	0	0	0	0	1	0	0	k__Bacteria;p__Pseudomonadota;c__Gammaproteobacteria;o__Pseudomonadales;f__Pseudomonadaceae;g__Pseudomonas;s__Pseudomonas_sp._RC3H12
NCBI2835787	0	0	0	0	0	1	0	0	0	0	0	0	0	0	0	k__Bacteria;p__Pseudomonadota;c__Alphaproteobacteria;o__Sphingomonadales;f__Sphingosinicellaceae;g__Polymorphobacter;s__Polymorphobacter_megasporae
NCBI2836181	0	4	0	0	0	29	3	0	5	0	0	0	0	0	0	k__Bacteria;p__Pseudomonadota;c__Gammaproteobacteria;o__Moraxellales;f__Moraxellaceae;g__Acinetobacter;s__Acinetobacter_sp._BHS4
NCBI2838237	0	0	0	0	0	1	0	0	0	0	0	0	1	0	2	k__Bacteria;p__Spirochaetota;c__Spirochaetia;o__Leptospirales;f__Leptospiraceae;g__Leptospira;s__Leptospira_sp._severe_002
NCBI2838877	0	0	0	1	8	0	13	0	0	0	0	0	0	0	0	k__Bacteria;p__Bacteroidota;c__Flavobacteriia;o__Flavobacteriales;f__Weeksellaceae;g__Chryseobacterium;s__Chryseobacterium_sp._ZHDP1
NCBI2839983	0	0	2	0	0	34	5	0	0	0	0	0	4	0	0	k__Bacteria;p__Pseudomonadota;c__Betaproteobacteria;o__Burkholderiales;f__Burkholderiaceae;g__Cupriavidus;s__Cupriavidus_sp._EM10
NCBI284016	1	0	0	0	2	13	1	0	0	0	0	0	0	0	0	k__Bacteria;p__Pseudomonadota;c__Alphaproteobacteria;o__Caulobacterales;f__Caulobacteraceae;g__Phenylobacterium;s__Phenylobacterium_zucineum
NCBI2840457	0	0	0	0	11	10	0	0	0	0	2	0	0	0	0	k__Bacteria;p__Actinomycetota;c__Actinomycetes;o__Propionibacteriales;f__Nocardioidaceae;g__Nocardioides;s__Nocardioides_sp._LMS-CY

NCBI2840468	0	0	0	0	0	8	0	0	0	0	0	0	0	0	0	k__Bacteria;p__Pseudomonadota;c__Alphaproteobacteria;o__Caulobacterales;f__Caulobacteraceae;g__Asticcacaulis;s__Asticcacaulis_sp._AND118
NCBI2840474	2	0	0	0	0	5	0	0	0	0	0	0	0	0	0	k__Bacteria;p__Pseudomonadota;c__Alphaproteobacteria;o__Rhodobacterales;f__Paracoccaceae;g__Gemmobacter;s__Gemmobacter_fulva
NCBI2841037	0	0	0	0	0	4	0	0	0	0	0	0	0	0	0	k__Bacteria;p__Bacillota;c__Clostridia;o__Eubacteriales;f__Eubacteriaceae;g__Eubacterium;s__Eubacterium_sp._c-25
NCBI2841063	0	5	0	0	0	12	0	0	0	0	0	0	0	0	0	k__Bacteria;p__Pseudomonadota;c__Gammaproteobacteria;o__Pseudomonadales;f__Pseudomonadaceae;g__Pseudomonas;s__Pseudomonas_sp._SK2
NCBI2841263	0	0	0	0	0	0	0	0	0	0	0	4	0	0	0	k__Bacteria;p__Candidatus_Saccharibacteria;c__;o__;f__;g__Candidatus_Minimicrobia;s__Candidatus_Minimicrobia_naudis
NCBI2841518	0	0	0	0	0	1	0	0	0	0	0	0	0	0	0	k__Bacteria;p__Bacillota;c__Clostridia;o__Eubacteriales;f__Christensenellaceae;g__Christensenella;s__Christensenella_sp._MSJ-20
NCBI2841528	0	0	0	0	0	0	0	1	0	0	0	1	0	0	0	k__Bacteria;p__Bacillota;c__Clostridia;o__Eubacteriales;f__Eubacteriaceae;g__Eubacterium;s__Eubacterium_sp._MSJ-33
NCBI2841594	0	0	0	3	0	2	0	20	0	0	18	0	0	0	0	k__Bacteria;p__Actinomycetota;c__Actinomycetes;o__Micrococcales;f__Microbacteriaceae;g__Curtobacterium;s__Curtobacterium_sp._L6-1
NCBI2842348	0	0	0	0	0	0	0	0	0	0	0	2	0	0	0	k__Bacteria;p__Pseudomonadota;c__Gammaproteobacteria;o__Pseudomonadales;f__Pseudomonadaceae;g__Pseudomonas;s__Pseudomonas_alvandae
NCBI2842349	0	0	0	0	0	0	1	0	0	0	0	0	0	0	0	k__Bacteria;p__Pseudomonadota;c__Gammaproteobacteria;o__Pseudomonadales;f__Pseudomonadaceae;g__Pseudomonas;s__Pseudomonas_asgharzadehiana

NCBI2842356	1	5	0	1	2	7	2	0	0	0	0	0	0	0	3	k__Bacteria;p__Pseudomonadota;c__Gammaproteobacteria;o__Pseudomonadales;f__Pseudomonadaceae;g__Pseudomonas;s__Pseudomonas_xanthosomae
NCBI2842456	51	33	26	62	168	120	33	7	27	9	2	25	8	11	9	k__Bacteria;p__Pseudomonadota;c__Betaproteobacteria;o__Burkholderiales;f__Burkholderiaceae;g__Ralstonia;s__Ralstonia_wenshanensis
NCBI2843216	0	0	0	0	0	6	0	0	0	0	0	0	0	0	0	k__Bacteria;p__Actinomycetota;c__Thermoleophilia;o__Miltoncostaeales;f__Miltoncostaeaceae;g__Miltoncostaea;s__Miltoncostaea_oceani
NCBI28447	0	2	0	0	0	11	0	0	0	0	0	0	0	0	0	k__Bacteria;p__Actinomycetota;c__Actinomycetes;o__Micrococcales;f__Microbacteriaceae;g__Clavibacter;s__Clavibacter_michiganensis
NCBI28448	898	928	955	2772	2286	1872	1470	865	1467	4843	4571	7135	21715	22488	27378	k__Bacteria;p__Pseudomonadota;c__Alphaproteobacteria;o__Rhodospirillales;f__Acetobacteraceae;g__Komagataeibacter;s__Komagataeibacter_xylinus
NCBI28449	0	0	0	0	0	0	0	0	0	0	1	17	0	0	0	k__Bacteria;p__Pseudomonadota;c__Betaproteobacteria;o__Neisseriales;f__Neisseriaceae;g__Neisseria;s__Neisseria_subflava
NCBI28450	0	0	0	1	2	4	1	0	0	0	0	1	0	0	0	k__Bacteria;p__Pseudomonadota;c__Betaproteobacteria;o__Burkholderiales;f__Burkholderiaceae;g__Burkholderia;s__Burkholderia_pseudomallei
NCBI2845128	0	0	7	0	0	0	0	0	0	0	0	7	0	0	0	k__Heunggongvirae;p__Uroviricota;c__Caudoviricetes;o__;f__;g__Ashduovirus;s__Ashduovirus_A2
NCBI2845134	31	10	39	50	5	11	16	20	29	5	23	58	17	16	27	k__Heunggongvirae;p__Uroviricota;c__Caudoviricetes;o__;f__;g__Junavirus;s__Junavirus_LJ
NCBI2845138	0	0	0	0	0	0	12	0	0	0	0	0	0	0	0	k__Heunggongvirae;p__Uroviricota;c__Caudoviricetes;o__;f__;g__Larmunavirus;s__Larmunavirus_Lrm1
NCBI2845143	864	905	936	1161	997	248	1223	965	1219	1238	964	1421	411	429	476	k__Heunggongvirae;p__Uroviricota;c__Caudoviricetes;o__;f__;g__Sukhumvitvirus;s__Sukhumvitvirus_T25
NCBI2	35	33	25	114	267	276	54	14	14	0	14	2	11	11	19	k__Bacteria;p__Bacteroidota;c__Sphingobacteriia;o__Sp

8454															hingobacteriales;f__Sphingobacteriaceae;g__Sphingobacterium;s__Sphingobacterium_multivorum
NCBI2846778	0	0	0	0	0	0	0	0	1	0	0	0	0	0	0	k__Bacteria;p__Bacillota;c__Bacilli;o__Bacillales;f__Bacillaceae;g__Bacillus;s__Bacillus_sp._NP157
NCBI285	73	28	16	7	90	836	24	0	5	0	22	22	0	0	0	k__Bacteria;p__Pseudomonadota;c__Betaproteobacteria;o__Burkholderiales;f__Comamonadaceae;g__Comamonas;s__Comamonas_testosteroni
NCBI2851567	0	0	0	0	0	16	0	0	0	0	0	0	0	0	0	k__Bacteria;p__Actinomycetota;c__Actinomycetes;o__Geodermatophilales;f__Geodermatophilaceae;g__Modestobacter;s__Modestobacter_sp._L9-4
NCBI2852099	0	0	0	0	0	9	0	0	0	0	0	0	0	0	0	k__Bacteria;p__Pseudomonadota;c__Betaproteobacteria;o__Burkholderiales;f__Oxalobacteraceae;g__Massilia;s__Massilia_sp._HC52
NCBI2853257	0	0	0	0	0	2	0	0	0	0	0	0	0	0	0	k__Bacteria;p__Pseudomonadota;c__Betaproteobacteria;o__Burkholderiales;f__Comamonadaceae;g__Comamonas;s__Comamonas_sp._Y33R10-2
NCBI2853439	0	0	0	0	0	1	0	0	0	0	0	0	0	0	0	k__Bacteria;p__Bacteroidota;c__Sphingobacteriia;o__Sphingobacteriales;f__Sphingobacteriaceae;g__Sphingobacterium;s__Sphingobacterium_sp._E70
NCBI285473	0	0	0	0	0	135	0	0	0	0	0	0	0	0	0	k__Bacteria;p__Actinomycetota;c__Actinomycetes;o__Kitasatosporales;f__Streptomycetaceae;g__Streptomyces;s__Streptomyces_rubrolavendulae
NCBI2855434	0	0	0	0	0	2	0	0	0	0	0	0	0	0	0	k__Bacteria;p__Pseudomonadota;c__Gammaproteobacteria;o__Pseudomonadales;f__Pseudomonadaceae;g__Pseudomonas;s__Pseudomonas_sp._AO-1
NCBI2855441	0	0	0	0	3	2	0	0	0	0	0	0	0	0	0	k__Bacteria;p__Pseudomonadota;c__Gammaproteobacteria;o__Oceanospirillales;f__Halomonadaceae;g__Halomonas;s__Halomonas_sp._18071143
NCBI2855689	0	0	0	0	0	31	0	0	0	0	0	0	0	0	0	k__Bacteria;p__Bacteroidota;c__Chitinophagia;o__Chitinophagales;f__Chitinophagaceae;g__Niabella;s__Niabell

															a_sp._I65
NCBI2856555	0	0	0	0	4	4	0	0	0	0	0	0	0	4	0	k__Bacteria;p__Actinomycetota;c__Actinomycetes;o__Micrococcales;f__Micrococcaceae;g__Micrococcus;s__Micrococcus_sp._KD337-16
NCBI28572	61	70	60	25	202	122	55	13	68	19	4	33	17	13	17	k__Fungi;p__Ascomycota;c__Eurotiomycetes;o__Eurotiales;f__Trichocomaceae;g__Talaromyces;s__Talaromyces_funiculosus
NCBI28576	0	0	0	0	0	0	1	0	0	0	0	0	0	0	0	k__Fungi;p__Ascomycota;c__Eurotiomycetes;o__Eurotiales;f__Trichocomaceae;g__Talaromyces;s__Talaromyces_variabilis
NCBI2860286	0	0	0	3	24	11	0	0	0	0	0	0	0	0	0	k__Bacteria;p__Pseudomonadota;c__Betaproteobacteria;o__Burkholderiales;f__Alcaligenaceae;g__Achromobacter;s__Achromobacter_sp._ES-001
NCBI2861280	0	0	0	0	0	294	0	0	0	0	0	0	0	0	0	k__Bacteria;p__Actinomycetota;c__Actinomycetes;o__Micrococcales;f__Microbacteriaceae;g__Microbacterium;s__Microbacterium_sp._PAMC21962
NCBI2861281	0	0	0	0	0	0	0	0	0	0	0	19	0	0	0	k__Bacteria;p__Actinomycetota;c__Actinomycetes;o__Micrococcales;f__Microbacteriaceae;g__Microbacterium;s__Microbacterium_sp._PAMC22086
NCBI2861282	7	9	8	9	33	256	8	0	10	0	0	14	0	0	0	k__Bacteria;p__Pseudomonadota;c__Betaproteobacteria;o__Burkholderiales;f__Oxalobacteraceae;g__Massilia;s__Massilia_sp._NP310
NCBI2861285	2	2	4	1	3	79	2	0	0	1	0	1	0	0	0	k__Bacteria;p__Pseudomonadota;c__Alphaproteobacteria;o__Caulobacterales;f__Caulobacteraceae;g__Brevundimonas;s__Brevundimonas_sp._PAMC22021
NCBI2864102	0	0	0	0	0	7	0	0	0	0	0	0	0	0	0	k__Bacteria;p__Pseudomonadota;c__Alphaproteobacteria;o__Hyphomicrobiales;f__Brucellaceae;g__Pseudochrobactrum;s__Pseudochrobactrum_sp._Wa41.01b-1

NCBI286556	2	0	5	5	16	28	3	6	12	0	0	3	6	9	0	k__Bacteria;p__Bacillota;c__Clostridia;o__Eubacteriales;f__Oscillospiraceae;g__Ethanoligenens;s__uncultured_Ethanoligenens_sp.
NCBI2865673	0	0	0	0	0	0	0	0	0	0	0	1	0	0	0	k__Bacteria;p__Actinomycetota;c__Actinomycetes;o__Kitasatosporales;f__Streptomycetaceae;g__Streptomyces;s__Streptomyces_akebiae
NCBI2865832	0	0	0	0	1	0	0	0	0	0	0	0	0	0	0	k__Bacteria;p__Actinomycetota;c__Actinomycetes;o__Pseudonocardiales;f__Pseudonocardiaceae;g__Amycolatopsis;s__Amycolatopsis_sp._DSM_110486
NCBI2865833	0	0	0	1	0	20	0	0	0	0	0	0	0	0	0	k__Bacteria;p__Actinomycetota;c__Actinomycetes;o__Pseudonocardiales;f__Pseudonocardiaceae;g__Pseudonocardia;s__Pseudonocardia_sp._DSM_110487
NCBI2866201	0	0	2	0	0	0	0	0	0	0	0	0	0	0	0	k__Bacteria;p__Pseudomonadota;c__Gammaproteobacteria;o__Enterobacterales;f__Enterobacteriaceae;g__Enterobacter;s__Enterobacter_sp._Colony194
NCBI2866282	0	0	0	120	82	355	0	0	0	0	0	0	0	0	0	k__Bacteria;p__Pseudomonadota;c__Gammaproteobacteria;o__Pseudomonadales;f__Pseudomonadaceae;g__Pseudomonas;s__Pseudomonas_sp._PS1(2021)
NCBI2866592	0	0	0	0	1	1	0	0	1	0	0	0	0	0	0	k__Bacteria;p__Pseudomonadota;c__Gammaproteobacteria;o__Pseudomonadales;f__Pseudomonadaceae;g__Pseudomonas;s__Pseudomonas_sp._Marseille-Q3773
NCBI2866626	6	0	0	13	9	14	0	0	0	0	0	0	0	0	0	k__Bacteria;p__Pseudomonadota;c__Gammaproteobacteria;o__Pseudomonadales;f__Pseudomonadaceae;g__Pseudomonas;s__Pseudomonas_sp._2hn
NCBI2866627	0	0	0	0	0	0	0	0	1	0	0	0	0	0	0	k__Bacteria;p__Bacillota;c__Bacilli;o__Bacillales;f__Bacillales_Family_X._Incertae_Sedis;g__Hydrogenibacillus;s__Hydrogenibacillus_sp._N12
NCBI2866628	106	256	272	72	357	4190	41	42	83	0	0	0	0	0	0	k__Bacteria;p__Pseudomonadota;c__Betaproteobacteria;o__Burkholderiales;f__Comamonadaceae;g__Diaphorobacter;s__Diaphorobacter_sp._MNS-0

NCBI2866807	0	0	0	0	0	0	2	0	0	0	0	0	0	0	0	k__Bacteria;p__Pseudomonadota;c__Gammaproteobacteria;o__Pseudomonadales;f__Pseudomonadaceae;g__Pseudomonas;s__Pseudomonas_sp._MM213
NCBI286727	0	0	0	0	5	0	0	0	0	0	0	0	0	0	0	k__Bacteria;p__Pseudomonadota;c__Alphaproteobacteria;o__Rhodospirillales;f__Azospirillaceae;g__Azospirillum;s__Azospirillum_oryzae
NCBI287	584	820	1413	240	2820	27870	725	117	311	282	215	3908	187	184	138	k__Bacteria;p__Pseudomonadota;c__Gammaproteobacteria;o__Pseudomonadales;f__Pseudomonadaceae;g__Pseudomonas;s__Pseudomonas_aeruginosa
NCBI2870346	0	1	0	3	13	17	1	0	0	0	1	0	0	0	0	k__Bacteria;p__Pseudomonadota;c__Gammaproteobacteria;o__Enterobacterales;f__Enterobacteriaceae;g__Enterobacter;s__Enterobacter_sp._C2
NCBI2870860	1	1	0	0	3	23	2	0	0	0	0	1	0	0	0	k__Bacteria;p__Pseudomonadota;c__Gammaproteobacteria;o__Pseudomonadales;f__Pseudomonadaceae;g__Pseudomonas;s__Pseudomonas_sp._DNDY-54
NCBI2871165	0	0	0	0	1	0	0	0	0	0	0	0	0	0	0	k__Bacteria;p__Bacteroidota;c__Flavobacteriia;o__Flavobacteriales;f__Flavobacteriaceae;g__Flavobacterium;s__Flavobacterium_sp._CHNK8
NCBI2871487	0	0	0	0	0	9	0	0	0	0	0	0	0	0	0	k__Bacteria;p__Actinomycetota;c__Actinomycetes;o__Kitasatosporales;f__Streptomycetaceae;g__Streptomyces;s__Streptomyces_sp._A144
NCBI2873270	0	0	0	0	0	2	0	0	0	0	0	3	0	0	0	k__Bacteria;p__Actinomycetota;c__Actinomycetes;o__Micrococcales;f__Microbacteriaceae;g__Leucobacter;s__Leucobacter_sp._NB_10
NCBI2874282	0	0	0	0	1	0	0	0	0	0	0	0	0	0	0	k__Bacteria;p__Bacillota;c__Bacilli;o__Bacillales;f__Bacillaceae;g__Metabacillus;s__Metabacillus_dongyingensis
NCBI2874628	0	0	0	0	4	6	0	0	0	0	0	0	0	0	0	k__Bacteria;p__Pseudomonadota;c__Gammaproteobacteria;o__Pseudomonadales;f__Pseudomonadaceae;g__Pseudomonas;s__Pseudomonas_sp._p1(2021b)

NCBI2875729	0	0	0	0	0	2	0	0	0	0	0	1	0	0	0	k__Bacteria;p__Actinomycetota;c__Actinomycetes;o__Micrococcales;f__Microbacteriaceae;g__Leucobacter;s__Leucobacter_sp._Psy1
NCBI2877001	2	14	2	0	30	132	2	0	0	0	0	0	3	0	8	k__Bacteria;p__Pseudomonadota;c__Alphaproteobacteria;o__Sphingomonadales;f__Erythrobacteraceae;g__Altererythrobacter;s__Altererythrobacter_sp._N1
NCBI2877527	0	0	0	0	3	15	0	0	0	0	0	2	0	0	0	k__Bacteria;p__Bacillota;c__Clostridia;o__Eubacteriales;f__Lachnospiraceae;g__Blautia;s__Blautia_sp._NBRC_113351
NCBI2877939	55	61	42	0	133	1268	68	3	26	5	5	32	6	2	6	k__Bacteria;p__Pseudomonadota;c__Betaproteobacteria;o__Neisseriales;f__Chromobacteriaceae;g__Vogesella;s__Vogesella_sp._XCS3
NCBI2877940	0	0	0	0	6	53	0	0	0	0	0	0	0	0	0	k__Bacteria;p__Pseudomonadota;c__Betaproteobacteria;o__Burkholderiales;f__Comamonadaceae;g__Kinneretia;s__Kinneretia_sp._XES5
NCBI2877941	1	0	0	0	1	85	5	0	0	2	2	0	0	0	0	k__Bacteria;p__Pseudomonadota;c__Alphaproteobacteria;o__Hyphomicrobiales;f__Rhizobiaceae;g__Shinella;s__Shinella_sp._XGS7
NCBI2878678	2	0	0	0	0	0	0	0	0	0	0	0	0	0	0	k__Bacteria;p__Actinomycetota;c__Actinomycetes;o__Mycobacteriales;f__Gordoniaceae;g__Gordonia;s__Gordonia_sp._WA4-43
NCBI2879116	1	0	0	0	0	0	0	0	0	0	0	0	0	0	0	k__Bacteria;p__Pseudomonadota;c__Alphaproteobacteria;o__Hyphomicrobiales;f__Brucellaceae;g__Pseudochrobactrum;s__Pseudochrobactrum_sp._XF203
NCBI2879427	0	0	0	0	0	6	0	0	0	0	0	0	0	0	0	k__Bacteria;p__Actinomycetota;c__Actinomycetes;o__Kitasatosporales;f__Streptomycetaceae;g__Streptomyces;s__Streptomyces_sp._WA6-1-16
NCBI288000	9	5	6	33	59	107	20	3	5	3	1	2	4	6	16	k__Bacteria;p__Pseudomonadota;c__Alphaproteobacteria;o__Hyphomicrobiales;f__Nitrobacteraceae;g__Bradyrhizobium;s__Bradyrhizobium_sp._BTAi1

NCBI2883106	12	13	13	0	33	473	9	0	7	1	1	10	0	0	5	k__Bacteria;p__Pseudomonadota;c__Gammaproteobacteria;o__Oceanospirillales;f__Halomonadaceae;g__Halomonas;s__Halomonas_sp._NyZ770
NCBI2883205	0	0	0	0	0	1	0	0	0	0	0	0	0	0	0	k__Bacteria;p__Pseudomonadota;c__Gammaproteobacteria;o__Pseudomonadales;f__Pseudomonadaceae;g__Pseudomonas;s__Pseudomonas_sp._L5B5
NCBI2883480	0	0	0	1	0	1	0	0	0	0	0	0	0	0	0	k__Bacteria;p__Pseudomonadota;c__Alphaproteobacteria;o__Sphingomonadales;f__Erythrobacteraceae;g__Porphyrobacter;s__Porphyrobacter_sp._GA68
NCBI2885078	0	0	0	0	0	2	0	0	0	0	0	12	0	0	0	k__Bacteria;p__Actinomycetota;c__Actinomycetes;o__Mycobacteriales;f__Nocardiaceae;g__Rhodococcus;s__Rhodococcus_sp._RDE2
NCBI2886360	0	0	0	0	2	2	332	0	16	0	0	0	0	0	0	k__Bacteria;p__Pseudomonadota;c__Gammaproteobacteria;o__Pseudomonadales;f__Pseudomonadaceae;g__Pseudomonas;s__Pseudomonas_sp._HN2-3
NCBI28901	0	5	0	35	13	175	7	0	0	0	0	566	0	0	5	k__Bacteria;p__Pseudomonadota;c__Gammaproteobacteria;o__Enterobacterales;f__Enterobacteriaceae;g__Salmonella;s__Salmonella_enterica
NCBI28903	0	0	0	0	1	0	0	0	0	0	0	0	0	0	0	k__Bacteria;p__Mycoplasmatota;c__Mollicutes;o__Mycoplasmatales;f__Mycoplasmataceae;g__Mycoplasmopsis;s__Mycoplasmopsis_bovis
NCBI2894079	4	8	3	0	5	42	12	0	2	2	0	0	0	2	2	k__Bacteria;p__Pseudomonadota;c__Gammaproteobacteria;o__Pseudomonadales;f__Pseudomonadaceae;g__Pseudomonas;s__Pseudomonas_oryziphila
NCBI2895796	0	0	0	0	0	5	0	0	0	0	0	0	0	0	0	k__Bacteria;p__Pseudomonadota;c__Alphaproteobacteria;o__Rhodobacterales;f__Paracoccaceae;g__Paracoccus;s__Paracoccus_sp._MA
NCBI2895947	0	0	0	0	0	12	0	0	0	0	0	0	0	0	0	k__Bacteria;p__Bacteroidota;c__Flavobacteriia;o__Flavobacteriales;f__Flavobacteriaceae;g__Flavobacterium;s__Flavobacterium_cyclinae

NCBI2897181	0	0	0	0	0	0	0	0	0	2	0	0	0	0	0	k__Bacteria;p__Bacteroidota;c__Flavobacteriia;o__Flavobacteriales;f__Flavobacteriaceae;g__Flavobacterium;s__Flavobacterium_channae
NCBI2897334	0	4	8	36	28	37	2	3	0	0	0	0	0	0	0	k__Bacteria;p__Pseudomonadota;c__Alphaproteobacteria;o__Hyphomicrobiales;f__Methylobacteriaceae;g__Methylorubrum;s__Methylorubrum_sp._B1-46
NCBI2898151	0	0	0	0	0	12	0	0	0	0	8	0	0	0	0	k__Bacteria;p__Actinomycetota;c__Actinomycetes;o__Micrococcales;f__Microbacteriaceae;g__Curtobacterium;s__Curtobacterium_sp._C1
NCBI2898187	0	0	0	0	0	2	0	0	0	0	0	0	0	0	0	k__Bacteria;p__Bacteroidota;c__Flavobacteriia;o__Flavobacteriales;f__Weeksellaceae;g__Faecalibacter;s__Faecalibacter_bovis
NCBI28985	1	0	0	0	0	0	0	0	0	0	1	0	0	0	0	k__Fungi;p__Ascomycota;c__Saccharomycetes;o__Saccharomycetales;f__Saccharomycetaceae;g__Kluyveromyces;s__Kluyveromyces_lactis
NCBI2898725	0	6	0	9	16	9	0	0	0	0	0	0	0	0	0	k__Bacteria;p__Pseudomonadota;c__Gammaproteobacteria;o__Enterobacterales;f__Enterobacteriaceae;g__Leclercia;s__Leclercia_sp._G3L
NCBI2899220	0	0	0	0	3	18	0	0	0	0	0	0	0	0	0	k__Bacteria;p__Pseudomonadota;c__Betaproteobacteria;o__Burkholderiales;f__Oxalobacteraceae;g__Massilia;s__Massilia_sp._DM-R-R2A-13
NCBI290053	0	0	0	0	1	0	0	0	0	0	0	1	0	0	0	k__Bacteria;p__Bacteroidota;c__Bacteroidia;o__Bacteroidales;f__Bacteroidaceae;g__Bacteroides;s__Bacteroides_helcogenes
NCBI2900548	0	0	0	0	0	0	0	0	1	0	0	1	0	0	0	k__Bacteria;p__Actinomycetota;c__;o__;f__;g__;s__Actinomycetota_bacterium
NCBI2901380	0	0	0	0	0	1	0	0	0	0	0	1	0	0	0	k__Bacteria;p__Pseudomonadota;c__Gammaproteobacteria;o__Pseudomonadales;f__Pseudomonadaceae;g__Pseudomonas;s__Pseudomonas_sp._NIBR-H-19

NCBI2901869	0	0	0	0	0	0	0	0	0	0	0	1	0	0	0	k__Bacteria;p__Pseudomonadota;c__Gammaproteobacteria;o__Xanthomonadales;f__Xanthomonadaceae;g__Luteimonas;s__Luteimonas_fraxinea
NCBI2904253	0	0	0	0	3	19	1	0	0	0	0	0	0	0	0	k__Bacteria;p__Pseudomonadota;c__Gammaproteobacteria;o__Xanthomonadales;f__Xanthomonadaceae;g__Lysobacter;s__Lysobacter_sp._5GHs7-4
NCBI290602	16	19	21	25	24	109	17	16	22	15	23	20	7	8	5	k__Bacteria;p__Chloroflexota;c__Chloroflexia;o__Chloroflexales;f__Roseiflexaceae;g__Roseiflexus;s__uncultured_Roseiflexus_sp.
NCBI291644	0	0	1	0	0	0	0	0	0	0	0	0	0	0	0	k__Bacteria;p__Bacteroidota;c__Bacteroidia;o__Bacteroidales;f__Bacteroidaceae;g__Bacteroides;s__Bacteroides_salyersiae
NCBI291645	0	0	0	0	0	0	5	0	0	0	0	0	0	0	0	k__Bacteria;p__Bacteroidota;c__Bacteroidia;o__Bacteroidales;f__Bacteroidaceae;g__Bacteroides;s__Bacteroides_nordii
NCBI292800	0	0	2	2	2	49	1	0	0	0	0	8	0	0	0	k__Bacteria;p__Bacillota;c__Clostridia;o__Eubacteriales;f__Oscillospiraceae;g__Flavonifractor;s__Flavonifractor_plautii
NCBI292913	1	0	5	1	12	134	6	0	0	0	0	1	0	0	0	k__Bacteria;p__Pseudomonadota;c__Alphaproteobacteria;o__Sphingomonadales;f__Sphingomonadaceae;g__Sphingopyxis;s__Sphingopyxis_sp._113P3
NCBI293	184	189	159	582	1641	4132	307	35	85	33	18	73	28	37	32	k__Bacteria;p__Pseudomonadota;c__Alphaproteobacteria;o__Caulobacterales;f__Caulobacteraceae;g__Brevundimonas;s__Brevundimonas_diminuta
NCBI293089	0	0	0	0	0	12	0	0	0	0	0	0	0	0	0	k__Bacteria;p__Pseudomonadota;c__Alphaproteobacteria;o__Hyphomicrobiales;f__Aurantimonadaceae;g__Martelella;s__Martelella_mediterranea
NCBI293091	2	0	0	0	1	1	0	0	0	0	0	0	0	0	0	k__Archaea;p__Euryarchaeota;c__Halobacteria;o__Haloferacales;f__Haloferacaceae;g__Haloquadratum;s__Haloquadratum_walsbyi

NCBI293227	0	0	0	0	1	0	0	0	0	0	0	0	0	0	0	k__Fungi;p__Ascomycota;c__Eurotiomycetes;o__Chaetothyriales;f__Cyphellophoraceae;g__Cyphellophora;s__Cyphellophora_europaea
NCBI29347	0	0	0	0	2	97	18	0	1	0	0	1	0	0	0	k__Bacteria;p__Bacillota;c__Clostridia;o__Eubacteriales;f__Lachnospiraceae;g__Lachnoclostridium;s__[Clostridium]_scindens
NCBI29382	2	0	0	2	0	1	0	3	0	0	0	0	0	0	0	k__Bacteria;p__Bacillota;c__Bacilli;o__Bacillales;f__Staphylococcaceae;g__Staphylococcus;s__Staphylococcus_cohnii
NCBI29384	0	0	0	0	1	0	0	0	0	0	0	0	0	0	0	k__Bacteria;p__Bacillota;c__Bacilli;o__Bacillales;f__Staphylococcaceae;g__Staphylococcus;s__Staphylococcus_kloosii
NCBI29385	0	0	9	2	0	0	0	0	0	0	0	0	0	0	0	k__Bacteria;p__Bacillota;c__Bacilli;o__Bacillales;f__Staphylococcaceae;g__Staphylococcus;s__Staphylococcus_saprophyticus
NCBI29388	1	2	1	8	9	111	3	3	1	0	0	6	0	0	0	k__Bacteria;p__Bacillota;c__Bacilli;o__Bacillales;f__Staphylococcaceae;g__Staphylococcus;s__Staphylococcus_capitis
NCBI29389	0	0	0	0	0	71	0	0	0	0	0	6	0	0	0	k__Bacteria;p__Bacillota;c__Bacilli;o__Lactobacillales;f__Streptococcaceae;g__Streptococcus;s__Streptococcus_alactolyticus
NCBI29394	0	0	0	0	0	2	0	0	0	0	0	0	0	0	0	k__Bacteria;p__Bacillota;c__Bacilli;o__Lactobacillales;f__Carnobacteriaceae;g__Dolosigranulum;s__Dolosigranulum_pigrum
NCBI294	8	0	2	13	97	107	95	0	12	0	0	0	0	0	6	k__Bacteria;p__Pseudomonadota;c__Gammaproteobacteria;o__Pseudomonadales;f__Pseudomonadaceae;g__Pseudomonas;s__Pseudomonas_fluorescens
NCBI29408	0	0	0	0	0	6	0	0	0	0	0	0	0	0	0	k__Bacteria;p__Pseudomonadota;c__Alphaproteobacteria;o__Hyphomicrobiales;f__Hyphomicrobiaceae;g__Rhodoplanes;s__Rhodoplanes_elegans

NCBI29430	10	22	5	1	12	66	16	0	0	0	1	5	5	4	0	k__Bacteria;p__Pseudomonadota;c__Gammaproteobacteria;o__Moraxellales;f__Moraxellaceae;g__Acinetobacter;s__Acinetobacter_haemolyticus
NCBI29438	0	0	0	0	700	2169	0	0	0	0	0	0	0	0	0	k__Bacteria;p__Pseudomonadota;c__Gammaproteobacteria;o__Pseudomonadales;f__Pseudomonadaceae;g__Pseudomonas;s__Pseudomonas_savastanoi
NCBI29442	0	0	0	0	1	0	0	0	0	0	0	0	0	0	0	k__Bacteria;p__Pseudomonadota;c__Gammaproteobacteria;o__Pseudomonadales;f__Pseudomonadaceae;g__Pseudomonas;s__Pseudomonas_tolaasii
NCBI29443	0	0	0	0	1	0	0	0	0	0	0	0	0	0	0	k__Bacteria;p__Pseudomonadota;c__Betaproteobacteria;o__Burkholderiales;f__Burkholderiaceae;g__Paucimonas;s__Paucimonas_lemoignei
NCBI29447	1	0	0	0	0	23	0	1	0	0	0	16	0	0	0	k__Bacteria;p__Pseudomonadota;c__Gammaproteobacteria;o__Xanthomonadales;f__Xanthomonadaceae;g__Xanthomonas;s__Xanthomonas_albilineans
NCBI29448	0	0	0	0	0	0	0	0	0	0	0	0	0	0	15	k__Bacteria;p__Pseudomonadota;c__Alphaproteobacteria;o__Hyphomicrobiales;f__Nitrobacteraceae;g__Bradyrhizobium;s__Bradyrhizobium_elkanii
NCBI29449	0	0	0	0	0	7	0	0	0	0	0	0	0	0	0	k__Bacteria;p__Pseudomonadota;c__Alphaproteobacteria;o__Hyphomicrobiales;f__Rhizobiaceae;g__Rhizobium;s__Rhizobium_etli
NCBI29459	0	0	0	0	0	0	237	0	0	0	0	0	0	0	0	k__Bacteria;p__Pseudomonadota;c__Alphaproteobacteria;o__Hyphomicrobiales;f__Brucellaceae;g__Brucella;s__Brucella_melitensis
NCBI29466	1	0	0	4	4	21	3	29	0	0	0	22	0	0	0	k__Bacteria;p__Bacillota;c__Negativicutes;o__Veillonellales;f__Veillonellaceae;g__Veillonella;s__Veillonella_parvula
NCBI294699	0	0	0	0	0	3	0	0	0	0	0	26	0	0	0	k__Bacteria;p__Bacillota;c__Bacilli;o__Bacillales;f__Bacillaceae;g__Anoxybacillus;s__Anoxybacillus_amylolyticus

NCBI29486	0	0	0	1	0	0	0	0	0	0	0	0	0	0	0	k__Bacteria;p__Pseudomonadota;c__Gammaproteobacteria;o__Enterobacterales;f__Yersiniaceae;g__Yersinia;s__Yersinia_ruckeri
NCBI29489	0	0	0	4	0	0	0	0	0	0	0	65	0	0	0	k__Bacteria;p__Pseudomonadota;c__Gammaproteobacteria;o__Aeromonadales;f__Aeromonadaceae;g__Aeromonas;s__Aeromonas_enteropelogenes
NCBI29570	0	0	0	0	0	4	0	0	0	0	0	0	0	0	0	k__Bacteria;p__Pseudomonadota;c__Gammaproteobacteria;o__Oceanospirillales;f__Halomonadaceae;g__Halomonas;s__Halomonas_meridiana
NCBI296	0	0	0	0	0	2	3	0	0	0	96	3	0	0	0	k__Bacteria;p__Pseudomonadota;c__Gammaproteobacteria;o__Pseudomonadales;f__Pseudomonadaceae;g__Pseudomonas;s__Pseudomonas_fragi
NCBI296591	0	2	0	0	1	21	2	0	0	0	0	0	0	0	0	k__Bacteria;p__Pseudomonadota;c__Betaproteobacteria;o__Burkholderiales;f__Comamonadaceae;g__Polaromonas;s__Polaromonas_sp._JS666
NCBI296745	0	0	0	0	0	2	0	0	0	0	2	0	0	0	0	k__Bacteria;p__Bacillota;c__Bacilli;o__Bacillales;f__Bacillaceae;g__Caldalkalibacillus;s__Caldalkalibacillus_thermarum
NCBI296842	0	0	0	0	0	1	0	0	0	0	0	0	0	0	0	k__Bacteria;p__Thermodesulfobacteriota;c__Desulfovibrionia;o__Desulfovibrionales;f__Desulfovibrionaceae;g__Solidesulfovibrio;s__Solidesulfovibrio_carbinolicus
NCBI297	0	0	1	0	0	2	0	0	2	0	0	0	0	0	1	k__Bacteria;p__Pseudomonadota;c__Hydrogenophilia;o__Hydrogenophilales;f__Hydrogenophilaceae;g__Hydrogenophilus;s__Hydrogenophilus_thermoluteolus
NCBI29833	0	22	0	0	0	0	0	0	0	0	0	0	0	0	0	k__Fungi;p__Ascomycota;c__Saccharomycetes;o__Saccharomycetales;f__Saccharomycodaceae;g__Hanseniaspora;s__Hanseniaspora_uvarum
NCBI29898	20	12	12	40	108	111	10	1	3	1	1	2	0	0	0	k__Fungi;p__Basidiomycota;c__Microbotryomycetes;o__Sporidiobolales;f__Sporidiobolaceae;g__Rhodotorula;s__Rhodotorula_graminis

NCBI29917	0	0	0	12	0	0	0	0	0	0	0	0	0	0	0	k__Fungi;p__Ascomycota;c__Dothideomycetes;o__Cladosporiales;f__Cladosporiaceae;g__Cladosporium;s__Cladosporium_cladosporioides
NCBI299262	0	0	0	0	0	1	0	0	0	0	0	0	0	0	0	k__Bacteria;p__Pseudomonadota;c__Alphaproteobacteria;o__Rhodobacterales;f__Roseobacteraceae;g__Tateyamaria;s__Tateyamaria_omphalii
NCBI300	9	7	2	0	11	111	4	0	1	1	0	7	0	7	0	k__Bacteria;p__Pseudomonadota;c__Gammaproteobacteria;o__Pseudomonadales;f__Pseudomonadaceae;g__Pseudomonas;s__Pseudomonas_mendocina
NCBI300019	0	0	0	0	0	36	0	0	0	0	0	0	0	0	0	k__Bacteria;p__Actinomycetota;c__Actinomycetes;o__Micrococcales;f__Microbacteriaceae;g__Microbacterium;s__Microbacterium_paludicola
NCBI300028	0	0	0	1	0	0	0	0	0	0	0	0	0	0	0	k__Bacteria;p__Actinomycetota;c__Actinomycetes;o__Mycobacteriales;f__Nocardiaceae;g__Rhodococcus;s__Rhodococcus_triatomae
NCBI300181	0	0	0	0	16	0	0	0	0	0	0	0	0	0	0	k__Bacteria;p__Pseudomonadota;c__Gammaproteobacteria;o__Enterobacterales;f__Yersiniaceae;g__Serratia;s__Serratia_ureilytica
NCBI300300	0	0	0	0	0	2	0	0	0	0	0	0	0	0	0	k__Bacteria;p__Pseudomonadota;c__Alphaproteobacteria;o__Hyphomicrobiales;f__Rhizobiaceae;g__Agrobacterium;s__Agrobacterium_viscosum
NCBI301	9	13	3	0	54	339	6	0	0	0	0	0	0	0	0	k__Bacteria;p__Pseudomonadota;c__Gammaproteobacteria;o__Pseudomonadales;f__Pseudomonadaceae;g__Pseudomonas;s__Pseudomonas_oleovorans
NCBI301301	0	0	1	3	0	4	0	0	0	0	0	4	0	0	0	k__Bacteria;p__Bacillota;c__Clostridia;o__Eubacteriales;f__Lachnospiraceae;g__Roseburia;s__Roseburia_hominis
NCBI301530	33	22	6	22	113	232	0	0	0	0	0	0	0	0	0	k__Fungi;p__Basidiomycota;c__Agaricomycetes;o__Cantharellales;f__Ceratobasidiaceae;g__Rhizoctonia;s__Rhizoctonia_fragariae

NCBI303	110	100	91	955	1571	2051	287	28	138	14	40	199	49	17	33	k__Bacteria;p__Pseudomonadota;c__Gammaproteobacteria;o__Pseudomonadales;f__Pseudomonadaceae;g__Pseudomonas;s__Pseudomonas_putida
NCBI303541	0	0	0	0	0	3	0	0	0	0	0	0	0	0	0	k__Bacteria;p__Bacillota;c__Bacilli;o__Lactobacillales;f__Lactobacillaceae;g__Lactobacillus;s__Lactobacillus_apis
NCBI304207	458882	464962	488092	762634	574499	304834	687778	511728	697416	700093	662329	994563	443869	491376	534508	k__Bacteria;p__Bacillota;c__Bacilli;o__Lactobacillales;f__Lactobacillaceae;g__Schleiferilactobacillus;s__Schleiferilactobacillus_harbinensis
NCBI304378	6	12	30	0	41	543	34	0	0	24	0	28	6	9	0	k__Bacteria;p__Pseudomonadota;c__Alphaproteobacteria;o__Sphingomonadales;f__Sphingomonadaceae;g__Sphingomonas;s__Sphingomonas_sp._IC081
NCBI305	149	90	78	200	379	450	110	33	53	39	40	31	43	21	22	k__Bacteria;p__Pseudomonadota;c__Betaproteobacteria;o__Burkholderiales;f__Burkholderiaceae;g__Ralstonia;s__Ralstonia_solanacearum
NCBI306	0	0	0	0	0	265	0	0	0	0	0	0	0	0	0	k__Bacteria;p__Pseudomonadota;c__Gammaproteobacteria;o__Pseudomonadales;f__Pseudomonadaceae;g__Pseudomonas;s__Pseudomonas_sp.
NCBI307486	0	0	0	1	1	1	2	1	0	0	0	1	0	0	0	k__Bacteria;p__Pseudomonadota;c__Betaproteobacteria;o__Burkholderiales;f__;g__Tepidimonas;s__Tepidimonas_taiwanensis
NCBI307643	0	0	0	0	0	9	0	0	0	0	0	0	0	0	1	k__Bacteria;p__Bacillota;c__Bacilli;o__Bacillales;f__;g__Exiguobacterium;s__Exiguobacterium_profundum
NCBI310581	0	0	0	0	0	1	0	0	0	0	0	0	0	0	0	k__Bacteria;p__Pseudomonadota;c__Alphaproteobacteria;o__Sphingomonadales;f__Sphingomonadaceae;g__Sphingopyxis;s__uncultured_Sphingopyxis_sp.
NCBI310767	0	1	0	0	0	1	0	0	0	0	0	2	0	0	0	k__Bacteria;p__Pseudomonadota;c__Betaproteobacteria;o__Rhodocyclales;f__Zoogloeaceae;g__Zoogloea;s__Zoogloea_oryzae

NCBI311180	0	0	0	0	0	0	0	0	0	0	2	16	0	0	0	k__Bacteria;p__Pseudomonadota;c__Alphaproteobacteria;o__Rhodobacterales;f__Roseobacteraceae;g__Yangia;s__Yangia_pacifica
NCBI311182	0	0	0	0	2	2	0	0	0	0	0	0	0	0	0	k__Bacteria;p__Pseudomonadota;c__Betaproteobacteria;o__Nitrosomonadales;f__Sterolibacteriaceae;g__Denitratisoma;s__Denitratisoma_oestradiolicum
NCBI312279	0	0	0	0	1	0	0	0	0	0	0	0	0	0	0	k__Bacteria;p__Bacteroidota;c__Cytophagia;o__Cytophagales;f__Spirosomaceae;g__Emticicia;s__Emticicia_oligotrophica
NCBI312306	6	17	1	47	93	132	14	0	5	1	4	0	0	5	0	k__Bacteria;p__Pseudomonadota;c__Gammaproteobacteria;o__Pseudomonadales;f__Pseudomonadaceae;g__Pseudomonas;s__Pseudomonas_entomophila
NCBI313409	0	0	0	0	0	0	0	0	0	3	0	0	0	0	0	k__Bacteria;p__Pseudomonadota;c__Alphaproteobacteria;o__Hyphomicrobiales;f__Beijerinckiaceae;g__Beijerinckia;s__uncultured_Beijerinckia_sp.
NCBI314100	0	0	0	0	2	0	3	0	2	0	0	0	0	0	0	k__Bacteria;p__;c__;o__;f__;g__;s__uncultured_murine_large_bowel_bacterium_BAC_31B
NCBI314722	0	0	0	0	0	2	0	0	1	0	0	0	0	0	0	k__Bacteria;p__Pseudomonadota;c__Gammaproteobacteria;o__Xanthomonadales;f__Xanthomonadaceae;g__Pseudoxanthomonas;s__Pseudoxanthomonas_suwonensis
NCBI315405	0	0	0	0	0	91	0	0	0	0	0	0	0	0	0	k__Bacteria;p__Bacillota;c__Bacilli;o__Lactobacillales;f__Streptococcaceae;g__Streptococcus;s__Streptococcus_gallolyticus
NCBI316	2665	2539	1263	197	6563	86457	2980	223	996	479	419	1178	438	263	570	k__Bacteria;p__Pseudomonadota;c__Gammaproteobacteria;o__Pseudomonadales;f__Pseudomonadaceae;g__Stutzerimonas;s__Stutzerimonas_stutzeri
NCBI317	0	5	0	19	22	46	0	0	5	0	0	0	0	0	0	k__Bacteria;p__Pseudomonadota;c__Gammaproteobacteria;o__Pseudomonadales;f__Pseudomonadaceae;g__Pseudomonas;s__Pseudomonas_syringae

NCBI317577	0	1	0	4	0	3	2	0	0	0	0	1	0	0	0	k__Bacteria;p__Deinococcota;c__Deinococci;o__Deinococcales;f__Deinococcaceae;g__Deinococcus;s__Deinococcus_ficus
NCBI318683	3405	3422	3557	9728	9348	6910	5444	3696	5148	24271	21843	34551	31830	34010	39868	k__Bacteria;p__Pseudomonadota;c__Alphaproteobacteria;o__Rhodospirillales;f__Acetobacteraceae;g__Gluconobacter;s__Gluconobacter_albidus
NCBI31870	0	0	0	0	0	1	0	0	0	0	0	1	0	0	0	k__Fungi;p__Ascomycota;c__Sordariomycetes;o__Glomerellales;f__Glomerellaceae;g__Colletotrichum;s__Colletotrichum_graminicola
NCBI318829	0	0	0	0	0	0	3	0	0	0	0	0	0	0	0	k__Fungi;p__Ascomycota;c__Sordariomycetes;o__Magnaporthales;f__Pyriculariaceae;g__Pyricularia;s__Pyricularia_oryzae
NCBI319707	3	3	2	1	0	44	0	0	3	0	4	3	6	1	6	k__Bacteria;p__Actinomycetota;c__Actinomycetes;o__Mycobacteriales;f__Mycobacteriaceae;g__Mycolicibacterium;s__Mycolicibacterium_aubagnense
NCBI319939	10	16	4	61	183	208	28	3	9	6	0	3	4	1	1	k__Bacteria;p__Pseudomonadota;c__Gammaproteobacteria;o__Pseudomonadales;f__Pseudomonadaceae;g__Pseudomonas;s__Pseudomonas_otitidis
NCBI31998	3	0	0	9	38	0	0	0	5	0	0	0	0	0	0	k__Bacteria;p__Pseudomonadota;c__Alphaproteobacteria;o__Hyphomicrobiales;f__Methylobacteriaceae;g__Methylobacterium;s__Methylobacterium_radiotolerans
NCBI32002	2	0	6	3	9	50	0	0	0	0	0	0	0	0	0	k__Bacteria;p__Pseudomonadota;c__Betaproteobacteria;o__Burkholderiales;f__Alcaligenaceae;g__Achromobacter;s__Achromobacter_denitrificans
NCBI32013	344	325	281	1540	3536	6084	498	100	228	43	29	73	131	53	77	k__Bacteria;p__Pseudomonadota;c__Betaproteobacteria;o__Burkholderiales;f__Comamonadaceae;g__Comamonas;s__Comamonas_terrigena
NCBI32025	0	0	0	0	0	0	0	0	0	0	0	1	0	0	0	k__Bacteria;p__Campylobacterota;c__Epsilonproteobacteria;o__Campylobacterales;f__Helicobacteraceae;g__Helicobacter;s__Helicobacter_hepaticus

NCBI320497	465	423	444	1349	1293	998	787	458	797	3481	3166	4956	4671	4852	5597	k__Bacteria;p__Pseudomonadota;c__Alphaproteobacteria;o__Rhodospirillales;f__Acetobacteraceae;g__Neoasaia;s__Neoasaia_chiangmaiensis
NCBI321662	0	0	0	0	0	0	30	0	0	0	0	0	0	0	0	k__Bacteria;p__Pseudomonadota;c__Gammaproteobacteria;o__Pseudomonadales;f__Pseudomonadaceae;g__Pseudomonas;s__Pseudomonas_moraviensis
NCBI321846	3	3	0	22	36	70	4	1	4	0	0	1	0	0	4	k__Bacteria;p__Pseudomonadota;c__Gammaproteobacteria;o__Pseudomonadales;f__Pseudomonadaceae;g__Pseudomonas;s__Pseudomonas_simiae
NCBI321983	0	0	0	0	0	8	0	0	0	0	0	0	0	0	0	k__Bacteria;p__Pseudomonadota;c__Betaproteobacteria;o__Burkholderiales;f__Oxalobacteraceae;g__Pseudoduganella;s__Pseudoduganella_albidiflava
NCBI321985	0	0	0	0	5	5	0	0	0	0	0	0	0	0	0	k__Bacteria;p__Pseudomonadota;c__Betaproteobacteria;o__Burkholderiales;f__Oxalobacteraceae;g__Pseudoduganella;s__Pseudoduganella_lutea
NCBI322596	0	0	0	1	0	0	0	0	0	0	0	0	0	0	0	k__Bacteria;p__Actinomycetota;c__Actinomycetes;o__Micrococcales;f__Dermacoccaceae;g__Dermacoccus;s__Dermacoccus_abyssi
NCBI323284	0	4	0	2	0	0	3	0	0	0	0	0	0	0	0	k__Bacteria;p__Pseudomonadota;c__Betaproteobacteria;o__Burkholderiales;f__Alcaligenaceae;g__Alcaligenes;s__Alcaligenes_aquatilis
NCBI327575	0	0	0	0	1	0	0	0	0	0	0	0	0	0	0	k__Bacteria;p__Bacteroidota;c__Flavobacteriia;o__Flavobacteriales;f__Flavobacteriaceae;g__Capnocytophaga;s__Capnocytophaga_leadbetteri
NCBI328812	0	0	0	0	1	2	6	0	0	0	0	5	0	0	0	k__Bacteria;p__Bacteroidota;c__Bacteroidia;o__Bacteroidales;f__Tannerellaceae;g__Parabacteroides;s__Parabacteroides_goldsteinii
NCBI328813	0	0	1	0	0	38	0	1	0	0	0	23	0	0	0	k__Bacteria;p__Bacteroidota;c__Bacteroidia;o__Bacteroidales;f__Rikenellaceae;g__Alistipes;s__Alistipes_onderdonkii

NCBI328814	1	0	0	2	6	19	21	0	0	0	0	31	0	0	0	k__Bacteria;p__Bacteroidota;c__Bacteroidia;o__Bacteroidales;f__Rikenellaceae;g__Alistipes;s__Alistipes_shahii
NCBI329	1124	951	782	1461	3557	3855	1305	280	596	271	304	521	195	201	307	k__Bacteria;p__Pseudomonadota;c__Betaproteobacteria;o__Burkholderiales;f__Burkholderiaceae;g__Ralstonia;s__Ralstonia_pickettii
NCBI329854	0	0	1	0	0	11	0	0	0	0	0	4	0	0	0	k__Bacteria;p__Bacteroidota;c__Bacteroidia;o__Bacteroidales;f__Bacteroidaceae;g__Bacteroides;s__Bacteroides_intestinalis
NCBI33010	0	0	1	1	2	24	0	0	1	0	0	0	0	0	0	k__Bacteria;p__Actinomycetota;c__Actinomycetes;o__Propionibacteriales;f__Propionibacteriaceae;g__Cutibacterium;s__Cutibacterium_avidum
NCBI33011	4	0	1	29	5	152	0	5	2	0	0	10	0	0	1	k__Bacteria;p__Actinomycetota;c__Actinomycetes;o__Propionibacteriales;f__Propionibacteriaceae;g__Cutibacterium;s__Cutibacterium_granulosum
NCBI330214	0	0	0	0	2	45	4	0	0	0	0	0	0	0	0	k__Bacteria;p__Nitrospirota;c__Nitrospiria;o__Nitrospirales;f__Nitrospiraceae;g__Nitrospira;s__Nitrospira_defluvii
NCBI33025	0	0	0	0	0	21	0	0	0	0	0	7	0	0	0	k__Bacteria;p__Bacillota;c__Negativicutes;o__Acidaminococcales;f__Acidaminococcaceae;g__Phascolarctobacterium;s__Phascolarctobacterium_faecium
NCBI33028	0	3	0	9	0	0	0	0	0	0	0	0	0	0	0	k__Bacteria;p__Bacillota;c__Bacilli;o__Bacillales;f__Staphylococcaceae;g__Staphylococcus;s__Staphylococcus_saccharolyticus
NCBI33034	0	0	0	0	0	0	0	0	0	0	0	1	0	0	0	k__Bacteria;p__Bacillota;c__Tissierellia;o__Tissierellales;f__Peptoniphilaceae;g__Anaerococcus;s__Anaerococcus_prevotii
NCBI33035	0	0	0	0	0	20	0	0	0	0	0	4	0	0	0	k__Bacteria;p__Bacillota;c__Clostridia;o__Eubacteriales;f__Lachnospiraceae;g__Blautia;s__Blautia_producta
NCBI33037	0	0	0	0	0	1	0	0	0	0	0	0	0	0	0	k__Bacteria;p__Bacillota;c__Tissierellia;o__Tissierellales;f__Peptoniphilaceae;g__Anaerococcus;s__Anaerococcu

																s_vaginalis
NCBI33038	1	0	1	27	0	39	0	1	3	0	0	91	0	0	1	k__Bacteria;p__Bacillota;c__Clostridia;o__Eubacteriales;f__Lachnospiraceae;g__Mediterraneibacter;s__[Ruminococcus]_gnavus
NCBI33039	1	0	1	0	1	36	0	3	0	0	0	93	0	0	0	k__Bacteria;p__Bacillota;c__Clostridia;o__Eubacteriales;f__Lachnospiraceae;g__Mediterraneibacter;s__[Ruminococcus]_torques
NCBI33043	0	0	0	0	0	1	1	0	0	0	0	6	0	0	0	k__Bacteria;p__Bacillota;c__Clostridia;o__Eubacteriales;f__Lachnospiraceae;g__Coprococcus;s__Coprococcus_eutactus
NCBI33050	1	5	4	0	12	141	10	0	0	0	0	3	0	0	0	k__Bacteria;p__Pseudomonadota;c__Alphaproteobacteria;o__Sphingomonadales;f__Sphingomonadaceae;g__Sphingopyxis;s__Sphingopyxis_macrogoltabida
NCBI33051	10	1	0	16	34	49	2	0	0	0	0	2	0	0	1	k__Bacteria;p__Pseudomonadota;c__Alphaproteobacteria;o__Sphingomonadales;f__Sphingomonadaceae;g__Sphingomonas;s__Sphingomonas_sanguinis
NCBI33052	7	4	5	1	17	195	8	0	0	0	2	4	0	0	0	k__Bacteria;p__Pseudomonadota;c__Alphaproteobacteria;o__Sphingomonadales;f__Sphingomonadaceae;g__Sphingopyxis;s__Sphingopyxis_terrae
NCBI33069	0	0	0	0	3	1	0	0	0	0	0	0	0	0	0	k__Bacteria;p__Pseudomonadota;c__Gammaproteobacteria;o__Pseudomonadales;f__Pseudomonadaceae;g__Pseudomonas;s__Pseudomonas_viridiflava
NCBI33171	0	0	0	0	0	0	2	0	0	0	0	0	0	0	0	k__Fungi;p__Ascomycota;c__Saccharomycetes;o__Saccharomycetales;f__Saccharomycetaceae;g__Eremothecium;s__Eremothecium_ashbyi
NCBI33203	1	0	0	0	0	0	0	0	0	0	0	0	0	0	0	k__Fungi;p__Ascomycota;c__Sordariomycetes;o__Hypocreales;f__Ophiocordycipitaceae;g__Purpureocillium;s__Purpureocillium_lilacinum
NCBI332055	2	0	0	0	13	62	0	0	0	0	0	0	0	0	5	k__Bacteria;p__Pseudomonadota;c__Alphaproteobacteria;o__Sphingomonadales;f__Sphingomonadaceae;g__Sph

																ingobium;s__Sphingobium_indicum
NCBI332168	0	0	0	0	0	1	0	0	0	0	0	0	0	0	0	k__Archaea;p__Euryarchaeota;c__Halobacteria;o__Halobacteriales;f__Halococcaceae;g__Halococcus;s__Halococcus_hamelinensis
NCBI334542	0	0	0	0	0	0	0	0	0	0	3	0	0	0	0	k__Bacteria;p__Actinomycetota;c__Actinomycetes;o__Mycobacteriales;f__Nocardiaceae;g__Rhodococcus;s__Rhodococcus_qingshengii
NCBI334852	0	0	0	0	106	54	0	0	0	0	0	0	0	0	0	k__Bacteria;p__Pseudomonadota;c__Alphaproteobacteria;o__Hyphomicrobiales;f__Methylobacteriaceae;g__Methylobacterium;s__Methylobacterium_oryzae
NCBI335406	1	0	0	0	0	6	0	0	0	0	0	0	0	0	0	k__Bacteria;p__Pseudomonadota;c__Alphaproteobacteria;o__Sphingomonadales;f__Sphingosinicellaceae;g__Sphingosinicella;s__Sphingosinicella_microcystinivorans
NCBI335924	0	0	0	0	0	5	0	0	1	0	0	0	0	0	0	k__Heunggongvirae;p__Uroviricota;c__Caudoviricetes;o__;f__;g__;s__Cyanobacteria_phage_AS-1
NCBI336203	0	15	0	0	53	346	7	0	7	0	8	7	0	5	0	k__Bacteria;p__Pseudomonadota;c__Alphaproteobacteria;o__Sphingomonadales;f__Sphingomonadaceae;g__Sphingobium;s__Sphingobium_fuliginis
NCBI336820	0	0	0	0	0	0	0	1	0	0	0	0	0	0	0	k__Bacteria;p__Bacteroidota;c__Sphingobacteriia;o__Sphingobacteriales;f__Sphingobacteriaceae;g__Pedobacter;s__Pedobacter_roseus
NCBI336988	1	0	0	1	1	0	0	2	1	3	0	0	1	0	0	k__Bacteria;p__Bacillota;c__Bacilli;o__Lactobacillales;f__Lactobacillaceae;g__Oenococcus;s__Oenococcus_kitaharae
NCBI337051	0	0	0	0	2	0	0	0	0	0	0	0	0	0	0	k__Shotokuvirae;p__Cossaviricota;c__Papovaviricetes;o__Zurhausenvirales;f__Papillomaviridae;g__Betapapillomavirus;s__Betapapillomavirus_1
NCBI338584	0	0	0	1	0	0	0	0	0	0	0	0	0	0	0	k__Bacteria;p__Actinomycetota;c__Actinomycetes;o__Micromonosporales;f__Micromonosporaceae;g__Polymo

															rphospora;s__Polymorphospora_rubra
NCBI33871	0	0	0	0	0	1	0	0	0	0	0	0	0	0	0	k__Bacteria;p__Actinomycetota;c__Coriobacteriia;o__Coriobacteriales;f__Coriobacteriaceae;g__Coriobacterium;s__Coriobacterium_glomerans
NCBI33889	1	0	1	4	7	28	2	0	1	0	0	0	0	0	0	k__Bacteria;p__Actinomycetota;c__Actinomycetes;o__Micrococcales;f__Brevibacteriaceae;g__Brevibacterium;s__Brevibacterium_casei
NCBI339	0	0	0	0	11	18	0	3	0	0	1	4	19	12	12	k__Bacteria;p__Pseudomonadota;c__Gammaproteobacteria;o__Xanthomonadales;f__Xanthomonadaceae;g__Xanthomonas;s__Xanthomonas_campestris
NCBI33903	0	0	0	0	0	2	0	0	0	0	0	0	0	0	0	k__Bacteria;p__Actinomycetota;c__Actinomycetes;o__Kitasatosporales;f__Streptomycetaceae;g__Streptomyces;s__Streptomyces_avermitilis
NCBI33905	2	0	0	3	8	24	5	0	0	0	0	1	0	0	0	k__Bacteria;p__Actinomycetota;c__Actinomycetes;o__Bifidobacteriales;f__Bifidobacteriaceae;g__Bifidobacterium;s__Bifidobacterium_thermophilum
NCBI33934	4	5	1	18	10	126	6	3	0	0	2	2557	0	0	3	k__Bacteria;p__Bacillota;c__Bacilli;o__Bacillales;f__Bacillaceae;g__Anoxybacillus;s__Anoxybacillus_flavithermus
NCBI33936	0	0	0	0	0	1	0	0	0	0	0	25	0	0	0	k__Bacteria;p__Bacillota;c__Bacilli;o__Bacillales;f__Bacillaceae;g__Aeribacillus;s__Aeribacillus_pallidus
NCBI33938	0	0	0	0	0	1	4	0	0	0	0	2	0	0	0	k__Bacteria;p__Bacillota;c__Bacilli;o__Bacillales;f__Bacillaceae;g__Geobacillus;s__Geobacillus_thermocatenulatus
NCBI33940	0	0	0	0	0	16	0	0	0	0	0	4	0	0	0	k__Bacteria;p__Bacillota;c__Bacilli;o__Bacillales;f__Bacillaceae;g__Geobacillus;s__Geobacillus_thermodenitrificans
NCBI33941	0	0	0	12	0	46	0	4	0	0	1	714	0	0	0	k__Bacteria;p__Bacillota;c__Bacilli;o__Bacillales;f__Bacillaceae;g__Geobacillus;s__Geobacillus_thermoleovora

															ns
NCBI33945	0	0	2	0	0	6	0	0	1	0	0	3	0	0	0	k__Bacteria;p__Bacillota;c__Bacilli;o__Lactobacillales;f__Enterococcaceae;g__Enterococcus;s__Enterococcus_avium
NCBI33959	0	0	2	4	30	100	144	2	18	1	1	41	0	0	0	k__Bacteria;p__Bacillota;c__Bacilli;o__Lactobacillales;f__Lactobacillaceae;g__Lactobacillus;s__Lactobacillus_johnsonii
NCBI33962	99	151	133	208	199	101	156	177	155	137	108	121	23	63	42	k__Bacteria;p__Bacillota;c__Bacilli;o__Lactobacillales;f__Lactobacillaceae;g__Lentilactobacillus;s__Lentilactobacillus_kefiri
NCBI33964	27	43	36	45	47	14	49	45	48	81	63	77	61	70	47	k__Bacteria;p__Bacillota;c__Bacilli;o__Lactobacillales;f__Lactobacillaceae;g__Leuconostoc;s__Leuconostoc_citreum
NCBI33990	0	0	0	0	0	1	0	0	0	0	0	0	0	0	0	k__Bacteria;p__Pseudomonadota;c__Alphaproteobacteria;o__Rickettsiales;f__Rickettsiaceae;g__Rickettsia;s__Rickettsia_bellii
NCBI33995	127	139	187	465	397	354	211	124	248	616	559	951	4465	4396	5429	k__Bacteria;p__Pseudomonadota;c__Alphaproteobacteria;o__Rhodospirillales;f__Acetobacteraceae;g__Komagataeibacter;s__Komagataeibacter_europaeus
NCBI33996	198	180	203	489	461	441	347	189	367	1644	1440	2236	2277	2248	2585	k__Bacteria;p__Pseudomonadota;c__Alphaproteobacteria;o__Rhodospirillales;f__Acetobacteraceae;g__Gluconacetobacter;s__Gluconacetobacter_diazotrophicus
NCBI34	0	0	0	0	0	17	0	0	0	0	0	0	0	0	0	k__Bacteria;p__Myxococcota;c__Myxococcia;o__Myxococcales;f__Myxococcaceae;g__Myxococcus;s__Myxococcus_xanthus
NCBI34004	0	0	0	0	0	6	0	0	0	0	0	2	0	0	0	k__Bacteria;p__Pseudomonadota;c__Alphaproteobacteria;o__Rhodobacterales;f__Paracoccaceae;g__Paracoccus;s__Paracoccus_aminovorans
NCBI340146	0	0	0	0	0	1	0	0	0	0	0	0	0	0	0	k__Bacteria;p__Bacillota;c__Bacilli;o__Bacillales;f__;g__Exiguobacterium;s__Exiguobacterium_mexicanum

NCBI34020	283	263	224	848	1905	4101	345	60	171	47	37	59	50	49	42	k__Bacteria;p__Pseudomonadota;c__Alphaproteobacteria;o__Hyphomicrobiales;f__Rhizobiaceae;g__Liberibacter;s__Candidatus_Liberibacter_africanus
NCBI34029	0	0	0	1	1	34	0	0	0	0	0	0	0	0	0	k__Bacteria;p__Pseudomonadota;c__Betaproteobacteria;o__Burkholderiales;f__;g__Leptothrix;s__Leptothrix_cholodnii
NCBI34038	11	1	0	100	266	683	40	0	0	6	0	0	0	12	0	k__Bacteria;p__Pseudomonadota;c__Gammaproteobacteria;o__Enterobacterales;f__Yersiniaceae;g__Rahnella;s__Rahnella_aquatilis
NCBI34062	117	117	77	146	350	1611	142	26	78	24	15	65	21	18	27	k__Bacteria;p__Pseudomonadota;c__Gammaproteobacteria;o__Moraxellales;f__Moraxellaceae;g__Moraxella;s__Moraxella_osloensis
NCBI34073	0	5	0	0	14	59	6	0	2	0	0	0	11	0	1	k__Bacteria;p__Pseudomonadota;c__Betaproteobacteria;o__Burkholderiales;f__Comamonadaceae;g__Variovorax;s__Variovorax_paradoxus
NCBI34085	0	0	0	0	1	1	0	0	0	0	0	0	0	0	0	k__Bacteria;p__Bacteroidota;c__Flavobacteriia;o__Flavobacteriales;f__Weeksellaceae;g__Riemerella;s__Riemerella_anatipestifer
NCBI34103	1	1	0	0	8	137	6	0	0	0	0	1	0	0	0	k__Bacteria;p__Pseudomonadota;c__Betaproteobacteria;o__Burkholderiales;f__Sphaerotilaceae;g__Sphaerotilus;s__Sphaerotilus_natans
NCBI341045	0	0	0	0	0	1	0	0	1	0	0	0	0	0	0	k__Bacteria;p__Pseudomonadota;c__Betaproteobacteria;o__Burkholderiales;f__Oxalobacteraceae;g__Herbaspirillum;s__Herbaspirillum_hiltneri
NCBI341458	52	69	17	41	0	23	18	0	17	34	0	0	0	0	0	k__Fungi;p__Ascomycota;c__Saccharomycetes;o__Saccharomycetales;f__Saccharomycetaceae;g__;s__uncultured_Saccharomyces
NCBI342668	0	0	0	1	0	0	1	1	0	2	0	0	0	0	1	k__Fungi;p__Ascomycota;c__Leotiomycetes;o__;f__Pseudeurotiaceae;g__Pseudogymnoascus;s__Pseudogymnoascus_verrucosus

NCBI343	0	0	0	0	0	13	0	0	0	0	0	0	0	0	0	k__Bacteria;p__Pseudomonadota;c__Gammaproteobacteria;o__Xanthomonadales;f__Xanthomonadaceae;g__Xanthomonas;s__Xanthomonas_translucens
NCBI34381	0	0	0	0	0	1	0	0	0	0	0	2	0	0	0	k__Fungi;p__Ascomycota;c__Eurotiomycetes;o__Eurotiales;f__Aspergillaceae;g__Aspergillus;s__Aspergillus_japonicus
NCBI343874	24	36	21	89	186	80	65	11	21	4	5	5	23	9	7	k__Bacteria;p__Bacteroidota;c__Flavobacteriia;o__Flavobacteriales;f__Weeksellaceae;g__Empedobacter;s__Empedobacter_falsenii
NCBI347	0	0	0	0	0	0	0	0	0	0	0	14	0	0	0	k__Bacteria;p__Pseudomonadota;c__Gammaproteobacteria;o__Xanthomonadales;f__Xanthomonadaceae;g__Xanthomonas;s__Xanthomonas_oryzae
NCBI348826	3	2	0	5	8	10	0	0	3	0	1	0	0	0	0	k__Archaea;p__Euryarchaeota;c__Halobacteria;o__Natrialbales;f__Natrialbaceae;g__Natronorubrum;s__Natronorubrum_aibiense
NCBI349751	0	0	0	0	0	0	0	0	0	0	0	1	0	0	0	k__Bacteria;p__Actinomycetota;c__Actinomycetes;o__Mycobacteriales;f__Corynebacteriaceae;g__Corynebacterium;s__Corynebacterium_marinum
NCBI351091	1	0	0	0	0	0	0	0	0	0	0	0	0	0	0	k__Bacteria;p__Bacillota;c__Clostridia;o__Eubacteriales;f__Oscillospiraceae;g__Oscillibacter;s__Oscillibacter_valericigenes
NCBI35244	0	0	0	0	1	6	2	2	0	0	1	1	0	0	0	k__Heunggongvirae;p__Peploviricota;c__Herviviricetes;o__Herpesvirales;f__Herpesviridae;g__Varicellovirus;s__Bovine_alphaherpesvirus_5
NCBI352475	10	16	12	3	71	747	16	6	2	6	0	3	0	0	6	k__Bacteria;p__Pseudomonadota;c__Alphaproteobacteria;o__Hyphomicrobiales;f__Rhizobiaceae;g__Shinella;s__Shinella_zoogloeoides
NCBI352858	0	0	0	0	0	0	1	0	0	0	0	0	0	0	0	k__Bacteria;p__Bacillota;c__Bacilli;o__Bacillales;f__Bacillaceae;g__Bacillus;s__Bacillus_sp._Y1
NCBI3	0	0	0	0	2	27	0	0	1	0	0	0	0	0	0	k__Bacteria;p__Thermodesulfobacteriota;c__Desulfurom

5554																onadia;o__Geobacterales;f__Geobacteraceae;g__Geobacter;s__Geobacter_sulfurreducens
NCBI356302	0	0	0	0	0	5	0	0	0	0	0	0	0	0	0	k__Bacteria;p__Pseudomonadota;c__Betaproteobacteria;o__Burkholderiales;f__Burkholderiaceae;g__Chitinimonas;s__Chitinimonas_koreensis
NCBI356837	0	0	0	0	0	1	0	0	0	0	0	0	0	0	0	k__Bacteria;p__Pseudomonadota;c__Betaproteobacteria;o__Rhodocyclales;f__Zoogloeaceae;g__Azoarcus;s__Azoarcus_sp._DN11
NCBI35703	0	0	0	0	0	0	0	0	0	0	0	0	15	0	0	k__Bacteria;p__Pseudomonadota;c__Gammaproteobacteria;o__Enterobacterales;f__Enterobacteriaceae;g__Citrobacter;s__Citrobacter_amalonaticus
NCBI357276	0	0	0	0	1	29	5	1	0	0	0	43	0	0	0	k__Bacteria;p__Bacteroidota;c__Bacteroidia;o__Bacteroidales;f__Bacteroidaceae;g__Phocaeicola;s__Phocaeicola_dorei
NCBI35755	0	0	0	0	0	3	0	0	0	0	0	0	0	0	0	k__Bacteria;p__Actinomycetota;c__Actinomycetes;o__Mycobacteriales;f__Corynebacteriaceae;g__Corynebacterium;s__Corynebacterium_kutscheri
NCBI35760	0	0	0	0	0	0	2	0	0	0	0	0	0	0	0	k__Bacteria;p__Actinomycetota;c__Actinomycetes;o__Bifidobacteriales;f__Bifidobacteriaceae;g__Bifidobacterium;s__Bifidobacterium_choerinum
NCBI35787	0	2	0	0	1	9	0	1	0	0	0	0	0	0	0	k__Bacteria;p__Bacillota;c__Bacilli;o__Lactobacillales;f__Lactobacillaceae;g__Limosilactobacillus;s__Limosilactobacillus_pontis
NCBI358	23	34	6	4	88	842	30	5	8	0	5	20	7	4	11	k__Bacteria;p__Pseudomonadota;c__Alphaproteobacteria;o__Hyphomicrobiales;f__Rhizobiaceae;g__Agrobacterium;s__Agrobacterium_tumefaciens
NCBI358220	24	63	20	5	67	1619	12	4	11	17	0	47	35	0	0	k__Bacteria;p__Pseudomonadota;c__Betaproteobacteria;o__Burkholderiales;f__Comamonadaceae;g__Acidovorax;s__Acidovorax_sp._KKS102
NCBI3	0	0	0	0	1	0	1	0	1	0	0	1	0	0	0	k__Bacteria;p__Bacillota;c__Bacilli;o__Bacillales;f__Ba

5841																cillaceae;g__Caldibacillus;s__Caldibacillus_thermoamylovorans
NCBI359	4	7	0	2	12	66	0	0	3	0	0	5	0	0	1	k__Bacteria;p__Pseudomonadota;c__Alphaproteobacteria;o__Hyphomicrobiales;f__Rhizobiaceae;g__Agrobacterium;s__Agrobacterium_rhizogenes
NCBI359110	0	0	0	0	1	0	0	0	0	0	0	0	0	0	0	k__Bacteria;p__Pseudomonadota;c__Gammaproteobacteria;o__Pseudomonadales;f__Pseudomonadaceae;g__Pseudomonas;s__Pseudomonas_extremaustralis
NCBI36015	12	0	21	18	74	131	0	9	30	51	24	51	0	40	45	k__Fungi;p__Ascomycota;c__Saccharomycetes;o__Saccharomycetales;f__Pichiaceae;g__Pichia;s__Pichia_kluyveri
NCBI36016	0	0	0	0	0	0	0	1	0	0	0	0	0	0	0	k__Fungi;p__Ascomycota;c__Saccharomycetes;o__Saccharomycetales;f__Phaffomycetaceae;g__Cyberlindnera;s__Cyberlindnera_americana
NCBI36022	0	0	0	0	0	0	0	0	0	0	0	2	0	0	0	k__Fungi;p__Ascomycota;c__Saccharomycetes;o__Saccharomycetales;f__Phaffomycetaceae;g__Cyberlindnera;s__Cyberlindnera_fabianii
NCBI36031	0	0	18	0	0	0	0	0	0	0	0	8	0	0	0	k__Fungi;p__Ascomycota;c__Saccharomycetes;o__Saccharomycetales;f__Phaffomycetaceae;g__Wickerhamomyces;s__Wickerhamomyces_sydowiorum
NCBI36033	34	13	4	7	8	4	10	14	7	36	39	8	13	6	10	k__Fungi;p__Ascomycota;c__Saccharomycetes;o__Saccharomycetales;f__Saccharomycetaceae;g__Vanderwaltozyma;s__Vanderwaltozyma_polyspora
NCBI360911	1	0	0	0	0	0	0	0	0	0	0	0	0	0	0	k__Bacteria;p__Bacillota;c__Bacilli;o__Bacillales;f__;g__Exiguobacterium;s__Exiguobacterium_sp._AT1b
NCBI361183	0	0	0	0	0	1	0	0	0	0	0	0	0	0	0	k__Bacteria;p__Pseudomonadota;c__Alphaproteobacteria;o__Sphingomonadales;f__Erythrobacteraceae;g__Altererythrobacter;s__Altererythrobacter_epoxidivorans
NCBI363952	17	40	28	12	48	250	25	0	5	0	0	5	0	5	0	k__Bacteria;p__Pseudomonadota;c__Betaproteobacteria;o__Burkholderiales;f__Comamonadaceae;g__Comamona

																s;s__Comamonas_thiooxydans
NCBI364317	0	0	0	0	0	4	0	0	0	0	0	0	0	0	0	k__Bacteria;p__Pseudomonadota;c__Betaproteobacteria;o__Burkholderiales;f__Comamonadaceae;g__Verminephrobacter;s__Verminephrobacter_eiseniae
NCBI364410	0	0	0	0	3	10	0	0	0	0	1	0	0	0	0	k__Bacteria;p__Pseudomonadota;c__Alphaproteobacteria;o__Rhodospirillales;f__Acetobacteraceae;g__Granulibacter;s__Granulibacter_bethesdensis
NCBI36630	0	0	0	0	1	0	0	0	0	0	0	0	0	0	0	k__Fungi;p__Ascomycota;c__Eurotiomycetes;o__Eurotiales;f__Aspergillaceae;g__Aspergillus;s__Aspergillus_fischeri
NCBI36745	1	0	0	0	0	0	0	0	0	0	0	0	0	0	0	k__Bacteria;p__Bacillota;c__Clostridia;o__Eubacteriales;f__Clostridiaceae;g__Clostridium;s__Clostridium_saccharoperbutylacetonicum
NCBI36746	0	0	0	0	0	0	0	0	1	0	0	0	0	0	0	k__Bacteria;p__Pseudomonadota;c__Gammaproteobacteria;o__Pseudomonadales;f__Pseudomonadaceae;g__Pseudomonas;s__Pseudomonas_cichorii
NCBI367743	0	0	0	0	0	0	0	0	0	0	0	1	0	0	0	k__Bacteria;p__Bacillota;c__Bacilli;o__Bacillales;f__Planococcaceae;g__Ureibacillus;s__Ureibacillus_thermophilus
NCBI36805	5	0	0	3	18	80	0	0	0	5	0	7	2	0	2	k__Bacteria;p__Actinomycetota;c__Actinomycetes;o__Micrococcales;f__Microbacteriaceae;g__Microbacterium;s__Microbacterium_aurum
NCBI36809	0	0	0	9	0	2	0	0	0	0	0	29	0	0	0	k__Bacteria;p__Actinomycetota;c__Actinomycetes;o__Mycobacteriales;f__Mycobacteriaceae;g__Mycobacteroides;s__Mycobacteroides_abscessus
NCBI36814	0	0	0	1	0	0	0	0	0	0	0	0	0	0	0	k__Bacteria;p__Actinomycetota;c__Actinomycetes;o__Mycobacteriales;f__Mycobacteriaceae;g__Mycolicibacterium;s__Mycolicibacterium_rhodesiae

NCBI368607	0	0	0	0	0	2	0	0	0	0	0	0	0	0	0	k__Bacteria;p__Pseudomonadota;c__Betaproteobacteria;o__Burkholderiales;f__Oxalobacteraceae;g__Janthinobacterium;s__Janthinobacterium_svalbardensis
NCBI36863	9	15	17	3	51	543	0	0	8	4	0	0	0	0	0	k__Bacteria;p__Pseudomonadota;c__Betaproteobacteria;o__Burkholderiales;f__Sphaerotilaceae;g__Ideonella;s__Ideonella_dechloratans
NCBI36873	0	0	0	0	0	0	0	0	0	0	0	2	0	0	0	k__Bacteria;p__Pseudomonadota;c__Betaproteobacteria;o__Burkholderiales;f__Burkholderiaceae;g__Paraburkholderia;s__Paraburkholderia_xenovorans
NCBI36911	0	0	4	0	0	0	0	0	0	0	0	0	0	0	0	k__Fungi;p__Ascomycota;c__Saccharomycetes;o__Saccharomycetales;f__Metschnikowiaceae;g__Clavispora;s__Clavispora_lusitaniae
NCBI369960	0	0	0	0	0	0	0	0	0	0	0	5	0	1	0	k__Pararnavirae;p__Artverviricota;c__Revtraviricetes;o__Ortervirales;f__Retroviridae;g__Gammaretrovirus;s__Porcine_type-C_oncovirus
NCBI370622	0	0	0	0	1	0	0	0	0	0	0	0	0	1	0	k__Bacteria;p__Pseudomonadota;c__Alphaproteobacteria;o__Hyphomicrobiales;f__Aurantimonadaceae;g__Aureimonas;s__Aureimonas_altamirensis
NCBI370769	0	0	0	0	1	0	1	0	0	0	0	0	0	0	0	k__Bacteria;p__Pseudomonadota;c__Gammaproteobacteria;o__Oceanospirillales;f__Halomonadaceae;g__Halomonas;s__Halomonas_denitrificans
NCBI371142	0	0	0	0	0	0	0	0	0	0	0	0	0	0	1	k__Bacteria;p__Bacteroidota;c__Sphingobacteriia;o__Sphingobacteriales;f__Sphingobacteriaceae;g__Sphingobacterium;s__Sphingobacterium_daejeonense
NCBI371155	0	0	0	0	0	475	0	0	0	0	0	31	0	0	0	k__Bacteria;p__Pseudomonadota;c__Betaproteobacteria;o__Burkholderiales;f__Comamonadaceae;g__Acidovorax;s__Acidovorax_sp._MUL2G8
NCBI371601	0	0	17	0	32	33	97	4	11	0	0	54	0	0	0	k__Bacteria;p__Bacteroidota;c__Bacteroidia;o__Bacteroidales;f__Bacteroidaceae;g__Bacteroides;s__Bacteroides_xylanisolvens

NCBI373	1	3	7	3	6	39	2	0	1	1	0	0	0	1	1	k__Bacteria;p__Pseudomonadota;c__Alphaproteobacteria;o__Hyphomicrobiales;f__Rhizobiaceae;g__Agrobacterium;s__Agrobacterium_vitis
NCBI374606	0	6	0	0	8	126	6	0	9	0	0	0	1	1	1	k__Bacteria;p__Pseudomonadota;c__Alphaproteobacteria;o__Hyphomicrobiales;f__Phyllobacteriaceae;g__Aminobacter;s__Aminobacter_sp._MSH1
NCBI374846	0	0	0	0	0	110	0	0	0	0	0	0	0	0	0	k__Bacteria;p__Actinomycetota;c__Actinomycetes;o__Mycobacteriales;f__Nocardiaceae;g__Rhodococcus;s__Rhodococcus_sp._R04
NCBI374858	0	0	0	0	0	35	0	0	0	0	0	0	0	0	0	k__Bacteria;p__Pseudomonadota;c__Betaproteobacteria;o__Rhodocyclales;f__Zoogloeaceae;g__Zoogloea;s__Zoogloea_caeni
NCBI375	0	0	0	0	2	28	0	0	0	4	0	0	0	1	0	k__Bacteria;p__Pseudomonadota;c__Alphaproteobacteria;o__Hyphomicrobiales;f__Nitrobacteraceae;g__Bradyrhizobium;s__Bradyrhizobium_japonicum
NCBI375175	59	56	58	125	109	75	102	55	56	56	39	50	14	24	17	k__Bacteria;p__Bacillota;c__Bacilli;o__Lactobacillales;f__Lactobacillaceae;g__Loigolactobacillus;s__Loigolactobacillus_backii
NCBI375286	10	7	5	3	55	481	12	1	0	0	1	10	2	2	2	k__Bacteria;p__Pseudomonadota;c__Betaproteobacteria;o__Burkholderiales;f__Oxalobacteraceae;g__Janthinobacterium;s__Janthinobacterium_sp._Marseille
NCBI376	153	227	141	1245	1578	10857	228	121	85	1103	451	145	1024	513	2335	k__Bacteria;p__Pseudomonadota;c__Alphaproteobacteria;o__Hyphomicrobiales;f__Nitrobacteraceae;g__Bradyrhizobium;s__Bradyrhizobium_sp.
NCBI37636	0	0	0	0	3	0	0	0	0	0	0	2	0	0	0	k__Bacteria;p__Deinococcota;c__Deinococci;o__Thermales;f__Thermaceae;g__Thermus;s__Thermus_scotoductus
NCBI376805	0	0	0	0	0	0	0	0	0	0	0	1	0	0	0	k__Bacteria;p__Bacteroidota;c__Bacteroidia;o__Bacteroidales;f__Bacteroidaceae;g__Phocaeicola;s__Phocaeicola_salanitronis

NCBI37727	0	0	0	0	5	7	2	0	0	0	0	0	0	0	0	k__Fungi;p__Ascomycota;c__Eurotiomycetes;o__Eurotiales;f__Trichocomaceae;g__Talaromyces;s__Talaromyces_marneffei
NCBI37734	40	85	52	291	474	325	127	14	40	4	2	16	25	16	18	k__Bacteria;p__Bacillota;c__Bacilli;o__Lactobacillales;f__Enterococcaceae;g__Enterococcus;s__Enterococcus_casseliflavus
NCBI377615	0	0	1	0	0	0	0	0	0	0	0	0	0	0	0	k__Bacteria;p__Bacillota;c__Bacilli;o__Bacillales;f__Paenibacillaceae;g__Thermobacillus;s__Thermobacillus_composti
NCBI378177	0	0	0	0	0	8	0	0	0	0	0	0	0	0	0	k__Bacteria;p__Pseudomonadota;c__Alphaproteobacteria;o__Hyphomicrobiales;f__Xanthobacteraceae;g__Azorhizobium;s__uncultured_Azorhizobium_sp.
NCBI37923	1	0	0	0	0	30	0	0	0	0	1	0	0	0	0	k__Bacteria;p__Actinomycetota;c__Actinomycetes;o__Micrococcales;f__Micrococcaceae;g__Rothia;s__Rothia_kristinae
NCBI37928	0	0	0	0	0	13	0	0	0	0	0	0	0	0	0	k__Bacteria;p__Actinomycetota;c__Actinomycetes;o__Micrococcales;f__Micrococcaceae;g__Arthrobacter;s__Arthrobacter_crystallopoietes
NCBI37929	0	0	0	0	0	0	1	0	0	0	0	0	0	0	0	k__Bacteria;p__Actinomycetota;c__Actinomycetes;o__Micrococcales;f__Micrococcaceae;g__Glutamicibacter;s__Glutamicibacter_nicotianae
NCBI379684	1809	1775	999	177	4677	68789	2022	157	719	356	279	994	258	208	343	k__Bacteria;p__Pseudomonadota;c__Alphaproteobacteria;o__Hyphomicrobiales;f__Rhizobiaceae;g__Rhizobium;s__Rhizobium_pseudoryzae
NCBI379895	0	0	0	0	0	10	0	0	0	0	0	1	0	0	0	k__Bacteria;p__Pseudomonadota;c__Betaproteobacteria;o__Burkholderiales;f__Comamonadaceae;g__Comamonas;s__Comamonas_odontotermitis
NCBI380	5	0	0	0	44	130	0	0	3	0	0	0	0	0	0	k__Bacteria;p__Pseudomonadota;c__Alphaproteobacteria;o__Hyphomicrobiales;f__Rhizobiaceae;g__Sinorhizobium;s__Sinorhizobium_fredii

NCBI380021	0	0	0	0	9	23	0	0	0	0	0	4	0	0	0	k__Bacteria;p__Pseudomonadota;c__Gammaproteobacteria;o__Pseudomonadales;f__Pseudomonadaceae;g__Pseudomonas;s__Pseudomonas_protegens
NCBI38018	823	814	844	1058	975	296	1294	1166	1180	1173	1055	1353	505	453	410	k__Viruses;p__;c__;o__;f__;g__;s__Bacteriophage_sp.
NCBI38033	0	0	0	1	0	2	0	0	0	0	0	1	0	0	0	k__Fungi;p__Ascomycota;c__Sordariomycetes;o__Sordariales;f__Chaetomiaceae;g__Chaetomium;s__Chaetomium_globosum
NCBI381630	0	0	11	0	0	0	0	0	0	0	0	0	0	0	0	k__Bacteria;p__Pseudomonadota;c__Alphaproteobacteria;o__Hyphomicrobiales;f__Methylobacteriaceae;g__Methylobacterium;s__Methylobacterium_jeotgali
NCBI382	1	6	1	1	3	100	0	0	0	1	0	1	0	0	0	k__Bacteria;p__Pseudomonadota;c__Alphaproteobacteria;o__Hyphomicrobiales;f__Rhizobiaceae;g__Sinorhizobium;s__Sinorhizobium_meliloti
NCBI38288	0	0	0	0	1	2	0	0	0	0	0	0	0	0	0	k__Bacteria;p__Actinomycetota;c__Actinomycetes;o__Mycobacteriales;f__Corynebacteriaceae;g__Corynebacterium;s__Corynebacterium_genitalium
NCBI38289	0	0	0	0	12	12	0	0	0	0	0	0	0	0	0	k__Bacteria;p__Actinomycetota;c__Actinomycetes;o__Mycobacteriales;f__Corynebacteriaceae;g__Corynebacterium;s__Corynebacterium_jeikeium
NCBI38290	0	0	0	0	0	5	0	0	0	0	0	1	0	0	0	k__Bacteria;p__Actinomycetota;c__Actinomycetes;o__Mycobacteriales;f__Corynebacteriaceae;g__Corynebacterium;s__Corynebacterium_macginleyi
NCBI38293	0	0	0	0	0	1	0	0	0	0	0	1	0	0	0	k__Bacteria;p__Pseudomonadota;c__Gammaproteobacteria;o__Vibrionales;f__Vibrionaceae;g__Photobacterium;s__Photobacterium_damselae
NCBI38301	0	0	0	1	0	0	0	1	0	0	0	0	0	0	0	k__Bacteria;p__Actinomycetota;c__Actinomycetes;o__Mycobacteriales;f__Corynebacteriaceae;g__Corynebacterium;s__Corynebacterium_minutissimum
NCBI3	0	0	0	0	0	0	0	0	0	0	0	1	0	0	0	k__Bacteria;p__Actinomycetota;c__Actinomycetes;o__

8302																Mycobacteriales;f__Corynebacteriaceae;g__Corynebacterium;s__Corynebacterium_mycetoides
NCBI38304	7	7	12	252	150	447	1	14	1	0	15	29	3	1	0	k__Bacteria;p__Actinomycetota;c__Actinomycetes;o__Mycobacteriales;f__Corynebacteriaceae;g__Corynebacterium;s__Corynebacterium_tuberculostearicum
NCBI38305	0	0	0	0	0	4	0	0	0	0	0	0	0	0	0	k__Bacteria;p__Actinomycetota;c__Actinomycetes;o__Mycobacteriales;f__Corynebacteriaceae;g__Corynebacterium;s__Corynebacterium_vitaeruminis
NCBI38308	1	0	0	5	9	7	7	0	0	36	8	28	29	27	21	k__Bacteria;p__Pseudomonadota;c__Alphaproteobacteria;o__Rhodospirillales;f__Acetobacteraceae;g__Gluconobacter;s__Gluconobacter_frateurii
NCBI38313	0	0	0	0	0	0	1	0	0	0	0	7	0	0	0	k__Bacteria;p__Pseudomonadota;c__Gammaproteobacteria;o__Alteromonadales;f__Shewanellaceae;g__Shewanella;s__Shewanella_algae
NCBI384	0	1	0	2	9	32	3	0	6	0	0	0	0	0	0	k__Bacteria;p__Pseudomonadota;c__Alphaproteobacteria;o__Hyphomicrobiales;f__Rhizobiaceae;g__Rhizobium;s__Rhizobium_leguminosarum
NCBI384636	0	0	3	4	4	3	2	0	0	1	0	0	0	0	0	k__Bacteria;p__Bacillota;c__Clostridia;o__Eubacteriales;f__Clostridiaceae;g__Hungatella;s__Hungatella_xylanolytica
NCBI387090	0	0	2	0	0	2	0	0	0	0	0	5	0	0	0	k__Bacteria;p__Bacteroidota;c__Bacteroidia;o__Bacteroidales;f__Bacteroidaceae;g__Phocaeicola;s__Phocaeicola_coprophilus
NCBI387661	2	0	0	0	0	0	0	0	0	0	0	1	0	0	0	k__Bacteria;p__Bacteroidota;c__Bacteroidia;o__Bacteroidales;f__Tannerellaceae;g__Parabacteroides;s__Parabacteroides_johnsonii
NCBI388259	0	0	0	0	1	0	9	0	0	0	0	1	0	0	0	k__Archaea;p__Euryarchaeota;c__Halobacteria;o__Natrialbales;f__Natrialbaceae;g__Natronorubrum;s__Natronorubrum_sulfidifaciens

NCBI388357	0	0	0	3	0	1	0	0	0	0	0	1	0	0	0	k__Bacteria;p__Actinomycetota;c__Actinomycetes;o__Micrococcales;f__Micrococcaceae;g__Kocuria;s__Kocuria_turfanensis
NCBI390919	0	0	0	0	1	0	0	0	0	0	0	0	0	0	0	k__Bacteria;p__Pseudomonadota;c__Gammaproteobacteria;o__Oceanospirillales;f__Halomonadaceae;g__Halomonas;s__Halomonas_olivaria
NCBI391290	0	0	0	0	0	0	0	0	0	0	0	17	0	0	0	k__Bacteria;p__Bacillota;c__Bacilli;o__Bacillales;f__Bacillaceae;g__Geobacillus;s__Geobacillus_sp._E263
NCBI391953	77	67	30	0	139	3134	55	0	38	9	13	32	0	9	14	k__Bacteria;p__Pseudomonadota;c__Betaproteobacteria;o__Burkholderiales;f__Sphaerotilaceae;g__Aquincola;s__Aquincola_tertiaricarbonis
NCBI392593	4	1	0	0	2	57	2	0	0	0	0	0	0	1	1	k__Bacteria;p__Pseudomonadota;c__Betaproteobacteria;o__Burkholderiales;f__Sphaerotilaceae;g__Inhella;s__Inhella_inkyongensis
NCBI392610	0	4	6	9	4	43	0	0	0	2	2	3	0	0	1	k__Bacteria;p__Pseudomonadota;c__Alphaproteobacteria;o__Sphingomonadales;f__Sphingomonadaceae;g__Sphingorhabdus;s__Sphingorhabdus_lacus
NCBI393283	1	0	0	0	0	0	0	0	0	0	0	0	0	0	0	k__Fungi;p__Ascomycota;c__Sordariomycetes;o__Xylariales;f__Sporocadaceae;g__Pestalotiopsis;s__Pestalotiopsis_fici
NCBI39483	0	0	0	0	0	1	0	0	0	0	0	0	0	0	0	k__Bacteria;p__Bacillota;c__Erysipelotrichia;o__Erysipelotrichales;f__Erysipelotrichaceae;g__Faecalitalea;s__Faecalitalea_cylindroides
NCBI39485	0	0	0	0	0	5	1	0	0	0	0	8	0	0	0	k__Bacteria;p__Bacillota;c__Clostridia;o__Eubacteriales;f__Lachnospiraceae;g__Lachnospira;s__Lachnospira_eligens
NCBI39488	0	0	0	0	2	48	4	3	1	0	1	43	0	1	0	k__Bacteria;p__Bacillota;c__Clostridia;o__Eubacteriales;f__Lachnospiraceae;g__Anaerobutyricum;s__Anaerobutyricum_hallii
NCBI3	0	0	8	12	0	332	16	23	0	0	0	196	5	0	0	k__Bacteria;p__Bacillota;c__Clostridia;o__Eubacteriales

9491															;f__Lachnospiraceae;g__;s__[Eubacterium]_rectale
NCBI39492	1	0	0	0	0	22	0	0	0	0	0	3	0	0	0	k__Bacteria;p__Bacillota;c__Clostridia;o__Eubacteriales;f__Oscillospiraceae;g__;s__[Eubacterium]_siraeum
NCBI395598	0	0	0	1	0	0	0	0	0	0	0	0	0	0	0	k__Bacteria;p__Pseudomonadota;c__Gammaproteobacteria;o__Pseudomonadales;f__Pseudomonadaceae;g__Pseudomonas;s__Pseudomonas_reinekei
NCBI396	0	0	0	0	0	5	0	0	0	0	7	0	0	0	0	k__Bacteria;p__Pseudomonadota;c__Alphaproteobacteria;o__Hyphomicrobiales;f__Rhizobiaceae;g__Rhizobium;s__Rhizobium_phaseoli
NCBI396015	0	0	0	0	0	72	0	0	0	0	0	3	0	0	0	k__Bacteria;p__Actinomycetota;c__Actinomycetes;o__Micrococcales;f__Micrococcaceae;g__Rothia;s__Rothia_terrae
NCBI396024	0	0	0	1	3	3	0	0	0	0	0	0	0	0	0	k__Fungi;p__Ascomycota;c__Eurotiomycetes;o__Eurotiales;f__Aspergillaceae;g__Aspergillus;s__Aspergillus_ruber
NCBI39687	0	0	0	0	18	0	0	0	0	0	0	0	0	0	0	k__Bacteria;p__Actinomycetota;c__Actinomycetes;o__Mycobacteriales;f__Mycobacteriaceae;g__Mycolicibacterium;s__Mycolicibacterium_austroafricanum
NCBI39689	0	0	0	0	18	0	0	0	0	0	0	0	0	0	0	k__Bacteria;p__Actinomycetota;c__Actinomycetes;o__Mycobacteriales;f__Mycobacteriaceae;g__Mycobacterium;s__Mycobacterium_gallinarum
NCBI39695	0	0	0	0	14	0	0	0	0	0	0	0	0	0	0	k__Bacteria;p__Actinomycetota;c__Actinomycetes;o__Mycobacteriales;f__Mycobacteriaceae;g__Mycolicibacterium;s__Mycolicibacterium_tokaiense
NCBI397260	3	8	0	0	10	97	2	0	0	0	0	1	1	0	0	k__Bacteria;p__Pseudomonadota;c__Alphaproteobacteria;o__Sphingomonadales;f__Sphingomonadaceae;g__Sphingomonas;s__Sphingomonas_sanxanigenens
NCBI39777	0	0	0	7	0	1	0	0	0	0	0	5	0	0	0	k__Bacteria;p__Bacillota;c__Negativicutes;o__Veillonellales;f__Veillonellaceae;g__Veillonella;s__Veillonella_atypica

NCBI39778	0	0	0	0	0	0	0	0	0	0	0	3	0	0	0	k__Bacteria;p__Bacillota;c__Negativicutes;o__Veillonellales;f__Veillonellaceae;g__Veillonella;s__Veillonella_dispar
NCBI397865	0	0	0	0	0	0	0	0	0	0	0	1	0	0	0	k__Bacteria;p__Bacteroidota;c__Bacteroidia;o__Bacteroidales;f__Barnesiellaceae;g__Barnesiella;s__Barnesiella_viscericola
NCBI39791	1	0	0	4	0	8	0	0	0	0	0	3	0	0	0	k__Bacteria;p__Actinomycetota;c__Actinomycetes;o__Mycobacteriales;f__Corynebacteriaceae;g__Corynebacterium;s__Corynebacterium_glucuronolyticum
NCBI398	0	0	0	0	0	1	0	0	0	0	0	0	0	0	1	k__Bacteria;p__Pseudomonadota;c__Alphaproteobacteria;o__Hyphomicrobiales;f__Rhizobiaceae;g__Rhizobium;s__Rhizobium_tropici
NCBI399	0	0	0	0	0	16	0	0	0	0	0	0	0	0	0	k__Bacteria;p__Pseudomonadota;c__Alphaproteobacteria;o__Hyphomicrobiales;f__Rhizobiaceae;g__Neorhizobium;s__Neorhizobium_galegae
NCBI399370	839	925	862	954	1313	2738	1036	1077	1220	1360	1126	1241	747	635	726	k__Bacteria;p__Bacillota;c__Bacilli;o__Lactobacillales;f__Lactobacillaceae;g__Liquorilactobacillus;s__Liquorilactobacillus_ghanensis
NCBI399497	0	0	0	0	0	12	0	0	0	0	0	0	0	0	0	k__Bacteria;p__Actinomycetota;c__Actinomycetes;o__Propionibacteriales;f__Propionibacteriaceae;g__Tessaracoccus;s__Tessaracoccus_flavescens
NCBI39950	0	0	0	0	0	0	0	1	0	0	0	0	0	0	0	k__Bacteria;p__Bacillota;c__Negativicutes;o__Veillonellales;f__Veillonellaceae;g__Dialister;s__Dialister_pneumosintes
NCBI39956	0	3	4	36	40	24	4	0	1	0	0	4	0	0	2	k__Bacteria;p__Pseudomonadota;c__Alphaproteobacteria;o__Hyphomicrobiales;f__Methylobacteriaceae;g__Methylobacterium;s__Methylobacterium_mesophilicum
NCBI39960	0	0	0	0	0	2	0	0	0	0	0	0	0	0	0	k__Bacteria;p__Pseudomonadota;c__Alphaproteobacteria;o__Sphingomonadales;f__Erythrobacteraceae;g__Erythrobacter;s__Erythrobacter_litoralis

NCBI399736	0	0	0	0	0	2	0	0	0	0	0	0	1	0	0	k__Bacteria;p__Actinomycetota;c__Actinomycetes;o__Micrococcales;f__Microbacteriaceae;g__Agrococcus;s__Agrococcus_jejuensis
NCBI399742	0	0	0	0	0	0	6	0	0	0	0	0	0	0	0	k__Bacteria;p__Pseudomonadota;c__Gammaproteobacteria;o__Enterobacterales;f__Enterobacteriaceae;g__Enterobacter;s__Enterobacter_sp._638
NCBI40127	0	0	0	0	0	1	0	0	0	0	0	0	0	0	0	k__Fungi;p__Ascomycota;c__Sordariomycetes;o__Sordariales;f__Sordariaceae;g__Neurospora;s__Neurospora_tetrasperma
NCBI40137	0	0	0	0	0	0	0	0	0	0	0	0	86	0	0	k__Bacteria;p__Pseudomonadota;c__Alphaproteobacteria;o__Hyphomicrobiales;f__Nitrobacteraceae;g__Afipia;s__Afipia_carboxidovorans
NCBI401472	0	1	0	7	1	37	1	0	2	1	0	1	2	0	0	k__Bacteria;p__Actinomycetota;c__Actinomycetes;o__Mycobacteriales;f__Corynebacteriaceae;g__Corynebacterium;s__Corynebacterium_ureicelerivorans
NCBI40214	54	40	141	140	168	299	39	5	20	4	0	109	2	2	13	k__Bacteria;p__Pseudomonadota;c__Gammaproteobacteria;o__Moraxellales;f__Moraxellaceae;g__Acinetobacter;s__Acinetobacter_johnsonii
NCBI40215	1658	1851	1084	105	3424	15058	2309	155	835	358	428	890	539	431	493	k__Bacteria;p__Pseudomonadota;c__Gammaproteobacteria;o__Moraxellales;f__Moraxellaceae;g__Acinetobacter;s__Acinetobacter_junii
NCBI40216	3	4	0	0	8	18	0	0	0	0	0	0	0	1	4	k__Bacteria;p__Pseudomonadota;c__Gammaproteobacteria;o__Moraxellales;f__Moraxellaceae;g__Acinetobacter;s__Acinetobacter_radioresistens
NCBI402297	0	0	2	0	0	0	0	0	0	0	0	0	0	0	0	k__Bacteria;p__Actinomycetota;c__Actinomycetes;o__Propionibacteriales;f__Nocardioidaceae;g__Nocardioides;s__Nocardioides_daphniae
NCBI40302	0	0	0	0	1	1	0	0	0	0	0	0	0	0	0	k__Fungi;p__Microsporidia;c__;o__Apansporoblastina;f__Nosematidae;g__Nosema;s__Nosema_ceranae
NCBI4	0	0	0	0	0	0	0	0	0	0	0	1	0	0	0	k__Bacteria;p__Actinomycetota;c__Actinomycetes;o__K

0318																itasatosporales;f__Streptomycetaceae;g__Streptomyces;s__Streptomyces_nodosus
NCBI40324	1224	1280	1105	6159	14325	20892	2453	345	1051	153	116	385	320	213	315	k__Bacteria;p__Pseudomonadota;c__Gammaproteobacteria;o__Xanthomonadales;f__Xanthomonadaceae;g__Stenotrophomonas;s__Stenotrophomonas_maltophilia
NCBI404386	0	0	0	0	0	1	0	0	0	0	0	0	0	0	0	k__Bacteria;p__Actinomycetota;c__Actinomycetes;o__Streptosporangiales;f__Streptosporangiaceae;g__Nonomuraea;s__Nonomuraea_coxensis
NCBI404582	0	0	0	0	3	0	0	0	0	0	0	0	0	0	0	k__Bacteria;p__Actinomycetota;c__Actinomycetes;o__Micrococcales;f__Micrococcaceae;g__Micrococcus;s__Micrococcus_sp._V7
NCBI40520	0	0	0	0	0	16	0	0	0	0	0	28	0	0	0	k__Bacteria;p__Bacillota;c__Clostridia;o__Eubacteriales;f__Lachnospiraceae;g__Blautia;s__Blautia_obeum
NCBI405212	0	4	0	0	5	0	0	0	1	0	0	0	0	0	0	k__Bacteria;p__Bacillota;c__Bacilli;o__Bacillales;f__Alicyclobacillaceae;g__Alicyclobacillus;s__Alicyclobacillus_acidocaldarius
NCBI40545	0	0	0	1	0	0	0	0	0	0	0	1	0	0	0	k__Bacteria;p__Pseudomonadota;c__Betaproteobacteria;o__Burkholderiales;f__Sutterellaceae;g__Sutterella;s__Sutterella_wadsworthensis
NCBI40559	14	7	4	43	79	71	18	8	12	1	1	0	5	1	0	k__Fungi;p__Ascomycota;c__Leotiomycetes;o__Helotiales;f__Sclerotiniaceae;g__Botrytis;s__Botrytis_cinerea
NCBI407020	0	2	0	0	0	7	0	0	0	0	0	0	0	0	0	k__Bacteria;p__Pseudomonadota;c__Alphaproteobacteria;o__Sphingomonadales;f__Sphingomonadaceae;g__Sphingobium;s__Sphingobium_sp._MI1205
NCBI407035	0	0	0	1	0	1	0	0	0	0	0	0	0	0	0	k__Bacteria;p__Bacillota;c__Bacilli;o__Bacillales;f__Staphylococcaceae;g__Salinicoccus;s__Salinicoccus_halodurans
NCBI408	0	2	4	8	26	61	3	0	5	0	0	0	0	0	0	k__Bacteria;p__Pseudomonadota;c__Alphaproteobacteria;o__Hyphomicrobiales;f__Methylobacteriaceae;g__Methylorubrum;s__Methylorubrum_extorquens

NCBI40872	0	0	0	0	0	2	0	0	0	0	0	0	0	0	0	k__Bacteria;p__Pseudomonadota;c__Alphaproteobacteria;o__;f__;g__;s__unidentified_eubacterium_clone_ESH212C
NCBI40988	0	1	0	0	0	0	0	0	0	0	1	0	0	0	0	k__Bacteria;p__Actinomycetota;c__Actinomycetes;o__Pseudonocardiales;f__Pseudonocardiaceae;g__Saccharomonospora;s__Saccharomonospora_azurea
NCBI40990	0	0	0	7	0	7	0	0	0	0	0	5	0	0	0	k__Bacteria;p__Actinomycetota;c__Actinomycetes;o__Pseudonocardiales;f__Pseudonocardiaceae;g__Saccharomonospora;s__Saccharomonospora_glauca
NCBI41	0	0	0	0	0	61	0	0	0	0	0	0	0	0	0	k__Bacteria;p__Myxococcota;c__Myxococcia;o__Myxococcales;f__Archangiaceae;g__Stigmatella;s__Stigmatella_aurantiaca
NCBI410072	0	0	0	0	0	13	1	0	0	0	0	14	0	0	0	k__Bacteria;p__Bacillota;c__Clostridia;o__Eubacteriales;f__Lachnospiraceae;g__Coprococcus;s__Coprococcus_comes
NCBI41058	0	0	0	0	0	5	0	0	0	0	0	0	0	0	0	k__Fungi;p__Ascomycota;c__Eurotiomycetes;o__Eurotiales;f__Aspergillaceae;g__Aspergillus;s__Aspergillus_sojae
NCBI41061	0	0	0	0	0	6	0	0	0	0	0	0	0	0	0	k__Fungi;p__Ascomycota;c__Eurotiomycetes;o__Eurotiales;f__Aspergillaceae;g__Aspergillus;s__Aspergillus_nomiae
NCBI41063	1	2	1	0	2	0	0	0	1	0	0	0	0	1	0	k__Fungi;p__Ascomycota;c__Eurotiomycetes;o__Eurotiales;f__Aspergillaceae;g__Penicilliopsis;s__Penicilliopsis_zonata
NCBI411486	0	0	0	0	0	31	0	0	0	0	0	32	0	0	0	k__Bacteria;p__Bacillota;c__Clostridia;o__Eubacteriales;f__Clostridiaceae;g__Clostridium;s__Clostridium_sp._M62/1
NCBI41170	2	0	0	4	14	4	0	0	0	0	0	1	0	0	0	k__Bacteria;p__Bacillota;c__Bacilli;o__Bacillales;f__;g__Exiguobacterium;s__Exiguobacterium_acetylicum
NCBI4	0	0	1	0	0	1	0	0	0	0	13	1	0	0	0	k__Bacteria;p__Pseudomonadota;c__Gammaproteobacte

1202															ria;o__Enterobacterales;f__Yersiniaceae;g__Ewingella;s__Ewingella_americana
NCBI412384	0	0	0	0	0	13	0	0	0	0	0	10	0	0	0	k__Bacteria;p__Bacillota;c__Bacilli;o__Bacillales;f__Bacillaceae;g__Priestia;s__Priestia_aryabhattai
NCBI41276	10	56	9	0	25	838	8	0	0	9	12	0	10	0	4	k__Bacteria;p__Pseudomonadota;c__Alphaproteobacteria;o__Caulobacterales;f__Caulobacteraceae;g__Brevundimonas;s__Brevundimonas_vesicularis
NCBI413882	0	1	0	0	0	9	0	0	0	0	0	0	0	0	0	k__Bacteria;p__Pseudomonadota;c__Betaproteobacteria;o__Burkholderiales;f__Sphaerotilaceae;g__Caldimonas;s__Caldimonas_brevitalea
NCBI41413	0	0	0	1	0	7	0	0	0	0	0	3	0	0	0	k__Fungi;p__Ascomycota;c__Eurotiomycetes;o__Eurotiales;f__Aspergillaceae;g__Aspergillus;s__Aspergillus_glaucus
NCBI415229	0	0	0	0	0	1	0	0	0	0	0	0	0	0	0	k__Bacteria;p__Pseudomonadota;c__Gammaproteobacteria;o__Xanthomonadales;f__Xanthomonadaceae;g__Pseudoxanthomonas;s__Pseudoxanthomonas_spadix
NCBI417367	0	0	0	0	0	0	0	0	0	0	1	0	0	0	0	k__Bacteria;p__Bacillota;c__Bacilli;o__Bacillales;f__Planococcaceae;g__Paenisporosarcina;s__Paenisporosarcina_antarctica
NCBI418223	3	2	0	38	27	31	0	0	0	2	0	0	0	0	0	k__Bacteria;p__Pseudomonadota;c__Alphaproteobacteria;o__Hyphomicrobiales;f__Methylobacteriaceae;g__Methylobacterium;s__Methylobacterium_phyllosphaerae
NCBI419475	0	0	2	1	1	1	0	0	0	0	0	0	0	0	0	k__Bacteria;p__Pseudomonadota;c__Alphaproteobacteria;o__Hyphomicrobiales;f__Brucellaceae;g__Brucella;s__Brucella_pseudogrignonensis
NCBI419476	0	0	0	0	0	0	0	0	0	0	0	4	0	0	0	k__Bacteria;p__Actinomycetota;c__Actinomycetes;o__Propionibacteriales;f__Nocardioidaceae;g__Nocardioides;s__Nocardioides_marinisabuli
NCBI41977	0	0	0	0	0	4	0	0	0	0	0	0	0	0	0	k__Bacteria;p__Pseudomonadota;c__Betaproteobacteria;o__Rhodocyclales;f__Zoogloeaceae;g__Parazoarcus;s__

															Parazoarcus_communis
NCBI41997	0	0	0	0	4	0	0	0	0	0	0	0	0	0	0	k__Bacteria;p__Bacillota;c__Bacilli;o__Lactobacillales;f__Enterococcaceae;g__Enterococcus;s__Enterococcus_saccharolyticus
NCBI421525	0	0	0	20	4	0	0	0	0	0	0	0	0	0	0	k__Bacteria;p__Bacteroidota;c__Flavobacteriia;o__Flavobacteriales;f__Weeksellaceae;g__Kaistella;s__Kaistella_haifensis
NCBI42197	0	0	1	0	0	65	0	0	4	0	0	130	0	0	0	k__Bacteria;p__Actinomycetota;c__Actinomycetes;o__Pseudonocardiales;f__Pseudonocardiaceae;g__Actinosynnema;s__Actinosynnema_pretiosum
NCBI42256	0	1	0	0	0	0	0	0	0	0	0	0	0	0	0	k__Bacteria;p__Actinomycetota;c__Rubrobacteria;o__Rubrobacterales;f__Rubrobacteraceae;g__Rubrobacter;s__Rubrobacter_radiotolerans
NCBI42444	0	0	0	0	0	5	0	0	0	0	0	0	0	0	0	k__Bacteria;p__Pseudomonadota;c__Alphaproteobacteria;o__Rhodobacterales;f__Roseobacteraceae;g__Marinovum;s__Marinovum_algicola
NCBI424757	0	0	0	0	0	0	0	0	0	0	0	1	0	0	0	k__Bacteria;p__Pseudomonadota;c__Alphaproteobacteria;o__Hyphomicrobiales;f__Aurantimonadaceae;g__Aureimonas;s__Aureimonas_frigidaquae
NCBI424800	1	0	0	2	2	4	3	0	0	0	1	0	0	0	0	k__Bacteria;p__Pseudomonadota;c__Alphaproteobacteria;o__Sphingomonadales;f__Sphingomonadaceae;g__Sphingomonas;s__Sphingomonas_insulae
NCBI425941	0	0	0	0	0	0	0	0	0	0	0	17	0	0	0	k__Bacteria;p__Bacteroidota;c__Bacteroidia;o__Bacteroidales;f__Prevotellaceae;g__Hoylesella;s__Hoylesella_nanceiensis
NCBI426114	0	0	0	0	0	1	0	0	0	0	0	0	0	0	0	k__Bacteria;p__Pseudomonadota;c__Betaproteobacteria;o__Burkholderiales;f__;g__Thiomonas;s__Thiomonas_arsenitoxydans
NCBI42817	0	0	0	7	3	1	0	0	0	0	0	1	0	0	0	k__Bacteria;p__Actinomycetota;c__Actinomycetes;o__Mycobacteriales;f__Corynebacteriaceae;g__Corynebacte

																rium;s__Corynebacterium_argentoratense
NCBI428712	0	0	1	0	0	0	0	1	0	0	0	0	0	0	0	k__Bacteria;p__Synergistota;c__Synergistia;o__Synergistales;f__Dethiosulfovibrionaceae;g__Jonquetella;s__Jonquetella_anthropi
NCBI43	0	0	0	0	0	41	0	0	0	0	0	0	0	0	0	k__Bacteria;p__Myxococcota;c__Myxococcia;o__Myxococcales;f__Archangiaceae;g__Cystobacter;s__Cystobacter_fuscus
NCBI431059	0	0	0	0	0	206	0	0	0	0	0	0	0	0	0	k__Bacteria;p__Pseudomonadota;c__Betaproteobacteria;o__Burkholderiales;f__Comamonadaceae;g__Pelomonas;s__Pelomonas_puraquae
NCBI431306	1652	1475	1687	5663	5467	4949	3299	2160	3287	14268	13282	20336	17701	18558	21401	k__Bacteria;p__Pseudomonadota;c__Alphaproteobacteria;o__Rhodospirillales;f__Acetobacteraceae;g__Acetobacter;s__Acetobacter_ghanensis
NCBI43263	160	306	58	12	294	2358	149	16	56	33	21	97	45	28	25	k__Bacteria;p__Pseudomonadota;c__Gammaproteobacteria;o__Pseudomonadales;f__Pseudomonadaceae;g__Pseudomonas;s__Pseudomonas_alcaligenes
NCBI434010	4	2	3	1	11	208	3	0	3	0	0	2	0	0	0	k__Bacteria;p__Pseudomonadota;c__Betaproteobacteria;o__Burkholderiales;f__Comamonadaceae;g__Hydrogenophaga;s__Hydrogenophaga_sp._PBL-H3
NCBI43435	1038	1189	1007	3322	2675	2203	1531	1003	1554	5511	5103	7583	24825	24408	30134	k__Bacteria;p__Pseudomonadota;c__Alphaproteobacteria;o__Rhodospirillales;f__Acetobacteraceae;g__Acetobacter;s__Acetobacter_aceti
NCBI436	194	136	146	511	450	364	233	186	257	760	734	1014	4964	4839	5665	k__Bacteria;p__Pseudomonadota;c__Alphaproteobacteria;o__Rhodospirillales;f__Acetobacteraceae;g__Novacetimonas;s__Novacetimonas_hansenii
NCBI436515	0	0	0	0	0	6	0	0	0	0	0	0	0	0	0	k__Bacteria;p__Pseudomonadota;c__Betaproteobacteria;o__Burkholderiales;f__Comamonadaceae;g__Variovorax;s__Variovorax_boronicumulans
NCBI43669	0	0	0	0	0	4	0	0	0	0	0	0	0	0	0	k__Bacteria;p__Actinomycetota;c__Actinomycetes;o__Micrococcales;f__Dermabacteraceae;g__Brachybacteriu

																m;s__Brachybacterium_faecium
NCBI43675	0	0	2	2	4	21	2	0	2	0	3	293	0	0	0	k__Bacteria;p__Actinomycetota;c__Actinomycetes;o__Micrococcales;f__Micrococcaceae;g__Rothia;s__Rothia_mucilaginosa
NCBI43768	1	0	0	1	2	7	0	0	0	0	0	7	0	0	0	k__Bacteria;p__Actinomycetota;c__Actinomycetes;o__Mycobacteriales;f__Corynebacteriaceae;g__Corynebacterium;s__Corynebacterium_matruchotii
NCBI43769	1	0	0	1	0	0	0	0	0	0	0	4	0	0	0	k__Bacteria;p__Actinomycetota;c__Actinomycetes;o__Mycobacteriales;f__Corynebacteriaceae;g__Corynebacterium;s__Corynebacterium_propinquum
NCBI43770	0	0	0	0	0	246	0	0	0	0	0	0	0	0	0	k__Bacteria;p__Actinomycetota;c__Actinomycetes;o__Mycobacteriales;f__Corynebacteriaceae;g__Corynebacterium;s__Corynebacterium_striatum
NCBI43771	0	0	0	0	0	13	0	0	0	0	0	1	0	0	0	k__Bacteria;p__Actinomycetota;c__Actinomycetes;o__Mycobacteriales;f__Corynebacteriaceae;g__Corynebacterium;s__Corynebacterium_urealyticum
NCBI437897	0	0	0	0	0	13	0	0	0	0	0	12	0	0	0	k__Bacteria;p__Bacillota;c__Negativicutes;o__Selenomonadales;f__Selenomonadaceae;g__Megamonas;s__Megamonas_funiformis
NCBI438	4881	5051	5676	14821	11865	11188	8165	5184	7020	24827	23427	33686	140967	137484	170568	k__Bacteria;p__Pseudomonadota;c__Alphaproteobacteria;o__Rhodospirillales;f__Acetobacteraceae;g__Acetobacter;s__Acetobacter_pasteurianus
NCBI43948	0	0	0	0	0	24	0	0	0	0	0	0	0	0	0	k__Bacteria;p__Pseudomonadota;c__Gammaproteobacteria;o__Aeromonadales;f__Aeromonadaceae;g__Tolumonas;s__Tolumonas_auensis
NCBI43990	0	1	2	11	2	10	0	2	0	0	0	12	0	0	0	k__Bacteria;p__Actinomycetota;c__Actinomycetes;o__Mycobacteriales;f__Corynebacteriaceae;g__Corynebacterium;s__Corynebacterium_segmentosum
NCBI440	18	9	26	558	875	561	74	45	24	21	0	22	7	7	7	k__Bacteria;p__Pseudomonadota;c__Alphaproteobacteria;o__Rhodospirillales;f__Acetobacteraceae;g__Acetobac

																ter;s__Acetobacter_sp.
NCBI44008	3	4	0	3	18	12	6	0	2	3	0	1	0	0	0	k__Bacteria;p__Bacillota;c__Bacilli;o__Lactobacillales;f__Enterococcaceae;g__Enterococcus;s__Enterococcus_cecorum
NCBI442	566551	539356	610970	1640669	1548901	1145130	939927	561933	853416	3913298	3592207	5840686	5439754	5832996	7049880	k__Bacteria;p__Pseudomonadota;c__Alphaproteobacteria;o__Rhodospirillales;f__Acetobacteraceae;g__Gluconobacter;s__Gluconobacter_oxydans
NCBI444444	0	0	0	0	0	0	0	0	0	0	0	20	0	0	0	k__Bacteria;p__Pseudomonadota;c__Alphaproteobacteria;o__Hyphomicrobiales;f__Chelatococcaceae;g__Chelatococcus;s__Chelatococcus_daeguensis
NCBI44470	0	0	0	0	1	0	0	0	0	0	0	0	0	0	0	k__Archaea;p__Euryarchaeota;c__Halobacteria;o__Natrialbales;f__Natrialbaceae;g__Natronococcus;s__Natronococcus_amylolyticus
NCBI445576	7	3	0	25	24	44	0	0	0	0	0	2	2	0	0	k__Bacteria;p__Actinomycetota;c__Actinomycetes;o__Pseudonocardiales;f__Pseudonocardiaceae;g__Pseudonocardia;s__Pseudonocardia_sp._AL041005-10
NCBI445709	0	0	0	0	0	1	0	0	0	0	0	0	0	0	0	k__Bacteria;p__Pseudomonadota;c__Betaproteobacteria;o__Burkholderiales;f__Burkholderiaceae;g__Pandoraea;s__Pandoraea_thiooxydans
NCBI445710	0	0	0	0	0	1	0	0	0	0	0	0	0	0	0	k__Bacteria;p__Pseudomonadota;c__Gammaproteobacteria;o__Xanthomonadales;f__Rhodanobacteraceae;g__Dyella;s__Dyella_thiooxydans
NCBI44574	0	2	0	0	2	0	0	0	0	0	0	0	0	0	0	k__Bacteria;p__Pseudomonadota;c__Betaproteobacteria;o__Nitrosomonadales;f__Nitrosomonadaceae;g__Nitrosomonas;s__Nitrosomonas_communis
NCBI446	0	0	0	0	0	0	0	0	0	0	0	1	0	0	0	k__Bacteria;p__Pseudomonadota;c__Gammaproteobacteria;o__Legionellales;f__Legionellaceae;g__Legionella;s__Legionella_pneumophila
NCBI446660	0	0	0	0	20	358	0	0	0	0	0	91	0	0	0	k__Bacteria;p__Actinomycetota;c__Coriobacteriia;o__Eggerthellales;f__Eggerthellaceae;g__Adlercreutzia;s__Ad

															lercreutzia_equolifaciens
NCBI446692	142772	144203	150564	480479	374807	288954	188195	120296	171434	412824	394520	617705	3958088	4006054	4926961	k__Bacteria;p__Pseudomonadota;c__Alphaproteobacteria;o__Rhodospirillales;f__Acetobacteraceae;g__Acetobacter;s__Acetobacter_senegalensis
NCBI446860	0	0	0	0	2	0	0	0	0	0	0	1	0	0	0	k__Bacteria;p__Actinomycetota;c__Actinomycetes;o__Micrococcales;f__Micrococcaceae;g__Kocuria;s__Kocuria_flava
NCBI447265	0	0	0	0	0	0	9	0	0	0	0	17	0	0	0	k__Fungi;p__;c__;o__;f__;g__;s__uncultured_yeast
NCBI448181	80	0	47	0	176	1162	72	0	0	0	0	0	0	0	37	k__Bacteria;p__Pseudomonadota;c__Alphaproteobacteria;o__Hyphomicrobiales;f__Rhizobiaceae;g__Ciceribacter;s__Ciceribacter_selenitireducens
NCBI449461	0	0	0	0	0	77	0	0	0	0	0	0	0	0	0	k__Bacteria;p__Actinomycetota;c__Actinomycetes;o__Propionibacteriales;f__Nocardioidaceae;g__Nocardioides;s__Nocardioides_humi
NCBI450378	0	0	0	0	1	31	0	0	0	0	0	1	0	0	0	k__Bacteria;p__Pseudomonadota;c__Alphaproteobacteria;o__Sphingomonadales;f__Erythrobacteraceae;g__Croceicoccus;s__Croceicoccus_marinus
NCBI45133	0	0	0	0	0	0	0	0	0	0	0	3	0	0	0	k__Fungi;p__Ascomycota;c__Dothideomycetes;o__Botryosphaeriales;f__Botryosphaeriaceae;g__Lasiodiplodia;s__Lasiodiplodia_theobromae
NCBI45151	0	0	0	0	0	1	0	0	0	0	0	0	0	0	0	k__Fungi;p__Ascomycota;c__Dothideomycetes;o__Pleosporales;f__Pleosporaceae;g__Pyrenophora;s__Pyrenophora_tritici-repentis
NCBI451876	4	3	0	3	11	58	3	1	4	0	0	0	0	0	0	k__Bacteria;p__Pseudomonadota;c__Alphaproteobacteria;o__Hyphomicrobiales;f__Rhizobiaceae;g__Rhizobium;s__Rhizobium_rhizoryzae
NCBI453246	0	0	0	0	8	208	14	0	0	0	0	5	14	0	0	k__Bacteria;p__Pseudomonadota;c__Alphaproteobacteria;o__Sphingomonadales;f__Sphingomonadaceae;g__Sph

															ingobium;s__Sphingobium_sp._TCM1
NCBI453783	0	0	0	0	0	8	0	0	0	0	0	0	0	0	0	k__Bacteria;p__Pseudomonadota;c__Gammaproteobacteria;o__Xanthomonadales;f__Xanthomonadaceae;g__Lysobacter;s__Lysobacter_soli
NCBI454005	18	45	54	82	79	294	65	53	37	64	41	47	11	23	10	k__Bacteria;p__Pseudomonadota;c__Gammaproteobacteria;o__Enterobacterales;f__Yersiniaceae;g__Yersinia;s__uncultured_Yersinia_sp.
NCBI454155	0	0	0	0	0	1	1	0	0	0	0	1	0	0	0	k__Bacteria;p__Bacteroidota;c__Bacteroidia;o__Bacteroidales;f__Prevotellaceae;g__Paraprevotella;s__Paraprevotella_xylaniphila
NCBI456327	7	15	0	40	50	114	0	0	0	0	0	0	0	0	0	k__Bacteria;p__Pseudomonadota;c__Gammaproteobacteria;o__Xanthomonadales;f__Xanthomonadaceae;g__Xanthomonas;s__Xanthomonas_euvesicatoria
NCBI45634	0	0	0	2	3	2	0	0	0	0	0	3	0	0	0	k__Bacteria;p__Bacillota;c__Bacilli;o__Lactobacillales;f__Streptococcaceae;g__Streptococcus;s__Streptococcus_cristatus
NCBI456827	0	0	0	0	0	0	0	0	0	3	3	0	4	5	6	k__Bacteria;p__Candidatus_Cloacimonetes;c__;o__;f__;g__Candidatus_Cloacimonas;s__Candidatus_Cloacimonas_acidaminovorans
NCBI458253	57	61	130	109	117	411	48	47	32	45	57	50	16	16	26	k__Bacteria;p__Bacillota;c__Clostridia;o__Eubacteriales;f__Lachnospiraceae;g__Coprococcus;s__uncultured_Coprococcus_sp.
NCBI459529	0	0	0	0	0	1	0	0	0	0	0	0	0	0	0	k__Bacteria;p__Bacteroidota;c__Sphingobacteriia;o__Sphingobacteriales;f__Sphingobacteriaceae;g__Sphingobacterium;s__Sphingobacterium_siyangense
NCBI45972	0	1	0	0	0	0	0	0	0	0	0	0	0	0	0	k__Bacteria;p__Bacillota;c__Bacilli;o__Bacillales;f__Staphylococcaceae;g__Staphylococcus;s__Staphylococcus_pasteuri

NCBI460016	0	0	0	0	0	1	0	0	0	0	0	2	0	0	0	k__Bacteria;p__Bacillota;c__Bacilli;o__Bacillales;f__Bacillaceae;g__Bacillus;s__Bacillus_sp._XAL601
NCBI460519	11	8	7	51	145	76	17	3	11	1	2	2	4	0	1	k__Fungi;p__Ascomycota;c__Saccharomycetes;o__Saccharomycetales;f__Phaffomycetaceae;g__Komagataella;s__Komagataella_phaffii
NCBI460523	0	0	0	0	0	0	0	0	0	0	1	0	0	0	0	k__Fungi;p__Ascomycota;c__Saccharomycetes;o__Saccharomycetales;f__Pichiaceae;g__Ogataea;s__Ogataea_polymorpha
NCBI46124	7	3	1	11	29	21	2	11	1	0	0	5	2	0	0	k__Bacteria;p__Bacillota;c__Bacilli;o__Lactobacillales;f__Carnobacteriaceae;g__Granulicatella;s__Granulicatella_adiacens
NCBI46125	0	0	0	0	1	0	0	1	0	0	0	0	0	0	0	k__Bacteria;p__Bacillota;c__Bacilli;o__Lactobacillales;f__Aerococcaceae;g__Abiotrophia;s__Abiotrophia_defectiva
NCBI46126	0	0	0	0	0	2	0	0	0	0	0	0	0	0	0	k__Bacteria;p__Bacillota;c__Bacilli;o__Bacillales;f__Staphylococcaceae;g__Staphylococcus;s__Staphylococcus_chromogenes
NCBI46165	0	0	0	0	0	0	0	0	1	0	0	0	0	0	0	k__Bacteria;p__Actinomycetota;c__Actinomycetes;o__Streptosporangiales;f__Thermomonosporaceae;g__Actinomadura;s__Actinomadura_verrucosospora
NCBI46256	37	32	38	21	22	18	34	36	40	57	40	59	35	50	58	k__Bacteria;p__Bacillota;c__Bacilli;o__Lactobacillales;f__Lactobacillaceae;g__Weissella;s__Weissella_hellenica
NCBI46429	0	0	0	0	0	10	0	0	0	0	0	0	0	0	0	k__Bacteria;p__Pseudomonadota;c__Alphaproteobacteria;o__Sphingomonadales;f__Sphingomonadaceae;g__Sphingobium;s__Sphingobium_chlorophenolicum
NCBI46472	19	1	64	57	120	95	20	0	29	1	3	232	5	0	6	k__Fungi;p__Ascomycota;c__Eurotiomycetes;o__Eurotiales;f__Aspergillaceae;g__Aspergillus;s__Aspergillus_versicolor
NCBI46503	1	0	2	0	2	93	6	1	0	0	0	34	0	0	0	k__Bacteria;p__Bacteroidota;c__Bacteroidia;o__Bacteroidales;f__Tannerellaceae;g__Parabacteroides;s__Parabact

																eroides_merdae
NCBI46506	1	0	2	0	1	186	47	3	0	0	0	43	0	0	0	k__Bacteria;p__Bacteroidota;c__Bacteroidia;o__Bacteroidales;f__Bacteroidaceae;g__Bacteroides;s__Bacteroides_stercoris
NCBI465721	0	1	0	0	0	13	0	0	0	0	0	0	0	0	0	k__Bacteria;p__Pseudomonadota;c__Gammaproteobacteria;o__Nevskiales;f__Steroidobacteraceae;g__Steroidobacter;s__Steroidobacter_denitrificans
NCBI465797	0	9	8	19	0	15	15	0	14	0	0	0	0	0	0	k__Bacteria;p__Pseudomonadota;c__Gammaproteobacteria;o__Moraxellales;f__Moraxellaceae;g__Acinetobacter;s__Acinetobacter_septicus
NCBI466153	0	0	0	0	0	12	0	0	0	0	0	0	0	0	0	k__Bacteria;p__Planctomycetota;c__Planctomycetia;o__Isosphaerales;f__Isosphaeraceae;g__Singulisphaera;s__Singulisphaera_acidiphila
NCBI46617	0	0	0	0	0	0	15	0	0	0	0	0	0	0	0	k__Fungi;p__Ascomycota;c__Saccharomycetes;o__Saccharomycetales;f__Saccharomycetaceae;g__Saccharomyces;s__Saccharomyces_douglasii
NCBI46634	0	0	0	0	3	0	0	0	0	0	0	0	0	0	0	k__Fungi;p__Ascomycota;c__Dothideomycetes;o__Dothideales;f__Saccotheciaceae;g__Aureobasidium;s__Aureobasidium_melanogenum
NCBI467094	0	0	0	0	0	3	0	0	0	0	0	0	0	0	0	k__Bacteria;p__Actinomycetota;c__Acidimicrobiia;o__Acidimicrobiales;f__Ilumatobacteraceae;g__Ilumatobacter;s__Ilumatobacter_coccineus
NCBI468911	2415	2400	2497	2717	2784	2802	3140	3025	3021	3541	2893	2838	1492	1505	1486	k__Bacteria;p__Bacillota;c__Bacilli;o__Lactobacillales;f__Lactobacillaceae;g__Liquorilactobacillus;s__Liquorilactobacillus_hordei
NCBI470	72	21	83	35	437	914	114	19	28	25	33	152	0	172	70	k__Bacteria;p__Pseudomonadota;c__Gammaproteobacteria;o__Moraxellales;f__Moraxellaceae;g__Acinetobacter;s__Acinetobacter_baumannii
NCBI470565	0	1	0	0	1	0	0	0	0	0	0	0	0	0	0	k__Bacteria;p__Bacteroidota;c__Bacteroidia;o__Bacteroidales;f__Prevotellaceae;g__Prevotella;s__Prevotella_hist

																icola
NCBI470933	0	0	0	0	0	0	0	0	0	0	0	0	0	0	2	k__Bacteria;p__Pseudomonadota;c__Gammaproteobacteria;o__Enterobacterales;f__Erwiniaceae;g__Pantoea;s__Pantoea_eucalypti
NCBI470934	0	0	0	2	10	4	0	0	0	0	0	0	0	0	0	k__Bacteria;p__Pseudomonadota;c__Gammaproteobacteria;o__Enterobacterales;f__Erwiniaceae;g__Pantoea;s__Pantoea_vagans
NCBI471	1	3	0	0	1	5	1	1	3	0	0	10	0	1	0	k__Bacteria;p__Pseudomonadota;c__Gammaproteobacteria;o__Moraxellales;f__Moraxellaceae;g__Acinetobacter;s__Acinetobacter_calcoaceticus
NCBI471189	0	0	0	0	0	16	0	0	0	0	0	11	0	0	0	k__Bacteria;p__Actinomycetota;c__Coriobacteriia;o__Eggerthellales;f__Eggerthellaceae;g__Gordonibacter;s__Gordonibacter_pamelaeae
NCBI472695	0	0	0	0	2	0	0	0	0	0	0	0	0	0	0	k__Bacteria;p__Pseudomonadota;c__Gammaproteobacteria;o__Enterobacterales;f__Erwiniaceae;g__Pantoea;s__Pantoea_septica
NCBI472705	0	0	25	0	0	0	0	0	0	0	0	0	0	0	0	k__Bacteria;p__Pseudomonadota;c__Gammaproteobacteria;o__Enterobacterales;f__Erwiniaceae;g__Pantoea;s__Pantoea_conspicua
NCBI47421	0	7	1	0	14	114	0	0	5	0	0	0	1	0	0	k__Bacteria;p__Pseudomonadota;c__Betaproteobacteria;o__Burkholderiales;f__Comamonadaceae;g__Hydrogenophaga;s__Hydrogenophaga_pseudoflava
NCBI47493	3	1	1	0	0	0	0	1	2	0	0	7	0	0	0	k__Bacteria;p__Bacillota;c__Bacilli;o__Lactobacillales;f__Lactobacillaceae;g__Limosilactobacillus;s__Limosilactobacillus_panis
NCBI475937	0	0	0	0	3	1	0	0	0	0	0	0	1	0	0	k__Bacteria;p__Pseudomonadota;c__Alphaproteobacteria;o__Hyphomicrobiales;f__Nitrobacteraceae;g__Rhodopseudomonas;s__Rhodopseudomonas_boonkerdii
NCBI4	2	0	1	6	1	12	0	0	0	0	1	2	0	0	0	k__Bacteria;p__Pseudomonadota;c__Betaproteobacteria;

7671																o__Burkholderiales;f__Burkholderiaceae;g__Lautropia;s__Lautropia_mirabilis
NCBI47678	0	0	0	0	0	2	18	4	0	0	0	14	0	0	0	k__Bacteria;p__Bacteroidota;c__Bacteroidia;o__Bacteroidales;f__Bacteroidaceae;g__Bacteroides;s__Bacteroides_caccae
NCBI47715	7254	7422	7686	12348	11888	2637	11806	11563	11802	13809	12013	15826	5939	6334	6592	k__Bacteria;p__Bacillota;c__Bacilli;o__Lactobacillales;f__Lactobacillaceae;g__Lacticaseibacillus;s__Lacticaseibacillus_rhamnosus
NCBI477641	0	0	0	0	0	57	0	0	0	0	0	0	0	0	0	k__Bacteria;p__Actinomycetota;c__Actinomycetes;o__Geodermatophilales;f__Geodermatophilaceae;g__Modestobacter;s__Modestobacter_marinus
NCBI47770	1	7	10	0	12	23	4	17	4	1	6	7	0	3	0	k__Bacteria;p__Bacillota;c__Bacilli;o__Lactobacillales;f__Lactobacillaceae;g__Lactobacillus;s__Lactobacillus_crispatus
NCBI477976	0	0	0	0	0	0	0	0	2	0	0	0	0	0	0	k__Bacteria;p__Deferribacterota;c__Deferribacteres;o__Deferribacterales;f__Calditerrivibrionaceae;g__Calditerrivibrio;s__Calditerrivibrio_nitroreducens
NCBI47853	0	0	0	4	0	0	0	0	0	0	0	0	0	0	0	k__Bacteria;p__Actinomycetota;c__Actinomycetes;o__Micromonosporales;f__Micromonosporaceae;g__Micromonospora;s__Micromonospora_carbonacea
NCBI47858	0	0	0	0	0	65	0	0	0	0	0	0	0	0	0	k__Bacteria;p__Actinomycetota;c__Actinomycetes;o__Micromonosporales;f__Micromonosporaceae;g__Micromonospora;s__Micromonospora_echinofusca
NCBI47875	0	0	0	0	0	46	0	0	0	0	0	0	0	0	0	k__Bacteria;p__Actinomycetota;c__Actinomycetes;o__Micromonosporales;f__Micromonosporaceae;g__Micromonospora;s__Micromonospora_sagamiensis
NCBI47877	17	20	13	212	394	518	86	7	24	2	0	6	5	0	0	k__Bacteria;p__Pseudomonadota;c__Gammaproteobacteria;o__Pseudomonadales;f__Pseudomonadaceae;g__Pseudomonas;s__Pseudomonas_amygdali
NCBI4	0	0	1	14	22	55	17	0	1	4	0	1	1	0	0	k__Bacteria;p__Pseudomonadota;c__Gammaproteobacte

7878																ria;o__Pseudomonadales;f__Pseudomonadaceae;g__Pseudomonas;s__Pseudomonas_azotoformans
NCBI47880	0	0	0	21	0	9	15	0	0	0	0	0	0	0	0	k__Bacteria;p__Pseudomonadota;c__Gammaproteobacteria;o__Pseudomonadales;f__Pseudomonadaceae;g__Pseudomonas;s__Pseudomonas_fulva
NCBI47883	5	1	0	15	30	50	6	0	1	0	0	0	0	0	0	k__Bacteria;p__Pseudomonadota;c__Gammaproteobacteria;o__Pseudomonadales;f__Pseudomonadaceae;g__Pseudomonas;s__Pseudomonas_synxantha
NCBI47884	0	0	0	0	0	1	0	0	0	0	0	0	0	0	0	k__Bacteria;p__Pseudomonadota;c__Gammaproteobacteria;o__Pseudomonadales;f__Pseudomonadaceae;g__Pseudomonas;s__Pseudomonas_taetrolens
NCBI47885	15	9	5	88	151	148	15	1	10	0	0	2	0	0	2	k__Bacteria;p__Pseudomonadota;c__Gammaproteobacteria;o__Pseudomonadales;f__Pseudomonadaceae;g__Pseudomonas;s__Pseudomonas_oryzihabitans
NCBI47903	5	6	7	27	78	69	11	3	7	2	1	2	0	0	2	k__Pararnavirae;p__Artverviricota;c__Revtraviricetes;o__Ortervirales;f__Caulimoviridae;g__Caulimovirus;s__Strawberry_vein_banding_virus
NCBI48	0	0	0	0	0	170	0	0	0	0	0	1	0	0	0	k__Bacteria;p__Myxococcota;c__Myxococcia;o__Myxococcales;f__Archangiaceae;g__Archangium;s__Archangium_gephyra
NCBI480	0	0	0	0	0	1	0	0	0	0	0	0	0	0	0	k__Bacteria;p__Pseudomonadota;c__Gammaproteobacteria;o__Moraxellales;f__Moraxellaceae;g__Moraxella;s__Moraxella_catarrhalis
NCBI480931	28	24	29	22	18	15	46	56	49	59	68	55	36	27	36	k__Bacteria;p__Bacillota;c__Bacilli;o__Lactobacillales;f__Lactobacillaceae;g__Liquorilactobacillus;s__Liquorilactobacillus_capillatus
NCBI481146	2417	2407	2353	6870	5323	4978	3085	2168	2903	7650	7057	10280	73992	70022	88467	k__Bacteria;p__Pseudomonadota;c__Alphaproteobacteria;o__Rhodospirillales;f__Acetobacteraceae;g__Acetobacter;s__Acetobacter_ascendens

NCBI482462	0	2	0	0	0	0	0	0	1	0	0	1	0	0	0	k__Bacteria;p__Actinomycetota;c__Actinomycetes;o__Mycobacteriales;f__Mycobacteriaceae;g__Mycobacterium;s__Mycobacterium_dioxanotrophicus
NCBI482564	3	0	0	0	22	41	14	0	1	1	0	0	0	0	0	k__Bacteria;p__Cyanobacteriota;c__Cyanophyceae;o__Oscillatoriales;f__Oscillatoriaceae;g__Oscillatoria;s__Oscillatoria_nigro-viridis
NCBI482957	0	0	0	5	0	10	0	0	0	0	0	0	0	0	0	k__Bacteria;p__Pseudomonadota;c__Betaproteobacteria;o__Burkholderiales;f__Burkholderiaceae;g__Burkholderia;s__Burkholderia_lata
NCBI48296	143	167	93	270	680	1637	228	50	85	20	11	94	62	48	36	k__Bacteria;p__Pseudomonadota;c__Gammaproteobacteria;o__Moraxellales;f__Moraxellaceae;g__Acinetobacter;s__Acinetobacter_pittii
NCBI48383	0	0	0	0	0	2	0	0	0	0	0	0	0	0	0	k__Bacteria;p__Pseudomonadota;c__Betaproteobacteria;o__Neisseriales;f__Neisseriaceae;g__Neisseria;s__Neisseria_cinerea
NCBI483199	10	0	20	35	14	87	10	4	19	13	9	27	57	18	52	k__Bacteria;p__Pseudomonadota;c__Alphaproteobacteria;o__Rhodospirillales;f__Acetobacteraceae;g__Acetobacter;s__Acetobacter_fabarum
NCBI4837	0	0	0	4	0	0	0	0	0	0	0	0	0	0	0	k__Fungi;p__Mucoromycota;c__Mucoromycetes;o__Mucorales;f__Phycomycetaceae;g__Phycomyces;s__Phycomyces_blakesleeanus
NCBI484429	16	0	0	0	0	37	0	0	0	0	0	0	0	0	0	k__Bacteria;p__Pseudomonadota;c__Alphaproteobacteria;o__Sphingomonadales;f__Sphingomonadaceae;g__Sphingobium;s__Sphingobium_sp._YBL2
NCBI485895	0	0	0	0	1	0	0	0	0	0	0	0	0	0	0	k__Bacteria;p__Pseudomonadota;c__Gammaproteobacteria;o__Pseudomonadales;f__Pseudomonadaceae;g__Pseudomonas;s__Pseudomonas_wayambapalatensis
NCBI487	0	2	0	0	0	0	0	0	0	0	0	10	0	0	0	k__Bacteria;p__Pseudomonadota;c__Betaproteobacteria;o__Neisseriales;f__Neisseriaceae;g__Neisseria;s__Neisseria_meningitidis

NCBI487316	3	5	0	20	43	64	4	2	4	1	1	3	2	1	1	k__Bacteria;p__Pseudomonadota;c__Gammaproteobacteria;o__Moraxellales;f__Moraxellaceae;g__Acinetobacter;s__Acinetobacter_soli
NCBI487698	0	0	0	0	0	185	0	0	0	0	0	0	0	0	0	k__Bacteria;p__Pseudomonadota;c__Gammaproteobacteria;o__Xanthomonadales;f__Xanthomonadaceae;g__Stenotrophomonas;s__Stenotrophomonas_pavanii
NCBI488	0	0	0	3	0	0	0	0	0	0	0	0	0	0	0	k__Bacteria;p__Pseudomonadota;c__Betaproteobacteria;o__Neisseriales;f__Neisseriaceae;g__Neisseria;s__Neisseria_mucosa
NCBI488447	2	0	0	0	17	32	1	0	0	4	0	2	0	0	5	k__Bacteria;p__Pseudomonadota;c__Betaproteobacteria;o__Burkholderiales;f__Burkholderiaceae;g__Burkholderia;s__Burkholderia_contaminans
NCBI488729	0	0	0	0	0	1	0	0	0	0	0	0	0	0	0	k__Bacteria;p__Pseudomonadota;c__Betaproteobacteria;o__Burkholderiales;f__Burkholderiaceae;g__Burkholderia;s__Burkholderia_metallica
NCBI488731	6	13	7	2	12	53	6	0	2	0	0	8	5	1	0	k__Bacteria;p__Pseudomonadota;c__Betaproteobacteria;o__Burkholderiales;f__Burkholderiaceae;g__Burkholderia;s__Burkholderia_seminalis
NCBI48935	346	318	211	29	905	9559	353	18	153	73	84	131	44	24	26	k__Bacteria;p__Pseudomonadota;c__Alphaproteobacteria;o__Sphingomonadales;f__Sphingomonadaceae;g__Novosphingobium;s__Novosphingobium_aromaticivorans
NCBI490	2	0	0	0	0	11	0	1	0	0	0	22	0	0	0	k__Bacteria;p__Pseudomonadota;c__Betaproteobacteria;o__Neisseriales;f__Neisseriaceae;g__Neisseria;s__Neisseria_sicca
NCBI4909	0	0	0	0	0	11	0	0	0	0	22	4	0	0	6	k__Fungi;p__Ascomycota;c__Saccharomycetes;o__Saccharomycetales;f__Pichiaceae;g__Pichia;s__Pichia_kudriavzevii
NCBI4911	0	0	0	0	0	0	0	0	9	0	0	15	0	0	7	k__Fungi;p__Ascomycota;c__Saccharomycetes;o__Saccharomycetales;f__Saccharomycetaceae;g__Kluyveromyces;s__Kluyveromyces_marxianus

NCBI4926	0	0	1	0	0	0	1	0	1	1	1	0	3	0	3	k__Fungi;p__Ascomycota;c__Saccharomycetes;o__Saccharomycetales;f__Pichiaceae;g__Pichia;s__Pichia_membranifaciens
NCBI492670	1099	1062	1032	6143	9143	5364	3357	433	1221	188	218	278	202	177	158	k__Bacteria;p__Bacillota;c__Bacilli;o__Bacillales;f__Bacillaceae;g__Bacillus;s__Bacillus_velezensis
NCBI4927	0	0	0	0	0	0	0	0	0	0	0	2	0	0	0	k__Fungi;p__Ascomycota;c__Saccharomycetes;o__Saccharomycetales;f__Phaffomycetaceae;g__Wickerhamomyces;s__Wickerhamomyces_anomalus
NCBI4929	0	0	0	0	0	1	2	0	0	0	0	14	0	0	1	k__Fungi;p__Ascomycota;c__Saccharomycetes;o__Saccharomycetales;f__Debaryomycetaceae;g__Meyerozyma;s__Meyerozyma_guilliermondii
NCBI4931	2	0	0	1	0	0	0	0	0	0	1	0	0	1	0	k__Fungi;p__Ascomycota;c__Saccharomycetes;o__Saccharomycetales;f__Saccharomycetaceae;g__Saccharomyces;s__Saccharomyces_bayanus
NCBI49319	0	0	0	0	0	5	0	0	0	0	0	0	0	0	0	k__Bacteria;p__Actinomycetota;c__Rubrobacteria;o__Rubrobacterales;f__Rubrobacteraceae;g__Rubrobacter;s__Rubrobacter_xylanophilus
NCBI4932	17275098	18879126	17954479	12512068	10175943	5998462	14411479	16042038	16544957	13388856	11087896	11497439	3411560	3319926	3231748	k__Fungi;p__Ascomycota;c__Saccharomycetes;o__Saccharomycetales;f__Saccharomycetaceae;g__Saccharomyces;s__Saccharomyces_cerevisiae
NCBI495	0	0	0	0	0	3	0	0	0	0	0	0	0	0	0	k__Bacteria;p__Pseudomonadota;c__Betaproteobacteria;o__Neisseriales;f__Neisseriaceae;g__Neisseria;s__Neisseria_elongata
NCBI4952	17	1	11	6	11	9	0	0	0	24	0	0	0	0	0	k__Fungi;p__Ascomycota;c__Saccharomycetes;o__Saccharomycetales;f__Dipodascaceae;g__Yarrowia;s__Yarrowia_lipolytica
NCBI4959	0	1	1	3	6	6	3	0	0	0	0	0	0	0	0	k__Fungi;p__Ascomycota;c__Saccharomycetes;o__Saccharomycetales;f__Debaryomycetaceae;g__Debaryomyces;s__Debaryomyces_hansenii

NCBI496014	0	0	0	0	1	6	0	0	0	0	0	0	0	0	0	k__Bacteria;p__Actinomycetota;c__Rubrobacteria;o__Rubrobacterales;f__Baekduiaceae;g__Baekduia;s__Baekduia_soli
NCBI497727	0	0	0	0	0	4	0	0	0	0	0	0	0	0	0	k__Archaea;p__Nitrososphaerota;c__Nitrososphaeria;o__Nitrososphaerales;f__Nitrososphaeraceae;g__Nitrososphaera;s__Candidatus_Nitrososphaera_gargensis
NCBI499555	0	0	1	0	0	0	0	0	0	0	0	0	0	0	0	k__Bacteria;p__Actinomycetota;c__Actinomycetes;o__Mycobacteriales;f__Dietziaceae;g__Dietzia;s__Dietzia_timorensis
NCBI5007	0	0	0	0	0	1	0	0	0	0	0	0	0	0	0	k__Fungi;p__Ascomycota;c__Saccharomycetes;o__Saccharomycetales;f__Pichiaceae;g__Brettanomyces;s__Brettanomyces_bruxellensis
NCBI502394	0	0	1	5	4	3	3	0	3	0	0	1	0	0	0	k__Bacteria;p__Deinococcota;c__Deinococci;o__Deinococcales;f__Deinococcaceae;g__Deinococcus;s__Deinococcus_gobiensis
NCBI503996	51	137	34	0	108	2743	106	6	25	10	0	27	35	36	18	k__Bacteria;p__Pseudomonadota;c__Betaproteobacteria;o__Burkholderiales;f__;g__Aquabacterium;s__Aquabacterium_sp._PL1F5
NCBI503999	0	0	0	0	0	9	0	0	0	0	0	0	0	0	0	k__Bacteria;p__Pseudomonadota;c__Betaproteobacteria;o__Rhodocyclales;f__Zoogloeaceae;g__Thauera;s__Thauera_sp._B4
NCBI504461	0	0	0	0	0	6	0	0	0	0	0	0	0	0	0	k__Bacteria;p__Pseudomonadota;c__Alphaproteobacteria;o__Caulobacterales;f__Caulobacteraceae;g__Caulobacter;s__Caulobacter_sp._FWC42
NCBI505	0	0	0	0	6	0	0	0	0	0	0	0	0	0	0	k__Bacteria;p__Pseudomonadota;c__Betaproteobacteria;o__Neisseriales;f__Neisseriaceae;g__Kingella;s__Kingella_oralis
NCBI5059	0	8	0	9	53	65	20	0	7	0	0	0	0	0	0	k__Fungi;p__Ascomycota;c__Eurotiomycetes;o__Eurotiales;f__Aspergillaceae;g__Aspergillus;s__Aspergillus_flavus

NCBI5061	26	21	36	103	202	111	46	8	10	0	0	33	0	6	3	k__Fungi;p__Ascomycota;c__Eurotiomycetes;o__Eurotiales;f__Aspergillaceae;g__Aspergillus;s__Aspergillus_niger
NCBI5068	0	0	0	0	0	1	0	0	0	0	0	0	0	0	0	k__Fungi;p__Ascomycota;c__Eurotiomycetes;o__Eurotiales;f__Aspergillaceae;g__Aspergillus;s__Aspergillus_tubingensis
NCBI50719	0	0	0	0	0	0	0	0	0	0	0	42	0	0	0	k__Bacteria;p__Pseudomonadota;c__Gammaproteobacteria;o__Vibrionales;f__Vibrionaceae;g__Vibrio;s__Vibrio_diabolicus
NCBI50741	0	0	0	0	0	0	0	0	0	0	0	1	0	0	0	k__Bacteria;p__Pseudomonadota;c__Gammaproteobacteria;o__Pseudomonadales;f__Marinobacteraceae;g__Marinobacter;s__Marinobacter_sp.
NCBI508451	0	0	0	0	1	0	1	0	0	0	0	0	0	0	0	k__Bacteria;p__Bacillota;c__Bacilli;o__Lactobacillales;f__Lactobacillaceae;g__Lactobacillus;s__Lactobacillus_taiwanensis
NCBI511	0	0	0	7	71	13	33	0	3	0	0	14	0	0	0	k__Bacteria;p__Pseudomonadota;c__Betaproteobacteria;o__Burkholderiales;f__Alcaligenaceae;g__Alcaligenes;s__Alcaligenes_faecalis
NCBI51101	0	0	21	0	0	0	0	0	0	0	0	0	0	0	0	k__Bacteria;p__Bacillota;c__Bacilli;o__Bacillales;f__Paenibacillaceae;g__Brevibacillus;s__Brevibacillus_agri
NCBI511062	0	0	0	0	0	0	0	0	0	0	0	1	0	0	0	k__Bacteria;p__Pseudomonadota;c__Gammaproteobacteria;o__Aeromonadales;f__Aeromonadaceae;g__Oceanimonas;s__Oceanimonas_sp._GK1
NCBI51173	1	0	0	0	0	0	0	0	0	0	0	6	0	0	0	k__Bacteria;p__Bacillota;c__Bacilli;o__Bacillales;f__Planococcaceae;g__Ureibacillus;s__Ureibacillus_thermosphaericus
NCBI51197	0	0	0	0	0	0	0	0	1	0	0	0	0	0	0	k__Bacteria;p__Bacillota;c__Clostridia;o__Eubacteriales;f__Desulfitobacteriaceae;g__Syntrophobotulus;s__Syntrophobotulus_glycolicus
NCBI5	0	0	0	0	0	0	0	0	0	0	0	1	0	0	0	k__Fungi;p__Ascomycota;c__Sordariomycetes;o__Hypo

127																creales;f__Nectriaceae;g__Fusarium;s__Fusarium_fujikuroi
NCBI512763	0	0	0	0	0	1	0	0	0	0	0	0	0	0	0	k__Bacteria;p__Bacteroidota;c__Cytophagia;o__Cytophagales;f__Hymenobacteraceae;g__Rufibacter;s__Rufibacter_tibetensis
NCBI5141	0	0	0	0	0	104	0	0	0	0	0	2	0	0	0	k__Fungi;p__Ascomycota;c__Sordariomycetes;o__Sordariales;f__Sordariaceae;g__Neurospora;s__Neurospora_crassa
NCBI51453	159	157	99	1747	3243	1810	449	80	140	12	16	40	30	38	30	k__Fungi;p__Ascomycota;c__Sordariomycetes;o__Hypocreales;f__Hypocreaceae;g__Trichoderma;s__Trichoderma_reesei
NCBI5147	1	0	3	0	6	66	2	0	0	0	0	1	0	0	0	k__Fungi;p__Ascomycota;c__Sordariomycetes;o__Sordariales;f__Sordariaceae;g__Sordaria;s__Sordaria_macrospora
NCBI51660	0	1	0	0	0	0	0	0	0	0	0	0	0	0	1	k__Fungi;p__Ascomycota;c__Saccharomycetes;o__Saccharomycetales;f__Saccharomycetaceae;g__Nakaseomyces;s__Nakaseomyces_bacillisporus
NCBI51663	115	111	131	176	215	144	123	118	149	122	96	110	57	60	57	k__Bacteria;p__Bacillota;c__Bacilli;o__Lactobacillales;f__Lactobacillaceae;g__Pediococcus;s__Pediococcus_damnosus
NCBI51665	0	0	315	0	0	0	0	0	0	0	0	0	0	0	0	k__Bacteria;p__Bacillota;c__Bacilli;o__Lactobacillales;f__Aerococcaceae;g__Aerococcus;s__Aerococcus_urinaeequi
NCBI51669	0	0	5	0	0	0	0	0	0	0	0	0	0	0	0	k__Bacteria;p__Bacillota;c__Bacilli;o__Lactobacillales;f__Enterococcaceae;g__Tetragenococcus;s__Tetragenococcus_halophilus
NCBI517719	0	0	0	2	0	0	0	0	0	0	0	0	0	0	0	k__Bacteria;p__Pseudomonadota;c__Alphaproteobacteria;o__Rhodobacterales;f__Paracoccaceae;g__Pseudooceanicola;s__Pseudooceanicola_nitratireducens
NCBI5	0	4	0	0	10	56	11	0	0	26	20	30	426	283	431	k__Bacteria;p__Pseudomonadota;c__Betaproteobacteria;

18																o__Burkholderiales;f__Alcaligenaceae;g__Bordetella;s__Bordetella_bronchiseptica
NCBI519	0	0	0	0	0	6	0	0	0	0	0	0	0	0	0	k__Bacteria;p__Pseudomonadota;c__Betaproteobacteria;o__Burkholderiales;f__Alcaligenaceae;g__Bordetella;s__Bordetella_parapertussis
NCBI5207	0	0	0	0	0	3	0	0	0	0	0	0	0	0	0	k__Fungi;p__Basidiomycota;c__Tremellomycetes;o__Tremellales;f__Cryptococcaceae;g__Cryptococcus;s__Cryptococcus_neoformans
NCBI520734	15	14	0	0	43	415	17	0	0	0	0	0	0	0	0	k__Bacteria;p__Pseudomonadota;c__Alphaproteobacteria;o__Sphingomonadales;f__Sphingomonadaceae;g__Sphingobium;s__Sphingobium_sp._RSMS
NCBI521	0	0	0	0	0	4	0	0	0	0	0	0	0	0	0	k__Bacteria;p__Pseudomonadota;c__Betaproteobacteria;o__Burkholderiales;f__Alcaligenaceae;g__Bordetella;s__Bordetella_avium
NCBI52133	5	2	2	0	5	30	1	0	2	0	5	10	20	0	0	k__Bacteria;p__Pseudomonadota;c__Gammaproteobacteria;o__Moraxellales;f__Moraxellaceae;g__Acinetobacter;s__Acinetobacter_venetianus
NCBI52227	0	0	0	0	0	1	0	0	0	0	0	0	0	0	0	k__Bacteria;p__Bacteroidota;c__Bacteroidia;o__Bacteroidales;f__Prevotellaceae;g__Prevotella;s__Prevotella_dentalis
NCBI52242	0	4	6	9	7	3	0	1	2	0	0	1	0	0	3	k__Bacteria;p__Bacillota;c__Bacilli;o__Lactobacillales;f__Lactobacillaceae;g__Lactobacillus;s__Lactobacillus_gallinarum
NCBI52256	0	0	0	0	0	0	0	0	0	39	0	0	0	0	0	k__Fungi;p__Ascomycota;c__Saccharomycetes;o__Saccharomycetales;f__Pichiaceae;g__Pichia;s__[Candida]_ethanolica
NCBI5286	0	0	0	0	0	0	0	0	0	0	0	1	0	0	0	k__Fungi;p__Basidiomycota;c__Microbotryomycetes;o__Sporidiobolales;f__Sporidiobolaceae;g__Rhodotorula;s__Rhodotorula_toruloides
NCBI5	7	4	7	15	60	249	19	0	0	0	0	0	0	0	0	k__Bacteria;p__Pseudomonadota;c__Alphaproteobacteri

29															a;o__Hyphomicrobiales;f__Brucellaceae;g__Brucella;s__Brucella_anthropi
NCBI5319	0	0	0	0	0	0	0	0	0	5	0	0	0	0	0	k__Fungi;p__Basidiomycota;c__Agaricomycetes;o__Polyporales;f__Irpicaceae;g__Irpex;s__Irpex_lacteus
NCBI5334	0	0	0	0	4	49	1	0	0	0	1	0	0	0	0	k__Fungi;p__Basidiomycota;c__Agaricomycetes;o__Agaricales;f__Schizophyllaceae;g__Schizophyllum;s__Schizophyllum_commune
NCBI53345	0	0	0	0	4	0	0	0	0	0	0	0	0	0	0	k__Bacteria;p__Bacillota;c__Bacilli;o__Lactobacillales;f__Enterococcaceae;g__Enterococcus;s__Enterococcus_durans
NCBI53346	21	20	6	73	100	48	35	9	7	1	5	2	5	6	5	k__Bacteria;p__Bacillota;c__Bacilli;o__Lactobacillales;f__Enterococcaceae;g__Enterococcus;s__Enterococcus_mundtii
NCBI53374	0	0	0	1	0	0	0	0	0	0	0	0	0	0	0	k__Bacteria;p__Actinomycetota;c__Actinomycetes;o__Mycobacteriales;f__Corynebacteriaceae;g__Corynebacterium;s__Corynebacterium_coyleae
NCBI53399	0	0	0	0	0	1	0	0	0	0	0	0	0	0	0	k__Bacteria;p__Pseudomonadota;c__Alphaproteobacteria;o__Hyphomicrobiales;f__Hyphomicrobiaceae;g__Hyphomicrobium;s__Hyphomicrobium_denitrificans
NCBI53408	0	0	0	4	0	0	0	0	0	0	0	0	0	0	0	k__Bacteria;p__Pseudomonadota;c__Gammaproteobacteria;o__Pseudomonadales;f__Pseudomonadaceae;g__Pseudomonas;s__Pseudomonas_citronellolis
NCBI53437	0	0	0	0	33	1	0	0	0	0	0	0	2	0	0	k__Bacteria;p__Actinomycetota;c__Actinomycetes;o__Streptosporangiales;f__Nocardiopsaceae;g__Nocardiopsis;s__Nocardiopsis_alba
NCBI53442	0	0	0	0	0	48	0	0	0	0	0	0	0	0	0	k__Bacteria;p__Bacillota;c__Clostridia;o__Eubacteriales;f__Eubacteriaceae;g__Eubacterium;s__Eubacterium_callanderi
NCBI53444	1	0	0	0	0	0	0	0	0	0	0	0	0	0	0	k__Bacteria;p__Bacillota;c__Bacilli;o__Lactobacillales;f__Lactobacillaceae;g__Fructilactobacillus;s__Fructilacto

																bacillus_lindneri
NCBI53458	0	0	1	4	3	42	0	0	0	0	0	9	0	0	0	k__Bacteria;p__Actinomycetota;c__Actinomycetes;o__Micrococcales;f__Intrasporangiaceae;g__Janibacter;s__Janibacter_limosus
NCBI5346	0	0	0	0	0	3	0	0	0	0	0	0	0	0	0	k__Fungi;p__Basidiomycota;c__Agaricomycetes;o__Agaricales;f__Psathyrellaceae;g__Coprinopsis;s__Coprinopsis_cinerea
NCBI53461	0	0	1	0	22	7	0	0	0	0	0	0	0	0	0	k__Bacteria;p__Actinomycetota;c__Actinomycetes;o__Nakamurellales;f__Nakamurellaceae;g__Nakamurella;s__Nakamurella_multipartita
NCBI535744	0	0	0	0	0	3	0	0	0	0	0	0	0	0	0	k__Bacteria;p__Pseudomonadota;c__Gammaproteobacteria;o__Enterobacterales;f__Enterobacteriaceae;g__Cronobacter;s__Cronobacter_universalis
NCBI536	0	0	0	0	0	1	0	0	0	0	0	0	0	0	0	k__Bacteria;p__Pseudomonadota;c__Betaproteobacteria;o__Neisseriales;f__Chromobacteriaceae;g__Chromobacterium;s__Chromobacterium_violaceum
NCBI536441	0	0	0	1	0	0	0	0	0	0	4	0	0	0	0	k__Bacteria;p__Bacteroidota;c__Flavobacteriia;o__Flavobacteriales;f__Weeksellaceae;g__Chryseobacterium;s__Chryseobacterium_taklimakanense
NCBI53660	89	98	77	185	79	0	93	84	98	71	106	102	104	76	59	k__Fungi;p__Ascomycota;c__Saccharomycetes;o__Saccharomycetales;f__Pichiaceae;g__Pichia;s__Pichia_deserticola
NCBI538949	0	0	0	0	0	0	3	0	0	0	0	0	0	0	0	k__Bacteria;p__Bacteroidota;c__Bacteroidia;o__Bacteroidales;f__Rikenellaceae;g__Alistipes;s__uncultured_Alistipes_sp.
NCBI538966	0	0	0	1	0	0	0	0	0	0	0	0	0	0	0	k__Bacteria;p__Bacteroidota;c__Cytophagia;o__Cytophagales;f__Spirosomaceae;g__Dyadobacter;s__Dyadobacter_sp._32
NCBI539	0	0	0	0	0	0	0	0	2	0	0	0	0	0	0	k__Bacteria;p__Pseudomonadota;c__Betaproteobacteria;o__Neisseriales;f__Neisseriaceae;g__Eikenella;s__Eiken

															ella_corrodens
NCBI539813	0	0	2	2	0	3	0	0	7	0	0	29	0	0	0	k__Bacteria;p__Pseudomonadota;c__Gammaproteobacteria;o__Enterobacterales;f__Enterobacteriaceae;g__Enterobacter;s__Enterobacter_mori
NCBI54005	0	0	2	0	0	1	1	1	0	0	0	0	0	0	0	k__Bacteria;p__Bacillota;c__Tissierellia;o__Tissierellales;f__Peptoniphilaceae;g__Peptoniphilus;s__Peptoniphilus_harei
NCBI54061	3	5	5	11	28	36	15	2	1	1	3	9	0	2	2	k__Bacteria;p__Pseudomonadota;c__Betaproteobacteria;o__Burkholderiales;f__Burkholderiaceae;g__Ralstonia;s__Ralstonia_sp.
NCBI540747	0	0	0	0	0	0	4	0	0	0	0	0	0	0	0	k__Bacteria;p__Pseudomonadota;c__Alphaproteobacteria;o__Rhodobacterales;f__Roseobacteraceae;g__Roseovarius;s__Roseovarius_indicus
NCBI54199	0	0	0	0	0	0	0	0	0	1	0	0	0	0	0	k__Fungi;p__Ascomycota;c__Saccharomycetes;o__Saccharomycetales;f__Saccharomycetaceae;g__Cyniclomyces;s__Cyniclomyces_guttulatus
NCBI542	0	0	1	0	0	4	0	0	1	13	16	43	54	23	27	k__Bacteria;p__Pseudomonadota;c__Alphaproteobacteria;o__Sphingomonadales;f__Zymomonadaceae;g__Zymomonas;s__Zymomonas_mobilis
NCBI54291	0	0	0	0	0	8	0	0	0	0	0	0	0	0	0	k__Bacteria;p__Pseudomonadota;c__Gammaproteobacteria;o__Enterobacterales;f__Enterobacteriaceae;g__Raoultella;s__Raoultella_ornithinolytica
NCBI54299	0	0	0	0	0	1	0	0	0	0	0	1	0	0	0	k__Bacteria;p__Cyanobacteriota;c__Cyanophyceae;o__Chroococcidiopsidales;f__Chroococcidiopsidaceae;g__Chroococcidiopsis;s__Chroococcidiopsis_thermalis
NCBI543047	83	54	111	63	35	134	153	128	242	177	81	52	16	48	48	k__Bacteria;p__Pseudomonadota;c__Gammaproteobacteria;o__Cardiobacteriales;f__Cardiobacteriaceae;g__Dichelobacter;s__uncultured_Dichelobacter_sp.
NCBI544580	0	0	0	0	12	11	0	112	0	0	0	5	0	0	0	k__Bacteria;p__Actinomycetota;c__Actinomycetes;o__Actinomycetales;f__Actinomycetaceae;g__Actinomyces;s_

																_Actinomyces_oris
NCBI544645	1	0	0	0	7	9	44	0	7	0	0	3	0	0	0	k__Bacteria;p__Bacteroidota;c__Bacteroidia;o__Bacteroidales;f__Odoribacteraceae;g__Butyricimonas;s__Butyricimonas_virosa
NCBI545	0	0	0	0	0	0	0	28	0	0	0	102	0	0	0	k__Bacteria;p__Pseudomonadota;c__Gammaproteobacteria;o__Enterobacterales;f__Enterobacteriaceae;g__Citrobacter;s__Citrobacter_koseri
NCBI545908	0	0	1	0	0	3	0	0	0	0	0	0	0	0	0	k__Bacteria;p__Pseudomonadota;c__Gammaproteobacteria;o__Pseudomonadales;f__Pseudomonadaceae;g__Pseudomonas;s__Pseudomonas_sp._LM8
NCBI546	67	0	20	106	212	394	99	0	31	0	0	31	0	0	0	k__Bacteria;p__Pseudomonadota;c__Gammaproteobacteria;o__Enterobacterales;f__Enterobacteriaceae;g__Citrobacter;s__Citrobacter_freundii
NCBI546160	0	0	0	1	0	0	0	0	0	0	0	0	0	0	0	k__Bacteria;p__Actinomycetota;c__Actinomycetes;o__Mycobacteriales;f__Dietziaceae;g__Dietzia;s__Dietzia_lutea
NCBI546367	0	0	2	12	2	15	0	0	0	0	0	0	0	0	0	k__Bacteria;p__Pseudomonadota;c__Gammaproteobacteria;o__Enterobacterales;f__Hafniaceae;g__Hafnia;s__Hafnia_paralvei
NCBI546871	0	0	0	0	6	16	0	0	0	0	0	0	0	0	0	k__Bacteria;p__Actinomycetota;c__Actinomycetes;o__Propionibacteriales;f__Nocardioidaceae;g__Friedmanniella;s__Friedmanniella_luteola
NCBI546874	0	0	0	0	0	0	2	0	0	0	0	0	0	0	0	k__Bacteria;p__Actinomycetota;c__Actinomycetes;o__Propionibacteriales;f__Propionibacteriaceae;g__Microlunatus;s__Microlunatus_sagamiharensis
NCBI5478	0	0	0	0	0	2	0	0	0	0	0	0	0	0	0	k__Fungi;p__Ascomycota;c__Saccharomycetes;o__Saccharomycetales;f__Saccharomycetaceae;g__Nakaseomyces;s__Nakaseomyces_glabratus
NCBI5	0	0	5	0	2	190	4	0	0	0	0	1	4	0	0	k__Bacteria;p__Pseudomonadota;c__Gammaproteobacte

48																ria;o__Enterobacterales;f__Enterobacteriaceae;g__Klebsiella;s__Klebsiella_aerogenes
NCBI5480	0	0	0	0	0	1	0	0	0	0	0	0	0	0	0	k__Fungi;p__Ascomycota;c__Saccharomycetes;o__Saccharomycetales;f__Debaryomycetaceae;g__Candida;s__Candida_parapsilosis
NCBI5481	3	0	0	2	0	0	0	3	0	0	0	1	0	0	0	k__Fungi;p__Ascomycota;c__Saccharomycetes;o__Saccharomycetales;f__;g__Diutina;s__Diutina_rugosa
NCBI5482	0	0	0	0	0	2	0	0	0	0	0	1	0	0	0	k__Fungi;p__Ascomycota;c__Saccharomycetes;o__Saccharomycetales;f__Debaryomycetaceae;g__Candida;s__Candida_tropicalis
NCBI549	1	2	57	1	12	11	0	2	0	0	9	0	0	0	0	k__Bacteria;p__Pseudomonadota;c__Gammaproteobacteria;o__Enterobacterales;f__Erwiniaceae;g__Pantoea;s__Pantoea_agglomerans
NCBI550	90	85	43	415	909	1718	208	19	50	7	11	6	26	24	34	k__Bacteria;p__Pseudomonadota;c__Gammaproteobacteria;o__Enterobacterales;f__Enterobacteriaceae;g__Enterobacter;s__Enterobacter_cloacae
NCBI550542	0	0	0	0	0	0	0	0	0	0	0	5	0	0	0	k__Bacteria;p__Bacillota;c__Bacilli;o__Bacillales;f__Bacillaceae;g__Geobacillus;s__Geobacillus_sp._Y412MC52
NCBI5507	0	0	0	0	0	0	0	0	0	0	0	1	0	0	0	k__Fungi;p__Ascomycota;c__Sordariomycetes;o__Hypocreales;f__Nectriaceae;g__Fusarium;s__Fusarium_oxysporum
NCBI551760	0	2	1	0	1	14	1	0	1	0	0	1	0	0	0	k__Bacteria;p__Pseudomonadota;c__Betaproteobacteria;o__Rhodocyclales;f__Rhodocyclaceae;g__Aromatoleum;s__Aromatoleum_aromaticum
NCBI5518	0	0	0	3	0	0	0	0	7	0	0	0	0	0	0	k__Fungi;p__Ascomycota;c__Sordariomycetes;o__Hypocreales;f__Nectriaceae;g__Fusarium;s__Fusarium_graminearum
NCBI55194	0	0	0	0	0	4	0	0	0	0	0	0	0	0	0	k__Fungi;p__Basidiomycota;c__Malasseziomycetes;o__Malasseziales;f__Malasseziaceae;g__Malassezia;s__Mal

															assezia_furfur
NCBI552	0	0	0	0	1	0	0	0	0	0	0	0	0	0	0	k__Bacteria;p__Pseudomonadota;c__Gammaproteobacteria;o__Enterobacterales;f__Erwiniaceae;g__Erwinia;s__Erwinia_amylovora
NCBI55211	0	0	0	0	1	0	0	0	0	0	0	0	0	3	0	k__Bacteria;p__Pseudomonadota;c__Gammaproteobacteria;o__Enterobacterales;f__Erwiniaceae;g__Erwinia;s__Erwinia_persicina
NCBI55212	0	0	0	3	3	0	0	0	0	0	0	0	0	0	0	k__Bacteria;p__Pseudomonadota;c__Gammaproteobacteria;o__Enterobacterales;f__Erwiniaceae;g__Erwinia;s__Erwinia_rhapontici
NCBI553	216	233	385	1450	3180	5514	461	85	226	24	30	47	94	76	65	k__Bacteria;p__Pseudomonadota;c__Gammaproteobacteria;o__Enterobacterales;f__Erwiniaceae;g__Pantoea;s__Pantoea_ananatis
NCBI5530	15	22	3	42	65	159	58	3	10	0	0	10	23	17	0	k__Fungi;p__Ascomycota;c__Sordariomycetes;o__Hypocreales;f__Clavicipitaceae;g__Metarhizium;s__Metarhizium_anisopliae
NCBI5535	0	0	0	0	13	34	0	0	0	0	0	0	0	0	0	k__Fungi;p__Basidiomycota;c__Microbotryomycetes;o__Sporidiobolales;f__Sporidiobolaceae;g__Rhodotorula;s__Rhodotorula_glutinis
NCBI5537	0	0	0	0	0	0	0	0	0	0	0	36	0	0	0	k__Fungi;p__Basidiomycota;c__Microbotryomycetes;o__Sporidiobolales;f__Sporidiobolaceae;g__Rhodotorula;s__Rhodotorula_mucilaginosa
NCBI553814	6	28	3	1	13	314	22	2	3	0	0	0	0	0	3	k__Bacteria;p__Pseudomonadota;c__Betaproteobacteria;o__Burkholderiales;f__Comamonadaceae;g__Acidovorax;s__Acidovorax_carolinensis
NCBI554344	0	0	0	0	0	23	0	0	0	0	0	0	0	0	0	k__Bacteria;p__Pseudomonadota;c__Gammaproteobacteria;o__Pseudomonadales;f__Pseudomonadaceae;g__Pseudomonas;s__Pseudomonas_toyotomiensis
NCBI5544	0	0	0	0	0	2	0	0	0	0	0	0	0	0	0	k__Fungi;p__Ascomycota;c__Sordariomycetes;o__Hypocreales;f__Hypocreaceae;g__Trichoderma;s__Trichoder

																ma_harzianum
NCBI55518	0	0	0	0	1	6	1	0	0	0	0	0	0	0	0	k__Bacteria;p__Pseudomonadota;c__Alphaproteobacteria;o__Rhodospirillales;f__Rhodospirillaceae;g__Magnetospirillum;s__Magnetospirillum_gryphiswaldense
NCBI55601	2035	1948	1775	8591	17186	27240	2580	532	1464	271	299	589	398	377	383	k__Bacteria;p__Pseudomonadota;c__Gammaproteobacteria;o__Vibrionales;f__Vibrionaceae;g__Vibrio;s__Vibrio_anguillarum
NCBI556288	0	0	0	0	18	3	0	0	0	0	0	0	0	0	0	k__Bacteria;p__Actinomycetota;c__Actinomycetes;o__Micrococcales;f__Dermabacteraceae;g__Brachybacterium;s__Brachybacterium_saurashtrense
NCBI556325	0	0	0	0	7	0	0	0	0	0	0	13	0	0	0	k__Bacteria;p__Actinomycetota;c__Actinomycetes;o__Micrococcales;f__Micrococcaceae;g__Neomicrococcus;s__Neomicrococcus_aestuarii
NCBI556499	0	0	0	1	0	1	0	0	0	0	0	0	0	0	0	k__Bacteria;p__Actinomycetota;c__Actinomycetes;o__Propionibacteriales;f__Propionibacteriaceae;g__Propionibacterium;s__Propionibacterium_acidifaciens
NCBI5580	0	0	0	0	3	0	0	0	0	0	0	0	0	0	0	k__Fungi;p__Ascomycota;c__Dothideomycetes;o__Dothideales;f__Saccotheciaceae;g__Aureobasidium;s__Aureobasidium_pullulans
NCBI558537	169	184	66	327	1242	2893	247	0	111	28	0	20	41	0	0	k__Bacteria;p__Pseudomonadota;c__Betaproteobacteria;o__Burkholderiales;f__Comamonadaceae;g__Delftia;s__Delftia_lacustris
NCBI5599	0	30	8	7	32	122	0	0	0	0	11	7	0	0	0	k__Fungi;p__Ascomycota;c__Dothideomycetes;o__Pleosporales;f__Pleosporaceae;g__Alternaria;s__Alternaria_alternata
NCBI56	0	0	0	0	0	10	0	0	0	0	0	0	0	0	0	k__Bacteria;p__Myxococcota;c__Myxococcia;o__Polyangiales;f__Polyangiaceae;g__Sorangium;s__Sorangium_cellulosum
NCBI561133	0	0	0	0	0	11	0	0	0	0	0	0	0	0	0	k__Bacteria;p__Pseudomonadota;c__Alphaproteobacteria;o__Caulobacterales;f__Caulobacteraceae;g__Brevundi

																monas;s__Brevundimonas_sp._Gc-2-c
NCBI56193	8	8	11	0	12	102	0	0	0	0	0	0	0	0	5	k__Bacteria;p__Pseudomonadota;c__Alphaproteobacteria;o__Sphingomonadales;f__Sphingomonadaceae;g__Sphingobium;s__Sphingobium_chungbukense
NCBI562	105	61	117	272	76	4546	225	75	0	0	54	22214	32	0	37	k__Bacteria;p__Pseudomonadota;c__Gammaproteobacteria;o__Enterobacterales;f__Enterobacteriaceae;g__Escherichia;s__Escherichia_coli
NCBI56361	0	0	0	0	0	8	0	0	0	0	0	0	0	0	0	k__Bacteria;p__Pseudomonadota;c__Alphaproteobacteria;o__Sphingomonadales;f__Sphingomonadaceae;g__Sphingomonas;s__Sphingomonas_ursincola
NCBI564	0	0	0	0	0	0	0	0	0	0	0	2	0	0	0	k__Bacteria;p__Pseudomonadota;c__Gammaproteobacteria;o__Enterobacterales;f__Enterobacteriaceae;g__Escherichia;s__Escherichia_fergusonii
NCBI56448	0	0	0	0	0	8	0	0	0	0	0	0	0	0	0	k__Bacteria;p__Pseudomonadota;c__Gammaproteobacteria;o__Xanthomonadales;f__Xanthomonadaceae;g__Xanthomonas;s__Xanthomonas_arboricola
NCBI56455	0	0	0	0	0	1	0	0	0	0	0	0	0	0	0	k__Bacteria;p__Pseudomonadota;c__Gammaproteobacteria;o__Xanthomonadales;f__Xanthomonadaceae;g__Xanthomonas;s__Xanthomonas_hyacinthi
NCBI56458	5	8	8	40	127	152	12	0	1	0	0	1	1	5	0	k__Bacteria;p__Pseudomonadota;c__Gammaproteobacteria;o__Xanthomonadales;f__Xanthomonadaceae;g__Xanthomonas;s__Xanthomonas_sacchari
NCBI56464	0	0	0	1	1	8	0	0	0	0	0	0	0	0	0	k__Bacteria;p__Pseudomonadota;c__Gammaproteobacteria;o__Xanthomonadales;f__Xanthomonadaceae;g__Xanthomonas;s__Xanthomonas_theicola
NCBI565	245	285	263	1290	2873	4710	429	100	289	39	38	46	151	94	80	k__Bacteria;p__Pseudomonadota;c__Gammaproteobacteria;o__Enterobacterales;f__Enterobacteriaceae;g__Atlantibacter;s__Atlantibacter_hermannii
NCBI5	3	0	6	14	17	30	0	0	1	2	0	1	0	0	1	k__Bacteria;p__Pseudomonadota;c__Gammaproteobacte

66																ria;o__Enterobacterales;f__Enterobacteriaceae;g__Pseudescherichia;s__Pseudescherichia_vulneris
NCBI56730	0	0	0	0	1	2	0	0	0	0	0	0	0	0	0	k__Bacteria;p__Pseudomonadota;c__Alphaproteobacteria;o__Hyphomicrobiales;f__Rhizobiaceae;g__Rhizobium;s__Rhizobium_gallicum
NCBI56765	0	0	0	0	0	15	0	0	0	0	0	0	0	0	0	k__Bacteria;p__;c__;o__;f__;g__;s__uncultured_marine_bacterium
NCBI57037	1525	1473	1594	2192	2048	495	2192	1917	2162	2085	1733	2555	845	754	841	k__Bacteria;p__Bacillota;c__Bacilli;o__Lactobacillales;f__Lactobacillaceae;g__Lacticaseibacillus;s__Lacticaseibacillus_zeae
NCBI570505	0	2	4	1	4	7	1	0	0	0	0	0	0	0	0	k__Bacteria;p__Pseudomonadota;c__Alphaproteobacteria;o__Hyphomicrobiales;f__Methylobacteriaceae;g__Methylobacterium;s__Methylobacterium_bullatum
NCBI571	10	30	16	110	169	860	29	14	45	0	0	14	25	0	13	k__Bacteria;p__Pseudomonadota;c__Gammaproteobacteria;o__Enterobacterales;f__Enterobacteriaceae;g__Klebsiella;s__Klebsiella_oxytoca
NCBI571800	0	0	0	0	1	0	0	0	0	0	0	0	0	0	0	k__Bacteria;p__Pseudomonadota;c__Gammaproteobacteria;o__Moraxellales;f__Moraxellaceae;g__Psychrobacter;s__Psychrobacter_sp._G
NCBI571913	0	0	0	0	0	2	0	0	0	0	0	0	0	0	0	k__Bacteria;p__Actinomycetota;c__Actinomycetes;o__Micrococcales;f__Dermacoccaceae;g__Luteipulveratus;s__Luteipulveratus_mongoliensis
NCBI573	13	30	58	206	360	3108	85	0	0	23	0	1343	19	0	0	k__Bacteria;p__Pseudomonadota;c__Gammaproteobacteria;o__Enterobacterales;f__Enterobacteriaceae;g__Klebsiella;s__Klebsiella_pneumoniae
NCBI573591	1	0	0	0	0	0	0	0	0	0	0	0	0	0	0	k__Heunggongvirae;p__Uroviricota;c__Caudoviricetes;o__;f__;g__;s__Stenotrophomonas_phage_S1
NCBI57480	0	0	0	0	0	1	0	0	0	0	0	0	0	0	0	k__Bacteria;p__Pseudomonadota;c__Betaproteobacteria;o__Neisseriales;f__Chromobacteriaceae;g__Microvirgula;s__Microvirgula_aerodenitrificans

NCBI57498	0	0	0	0	0	4	0	0	0	0	0	0	0	0	0	k__Bacteria;p__Deinococcota;c__Deinococci;o__Deinococcales;f__Deinococcaceae;g__Deinococcus;s__Deinococcus_grandis
NCBI574987	7674	7482	7843	21365	21072	15915	12327	7959	11783	54351	48812	76913	79697	83340	96780	k__Bacteria;p__Pseudomonadota;c__Alphaproteobacteria;o__Rhodospirillales;f__Acetobacteraceae;g__Gluconobacter;s__Gluconobacter_sphaericus
NCBI575200	0	0	0	0	1	2	0	0	0	0	1	0	0	0	0	k__Bacteria;p__Actinomycetota;c__Actinomycetes;o__Mycobacteriales;f__Corynebacteriaceae;g__Corynebacterium;s__Corynebacterium_maris
NCBI576784	0	0	0	0	0	0	0	0	0	0	0	15	0	0	0	k__Bacteria;p__Actinomycetota;c__Actinomycetes;o__Kitasatosporales;f__Streptomycetaceae;g__Streptomyces;s__Streptomyces_iranensis
NCBI577	0	0	1	7	3	2	0	0	0	0	0	0	0	0	0	k__Bacteria;p__Pseudomonadota;c__Gammaproteobacteria;o__Enterobacterales;f__Enterobacteriaceae;g__Raoultella;s__Raoultella_terrigena
NCBI57704	0	0	0	0	0	3	0	0	0	0	0	0	0	0	0	k__Bacteria;p__Actinomycetota;c__Actinomycetes;o__Mycobacteriales;f__Tsukamurellaceae;g__Tsukamurella;s__Tsukamurella_tyrosinosolvens
NCBI57706	0	0	0	0	74	0	0	0	0	0	0	0	0	0	0	k__Bacteria;p__Pseudomonadota;c__Gammaproteobacteria;o__Enterobacterales;f__Enterobacteriaceae;g__Citrobacter;s__Citrobacter_braakii
NCBI57975	1	0	0	0	0	0	0	0	0	0	0	0	0	0	0	k__Bacteria;p__Pseudomonadota;c__Betaproteobacteria;o__Burkholderiales;f__Burkholderiaceae;g__Burkholderia;s__Burkholderia_thailandensis
NCBI580165	0	0	0	1	0	0	0	0	0	0	0	0	0	0	0	k__Bacteria;p__Bacillota;c__Bacilli;o__Bacillales;f__Bacillaceae;g__Bacillus;s__Bacillus_cytotoxicus
NCBI58169	6	13	37	110	144	380	8	21	48	0	6	6	15	9	14	k__Bacteria;p__Pseudomonadota;c__Gammaproteobacteria;o__Enterobacterales;f__Yersiniaceae;g__Rahnella;s__Rahnella_inusitata
NCBI5	289	292	254	119	267	388	455	83	192	13	53	85	83	107	98	k__Bacteria;p__Pseudomonadota;c__Gammaproteobacte

82				1	0	0										ria;o__Enterobacterales;f__Morganellaceae;g__Morganella;s__Morganella_morganii
NCBI584	0	1	3	2	20	41	0	0	0	0	0	5	0	0	0	k__Bacteria;p__Pseudomonadota;c__Gammaproteobacteria;o__Enterobacterales;f__Morganellaceae;g__Proteus;s__Proteus_mirabilis
NCBI585	0	0	0	0	2	0	0	0	0	0	0	0	0	0	0	k__Bacteria;p__Pseudomonadota;c__Gammaproteobacteria;o__Enterobacterales;f__Morganellaceae;g__Proteus;s__Proteus_vulgaris
NCBI586239	4	2	4	9	18	3	0	1	0	27	11	33	38	13	51	k__Bacteria;p__Pseudomonadota;c__Alphaproteobacteria;o__Rhodospirillales;f__Acetobacteraceae;g__Gluconobacter;s__Gluconobacter_roseus
NCBI58627	48	32	4	8	28	6	9	34	8	86	56	23	10	18	11	k__Fungi;p__Ascomycota;c__Saccharomycetes;o__Saccharomycetales;f__Debaryomycetaceae;g__Debaryomyces;s__Debaryomyces_fabryi
NCBI587	5	0	0	16	11	88	24	0	0	0	0	0	0	0	0	k__Bacteria;p__Pseudomonadota;c__Gammaproteobacteria;o__Enterobacterales;f__Morganellaceae;g__Providencia;s__Providencia_rettgeri
NCBI587753	0	0	0	0	25	21	11	0	0	0	0	0	0	0	0	k__Bacteria;p__Pseudomonadota;c__Gammaproteobacteria;o__Pseudomonadales;f__Pseudomonadaceae;g__Pseudomonas;s__Pseudomonas_chlororaphis
NCBI588	0	0	0	0	39	45	0	0	16	2	0	1053	0	0	0	k__Bacteria;p__Pseudomonadota;c__Gammaproteobacteria;o__Enterobacterales;f__Morganellaceae;g__Providencia;s__Providencia_stuartii
NCBI58853	1	0	0	0	0	1	0	0	0	0	0	0	0	0	0	k__Fungi;p__Ascomycota;c__Sordariomycetes;o__Hypocreales;f__Hypocreaceae;g__Trichoderma;s__Trichoderma_citrinoviride
NCBI588596	0	0	0	0	0	0	0	0	0	0	1	0	0	0	0	k__Fungi;p__Mucoromycota;c__Glomeromycetes;o__Glomerales;f__Glomeraceae;g__Rhizophagus;s__Rhizophagus_irregularis

NCBI588726	0	0	0	0	1	0	0	0	0	0	0	0	0	0	0	k__Fungi;p__Ascomycota;c__Saccharomycetes;o__Saccharomycetales;f__Saccharomycetaceae;g__Kazachstania;s__Kazachstania_naganishii
NCBI588898	0	0	0	5	0	2	0	3	0	0	0	0	0	0	0	k__Archaea;p__Euryarchaeota;c__Halobacteria;o__Natrialbales;f__Natrialbaceae;g__Haloterrigena;s__Haloterrigena_daqingensis
NCBI588932	2	3	4	19	40	77	2	2	2	1	0	1	2	0	1	k__Bacteria;p__Pseudomonadota;c__Alphaproteobacteria;o__Caulobacterales;f__Caulobacteraceae;g__Brevundimonas;s__Brevundimonas_naejangsanensis
NCBI592375	715	856	624	958	1517	3309	994	992	928	861	768	849	411	582	282	k__Bacteria;p__Bacillota;c__Bacilli;o__Bacillales;f__Listeriaceae;g__Listeria;s__uncultured_Listeria_sp.
NCBI59405	0	0	0	0	0	96	0	0	0	10	5	11	0	0	0	k__Bacteria;p__Pseudomonadota;c__Betaproteobacteria;o__Rhodocyclales;f__Zoogloeaceae;g__Thauera;s__Thauera_aromatica
NCBI59505	0	0	0	0	0	1	0	0	0	0	0	0	0	0	0	k__Bacteria;p__Actinomycetota;c__Actinomycetes;o__Actinomycetales;f__Actinomycetaceae;g__Actinotignum;s__Actinotignum_schaalii
NCBI59620	46	58	46	85	188	314	47	21	70	25	20	44	27	32	21	k__Bacteria;p__Bacillota;c__Clostridia;o__Eubacteriales;f__Clostridiaceae;g__Clostridium;s__uncultured_Clostridium_sp.
NCBI59748	6	1	8	7	24	41	17	1	9	0	2	1	0	2	2	k__Bacteria;p__Mycoplasmatota;c__Mollicutes;o__Acholeplasmatales;f__Acholeplasmataceae;g__Candidatus_Phytoplasma;s__Candidatus_Phytoplasma_australiense
NCBI59779	2	3	0	30	29	57	3	0	0	1	0	12	3	13	0	k__Bacteria;p__Pseudomonadota;c__Alphaproteobacteria;o__Rhodobacterales;f__Paracoccaceae;g__Paracoccus;s__Paracoccus_marcusii
NCBI59803	0	0	0	0	0	63	0	0	0	0	0	0	8	10	0	k__Bacteria;p__Pseudomonadota;c__Alphaproteobacteria;o__Sphingomonadales;f__Sphingomonadaceae;g__Sphingomonas;s__Sphingomonas_echinoides
NCBI5	0	1	6	9	28	103	2	0	0	0	0	1	0	6	0	k__Bacteria;p__Pseudomonadota;c__Gammaproteobacte

9814																ria;o__Enterobacterales;f__Erwiniaceae;g__Pantoea;s__Pantoea_dispersa
NCBI60137	0	0	0	0	0	0	0	0	0	0	0	2	0	0	0	k__Bacteria;p__Pseudomonadota;c__Alphaproteobacteria;o__Rhodobacterales;f__Roseobacteraceae;g__Sulfitobacter;s__Sulfitobacter_pontiacus
NCBI60172	0	0	0	1	3	2	0	0	3	0	0	0	0	0	0	k__Fungi;p__Ascomycota;c__Eurotiomycetes;o__Eurotiales;f__Aspergillaceae;g__Penicillium;s__Penicillium_solitum
NCBI604330	0	0	0	0	0	6	0	0	0	0	0	1	0	0	0	k__Bacteria;p__Actinomycetota;c__Coriobacteriia;o__Coriobacteriales;f__Atopobiaceae;g__Parafannyhessea;s__Parafannyhessea_umbonata
NCBI60520	41	32	27	79	73	25	38	28	44	64	49	73	45	31	57	k__Bacteria;p__Bacillota;c__Bacilli;o__Lactobacillales;f__Lactobacillaceae;g__Lactiplantibacillus;s__Lactiplantibacillus_paraplantarum
NCBI60552	0	0	0	0	20	0	0	0	0	0	0	0	0	0	0	k__Bacteria;p__Pseudomonadota;c__Betaproteobacteria;o__Burkholderiales;f__Burkholderiaceae;g__Burkholderia;s__Burkholderia_vietnamiensis
NCBI60920	0	0	0	2	0	0	0	0	0	0	0	0	0	0	0	k__Bacteria;p__Actinomycetota;c__Actinomycetes;o__Micrococcales;f__Sanguibacteraceae;g__Sanguibacter;s__Sanguibacter_keddieii
NCBI614	0	0	0	0	6	3	0	0	0	0	0	0	0	0	0	k__Bacteria;p__Pseudomonadota;c__Gammaproteobacteria;o__Enterobacterales;f__Yersiniaceae;g__Serratia;s__Serratia_liquefaciens
NCBI614068	0	0	0	0	0	472	0	0	0	0	0	0	0	0	0	k__Bacteria;p__Actinomycetota;c__Actinomycetes;o__Micrococcales;f__Microbacteriaceae;g__Microbacterium;s__Microbacterium_sp._MA1
NCBI615	6	0	0	9	9	255	15	0	3	0	134	148	0	0	0	k__Bacteria;p__Pseudomonadota;c__Gammaproteobacteria;o__Enterobacterales;f__Yersiniaceae;g__Serratia;s__Serratia_marcescens
NCBI6	0	0	0	6	0	0	0	0	0	0	0	14	0	0	0	k__Bacteria;p__Pseudomonadota;c__Gammaproteobacte

1645																ria;o__Enterobacterales;f__Enterobacteriaceae;g__Enterobacter;s__Enterobacter_asburiae
NCBI61646	0	0	0	3	0	0	4	0	0	0	0	0	0	0	0	k__Bacteria;p__Pseudomonadota;c__Gammaproteobacteria;o__Enterobacterales;f__Enterobacteriaceae;g__Lelliottia;s__Lelliottia_amnigena
NCBI61648	0	0	0	0	0	0	0	0	1	0	0	0	0	0	0	k__Bacteria;p__Pseudomonadota;c__Gammaproteobacteria;o__Enterobacterales;f__Enterobacteriaceae;g__Kluyvera;s__Kluyvera_intermedia
NCBI617123	0	0	0	0	0	0	0	0	0	0	0	2	0	0	0	k__Bacteria;p__Bacillota;c__Clostridia;o__Eubacteriales;f__Lachnospiraceae;g__Lachnoanaerobaculum;s__Lachnoanaerobaculum_umeaense
NCBI618	0	0	0	1	2	0	0	0	0	0	0	0	0	0	0	k__Bacteria;p__Pseudomonadota;c__Gammaproteobacteria;o__Enterobacterales;f__Yersiniaceae;g__Serratia;s__Serratia_odorifera
NCBI621376	0	0	0	0	0	0	0	0	0	0	0	1	0	0	0	k__Bacteria;p__Pseudomonadota;c__Gammaproteobacteria;o__Alteromonadales;f__Pseudoalteromonadaceae;g__Pseudoalteromonas;s__Pseudoalteromonas_donghaensis
NCBI626932	0	2	0	0	0	7	0	0	0	0	0	2	0	0	0	k__Bacteria;p__Bacteroidota;c__Bacteroidia;o__Bacteroidales;f__Rikenellaceae;g__Alistipes;s__Alistipes_indistinctus
NCBI627192	0	2	0	0	0	10	0	0	0	0	0	0	0	0	0	k__Bacteria;p__Pseudomonadota;c__Alphaproteobacteria;o__Sphingomonadales;f__Sphingomonadaceae;g__Sphingobium;s__Sphingobium_sp._SYK-6
NCBI63	0	0	1	0	1	29	0	0	0	0	0	0	0	0	0	k__Bacteria;p__Pseudomonadota;c__Betaproteobacteria;o__Neisseriales;f__Neisseriaceae;g__Vitreoscilla;s__Vitreoscilla_filiformis
NCBI630	3713	1959	5633	146	10755	22	347	20	289	24	158	0	8	244	3	k__Bacteria;p__Pseudomonadota;c__Gammaproteobacteria;o__Enterobacterales;f__Yersiniaceae;g__Yersinia;s__Yersinia_enterocolitica

NCBI632112	0	0	0	0	0	1	0	0	3	0	1	1	0	0	2	k__Heunggongvirae;p__Uroviricota;c__Caudoviricetes;o__;f__Herelleviridae;g__Mooreparkvirus;s__Mooreparkvirus_Lb3381
NCBI633	0	0	0	0	0	2	3	0	0	0	0	0	0	0	0	k__Bacteria;p__Pseudomonadota;c__Gammaproteobacteria;o__Enterobacterales;f__Yersiniaceae;g__Yersinia;s__Yersinia_pseudotuberculosis
NCBI633415	0	0	0	0	0	10	0	0	0	0	0	0	0	0	0	k__Bacteria;p__Pseudomonadota;c__Gammaproteobacteria;o__Aeromonadales;f__Aeromonadaceae;g__Aeromonas;s__Aeromonas_sanarellii
NCBI633417	0	0	0	0	12	0	0	0	0	0	0	0	0	0	0	k__Bacteria;p__Pseudomonadota;c__Gammaproteobacteria;o__Aeromonadales;f__Aeromonadaceae;g__Aeromonas;s__Aeromonas_taiwanensis
NCBI63577	0	0	0	0	0	0	0	0	0	0	0	0	0	0	1	k__Fungi;p__Ascomycota;c__Sordariomycetes;o__Hypocreales;f__Hypocreaceae;g__Trichoderma;s__Trichoderma_atroviride
NCBI636	0	0	0	0	0	0	0	0	0	0	0	1	0	0	0	k__Bacteria;p__Pseudomonadota;c__Gammaproteobacteria;o__Enterobacterales;f__Hafniaceae;g__Edwardsiella;s__Edwardsiella_tarda
NCBI641107	0	0	1	0	1	0	0	0	0	0	0	0	0	0	0	k__Bacteria;p__Bacillota;c__Clostridia;o__Eubacteriales;f__Clostridiaceae;g__Clostridium;s__Clostridium_sp._DL-VIII
NCBI641148	0	0	0	1	9	3	0	0	0	0	0	2	0	0	0	k__Bacteria;p__Pseudomonadota;c__Betaproteobacteria;o__Neisseriales;f__Neisseriaceae;g__Neisseria;s__Neisseria_sp._oral_taxon_014
NCBI644	3	23	5	0	29	55	39	0	8	0	0	81	0	0	0	k__Bacteria;p__Pseudomonadota;c__Gammaproteobacteria;o__Aeromonadales;f__Aeromonadaceae;g__Aeromonas;s__Aeromonas_hydrophila
NCBI645	0	0	0	0	3	0	0	0	0	0	0	0	0	0	0	k__Bacteria;p__Pseudomonadota;c__Gammaproteobacteria;o__Aeromonadales;f__Aeromonadaceae;g__Aeromonas;s__Aeromonas_salmonicida

NCBI646	13	0	0	0	0	0	0	0	0	0	0	0	0	0	0	k__Bacteria;p__Pseudomonadota;c__Gammaproteobacteria;o__Aeromonadales;f__Aeromonadaceae;g__Aeromonas;s__Aeromonas_sobria
NCBI648	0	0	37	27	0	92	20	0	0	0	0	1311	0	0	0	k__Bacteria;p__Pseudomonadota;c__Gammaproteobacteria;o__Aeromonadales;f__Aeromonadaceae;g__Aeromonas;s__Aeromonas_caviae
NCBI648995	464	332	304	194	1372	14352	414	93	156	115	34	325	40	30	55	k__Bacteria;p__Pseudomonadota;c__Alphaproteobacteria;o__Hyphomicrobiales;f__Rhizobiaceae;g__Rhizobium;s__Rhizobium_pusense
NCBI649739	0	0	0	1	0	0	0	0	0	0	0	0	0	0	0	k__Bacteria;p__Actinomycetota;c__Actinomycetes;o__Actinomycetales;f__Actinomycetaceae;g__Actinomyces;s__Actinomyces_sp._oral_taxon_848
NCBI649756	0	1	15	10	0	36	0	1	1	0	1	74	0	0	0	k__Bacteria;p__Bacillota;c__Clostridia;o__Eubacteriales;f__Lachnospiraceae;g__Anaerostipes;s__Anaerostipes_hadrus
NCBI651	0	0	0	0	0	11	15	0	0	0	0	5	0	0	0	k__Bacteria;p__Pseudomonadota;c__Gammaproteobacteria;o__Aeromonadales;f__Aeromonadaceae;g__Aeromonas;s__Aeromonas_media
NCBI651561	0	5	0	0	0	0	0	0	0	0	0	0	0	0	0	k__Bacteria;p__Bacteroidota;c__Flavobacteriia;o__Flavobacteriales;f__Weeksellaceae;g__Chryseobacterium;s__Chryseobacterium_arthrosphaerae
NCBI651822	0	0	0	0	2	0	0	0	0	0	0	0	0	0	0	k__Bacteria;p__Synergistota;c__Synergistia;o__Synergistales;f__Aminobacteriaceae;g__Fretibacterium;s__Fretibacterium_fastidiosum
NCBI652716	0	0	0	0	0	1	0	0	0	0	0	14	0	0	0	k__Bacteria;p__Bacteroidota;c__Bacteroidia;o__Bacteroidales;f__Prevotellaceae;g__Prevotella;s__Prevotella_sp._oral_taxon_299
NCBI652764	0	0	0	0	0	4	0	0	0	0	0	0	0	0	0	k__Bacteria;p__Pseudomonadota;c__Alphaproteobacteria;o__Rhodospirillales;f__Azospirillaceae;g__Azospirillum;s__Azospirillum_sp._TSH100

NCBI653931	0	0	0	0	0	1	0	0	0	0	0	0	0	0	0	k__Bacteria;p__Pseudomonadota;c__Alphaproteobacteria;o__Sphingomonadales;f__Sphingomonadaceae;g__Sphingomonas;s__Sphingomonas_alpina
NCBI654	0	0	0	0	6	26	0	8	0	0	2	123	0	0	0	k__Bacteria;p__Pseudomonadota;c__Gammaproteobacteria;o__Aeromonadales;f__Aeromonadaceae;g__Aeromonas;s__Aeromonas_veronii
NCBI65553	1	0	0	0	0	0	0	0	0	0	0	0	0	0	0	k__Bacteria;p__Campylobacterota;c__Epsilonproteobacteria;o__Campylobacterales;f__Sulfurospirillaceae;g__Sulfurospirillum;s__Sulfurospirillum_deleyianum
NCBI65656	1	3	0	0	2	18	1	0	0	0	0	0	0	0	1	k__Bacteria;p__Pseudomonadota;c__Betaproteobacteria;o__Burkholderiales;f__Comamonadaceae;g__Hydrogenophaga;s__Hydrogenophaga_taeniospiralis
NCBI657323	0	0	0	0	0	74	0	0	0	0	0	3	0	0	0	k__Bacteria;p__Bacillota;c__Clostridia;o__Eubacteriales;f__Oscillospiraceae;g__Ruminococcus;s__Ruminococcus_sp._SR1/5
NCBI65741	0	0	0	0	0	3	0	0	0	0	0	0	0	0	0	k__Bacteria;p__Pseudomonadota;c__Gammaproteobacteria;o__Pseudomonadales;f__Pseudomonadaceae;g__Pseudomonas;s__Pseudomonas_knackmussii
NCBI658630	0	0	0	0	0	1	0	0	0	0	0	0	0	0	0	k__Bacteria;p__Pseudomonadota;c__Gammaproteobacteria;o__Pseudomonadales;f__Pseudomonadaceae;g__Pseudomonas;s__Pseudomonas_sp._CMR5c
NCBI65958	571	543	649	10022	14038	10855	914	621	937	508	531	722	963	924	1152	k__Bacteria;p__Pseudomonadota;c__Alphaproteobacteria;o__Rhodospirillales;f__Acetobacteraceae;g__Komagataeibacter;s__Komagataeibacter_oboediens
NCBI65959	13190	11785	15187	45011	36180	26006	14639	11475	14906	47766	47336	70399	404758	389321	461243	k__Bacteria;p__Pseudomonadota;c__Alphaproteobacteria;o__Rhodospirillales;f__Acetobacteraceae;g__Acetobacter;s__Acetobacter_pomorum
NCBI66229	0	0	0	9	34	0	0	0	0	0	0	0	0	0	10	k__Bacteria;p__Pseudomonadota;c__Alphaproteobacteria;o__Rhodospirillales;f__Acetobacteraceae;g__Komagataeibacter;s__Komagataeibacter_intermedius

NCBI662548	0	0	0	0	0	5	0	0	0	0	0	0	0	0	0	k__Bacteria;p__Pseudomonadota;c__Betaproteobacteria;o__Burkholderiales;f__Comamonadaceae;g__Variovorax;s__Variovorax_sp._RA8
NCBI66269	0	0	2	1	0	1	0	0	0	0	0	0	0	0	0	k__Bacteria;p__Pseudomonadota;c__Gammaproteobacteria;o__Enterobacterales;f__Erwiniaceae;g__Pantoea;s__Pantoea_stewartii
NCBI663	2	0	0	0	0	6	1	0	0	0	0	119	0	0	0	k__Bacteria;p__Pseudomonadota;c__Gammaproteobacteria;o__Vibrionales;f__Vibrionaceae;g__Vibrio;s__Vibrio_alginolyticus
NCBI664683	0	0	0	0	0	4	0	0	0	0	0	0	0	0	0	k__Bacteria;p__Pseudomonadota;c__Gammaproteobacteria;o__Oceanospirillales;f__Halomonadaceae;g__Halomonas;s__Halomonas_titanicae
NCBI665099	0	0	0	3	0	0	0	0	0	0	0	0	0	0	0	k__Bacteria;p__Bacillota;c__Bacilli;o__Bacillales;f__Bacillaceae;g__Cytobacillus;s__Cytobacillus_oceanisediminis
NCBI665913	0	0	0	0	0	12	0	0	0	0	0	0	0	0	0	k__Bacteria;p__Pseudomonadota;c__Gammaproteobacteria;o__Enterobacterales;f__Erwiniaceae;g__Mixta;s__Mixta_calida
NCBI665914	0	0	16	1	11	3	0	0	0	0	1	0	0	0	0	k__Bacteria;p__Pseudomonadota;c__Gammaproteobacteria;o__Enterobacterales;f__Erwiniaceae;g__Mixta;s__Mixta_gaviniae
NCBI667019	0	0	0	0	0	1	0	0	0	0	0	0	0	0	0	k__Bacteria;p__Pseudomonadota;c__Betaproteobacteria;o__Burkholderiales;f__Comamonadaceae;g__Curvibacter;s__Curvibacter_putative_symbiont_of_Hydra_magnipapillata
NCBI669	0	0	0	0	0	2	0	1	0	0	0	59	0	0	3	k__Bacteria;p__Pseudomonadota;c__Gammaproteobacteria;o__Vibrionales;f__Vibrionaceae;g__Vibrio;s__Vibrio_harveyi
NCBI670	0	2	12	0	0	69	1	11	0	0	5	246	1	0	0	k__Bacteria;p__Pseudomonadota;c__Gammaproteobacteria;o__Vibrionales;f__Vibrionaceae;g__Vibrio;s__Vibrio

															_parahaemolyticus
NCBI672	0	0	0	0	0	0	0	0	0	0	0	1	0	0	0	k__Bacteria;p__Pseudomonadota;c__Gammaproteobacteria;o__Vibrionales;f__Vibrionaceae;g__Vibrio;s__Vibrio_vulnificus
NCBI67282	0	0	0	2	5	0	0	0	0	0	0	0	0	0	0	k__Bacteria;p__Actinomycetota;c__Actinomycetes;o__Kitasatosporales;f__Streptomycetaceae;g__Streptomyces;s__Streptomyces_calvus
NCBI674529	0	0	0	0	0	1	1	0	0	0	0	1	0	0	0	k__Bacteria;p__Bacteroidota;c__Bacteroidia;o__Bacteroidales;f__Bacteroidaceae;g__Bacteroides;s__Bacteroides_faecis
NCBI674703	0	0	0	0	0	2	0	0	0	0	0	0	0	0	1	k__Bacteria;p__Pseudomonadota;c__Alphaproteobacteria;o__Hyphomicrobiales;f__Hyphomicrobiaceae;g__Rhodoplanes;s__Rhodoplanes_sp._Z2-YC6860
NCBI675864	0	0	0	0	0	46	0	0	0	0	0	0	0	3	0	k__Bacteria;p__Actinomycetota;c__Actinomycetes;o__Propionibacteriales;f__Propionibacteriaceae;g__Auraticoccus;s__Auraticoccus_monumenti
NCBI67826	0	0	0	0	7	0	0	0	0	0	0	0	0	0	0	k__Bacteria;p__Pseudomonadota;c__Gammaproteobacteria;o__Enterobacterales;f__Enterobacteriaceae;g__Citrobacter;s__Citrobacter_sedlakii
NCBI680	0	0	0	0	0	0	0	0	0	0	0	5	0	0	0	k__Bacteria;p__Pseudomonadota;c__Gammaproteobacteria;o__Vibrionales;f__Vibrionaceae;g__Vibrio;s__Vibrio_campbellii
NCBI682749	0	0	0	0	0	0	1	0	0	0	0	0	0	0	0	k__Fungi;p__Chytridiomycota;c__Chytridiomycetes;o__Rhizophydiales;f__Rhizophydiaceae;g__;s__uncultured_Rhizophydium
NCBI684066	0	0	0	0	0	2	0	0	0	0	0	0	0	0	0	k__Bacteria;p__Bacillota;c__Bacilli;o__Lactobacillales;f__Streptococcaceae;g__Streptococcus;s__Streptococcus_lactarius
NCBI685565	0	0	0	0	0	0	1	0	0	0	0	0	0	0	0	k__Bacteria;p__Cyanobacteriota;c__Cyanophyceae;o__Pseudanabaenales;f__Pseudanabaenaceae;g__Pseudanabae

																na;s__Pseudanabaena_sp._ABRG5-3
NCBI686597	0	0	0	0	0	24	0	0	0	0	0	0	0	0	0	k__Bacteria;p__Pseudomonadota;c__Alphaproteobacteria;o__Hyphomicrobiales;f__Aurantimonadaceae;g__Martelella;s__Martelella_sp._AD-3
NCBI68825	0	0	0	0	1	1	1	0	0	0	0	0	0	0	0	k__Fungi;p__Ascomycota;c__Eurotiomycetes;o__Eurotiales;f__Trichocomaceae;g__Rasamsonia;s__Rasamsonia_emersonii
NCBI688394	0	0	0	0	0	1	0	0	0	0	0	0	0	0	0	k__Fungi;p__Mucoromycota;c__Mucoromycetes;o__Mucorales;f__Lichtheimiaceae;g__Lichtheimia;s__Lichtheimia_ramosa
NCBI68895	17	7	3	12	12	30	6	0	6	0	3	11	0	0	0	k__Bacteria;p__Pseudomonadota;c__Betaproteobacteria;o__Burkholderiales;f__Burkholderiaceae;g__Cupriavidus;s__Cupriavidus_basilensis
NCBI68909	0	0	0	1	2	8	0	2	0	0	0	2	0	0	0	k__Bacteria;p__Deinococcota;c__Deinococci;o__Deinococcales;f__Deinococcaceae;g__Deinococcus;s__Deinococcus_geothermalis
NCBI690256	0	0	0	0	0	3	0	0	0	0	0	4	0	0	0	k__Fungi;p__Ascomycota;c__Sordariomycetes;o__Glomerellales;f__Glomerellaceae;g__Colletotrichum;s__Colletotrichum_fructicola
NCBI691437	0	0	0	0	0	0	0	0	0	0	0	15	0	0	0	k__Bacteria;p__Bacillota;c__Bacilli;o__Bacillales;f__Bacillaceae;g__Geobacillus;s__Geobacillus_sp._C56-T3
NCBI69218	1	0	0	1	1	17	0	0	0	0	0	2	0	0	0	k__Bacteria;p__Pseudomonadota;c__Gammaproteobacteria;o__Enterobacterales;f__Enterobacteriaceae;g__Enterobacter;s__Enterobacter_cancerogenus
NCBI69328	0	0	0	0	0	10	0	0	0	0	0	0	0	0	0	k__Bacteria;p__Pseudomonadota;c__Gammaproteobacteria;o__Pseudomonadales;f__Pseudomonadaceae;g__Pseudomonas;s__Pseudomonas_sp._VLB120
NCBI69362	0	0	0	0	0	0	0	0	0	0	0	2	0	0	0	k__Bacteria;p__Actinomycetota;c__Actinomycetes;o__Micrococcales;f__Microbacteriaceae;g__Microbacterium

															;s__Microbacterium_schleiferi
NCBI69373	0	0	0	0	1	43	0	0	0	0	0	0	0	0	0	k__Bacteria;p__Actinomycetota;c__Actinomycetes;o__Micrococcales;f__Microbacteriaceae;g__Curtobacterium;s__Curtobacterium_pusillum
NCBI69395	0	0	0	0	0	3	0	0	0	0	0	0	0	0	0	k__Bacteria;p__Pseudomonadota;c__Alphaproteobacteria;o__Caulobacterales;f__Caulobacteraceae;g__Caulobacter;s__Caulobacter_henricii
NCBI696485	0	0	0	0	0	1	0	0	0	0	0	6	0	0	0	k__Bacteria;p__Pseudomonadota;c__Gammaproteobacteria;o__Vibrionales;f__Vibrionaceae;g__Vibrio;s__Vibrio_owensii
NCBI69665	115	173	82	39	369	4213	168	12	47	23	17	53	21	12	16	k__Bacteria;p__Pseudomonadota;c__Alphaproteobacteria;o__Caulobacterales;f__Caulobacteraceae;g__Caulobacter;s__Caulobacter_sp._FWC26
NCBI69781	2	1	1	0	8	115	5	4	0	0	1	1	0	0	0	k__Fungi;p__Ascomycota;c__Eurotiomycetes;o__Eurotiales;f__Aspergillaceae;g__Penicillium;s__Penicillium_oxalicum
NCBI69823	0	0	0	0	0	1	0	0	0	0	0	0	0	0	0	k__Bacteria;p__Bacillota;c__Negativicutes;o__Selenomonadales;f__Selenomonadaceae;g__Selenomonas;s__Selenomonas_sputigena
NCBI69964	0	0	0	0	0	1	0	0	0	0	0	0	0	0	0	k__Bacteria;p__Pseudomonadota;c__Gammaproteobacteria;o__Pseudomonadales;f__Pseudomonadaceae;g__Azotobacter;s__Azotobacter_salinestris
NCBI69966	0	0	1	0	0	0	0	0	0	0	0	0	0	0	0	k__Bacteria;p__Bacillota;c__Bacilli;o__Bacillales;f__Staphylococcaceae;g__Macrococcus;s__Macrococcus_caseolyticus
NCBI702113	0	0	9	0	35	213	10	0	0	0	0	7	0	0	0	k__Bacteria;p__Pseudomonadota;c__Alphaproteobacteria;o__Sphingomonadales;f__Sphingomonadaceae;g__Novosphingobium;s__Novosphingobium_sp._PP1Y
NCBI702967	0	0	0	0	0	2	0	0	0	0	0	0	0	0	0	k__Bacteria;p__Actinomycetota;c__Actinomycetes;o__Mycobacteriales;f__Corynebacteriaceae;g__Corynebacte

															rium;s__Corynebacterium_sp._NML98-0116
NCBI703	0	0	0	3	3	5	0	0	0	0	0	0	0	0	0	k__Bacteria;p__Pseudomonadota;c__Gammaproteobacteria;o__Enterobacterales;f__Enterobacteriaceae;g__Plesiomonas;s__Plesiomonas_shigelloides
NCBI703222	0	0	0	0	0	6	0	0	0	0	0	0	0	0	0	k__Bacteria;p__Actinomycetota;c__Actinomycetes;o__Pseudonocardiales;f__Pseudonocardiaceae;g__Kibdelosporangium;s__Kibdelosporangium_sp._MJ126-NF4
NCBI70346	0	2	6	0	7	11	0	0	0	0	0	0	0	0	6	k__Bacteria;p__Pseudomonadota;c__Gammaproteobacteria;o__Moraxellales;f__Moraxellaceae;g__Acinetobacter;s__Acinetobacter_variabilis
NCBI70348	8	13	5	2	28	104	19	0	0	5	1	9	7	2	1	k__Bacteria;p__Pseudomonadota;c__Gammaproteobacteria;o__Moraxellales;f__Moraxellaceae;g__Acinetobacter;s__Acinetobacter_dispersus
NCBI704125	1	0	0	2	0	0	0	0	0	0	0	0	0	0	0	k__Bacteria;p__Bacillota;c__Clostridia;o__Eubacteriales;f__Clostridiaceae;g__Clostridium;s__Clostridium_gelidum
NCBI70584	32	0	0	0	0	24	0	0	0	0	0	0	0	0	0	k__Bacteria;p__Pseudomonadota;c__Betaproteobacteria;o__Burkholderiales;f__;g__Aquabacterium;s__Aquabacterium_parvum
NCBI70586	13	0	0	0	18	271	0	0	0	0	0	0	0	0	0	k__Bacteria;p__Pseudomonadota;c__Betaproteobacteria;o__Burkholderiales;f__;g__Aquabacterium;s__Aquabacterium_commune
NCBI706196	85	69	25	22	27	24	41	62	32	146	62	47	56	15	18	k__Fungi;p__Ascomycota;c__Saccharomycetes;o__Saccharomycetales;f__Saccharomycetaceae;g__Saccharomyces;s__Saccharomyces_arboricola
NCBI706438	0	0	0	1	3	11	1	6	0	0	0	0	0	0	0	k__Bacteria;p__Actinomycetota;c__Actinomycetes;o__Actinomycetales;f__Actinomycetaceae;g__Actinomyces;s__Actinomyces_sp._oral_taxon_171

NCBI709810	0	0	0	2	0	10	0	0	0	0	0	0	0	0	0	k__Bacteria;p__Pseudomonadota;c__Alphaproteobacteria;o__Rhodospirillales;f__Azospirillaceae;g__Azospirillum;s__Azospirillum_sp._TSA2s
NCBI709883	0	0	0	0	0	57	0	0	0	0	0	0	0	0	0	k__Bacteria;p__Actinomycetota;c__Actinomycetes;o__Micromonosporales;f__Micromonosporaceae;g__Micromonospora;s__Micromonospora_zamorensis
NCBI710648	0	0	0	0	0	16	0	0	0	0	0	0	0	0	0	k__Bacteria;p__Pseudomonadota;c__Gammaproteobacteria;o__Moraxellales;f__Moraxellaceae;g__Acinetobacter;s__Acinetobacter_sp._Tol_5
NCBI712116	0	10	0	0	3	10	0	68	0	0	0	2	0	0	0	k__Bacteria;p__Actinomycetota;c__Actinomycetes;o__Actinomycetales;f__Actinomycetaceae;g__Actinomyces;s__Actinomyces_sp._oral_taxon_169
NCBI712122	0	0	0	3	1	13	1	0	0	0	0	21	0	0	0	k__Bacteria;p__Actinomycetota;c__Actinomycetes;o__Actinomycetales;f__Actinomycetaceae;g__Actinomyces;s__Actinomyces_sp._oral_taxon_414
NCBI712270	0	0	0	0	3	18	0	0	0	0	0	0	0	0	0	k__Bacteria;p__Actinomycetota;c__Actinomycetes;o__Mycobacteriales;f__Dietziaceae;g__Dietzia;s__Dietzia_sp._oral_taxon_368
NCBI712357	0	0	0	0	0	0	0	0	0	0	0	0	0	0	1	k__Bacteria;p__Fusobacteriota;c__Fusobacteriia;o__Fusobacteriales;f__Leptotrichiaceae;g__Leptotrichia;s__Leptotrichia_sp._oral_taxon_212
NCBI71237	0	0	0	0	0	0	0	0	0	0	0	1	0	0	0	k__Bacteria;p__Bacillota;c__Bacilli;o__Bacillales;f__Staphylococcaceae;g__Mammaliicoccus;s__Mammaliicoccus_vitulinus
NCBI712411	0	0	0	0	0	1	0	0	0	0	0	0	0	0	0	k__Bacteria;p__Actinomycetota;c__Coriobacteriia;o__Coriobacteriales;f__Atopobiaceae;g__Olsenella;s__Olsenella_sp._oral_taxon_807
NCBI712435	0	0	0	0	0	1	0	0	0	0	0	0	0	0	0	k__Bacteria;p__Bacteroidota;c__Bacteroidia;o__Bacteroidales;f__Porphyromonadaceae;g__Porphyromonas;s__Porphyromonas_sp._oral_taxon_275

NCBI712471	0	0	0	0	0	0	0	0	0	0	0	1	0	0	0	k__Bacteria;p__Bacteroidota;c__Bacteroidia;o__Bacteroidales;f__Prevotellaceae;g__Prevotella;s__Prevotella_sp._oral_taxon_475
NCBI712623	0	1	0	1	0	0	0	1	0	0	0	9	1	0	0	k__Bacteria;p__Bacillota;c__Bacilli;o__Lactobacillales;f__Streptococcaceae;g__Streptococcus;s__Streptococcus_sp._oral_taxon_061
NCBI712633	0	0	2	0	0	2	0	0	0	0	0	21	0	0	2	k__Bacteria;p__Bacillota;c__Bacilli;o__Lactobacillales;f__Streptococcaceae;g__Streptococcus;s__Streptococcus_sp._oral_taxon_431
NCBI712710	0	0	0	2	0	2	0	2	0	0	0	0	0	0	0	k__Bacteria;p__Bacteroidota;c__Bacteroidia;o__Bacteroidales;f__Tannerellaceae;g__Tannerella;s__Tannerella_serpentiformis
NCBI712982	0	0	0	0	0	0	0	0	0	0	0	1	0	0	0	k__Bacteria;p__Bacillota;c__Clostridia;o__Eubacteriales;f__Lachnospiraceae;g__;s__Lachnospiraceae_bacterium_oral_taxon_096
NCBI713030	0	0	1	0	0	0	0	0	0	0	0	0	0	0	0	k__Bacteria;p__Bacillota;c__Negativicutes;o__Selenomonadales;f__Selenomonadaceae;g__Selenomonas;s__Selenomonas_sp._oral_taxon_136
NCBI713051	0	1	0	0	0	0	0	0	0	0	0	0	0	0	0	k__Bacteria;p__Candidatus_Saccharibacteria;c__;o__;f__;g__;s__TM7_phylum_sp._oral_taxon_349
NCBI714067	0	0	0	0	0	0	0	0	1	0	0	3	0	0	0	k__Bacteria;p__Bacillota;c__Bacilli;o__Bacillales;f__Thermoactinomycetaceae;g__Kroppenstedtia;s__Kroppenstedtia_eburnea
NCBI71433	0	0	0	0	12	0	0	0	0	0	0	2	0	0	0	k__Bacteria;p__Pseudomonadota;c__Alphaproteobacteria;o__Hyphomicrobiales;f__Phyllobacteriaceae;g__Mesorhizobium;s__Mesorhizobium_amorphae
NCBI71452	0	0	0	0	0	0	1	0	0	0	0	3	0	0	0	k__Bacteria;p__Bacillota;c__Bacilli;o__Lactobacillales;f__Enterococcaceae;g__Enterococcus;s__Enterococcus_raffinosus

NCBI715451	0	0	0	0	0	24	0	0	0	0	0	0	0	0	0	k__Bacteria;p__Pseudomonadota;c__Gammaproteobacteria;o__Alteromonadales;f__Alteromonadaceae;g__Alteromonas;s__Alteromonas_naphthalenivorans
NCBI717785	0	0	0	0	0	0	0	0	0	1	0	0	0	0	0	k__Bacteria;p__Pseudomonadota;c__Alphaproteobacteria;o__Hyphomicrobiales;f__Hyphomicrobiaceae;g__Hyphomicrobium;s__Hyphomicrobium_sp._MC1
NCBI71999	0	5	9	12	13	71	2	9	0	0	1	8	0	128	5	k__Bacteria;p__Actinomycetota;c__Actinomycetes;o__Micrococcales;f__Micrococcaceae;g__Kocuria;s__Kocuria_palustris
NCBI72000	0	0	1	0	0	31	4	0	2	0	0	1	0	0	0	k__Bacteria;p__Actinomycetota;c__Actinomycetes;o__Micrococcales;f__Micrococcaceae;g__Kocuria;s__Kocuria_rhizophila
NCBI721785	956	1241	577	51	2901	32637	1237	77	472	148	171	525	198	103	182	k__Bacteria;p__Pseudomonadota;c__Betaproteobacteria;o__Burkholderiales;f__Comamonadaceae;g__Diaphorobacter;s__[Acidovorax]_ebreus
NCBI722472	6	0	0	0	6	0	0	0	0	1	0	0	0	0	0	k__Bacteria;p__Pseudomonadota;c__Alphaproteobacteria;o__Hyphomicrobiales;f__Nitrobacteraceae;g__Bradyrhizobium;s__Bradyrhizobium_lablabi
NCBI72557	0	0	0	0	0	32	0	0	0	0	0	0	0	0	0	k__Bacteria;p__Pseudomonadota;c__Betaproteobacteria;o__Burkholderiales;f__Alcaligenaceae;g__Achromobacter;s__Achromobacter_ruhlandii
NCBI726	0	0	0	1	0	0	0	0	0	0	0	0	0	0	0	k__Bacteria;p__Pseudomonadota;c__Gammaproteobacteria;o__Pasteurellales;f__Pasteurellaceae;g__Haemophilus;s__Haemophilus_haemolyticus
NCBI727	0	0	0	0	2	4	3	4	0	0	1	21	0	0	0	k__Bacteria;p__Pseudomonadota;c__Gammaproteobacteria;o__Pasteurellales;f__Pasteurellaceae;g__Haemophilus;s__Haemophilus_influenzae
NCBI728066	0	0	0	0	0	0	0	0	0	0	0	2	0	0	0	k__Bacteria;p__Actinomycetota;c__Actinomycetes;o__Micrococcales;f__Micrococcaceae;g__Pseudarthrobacter;s__Pseudarthrobacter_equi

NCBI729	0	0	2	9	1	5	1	0	0	1	0	39	0	0	0	k__Bacteria;p__Pseudomonadota;c__Gammaproteobacteria;o__Pasteurellales;f__Pasteurellaceae;g__Haemophilus;s__Haemophilus_parainfluenzae
NCBI730	1	0	0	1	0	0	0	0	0	0	0	0	0	0	0	k__Bacteria;p__Pseudomonadota;c__Gammaproteobacteria;o__Pasteurellales;f__Pasteurellaceae;g__Haemophilus;s__[Haemophilus]_ducreyi
NCBI732	0	0	0	2	0	0	0	0	0	0	0	0	0	0	0	k__Bacteria;p__Pseudomonadota;c__Gammaproteobacteria;o__Pasteurellales;f__Pasteurellaceae;g__Aggregatibacter;s__Aggregatibacter_aphrophilus
NCBI735	0	0	0	0	0	2	0	0	0	0	0	0	0	0	0	k__Bacteria;p__Pseudomonadota;c__Gammaproteobacteria;o__Pasteurellales;f__Pasteurellaceae;g__Haemophilus;s__Haemophilus_parahaemolyticus
NCBI73501	0	0	0	0	0	0	0	0	0	0	0	1	0	0	0	k__Fungi;p__Ascomycota;c__Sordariomycetes;o__Hypocreales;f__Cordycipitaceae;g__Cordyceps;s__Cordyceps_militaris
NCBI739141	37	31	41	205	330	363	59	11	24	0	0	0	0	6	0	k__Bacteria;p__Pseudomonadota;c__Alphaproteobacteria;o__Hyphomicrobiales;f__Methylobacteriaceae;g__Methylobacterium;s__Methylobacterium_sp._XJLW
NCBI742013	0	0	25	21	65	92	50	0	0	0	0	0	0	0	0	k__Bacteria;p__Pseudomonadota;c__Betaproteobacteria;o__Burkholderiales;f__Comamonadaceae;g__Delftia;s__Delftia_sp._Cs1-4
NCBI743009	0	0	0	0	0	0	0	0	0	0	0	1	0	0	0	k__Bacteria;p__Actinomycetota;c__Actinomycetes;o__Micrococcales;f__Microbacteriaceae;g__Microbacterium;s__Microbacterium_oryzae
NCBI74313	0	1	0	0	0	2	0	0	0	0	0	0	0	0	0	k__Bacteria;p__Pseudomonadota;c__Alphaproteobacteria;o__Caulobacterales;f__Caulobacteraceae;g__Brevundimonas;s__Brevundimonas_subvibrioides
NCBI74316	0	0	0	0	0	0	19	0	0	0	0	0	0	0	0	k__Bacteria;p__Pseudomonadota;c__Alphaproteobacteria;o__Caulobacterales;f__Caulobacteraceae;g__Brevundimonas;s__Brevundimonas_aurantiaca

NCBI74329	6	42	14	10	79	1100	48	3	7	4	4	14	0	3	3	k__Bacteria;p__Pseudomonadota;c__Alphaproteobacteria;o__Caulobacterales;f__Caulobacteraceae;g__Brevundimonas;s__Brevundimonas_mediterranea
NCBI74426	0	0	2	1	8	1125	1	7	0	0	1	311	0	0	0	k__Bacteria;p__Actinomycetota;c__Coriobacteriia;o__Coriobacteriales;f__Coriobacteriaceae;g__Collinsella;s__Collinsella_aerofaciens
NCBI745310	0	0	0	0	0	7	0	0	0	0	0	0	0	0	0	k__Bacteria;p__Pseudomonadota;c__Alphaproteobacteria;o__Sphingomonadales;f__Sphingomonadaceae;g__Sphingomonas;s__Sphingomonas_sp._MM-1
NCBI746128	0	1	4	0	0	6	0	0	0	0	0	5	0	0	0	k__Fungi;p__Ascomycota;c__Eurotiomycetes;o__Eurotiales;f__Aspergillaceae;g__Aspergillus;s__Aspergillus_fumigatus
NCBI747	462	351	317	1087	2441	2115	459	123	248	86	103	128	187	159	176	k__Bacteria;p__Pseudomonadota;c__Gammaproteobacteria;o__Pasteurellales;f__Pasteurellaceae;g__Pasteurella;s__Pasteurella_multocida
NCBI747082	0	10	0	0	5	0	16	10	4	6	0	11	0	0	6	k__Fungi;p__Ascomycota;c__Saccharomycetes;o__Saccharomycetales;f__Saccharomycetaceae;g__;s__uncultured_Pichia
NCBI747372	51	51	48	85	57	474	45	41	62	40	59	49	13	21	34	k__Bacteria;p__Bacillota;c__Tissierellia;o__Tissierellales;f__Peptoniphilaceae;g__Parvimonas;s__uncultured_Parvimonas_sp.
NCBI748280	0	0	0	0	0	4	0	0	0	0	0	0	0	0	0	k__Bacteria;p__Pseudomonadota;c__Betaproteobacteria;o__Neisseriales;f__Chromobacteriaceae;g__Pseudogulbenkiania;s__Pseudogulbenkiania_sp._NH8B
NCBI74829	72	30	26	3	154	1665	48	3	9	5	4	51	0	12	36	k__Bacteria;p__Pseudomonadota;c__Gammaproteobacteria;o__Pseudomonadales;f__Pseudomonadaceae;g__Stutzerimonas;s__Stutzerimonas_balearica
NCBI749	0	0	0	0	0	0	0	0	0	0	0	1	0	0	0	k__Bacteria;p__Pseudomonadota;c__Gammaproteobacteria;o__Pasteurellales;f__Pasteurellaceae;g__;s__[Pasteurella]_aerogenes

NCBI751585	0	0	0	1	0	10	0	0	0	0	0	2	0	0	1	k__Bacteria;p__Bacillota;c__Clostridia;o__Eubacteriales;f__Lachnospiraceae;g__Coprococcus;s__Coprococcus_sp._ART55/1
NCBI75385	0	0	0	0	4	23	0	2	0	0	0	1	0	0	0	k__Bacteria;p__Actinomycetota;c__Actinomycetes;o__Propionibacteriales;f__Nocardioidaceae;g__Micropruina;s__Micropruina_glycogenica
NCBI754476	0	0	0	0	0	1	0	0	0	0	0	0	0	0	0	k__Bacteria;p__Pseudomonadota;c__Gammaproteobacteria;o__Thiotrichales;f__Piscirickettsiaceae;g__Methylophaga;s__Methylophaga_nitratireducenticrescens
NCBI75553	0	0	1	0	0	0	0	0	0	0	0	0	0	0	0	k__Fungi;p__Ascomycota;c__Eurotiomycetes;o__Eurotiales;f__Aspergillaceae;g__Aspergillus;s__Aspergillus_viridinutans
NCBI75588	0	0	0	3	0	0	0	0	0	0	0	0	0	0	0	k__Bacteria;p__Pseudomonadota;c__Gammaproteobacteria;o__Pseudomonadales;f__Pseudomonadaceae;g__Pseudomonas;s__Pseudomonas_libanensis
NCBI756892	3	3	1	0	9	22	2	0	1	0	1	0	0	0	0	k__Bacteria;p__Pseudomonadota;c__Gammaproteobacteria;o__Moraxellales;f__Moraxellaceae;g__Acinetobacter;s__Acinetobacter_indicus
NCBI75750	0	0	3	18	38	16	17	3	5	0	4	41	0	0	1	k__Fungi;p__Ascomycota;c__Eurotiomycetes;o__Eurotiales;f__Aspergillaceae;g__Aspergillus;s__Aspergillus_sydowii
NCBI758826	0	0	0	0	0	12	1	0	0	0	0	0	0	0	0	k__Bacteria;p__Pseudomonadota;c__Betaproteobacteria;o__Burkholderiales;f__Comamonadaceae;g__Acidovorax;s__Acidovorax_radicis
NCBI759811	0	0	0	0	0	0	0	0	0	0	0	5	0	0	0	k__Bacteria;p__Bacillota;c__Bacilli;o__Bacillales;f__Bacillaceae;g__Lysinibacillus;s__Lysinibacillus_pakistanensis
NCBI759878	1	0	1	0	0	0	0	0	3	0	0	0	3	0	0	k__Bacteria;p__Chrysiogenota;c__Chrysiogenetes;o__Chrysiogenales;f__Chrysiogenaceae;g__Desulfurispirillum;s__uncultured_Desulfurispirillum_sp.

NCBI76115	0	0	0	0	0	5	0	0	0	0	0	0	0	0	0	k__Bacteria;p__Pseudomonadota;c__Betaproteobacteria;o__Rhodocyclales;f__Rhodocyclaceae;g__Aromatoleum;s__Aromatoleum_bremense
NCBI76116	0	0	0	0	0	1	0	0	0	0	0	0	0	0	0	k__Bacteria;p__Pseudomonadota;c__Betaproteobacteria;o__Rhodocyclales;f__Rhodocyclaceae;g__Aromatoleum;s__Aromatoleum_petrolei
NCBI76123	2	0	0	0	0	0	0	0	0	0	0	0	0	0	0	k__Bacteria;p__Bacteroidota;c__Bacteroidia;o__Bacteroidales;f__Prevotellaceae;g__Hoylesella;s__Hoylesella_enoeca
NCBI76259	44	36	29	19	111	800	45	1	9	7	7	16	10	9	5	k__Bacteria;p__Pseudomonadota;c__Betaproteobacteria;o__Rhodocyclales;f__Azonexaceae;g__Ferribacterium;s__Ferribacterium_limneticum
NCBI765821	0	0	0	0	0	0	0	0	0	0	0	1	0	0	0	k__Bacteria;p__Bacillota;c__Clostridia;o__Eubacteriales;f__Lachnospiraceae;g__Blautia;s__uncultured_Blautia_sp.
NCBI76731	6	4	0	0	3	138	1	0	0	1	0	0	0	0	0	k__Bacteria;p__Pseudomonadota;c__Betaproteobacteria;o__Burkholderiales;f__Sphaerotilaceae;g__Roseateles;s__Roseateles_depolymerans
NCBI76758	0	0	0	0	1	2	0	0	0	0	0	0	0	0	0	k__Bacteria;p__Pseudomonadota;c__Gammaproteobacteria;o__Pseudomonadales;f__Pseudomonadaceae;g__Pseudomonas;s__Pseudomonas_orientalis
NCBI76759	0	8	0	0	26	34	4	0	0	0	0	0	0	5	0	k__Bacteria;p__Pseudomonadota;c__Gammaproteobacteria;o__Pseudomonadales;f__Pseudomonadaceae;g__Pseudomonas;s__Pseudomonas_monteilii
NCBI76760	0	0	0	0	4	8	1	0	0	0	0	0	0	0	0	k__Bacteria;p__Pseudomonadota;c__Gammaproteobacteria;o__Pseudomonadales;f__Pseudomonadaceae;g__Pseudomonas;s__Pseudomonas_rhodesiae
NCBI76761	0	6	5	0	42	272	28	0	0	0	0	0	0	0	0	k__Bacteria;p__Pseudomonadota;c__Gammaproteobacteria;o__Pseudomonadales;f__Pseudomonadaceae;g__Pseudomonas;s__Pseudomonas_veronii

NCBI76773	3	3	3	10	53	40	0	10	0	0	0	1	1	1	2	k__Fungi;p__Basidiomycota;c__Malasseziomycetes;o__Malasseziales;f__Malasseziaceae;g__Malassezia;s__Malassezia_globosa
NCBI76775	2	2	8	100	38	141	6	9	5	0	4	40	1	4	8	k__Fungi;p__Basidiomycota;c__Malasseziomycetes;o__Malasseziales;f__Malasseziaceae;g__Malassezia;s__Malassezia_restricta
NCBI76777	0	0	0	0	0	0	0	0	1	0	1	0	0	0	0	k__Fungi;p__Basidiomycota;c__Malasseziomycetes;o__Malasseziales;f__Malasseziaceae;g__Malassezia;s__Malassezia_sympodialis
NCBI76832	0	0	0	0	0	5	0	0	0	0	0	28	0	0	0	k__Bacteria;p__Bacteroidota;c__Flavobacteriia;o__Flavobacteriales;f__Flavobacteriaceae;g__Myroides;s__Myroides_odoratimimus
NCBI76862	1	0	0	0	0	0	0	0	0	0	0	0	0	0	0	k__Bacteria;p__Actinomycetota;c__Actinomycetes;o__Micrococcales;f__Cellulomonadaceae;g__Cellulomonas;s__Cellulomonas_iranensis
NCBI76936	0	0	0	0	0	0	2	0	0	0	0	0	0	0	0	k__Bacteria;p__Campylobacterota;c__Epsilonproteobacteria;o__Campylobacterales;f__Helicobacteraceae;g__Helicobacter;s__Helicobacter_typhlonius
NCBI76947	0	0	1	0	0	10	0	0	0	0	0	0	0	0	0	k__Bacteria;p__Pseudomonadota;c__Alphaproteobacteria;o__Sphingomonadales;f__Sphingomonadaceae;g__Sphingobium;s__Sphingobium_herbicidovorans
NCBI77133	5706	5606	5198	22297	53379	125715	7765	1659	3587	838	1158	1392	1173	1158	974	k__Bacteria;p__;c__;o__;f__;g__;s__uncultured_bacterium
NCBI77635	0	0	0	1	0	2	0	0	0	0	0	0	0	0	0	k__Bacteria;p__Actinomycetota;c__Actinomycetes;o__Bifidobacteriales;f__Bifidobacteriaceae;g__Bifidobacterium;s__Bifidobacterium_subtile
NCBI78259	83	114	98	112	106	25	124	107	114	139	100	155	68	34	47	k__Bacteria;p__Actinomycetota;c__Actinomycetes;o__Bifidobacteriales;f__Bifidobacteriaceae;g__Scardovia;s__Scardovia_inopinata
NCBI7	133	147	136	727	160	233	271	46	111	21	24	23	20	38	33	k__Bacteria;p__Pseudomonadota;c__Gammaproteobacte

8327					8	9										ria;o__Pseudomonadales;f__Pseudomonadaceae;g__Pseudomonas;s__Pseudomonas_mosselii
NCBI78398	0	0	3	0	0	0	0	0	0	0	0	0	0	0	0	k__Bacteria;p__Pseudomonadota;c__Gammaproteobacteria;o__Enterobacterales;f__Pectobacteriaceae;g__Pectobacterium;s__Pectobacterium_odoriferum
NCBI78543	0	0	0	0	0	2	0	0	0	0	0	0	0	0	0	k__Bacteria;p__Pseudomonadota;c__Gammaproteobacteria;o__Pseudomonadales;f__Pseudomonadaceae;g__Pseudomonas;s__Pseudomonas_migulae
NCBI78587	15	5	4	0	40	253	15	0	6	2	5	2	1	2	0	k__Bacteria;p__Pseudomonadota;c__Alphaproteobacteria;o__Caulobacterales;f__Caulobacteraceae;g__Asticcacaulis;s__Asticcacaulis_excentricus
NCBI795324	0	0	0	0	0	25	0	0	0	0	0	0	0	0	0	k__Bacteria;p__;c__;o__;f__;g__;s__bacterium_enrichment_culture_clone_2b(2010)
NCBI795665	0	3	0	0	0	34	0	0	0	0	0	0	0	0	0	k__Bacteria;p__Pseudomonadota;c__Betaproteobacteria;o__Burkholderiales;f__Comamonadaceae;g__Hydrogenophaga;s__Hydrogenophaga_sp._PBC
NCBI79604	0	0	0	0	0	0	0	0	0	0	0	1	0	0	0	k__Bacteria;p__Actinomycetota;c__Coriobacteriia;o__Eggerthellales;f__Eggerthellaceae;g__Denitrobacterium;s__Denitrobacterium_detoxificans
NCBI797277	0	0	0	0	0	3	0	0	0	0	0	0	0	0	0	k__Bacteria;p__Pseudomonadota;c__Gammaproteobacteria;o__Pseudomonadales;f__Pseudomonadaceae;g__Halopseudomonas;s__Halopseudomonas_litoralis
NCBI79880	0	0	0	0	1	1	0	0	0	0	0	0	1	0	0	k__Bacteria;p__Bacillota;c__Bacilli;o__Bacillales;f__Bacillaceae;g__Alkalihalobacillus;s__Alkalihalobacillus_clausii
NCBI79885	0	0	0	1	2	0	0	0	0	0	0	0	0	0	0	k__Bacteria;p__Bacillota;c__Bacilli;o__Bacillales;f__Bacillaceae;g__Alkalihalophilus;s__Alkalihalophilus_pseudofirmus
NCBI80842	0	6	1	5	4	83	1	0	0	8	3	1	14	4	11	k__Bacteria;p__Pseudomonadota;c__Betaproteobacteria;o__Burkholderiales;f__Oxalobacteraceae;g__Herbaspirill

																um;s__Herbaspirillum_rubrisubalbicans
NCBI80866	25	25	30	163	342	375	187	0	45	8	0	19	23	4	0	k__Bacteria;p__Pseudomonadota;c__Betaproteobacteria;o__Burkholderiales;f__Comamonadaceae;g__Delftia;s__Delftia_acidovorans
NCBI80867	5	5	0	3	10	114	12	1	8	2	0	3	0	0	0	k__Bacteria;p__Pseudomonadota;c__Betaproteobacteria;o__Burkholderiales;f__Comamonadaceae;g__Acidovorax;s__Acidovorax_avenae
NCBI80869	11	10	0	0	21	345	18	0	5	0	0	3	0	3	0	k__Bacteria;p__Pseudomonadota;c__Betaproteobacteria;o__Burkholderiales;f__Comamonadaceae;g__Acidovorax;s__Acidovorax_citrulli
NCBI80878	0	0	0	0	66	536	63	0	0	0	0	0	0	37	0	k__Bacteria;p__Pseudomonadota;c__Betaproteobacteria;o__Burkholderiales;f__Comamonadaceae;g__Acidovorax;s__Acidovorax_temperans
NCBI80880	2	2	0	0	6	31	1	0	1	0	1	1	0	0	0	k__Bacteria;p__Pseudomonadota;c__Betaproteobacteria;o__Burkholderiales;f__Comamonadaceae;g__Hylemonella;s__Hylemonella_gracilis
NCBI81056	0	1	0	0	0	0	0	0	0	0	0	0	0	0	0	k__Fungi;p__Basidiomycota;c__Agaricomycetes;o__Polyporales;f__Phaeolaceae;g__Wolfiporia;s__Wolfiporia_cocos
NCBI81412	0	0	0	0	0	5	0	0	0	0	0	0	0	0	0	k__Bacteria;p__Synergistota;c__Synergistia;o__Synergistales;f__Synergistaceae;g__Aminomonas;s__Aminomonas_paucivorans
NCBI817	0	0	16	0	14	29	92	24	0	1	0	31	0	0	0	k__Bacteria;p__Bacteroidota;c__Bacteroidia;o__Bacteroidales;f__Bacteroidaceae;g__Bacteroides;s__Bacteroides_fragilis
NCBI818	5	0	7	1	3	94	47	1	0	0	0	62	0	0	0	k__Bacteria;p__Bacteroidota;c__Bacteroidia;o__Bacteroidales;f__Bacteroidaceae;g__Bacteroides;s__Bacteroides_thetaiotaomicron
NCBI820	0	0	4	5	45	190	204	13	3	0	0	114	0	0	0	k__Bacteria;p__Bacteroidota;c__Bacteroidia;o__Bacteroidales;f__Bacteroidaceae;g__Bacteroides;s__Bacteroides_

																uniformis
NCBI821	0	3	17	17	12	246	113	4	9	0	0	115	0	0	3	k__Bacteria;p__Bacteroidota;c__Bacteroidia;o__Bacteroidales;f__Bacteroidaceae;g__Phocaeicola;s__Phocaeicola_vulgatus
NCBI82135	0	0	0	0	0	3	0	0	0	0	0	2	0	0	0	k__Bacteria;p__Actinomycetota;c__Actinomycetes;o__Actinomycetales;f__Actinomycetaceae;g__Fannyhessea;s__Fannyhessea_vaginae
NCBI82268	0	0	0	0	0	0	0	1	0	0	0	0	0	0	0	k__Fungi;p__Chytridiomycota;c__Monoblepharidomycetes;o__Monoblepharidales;f__;g__Hyaloraphidium;s__Hyaloraphidium_curvatum
NCBI823	0	0	15	9	62	112	566	0	32	5	0	26	0	0	0	k__Bacteria;p__Bacteroidota;c__Bacteroidia;o__Bacteroidales;f__Tannerellaceae;g__Parabacteroides;s__Parabacteroides_distasonis
NCBI82348	0	0	0	1	0	0	0	0	0	0	0	0	0	0	0	k__Bacteria;p__Bacillota;c__Bacilli;o__Lactobacillales;f__Streptococcaceae;g__Streptococcus;s__Streptococcus_pluranimalium
NCBI82367	1	3	0	2	11	111	15	2	4	2	0	8	4	0	0	k__Bacteria;p__Pseudomonadota;c__Alphaproteobacteria;o__Rhodobacterales;f__Paracoccaceae;g__Paracoccus;s__Paracoccus_pantotrophus
NCBI82380	0	0	0	18	10	0	0	0	0	0	0	0	0	0	0	k__Bacteria;p__Actinomycetota;c__Actinomycetes;o__Micrococcales;f__Microbacteriaceae;g__Microbacterium;s__Microbacterium_oxydans
NCBI824	0	0	0	0	0	0	0	5	0	0	0	0	0	0	0	k__Bacteria;p__Campylobacterota;c__Epsilonproteobacteria;o__Campylobacterales;f__Campylobacteraceae;g__Campylobacter;s__Campylobacter_gracilis
NCBI82541	14	15	0	16	42	169	27	5	19	6	7	33	20	0	41	k__Bacteria;p__Pseudomonadota;c__Betaproteobacteria;o__Burkholderiales;f__Burkholderiaceae;g__Cupriavidus;s__Cupriavidus_gilardii
NCBI82633	78	147	62	0	206	3642	168	14	46	26	12	40	20	32	13	k__Bacteria;p__Pseudomonadota;c__Betaproteobacteria;o__Burkholderiales;f__Burkholderiaceae;g__Cupriavidus

															;s__Cupriavidus_pauculus
NCBI82688	104697	116721	112133	56389	60748	41287	155088	177883	173063	301944	251109	240973	135702	127916	128648	k__Bacteria;p__Bacillota;c__Bacilli;o__Lactobacillales;f__Lactobacillaceae;g__Liquorilactobacillus;s__Liquorilactobacillus_nagelii
NCBI82983	0	0	0	0	0	0	0	0	0	0	0	1	0	0	0	k__Bacteria;p__Pseudomonadota;c__Gammaproteobacteria;o__Enterobacterales;f__Hafniaceae;g__Obesumbacterium;s__Obesumbacterium_proteus
NCBI82996	0	0	0	0	0	1	0	0	0	0	0	0	0	0	0	k__Bacteria;p__Pseudomonadota;c__Gammaproteobacteria;o__Enterobacterales;f__Yersiniaceae;g__Serratia;s__Serratia_plymuthica
NCBI83263	0	0	0	0	0	5	0	0	0	0	0	0	0	0	0	k__Bacteria;p__Pseudomonadota;c__Alphaproteobacteria;o__Hyphomicrobiales;f__Phyllobacteriaceae;g__Aminobacter;s__Aminobacter_aminovorans
NCBI83291	0	0	0	57	0	1	0	0	0	0	0	2	0	0	0	k__Bacteria;p__Actinomycetota;c__Actinomycetes;o__Kitasatosporales;f__Streptomycetaceae;g__Streptomyces;s__Streptomyces_armeniacus
NCBI833	0	0	0	0	0	0	0	1	0	0	0	0	0	0	0	k__Bacteria;p__Fibrobacterota;c__Fibrobacteria;o__Fibrobacterales;f__Fibrobacteraceae;g__Fibrobacter;s__Fibrobacter_succinogenes
NCBI83406	1	0	0	0	0	1	0	0	0	0	0	0	0	0	0	k__Bacteria;p__Pseudomonadota;c__Gammaproteobacteria;o__;f__;g__;s__gamma_proteobacterium_HdN1
NCBI83427	2078	2688	2594	2890	3836	5940	4011	3136	2961	4423	2750	4005	1941	1374	1845	k__Bacteria;p__Bacillota;c__Bacilli;o__Lactobacillales;f__Streptococcaceae;g__Streptococcus;s__uncultured_Streptococcus_sp.
NCBI83428	1204	1870	1225	1777	1814	3446	2544	2012	1945	2777	2006	2229	1161	755	770	k__Bacteria;p__Bacillota;c__Bacilli;o__Bacillales;f__Bacillaceae;g__Bacillus;s__uncultured_Bacillus_sp.
NCBI83451	0	0	0	0	0	116	0	0	0	0	0	0	0	0	0	k__Bacteria;p__Myxococcota;c__Myxococcia;o__Myxococcales;f__Archangiaceae;g__Archangium;s__Archangium_violaceum

NCBI83453	0	0	0	0	0	5	0	0	0	0	0	0	0	0	0	k__Bacteria;p__Myxococcota;c__Myxococcia;o__Myxococcales;f__Archangiaceae;g__Melittangium;s__Melittangium_boletus
NCBI83526	0	0	0	0	0	0	0	0	0	1	0	0	0	0	0	k__Bacteria;p__Bacillota;c__Bacilli;o__Lactobacillales;f__Lactobacillaceae;g__Companilactobacillus;s__Companilactobacillus_paralimentarius
NCBI83627	0	0	0	1	0	1	0	0	0	0	0	0	0	0	0	k__Bacteria;p__Pseudomonadota;c__Alphaproteobacteria;o__Hyphomicrobiales;f__Nitrobacteraceae;g__Bradyrhizobium;s__Bradyrhizobium_genosp._B
NCBI83655	160	176	135	910	1994	3683	287	36	152	19	32	31	59	32	48	k__Bacteria;p__Pseudomonadota;c__Gammaproteobacteria;o__Enterobacterales;f__Enterobacteriaceae;g__Leclercia;s__Leclercia_adecarboxylata
NCBI83683	0	0	0	0	0	0	0	0	0	0	0	1	0	0	0	k__Bacteria;p__Bacillota;c__Bacilli;o__Lactobacillales;f__Lactobacillaceae;g__Lactobacillus;s__Lactobacillus_amylolyticus
NCBI837	0	0	0	1	1	0	0	0	0	0	0	3	0	0	0	k__Bacteria;p__Bacteroidota;c__Bacteroidia;o__Bacteroidales;f__Porphyromonadaceae;g__Porphyromonas;s__Porphyromonas_gingivalis
NCBI839	0	0	0	0	0	2	0	0	0	0	1	2	0	0	0	k__Bacteria;p__Bacteroidota;c__Bacteroidia;o__Bacteroidales;f__Prevotellaceae;g__Prevotella;s__Prevotella_ruminicola
NCBI84023	0	9	0	0	0	58	0	0	0	0	0	0	0	0	0	k__Bacteria;p__Bacillota;c__Clostridia;o__Eubacteriales;f__Clostridiaceae;g__Clostridium;s__Clostridium_autoethanogenum
NCBI84112	0	0	12	0	0	64	0	2	0	0	0	58	0	0	0	k__Bacteria;p__Actinomycetota;c__Coriobacteriia;o__Eggerthellales;f__Eggerthellaceae;g__Eggerthella;s__Eggerthella_lenta
NCBI84275	1	0	1	0	0	0	0	0	0	0	0	0	0	0	0	k__Fungi;p__Ascomycota;c__Dothideomycetes;o__Mycosphaerellales;f__Mycosphaerellaceae;g__Cercospora;s__Cercospora_kikuchii

NCBI84292	0	0	0	2	1	29	0	0	0	0	0	0	0	0	0	k__Bacteria;p__Actinomycetota;c__Actinomycetes;o__Micrococcales;f__Microbacteriaceae;g__Microbacterium;s__Microbacterium_chocolatum
NCBI84595	0	0	0	0	0	5	0	0	0	0	0	0	0	0	0	k__Bacteria;p__Actinomycetota;c__Actinomycetes;o__Mycobacteriales;f__Gordoniaceae;g__Gordonia;s__Gordonia_polyisoprenivorans
NCBI84751	0	0	0	0	0	0	0	0	0	0	0	0	0	1	0	k__Fungi;p__Basidiomycota;c__Ustilaginomycetes;o__Ustilaginales;f__Ustilaginaceae;g__Pseudozyma;s__Pseudozyma_flocculosa
NCBI84753	0	0	1	5	1	13	2	0	1	0	0	1	0	0	0	k__Fungi;p__Basidiomycota;c__Ustilaginomycetes;o__Ustilaginales;f__Ustilaginaceae;g__Moesziomyces;s__Moesziomyces_antarcticus
NCBI849058	0	0	0	0	0	0	3	0	0	0	0	8	4	0	0	k__Fungi;p__Ascomycota;c__Saccharomycetes;o__Saccharomycetales;f__Dipodascaceae;g__;s__uncultured_Yarrowia
NCBI850	0	0	0	0	0	0	3	0	0	0	0	0	0	0	2	k__Bacteria;p__Fusobacteriota;c__Fusobacteriia;o__Fusobacteriales;f__Fusobacteriaceae;g__Fusobacterium;s__Fusobacterium_mortiferum
NCBI85085	0	0	0	0	0	17	0	0	0	0	0	0	0	0	0	k__Bacteria;p__Actinomycetota;c__Actinomycetes;o__Micrococcales;f__Micrococcaceae;g__Pseudarthrobacter;s__Pseudarthrobacter_chlorophenolicus
NCBI853	0	1	8	17	27	718	67	20	0	0	1	530	0	0	0	k__Bacteria;p__Bacillota;c__Clostridia;o__Eubacteriales;f__Oscillospiraceae;g__Faecalibacterium;s__Faecalibacterium_prausnitzii
NCBI85336	0	0	0	0	0	15	0	0	0	0	0	0	0	0	0	k__Bacteria;p__Actinomycetota;c__Actinomycetes;o__Micrococcales;f__Micrococcaceae;g__Rothia;s__Rothia_nasimurium
NCBI85643	4	5	4	0	0	87	14	0	7	0	0	0	0	0	8	k__Bacteria;p__Pseudomonadota;c__Betaproteobacteria;o__Rhodocyclales;f__Zoogloeaceae;g__Thauera;s__Thauera_sp._MZ1T

NCBI856822	0	0	0	0	0	1	0	0	0	1	0	0	1	0	1	k__Fungi;p__Ascomycota;c__Eurotiomycetes;o__Chaetothyriales;f__Herpotrichiellaceae;g__Fonsecaea;s__Fonsecaea_nubica
NCBI85693	0	0	0	0	0	7	0	0	0	0	0	0	0	0	0	k__Bacteria;p__Actinomycetota;c__Actinomycetes;o__Mycobacteriales;f__Mycobacteriaceae;g__Mycolicibacterium;s__Mycolicibacterium_monacense
NCBI85698	1484	1517	1154	5643	14750	26201	2519	511	1256	261	246	466	896	774	1055	k__Bacteria;p__Pseudomonadota;c__Betaproteobacteria;o__Burkholderiales;f__Alcaligenaceae;g__Achromobacter;s__Achromobacter_xylosoxidans
NCBI857252	0	0	0	0	1	5	0	0	0	0	0	4	0	0	4	k__Bacteria;p__Pseudomonadota;c__Gammaproteobacteria;o__Pseudomonadales;f__Pseudomonadaceae;g__Halopseudomonas;s__Halopseudomonas_aestusnigri
NCBI857417	0	0	0	31	8	68	0	0	3	0	0	1	0	8	0	k__Bacteria;p__Actinomycetota;c__Actinomycetes;o__Micrococcales;f__Intrasporangiaceae;g__Janibacter;s__Janibacter_indicus
NCBI858422	0	0	0	0	64	0	0	0	0	1	0	1	0	0	0	k__Bacteria;p__Pseudomonadota;c__Alphaproteobacteria;o__Hyphomicrobiales;f__Nitrobacteraceae;g__Bradyrhizobium;s__Bradyrhizobium_sp._CCBAU_051011
NCBI858423	0	0	0	0	0	3	0	0	0	0	0	0	0	0	0	k__Bacteria;p__Pseudomonadota;c__Alphaproteobacteria;o__Hyphomicrobiales;f__Nitrobacteraceae;g__Bradyrhizobium;s__Bradyrhizobium_arachidis
NCBI859143	0	0	0	0	0	0	0	0	0	0	0	4	0	0	1	k__Bacteria;p__Bacillota;c__Bacilli;o__Bacillales;f__Bacillaceae;g__Cytobacillus;s__Cytobacillus_kochii
NCBI85929	0	0	0	0	1	0	0	0	0	0	0	1	0	0	0	k__Fungi;p__Ascomycota;c__Dothideomycetes;o__Mycosphaerellales;f__Mycosphaerellaceae;g__Sphaerulina;s__Sphaerulina_musiva
NCBI861	0	0	0	0	0	0	0	2	0	0	0	1	0	0	0	k__Bacteria;p__Fusobacteriota;c__Fusobacteriia;o__Fusobacteriales;f__Fusobacteriaceae;g__Fusobacterium;s__Fusobacterium_ulcerans
NCBI8	0	0	0	0	0	1	0	0	0	0	0	0	0	0	0	k__Bacteria;p__Pseudomonadota;c__Gammaproteobacte

6102																ria;o__Alteromonadales;f__Idiomarinaceae;g__Idiomarina;s__Idiomarina_abyssalis
NCBI861299	0	0	0	0	1	51	0	0	0	0	0	0	0	0	0	k__Bacteria;p__Gemmatimonadota;c__Gemmatimonadetes;o__Gemmatimonadales;f__Gemmatimonadaceae;g__Gemmatirosa;s__Gemmatirosa_kalamazoonensis
NCBI861445	3	0	0	9	8	0	1	0	0	0	3	0	0	0	0	k__Bacteria;p__Pseudomonadota;c__Gammaproteobacteria;o__Moraxellales;f__Moraxellaceae;g__Psychrobacter;s__Psychrobacter_sanguinis
NCBI86174	0	0	0	0	0	2	0	0	0	0	0	0	0	0	0	k__Bacteria;p__Pseudomonadota;c__Betaproteobacteria;o__Rhodocyclales;f__Zoogloeaceae;g__Thauera;s__Thauera_butanivorans
NCBI86185	0	0	0	0	0	0	0	0	0	0	1	0	0	0	0	k__Bacteria;p__Pseudomonadota;c__Gammaproteobacteria;o__Pseudomonadales;f__Pseudomonadaceae;g__Pseudomonas;s__Pseudomonas_lundensis
NCBI86188	46	29	40	241	563	833	58	13	0	0	15	13	0	21	0	k__Bacteria;p__Pseudomonadota;c__Gammaproteobacteria;o__Xanthomonadales;f__Xanthomonadaceae;g__Stenotrophomonas;s__Stenotrophomonas_geniculata
NCBI86265	0	0	0	0	0	0	2	0	0	0	0	0	0	0	0	k__Bacteria;p__Pseudomonadota;c__Gammaproteobacteria;o__Pseudomonadales;f__Pseudomonadaceae;g__Pseudomonas;s__Pseudomonas_thivervalensis
NCBI862751	0	0	1	0	0	0	3	0	0	4	1	7	6	13	11	k__Bacteria;p__Actinomycetota;c__Actinomycetes;o__Kitasatosporales;f__Streptomycetaceae;g__Streptomyces;s__Streptomyces_sp._SirexAA-E
NCBI863	0	0	0	0	0	2	0	0	0	0	0	0	0	0	0	k__Bacteria;p__Bacillota;c__Clostridia;o__Eubacteriales;f__Syntrophomonadaceae;g__Syntrophomonas;s__Syntrophomonas_wolfei
NCBI86332	0	0	0	0	0	0	0	1	0	0	0	0	0	0	0	k__Bacteria;p__Bacillota;c__Clostridia;o__Eubacteriales;f__Eubacteriales_Family_XIII._Incertae_Sedis;g__Mogibacterium;s__Mogibacterium_pumilum

NCBI863372	170	413	134	404	909	12161	215	156	103	886	559	132	1413	643	2322	k__Bacteria;p__Pseudomonadota;c__Betaproteobacteria;o__Burkholderiales;f__Oxalobacteraceae;g__Herbaspirillum;s__Herbaspirillum_huttiense
NCBI863934	28	121	13	0	222	3469	143	0	52	29	14	73	30	20	46	k__Bacteria;p__Pseudomonadota;c__Alphaproteobacteria;o__Sphingomonadales;f__Sphingomonadaceae;g__Sphingobium;s__Sphingobium_sp._PNB
NCBI864828	7	0	21	1	14	8	0	0	0	0	0	0	0	0	0	k__Bacteria;p__Pseudomonadota;c__Betaproteobacteria;o__Burkholderiales;f__Oxalobacteraceae;g__Pseudoduganella;s__Pseudoduganella_umbonata
NCBI86664	1	0	0	0	0	0	0	0	0	0	0	0	0	0	0	k__Bacteria;p__Bacillota;c__Bacilli;o__Bacillales;f__Bacillaceae;g__Priestia;s__Priestia_flexa
NCBI871742	0	0	0	0	0	3	0	0	0	0	0	0	0	0	0	k__Bacteria;p__Pseudomonadota;c__Betaproteobacteria;o__Burkholderiales;f__Oxalobacteraceae;g__Pseudoduganella;s__Pseudoduganella_flava
NCBI87229	0	0	0	0	0	0	0	0	0	5	3	3	0	5	7	k__Fungi;p__Ascomycota;c__Leotiomycetes;o__Helotiales;f__Sclerotiniaceae;g__Botrytis;s__Botrytis_porri
NCBI876	0	0	0	1	0	0	0	2	0	0	0	0	0	0	0	k__Bacteria;p__Thermodesulfobacteriota;c__Desulfovibrionia;o__Desulfovibrionales;f__Desulfovibrionaceae;g__Desulfovibrio;s__Desulfovibrio_desulfuricans
NCBI876091	0	0	0	0	0	0	6	0	0	0	0	0	0	0	0	k__Bacteria;p__Bacillota;c__Clostridia;o__Eubacteriales;f__Oscillospiraceae;g__Oscillibacter;s__uncultured_Oscillibacter_sp.
NCBI876364	63	56	22	20	196	1005	70	26	31	31	11	17	32	0	0	k__Bacteria;p__Pseudomonadota;c__Betaproteobacteria;o__Burkholderiales;f__Burkholderiaceae;g__Cupriavidus;s__Cupriavidus_sp._USMAA2-4
NCBI87883	0	5	0	8	7	76	0	0	5	0	1	7	0	0	2	k__Bacteria;p__Pseudomonadota;c__Betaproteobacteria;o__Burkholderiales;f__Burkholderiaceae;g__Burkholderia;s__Burkholderia_multivorans
NCBI879274	56	58	39	2	181	2157	87	5	14	3	6	64	22	0	23	k__Bacteria;p__Pseudomonadota;c__Alphaproteobacteria;o__Hyphomicrobiales;f__Rhizobiaceae;g__Shinella;s__

															_Shinella_sp._HZN7
NCBI881	0	0	0	0	0	0	0	0	0	0	0	8	0	0	0	k__Bacteria;p__Thermodesulfobacteriota;c__Desulfovibrionia;o__Desulfovibrionales;f__Desulfovibrionaceae;g__Desulfovibrio;s__Desulfovibrio_vulgaris
NCBI88190	0	0	0	0	0	0	0	0	0	0	0	0	0	0	1	k__Bacteria;p__Deinococcota;c__Deinococci;o__Thermales;f__Thermaceae;g__Thermus;s__Thermus_antranikianii
NCBI88233	30	28	36	39	24	24	43	28	38	31	33	50	34	18	32	k__Bacteria;p__Bacillota;c__Bacilli;o__Lactobacillales;f__Lactobacillaceae;g__Lacticaseibacillus;s__Lacticaseibacillus_manihotivorans
NCBI88688	1	1	0	1	1	31	0	0	0	0	0	0	0	1	0	k__Bacteria;p__Pseudomonadota;c__Alphaproteobacteria;o__Caulobacterales;f__Caulobacteraceae;g__Caulobacter;s__Caulobacter_segnis
NCBI888845	0	2	0	0	0	0	0	0	0	0	0	0	0	0	0	k__Bacteria;p__Myxococcota;c__Myxococcia;o__Polyangiales;f__Polyangiaceae;g__Minicystis;s__Minicystis_rosea
NCBI89059	1	0	0	0	0	0	1	1	0	0	0	0	0	0	0	k__Bacteria;p__Bacillota;c__Bacilli;o__Lactobacillales;f__Lactobacillaceae;g__Ligilactobacillus;s__Ligilactobacillus_acidipiscis
NCBI89152	0	0	0	0	0	1	0	0	0	0	0	0	0	0	0	k__Bacteria;p__Bacillota;c__Clostridia;o__Eubacteriales;f__Peptostreptococcaceae;g__Peptacetobacter;s__Peptacetobacter_hiranonis
NCBI891974	0	0	0	0	0	7	0	0	0	0	0	0	0	0	0	k__Bacteria;p__Pseudomonadota;c__Gammaproteobacteria;o__Enterobacterales;f__Enterobacteriaceae;g__;s__Plautia_stali_symbiont
NCBI89584	0	0	0	0	22	0	0	0	4	0	0	0	0	0	0	k__Bacteria;p__Pseudomonadota;c__Alphaproteobacteria;o__Rhodospirillales;f__Acetobacteraceae;g__Gluconacetobacter;s__Gluconacetobacter_liquefaciens
NCBI9	1	1	3	2	11	4	5	1	2	0	0	0	2	1	0	k__Bacteria;p__Pseudomonadota;c__Gammaproteobacte

																ria;o__Enterobacterales;f__Erwiniaceae;g__Buchnera;s__Buchnera_aphidicola
NCBI901	0	0	0	0	0	2	0	0	0	0	0	2	0	0	0	k__Bacteria;p__Thermodesulfobacteriota;c__Desulfovibrionia;o__Desulfovibrionales;f__Desulfovibrionaceae;g__Desulfovibrio;s__Desulfovibrio_piger
NCBI904039	0	0	0	0	2	0	0	0	0	0	0	0	0	0	0	k__Bacteria;p__Actinomycetota;c__Actinomycetes;o__Micrococcales;f__Micrococcaceae;g__Arthrobacter;s__Arthrobacter_sp._NEB_688
NCBI90426	0	0	0	0	0	0	0	0	0	0	0	1	0	0	0	k__Archaea;p__Euryarchaeota;c__Methanomicrobia;o__Methanotrichales;f__Methanotrichaceae;g__Methanothrix;s__Methanothrix_sp.
NCBI904291	0	0	0	0	0	6	0	0	0	0	0	0	0	0	0	k__Bacteria;p__Actinomycetota;c__Actinomycetes;o__Micrococcales;f__Microbacteriaceae;g__Microbacterium;s__Microbacterium_sediminis
NCBI905	0	0	0	0	0	0	0	0	0	0	0	1	0	0	0	k__Bacteria;p__Bacillota;c__Negativicutes;o__Acidaminococcales;f__Acidaminococcaceae;g__Acidaminococcus;s__Acidaminococcus_fermentans
NCBI907	0	0	0	1	0	7	0	1	0	0	0	3	0	0	0	k__Bacteria;p__Bacillota;c__Negativicutes;o__Veillonellales;f__Veillonellaceae;g__Megasphaera;s__Megasphaera_elsdenii
NCBI907061	0	9	0	0	0	0	0	0	0	0	0	0	0	0	0	k__Bacteria;p__Pseudomonadota;c__Alphaproteobacteria;o__Sphingomonadales;f__Sphingomonadaceae;g__Sphingomonas;s__Sphingomonas_sp._Fr1
NCBI912	0	0	0	0	0	4	0	0	1	0	0	0	0	0	0	k__Bacteria;p__Pseudomonadota;c__Alphaproteobacteria;o__Hyphomicrobiales;f__Nitrobacteraceae;g__Nitrobacter;s__Nitrobacter_hamburgensis
NCBI912630	2	0	0	0	1	0	0	0	0	0	0	0	0	0	0	k__Bacteria;p__Actinomycetota;c__Actinomycetes;o__Micrococcales;f__Microbacteriaceae;g__Microbacterium;s__Microbacterium_sp._LKL04

NCBI912801	0	0	0	0	0	0	0	0	0	0	0	5	0	0	0	k__Bacteria;p__Actinomycetota;c__Actinomycetes;o__Mycobacteriales;f__Dietziaceae;g__Dietzia;s__Dietzia_sp._DQ12-45-1b
NCBI913	0	0	0	0	0	2	0	0	0	0	0	0	1	0	0	k__Bacteria;p__Pseudomonadota;c__Alphaproteobacteria;o__Hyphomicrobiales;f__Nitrobacteraceae;g__Nitrobacter;s__Nitrobacter_winogradskyi
NCBI915174	0	0	0	0	0	9	0	0	0	0	0	0	0	0	0	k__Bacteria;p__Bacillota;c__Clostridia;o__Eubacteriales;f__;g__;s__Clostridiales_bacterium_40-4c
NCBI91915	0	0	0	0	0	0	0	0	0	0	0	0	0	1	1	k__Bacteria;p__Pseudomonadota;c__Alphaproteobacteria;o__Rhodospirillales;f__Acetobacteraceae;g__Asaia;s__Asaia_bogorensis
NCBI920	0	0	0	0	1	0	0	0	0	0	1	0	0	0	0	k__Bacteria;p__Pseudomonadota;c__Acidithiobacillia;o__Acidithiobacillales;f__Acidithiobacillaceae;g__Acidithiobacillus;s__Acidithiobacillus_ferrooxidans
NCBI921	1	0	0	0	0	0	0	0	0	0	0	0	0	0	0	k__Bacteria;p__Pseudomonadota;c__Alphaproteobacteria;o__Hyphomicrobiales;f__Xanthobacteraceae;g__Starkeya;s__Starkeya_novella
NCBI924	0	0	0	0	1	0	0	0	0	0	0	0	0	0	0	k__Bacteria;p__Pseudomonadota;c__Betaproteobacteria;o__Nitrosomonadales;f__Thiobacillaceae;g__Thiobacillus;s__Thiobacillus_sp.
NCBI926	0	0	0	0	0	1	0	0	0	0	0	0	0	0	0	k__Bacteria;p__Pseudomonadota;c__Betaproteobacteria;o__Burkholderiales;f__;g__Thiomonas;s__Thiomonas_intermedia
NCBI92637	0	0	0	0	0	1	0	0	0	0	0	0	0	0	0	k__Fungi;p__Ascomycota;c__Sordariomycetes;o__Hypocreales;f__Clavicipitaceae;g__Metarhizium;s__Metarhizium_acridum
NCBI92645	0	0	0	0	0	23	0	0	0	0	1	1	2	0	1	k__Bacteria;p__Pseudomonadota;c__Betaproteobacteria;o__Burkholderiales;f__Oxalobacteraceae;g__Herbaspirillum;s__Herbaspirillum_frisingense
NCBI9	5	13	4	0	17	292	9	0	1	0	3	3	0	0	0	k__Bacteria;p__Pseudomonadota;c__Alphaproteobacteri

3064																a;o__Sphingomonadales;f__Sphingomonadaceae;g__Sphingomonas;s__Sphingomonas_koreensis
NCBI93218	0	0	0	0	0	6	0	0	0	0	0	0	0	0	0	k__Bacteria;p__Pseudomonadota;c__Betaproteobacteria;o__Burkholderiales;f__Burkholderiaceae;g__Pandoraea;s__Pandoraea_apista
NCBI93220	0	0	0	0	0	13	0	0	0	0	0	0	0	0	6	k__Bacteria;p__Pseudomonadota;c__Betaproteobacteria;o__Burkholderiales;f__Burkholderiaceae;g__Pandoraea;s__Pandoraea_pnomenusa
NCBI93221	9	17	1	23	20	11	17	5	11	28	31	30	309	281	347	k__Bacteria;p__Pseudomonadota;c__Betaproteobacteria;o__Burkholderiales;f__Burkholderiaceae;g__Pandoraea;s__Pandoraea_pulmonicola
NCBI93222	0	0	0	0	0	3	0	0	0	0	0	0	0	0	0	k__Bacteria;p__Pseudomonadota;c__Betaproteobacteria;o__Burkholderiales;f__Burkholderiaceae;g__Pandoraea;s__Pandoraea_sputorum
NCBI935199	2	0	0	0	0	0	0	0	0	0	0	0	0	0	0	k__Bacteria;p__Actinomycetota;c__Actinomycetes;o__Mycobacteriales;f__Nocardiaceae;g__Rhodococcus;s__Rhodococcus_sp._p52
NCBI936337	0	0	0	0	2	0	0	0	0	0	0	0	0	0	0	k__Bacteria;p__Actinomycetota;c__Actinomycetes;o__Micrococcales;f__Microbacteriaceae;g__Microbacterium;s__Microbacterium_amylolyticum
NCBI938155	252	209	246	596	518	236	191	205	245	206	153	247	97	110	152	k__Bacteria;p__Bacillota;c__Bacilli;o__Lactobacillales;f__Lactobacillaceae;g__Companilactobacillus;s__Companilactobacillus_futsaii
NCBI94132	0	1	0	0	0	20	0	0	0	0	0	0	0	0	0	k__Bacteria;p__Pseudomonadota;c__Betaproteobacteria;o__Burkholderiales;f__Comamonadaceae;g__Ramlibacter;s__Ramlibacter_tataouinensis
NCBI945844	0	0	0	0	33	36	7	1	0	0	0	0	0	0	0	k__Bacteria;p__Pseudomonadota;c__Betaproteobacteria;o__Burkholderiales;f__Oxalobacteraceae;g__Massilia;s__Massilia_oculi
NCBI9	1	0	0	0	0	2	0	0	11	0	6	0	0	0	0	k__Bacteria;p__Pseudomonadota;c__Alphaproteobacteri

4625																a;o__Hyphomicrobiales;f__Brucellaceae;g__Brucella;s__Brucella_intermedia
NCBI946333	0	0	0	0	0	9	0	0	0	0	0	0	0	0	0	k__Bacteria;p__Pseudomonadota;c__Betaproteobacteria;o__Burkholderiales;f__;g__Rhizobacter;s__Rhizobacter_gummiphilus
NCBI946435	0	0	0	0	0	0	0	0	0	0	2	0	0	0	0	k__Bacteria;p__Bacillota;c__Bacilli;o__Bacillales;f__Staphylococcaceae;g__Jeotgalicoccus;s__Jeotgalicoccus_sp._ATCC_8456
NCBI948311	0	0	0	0	0	0	0	0	0	0	0	0	1	0	0	k__Fungi;p__Ascomycota;c__Sordariomycetes;o__Hypocreales;f__Nectriaceae;g__Fusarium;s__Fusarium_proliferatum
NCBI948519	0	0	0	0	0	3	25	0	0	0	0	0	0	0	0	k__Bacteria;p__Pseudomonadota;c__Gammaproteobacteria;o__Aeromonadales;f__Aeromonadaceae;g__Aeromonas;s__Aeromonas_rivipollensis
NCBI95485	0	0	0	0	0	13	0	0	0	0	0	0	0	0	0	k__Bacteria;p__Pseudomonadota;c__Betaproteobacteria;o__Burkholderiales;f__Burkholderiaceae;g__Burkholderia;s__Burkholderia_stabilis
NCBI95486	0	0	0	0	8	10	2	0	0	0	0	0	0	0	0	k__Bacteria;p__Pseudomonadota;c__Betaproteobacteria;o__Burkholderiales;f__Burkholderiaceae;g__Burkholderia;s__Burkholderia_cenocepacia
NCBI95641	0	0	0	0	0	1	0	0	0	0	0	0	0	0	0	k__Bacteria;p__Pseudomonadota;c__Gammaproteobacteria;o__Methylococcales;f__Methylococcaceae;g__Methylotuvimicrobium;s__Methylotuvimicrobium_buryatense
NCBI96344	16	7	6	0	7	166	16	0	1	0	3	0	0	0	2	k__Bacteria;p__Pseudomonadota;c__Betaproteobacteria;o__Burkholderiales;f__Burkholderiaceae;g__Cupriavidus;s__Cupriavidus_oxalaticus
NCBI964	6	1	0	2	1	48	0	0	0	7	2	2	2	0	7	k__Bacteria;p__Pseudomonadota;c__Betaproteobacteria;o__Burkholderiales;f__Oxalobacteraceae;g__Herbaspirillum;s__Herbaspirillum_seropedicae
NCBI9	0	0	0	3	2	229	1	0	0	0	0	9	0	0	0	k__Bacteria;p__Bacillota;c__Bacilli;o__Lactobacillales;f

7478																__Lactobacillaceae;g__Limosilactobacillus;s__Limosilactobacillus_mucosae
NCBI97707	489	529	318	0	999	17615	531	0	234	0	0	0	0	96	0	k__Bacteria;p__Pseudomonadota;c__Alphaproteobacteria;o__Hyphomicrobiales;f__Rhizobiaceae;g__;s__arsenite-oxidising_bacterium_NT-25
NCBI980427	0	0	0	3	1	3	1	0	4	0	0	2	0	0	0	k__Bacteria;p__Deinococcota;c__Deinococci;o__Deinococcales;f__Deinococcaceae;g__Deinococcus;s__Deinococcus_wulumuqiensis
NCBI981386	0	0	0	0	2	6	0	0	0	0	0	0	0	5	1	k__Bacteria;p__Pseudomonadota;c__Alphaproteobacteria;o__Hyphomicrobiales;f__Brucellaceae;g__Brucella;s__Brucella_vulpis
NCBI984962	0	0	0	0	0	0	2	0	0	0	0	1	0	0	0	k__Fungi;p__Basidiomycota;c__Agaricomycetes;o__Russulales;f__Bondarzewiaceae;g__Heterobasidion;s__Heterobasidion_irregulare
NCBI986	0	0	0	0	4	16	4	0	0	0	0	0	0	0	0	k__Bacteria;p__Bacteroidota;c__Flavobacteriia;o__Flavobacteriales;f__Flavobacteriaceae;g__Flavobacterium;s__Flavobacterium_johnsoniae
NCBI99007	0	0	0	0	0	1	0	0	0	0	0	0	0	0	0	k__Archaea;p__Thermoproteota;c__Thermoprotei;o__Thermoproteales;f__Thermoproteaceae;g__Pyrobaculum;s__Pyrobaculum_oguniense
NCBI991904	0	1	0	0	2	44	1	0	0	0	0	0	0	0	0	k__Bacteria;p__Pseudomonadota;c__Alphaproteobacteria;o__Rhodobacterales;f__Paracoccaceae;g__Polymorphum;s__Polymorphum_gilvum
NCBI996	1	0	0	0	0	0	0	0	3	1	0	0	0	0	1	k__Bacteria;p__Bacteroidota;c__Flavobacteriia;o__Flavobacteriales;f__Flavobacteriaceae;g__Flavobacterium;s__Flavobacterium_columnare

Supplementary Table S3 The volatile aroma compounds detected and measured in the samples collected from strawberry juice fermented with water kefir grains.

Aroma compounds	Retention time (min)	Relative contents (>1%)of volatile aroma compounds of fermented strawberry juice (%)					
		0h	12h	24h	36h	48h	60h
Esters							
1-Butanol, 3-methyl-, acetate	5.707	ND	ND	ND	ND	0.708±0.034	1.176±0.131
Octanoic acid, methyl ester	12.586	ND	0.859±0.037	1.101±0.105	1.364±0.114	0.730±0.045	0.633±0.032
Acetic acid, hexyl ester	9.404	ND	1.494±0.116	1.427±0.098	0.857±0.028	0.419±0.025	0.321±0.018
Octanoic acid, ethyl ester	14.674	ND	6.702±0.403	17.173±1.132	15.168±1.093	12.868±0.98	9.728±1.006
Decanoic acid, methyl ester	18.158	ND	1.264±0.086	2.816±0.157	3.228±0.226	3.688±0.268	3.777±0.198
2-Propenoic acid, 3-phenyl-, methyl ester, (E)-	19.776	8.245±0.578	2.718±0.083	1.155±0.062	1.078±0.085	0.611±0.028	0.620±0.071
Ethyl 9-decenoate	19.826	ND	ND	1.871±0.104	2.173±0.117	2.117±0.201	3.553±0.235
Propanoic acid, ethyl ester	20.100	ND	ND	ND	ND	1.461	ND
Decanoic acid, ethyl ester	20.026	ND	4.195±0.236	24.546±1.346	28.926±2.119	34.162±2.612	35.049±3.028
Dodecanoic acid, methyl ester	23.209	ND	0.440±0.031	1.421±0.097	0.959±0.103	1.111±0.058	1.125±0.072
Dodecanoic acid, ethyl ester	24.862	ND	1.183±0.0677	8.378±0.369	6.573±0.258	8.088±0.481	8.882±0.612
2,2,4-Trimethyl-1,3-pentanediol diisobutyrate	24.974	1.449±0.063	0.285±0.021	0.273±0.012	0.114±0.006	0.163±0.020	0.185±0.015
γ.-Dodecalactone	26.854	2.920±0.106	0.890±0.045	0.514±0.028	0.452±0.018	0.256±0.029	0.308±0.017
Hexadecanoic acid, methyl ester	33.466	1.734±0.076	1.595±0.0837	0.993±0.064	0.764±0.045	0.933±0.049	1.071±0.072
Ethyl 9-hexadecenoate	34.781	ND	0.314±0.031	1.133±0.087	0.928±0.064	1.243±0.073	1.013±0.058
Hexadecanoic acid, ethyl ester	35.287	ND	1.153±0.062	1.365±0.058	1.400±0.103	2.073±0.116	3.042±0.172
9,12-Octadecadienoic acid (Z,Z)-, methyl ester	37.159	1.645±0.123	2.437±0.147	ND	0.854±0.042	0.990±0.061	1.166±0.085

Aroma compounds	Retention time (min)	Relative contents (>1%)of volatile aroma compounds of fermented strawberry juice (%)					
		0h	12h	24h	36h	48h	60h
9,12,15-Octadecatrienoic acid, methyl ester, (Z,Z,Z)-	37.267	1.531±0.074	2.140±0.117	0.962±0.102	0.538±0.029	0.625±0.031	ND
Linoleic acid ethyl ester	38.167	ND	3.207±0.156	2.573±0.134	2.237±0.162	2.737±0.118	3.967±0.204
(E)-9-Octadecenoic acid ethyl ester	38.249	ND	ND	ND	0.554±0.015	0.697±0.042	1.973±0.118
9,12,15-Octadecatrienoic acid, ethyl ester, (Z,Z,Z)-	38.262	ND	2.567±0.068	1.612±0.113	1.599±0.063	1.278±0.047	1.045±0.052
Volatile acids							
Oxalic acid	2.072	ND	1.862±0.114	ND	ND	ND	ND
Formic acid	2.099	ND	1.292±0.071	ND	ND	ND	ND
Acetic acid	6.750	1.498±0.11	0.479±0.012	0.534±0.016	0.610±0.020	0.76±0.026	1.709±0.217
Pentanoic acid	8.936	1.947±0.117	8.012±0.472	ND	0.445±0.008	1.688±0.124	0.377±0.005
Octanoic acid	14.194	1.220±0.056	ND	ND	ND	ND	ND
Butanoic acid-3-methyl-	14.679	ND	ND	ND	ND	ND	2.408±0.115
n-Decanoic acid	19.630	ND	2.063±0.164	2.290±0.118	2.379±0.138	1.644±0.145	ND
Alcohols							
1-Pentanol	3.261	ND	ND	ND	ND	2.439±0.097	ND
2,3-Butanediol	3.304	ND	ND	1.159±0.088	0.106±0.004	ND	ND
1-Hexanol	5.645	ND	3.295±0.241	ND	ND	ND	ND
Phenylethyl Alcohol	12,293	ND	2.201±0.185	1.686±0.140	1.831±0.114	1.076±0.082	1.267±0.048
dl-Isopulegol	16.475	1.099±0.073	ND	ND	ND	ND	ND
Aldehydes							
Benzaldehyde	7.980	2.835±0.116	ND	ND	ND	ND	ND
2-Octenal, (E)-	10.667	2.049±0.124	0.158±0.016	ND	ND	ND	ND
Nonanal	12.006	1.933±0.146	0.213±0.041	0.278±0.011	ND	0.149	ND
2-Nonenal, (E)-	13.615	1.847±0.117	0.181±0.008	0.147±0.010	0.074±0.003	0.070±0.006	ND
Benzaldehyde, 3,4-dimethyl-	15.224	3.347±0.148	ND	0.216±0.012	0.288±0.015	0.086±0.010	0.253±0.016
2-Decenal, (E)-	16.476	1.857±0.141	0.392±0.028	0.258±0.017	0.149±0.009	0.111±0.005	0.090±0.003

Aroma compounds	Retention time (min)	Relative contents (>1%)of volatile aroma compounds of fermented strawberry juice (%)					
		0h	12h	24h	36h	48h	60h
5-Hydroxypentanal	26.854	1.181±0.102	ND	ND	ND	ND	ND
Ketones							
2-Octanone	8.811	1.781±0.114	ND	ND	ND	ND	ND
Acetophenone	10.957	0.576±0.023	ND	ND	ND	ND	ND
Alkanes							
Cyclopentane	3.327	ND	1.239±0.116	ND	ND	ND	ND
Cyclobutane, 1,1,2,3,3-pentamethyl-	5.747	ND	ND	1.847±0.137	ND	ND	ND
Benzene, 1,1'-(1,2-cyclobutanediyl)bis-, Cis-	6.128	ND	ND	ND	1.830±0.202	ND	ND
Styren	6.153	ND	22.540±3.077	5.452±0.461	2.412±0.182	ND	0.976±0.062
Naphthalene, 1,2,3,4-tetrahydro-1,6,8- Trimethyl-	15.092	0.855±0.048	0.619±0.038	0.213±0.014	0.737±0.027	0.355±0.021	0.203±0.011
Naphthalene, 1,2,3,4-tetrahydro-1,1,6-trimethyl-	17.291	8.019±0.517	2.647±0.244	0.824±0.051	0.304±0.018	0.212±0.011	0.456±0.032
1H-Indene, 2,3-dihydro-1,1,5,6-tetramethyl-	18.916	2.595±0.146	0.880±0.061	ND	ND	0.188±0.021	ND
Naphthalene, decahydro-2,2-dimethyl-	22.938	2.277±0.155	ND	ND	ND	ND	0.311±0.018
Volatile phenols							
2,4-Di-tert-butylphenol	23.038±1.982	11.441±0.834	3.461±0.268	1.524±0.117	1.688±0.145	0.778±0.048	1.824±0.161

Note: 0h, 12h, 24h,36h,48h and 60h, represent the samples collected from different fermented time, respectively. The data were presented as mean ± SD.

Supplementary Table S4 The statistical analysis VIP values of the discriminant metabolites for each sample of the strawberry juice with water kefir grains

Var ID (Primary)	M2.VIP[4+0+1]	2.57059 * M2.VIP[4]cvSE
Propanoic acid, ethyl ester	1.45988	3.10224
Butanedioic acid, phenyl-	1.45988	3.10224
1-Pentanol	1.45988	3.10224
Butanoic acid, 3-methyl-	1.31927	2.69169
Acetic acid	1.17071	1.09608
(E)-9-Octadecenoic acid ethyl ester	1.16775	1.29276
1-Butanol, 3-methyl-, acetate	1.15638	0.910271
9,12-Octadecadienoic acid (Z,Z)-, methyl ester	1.12607	1.07186
n-Decanoic acid	1.12505	0.83401
2,3-Butanediol	1.11351	2.60554
Pentanoic acid	1.09646	1.70832
Oxalic acid	1.07469	2.10365
Formic acid	1.07469	2.10365
1-Hexanol	1.07469	2.10365
Cyclopentane	1.07469	2.10365
Acetic acid, hexyl ester	1.05374	0.460136
Styrene	1.05123	1.61411
Cyclobutane, 1,1,2,3,3-pentamethyl-	1.05082	2.98568
Hexadecanoic acid, ethyl ester	1.02437	0.687671
Linoleic acid ethyl ester	0.996079	0.702037
9,12,15-Octadecatrienoic acid, methyl ester, (Z,Z,Z)	0.991796	0.793057
Ethyl 9-decenoate	0.991404	0.566764
Octanoic acid, ethyl ester	0.98829	0.173532
9,12,15-Octadecatrienoic acid, ethyl ester, (Z,Z,Z)-	0.976293	0.669706
Phenylethyl Alcohol	0.965173	0.628213
Octanoic acid, methyl ester	0.939756	0.658385
Decanoic acid, ethyl ester	0.933836	0.256506
Ethyl 9-hexadecenoate	0.932554	0.196779
Hexadecanoic acid, methyl ester	0.928572	0.525811
Dodecanoic acid, ethyl ester	0.92289	0.257688
Decanoic acid, methyl ester	0.907989	0.197067
Naphthalene, decahydro-2,2-dimethyl-	0.891585	1.32064
Benzaldehyde, 3,4-dimethyl-	0.890834	1.49659

Dodecanoic acid, methyl ester	0.881804	0.445802
Octanoic acid	0.881422	1.52902
1-Hexanol, 5-methyl-2-(1-methylethyl)-, acetate	0.881422	1.52902
Cyclobutaneethanol, .beta.-methylene-	0.881422	1.52902
dl-Isopulegol	0.881422	1.52902
Benzaldehyde	0.881422	1.52902
5-Hydroxypentanal	0.881422	1.52902
2-Octanone	0.881422	1.52902
Acetophenone	0.881422	1.52902
2-Nonenal, (E)-	0.879501	1.50094
2,4-Di-tert-butylphenol	0.879106	1.03488
Nonanal	0.877089	1.50186
1H-Indene, 2,3-dihydro-1,1,5,6-tetramethyl-	0.876374	0.76314
γ-Dodecalactone	0.876187	1.1228
2-Octenal, (E)-	0.875209	1.38725
2-Propenoic acid, 3-phenyl-, methyl ester, (E)-	0.873363	0.99451
Naphthalene, 1,2,3,4-tetrahydro-1,1,6-trimethyl-	0.871803	0.918856
2-Decenal, (E)-	0.818269	0.901888
2,2,4-Trimethyl-1,3-pentanediol diisobutyrate	0.792052	0.853902
Naphthalene, 1,2,3,4-tetrahydro-1,6,8-trimethyl-	0.739109	1.19368
Benzene, 1,1'-(1,2-cyclobutanediyl)bis-, cis-	0.530564	3.46968
